# Synthesis of aza-quaternary centers *via* Pictet–Spengler reactions of ketonitrones†

**DOI:** 10.1039/d1sc00882j

**Published:** 2021-03-16

**Authors:** Tessa Lynch-Colameta, Sarah Greta, Scott A. Snyder

**Affiliations:** Department of Chemistry, University of Chicago 5735 S. Ellis Avenue Chicago IL 60637 USA sasnyder@uchicago.edu

## Abstract

Despite the array of advances that have been made in Pictet–Spengler chemistry, particularly as it relates to the synthesis of β-carboline derivatives of both natural and designed origin, the ability to use such reactions to generate aza-quaternary centers remains limited. Herein, we report a simple procedure that enables the synthesis of a variety of such products by harnessing the distinct reactivity profiles of ketonitrones as activated by commercially available acyl chlorides. Notably, the reaction process is mild, fast, and high-yielding (54–97%) for a diverse collection of substrates, including some typically challenging ones, such as indole cores with electron-deficient substituents. In addition, by deploying an acyl bromide in combination with a thiourea promoter, a catalytic, asymmetric version has been established, leading to good levels of enantioselectivity (up to 83% ee) for several ketonitrones. Finally, the resultant N–O bonds within the products can also be functionalized in several unique ways, affording valuable complementarity to existing Pictet–Spengler variants based on the use of imines.

## Abstract 2

Despite the array of advances that have been made in Pictet–Spengler chemistry, particularly as it relates to the synthesis of β-carboline derivatives of both natural and designed origin, the ability to use such reactions to generate aza-quaternary centers remains limited.

## Graphical abstract

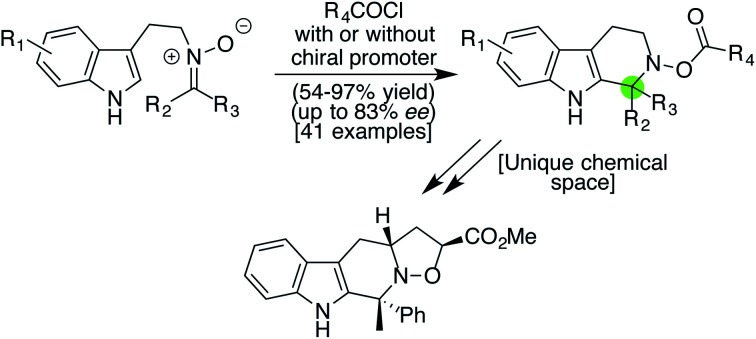

## Introduction

**Scheme 1:**
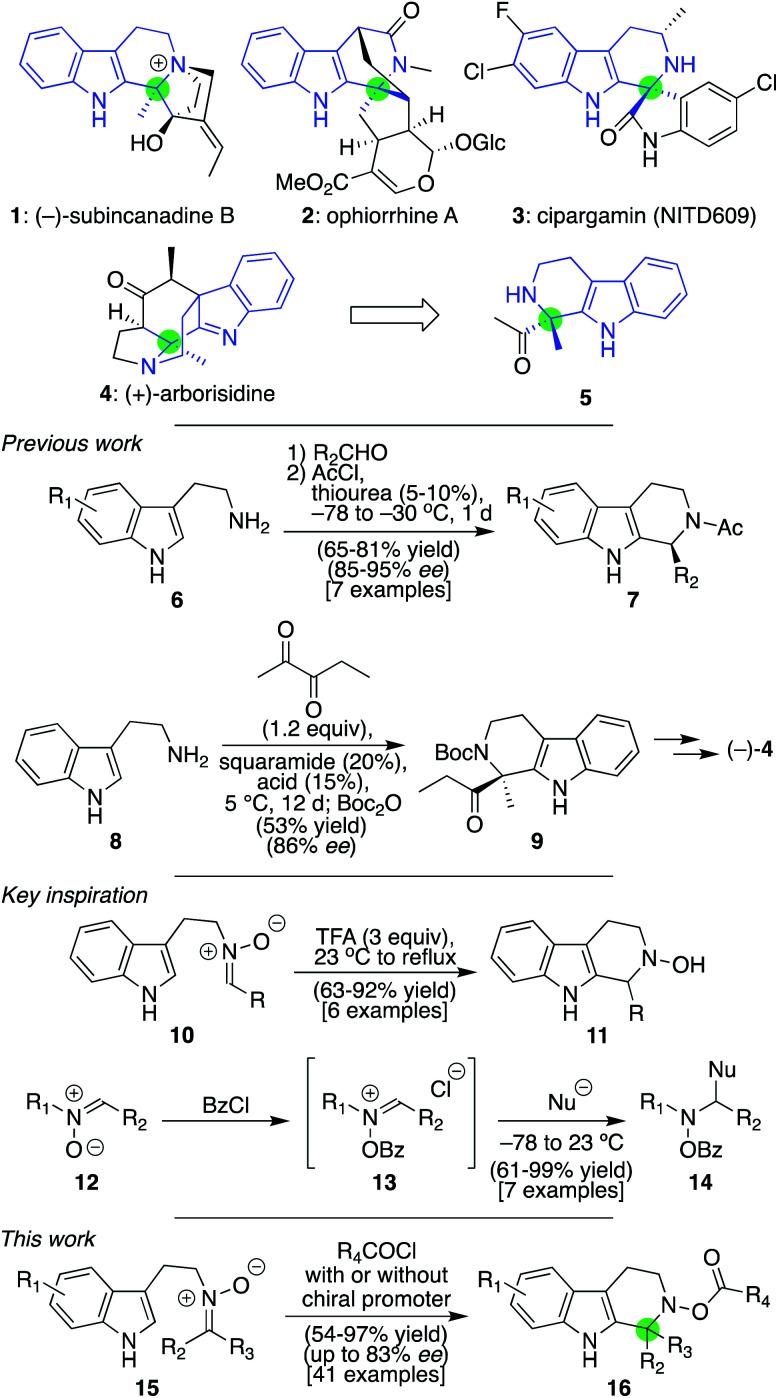
Selected structures of natural products and drugs containing a shared β-carboline framework (colored in blue) with an aza-quaternary center (colored in green), key precedents for asymmetric Pictet–Spengler chemistry leading to such products, and the inspiration and development of a unique approach to access the desired framework using ketonitrones leading to both racemic and enantioenriched syntheses of **16**.

Given that the tetrahydro-β-carboline core is a privileged scaffold found in natural products, folk medicines, and mainline pharmaceutical agents, numerous methods have been developed for its synthesis.^1^ The most powerful of these, arguably, is the Pictet–Spengler reaction,^2,3^ especially given recent advances in asymmetric variants promoted by hydrogen bonding catalysts such as phosphoric acids and thioureas,^4^ as exemplified through the conversion of **6** to **7** (Scheme 1).^4*a*^ Less common, though no less valuable, are β-carbolines that possess an aza-quaternary center, with **1–4** being representative cases.^5^ Methods to access these materials, by contrast, are far less developed.^4*b*,6,7^ Indeed, in our own recent total synthesis of (+)-**4**, we could not directly form the lone chiral center of **5** as a single enantiomer from achiral materials using tryptamine and a diketone reactant *via* a Pictet–Spengler reaction.^5*h*^ Nonetheless, in a recent synthesis of the same target by Zhu and co-workers, extensive investigations ultimately identified the means to achieve an enantioselective synthesis of **9** (in 86% ee), despite requiring prolonged reaction times (12 d).^5*i*^ Only one other report of an asymmetric variant involving non-spirocyclic 1,2-dicarbonyls is known, and it required stoichiometric amounts of a chiral promoter for success.^6*k*^ Herein, we delineate a distinct approach for generating such aza-quaternary centers through Pictet–Spengler chemistry, both in racemic and asymmetric form, by taking advantage of the unique chemistry of nitrones. As illustrated by >40 examples, the identified process leads to the rapid and high-yielding synthesis of materials with a diverse array of electronic properties, most of which were previously unsynthesized through Pictet–Spengler reactions. Furthermore, many of these products proved accessible in enantioenriched form (up to 83% ee) when our core procedure was modified slightly with catalytic amounts of an appropriate chiral promoter. And, as demonstrated by some concluding examples, the presence of the resultant N–O bond in the products could be leveraged in several distinct ways to access additional complexity that would be difficult to obtain from materials fashioned through a traditional Pictet–Spengler approach.

Attracted by this general challenge and drawing inspiration from our recent work using nitrones to forge functionalized piperidines through Mannich-type chemistry,^8^ we wondered if such species might also be of service here. Although a limited number of nitrone-based Pictet–Spengler reactions have been reported,^9^ nearly every example utilizes aldonitrones (such as **10**) under conditions that tend to require strong acid and/or high temperatures to promote successful cyclization to **11**;^9*f*^ in the case of reported asymmetric reactions, superstoichiometric amounts of chiral promoters have been needed.^9*i*–*k*^ To the best of our knowledge, just one example of a successful reaction with a ketonitrone, in which acetone was utilized to generate that species, has been reported in a racemic format.^9*d*^ This dearth of examples may reflect that ketonitrones are generally less reactive than their aldonitrone counterparts. However, it has been shown in separate studies that if nitrones more generally, such as **12**, are converted into their corresponding *N*-acyloxyiminium ions (**13**), their reactivity is greatly enhanced and can undergo nucleophilic attack to generate materials of type **14**.^10^ We anticipated that such a mode of activation might enable Pictet–Spengler chemistry to proceed when it otherwise would not. In addition, the added acyloxy group might also at the same time confer structural advantages that would render an asymmetric version of the reaction easier to achieve than standard imine-based Pictet–Spengler reactions.

## Results and discussion

Our studies began with efforts to affect a racemic version of the process, utilizing ketonitrone **19** (Table 1) as our inaugural starting material. This compound was readily prepared on gram scale over the course of three steps from tryptophol (**17**, see ESI† for details), and is an easily purifiable, bench-stable solid. In short order, we found that if this compound was combined with 1.05 equiv. of benzoyl chloride in the presence of 4 Å molecular sieves in CH_2_Cl_2_ at 23 °C, the desired product (**20a**) was obtained in 86% yield after just 30 min; X-ray crystallographic analysis confirmed its identity. Of note, the presence of molecular sieves was crucial to prevent the hydrolysis of the *N*-acyloxyiminium species formed *in situ*, which led to diminished yields.^11^ Pleasingly, screening of numerous acyl chlorides showed the reaction process to be just as facile irrespective of their steric bulk or electronic properties (entries 2–5), with phenylacetyl chloride providing the highest yield at 95%. We view such breadth of success with the activating partner as an attractive feature given that this group can serve as a protective device (with different means of deprotection and/or stability to subsequent reaction conditions as a result) and might provide critical functionality that could enable chiral differentiation.

**Table 1:** Synthesis of nitrone **19** and screening of acyl chlorides to determine optimized conditions to generate **20**^a^

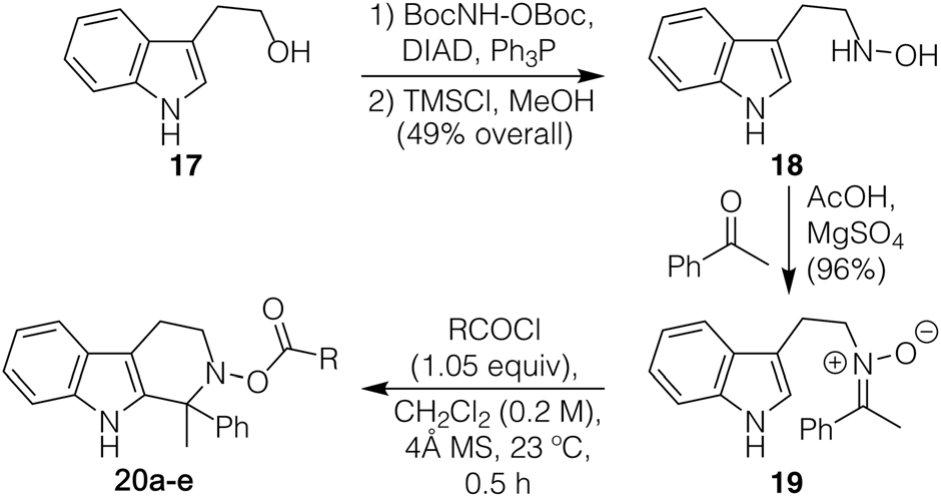			
Entry	R	Product	Yield [%]
1	Ph	**20a** [X-ray]	86
2	Me	**20b**	86
3	*t*-Bu	**20c**	88
4	OMe	**20d**	87
5	CH_2_Ph	**20e**	95

a Final reactions were performed with **19** (0.25 mmol) under argon.

**Scheme 2:**
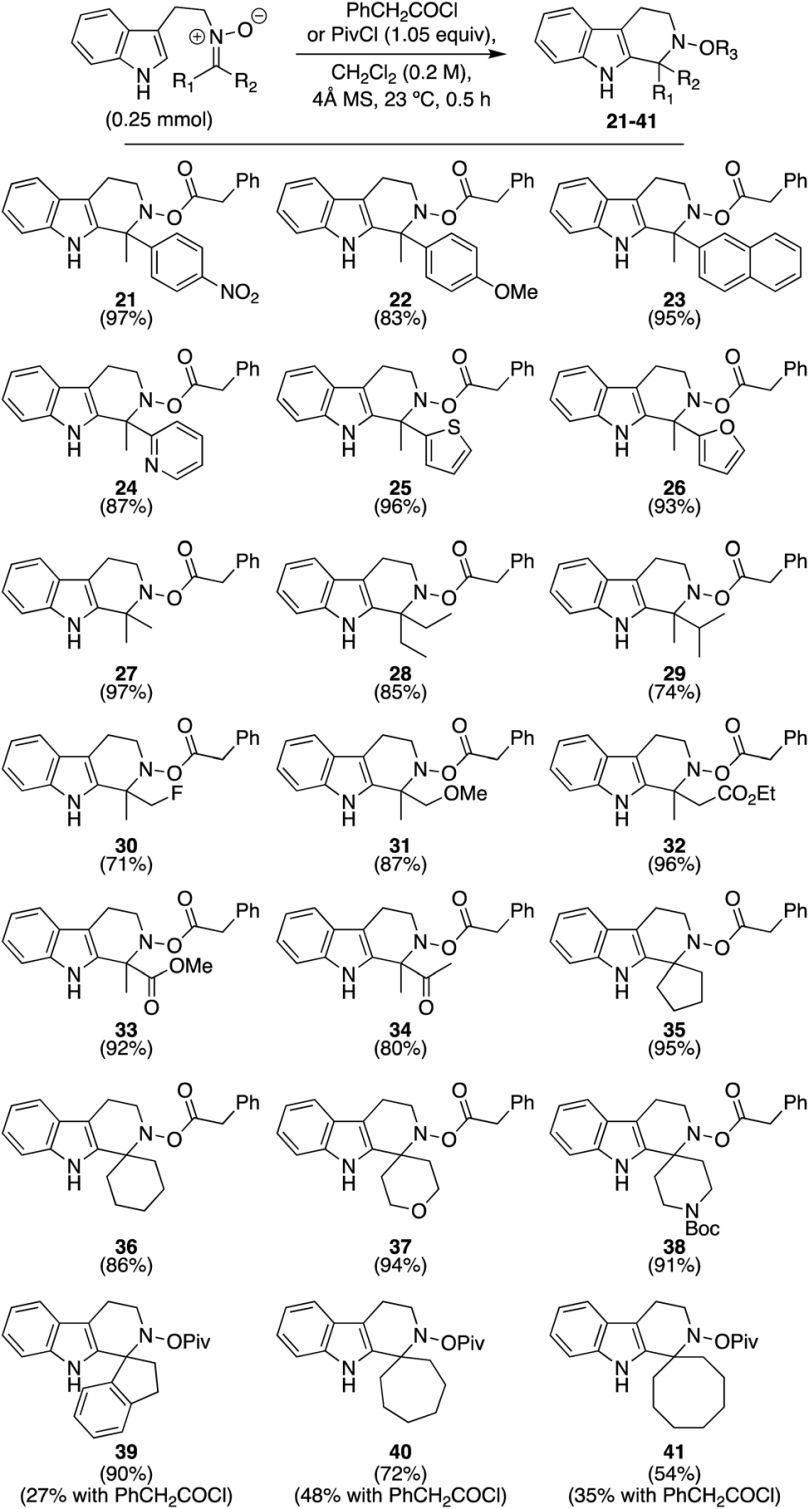
Exploration of substrate scope with various ketonitrones.

With this initial condition screen completed, we then turned our attention to exploring the overall scope. Scheme 2 presents our efforts to evaluate distinct ketonitrones generated from hydroxylamine precursor **18** (*cf.*Table 1). Some nitrones were synthesized as inseparable *E*-/*Z*-mixtures, as denoted within the ESI,† but this element did not affect the outcome of these racemic reactions. As shown, a wide range of both ketonitrones and their respective products were readily synthesized, with yields for the final compounds ranging from 71–97% for **21–38** when phenylacetyl chloride was used as the activating species. These products include aryl and heteroaryl groups (**21–26**), with those rings containing electron-deficient groups affording elevated yields. Simple alkyl substituents could also be incorporated (**27–30**), yet the amount of steric hindrance is relevant to the degree of success as revealed by the decreased yield obtained for **29** (74%). Pleasingly, ether, ester, and ketone-substituted nitrones worked as well (**31–34**),^12^ as did materials leading to spirocyclic products (**35–38**). However, we found for the latter category that when substrates had extended ring sizes or an indanone-based system, the yields obtained for their products were significantly lower (<50%); compounds of this type are known to be challenging in the imine variant of this, and related, reactions.^13^ Surmising that increased hydrolysis of the *N*-acyloxyiminium species over prolonged reaction times might be the culprit,^11^ we switched the activating species to a more sterically encumbered acyl chloride (*i.e.* pivaloyl chloride) in hopes it would still enable sufficient intramolecular cyclization while slowing down the intermolecular decomposition/hydrolysis pathways. As proposed, the yields for **39–41** were significantly enhanced. Therefore, we recommend this activating species in particular for ketonitrone starting materials that are ineffective with smaller acyl groups. Notably, the only real limitations identified from this initial screen were that ketonitrones possessing very sterically encumbered (*i.e.* if R_1_ or R_2_ = *t*-butyl) or electron-deficient groups (*i.e.* if R_1_ or R_2_ = CF_3_) could not be prepared; as long as the ketonitrone could be made, a successful Pictet–Spengler cyclization was observed in every case.

**Scheme 3:**
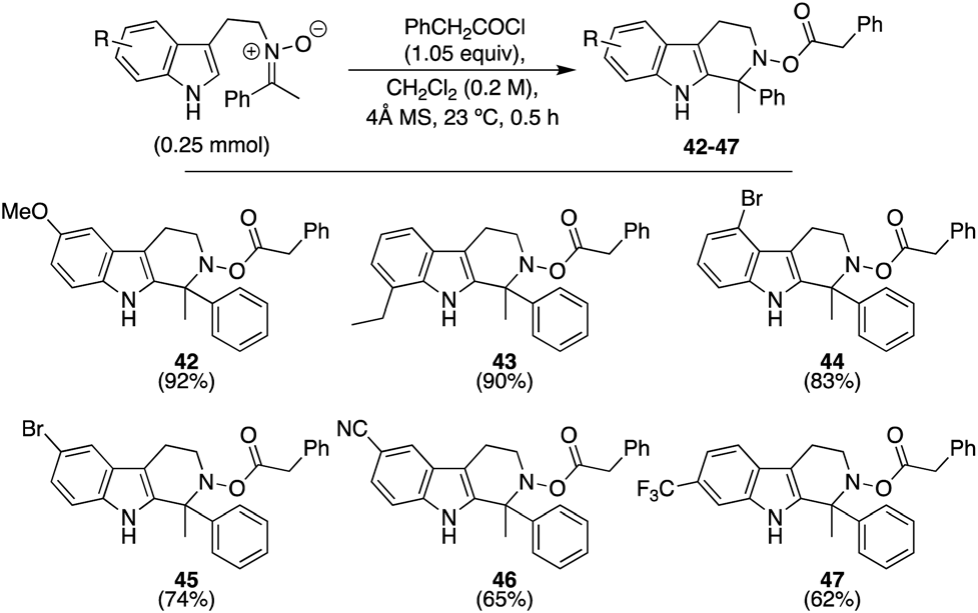
Continued exploration of scope using substituted indoles.

We next examined the extent to which the electronic properties of the indole core could be modulated. As seen in Scheme 3, and as expected, those substrates possessing electron-donating groups proceeded smoothly to afford **42** and **43** in high yield (92 and 90%, respectively). To our delight, the reaction also tolerated both weak and strongly electron-withdrawing groups at either the 4-, 5-, or 6-positions, with those substrates possessing the stronger withdrawing groups only leading to an ∼10% decrease in yield overall, to afford **44–47** in 62–83% yield. In general, these latter two substrates reflect examples that have not yet been demonstrated to succeed with reasonable yield in standard, imine-based Pictet–Spengler reactions,^14^ highlighting how activated nitrones provide enhanced electrophilicity that can broaden scope.

Given this range of prepared racemic products possessing aza-quaternary centers, our next focus was on rendering this process asymmetric. We began by testing thioureas as enantioselective promoters, given their broad application in asymmetric catalysis, particularly as relates to anion-binding-mediated enantioselection.^15^ Our first effective hit came with one particular thiourea (**48**)^16,17^ using substrate **19**, 4 Å molecular sieves, and benzoyl chloride as the activating group in CH_2_Cl_2_ at a concentration of 0.015 M at −78 °C (Table 2, entry 1). In this case, while the product yield was reasonable (79%), only modest enantioselectivity (51% ee) was observed. Though many acyl chlorides were subsequently screened (see ESI† Section), none provided substantial improvements over this lead. However, a solvent screen showed that if the reaction medium was changed to toluene (entry 2), enantioselectivity could be enhanced (to 64% ee) at the expense of yield (52%). A screening of additional catalysts (not shown, see ESI†) was able to increase the enantioselectivity to 81% ee, but could not provide any solution to the low yield of the reaction. Encouraged by these results and the documented effect that different anions can have on binding,^15^ we next changed the activating species to benzoyl bromide, and finally saw retention in yields with increases in enantioselectivity (75% and 83% ee, respectively, entry 3). We believe this finding to be significant, in that it implies our reaction may not be going through a traditional anion-binding mechanism since a switch to bromide under such a manifold would normally lead to a decrease in chiral selection.^4*b*^ Hence, we propose that the catalyst itself could be interacting with the carbonyl of the *N*-acyloxyiminium species, with the phenyl group of the activating group likely being involved as well.^18^ Following extensive optimization, the reaction afforded the best results when performed using 10 mol% catalyst loading of thiourea **48** in toluene at −78 °C at a concentration of 0.01 M (entry 6).

**Table 2:** Optimization of an asymmetric version of the reaction promoted by thiourea **48**^a^

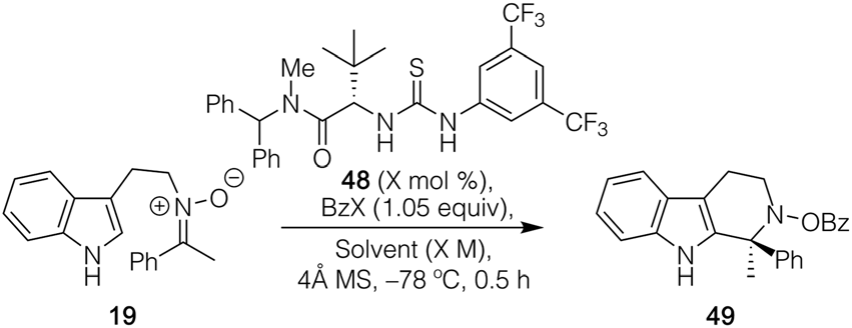						
Entry	Solvent	BzX	M (mol L^−1^)	(X mol%)	Yield [%]	ee [%]
1	CH_2_Cl_2_	BzCl	0.015	20 mol%	79	51
2	Toluene	BzCl	0.015	20 mol%	52	64
3	Toluene	BzBr	0.015	20 mol%	75	83
4	Toluene	BzBr	0.015	10 mol%	83	83
5	Toluene	BzBr	0.020	10 mol%	83	78
6	Toluene	BzBr	0.010	10 mol%	85	84

a Final reactions were performed with **19** (0.09 mmol) under argon.

**Scheme 4:**
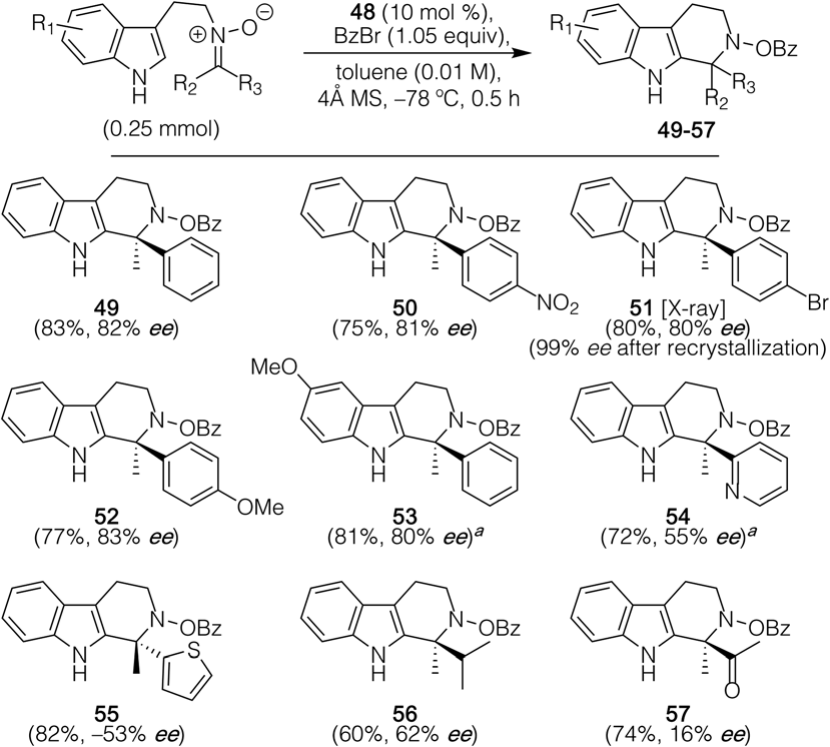
Substrate scope for the asymmetric variant of the reaction. ^*a*^ Performed in toluene : CH_2_Cl_2_ (4 : 1).

As indicated in Scheme 4, these conditions enabled effective chiral selection for ketonitrones possessing a variety of appended benzene rings, with **49–53** being obtained in good yield and enantioselectivities (80–83% ee); an X-ray crystal structure of **51** provided the means to establish their absolute configuration, with the other products being assigned based on analogy.^19^ Impressively, this crystallization afforded **51** with near optical purity (99% ee). These results in terms of enantioselectivities for these substrates are entirely in line with the best previous literature precedent (Scheme 1, product **9**, 86% ee) for aza-quaternary center generation, where only a single substrate was probed, yet with much higher yields and significantly faster reaction times. In addition, substrates possessing a heterocyclic substituent (pyridine or thiophene) also worked well, though with decreased enantioselectivity (55% ee and 53% ee, respectively). Unlike with the racemic variant of the reaction, critical for the results of all these asymmetric cases was that their ketonitrones could be obtained as single regioisomers. By contrast, most of the alkyl–alkyl substituted nitrones that were synthesized exist as inseparable mixtures of *E*-/*Z*-isomers. For one such material where separation could be accomplished, only moderate levels of enantiocontrol (62% ee) were observed with the resultant product (**56**). Still, we were able to show with this substrate that *E*-/*Z*-isomers gave approximately equal, but opposite, enantioselectivities under the optimized conditions.^20^ The presence of an additional carbonyl group (as in **57**) proved detrimental, likely due to the competing racemic background reaction, noting that all reactions appear to suffer from some degree of background reaction in eroding enantioselection. Substrates with strongly electron-withdrawing groups on the indole core (not shown) were incompatible with the asymmetric reaction conditions, affording only the undesired hydrolysis side-products.

**Scheme 5:**
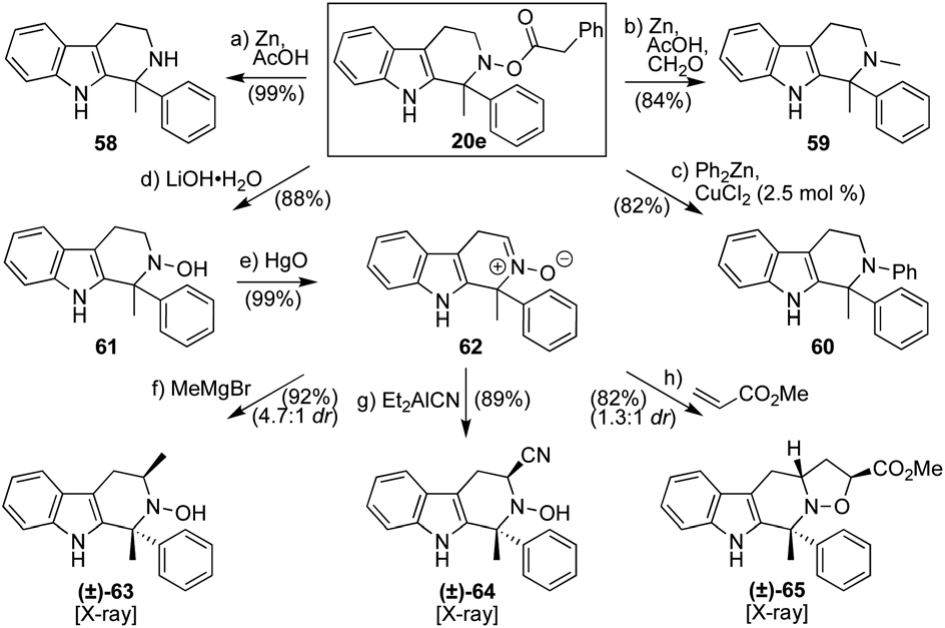
Selected transformations of Pictet–Spengler product **20e** to afford additional compounds of value: (a) Zn (15 equiv.), AcOH, 60 °C, 3 h, 99%; (b) Zn (15 equiv.), CH_2_O (6.0 equiv.), AcOH, 60 °C, 3 h, 84%; (c) Ph_2_Zn (1.1 equiv.), CuCl_2_ (2.5 mol%), THF, 23 °C, 2 h, 82%; (d) LiOH·H_2_O (1.1 equiv.), MeOH, 23 °C, 0.5 h, 88%; (e) HgO (3.0 equiv.), CH_2_Cl_2_ : MeOH (10 : 1), 23 °C, 0.5 h, sonication, 99%; (f) MeMgBr (10 equiv.), THF, −78 to 0 °C, 1 h, 92%, 4.7 : 1 dr; (g) Et_2_AlCN (3.0 equiv.), CH_2_Cl_2_, 0 to 23 °C, 2 h, 89%; (h) methyl acrylate (10 equiv.), CH_2_Cl_2_, 23 °C, 2 h, 82%, 1.3 : 1 dr at aza-quaternary center.

Finally, while the use of ketonitrones in this procedure does necessitate the expenditure of some supplemental synthetic operations to prepare the relevant starting materials, this investment can pay added synthetic dividends beyond substrate scope and the potential for enantiocontrol. As shown in Scheme 5, one can, of course, readily cleave the N–O bond within **20e** through standard treatment with Zn in AcOH at 60 °C to generate **58** in quantitative yield.^8,21^ More valuable, potentially, is the ability to affect that same reaction and perform a reductive amination in the same pot, shown here by the formation of **59**.^8,22^ Equally important, **20e** can engage directly in electrophilic amination reactions.^23,24^ Although *O*-acyl hydroxylamines are commonly used in this event, the phenylacetyl group has never been tested to the best of our knowledge, with the neighboring aza-quaternary center also providing a high degree of steric bulk that might thwart success. Nevertheless, applying Johnson's conditions,^23a^ we were able to access the desired tertiary amine **60***via* a copper-catalyzed electrophilic amination with a diorganozinc reagent (Ph_2_Zn).^24^ Lastly, following simple deprotection of the acyl group using LiOH·H_2_O to release free hydroxylamine **61**,^25^ aldonitrone **62** was prepared through the action of HgO.^8,26^ This species could then be engaged by nucleophiles (such as MeMgBr^27^ and Et_2_AlCN^28^) or in a [3 + 2]-cycloaddition reaction^29^ with methyl acrylate to afford **63–65**, respectively.^30^ All of these latter products were confirmed by X-ray crystallography as possessing the relative configurations shown.

## Conclusions

In conclusion, we have developed a mild, fast, and high-yielding method to generate a variety of tetrahydro-β-carbolines bearing aza-quaternary centers by effecting Pictet–Spengler reactions of ketonitrones, rendered competent here by converting them into *N*-acyloxyiminium ions. This approach allows for significant substrate scope with high functional group tolerance and good to excellent yields. Moreover, we have established a catalytic, asymmetric variant of the reaction that proceeds with good enantioselectivity, along with an initial understanding of the range for its efficacy. Overall, we believe this method can be utilized in diverse synthetic applications to access compounds of value, such as those highlighted in Scheme 1, with the range of functionalizations available to the final products supporting the validity of this idea. Such efforts are the subject of current endeavors.

## Conflicts of interest

There are no conflicts to declare.

## Author contributions

S. A. S. and T. L.-C. conceived the project. S. A. S. directed the research, and S. A. S. and T. L.-C. composed the manuscript and the and the ESI Section.† T. L.-C. developed the initial reactions and explored substrate scope, while S. G. completed the synthesis of several starting materials and contributed significantly to the examples in the substrate tables.

## Supplementary Material

SC-012-D1SC00882J-s001

SC-012-D1SC00882J-s002
