# Supplementary material for: Synthesis of aza-quaternary centers via Pictet–Spengler reactions of ketonitrones
Source: Chem Sci. 2021 Mar 16;12(17):6181–7. doi: 10.1039/d1sc00882j (PMC8098696; doi:10.1039/d1sc00882j)
Supplement: SC-012-D1SC00882J-s001 [file SC-012-D1SC00882J-s001.pdf]

Electronic Supplementary Material (ESI) for Chemical Science.
This journal is © The Royal Society of Chemistry 2021

Synthesis of *Aza*-Quaternary Centers via Pictet–Spengler Reactions of Ketonitrones

Tessa Lynch-Colameta, Sarah Greta, and Scott A. Snyder*

Department of Chemistry, The University of Chicago, 5735 S. Ellis Avenue, Chicago, IL 60637 (USA)

Supporting Information

Table of Contents

A.	General Procedures	S2
B.	Abbreviations	S2
C.	Preparations of Hydroxylamines	S3
D.	General Procedure for Preparation of Nitrones	S7
E.	General Procedure for Racemic Pictet–Spengler Reactions of Nitrones	S16
F.	Identification of Hydrolysis By-product	S26
G.	Catalyst Screening and Optimization	S27
H.	General Procedure for Enantioselective Pictet–Spengler Reactions of Nitrones	S29
I.	Derivatizations of Pictet–Spengler Products	S32
J.	Mechanistic Understanding	S37
K.	References	S38
L.	NMR Spectra	S39
M.	HPLC Traces	S135
N.	X-Ray Crystallography Data	S145

Experimental Data for Compounds

A. General Procedures. All reactions were carried out under an argon atmosphere with dry solvents under anhydrous conditions, unless otherwise noted. Dry tetrahydrofuran (THF), toluene, acetonitrile (MeCN), and dichloromethane (CH_2Cl_2) were obtained by passing commercially available pre-dried, oxygen-free formulations through activated alumina columns. Yields refer to chromatographically and spectroscopically (^{1}H and ^{13}C NMR) homogeneous materials, unless otherwise stated. Reagents were purchased at the highest commercial quality and used without further purification, unless otherwise stated. Reactions were magnetically stirred and monitored by thin-layer chromatography (TLC) carried out on 0.25 mm E. Merck silica gel plates (60F-254) using UV light as visualizing agent, and an aqueous solution of ceric ammonium sulfate, ammonium molybdate, and sulfuric acid or aqueous solution of potassium permanganate and sodium bicarbonate and heat as a developing agent. SiliCycle silica gel (60, academic grade, particle size 0.040–0.063 mm) was used for flash column chromatography. Preparative thin-layer chromatography separations were carried out on 0.50 mm E. Merck silica gel plates (60F-254). NMR spectra were recorded on Bruker 400 and 500 MHz instruments and calibrated using residual undeuterated solvent as an internal reference. The following abbreviations were used to explain the multiplicities: s = singlet, d = doublet, t = triplet, q = quartet, br = broad, m = multiplet. IR spectra were recorded on a Nicolet iS5 FT-IR spectrometer using neat thin film technique. High-resolution mass spectra (HRMS) were recorded on Agilent 6244 TOF-MS using ESI (Electrospray Ionization) or CI (Chemical Ionization) at the University of Chicago Mass Spectroscopy Core Facility. Chiral high-performance liquid chromatography (HPLC) analysis was performed using a Shimadzu Prominence analytical chromatograph with commercial ChiralPak columns (OD-H). The X-ray diffraction data were measured on a Bruker D8 VENTURE diffractometer at the University of Chicago X-ray Laboratory.

B. Abbreviations. EtOAc = ethyl acetate, AcOH = acetic acid, MeOH = methanol, DIAD = diisopropyl azodicarboxylate, TMSCl = trimethylsilyl chloride, IBX = 2-iodoxybenzoic acid, 4Å MS = 4Å molecular sieves, DMSO = dimethyl sulfoxide, BzCl = benzoyl chloride, AcCl = acetyl chloride, PivCl = pivaloyl chloride, MTBE = methyl *tert*-butyl ether, Nap = naphthalene, 4-DMAP = 4-dimethylaminopyridine, TMPDA = *N*,*N*,*N′*,*N′*-tetramethyl-1,3-propanediamine.

C. Preparation of Hydroxylamines.

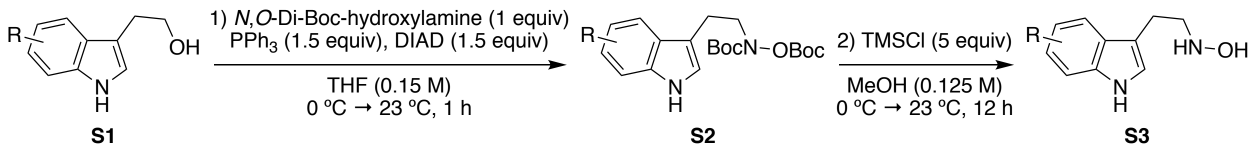

Method A.[1,2] *Step 1:* To a solution of tryptophol **S1** (1.0 equiv), Ph_3P (1.5 equiv), *N*,*O*-Di-Boc-hydroxylamine (1.0 equiv) in THF (0.15 M) at 0 ºC was added DIAD (1.5 equiv) dropwise under an argon atmosphere. The resultant mixture was warmed to 23 ºC and stirred for 1 h. Upon completion, MeOH (10.0 equiv) was added and the reaction was stirred for 15 min. The reaction contents were then concentrated directly and the resultant crude material was purified by flash column chromatography (silica gel, hexanes/EtOAc = 1/0→3/1) to yield **S2**. *Step 2:* Next, to a solution of the newly-formed **S2** (1.0 equiv) in MeOH (0.125 M) at 0 ºC was added TMSCl (5.0 equiv) dropwise under an argon atmosphere. The resultant mixture was slowly warmed to 23 ºC and stirred for 12 h. Upon completion, the reaction contents were concentrated directly and then diluted with CH_2Cl_2:MeOH (9:1). Saturated aqueous Na_2CO_3 was added, the contents were poured into a separatory funnel, and then the product was extracted with CH_2Cl_2 (3 ×). The combined organic extracts were then dried (Na_2SO_4), filtered, and concentrated. The resultant crude material was purified either by flash column chromatography (silica gel, CH_2Cl_2/MeOH = 1/0→9/1) or recrystallization (CH_2Cl_2) to afford **S3**.

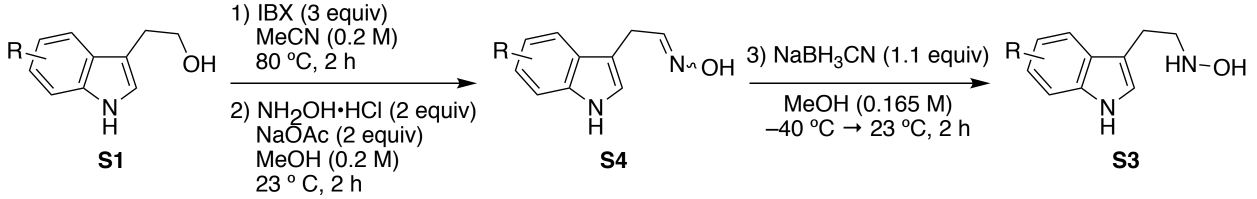

Method B.[3,4] *Step 1:* To a solution of tryptophol **S1** (1.0 equiv) in MeCN (0.2 M) at 23 ºC was added IBX (3.0 equiv) under an argon atmosphere. The resultant mixture was then heated at 80 ºC with stirring for 2 h. Upon completion, the reaction contents were cooled to 23 ºC, filtered through a pad of Celite, and rinsed with MeCN. The reaction contents were then concentrated directly to yield an aldehyde (not shown). This material was carried forward without any further purification. *Step 2:* Next, to a solution of the newly-prepared aldehyde (1.0 equiv) and NaOAc (2.0 equiv) in MeOH (0.2 M) at 23 º C was added NH_2OH•HCl (2.0 equiv) under an argon atmosphere. The resultant mixture was stirred for 2 h at 23 ºC. Upon completion, the reaction contents were concentrated directly and then diluted with EtOAc. Saturated aqueous $NaHCO_3$ was added, the contents were poured into a separatory funnel, and then the product was extracted with EtOAc (3 ×). The combined organic extracts were then dried (Na_2SO_4), filtered, and concentrated. The resultant crude material was purified by flash column chromatography (silica gel, CH_2Cl_2/MeOH = 1/0→9/1) to yield **S4**. *Step 3:* Finally, to a solution of **S4** (1.0 equiv) and methyl orange (~5 mg) in MeOH (0.2 M) at –40 ºC was concurrently added a solution of $NaBH_3CN$ (1.1 equiv) in MeOH (1.0 M) and 6 M aqueous HCl/MeOH (1/1) dropwise open to air. The resultant mixture was slowly warmed to 23 ºC over 2 h, adding HCl as needed to maintain the color of the reaction solution red (pH < 3.1). Upon completion, the reaction contents were concentrated directly and then diluted with Et_2O. Saturated aqueous NaCl was added and

the solution was basified with 6 M aqueous KOH. The contents were then poured into a separatory funnel and then the product was extracted with EtOAc (3 ×). The combined organic extracts were then dried (Na_2SO_4), filtered, and concentrated. The resultant crude material was purified by flash column chromatography (silica gel, CH_2Cl_2/MeOH = 1/0→9/1) to yield **S3**.

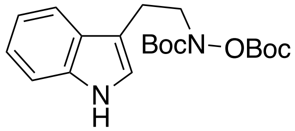

S5

***tert*-butyl (2-(1*H*-indol-3-yl)ethyl)((*tert*-butoxycarbonyl)oxy)carbamate (S5).** Prepared using Method A, Step 1 described above, starting from tryptophol **17** (2.00 g, 12.41 mmol), ultimately yielding **S5** (2.81 g, 60% yield) as a pale yellow solid. **S5**: R_f = 0.44 (silica gel, hexanes/EtOAc = 4/1); IR (film) ν_{max} 3373, 2980, 1786, 1710, 1597, 1458, 1395, 1370, 1244, 1145, 1099, 743 cm^{-1}; ^{1}H NMR (500 MHz, $CDCl_3$) δ 8.06 (br s, 1 H, exchangeable), 7.62 (d, *J* = 7.8 Hz, 1 H), 7.36 (d, *J* = 8.1 Hz, 1 H), 7.22–7.15 (m, 1 H), 7.15–7.09 (m, 1 H), 7.06 (d, *J* = 2.1 Hz, 1 H), 3.91 (m, 2 H), 3.09 (t, *J* = 7.6 Hz, 2 H), 1.54 (s, 9 H), 1.38 (s, 9 H); ^{13}C NMR (101 MHz, $CDCl_3$) δ 154.9, 152.5, 136.4, 127.5, 122.5, 122.0, 119.3, 118.7, 112.3, 111.3, 85.0, 82.4, 50.9, 28.1, 27.7, 23.1; HRMS (ESI) calcd for $C_{20}H_{29}N_2O_5^+$ $[M + H]^+$ 377.2071, found 377.2071.

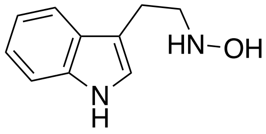

18

***N*-(2-(1*H*-indol-3-yl)ethyl)hydroxylamine (18).** Prepared using Method A, Step 2 described above with **S5** (2.50 g, 6.64 mmol), ultimately yielding **18** (0.950 g, 81% yield) as a white solid. **18**: R_f = 0.36 (silica gel, CH_2Cl_2/MeOH = 9/1); IR (film) ν_{max} 3395, 2926, 1597, 1564, 1455, 1228, 1093, 1024, 857, 808, 742 cm^{-1}; ^{1}H NMR (500 MHz, DMSO-d_6) δ 10.77 (br s, 1 H, exchangeable), 7.50 (d, *J* = 7.8 Hz, 1 H), 7.32 (d, *J* = 8.1 Hz, 1 H), 7.23 (br s, 1 H, exchangeable), 7.13 (d, *J* = 2.3 Hz, 1 H), 7.08–7.02 (m, 1 H), 7.00–6.92 (m, 1 H), 5.59 (br s, 1 H, exchangeable), 3.01 (t, *J* = 7.5 Hz, 2 H), 2.84 (t, *J* = 7.4 Hz, 2 H); ^{13}C NMR (101 MHz, DMSO-d_6) δ 136.3, 127.4, 122.6, 120.9, 118.3, 118.2, 112.5, 111.4, 54.5, 23.0; HRMS (CI) calcd for $C_{10}H_{13}N_2O^+$ $[M + H]^+$ 177.1022, found 177.1026.

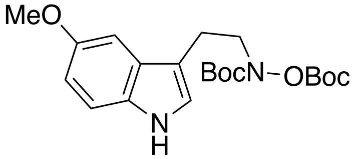

S6

***tert*-butyl ((*tert*-butoxycarbonyl)oxy)(2-(5-methoxy-1*H*-indol-3-yl)ethyl)carbamate (S6).** Prepared using Method A, Step 1 described above, starting from 5-methoxytryptophol (0.900 g, 4.71 mmol), ultimately yielding **S6** (0.950 g, 50% yield) as a yellow solid. **S6**: R_f = 0.30 (silica gel, hexanes/EtOAc = 4/1); IR (film) ν_{max} 3373, 1785, 1597, 1564, 1482, 1448, 1394, 1219, 1145, 1097 cm^{-1}; ^{1}H NMR (400 MHz, $CDCl_3$) δ 8.09 (br s, 1 H, exchangeable), 7.24 (d, *J* = 8.8 Hz, 1 H), 7.06 (d, *J* = 2.5 Hz, 1 H), 7.01 (d, *J* = 2.5 Hz, 1 H), 6.85 (dd, *J* = 8.8, 2.5 Hz, 1 H), 3.96–3.88 (m, 2 H), 3.87 (s, 3 H), 3.06 (t, *J* = 7.1 Hz, 2 H), 1.54 (s, 9 H), 1.37 (s, 9 H); ^{13}C NMR (101 MHz, $CDCl_3$) δ 154.9, 154.0, 152.5, 131.5, 127.9, 123.2, 112.3, 112.2, 112.0, 100.6, 84.9, 82.3, 56.0, 50.8, 28.1, 27.8, 23.1; HRMS (ESI) calcd for $C_{21}H_{31}N_2O_6^+$ $[M + H]^+$ 407.2177, found 407.2175.

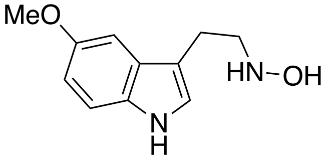

S7

***N*-(2-(5-methoxy-1*H*-indol-3-yl)ethyl)hydroxylamine (S7).** Prepared using Method A, Step 2 described above with **S6** (0.870 g, 2.14 mmol), ultimately yielding **S7** (0.355 g, 80% yield) as a white solid. **S7**: R_f = 0.35 (silica gel, CH_2Cl_2/MeOH = 9/1); IR (film) ν_{max} 3409, 2936, 1597, 1485, 1450, 1295, 1217, 1173, 1069, 1029, 798 cm^{-1}; ^{1}H NMR (400 MHz, DMSO-d_6) δ 10.62 (br s, 1 H

exchangeable), 7.30–7.18 (m, 2 H), 7.09 (d, *J* = 2.4 Hz, 1 H), 6.99 (d, *J* = 2.4 Hz, 1 H), 6.71 (dd, *J* = 8.7, 2.4 Hz, 1 H), 5.60 (br s, 1 H, exchangeable), 3.76 (s, 3 H), 3.02 (t, *J* = 7.4 Hz, 2 H), 2.82 (t, *J* = 7.4 Hz, 2 H); ^{13}C NMR (101 MHz, DMSO-d_6) δ 152.9, 131.4, 127.6, 123.3, 112.2, 112.0, 111.0, 100.1, 55.3, 54.4, 22.9; HRMS (CI) calcd for $C_{11}H_{15}N_2O_2^+$ [M + H]$^+$ 207.1128, found 207.1130.

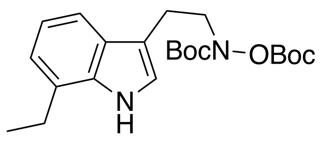

S8

***tert*-butyl ((*tert*-butoxycarbonyl)oxy)(2-(7-ethyl-1*H*-indol-3-yl)ethyl)carbamate (S8).** Prepared using Method A, Step 1 described above, starting from 7-ethyltryptophol (1.00 g, 5.28 mmol), ultimately yielding **S8** (1.30 g, 61% yield) as a yellow solid. **S8**: R_f = 0.50 (silica gel, hexanes/EtOAc = 4/1); IR (film) ν_{max} 3373, 2978, 1786, 1712, 1597, 1564, 1448, 1395, 1370, 1246, 1151, 1131 cm^{-1}; ^{1}H NMR (400 MHz, $CDCl_3$) δ 8.14 (br s, 1 H, exchangeable), 7.49 (d, *J* = 7.4 Hz, 1 H), 7.12–7.01 (m, 3 H), 4.00–3.84 (m, 2 H), 3.10 (t, *J* = 7.7 Hz, 2 H), 2.85 (q, *J* = 7.6 Hz, 2 H), 1.55 (s, 9 H), 1.38 (s, 9 H), 1.34 (t, *J* = 7.6 Hz, 3 H); ^{13}C NMR (101 MHz, $CDCl_3$) δ 154.9, 152.5, 135.2, 127.2, 126.7, 122.1, 120.6, 119.7, 116.5, 112.9, 84.9, 82.3, 50.9, 28.1, 27.7, 24.1, 23.2, 14.0; HRMS (CI) calcd for $C_{22}H_{33}N_2O_5^+$ [M + H]$^+$ 405.2384, found 405.2376.

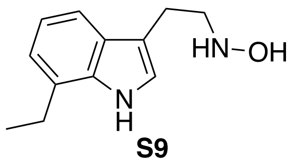

S9

***N*-(2-(7-ethyl-1*H*-indol-3-yl)ethyl)hydroxylamine (S9).** Prepared using Method A, Step 2 described above with **S8** (1.20 g, 2.97 mmol), ultimately yielding **S9** (0.400 g, 66% yield) as a white solid. **S9**: R_f = 0.45 (silica gel, CH_2Cl_2/MeOH = 9/1); IR (film) ν_{max} 3402, 2925, 1597, 1566, 1436, 1411, 1222, 1095, 794, 742 cm^{-1}; ^{1}H NMR (400 MHz, DMSO-d_6) δ 10.75 (br s, 1 H, exchangeable), 7.35 (dd, *J* = 7.4, 1.6 Hz, 1 H), 7.27 (br s, 1 H, exchangeable), 7.12 (d, *J* = 2.5 Hz, 1 H), 6.95–6.86 (m, 2 H), 5.60 (br s, 1 H, exchangeable), 3.08–2.98 (m, 2 H), 2.89–2.78 (m, 4 H), 1.26 (t, *J* = 7.5 Hz, 3 H); ^{13}C NMR (101 MHz, DMSO-d_6) δ 135.0, 127.2, 126.8, 122.2, 119.6, 118.5, 116.0, 112.8, 54.5, 23.7, 23.1, 14.5; HRMS (CI) calcd for $C_{12}H_{17}N_2O^+$ [M + H]$^+$ 205.1335, found 205.1337.

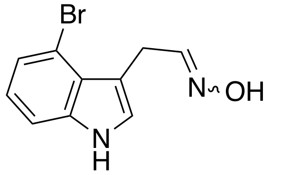

S10

2-(4-bromo-1*H*-indol-3-yl)acetaldehyde oxime (S10). Prepared using Method B, Steps 1 and 2 described above, starting from 4-bromotryptophol (2.10 g, 8.75 mmol), ultimately yielding **S10** (1.26 g, 57% yield over 2 steps, single undetermined stereoisomer) as a pale yellow solid. **S10**: R_f = 0.38 (silica gel, CH_2Cl_2/MeOH = 9/1); IR (film) ν_{max} 3419, 2924, 1620, 1424, 1334, 1186, 1043, 912, 773, 736 cm^{-1}; ^{1}H NMR (500 MHz, DMSO-d_6) δ 11.29 (br s, 1 H, exchangeable), 10.93 (br s, 1 H, exchangeable), 7.38 (d, *J* = 8.0 Hz, 1 H), 7.33 (d, *J* = 2.5 Hz, 1 H), 7.17 (d, *J* = 7.5 Hz, 1 H), 6.98 (t, *J* = 7.8 Hz, 1 H), 6.87 (t, *J* = 4.8 Hz, 1 H), 3.91 (d, *J* = 4.8 Hz, 2 H); ^{13}C NMR (101 MHz, DMSO-d_6) δ 150.0, 137.8, 125.7, 124.8, 122.7, 122.3, 112.8, 111.4, 110.3, 22.5; HRMS (ESI) calcd for $C_{10}H_{10}BrN_2O^+$ [M + H]$^+$ 252.9971, found 252.9971.

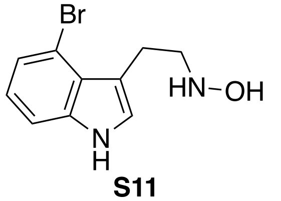

S11

***N*-(2-(4-bromo-1*H*-indol-3-yl)ethyl)hydroxylamine (S11).** Prepared using Method A, Step 3 described above with **S10** (0.360 g, 1.42 mmol), ultimately yielding **S11** (0.314 g, 87% yield) as a white solid. **S11**: R_f = 0.29 (silica gel, CH_2Cl_2/MeOH = 9/1); IR (film) ν_{max} 3272, 1477, 1333, 1183, 1119, 1032, 913, 856, 769, 736 cm^{-1}; ^{1}H NMR (500 MHz, DMSO-

d_6) δ 11.18 (br s, 1 H, exchangeable), 7.36 (d, J = 8.1 Hz, 1 H), 7.25 (d, J = 2.4 Hz, 1 H), 7.15 (d, J = 7.6 Hz, 1 H), 6.95 (t, J = 7.8 Hz, 1 H), 5.75 (br s, 1 H, exchangeable), 3.13–3.01 (m, 4 H); ^{13}C NMR (101 MHz, DMSO-d_6) δ 137.8, 125.2, 124.9, 122.5, 121.9, 113.0, 112.9, 111.2, 55.4, 23.8; HRMS (ESI) calcd for $C_{10}H_{12}BrN_2O^+$ $[M + H]^+$ 255.0128, found 255.0124.

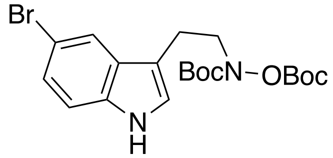

S12

***tert*-butyl (2-(5-bromo-1*H*-indol-3-yl)ethyl)((*tert*-butoxycarbonyl)oxy)carbamate (S12).** Prepared using Method A, Step 1 described above, starting from 5-bromotryptophol (1.10 g, 4.58 mmol), ultimately yielding **S12** (0.506 g, 24% yield) as a pale yellow solid. **S12**: R_f = 0.53 (silica gel, hexanes/EtOAc = 2/1); IR (film) ν_{max} 3354, 2981, 1785, 1715, 1597, 1456, 1370, 1242, 1147, 1102 cm^{-1}; ^1H NMR (500 MHz, $CDCl_3$) δ 8.20 (br s, 1 H, exchangeable), 7.72 (d, J = 1.8 Hz, 1 H), 7.27–7.19 (m, 2 H), 7.05 (d, J = 2.4 Hz, 1 H), 3.94–3.82 (m, 2 H), 3.03 (t, J = 7.3 Hz, 2 H), 1.54 (s, 9 H), 1.36 (s, 9 H); ^{13}C NMR (101 MHz, $CDCl_3$) δ 154.9, 152.4, 135.0, 129.3, 124.9, 123.8, 121.4, 112.8 (2 C), 112.3, 85.1, 82.5, 50.7, 28.1, 27.8, 23.0; HRMS (ESI) calcd for $C_{20}H_{28}BrN_2O_5^+$ $[M + H]^+$ 455.1176, found 455.1177.

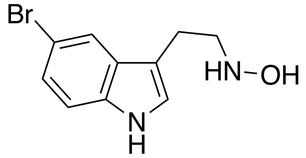

S13

***N*-(2-(5-bromo-1*H*-indol-3-yl)ethyl)hydroxylamine (S13).** Prepared using Method A, Step 2 described above with **S12** (0.410 g, 0.89 mmol), ultimately yielding **S13** (0.172 g, 75% yield) as a white solid. **S13**: R_f = 0.42 (silica gel, CH_2Cl_2/MeOH = 9/1); IR (film) ν_{max} 3425, 2921, 1597, 1564, 1449, 1394, 1220, 1096, 1049, 859, 797, 735 cm^{-1}; ^1H NMR (500 MHz, DMSO-d_6) δ 11.01 (br s, 1 H, exchangeable), 7.67 (d, J = 2.0 Hz, 1 H), 7.30 (d, J = 8.6 Hz, 1 H), 7.24 (br s, 1 H, exchangeable), 7.20 (d, J = 2.4 Hz, 1 H), 7.16 (dd, J = 8.6, 1.9 Hz, 1 H), 5.63 (br s, 1 H, exchangeable), 2.97 (t, J = 7.4 Hz, 2 H), 2.81 (t, J = 7.4 Hz, 2 H); ^{13}C NMR (101 MHz, DMSO-d_6) δ 134.9, 129.2, 124.4, 123.2, 120.6, 113.3, 112.4, 110.8, 54.4, 22.6; HRMS (ESI) calcd for $C_{10}H_{12}BrN_2O^+$ $[M + H]^+$ 255.0128, found 255.0132.

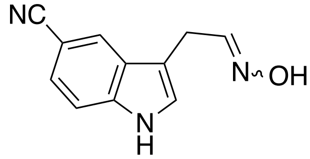

S14

(*E/Z*)-3-(2-(hydroxyimino)ethyl)-1*H*-indole-5-carbonitrile (S14). Prepared using Method B, Steps 1 and 2 described above, starting from 5-carbonitriletryptophol[5] (0.600 g, 3.22 mmol), ultimately yielding **S14** (0.415 g, 64% yield over 2 steps, 1:1 *E:Z*) as a yellow solid. **S14**: R_f = 0.42 (silica gel, CH_2Cl_2/MeOH = 9/1); IR (film) ν_{max} 3363, 2227, 1597, 1564, 1485, 1395, 1219 1100, 858, 799 cm^{-1}; ^1H NMR (500 MHz, DMSO-d_6) δ 11.51 (br s, 2 H, exchangeable), 11.09 (br s, 1 H, exchangeable), 10.53 (br s, 1 H, exchangeable), 8.08–8.01 (m, 2 H), 7.56–7.49 (m, 2 H), 7.49–7.38 (m, 5 H), 6.85 (t, J = 5.4 Hz, 1 H), 3.72 (d, J = 6.3 Hz, 2 H), 3.59 (d, J = 5.5 Hz, 2 H); ^{13}C NMR (101 MHz, DMSO-d_6) δ 148.5, 148.1, 138.1, 138.0, 126.9, 126.9, 126.0, 125.9, 124.3 124.2, 123.9 (2 C), 120.9 (2 C), 112.8 (2 C), 111.0, 111.0, 100.5, 100.5, 25.3, 20.8; HRMS (ESI) calcd for $C_{11}H_{10}N_3O^+$ $[M + H]^+$ 200.0818, found 200.0818.

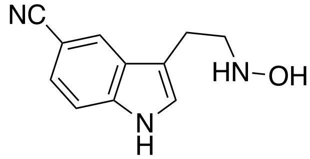

S15

3-(2-(hydroxyamino)ethyl)-1*H*-indole-5-carbonitrile (S15). Prepared using Method A, Step 3 described above with **S14** (0.150 g, 0.74 mmol), ultimately yielding **S15** (0.140 g, 94% yield) as a white solid. **S15**: R_f = 0.27 (silica gel, CH_2Cl_2/MeOH = 9/1); IR (film) ν_{max}

3362, 2924, 2370, 1597, 1563, 1448, 1394, 1219, 1091, 861 cm^{-1}; ^{1}H NMR (500 MHz, DMSO-d_6) δ 11.40 (br s, 1 H, exchangeable), 8.21–7.92 (m, 1 H), 7.49 (d, J = 8.4 Hz, 1 H), 7.40 (dd, J = 8.4, 1.6 Hz, 1 H), 7.36 (d, J = 2.3 Hz, 1 H), 7.25 (br s, 1 H, exchangeable), 5.65 (br s, 1 H, exchangeable), 3.00 (t, J = 7.2 Hz, 2 H), 2.86 (t, J = 7.2 Hz, 2 H); ^{13}C NMR (101 MHz, DMSO-d_6) δ 137.9, 127.3, 125.4, 124.3, 123.5, 121.0, 114.1, 112.6, 100.2, 54.3, 22.5; HRMS (ESI) calcd for $C_{11}H_{12}N_3O^+$ $[M + H]^+$ 202.0975, found 202.0975.

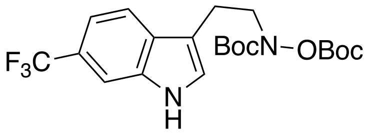

S16

***tert*-butyl ((*tert*-butoxycarbonyl)oxy)(2-(6-(trifluoromethyl)-1*H*-indol-3-yl)ethyl)carbamate (S16).** Prepared using Method A, Step 1 described above, starting from 6-trifluoromethyltryptophol[6] (1.00 g, 4.36 mmol), ultimately yielding **S16** (0.556 g, 29% yield) as a pale yellow solid. **S16**: R_f = 0.44 (silica gel, hexanes/EtOAc = 4/1); IR (film) ν_{max} 3357, 2982, 1786, 1709, 1597, 1459, 1395, 1337, 1242, 1149, 1107, 1070 cm^{-1}; ^{1}H NMR (500 MHz, $CDCl_3$) δ 8.37 (br s, 1 H, exchangeable), 7.69 (dd, J = 8.4, 0.8 Hz, 1 H), 7.65 (s, 1 H), 7.35 (dd, J = 8.6, 1.6 Hz, 1 H), 7.20 (d, J = 2.4 Hz, 1 H), 3.99–3.83 (m, 2 H), 3.09 (t, J = 7.3 Hz, 2 H), 1.53 (s, 9 H), 1.34 (s, 9 H); ^{13}C NMR (101 MHz, $CDCl_3$) δ 154.9, 152.4, 135.2, 129.8, 125.4 (q, J = 271.6 Hz) 125.3, 124.2 (q, J = 31.6 Hz), 119.2, 116.1, 112.9, 108.9, 85.2, 82.5, 50.7, 28.1, 27.7, 23.0; HRMS (ESI) calcd for $C_{21}H_{27}F_3N_2NaO_5^+$ $[M + Na]^+$ 467.1764, found 467.1769.

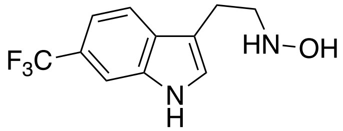

S17

***N*-(2-(6-(trifluoromethyl)-1*H*-indol-3-yl)ethyl)hydroxylamine (S17).** To a solution of **S16** (0.500 g, 1.13 mmol, 1 equiv) in CH_2Cl_2 (2.25 mL) at 23 ºC was added TFA (4.5 mL) and stirred for 2 h under an argon atmosphere. Then followed Method A, Step 2 work-up and purification procedure described above, ultimately yielding **S17** (0.212 g, 77% yield) as a white solid. **S17**: R_f = 0.41 (silica gel, CH_2Cl_2/MeOH = 9/1); IR (film) ν_{max} 3269, 1597, 1563, 1457, 1337, 1243, 1219, 1162, 1109, 1052, 815 cm^{-1}; ^{1}H NMR (400 MHz, DMSO-d_6) δ 11.27 (br s, 1 H, exchangeable), 7.74–7.66 (m, 2 H), 7.41 (d, J = 2.4 Hz, 1 H), 7.31–7.22 (m, 2 H), 5.64 (br s, 1 H, exchangeable), 3.02 (t, J = 7.4 Hz, 2 H), 2.89 (t, J = 7.3 Hz, 2 H); ^{13}C NMR (101 MHz, DMSO-d_6) δ 135.0, 129.8, 126.3, 125.6 (q, J = 271.2 Hz), 121.3 (q, J = 31.0 Hz), 119.2, 114.4, 113.2, 108.7, 54.3, 22.7; HRMS (ESI) calcd for $C_{11}H_{12}F_3N_2O^+$ $[M + H]^+$ 245.0896, found 245.0899.

D. General Procedure for Preparation of Nitrones.[7]

To a solution of hydroxylamine **18** (0.100 g, 0.57 mmol, 1.0 equiv) in CH_2Cl_2/MeOH (1/1, 3.8 mL) or MeOH (3.8 mL) at 23 ºC was added the requisite ketone (0.85–2.84 mmol, 1.5–5.0 equiv), AcOH (10 drops, from a syringe fitted with 3” needle), and $MgSO_4$ (0.205 g, 1.70 mmol, 3.0 equiv) under an argon atmosphere. The resultant mixture was stirred for 12 h either at 23 ºC or 50 ºC. Upon completion, the contents were quenched at 23 ºC with saturated aqueous $NaHCO_3$ (5 mL), poured into a separatory funnel, and extracted with CH_2Cl_2 (3 × 5 mL). The combined organic extracts were then dried (Na_2SO_4), filtered, and concentrated. The resultant crude material was purified by flash column chromatography (silica gel, CH_2Cl_2/MeOH = 1/0→9/1) to yield nitrone **19**, **S18**–**S45**. *Note:* The ratio of mixed *E-/Z-* isomers can vary depending on conditions (time, temperature, and equivalents). In addition, the *E-/Z-* ratio for each nitrone was determined by NOESY experiments.

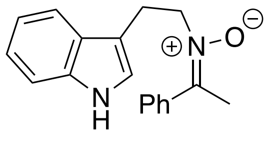
19

(*E*)-*N*-(2-(1*H*-indol-3-yl)ethyl)-1-phenylethan-1-imine oxide (19). Prepared using the general procedure described above with **18** and acetophenone (5.0 equiv) in CH_2Cl_2/MeOH (1/1) at 23 ºC, ultimately yielding **19** (0.152 g, 96% yield) as a white solid. When executed on gram scale with **18** (1.41 g, 8.00 mmol, 1.0 equiv), acetophenone (3.0 equiv), and AcOH (1 equiv) in CH_2Cl_2/MeOH (1/1) at 23 ºC, ultimately yielding **19** (2.13 g, 96% yield) as a white solid. **19**: R_f = 0.62 (silica gel, CH_2Cl_2/MeOH = 9/1); IR (film) ν_{max} 3213, 2922, 1597, 1564, 1446, 1220, 1165, 1071, 763, 744, 700 cm^{-1}; ^1H NMR (500 MHz, $CDCl_3$) δ 8.23 (br s, 1 H, exchangeable), 7.36 (d, *J* = 8.2 Hz, 1 H), 7.29–7.22 (m, 2 H), 7.21–7.13 (m, 3 H), 7.02–6.95 (m, 2 H), 6.74 (m, 2 H), 4.07 (t, *J* = 7.0 Hz, 2 H), 3.37 (t, *J* = 7.1 Hz, 2 H), 2.36 (s, 3 H); 13C NMR (101 MHz, $CDCl_3$) δ 148.5, 136.3, 136.1, 128.9, 128.7, 127.4, 127.2, 122.7, 122.0, 119.3, 118.5, 111.7, 111.3, 60.3, 23.9, 20.8; HRMS (ESI) calcd for $C_{18}H_{19}N_2O^+$ $[M + H]^+$ 279.1492, found 279.1497.

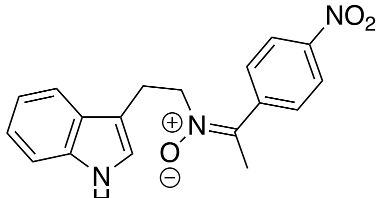
S18

(*E*)-*N*-(2-(1*H*-indol-3-yl)ethyl)-1-(4-nitrophenyl)ethan-1-imine oxide (S18). Prepared using the general procedure described above with **18** and 4′-nitroacetophenone (2.5 equiv) in CH_2Cl_2/MeOH (1/1) at 23 ºC, ultimately yielding **S18** (0.162 g, 88% yield) as a bright yellow solid. **S18**: R_f = 0.43 (silica gel, CH_2Cl_2/MeOH = 9/1); IR (film) ν_{max} 3245, 1598, 1561, 1518, 1400, 1349, 1219, 1069, 857, 744 cm^{-1}; ^1H NMR (400 MHz, $CDCl_3$) δ 8.50 (br s, 1 H, exchangeable), 7.83–7.75 (m, 2 H), 7.42–7.36 (m, 1 H), 7.22–7.14 (m, 2 H), 6.99 (d, *J* = 2.3 Hz, 1 H), 6.95–6.88 (m, 1 H), 6.60–6.53 (m, 2 H), 4.10 (t, *J* = 6.0 Hz, 2 H), 3.33 (t, *J* = 6.1 Hz, 2 H), 2.28 (s, 3 H); ^{13}C NMR (101 MHz, $CDCl_3$) δ 147.3, 146.2, 142.0, 136.3, 128.3, 127.4, 123.6, 122.9, 122.5, 119.7, 118.3, 111.6, 111.4, 60.8, 23.7, 20.5; HRMS (CI) calcd for $C_{18}H_{18}N_3O_3^+$ $[M + H]^+$ 324.1343, found 324.1342.

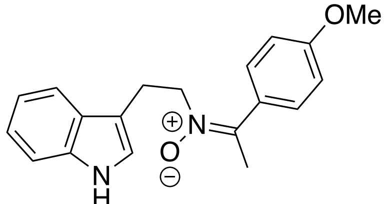
S19

(*E*)-*N*-(2-(1*H*-indol-3-yl)ethyl)-1-(4-methoxyphenyl)ethan-1-imine oxide (S19). Prepared using the general procedure described above with **18** and 4′-methoxyacetophenone (3.5 equiv) in MeOH at 50 ºC, ultimately yielding **S19** (0.155 g, 89% yield) as a gray solid. **S19**: R_f = 0.46 (silica gel, CH_2Cl_2/MeOH = 9/1); IR (film) ν_{max} 3181, 2922, 1607, 1513, 1457, 1289, 1250, 1165, 1071, 1028, 833, 743 cm^{-1}; ^1H NMR (500 MHz, $CDCl_3$) δ 8.19 (br s, 1 H, exchangeable), 7.39–7.33 (m, 1 H), 7.28 (d, *J* = 7.9 Hz, 1 H), 7.21–7.13 (m, 1 H), 7.04–6.97 (m, 2 H), 6.66 (d, *J* = 0.9 Hz, 4 H), 4.08 (t, *J* = 7.0 Hz, 2 H), 3.76 (s, 3 H), 3.36 (t, *J* = 7.0 Hz, 2 H), 2.34 (s, 3 H); ^{13}C NMR (101 MHz, $CDCl_3$) δ 159.8, 148.6, 136.4, 128.6, 128.3, 127.5, 122.8, 121.9, 119.3, 118.6, 114.0, 111.7, 111.3, 60.1, 55.4, 23.9, 20.9; HRMS (ESI) calcd for $C_{19}H_{21}N_2O_2^+$ $[M + H]^+$ 309.1598, found 309.1604.

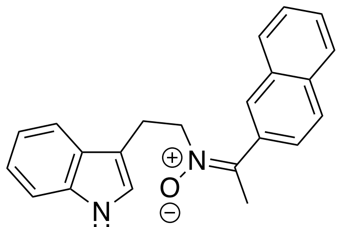
S20

(*E*)-*N*-(2-(1*H*-indol-3-yl)ethyl)-1-(naphthalen-2-yl)ethan-1-imine oxide (S20). Prepared using the general procedure described above with **18** and 2-acetonaphthone (5.0 equiv) in CH_2Cl_2/MeOH (1/1) at 23 ºC, ultimately yielding **S20** (0.160 g, 86% yield) as a pale yellow solid. **S20**: R_f = 0.51 (silica gel, CH_2Cl_2/MeOH = 9/1); IR (film) ν_{max} 3210, 1597, 1563, 1448, 1389, 1219, 1164, 1065, 858, 744 cm^{-}

[1]; ^{1}H NMR (500 MHz, $CDCl_3$) δ 8.14 (br s, 1 H, exchangeable), 7.77 (d, *J* = 8.1 Hz, 1 H), 7.66 (d, *J* = 8.5 Hz, 1 H), 7.53–7.47 (m, 1 H), 7.47–7.42 (m, 1 H), 7.42–7.36 (m, 2 H), 7.24 (d, *J* = 7.9 Hz, 1 H), 7.18–7.12 (m, 1 H), 7.01–6.97 (m, 1 H), 6.97–6.94 (m, 1 H), 6.91–6.83 (m, 2 H), 4.13 (t, *J* = 6.7 Hz, 2 H), 3.41 (t, *J* = 6.8 Hz, 2 H), 2.43 (s, 3 H); ^{13}C NMR (101 MHz, $CDCl_3$) δ 148.7, 136.3, 133.3, 132.8, 132.7, 128.6, 128.3, 127.7, 127.5, 127.1, 127.0, 126.8, 124.4, 122.9, 122.1, 119.4, 118.6, 111.7, 111.3, 60.3, 23.7, 21.0; HRMS (CI) calcd for $C_{22}H_{21}N_2O^+$ $[M + H]^+$ 329.1648, found 329.1657.

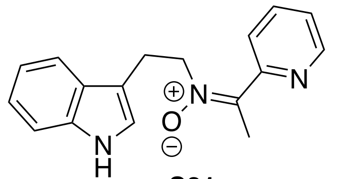
S21

(*E*)-*N*-(2-(1*H*-indol-3-yl)ethyl)-1-(pyridin-2-yl)ethan-1-imine oxide (S21). Prepared using the general procedure described above with **18** and 2-acetylpyridine (3.0 equiv) in CH_2Cl_2/MeOH (1/1) at 23 ºC, ultimately yielding **S21** (0.120 g, 76% yield) as a yellow solid. **S21**: R_f = 0.37 (silica gel, CH_2Cl_2/MeOH = 9/1); IR (film) ν_{max} 3220, 2922, 1586, 1561, 1430, 1264, 1223, 1169, 1102, 1085, 784, 743 cm^{-1}; ^{1}H NMR (500 MHz, $CDCl_3$) δ 8.60–8.44 (m, 1 H), 8.24 (br s, 1 H, exchangeable), 7.43–7.30 (m, 3 H), 7.17–7.11 (m, 1 H), 7.09 (ddd, *J* = 7.6, 4.8, 1.0 Hz, 1 H), 7.02–6.95 (m, 2 H), 6.67–6.61 (m, 1 H), 4.32 (t, *J* = 7.1 Hz, 2 H), 3.41 (t, *J* = 7.1 Hz, 2 H), 2.40 (s, 3 H); ^{13}C NMR (101 MHz, $CDCl_3$) δ 153.8, 149.2, 146.4, 136.5, 136.3, 127.4, 123.2, 123.1, 122.7, 122.0, 119.3, 118.5, 112.0, 111.3, 61.0, 24.1, 19.4; HRMS (ESI) calcd for $C_{17}H_{18}N_3O^+$ $[M + H]^+$ 280.1444, found 280.1451.

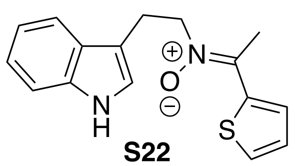
S22

(*Z*)-*N*-(2-(1*H*-indol-3-yl)ethyl)-1-(thiophen-2-yl)ethan-1-imine oxide (S22). Prepared using the general procedure described above with **18** and 2-acetylthiophene (5.0 equiv) in MeOH at 50 ºC, ultimately yielding **S22** (0.136 g, 84% yield) as a yellow solid. **S22**: R_f = 0.55 (silica gel, CH_2Cl_2/MeOH = 9/1); IR (film) ν_{max} 3180, 2921, 1597, 1563, 1456, 1419, 1365, 1337, 1212, 1160, 1101, 744 cm^{-1}; ^{1}H NMR (500 MHz, $CDCl_3$) δ 8.10 (br s, 1 H, exchangeable), 7.63 (d, *J* = 7.8 Hz, 1 H), 7.48 (d, *J* = 5.1 Hz, 1 H), 7.41–7.33 (m, 2 H), 7.23–7.17 (m, 1 H), 7.16–7.08 (m, 2 H), 6.99 (d, *J* = 2.2 Hz, 1 H), 4.36 (t, *J* = 7.0 Hz, 2 H), 3.48 (t, *J* = 7.0 Hz, 2 H), 2.15 (s, 3 H); ^{13}C NMR (101 MHz, $CDCl_3$) δ 137.6, 136.3, 134.6, 130.2, 129.1, 127.2, 126.0, 122.9, 122.2, 119.7, 118.4, 111.5 (2 C), 59.9, 23.8, 16.4; HRMS (ESI) calcd for $C_{16}H_{17}N_2OS^+$ $[M + H]^+$ 285.1056, found 285.1058.

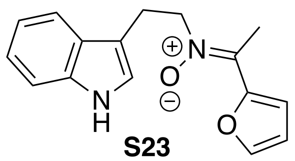
S23

(*Z*)-*N*-(2-(1*H*-indol-3-yl)ethyl)-1-(furan-2-yl)ethan-1-imine oxide (S23). Prepared using the general procedure described above with **18** and 2-acetylfuran (5.0 equiv) in MeOH at 50 ºC, ultimately yielding **S23** (0.126 g, 83% yield) as a yellow solid. **S23**: R_f = 0.47 (silica gel, CH_2Cl_2/MeOH = 9/1); IR (film) ν_{max} 3178, 1597, 1563, 1482, 1449, 1389, 1219, 1153, 1073, 744 cm^{-1}; ^{1}H NMR (500 MHz, $CDCl_3$) δ 8.16–8.05 (m, 2 H, 1 exchangeable), 7.64 (d, *J* = 7.7 Hz, 1 H), 7.50–7.45 (m, 1 H), 7.40–7.33 (m, 1 H), 7.24–7.17 (m, 1 H), 7.16–7.08 (m, 1 H), 7.04 (d, *J* = 2.3 Hz, 1 H), 6.58 (dd, *J* = 3.5, 1.7 Hz, 1 H), 4.27 (t, *J* = 7.2 Hz, 2 H), 3.45 (t, *J* = 7.2 Hz, 2 H), 2.12 (s, 3 H); ^{13}C NMR (101 MHz, $CDCl_3$) δ 148.0, 143.6, 136.4, 134.4, 127.2, 122.9, 122.1, 119.5, 118.3, 117.0, 112.7, 111.5, 111.4, 60.7, 23.7, 14.7; HRMS (CI) calcd for $C_{16}H_{17}N_2O_2^+$ $[M + H]^+$ 269.1285, found 269.1287.

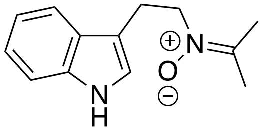

S24

***N*-(2-(1*H*-indol-3-yl)ethyl)propan-2-imine oxide (S24).** Prepared using the general procedure described above with **18** and acetone (50.0 equiv) at 23 ºC, ultimately yielding **S24** (0.112 g, 91% yield) as a white solid. **S24**: R_f = 0.38 (silica gel, CH_2Cl_2/MeOH = 9/1); IR (film) ν_{max} 3182, 2922, 1597, 1564, 1448, 1392, 1218, 1141, 1071, 858, 798, 744 cm^{-1}; ^1H NMR (500 MHz, $CDCl_3$) δ 8.72 (br s, 1 H, exchangeable), 7.61 (d, *J* = 7.9 Hz, 1 H), 7.38 (d, *J* = 8.1 Hz, 1 H), 7.22–7.16 (m, 1 H), 7.14–7.08 (m, 1 H), 7.04 (d, *J* = 2.4 Hz, 1 H), 4.13 (t, *J* = 6.9 Hz, 2 H), 3.39 (t, *J* = 6.9 Hz, 2 H), 2.08 (s, 3 H), 1.66 (s, 3 H); ^{13}C NMR (101 MHz, $CDCl_3$) δ 144.9, 136.3, 127.3, 122.7, 122.1, 119.5, 118.4, 111.8, 111.4, 59.4, 23.4, 20.3, 20.1; HRMS (ESI) calcd for $C_{13}H_{17}N_2O^+$ $[M + H]^+$ 217.1335, found 217.1336.

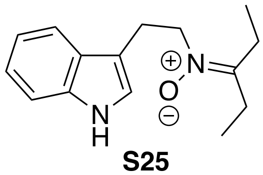

S25

***N*-(2-(1*H*-indol-3-yl)ethyl)pentan-3-imine oxide (S25).** Prepared using the general procedure described above with **18** and 3-pentanone (5.0 equiv) in CH_2Cl_2/MeOH (1/1) at 23 ºC, ultimately yielding **S25** (0.133 g, 96% yield) as a yellow solid. **S25**: R_f = 0.49 (silica gel, CH_2Cl_2/MeOH = 9/1); IR (film) ν_{max} 3173, 2974, 1597, 1458, 1342, 1234, 1141, 1108, 1072, 743 cm^{-1}; ^1H NMR (500 MHz, $CDCl_3$) δ 8.42 (br s, 1 H, exchangeable), 7.63 (d, *J* = 7.8 Hz, 1 H), 7.41–7.34 (m, 1 H), 7.23–7.16 (m, 1 H), 7.15–7.09 (m, 1 H), 7.06 (d, *J* = 2.2 Hz, 1 H), 4.11 (t, *J* = 7.0 Hz, 2 H), 3.41 (t, *J* = 7.0 Hz, 2 H), 2.54 (q, *J* = 7.5 Hz, 2 H), 2.02 (q, *J* = 7.6 Hz, 2 H), 1.07 (t, *J* = 7.5 Hz, 3 H), 0.81 (t, *J* = 7.6 Hz, 3 H); ^{13}C NMR (101 MHz, $CDCl_3$) δ 154.8, 136.5, 127.2, 123.2, 121.8, 119.2, 118.2, 111.6, 111.1, 59.0, 24.9, 24.2, 23.7, 11.0, 9.3; HRMS (CI) calcd for $C_{15}H_{21}N_2O^+$ $[M + H]^+$ 245.1648, found 245.1660.

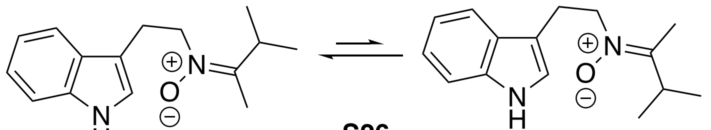

S26

(*E/Z*)-*N*-(2-(1*H*-indol-3-yl)ethyl)-3-methylbutan-2-imine oxide (S26). Prepared using the general procedure described above with **18** and methyl isopropyl ketone (5.0 equiv) in CH_2Cl_2/MeOH (1/1) at 23 ºC, ultimately yielding **S26** (0.130 g, 94% yield, 1.4:1 *E*:*Z*) as a yellow solid. **S26**: R_f = 0.43 (silica gel, CH_2Cl_2/MeOH = 9/1); IR (film) ν_{max} 3178, 1597, 1561, 1449, 1389, 1207, 1095,1074, 858, 744 cm^{-1}; ^1H NMR (500 MHz, $CDCl_3$, 1.4:1 *E*:*Z*) δ 8.53 (br s, 1 H, exchangeable), 7.63 (t, *J* = 7.6 Hz, 1 H), 7.40–7.32 (m, 1 H), 7.22–7.15 (m, 1 H), 7.15–7.09 (m, 1 H), 7.08–7.03 (m, 1 H), 4.19 (t, *J* = 6.8 Hz, 1.1 H), 4.11 (t, *J* = 6.9 Hz, 0.8 H), 3.84–3.74 (m, 0.3 H), 3.43–3.35 (m, 2 H), 2.60–2.48 (m, 0.6 H), 1.94 (s, 1.7 H), 1.48 (s, 1.2 H), 0.93 (d, *J* = 7.0 Hz, 2.5 H), 0.65 (d, *J* = 6.8 Hz, 3.5 H); ^{13}C NMR (101 MHz, $CDCl_3$, 1.4:1 *E*:*Z*) δ 154.5, 153.2, 136.6, 136.5, 127.3, 127.3, 123.2, 123.0, 122.0, 121.9, 119.4, 119.4, 118.3, 118.2, 111.6, 111.6, 111.4, 111.3, 59.8, 58.9, 31.4, 28.4, 23.9, 23.4, 19.3, 18.3, 13.4, 13.1; HRMS (ESI) calcd for $C_{15}H_{21}N_2O^+$ $[M + H]^+$ 245.1648, found 245.1653.

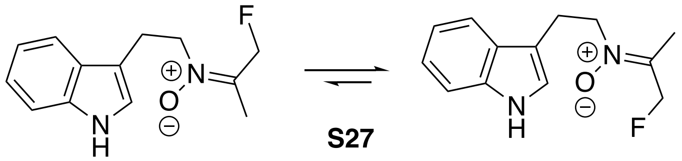

S27

(*E/Z*)-*N*-(2-(1*H*-indol-3-yl)ethyl)-1-fluoropropan-2-imine oxide (S27). Prepared using the general procedure described above with **18** and fluoroacetone (5.0 equiv) in CH_2Cl_2/MeOH (1/1) at 23 ºC, ultimately yielding **S27** (0.060 g, 45% yield, 1:3.6 *E*:*Z*) as a white solid. **S27**: R_f = 0.41 (silica gel, CH_2Cl_2/MeOH = 9/1); IR (film) ν_{max} 3220, 1597, 1562, 1481,

1448, 1389, 1219, 1157, 1030, 708, 745 cm^{-1}; ^{1}H NMR (500 MHz, $CDCl_3$, 1:3.6 *E*:*Z*) δ 8.11 (br s, 1 H, exchangeable), 7.65–7.57 (m, 1 H), 7.41–7.34 (m, 1 H), 7.24–7.19 (m, 1 H), 7.17–7.11 (m, 1 H), 7.09–7.03 (m, 1 H), 5.26 (d, *J* = 48.5 Hz, 1.5 H), 4.44 (d, *J* = 47.2 Hz, 0.4 H), 4.22 (t, *J* = 7.3 Hz, 0.4 H), 4.11 (t, *J* = 6.7 Hz, 1.6 H), 3.43–3.35 (m, 2 H), 2.09 (d, *J* = 4.2 Hz, 0.6 H), 1.67–1.57 (m, 2.6 H); ^{13}C NMR (101 MHz, $CDCl_3$, 1:3.6 *E*:*Z*) δ 145.29 (d, *J* = 28.3 Hz), 142.24 (d, *J* = 14.6 Hz), 136.4, 136.3, 127.2, 127.1, 122.8, 122.8, 122.5, 122.4, 119.8, 119.7, 118.3, 118.2, 111.6, 111.5, 111.4, 111.4, 80.7 (d, *J* = 169.9 Hz), 79.1 (d, *J* = 168.4 Hz), 61.2, 60.1, 24.1, 23.3, 16.7 (d, *J* = 2.9 Hz), 13.2 (d, *J* = 6.3 Hz); HRMS (CI) calcd for $C_{13}H_{16}FN_2O^+$ $[M + H]^+$ 235.1241, found 235.1242.

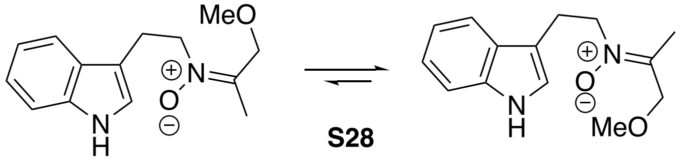

(*E/Z*)-*N*-(2-(1*H*-indol-3-yl)ethyl)-1-methoxypropan-2-imine oxide (S28). Prepared using the general procedure described above with **18** and methoxyacetone (5.0 equiv) in CH_2Cl_2/MeOH (1/1) at 23 ºC, ultimately yielding **S28** (0.118 g, 84% yield, 1:1.9 *E*:*Z*) as a yellow solid. **S28**: R_f = 0.49 (silica gel, CH_2Cl_2/MeOH = 9/1); IR (film) ν_{max} 3220, 2925, 1597, 1562, 1449, 1389, 1198, 1105, 798, 744 cm^{-1}; ^{1}H NMR (500 MHz, $CDCl_3$, 1:1.9 *E*:*Z*) δ 8.19 (br s, 1 H, exchangeable), 7.63 (d, *J* = 7.9 Hz, 1 H), 7.37 (d, *J* = 8.1 Hz, 1 H), 7.23–7.17 (m, 1 H), 7.16–7.11 (m, 1 H), 7.06 (d, *J* = 2.2 Hz, 1 H), 4.35 (s, 1.3 H), 4.19 (t, *J* = 6.9 Hz, 0.7 H), 4.11 (t, *J* = 6.9 Hz, 1.3 H), 3.61 (s, 0.7 H), 3.43–3.36 (m, 2 H), 3.28 (s, 1.8 H), 3.04 (s, 0.9 H), 2.09 (s, 1 H), 1.64 (s, 1.8 H); ^{13}C NMR (101 MHz, $CDCl_3$, 1:1.9 *E*:*Z*) δ 147.9, 145.6, 136.4, 136.4, 127.2 (2 C), 123.0, 122.9, 122.1 (2 C), 119.5 (2 C), 118.3, 118.3, 111.6 (2 C), 111.4 (2 C), 70.7, 70.1, 60.2, 60.1, 59.2, 58.3, 23.9, 23.3, 17.1, 13.7; HRMS (ESI) calcd for $C_{14}H_{19}N_2O_2^+$ $[M + H]^+$ 247.1441, found 247.1445.

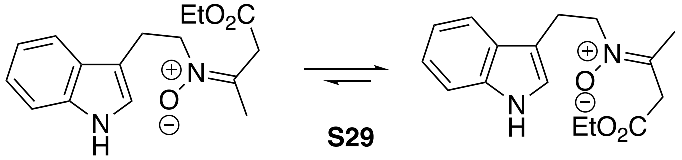

(*E/Z*)-*N*-(2-(1*H*-indol-3-yl)ethyl)-4-ethoxy-4-oxobutan-2-imine oxide (S29). Prepared using the general procedure described above with **18** and ethyl acetoacetate (5.0 equiv) in CH_2Cl_2/MeOH (1/1) at 23 ºC, ultimately yielding **S29** (0.144 g, 88% yield, 1:4.0 *E*:*Z*) as a white solid. **S29**: R_f = 0.46 (silica gel, CH_2Cl_2/MeOH = 9/1); IR (film) ν_{max} 3181, 2980, 1734, 1598, 1458, 1369, 1303, 1158, 1031, 744 cm^{-1}; ^{1}H NMR (500 MHz, $CDCl_3$, 1:4.0 *E*:*Z*) δ 8.34 (br s, 1 H, exchangeable), 7.61 (d, *J* = 7.8 Hz, 1 H), 7.41–7.34 (m, 1 H), 7.23–7.16 (m, 1 H), 7.16–7.04 (m, 2 H), 4.23–4.12 (m, 3.4 H), 4.05 (q, *J* = 7.1 Hz, 0.4 H), 3.50 (s, 1.6 H), 3.43–3.36 (m, 2 H), 2.95 (s, 0.4 H), 2.12 (s, 0.6 H), 1.77 (s, 2.4 H), 1.26 (t, *J* = 7.1 Hz, 2.3 H), 1.19 (t, *J* = 7.1 Hz, 0.6 H); ^{13}C NMR (101 MHz, $CDCl_3$, 1:4.0 *E*:*Z*) δ 168.5, 167.5, 141.4, 140.9, 136.4, 127.2, 123.2, 123.0, 122.1, 122.0, 119.5, 119.4, 118.3, 118.2, 111.6 (2 C), 111.3, 111.2, 61.8, 61.2, 60.3, 59.7, 39.4, 38.9, 23.6, 23.4, 19.4, 19.3, 14.2, 14.1; HRMS (ESI) calcd for $C_{16}H_{21}N_2O_3^+$ $[M + H]^+$ 289.1547, found 289.1556.

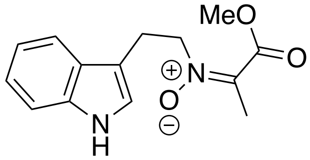

S30

(*E*)-*N*-(2-(1*H*-indol-3-yl)ethyl)-1-methoxy-1-oxopropan-2-imine oxide (S30). Prepared using the general procedure described above with **18** and methyl pyruvate (2.0 equiv) in CH_2Cl_2/MeOH (1/1) at 23 ºC for 2 h, ultimately yielding **S30** (0.146 g, 99% yield) as a pink

solid. **S30**: R_f = 0.54 (silica gel, CH_2Cl_2/MeOH = 9/1); IR (film) ν_{max} 3278, 1718, 1597, 1562, 1448, 1389, 1304, 1219, 1194, 1136, 744 cm^{-1}; ^1H NMR (500 MHz, $CDCl_3$) δ 8.06 (br s, 1 H, exchangeable), 7.71 (d, *J* = 7.6 Hz, 1 H), 7.39–7.33 (m, 1 H), 7.23–7.18 (m, 1 H), 7.17–7.12 (m, 1 H), 7.06 (d, *J* = 2.3 Hz, 1 H), 4.78 (t, *J* = 7.5 Hz, 2 H), 3.55 (s, 3 H), 3.36 (t, *J* = 7.7 Hz, 2 H), 2.19 (s, 3 H); ^{13}C NMR (101 MHz, $CDCl_3$) δ 162.8, 138.5, 136.3, 127.2, 122.8, 122.2, 119.6, 118.9, 111.6, 111.2, 64.3, 52.5, 25.0, 15.4; HRMS (ESI) calcd for $C_{14}H_{17}N_2O_3^+$ $[M + H]^+$ 261.1234, found 261.1235.

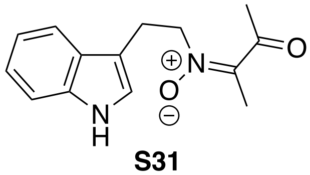

S31

(*E*)-*N*-(2-(1*H*-indol-3-yl)ethyl)-3-oxobutan-2-imine oxide (S31). Prepared using the general procedure described above with **18** and diacetyl (1.5 equiv) in CH_2Cl_2/MeOH (1/1) at 23 ºC for 2 h, ultimately yielding **S31** (0.137 g, 99% yield) as a yellow solid. **S31**: R_f = 0.65 (silica gel, CH_2Cl_2/MeOH = 9/1); IR (film) ν_{max} 3283, 1679, 1508, 1458, 1426, 1358, 1292, 1111, 970, 745 cm^{-1}; ^1H NMR (500 MHz, $CDCl_3$) δ 8.07 (br s, 1 H, exchangeable), 7.73 (d, *J* = 7.8 Hz, 1 H), 7.38–7.32 (m, 1 H), 7.24–7.12 (m, 2 H), 7.08 (d, *J* = 2.1 Hz, 1 H), 4.62 (t, *J* = 7.4 Hz, 2 H), 3.31 (t, *J* = 7.4 Hz, 2 H), 2.19 (s, 3 H), 1.96 (s, 3 H); ^{13}C NMR (101 MHz, $CDCl_3$) δ 193.3, 144.5, 136.2, 127.3, 122.8, 122.2, 119.7, 118.9, 111.7, 111.2, 64.1, 29.3, 24.9, 16.1; HRMS (ESI) calcd for $C_{14}H_{17}N_2O_2^+$ $[M + H]^+$ 245.1285, found 245.1288.

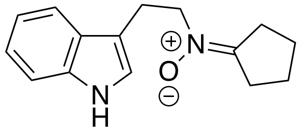

S32

***N*-(2-(1*H*-indol-3-yl)ethyl)cyclopentanimine oxide (S32).** Prepared using the general procedure described above with **18** and cyclopentanone (5.0 equiv) in CH_2Cl_2/MeOH (1/1) at 23 ºC, ultimately yielding **S32** (0.135 g, 98% yield) as a white solid. **S32**: R_f = 0.38 (silica gel, CH_2Cl_2/MeOH = 9/1); IR (film) ν_{max} 3179, 2964, 1597, 1563, 1449, 1395, 1340, 1136, 972, 798, 744 cm^{-1}; ^1H NMR (500 MHz, $CDCl_3$) δ 8.35 (br s, 1 H, exchangeable), 7.65 (d, *J* = 7.8 Hz, 1 H), 7.40–7.34 (m, 1 H), 7.22–7.16 (m, 1 H), 7.15–7.09 (m, 1 H), 7.07 (d, *J* = 2.4 Hz, 1 H), 4.02 (t, *J* = 6.7 Hz, 2 H), 3.40 (t, *J* = 6.7 Hz, 2 H), 2.58 (t, *J* = 7.4 Hz, 2 H), 1.92 (t, *J* = 7.2 Hz, 2 H), 1.63–1.54 (m, 2 H), 1.50–1.40 (m, 2 H); ^{13}C NMR (101 MHz, $CDCl_3$) δ 157.9, 136.5, 127.4, 123.0, 121.9, 119.3, 118.4, 111.6, 111.6, 61.7, 31.2, 31.0, 26.1, 24.4, 23.2; HRMS (CI) calcd for $C_{15}H_{19}N_2O^+$ $[M + H]^+$ 243.1492, found 243.1497.

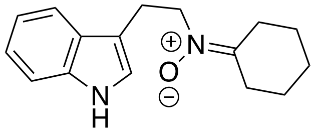

S33

***N*-(2-(1*H*-indol-3-yl)ethyl)cyclohexanimine oxide (S33).** Prepared using the general procedure described above with **18** and cyclohexanone (5.0 equiv) in CH_2Cl_2/MeOH (1/1) at 23 ºC, ultimately yielding **S33** (0.127 g, 87% yield) as a pale yellow solid. **S33**: R_f = 0.36 (silica gel, CH_2Cl_2/MeOH = 9/1); IR (film) ν_{max} 3170, 2932, 2861, 1597, 1561, 1448, 1342, 1198, 1133, 1105, 1073, 743 cm^{-1}; ^1H NMR (500 MHz, $CDCl_3$) δ 8.60 (br s, 1 H, exchangeable), 7.63 (d, *J* = 7.8 Hz, 1 H), 7.40–7.34 (m, 1 H), 7.21–7.15 (m, 1 H), 7.15–7.09 (m, 1 H), 7.06 (d, *J* = 2.2 Hz, 1 H), 4.17 (t, *J* = 6.7 Hz, 2 H), 3.38 (t, *J* = 6.7 Hz, 2 H), 2.69 (t, *J* = 6.5 Hz, 2 H), 1.99 (t, *J* = 6.5 Hz, 2 H), 1.54–1.46 (m, 2 H), 1.37–1.29 (m, 2 H), 1.09–1.00 (m, 2 H); ^{13}C NMR (101 MHz, $CDCl_3$) δ 151.6, 136.5, 127.4, 123.1, 122.0, 119.4, 118.3, 111.6 (2 C), 59.2, 29.9, 27.1, 25.2, 24.6, 24.5, 23.7; HRMS (ESI) calcd for $C_{16}H_{21}N_2O^+$ $[M + H]^+$ 257.1648, found 257.1658.

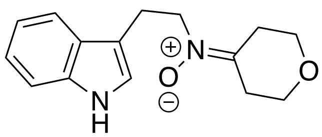

S34

***N*-(2-(1*H*-indol-3-yl)ethyl)tetrahydro-4*H*-pyran-4-imine oxide (S34).** Prepared using the general procedure described above with **18**

and tetrahydro-4*H*-pyran-4-one (5.0 equiv) in CH_2Cl_2/MeOH (1/1) at 23 ºC, ultimately yielding **S34** (0.120 g, 82% yield) as a yellow solid. **S34**: R_f = 0.34 (silica gel, CH_2Cl_2/MeOH = 9/1); IR (film) ν_{max} 3179, 2860, 1597, 1562, 1457, 1388, 1341, 1139, 1102, 1009, 746 cm^{-1}; ^1H NMR (500 MHz, $CDCl_3$) δ 8.38 (br s, 1 H, exchangeable), 7.65 (d, *J* = 7.8 Hz, 1 H), 7.41–7.35 (m, 1 H), 7.23–7.17 (m, 1 H), 7.15–7.10 (m, 1 H), 7.08 (d, *J* = 2.2 Hz, 1 H), 4.14 (t, *J* = 6.3 Hz, 2 H), 3.48 (t, *J* = 5.9 Hz, 2 H), 3.41 (t, *J* = 6.3 Hz, 2 H), 2.89 (t, *J* = 5.7 Hz, 2 H), 2.77 (t, *J* = 5.9 Hz, 2 H), 1.98 (t, *J* = 5.7 Hz, 2 H); ^{13}C NMR (101 MHz, $CDCl_3$) δ 145.8, 136.4, 127.5, 123.0, 122.4, 119.8, 118.3, 111.7, 111.6, 65.9, 65.5, 59.3, 30.3, 27.6, 23.5; HRMS (CI) calcd for $C_{15}H_{19}N_2O_2^+$ $[M + H]^+$ 259.1441, found 259.1444.

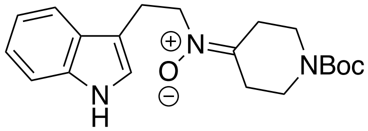

S35

***N*-(2-(1*H*-indol-3-yl)ethyl)-1-(*tert*-butoxycarbonyl)piperidin-4-imine oxide (S35).** Prepared using the general procedure described above with **18** and 1-Boc-4-piperidone (3.5 equiv) in CH_2Cl_2/MeOH (1/1) at 23 ºC, ultimately yielding **S35** (0.184 g, 91% yield) as a white solid. **S35**: R_f = 0.43 (silica gel, CH_2Cl_2/MeOH = 9/1); IR (film) ν_{max} 2972, 1695, 1597, 1564, 1394, 1363, 1223, 1148, 1000, 858, 744 cm^{-1}; ^1H NMR (500 MHz, $CDCl_3$) δ 8.18 (br s, 1 H, exchangeable), 7.64 (d, *J* = 7.8 Hz, 1 H), 7.39–7.33 (m, 1 H), 7.22–7.17 (m, 1 H), 7.15–7.09 (m, 1 H), 7.07 (d, *J* = 2.2 Hz, 1 H), 4.14 (t, *J* = 6.3 Hz, 2 H), 3.40 (t, *J* = 6.3 Hz, 2 H), 3.30–3.19 (m, 2 H), 2.75 (t, *J* = 5.7 Hz, 2 H), 2.69 (t, *J* = 6.1 Hz, 2 H), 1.99 (t, *J* = 6.0 Hz, 2 H), 1.40 (s, 9 H); ^{13}C NMR (101 MHz, $CDCl_3$) δ 154.5, 146.6, 136.3, 127.5, 122.8, 122.5, 119.8, 118.4, 111.9, 111.6, 80.2, 59.8, 41.2, 40.7, 28.9, 28.5, 27.1, 23.6; HRMS (ESI) calcd for $C_{20}H_{28}N_3O_3^+$ $[M + H]^+$ 358.2125, found 358.2133.

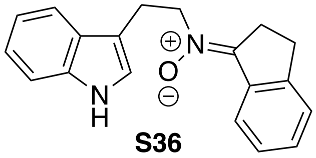
S36

(*Z*)-*N*-(2-(1*H*-indol-3-yl)ethyl)-2,3-dihydro-1*H*-inden-1-imine oxide (S36). Prepared using the general procedure described above with **18** and 1-indanone (3.0 equiv) in MeOH at 50 ºC, ultimately yielding **S36** (0.149 g, 90% yield) as a brown solid. **S36**: R_f = 0.49 (silica gel, CH_2Cl_2/MeOH = 9/1); IR (film) ν_{max} 2850, 1595, 1561, 1445, 1400, 1174, 1097, 1067, 760, 747 cm^{-1}; ^1H NMR (500 MHz, $CDCl_3$) δ 8.96 (d, *J* = 7.3 Hz, 1 H), 8.09 (br s, 1 H, exchangeable), 7.65 (d, *J* = 7.9 Hz, 1 H), 7.41–7.30 (m, 3 H), 7.24–7.16 (m, 2 H), 7.13–7.08 (m, 1 H), 7.06 (d, *J* = 2.2 Hz, 1 H), 4.17 (t, *J* = 7.0 Hz, 2 H), 3.50 (t, *J* = 7.0 Hz, 2 H), 2.93–2.86 (m, 2 H), 2.59–2.53 (m, 2 H); ^{13}C NMR (101 MHz, $CDCl_3$) δ 150.0, 148.1, 136.3, 134.8, 131.1, 127.4, 127.2, 127.2, 124.7, 122.9, 122.3, 119.7, 118.5, 112.0, 111.5, 62.3, 29.1, 28.9, 23.4; HRMS (CI) calcd for $C_{19}H_{19}N_2O^+$ $[M + H]^+$ 291.1492, found 291.1503.

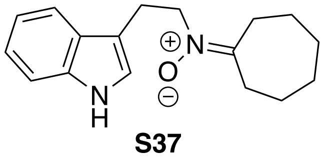
S37

***N*-(2-(1*H*-indol-3-yl)ethyl)cycloheptanimine oxide (S37).** Prepared using the general procedure described above with **18** and cycloheptanone (5.0 equiv) in CH_2Cl_2/MeOH (1/1) at 23 ºC, ultimately yielding **S37** (0.153 g, 99% yield) as a yellow solid. **S37**: R_f = 0.51 (silica gel, CH_2Cl_2/MeOH = 9/1); IR (film) ν_{max} 3173, 2924, 2854, 1597, 1564, 1449, 1211, 1140, 1105, 743 cm^{-1}; ^1H NMR (500 MHz, $CDCl_3$) δ 8.37 (br s, 1 H, exchangeable), 7.63 (d, *J* = 7.8 Hz, 1 H), 7.41–7.33 (m, 1 H), 7.22–7.16 (m, 1 H), 7.15–7.10 (m, 1 H), 7.07 (d, *J* = 2.3 Hz, 1 H), 4.17 (t, *J* = 7.0 Hz, 2 H), 3.40 (t, *J* = 6.9 Hz, 2 H), 2.79–2.71 (m, 2 H), 2.20–2.14 (m, 2 H), 1.64–1.57 (m, 2 H), 1.46–1.38 (m, 2 H), 1.35–1.28 (m, 2 H), 1.25–1.19 (m, 2 H); ^{13}C NMR (101 MHz, $CDCl_3$) δ 155.0, 136.5, 127.4, 123.0, 121.9,

119.3, 118.3, 111.6, 111.4, 59.4, 31.9, 31.2, 29.7, 29.5, 26.1, 24.6, 23.8; HRMS (CI) calcd for $C_{17}H_{23}N_2O^+$ $[M + H]^+$ 271.1805, found 271.1815.

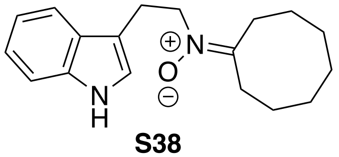
S38

***N*-(2-(1*H*-indol-3-yl)ethyl)cyclooctanimine oxide (S38).** Prepared using the general procedure described above with **18** and cyclooctanone (5.0 equiv) in CH_2Cl_2/MeOH (1/1) at 23 ºC, ultimately yielding **S38** (0.161 g, 99% yield) as a yellow solid. **S38**: R_f = 0.49 (silica gel, CH_2Cl_2/MeOH = 9/1); IR (film) ν_{max} 3173, 2924, 1597, 1562, 1448, 1400, 1355, 1217, 1142, 1109, 743 cm^{-1}; ^1H NMR (500 MHz, $CDCl_3$) δ 8.25 (br s, 1 H, exchangeable), 7.64 (d, *J* = 7.8 Hz, 1 H), 7.41–7.34 (m, 1 H), 7.23–7.17 (m, 1 H), 7.16–7.10 (m, 1 H), 7.08 (d, *J* = 2.4 Hz, 1 H), 4.13 (t, *J* = 7.2 Hz, 2 H), 3.42 (t, *J* = 7.2 Hz, 2 H), 2.69–2.60 (m, 2 H), 2.23–2.13 (m, 2 H), 1.80–1.73 (m, 2 H), 1.55–1.47 (m, 2 H), 1.45–1.38 (m, 2 H), 1.33–1.27 (m, 2 H), 1.26–1.20 (m, 2 H); ^{13}C NMR (101 MHz, $CDCl_3$) δ 154.5, 136.6, 127.3, 123.1, 121.9, 119.2, 118.3, 111.6, 111.4, 59.0, 30.7, 29.7, 28.1, 26.8, 25.9, 25.4, 23.5, 23.3; HRMS (ESI) calcd for $C_{18}H_{25}N_2O^+$ $[M + H]^+$ 285.1961, found 285.1972.

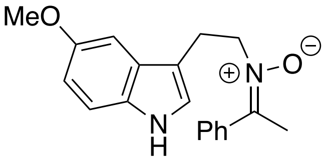
S39

(*E*)-*N*-(2-(5-methoxy-1*H*-indol-3-yl)ethyl)-1-phenylethan-1-imine oxide (S39). Prepared using the general procedure described above with **S7** and acetophenone (5.0 equiv) in CH_2Cl_2/MeOH (1/1) at 23 ºC, ultimately yielding **S39** (0.131 g, 88% yield) as a white solid. **S39**: R_f = 0.44 (silica gel, CH_2Cl_2/MeOH = 9/1); IR (film) ν_{max} 3325, 1597, 1486, 1447, 1394, 1216, 1071, 858, 798, 700 cm^{-1}; ^1H NMR (500 MHz, $CDCl_3$) δ 8.06 (br s, 1 H, exchangeable), 7.26–7.22 (m, 2 H), 7.19–7.13 (m, 2 H), 6.97 (d, *J* = 2.4 Hz, 1 H), 6.82 (dd, *J* = 8.7, 2.4 Hz, 1 H), 6.75–6.70 (m, 3 H), 4.07 (t, *J* = 6.9 Hz, 2 H), 3.74 (s, 3 H), 3.33 (t, *J* = 7.0 Hz, 2 H), 2.35 (s, 3 H); ^{13}C NMR (101 MHz, $CDCl_3$) δ 154.0, 148.4, 136.2, 131.4, 128.8, 128.7, 127.8, 127.3, 123.5, 112.6, 112.0, 111.5, 100.0, 60.1, 56.0, 23.9, 20.8; HRMS (CI) calcd for $C_{19}H_{21}N_2O_2^+$ $[M + H]^+$ 309.1598, found 309.1599.

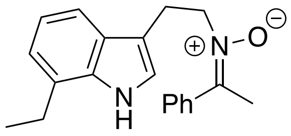
S40

(*E*)-*N*-(2-(7-ethyl-1*H*-indol-3-yl)ethyl)-1-phenylethan-1-imine oxide (S40). Prepared using the general procedure described above with **S9** and acetophenone (5.0 equiv) in CH_2Cl_2/MeOH (1/1) at 23 ºC, ultimately yielding **S40** (0.131 g, 87% yield) as a pale yellow solid. **S40**: R_f = 0.50 (silica gel, CH_2Cl_2/MeOH = 9/1); IR (film) ν_{max} 3261, 1597, 1564, 1485, 1448, 1395, 1220, 1080, 858, 798 cm^{-1}; ^1H NMR (500 MHz, $CDCl_3$) δ 8.08 (br s, 1 H, exchangeable), 7.28–7.25 (m, 1 H), 7.22–7.15 (m, 2 H), 7.09 (d, *J* = 7.8 Hz, 1 H), 7.02–6.98 (m, 2 H), 6.97–6.92 (m, 1 H), 6.80–6.74 (m, 2 H), 4.07 (t, *J* = 7.2 Hz, 2 H), 3.36 (t, *J* = 7.2 Hz, 2 H), 2.86 (q, *J* = 7.6 Hz, 2 H), 2.37 (s, 3 H), 1.36 (t, *J* = 7.6 Hz, 3 H); ^{13}C NMR (101 MHz, $CDCl_3$) δ 148.4, 136.2, 135.1, 128.8, 128.7, 128.6, 127.2, 126.8, 122.4, 120.6, 119.7, 116.2, 112.1, 60.4, 24.1, 24.0, 20.8, 14.2; HRMS (ESI) calcd for $C_{20}H_{23}N_2O^+$ $[M + H]^+$ 307.1805, found 307.1808.

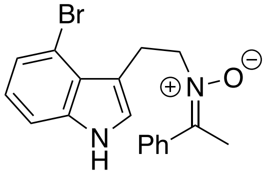
S41

(*E*)-*N*-(2-(4-bromo-1*H*-indol-3-yl)ethyl)-1-phenylethan-1-imine oxide (S41). Prepared using the general procedure described above with **S11** and acetophenone (5.0 equiv) in CH_2Cl_2/MeOH (1/1) at 23 ºC, ultimately yielding **S41** (0.127 mg, 91% yield) as a pale yellow solid. **S41**: R_f = 0.49 (silica gel, CH_2Cl_2/MeOH = 9/1); IR (film) ν_{max} 3155, 1597, 1562, 1424,

1333, 1221, 1193, 1156, 1071, 1044, 738, 700 cm^{-1}; ^{1}H NMR (500 MHz, $CDCl_3$) δ 9.36 (br s, 1 H, exchangeable), 7.37 (dd, *J* = 8.1, 0.9 Hz, 1 H), 7.22–7.15 (m, 2 H), 7.09 (dd, *J* = 7.6, 0.9 Hz, 1 H), 7.05–7.00 (m, 2 H), 6.97 (t, *J* = 7.8 Hz, 1 H), 6.58–6.52 (m, 2 H), 4.25 (t, *J* = 6.4 Hz, 2 H), 3.52 (t, *J* = 6.4 Hz, 2 H), 2.31 (s, 3 H); ^{13}C NMR (101 MHz, $CDCl_3$) δ 149.7, 138.0, 135.6, 128.8, 128.6, 127.0, 125.9, 125.6, 123.5, 122.6, 114.0, 111.5, 111.0, 61.3, 24.2, 20.8; HRMS (ESI) calcd for $C_{18}H_{18}BrN_2O^+$ $[M + H]^+$ 357.0597, found 357.0610.

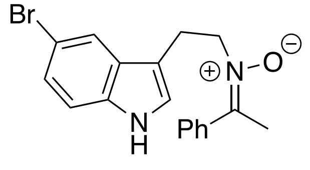

S42

(*E*)-*N*-(2-(5-bromo-1*H*-indol-3-yl)ethyl)-1-phenylethan-1-imine oxide (S42). Prepared using the general procedure described above with **S13** and acetophenone (5.0 equiv) in CH_2Cl_2/MeOH (1/1) at 23 ºC, ultimately yielding **S42** (0.114 g, 81% yield) as a white solid. **S42**: R_f = 0.68 (silica gel, CH_2Cl_2/MeOH = 9/1); IR (film) ν_{max} 3199, 1597, 1564, 1449, 1394, 1219, 1159, 1071, 882, 858, 798, 699 cm^{-1}; ^{1}H NMR (500 MHz, $CDCl_3$) δ 8.91 (br s, 1 H, exchangeable), 7.33–7.29 (m, 1 H), 7.28–7.27 (m, 1 H), 7.25–7.23 (m, 1 H), 7.23–7.17 (m, 3 H), 6.99 (d, *J* = 2.4 Hz, 1 H), 6.75–6.67 (m, 2 H), 4.06 (t, *J* = 6.6 Hz, 2 H), 3.29 (t, *J* = 6.9 Hz, 2 H), 2.36 (s, 3 H); ^{13}C NMR (101 MHz, $CDCl_3$) δ 148.8, 135.9, 135.0, 129.2, 129.2, 128.8, 127.1, 124.9, 124.2, 121.1, 112.8, 112.8, 111.4, 60.0, 23.8, 20.8; HRMS (ESI) calcd for $C_{18}H_{18}BrN_2O^+$ $[M + H]^+$ 357.0597, found 357.0598.

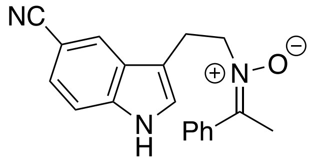

S43

(*E*)-*N*-(2-(5-cyano-1*H*-indol-3-yl)ethyl)-1-phenylethan-1-imine oxide (S43). Prepared using the general procedure described above with **S15** and acetophenone (5.0 equiv) in CH_2Cl_2/MeOH (1/1) at 23 ºC, ultimately yielding **S43** (0.094 g, 62% yield) as a white solid. **S43**: R_f = 0.40 (silica gel, CH_2Cl_2/MeOH = 9/1); IR (film) ν_{max} 3289, 2362, 2218, 1597, 1572, 1479, 1401, 1223, 1138, 1069, 802 cm^{-1}; ^{1}H NMR (500 MHz, $CDCl_3$) δ 9.71 (br s, 1 H, exchangeable), 7.46 (d, *J* = 8.4 Hz, 1 H), 7.42–7.39 (m, 1 H), 7.39–7.32 (m, 2 H), 7.24–7.18 (m, 2 H), 7.12 (d, *J* = 2.3 Hz, 1 H), 6.75–6.70 (m, 2 H), 4.10 (t, *J* = 6.9 Hz, 2 H), 3.33 (t, *J* = 6.9 Hz, 2 H), 2.35 (s, 3 H); ^{13}C NMR (101 MHz, $CDCl_3$) δ 149.2, 138.1, 135.8, 129.5, 128.9, 127.2, 127.1, 125.3, 124.9, 124.1, 120.7, 112.4, 112.4, 102.4, 59.8, 23.6, 20.7; HRMS (CI) calcd for $C_{19}H_{18}N_3O^+$ $[M + H]^+$ 304.1444, found 304.1446.

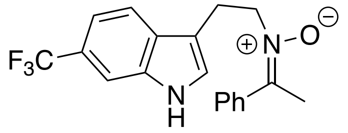

S44

(*E*)-1-phenyl-*N*-(2-(6-(trifluoromethyl)-1*H*-indol-3-yl)ethyl)ethan-1-imine oxide (S44). Prepared using the general procedure described above with **S17** and acetophenone (5.0 equiv) in CH_2Cl_2/MeOH (1/1) at 23 ºC, ultimately yielding **S44** (0.125 g, 88% yield) as a white solid. **S44**: R_f = 0.57 (silica gel, CH_2Cl_2/MeOH = 9/1); IR (film) ν_{max} 3182, 1597, 1564, 1448, 1393, 1336, 1220, 1158, 1111, 1072, 700 cm^{-1}; ^{1}H NMR (500 MHz, $CDCl_3$) δ 8.65 (br s, 1 H, exchangeable), 7.69–7.62 (m, 1 H), 7.29 (d, *J* = 8.4 Hz, 1 H), 7.26–7.22 (m, 1 H), 7.19 (dd, *J* = 8.4, 1.6 Hz, 1 H), 7.17–7.11 (m, 3 H), 6.70–6.63 (m, 2 H), 4.09 (t, *J* = 6.9 Hz, 2 H), 3.37 (t, *J* = 6.8 Hz, 2 H), 2.33 (s, 3 H); ^{13}C NMR (101 MHz, $CDCl_3$) δ 149.5, 135.7, 135.4, 129.6, 129.1, 128.8, 127.0, 125.8, 125.4 (q, *J* = 271.4 Hz), 123.9 (q, *J* = 31.8 Hz), 118.7, 115.9, 111.6, 109.0, 60.0, 23.6, 20.8; HRMS (CI) calcd for $C_{19}H_{18}F_3N_2O^+$ $[M + H]^+$ 347.1366, found 347.1370.

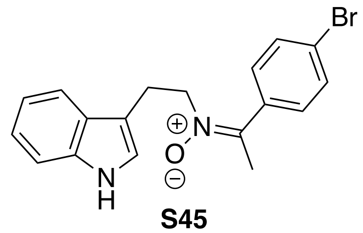

S45

(*E*)-*N*-(2-(1*H*-indol-3-yl)ethyl)-1-(4-bromophenyl)ethan-1-imine oxide (S45). Prepared using the general procedure described

above with **18** and 4′-bromoacetophenone (2.5 equiv) in MeOH at 50 ºC, ultimately yielding **S45** (0.170 g, 84% yield) as a white solid. **S45**: R_f = 0.60 (silica gel, CH_2Cl_2/MeOH = 9/1); IR (film) 3184, 1577, 1484, 1457, 1221, 1165, 1075, 1009, 825, 742 ν_{max} cm^{-1}; ^{1}H NMR (400 MHz, $CDCl_3$) δ 8.25 (br s, 1 H, exchangeable), 7.37 (d, *J* = 8.1 Hz, 1 H), 7.28–7.24 (m, 1 H), 7.23–7.16 (m, 3 H), 7.05–7.00 (m, 1 H), 6.99 (d, *J* = 2.3 Hz, 1 H), 6.48–6.39 (m, 2 H), 4.06 (t, *J* = 6.2 Hz, 1 H), 3.35 (t, *J* = 6.2 Hz, 1 H) 2.29 (s, 3 H); ^{13}C NMR (101 MHz, $CDCl_3$) δ 147.3, 136.3, 134.9, 131.8, 128.9, 127.4, 123.1, 122.8, 122.2, 119.6, 118.5, 111.6, 111.3, 60.4, 23.7, 20.6; HRMS (CI) calcd for $C_{18}H_{18}BrN_2O^+$ $[M + H]^+$ 357.0597, found 357.0596.

E. General Procedure for Racemic Pictet–Spengler Reactions of Nitrones.

To a solution of nitrone (0.25 mmol, 1.0 equiv) in CH_2Cl_2 (1.25 mL, dried over 4Å MS beads) at 23 ºC was added molecular sieves (4Å, powder, ~150 mg), and the resultant slurry was stirred for 10 min under an argon atmosphere. Next, the acyl chloride (0.26 mmol, 1.05 equiv) was added and the reaction mixture was stirred for 30 min at 23 ºC. Upon completion, the contents were quenched by the addition of saturated aqueous $NaHCO_3$ (5 mL), poured into a separatory funnel, and extracted with CH_2Cl_2 (3 × 5 mL). The combined organic extracts were then dried (Na_2SO_4), filtered, and concentrated. The resultant crude material was purified by flash column chromatography (silica gel, hexanes/EtOAc = 1/0→2/1) to yield **20**–**47**. *Note:* These reactions must be run under strictly anhydrous conditions to avoid hydrolysis of the *N*-acyloxyiminium species.

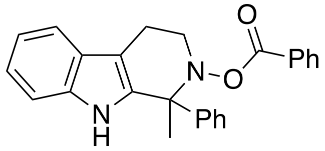

20a

1-methyl-1-phenyl-1,3,4,9-tetrahydro-2*H*-pyrido[3,4-*b*]indol-2-yl benzoate (20a). Prepared using the general procedure described above with **19** and benzoyl chloride, ultimately yielding **20a** (0.083 g, 86% yield) as a white solid. **20a**: R_f = 0.50 (silica gel, hexanes/EtOAc = 4/1); IR (film) ν_{max} 3363, 1726, 1598, 1450, 1272, 1092, 1063, 1023, 745, 700 cm^{-1}; ^{1}H NMR (500 MHz, $CDCl_3$) δ 8.00–7.84 (m, 3 H, 1 exchangeable), 7.63 (d, *J* = 7.7 Hz, 1 H), 7.53 (t, *J* = 7.4 Hz, 1 H), 7.43–7.35 (m, 5 H), 7.31–7.24 (m, 4 H), 7.23–7.19 (m, 1 H), 3.69–3.57 (m, 1 H), 3.44–3.26 (m, 1 H), 3.22–3.14 (m, 1 H), 2.93–2.78 (m, 1 H), 1.97 (s, 3 H); ^{13}C NMR (101 MHz, $CDCl_3$) δ 165.5, 136.2, 135.2, 133.2, 129.6, 129.3, 128.5, 128.4, 128.0, 127.9 (2 C), 127.1, 122.2, 119.8, 118.8, 111.2, 108.6, 65.9, 48.1, 25.2, 18.4; HRMS (CI) calcd for $C_{25}H_{23}N_2O_2^+$ $[M + H]^+$ 383.1754, found 383.1753.

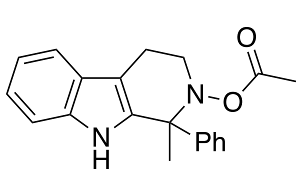

20b

1-methyl-1-phenyl-1,3,4,9-tetrahydro-2*H*-pyrido[3,4-*b*]indol-2-yl acetate (20b). Prepared using the general procedure described above with **19** and acetyl chloride, ultimately yielding **20b** (0.069 g, 86% yield) as a white solid. **20b**: R_f = 0.40 (silica gel, hexanes/EtOAc = 4/1); IR (film) ν_{max} 3363, 1238, 1744, 1597, 1448, 1366, 1297, 1221, 745, 701 cm^{-1}; ^{1}H NMR (500 MHz, $CDCl_3$) δ 7.76 (br s, 1 H, exchangeable), 7.57 (d, *J* = 7.8 Hz, 1 H), 7.37 (d, *J* = 8.0 Hz, 1 H), 7.35–7.30 (m, 2 H), 7.30–7.24 (m, 3 H), 7.24–7.20 (m, 1 H), 7.19–7.14 (m, 1 H), 3.58–3.44 (m, 1 H), 3.31–3.17 (m, 1 H), 3.10–3.02 (m, 1 H), 2.87–2.71 (m, 1 H), 1.98 (s, 3 H), 1.88 (s, 3 H); ^{13}C NMR (101 MHz, $CDCl_3$) δ 170.2, 136.1, 135.2, 128.4, 127.9 (2 C), 127.8, 127.0, 122.2, 119.8, 118.7, 111.1, 108.5, 65.5, 47.9, 24.7, 19.6, 18.3; HRMS (CI) calcd for $C_{20}H_{21}N_2O_2^+$ $[M + H]^+$ 321.1598, found 321.1601.

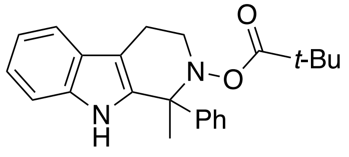

20c

1-methyl-1-phenyl-1,3,4,9-tetrahydro-2*H*-pyrido[3,4-*b*]indol-2-yl pivalate (20c). Prepared using the general procedure described above with **19** and pivaloyl chloride, ultimately yielding **20c** (0.080 g, 88% yield) as a white solid. **20c**: R_f = 0.49 (silica gel, hexanes/EtOAc = 4/1); IR (film) ν_{max} 3363, 2974, 1732, 1597, 1450, 1396, 1276, 1118, 1024, 744, 700 cm^{-1}; ^{1}H NMR (500 MHz, $CDCl_3$) δ 7.83 (br s, 1 H, exchangeable), 7.59 (d, *J* = 7.7 Hz, 1 H), 7.41–7.33 (m, 3 H), 7.31–7.24 (m, 3 H), 7.24–7.15 (m, 2 H), 3.56–3.43 (m, 1 H), 3.34–3.19 (m, 1 H), 3.08–2.99 (m, 1 H), 2.92–2.77 (m, 1 H), 1.91 (s, 3 H), 1.11 (s, 9 H); ^{13}C NMR (101 MHz, $CDCl_3$) δ 176.8, 136.2, 135.6, 128.3, 127.9 (2 C), 127.8, 127.0, 122.0, 119.7, 118.7, 111.1, 108.3, 65.6, 47.8, 39.0, 27.3, 24.5, 18.5; HRMS (CI) calcd for $C_{23}H_{27}N_2O_2^+$ $[M + H]^+$ 363.2067, found 363.2066.

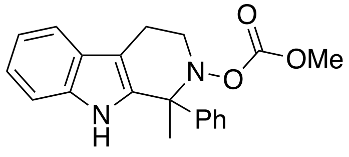

20d

Methyl (1-methyl-1-phenyl-1,3,4,9-tetrahydro-2*H*-pyrido[3,4-*b*]indol-2-yl) carbonate (20d). Prepared using the general procedure described above with **19** and methyl chloroformate, ultimately yielding **20d** (0.074 g, 87% yield) as a white solid. **20d**: R_f = 0.39 (silica gel, hexanes/EtOAc = 4/1); IR (film) ν_{max} 3380, 1755, 1597, 1440, 1229, 1128, 940, 858, 745, 700 cm^{-1}; ^{1}H NMR (500 MHz, $CDCl_3$) δ 7.74 (br s, 1 H, exchangeable), 7.55 (d, *J* = 7.8 Hz, 1 H), 7.38–7.30 (m, 3 H), 7.30–7.25 (m, 3 H), 7.23–7.18 (m, 1 H), 7.18–7.12 (m, 1 H), 3.78 (s, 3 H), 3.63–3.51 (m, 1 H), 3.32–3.19 (m, 1 H), 3.14–3.06 (m, 1 H), 2.86–2.73 (m, 1 H), 1.92 (s, 3 H); ^{13}C NMR (101 MHz, $CDCl_3$) δ 156.2, 136.2, 134.8, 128.4, 127.9, 127.8, 127.7, 127.0, 122.2, 119.8, 118.7, 111.1, 108.4, 66.1, 55.2, 48.1, 24.4, 18.3; HRMS (CI) calcd for $C_{20}H_{21}N_2O_3^+$ $[M + H]^+$ 337.1547, found 337.1550.

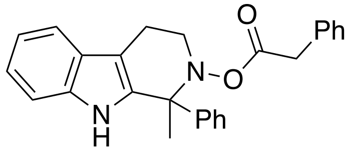

20e

1-methyl-1-phenyl-1,3,4,9-tetrahydro-2*H*-pyrido[3,4-*b*]indol-2-yl 2-phenylacetate (20e). Prepared using the general procedure described above with **19** and phenylacetyl chloride, ultimately yielding **20e** (0.095 g, 95% yield) as a white solid. **20e**: R_f = 0.44 (silica gel, hexanes/EtOAc = 4/1); IR (film) ν_{max} 3374, 3058, 1745, 1453, 1298, 1232, 1116, 1025, 745, 700 cm^{-1}; ^{1}H NMR (500 MHz, $CDCl_3$) δ 7.70 (br s, 1 H, exchangeable), 7.56 (d, *J* = 7.7 Hz, 1 H), 7.35 (d, *J* = 8.0 Hz, 1 H), 7.32–7.20 (m, 9 H), 7.19–7.14 (m, 3 H), 3.53 (s, 2 H), 3.48–3.41 (m, 1 H), 3.27–3.15 (m, 1 H), 3.05–2.92 (m, 1 H), 2.84–2.72 (m, 1 H), 1.78 (s, 3 H); ^{13}C NMR (101 MHz, $CDCl_3$) δ 170.4, 136.2, 135.3, 133.7, 129.3, 128.7, 128.3 (2 C), 127.8 (2 C), 127.2, 127.0, 122.1, 119.8, 118.7, 111.1, 108.4, 65.7, 48.0, 40.3, 24.3, 18.5; HRMS (ESI) calcd for $C_{26}H_{24}N_2O_2^+$ $[M + H]^+$ 397.1911, found 397.1912.

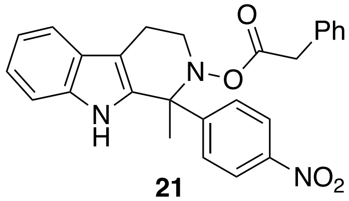

21

1-methyl-1-(4-nitrophenyl)-1,3,4,9-tetrahydro-2*H*-pyrido[3,4-*b*]indol-2-yl 2-phenylacetate (21). Prepared using the general procedure described above with **S18** and phenylacetyl chloride, ultimately yielding **21** (0.107 g, 97% yield) as a bright yellow solid. **21**: R_f = 0.34 (silica gel, hexanes/EtOAc = 4/1); IR (film) ν_{max} 3245, 1598, 1561, 1518, 1400, 1349, 1219, 1069, 857, 744 cm^{-1}; ^{1}H NMR (500 MHz, $CDCl_3$) δ 8.03 (d, *J* = 8.4 Hz, 2 H), 7.66 (br s, 1 H, exchangeable), 7.57 (d, *J* = 7.8 Hz, 1 H), 7.53–7.39 (m, 2 H), 7.35 (d, *J* = 8.0 Hz, 1 H), 7.28–7.21 (m, 4 H), 7.20–7.11 (m, 3

H), 3.57–3.46 (m, 3 H), 3.23–3.12 (m, 1 H), 3.05–2.95 (m, 1 H), 2.92–2.80 (m, 1 H), 1.79 (s, 3 H); ^{13}C NMR (101 MHz, $CDCl_3$) δ 170.0, 147.3, 136.4, 134.0, 133.4, 129.1, 128.8, 128.7 (2 C), 127.4, 126.7, 123.4, 122.7, 120.1, 118.9, 111.3, 108.8, 65.4, 48.3, 40.3, 23.7, 18.7; HRMS (ESI) calcd for $C_{26}H_{24}N_3O_4^+$ [M + H]$^+$ 442.1761, found 442.1759.

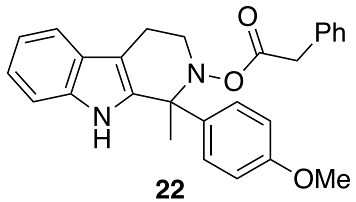

22

1-(4-methoxyphenyl)-1-methyl-1,3,4,9-tetrahydro-2*H*-pyrido[3,4-*b*]indol-2-yl 2-phenylacetate (22). Prepared using the general procedure described above with **S19** and phenylacetyl chloride, ultimately yielding **22** (0.088 g, 83% yield) as a yellow solid. **22**: R_f = 0.62 (silica gel, hexanes/EtOAc = 2/1); IR (film) ν_{max} 3373, 1746, 1607, 1510, 1454, 1299, 1249, 1180, 1116, 744 cm^{-1}; ^{1}H NMR (500 MHz, $CDCl_3$) δ 7.69 (br s, 1 H, exchangeable), 7.56 (d, J = 7.7 Hz, 1 H), 7.34 (d, J = 7.9 Hz, 1 H), 7.30–7.09 (m, 9 H), 6.79–6.72 (m, 2 H), 3.77 (s, 3 H), 3.53 (s, 2 H), 3.47–3.39 (m, 1 H), 3.28–3.17 (m, 1 H), 3.02–2.92 (m, 1 H), 2.87–2.71 (m, 1 H), 1.77 (s, 3 H); ^{13}C NMR (101 MHz, $CDCl_3$) δ 170.4, 159.1, 136.2, 135.6, 133.8, 129.3, 129.1 (2 C), 128.6, 127.2, 127.0, 122.1, 119.7, 118.6, 113.5, 111.1, 108.2, 65.2, 55.4, 47.9, 40.3, 24.3, 18.6; HRMS (ESI) calcd for $C_{27}H_{27}N_2O_3^+$ [M + H]$^+$ 427.2016, found 427.2013.

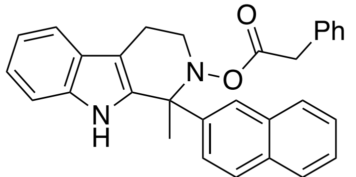

23

1-methyl-1-(naphthalen-2-yl)-1,3,4,9-tetrahydro-2*H*-pyrido[3,4-*b*]indol-2-yl 2-phenylacetate (23). Prepared using the general procedure described above with **S20** and phenylacetyl chloride, ultimately yielding **23** (0.106 g, 95% yield) as a pale yellow solid. **23**: R_f = 0.44 (silica gel, hexanes/EtOAc = 4/1); IR (film) ν_{max} 3373, 3058, 1745, 1597, 1454, 1297, 1230, 1115, 744, 696 cm^{-1}; ^{1}H NMR (500 MHz, $CDCl_3$) δ 7.80 (d, J = 7.8 Hz, 1 H), 7.77–7.68 (m, 3 H), 7.67–7.62 (m, 1 H), 7.59 (d, J = 7.6 Hz, 1 H), 7.55 (br s, 1 H, exchangeable), 7.49–7.41 (m, 2 H), 7.36 (d, J = 7.9 Hz, 1 H), 7.26–7.22 (m, 1 H), 7.21–7.13 (m, 4 H), 7.12–7.05 (m, 2 H), 3.56–3.46 (m, 3 H), 3.35–3.24 (m, 1 H), 3.05–2.96 (m, 1 H), 2.91–2.78 (m, 1 H), 1.87 (s, 3 H); ^{13}C NMR (101 MHz, $CDCl_3$) δ 170.4, 136.3, 135.5, 133.7, 132.9, 129.1, 128.7, 128.6, 128.4, 128.2 (2 C), 127.6, 127.2, 127.0, 126.5, 126.4, 126.2, 125.9, 122.2, 119.8, 118.7, 111.2, 108.4, 65.8, 48.0, 40.3, 23.9, 18.7; HRMS (CI) calcd for $C_{30}H_{27}N_2O_2^+$ [M + H]$^+$ 447.2067, found 447.2064.

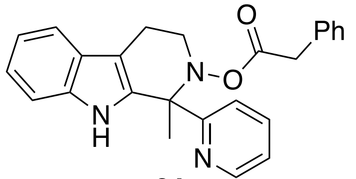

24

1-methyl-1-(pyridin-2-yl)-1,3,4,9-tetrahydro-2*H*-pyrido[3,4-*b*]indol-2-yl 2-phenylacetate (24). Prepared using the general procedure described above with **S21** and phenylacetyl chloride, ultimately yielding **24** (0.087 g, 87% yield) as a yellow solid. **24**: R_f = 0.21 (silica gel, hexanes/EtOAc = 4/1); IR (film) ν_{max} 3059, 2934, 1754, 1590, 1455, 1431, 1300, 1233, 1112, 742 cm^{-1}; ^{1}H NMR (500 MHz, $CDCl_3$, 45 ºC) δ 9.14 (br s, 1 H, exchangeable), 8.60–8.49 (m, 1 H), 7.79 (d, J = 8.0 Hz, 1 H), 7.56–7.47 (m, 2 H), 7.34 (d, J = 8.1 Hz, 1 H), 7.23–7.14 (m, 4 H), 7.14–7.05 (m, 4 H), 3.74–3.63 (m, 2 H), 3.53–3.40 (m, 2 H), 3.10–3.02 (m, 1 H), 2.92–2.86 (m, 1 H), 1.90 (s, 3 H); ^{13}C NMR (126 MHz, $CDCl_3$, 45 ºC) δ 169.9, 163.4, 148.4, 137.1, 136.6, 136.4, 133.5, 129.2, 128.6, 127.2, 126.8, 122.2, 121.8, 120.9, 119.4, 118.5, 111.3, 106.5, 67.3, 48.5, 40.4, 22.2, 19.3; HRMS (ESI) calcd for $C_{25}H_{24}N_3O_2^+$ [M + H]$^+$ 398.1863, found 398.1867.

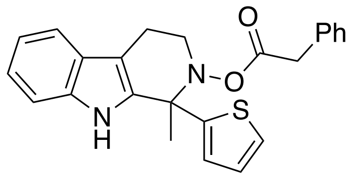

25

1-methyl-1-(thiophen-2-yl)-1,3,4,9-tetrahydro-2*H*-pyrido[3,4-*b*]indol-2-yl 2-phenylacetate (25). Prepared using the general procedure described above with **S22** and phenylacetyl chloride, ultimately yielding **25** (0.096 g, 96% yield) as a yellow solid. **25**: R_f = 0.46 (silica gel, hexanes/EtOAc = 4/1); IR (film) ν_{max} 3379, 1749, 1598, 1454, 1299, 1232, 1114, 1074, 743, 697 cm^{-1}; ^{1}H NMR (500 MHz, $CDCl_3$) δ 7.68 (br s, 1 H, exchangeable), 7.53 (d, *J* = 7.8 Hz, 1 H), 7.37–7.25 (m, 5 H), 7.24–7.17 (m, 3 H), 7.16–7.11 (m, 1 H), 6.92–6.81 (m, 1 H), 6.76–6.57 (m, 1 H), 3.62–3.54 (m, 2 H), 3.50–3.34 (m, 2 H), 3.09–2.93 (m, 1 H), 2.92–2.84 (m, 1 H), 1.86 (s, 3 H); ^{13}C NMR (101 MHz, $CDCl_3$) δ 170.1, 136.3, 135.1, 133.6, 129.3, 128.7, 127.2, 126.6 (2 C), 126.2, 126.1, 122.2, 119.7, 118.7, 111.2 (2 C), 107.9, 63.7, 48.4, 40.2, 25.4, 19.3; HRMS (ESI) calcd for $C_{24}H_{23}N_2O_2S^+$ $[M + H]^+$ 403.1475, found 403.1476.

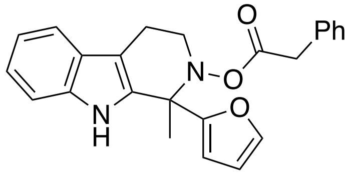

26

1-(furan-2-yl)-1-methyl-1,3,4,9-tetrahydro-2*H*-pyrido[3,4-*b*]indol-2-yl 2-phenylacetate (26). Prepared using the general procedure described above with **S23** and phenylacetyl chloride, ultimately yielding **26** (0.090 g, 93% yield) as a pale yellow solid. **26**: R_f = 0.31 (silica gel, hexanes/EtOAc = 4/1); IR (film) ν_{max} 3373, 1750, 1496, 1454, 1300, 1233, 1157, 1115, 1011, 744, 697 cm^{-1}; ^{1}H NMR (500 MHz, $CDCl_3$) δ 7.69 (br s, 1 H, exchangeable), 7.52 (d, *J* = 7.7 Hz, 1 H), 7.41–7.34 (m, 1 H), 7.33–7.25 (m, 4 H), 7.25–7.21 (m, 2 H), 7.21–7.16 (m, 1 H), 7.16–7.10 (m, 1 H), 6.30–6.20 (m, 1 H), 5.97 (d, *J* = 3.3 Hz, 1 H), 3.60–3.52 (m, 2 H), 3.51–3.41 (m, 2 H), 3.04–2.94 (m, 1 H), 2.93–2.86 (m, 1 H), 1.81 (s, 3 H); ^{13}C NMR (101 MHz, $CDCl_3$) δ 170.1, 154.4, 142.7, 136.3, 133.8, 133.6, 129.3, 128.6, 127.2, 126.6, 122.2, 119.6, 118.6, 111.2, 110.1, 109.7, 108.2, 62.3, 48.7, 40.2, 22.4, 19.2; HRMS (CI) calcd for $C_{24}H_{23}N_2O_3^+$ $[M + H]^+$ 387.1703, found 387.1703.

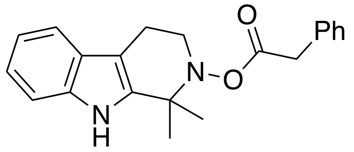

27

1,1-dimethyl-1,3,4,9-tetrahydro-2*H*-pyrido[3,4-*b*]indol-2-yl 2-phenylacetate (27). Prepared using the general procedure described above with **S24** and phenylacetyl chloride, ultimately yielding **27** (0.081 g, 97% yield) as a white solid. **27**: R_f = 0.50 (silica gel, hexanes/EtOAc = 2/1); IR (film) ν_{max} 3364, 2981, 1746, 1598, 1454, 1317, 1299, 1231, 1120, 744, 696 cm^{-1}; ^{1}H NMR (500 MHz, $CDCl_3$) δ 7.68 (br s, 1 H, exchangeable), 7.48 (d, *J* = 7.7 Hz, 1 H), 7.33 (d, *J* = 8.0 Hz, 1 H), 7.30–7.22 (m, 5 H), 7.20–7.15 (m, 1 H), 7.15–7.08 (m, 1 H), 3.61 (s, 2 H), 3.52 (t, *J* = 5.6 Hz, 2 H), 2.83 (t, *J* = 5.0 Hz, 2 H), 1.46 (s, 6 H); ^{13}C NMR (126 MHz, $CDCl_3$, 45 ºC) δ 170.3, 137.6, 136.2, 133.8, 129.3, 128.7, 127.3, 127.2, 121.9, 119.7, 118.4, 111.0, 106.3, 59.8, 48.3, 40.4, 26.2, 18.8; HRMS (ESI) calcd for $C_{21}H_{23}N_2O_2^+$ $[M + H]^+$ 335.1754, found 335.1756.

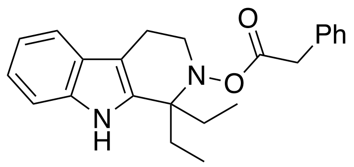

28

1,1-diethyl-1,3,4,9-tetrahydro-2*H*-pyrido[3,4-*b*]indol-2-yl 2-phenylacetate (28). Prepared using the general procedure described above with **S25** and phenylacetyl chloride, ultimately yielding **28** (0.077 g, 85% yield) as a yellow solid. **28**: R_f = 0.42 (silica gel, hexanes/EtOAc = 4/1); IR (film) ν_{max} 3387, 2972, 1746, 1597, 1496, 1454, 1339, 1231, 1119, 744 cm^{-1}; ^{1}H NMR (500 MHz, $CDCl_3$) δ 7.65 (br s, 1 H, exchangeable), 7.49 (d, *J* = 7.8 Hz, 1 H), 7.33 (d, *J* = 8.0 Hz, 1

H), 7.31–7.21 (m, 5 H), 7.20–7.15 (m, 1 H), 7.13–7.09 (m, 1 H), 3.62–3.52 (m, 4 H), 2.83 (t, *J* = 6.1 Hz, 2 H), 1.86–1.70 (m, 4 H), 0.85 (t, *J* = 7.5 Hz, 6 H); ^{13}C NMR (101 MHz, $CDCl_3$) δ 170.0, 136.1, 135.9, 133.8, 129.2, 128.7, 127.3, 126.9, 121.7, 119.5, 118.3, 110.9, 107.6, 65.6, 47.5, 40.6, 28.0, 18.9, 8.7; HRMS (ESI) calcd for $C_{23}H_{27}N_2O_2^+$ [M + H]$^+$ 363.2067, found 363.2072.

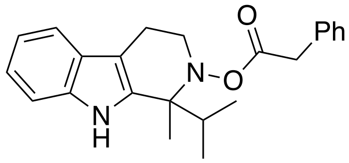

29

1-isopropyl-1-methyl-1,3,4,9-tetrahydro-2*H*-pyrido[3,4-*b*]indol-2-yl 2-phenylacetate (29). Prepared using the general procedure described above with **S26** and phenylacetyl chloride, ultimately yielding **29** (0.067 g, 74% yield) as a yellow solid. **29**: R_f = 0.40 (silica gel, hexanes/EtOAc = 4/1); IR (film) ν_{max} 3388, 2965, 1744, 1597, 1454, 1389, 1297, 1229, 1122, 744, 696 cm^{-1}; ^{1}H NMR (500 MHz, $CDCl_3$) δ 7.66 (br s, 1 H, exchangeable), 7.49 (d, *J* = 7.7 Hz, 1 H), 7.33 (d, *J* = 8.0 Hz, 1 H), 7.31–7.21 (m, 5 H), 7.20–7.15 (m, 1 H), 7.14–7.09 (m, 1 H), 3.58–3.50 (m, 3 H), 3.50–3.44 (m, 1 H), 2.90–2.81 (m, 1 H), 2.81–2.74 (m, 1 H), 2.03–1.94 (m, 1 H), 1.33 (s, 3 H), 1.06 (d, *J* = 6.9 Hz, 3 H), 0.95 (d, *J* = 6.8 Hz, 3 H); ^{13}C NMR (101 MHz, $CDCl_3$) δ 170.2, 137.3, 135.9, 133.8, 129.3, 128.6, 127.2, 126.8, 121.8, 119.5, 118.4, 110.8, 107.4, 65.2, 47.7, 40.4, 37.2, 18.6, 17.9, 17.4; HRMS (CI) calcd for $C_{23}H_{27}N_2O_2^+$ [M + H]$^+$ 363.2067, found 363.2066.

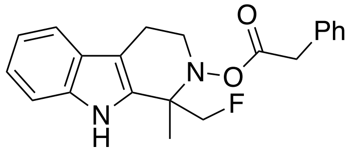

30

1-(fluoromethyl)-1-methyl-1,3,4,9-tetrahydro-2*H*-pyrido[3,4-*b*]indol-2-yl 2-phenylacetate (30). Prepared using the general procedure described above with **S27** and phenylacetyl chloride, ultimately yielding **30** (0.063 g, 71% yield) as a yellow solid. **30**: R_f = 0.26 (silica gel, hexanes/EtOAc = 4/1); IR (film) ν_{max} 3372, 1750, 1598, 1454, 1301, 1230, 1117, 1030, 745, 697 cm^{-1}; ^{1}H NMR (500 MHz, $CDCl_3$) δ 8.02 (br s, 1 H, exchangeable), 7.50 (d, *J* = 7.8 Hz, 1 H), 7.37–7.23 (m, 6 H), 7.23–7.16 (m, 1 H), 7.15–7.08 (m, 1 H), 4.48 (ddd, *J* = 62.2, 47.0, 8.5 Hz, 2 H), 3.61 (s, 2 H), 3.55–3.46 (m, 2 H), 3.00–2.88 (m, 1 H), 2.87–2.76 (m, 1 H), 1.54 (s, 3 H); ^{13}C NMR (126 MHz, $CDCl_3$, 45 ºC) δ 169.8, 136.5, 134.2, 133.5, 129.3, 128.9, 127.5, 126.4, 122.4, 119.8, 118.6, 111.2, 107.5, 87.97 (d, *J* = 172.9 Hz), 62.22 (d, *J* = 19.4 Hz), 48.9, 40.4, 20.0, 19.1; HRMS (CI) calcd for $C_{21}H_{22}FN_2O_2^+$ [M + H]$^+$ 353.1660, found 353.1660.

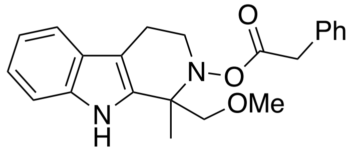

31

1-(methoxymethyl)-1-methyl-1,3,4,9-tetrahydro-2*H*-pyrido[3,4-*b*]indol-2-yl 2-phenylacetate (31). Prepared using the general procedure described above with **S28** and phenylacetyl chloride, ultimately yielding **31** (0.080 g, 87% yield) as a yellow solid. **31**: R_f = 0.53 (silica gel, hexanes/EtOAc = 2/1); IR (film) ν_{max} 3437, 2922, 1754, 1598, 1454, 1318, 1301, 1230, 1109, 745, 696 cm^{-1}; ^{1}H NMR (500 MHz, $CDCl_3$) δ 8.47 (br s, 1 H, exchangeable), 7.48 (d, *J* = 7.7 Hz, 1 H), 7.38–7.26 (m, 6 H), 7.19–7.13 (m, 1 H), 7.12–7.06 (m, 1 H), 3.61 (s, 2 H), 3.55–3.45 (m, 3 H), 3.33 (s, 4 H), 3.02–2.91 (m, 1 H), 2.81–2.72 (m, 1 H), 1.50 (s, 3 H); ^{13}C NMR (126 MHz, $CDCl_3$, 45 ºC) δ 170.0, 136.6, 136.2, 133.8, 129.4, 128.8, 127.4, 126.5, 121.8, 119.4, 118.4, 111.1, 106.1, 78.4, 62.4, 59.7, 48.7, 40.6, 21.0, 19.2; HRMS (CI) calcd for $C_{22}H_{25}N_2O_3^+$ [M + H]$^+$ 365.1860, found 365.1862.

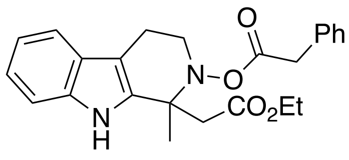

32

1-(2-ethoxy-2-oxoethyl)-1-methyl-1,3,4,9-tetrahydro-2*H*-pyrido[3,4-*b*]indol-2-yl 2-phenylacetate (32). Prepared using the general procedure described above with **S29** and phenylacetyl chloride, ultimately yielding **32** (0.097 g, 96% yield) as a white solid. **32**: R_f = 0.29 (silica gel, hexanes/EtOAc = 4/1); IR (film) ν_{max} 3364, 2903, 2383, 2315, 1760, 1597, 1454, 1226, 1117, 1031 cm^{-1}; ^{1}H NMR (500 MHz, $CDCl_3$) δ 9.46 (br s, 1 H, exchangeable), 7.47 (d, *J* = 7.8 Hz, 1 H), 7.36 (d, *J* = 8.1 Hz, 1 H), 7.33–7.24 (m, 5 H), 7.21–7.14 (m, 1 H), 7.13–7.05 (m, 1 H), 4.27–4.11 (m, 2 H), 3.62 (s, 2 H), 3.55–3.45 (m, 2 H), 2.96–2.85 (m, 1 H), 2.84–2.74 (m, 3 H), 1.57 (s, 3 H), 1.28 (t, *J* = 7.1 Hz, 3 H); ^{13}C NMR (126 MHz, $CDCl_3$, 45 ºC) δ 173.2, 169.9, 136.2, 136.0, 133.7, 129.3, 128.8, 127.4, 126.5, 122.0, 119.5, 118.4, 111.4, 106.6, 61.3, 61.2, 48.3, 43.3, 40.5, 23.0, 19.0, 14.2; HRMS (CI) calcd for $C_{24}H_{27}N_2O_4^+$ $[M + H]^+$ 407.1965, found 407.1963.

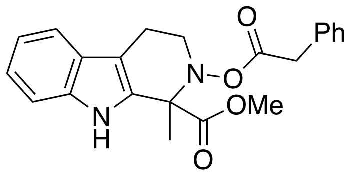

33

Methyl 1-methyl-2-(2-phenylacetoxy)-2,3,4,9-tetrahydro-1*H*-pyrido[3,4-*b*]indole-1-carboxylate (33). Prepared using the general procedure described above with **S30** and phenylacetyl chloride, ultimately yielding **33** (0.087 g, 92% yield) as a yellow solid. **33**: R_f = 0.38 (silica gel, hexanes/EtOAc = 2/1); IR (film) ν_{max} 3365, 2383, 2315, 1764, 1598, 1454, 1232, 1119, 1073, 736 cm^{-1}; ^{1}H NMR (500 MHz, $CDCl_3$) δ 8.50 (br s, 1 H, exchangeable), 7.50 (d, *J* = 7.8 Hz, 1 H), 7.37 (d, *J* = 8.1 Hz, 1 H), 7.34–7.23 (m, 5 H), 7.23–7.17 (m, 1 H), 7.15–7.09 (m, 1 H), 3.87–3.75 (m, 1 H), 3.59–3.47 (m, 3 H), 3.45 (s, 3 H), 3.03–2.94 (m, 1 H), 2.87–2.79 (m, 1 H), 1.74 (s, 3 H); ^{13}C NMR (126 MHz, $CDCl_3$, 45 ºC) δ 172.5, 169.4, 136.6, 133.6, 131.7, 129.3, 128.7, 127.3, 126.6, 122.5, 119.8, 118.6, 111.3, 108.1, 67.1, 52.6, 48.5, 40.3, 24.3, 18.7; HRMS (CI) calcd for $C_{22}H_{23}N_2O_4^+$ $[M + H]^+$ 379.1652, found 379.1651.

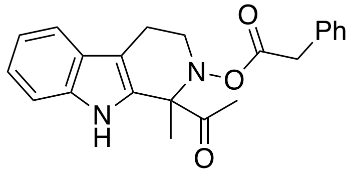

34

1-acetyl-1-methyl-1,3,4,9-tetrahydro-2*H*-pyrido[3,4-*b*]indol-2-yl 2-phenylacetate (34). Prepared using the general procedure described above with **S31** and phenylacetyl chloride, ultimately yielding **34** (0.072 g, 80% yield) as a white solid. **34**: R_f = 0.34 (silica gel, hexanes/EtOAc = 4/1); IR (film) ν_{max} 3379, 1757, 1713, 1597, 1454, 1352, 1300, 1231, 1112, 745, 695 cm^{-1}; ^{1}H NMR (500 MHz, $CDCl_3$) δ 8.36 (br s, 1 H, exchangeable), 7.50 (d, *J* = 7.8 Hz, 1 H), 7.36 (d, *J* = 8.1 Hz, 1 H), 7.33–7.16 (m, 6 H), 7.15–7.09 (m, 1 H), 3.76–3.66 (m, 1 H), 3.55 (s, 2 H), 3.49–3.41 (m, 1 H), 3.05–2.93 (m, 1 H), 2.83–2.73 (m, 1 H), 2.09 (s, 3 H), 1.60 (s, 3 H); ^{13}C NMR (101 MHz, $CDCl_3$) δ 209.9, 169.4, 136.6, 133.1, 132.1, 129.2, 128.8, 127.6, 126.4, 122.4, 119.7, 118.5, 111.4, 107.8, 71.7, 48.2, 40.3, 24.6, 21.1, 19.0; HRMS (CI) calcd for $C_{22}H_{23}N_2O_3^+$ $[M + H]^+$ 363.1703, found 363.1707.

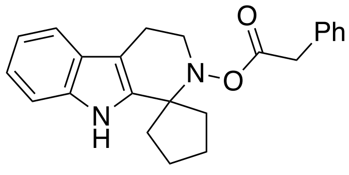

35

4',9'-dihydrospiro[cyclopentane-1,1'-pyrido[3,4-*b*]indol]-2'(3'*H*)-yl 2-phenylacetate (35). Prepared using the general procedure described above with **S32** and phenylacetyl chloride, ultimately yielding **35** (0.086 g, 95% yield) as a white solid. **35**: R_f = 0.31 (silica gel, hexanes/EtOAc = 4/1); IR (film) ν_{max} 3376, 2958, 1745, 1598, 1454, 1299, 1230, 1117, 744, 696 cm^{-1}; ^{1}H NMR (500 MHz, $CDCl_3$) δ 7.66 (br s, 1 H, exchangeable), 7.49 (d, *J* = 7.7 Hz, 1 H), 7.34 (d, *J* = 7.9 Hz, 1

H), 7.29–7.21 (m, 5 H), 7.21–7.15 (m, 1 H), 7.15–7.10 (m, 1 H), 3.74–3.60 (m, 1 H), 3.56 (s, 2 H), 3.51–3.36 (m, 1 H), 2.94–2.56 (m, 2 H), 2.12–2.05 (m, 2 H), 1.96–1.84 (m, 4 H), 1.82–1.73 (m, 2 H); ^{13}C NMR (101 MHz, $CDCl_3$) δ 170.4, 137.3, 135.9, 133.8, 129.2, 128.7, 127.3, 127.1, 121.8, 119.7, 118.3, 110.9, 106.6, 70.5, 49.1, 41.0, 40.4, 36.1, 25.7, 25.6, 17.7; HRMS (ESI) calcd for $C_{23}H_{25}N_2O_2^+$ $[M + H]^+$ 361.1911, found 361.1913.

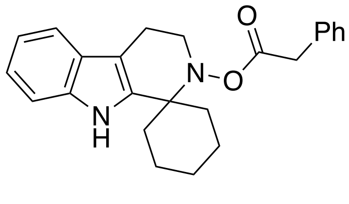

36

4',9'-dihydrospiro[cyclohexane-1,1'-pyrido[3,4-*b*]indol]-2'(3'*H*)-yl 2-phenylacetate (36). Prepared using the general procedure described above with **S33** and phenylacetyl chloride, ultimately yielding **36** (0.080 g, 86% yield) as a white solid. **36**: R_f = 0.41 (silica gel, hexanes/EtOAc = 4/1); IR (film) ν_{max} 3379, 2938, 2854, 1738, 1598, 1446, 1297, 1271, 1230, 1118, 744, 695 cm^{-1}; ^{1}H NMR (500 MHz, $CDCl_3$) δ 7.75 (br s, 1 H, exchangeable), 7.50 (d, *J* = 7.7 Hz, 1 H), 7.35 (d, *J* = 8.0 Hz, 1 H), 7.27–7.20 (m, 5 H), 7.20–7.16 (m, 1 H), 7.15–7.11 (m, 1 H), 3.80–3.66 (m, 1 H), 3.57–3.46 (m, 3 H), 2.98–2.77 (m, 1 H), 2.70–2.50 (m, 1 H), 2.02–1.88 (m, 3 H), 1.74–1.62 (m, 4 H), 1.50–1.42 (m, 1 H), 1.40–1.27 (m, 2 H); ^{13}C NMR (101 MHz, $CDCl_3$) δ 170.2, 138.0, 135.6, 133.8, 129.2, 128.6, 127.2 (2 C), 121.7, 119.6, 118.3, 111.0, 106.6, 60.9, 46.7, 40.5, 38.7, 33.2, 25.9, 21.5, 21.1, 17.2; HRMS (ESI) calcd for $C_{24}H_{27}N_2O_2^+$ $[M + H]^+$ 375.2067, found 375.2068.

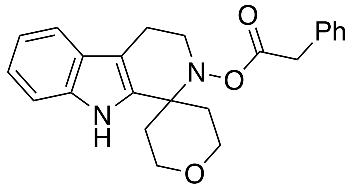

37

2,3,4',5,6,9'-hexahydrospiro[pyran-4,1'-pyrido[3,4-*b*]indol]-2'(3'*H*)-yl 2-phenylacetate (37). Prepared using the general procedure described above with **S34** and phenylacetyl chloride, ultimately yielding **37** (0.089 g, 94% yield) as a yellow solid. **37**: R_f = 0.29 (silica gel, hexanes/EtOAc = 2/1); IR (film) ν_{max} 3295, 2956, 1748, 1597, 1453, 1305, 1264, 1229, 1105, 744 cm^{-1}; ^{1}H NMR (500 MHz, $CDCl_3$) δ 7.92 (br s, 1 H, exchangeable), 7.51 (d, *J* = 7.7 Hz, 1 H), 7.36 (d, *J* = 8.0 Hz, 1 H), 7.29–7.18 (m, 6 H), 7.17–7.11 (m, 1 H), 4.19–4.00 (m, 1 H), 3.81–3.64 (m, 3 H), 3.61–3.42 (m, 4 H), 3.03–2.79 (m, 1 H), 2.75–2.51 (m, 1 H), 2.05–1.95 (m, 2 H), 1.86–1.77 (m, 2 H); ^{13}C NMR (101 MHz, $CDCl_3$) δ 169.9, 136.1, 136.0, 133.5, 129.1, 128.8, 127.4, 126.9, 122.0, 119.6, 118.4, 111.3, 107.2, 63.3, 63.1, 59.0, 47.2, 40.5, 38.0, 32.9, 17.1; HRMS (ESI) calcd for $C_{23}H_{25}N_2O_3^+$ $[M + H]^+$ 377.1860, found 377.1865.

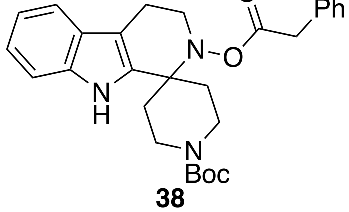

38

***tert*-butyl 2'-(2-phenylacetoxy)-2',3',4',9'-tetrahydrospiro[piperidine-4,1'-pyrido[3,4-*b*]indole]-1-carboxylate (38).** Prepared using the general procedure described above with **S35** and phenylacetyl chloride, ultimately yielding **38** (0.109 g, 91% yield) as a white solid. **38**: R_f = 0.31 (silica gel, hexanes/EtOAc = 4/1); IR (film) ν_{max} 3304, 2975, 1755, 1670, 1598, 1435, 1366, 1247, 1163, 1113, 744 cm^{-1}; ^{1}H NMR (500 MHz, $CDCl_3$) δ 8.36 (br s, 1 H, exchangeable), 7.51 (d, *J* = 7.7 Hz, 1 H), 7.36 (d, *J* = 8.0 Hz, 1 H), 7.25–7.10 (m, 7 H), 3.97–3.62 (m, 3 H), 3.58–3.32 (m, 4 H), 3.13–2.78 (m, 2 H), 2.77–2.54 (m, 1 H), 1.92–1.78 (m, 4 H), 1.48 (s, 9 H); ^{13}C NMR (101 MHz, $CDCl_3$) δ 169.9, 154.7, 136.2, 136.1, 133.5, 129.1, 128.7, 127.4, 126.7, 121.8, 119.4, 118.2, 111.5, 106.7, 79.9, 59.7, 47.2, 40.6, 40.0, 38.7, 37.4, 32.5, 28.7, 17.2; HRMS (CI) calcd for $C_{28}H_{34}N_3O_4^+$ $[M + H]^+$ 476.2544, found 476.2543.

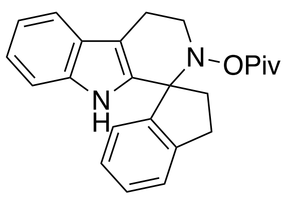

39

2,3,4',9'-tetrahydrospiro[indene-1,1'-pyrido[3,4-*b*]indol]-2'(3'*H*)-yl pivalate (39). Prepared using the general procedure described above with **S36** and pivaloyl chloride, ultimately yielding **39** (0.085 g, 90% yield) as a brown solid. **39**: R_f = 0.57 (silica gel, hexanes/EtOAc = 4/1); IR (film) ν_{max} 3388, 2970, 1739, 1597, 1478, 1460, 1396, 1303, 1274, 1125, 744 cm^{-1}; ^{1}H NMR (500 MHz, CDCl$_3$, 45 ºC) δ 7.58–7.53 (m, 1 H), 7.40 (br s, 1 H, exchangeable), 7.34–7.27 (m, 2 H), 7.23–7.18 (m, 1 H), 7.17–7.08 (m, 4 H), 3.74–3.64 (m, 1 H), 3.58–3.50 (m, 1 H), 3.40–3.29 (m, 1 H), 3.25–3.16 (m, 1 H), 3.08–2.99 (m, 1 H), 2.96–2.89 (m, 1 H), 2.85–2.73 (m, 1 H), 2.47–2.36 (m, 1 H), 1.00 (s, 9 H); ^{13}C NMR (126 MHz, CDCl$_3$, 45 ºC) δ 176.0, 145.6, 142.9, 136.5, 136.2, 129.0, 127.0, 126.9, 126.5, 124.7, 122.0, 119.7, 118.5, 111.1, 108.5, 74.6, 49.7, 38.6, 30.8, 27.2, 27.1, 19.4; HRMS (CI) calcd for $C_{24}H_{27}N_2O_2^+$ [M + H]$^+$ 375.2067, found 375.2066.

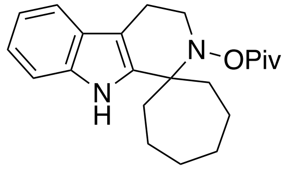

40

4',9'-dihydrospiro[cycloheptane-1,1'-pyrido[3,4-*b*]indol]-2'(3'*H*)-yl pivalate (49). Prepared using the general procedure described above with **S37** and pivaloyl chloride, ultimately yielding **40** (0.064 g, 72% yield) as a white solid. **40**: R_f = 0.54 (silica gel, hexanes/EtOAc = 4/1); IR (film) ν_{max} 3379, 2928, 1728, 1597, 1564, 1448, 1395, 1277, 1127, 744 cm^{-1}; ^{1}H NMR (500 MHz, CDCl$_3$, 45 ºC) δ 7.85 (br s, 1 H, exchangeable), 7.51 (d, *J* = 7.7 Hz, 1 H), 7.36 (d, *J* = 8.0 Hz, 1 H), 7.21–7.16 (m, 1 H), 7.15–7.11 (m, 1 H), 3.69–3.56 (m, 2 H), 2.94–2.72 (m, 2 H), 2.21–2.10 (m, 2 H), 1.98–1.88 (m, 4 H), 1.80–1.74 (m, 2 H), 1.69–1.57 (m, 4 H), 1.18 (s, 9 H); ^{13}C NMR (126 MHz, CDCl$_3$, 45 ºC) δ 176.4, 139.6, 135.9, 127.4, 121.6, 119.6, 118.4, 110.9, 105.6, 64.7, 47.1, 39.1, 30.0, 27.4, 27.3, 23.1, 17.6; HRMS (CI) calcd for $C_{22}H_{31}N_2O_2^+$ [M + H]$^+$ 355.2380, found 355.2378.

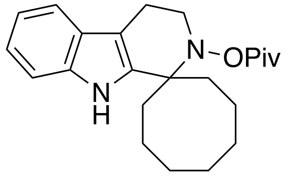

41

4',9'-dihydrospiro[cyclooctane-1,1'-pyrido[3,4-*b*]indol]-2'(3'*H*)-yl pivalate (41). Prepared using the general procedure described above with **S38** and pivaloyl chloride, ultimately yielding **41** (0.050 g, 54% yield) as a white solid. **41**: R_f = 0.58 (silica gel, hexanes/EtOAc = 4/1); IR (film) ν_{max} 3378, 2925, 1728, 1597, 1564, 1448, 1396, 1278, 1127, 1028, 743 cm^{-1}; ^{1}H NMR (500 MHz, CDCl$_3$, 45 ºC) δ 7.81 (br s, 1 H, exchangeable), 7.51 (d, *J* = 7.7 Hz, 1 H), 7.36 (d, *J* = 8.0 Hz, 1 H), 7.21–7.15 (m, 1 H), 7.14–7.09 (m, 1 H), 3.73–3.54 (m, 2 H), 2.99–2.65 (m, 2 H), 2.20–2.08 (m, 2 H), 2.05–1.95 (m, 4 H), 1.72–1.55 (m, 8 H), 1.15 (s, 9 H); ^{13}C NMR (126 MHz, CDCl$_3$, 45 ºC) δ 176.3, 139.4, 135.8, 127.4, 121.6, 119.6, 118.4, 110.9, 105.5, 64.2, 46.9, 39.1, 28.4, 27.4, 27.3, 25.3, 22.0, 17.6; HRMS (ESI) calcd for $C_{23}H_{33}N_2O_2^+$ [M + H]$^+$ 369.2537, found 369.2537.

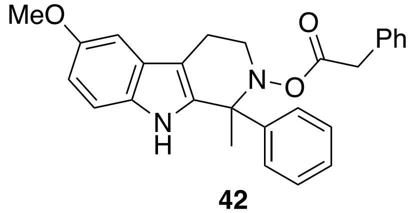

42

6-methoxy-1-methyl-1-phenyl-1,3,4,9-tetrahydro-2*H*-pyrido[3,4-*b*]indol-2-yl 2-phenylacetate (42). Prepared using the general procedure described above with **S39** and phenylacetyl chloride, ultimately yielding **42** (0.098 g, 92% yield) as a brown solid. **42**: R_f = 0.37 (silica gel, hexanes/EtOAc = 4/1); IR (film) ν_{max} 3372, 1746, 1597, 1564, 1484, 1453, 1217, 1165, 1116, 1027, 699 cm^{-1}; ^{1}H NMR (500 MHz, CDCl$_3$) δ 7.62 (br s, 1 H,

exchangeable), 7.33–7.20 (m, 9 H), 7.20–7.14 (m, 2 H), 7.01 (d, J = 2.5 Hz, 1 H), 6.87 (dd, J = 8.7, 2.5 Hz, 1 H), 3.89 (s, 3 H), 3.53 (s, 2 H), 3.48–3.39 (m, 1 H), 3.25–3.14 (m, 1 H), 3.00–2.89 (m, 1 H), 2.78–2.67 (m, 1 H), 1.77 (s, 3 H); ^{13}C NMR (101 MHz, $CDCl_3$) δ 170.5, 154.2, 136.1, 133.7, 131.2, 129.2, 128.6, 128.2 (2 C), 127.9, 127.7 (2 C), 127.3, 127.2, 111.9, 108.0, 100.7, 65.7, 56.0, 47.9, 40.2, 24.3, 18.4; HRMS (ESI) calcd for $C_{27}H_{27}N_2O_3^+$ [M + H]$^+$ 427.2016, found 427.2015.

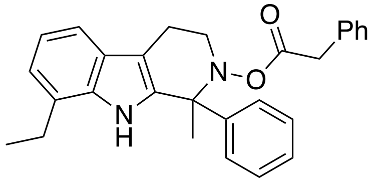

43

8-ethyl-1-methyl-1-phenyl-1,3,4,9-tetrahydro-2*H*-pyrido[3,4-*b*]indol-2-yl 2-phenylacetate (43). Prepared using the general procedure described above with **S40** and phenylacetyl chloride, ultimately yielding **43** (0.096 g, 90% yield) as a yellow solid. **43**: R_f = 0.50 (silica gel, hexanes/EtOAc = 4/1); IR (film) ν_{max} 3382, 2965, 1742, 1597, 1564, 1494, 1446, 1393, 1236, 1116, 700 cm^{-1}; ^{1}H NMR (500 MHz, $CDCl_3$) δ 7.60 (br s, 1 H, exchangeable), 7.43 (d, J = 7.8 Hz, 1 H), 7.32–7.22 (m, 8 H), 7.21–7.17 (m, 2 H), 7.17–7.12 (m, 1 H), 7.11–7.05 (m, 1 H), 3.53 (s, 2 H), 3.47–3.40 (m, 1 H), 3.24–3.14 (m, 1 H), 3.03–2.93 (m, 1 H), 2.85 (q, J = 7.6 Hz, 2 H), 2.79–2.68 (m, 1 H), 1.81 (s, 3 H), 1.36 (t, J = 7.6 Hz, 3 H); ^{13}C NMR (101 MHz, $CDCl_3$) δ 170.4, 135.0, 134.7, 133.7, 129.2, 128.6, 128.3, 128.0, 127.8 (2 C), 127.2, 126.7, 126.5, 120.7, 120.1, 116.4, 109.0, 65.7, 47.9, 40.2, 24.5, 24.0, 18.4, 13.9; HRMS (CI) calcd for $C_{28}H_{29}N_2O_2^+$ [M + H]$^+$ 425.2224, found 425.2225.

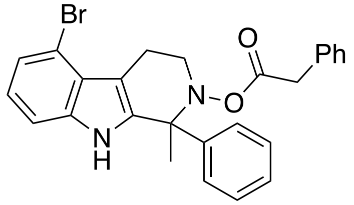

44

5-bromo-1-methyl-1-phenyl-1,3,4,9-tetrahydro-2*H*-pyrido[3,4-*b*]indol-2-yl 2-phenylacetate (44). Prepared using the general procedure described above with **S41** and phenylacetyl chloride, ultimately yielding **44** (0.099 g, 83% yield) as a white solid. **44**: R_f = 0.44 (silica gel, hexanes/EtOAc = 4/1); IR (film) ν_{max} 3357, 1743, 1597, 1494, 1446, 1317, 1237, 1118, 729, 700 cm^{-1}; ^{1}H NMR (500 MHz, $CDCl_3$) δ 7.85 (br s, 1 H, exchangeable), 7.32–7.20 (m, 10 H), 7.20–7.14 (m, 2 H), 7.01 (t, J = 7.9 Hz, 1 H), 3.54 (s, 2 H), 3.47–3.39 (m, 1 H), 3.34–3.24 (m, 1 H), 3.23–3.11 (m, 2 H), 1.77 (s, 3 H); ^{13}C NMR (101 MHz, $CDCl_3$) δ 170.4, 137.1, 136.3, 133.6, 129.3, 128.7, 128.4, 127.9, 127.7, 127.6, 127.3, 125.9, 123.7, 122.9, 114.3, 110.3, 109.2, 65.6, 47.9, 40.3, 24.4, 20.5; HRMS (ESI) calcd for $C_{26}H_{24}BrN_2O_2^+$ [M + H]$^+$ 475.1016, found 475.1017.

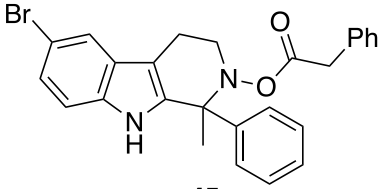

45

6-bromo-1-methyl-1-phenyl-1,3,4,9-tetrahydro-2*H*-pyrido[3,4-*b*]indol-2-yl 2-phenylacetate (45). Prepared using the general procedure described above with **S42** and phenylacetyl chloride, ultimately yielding **45** (0.089 g, 74% yield) as a white solid. **45**: R_f = 0.44 (silica gel, hexanes/EtOAc = 2/1); IR (film) ν_{max} 3362, 1743, 1597, 1447, 1301, 1220, 1117, 858, 798, 700 cm^{-1}; ^{1}H NMR (500 MHz, $CDCl_3$) δ 7.76 (br s, 1 H, exchangeable), 7.67 (d, J = 1.9 Hz, 1 H), 7.31–7.27 (m, 1 H), 7.26–7.19 (m, 9 H), 7.18–7.12 (m, 2 H), 3.53 (s, 2 H), 3.47–3.38 (m, 1 H), 3.24–3.12 (m, 1 H), 2.97–2.86 (m, 1 H), 2.78–2.65 (m, 1 H), 1.76 (s, 3 H); ^{13}C NMR (101 MHz, $CDCl_3$) δ 170.3, 136.7, 134.8, 133.6, 129.2, 128.7, 128.7, 128.4, 128.0, 127.7, 127.6, 127.3, 124.9, 121.3, 113.0, 112.6, 108.0, 65.6, 47.7, 40.3, 24.3, 18.3; HRMS (ESI) calcd for $C_{26}H_{24}BrN_2O_2^+$ [M + H]$^+$ 475.1016, found 475.1014.

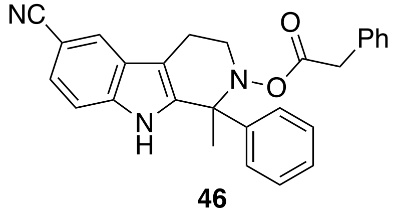

46

6-cyano-1-methyl-1-phenyl-1,3,4,9-tetrahydro-2*H*-pyrido[3,4-*b*]indol-2-yl 2-phenylacetate (46). Prepared using the general procedure described above with **S43** and phenylacetyl chloride, ultimately yielding **46** (0.069 g, 65% yield) as a white solid. **46**: R_f = 0.44 (silica gel, hexanes/EtOAc = 2/1); IR (film) ν_{max} 3328, 2219, 1747, 1598, 1473, 1446, 1319, 1232, 1184, 1116, 699 cm^{-1}; ^{1}H NMR (500 MHz, $CDCl_3$) δ 8.20 (br s, 1 H, exchangeable), 7.92–7.84 (m, 1 H), 7.46–7.35 (m, 2 H), 7.31–7.17 (m, 8 H), 7.17–7.11 (m, 2 H), 3.53 (s, 2 H), 3.48–3.40 (m, 1 H), 3.25–3.15 (m, 1 H), 2.98–2.89 (m, 1 H), 2.81–2.68 (m, 1 H), 1.78 (s, 3 H); ^{13}C NMR (101 MHz, $CDCl_3$) δ 170.3, 138.0, 137.8, 133.5, 129.2, 128.7, 128.4, 128.1, 127.6, 127.6, 127.3, 126.8, 125.1, 124.1, 120.9, 112.0, 109.0, 102.6, 65.6, 47.6, 40.2, 24.3, 18.1; HRMS (ESI) calcd for $C_{27}H_{24}N_3O_2^+$ $[M + H]^+$ 422.1863, found 422.1863.

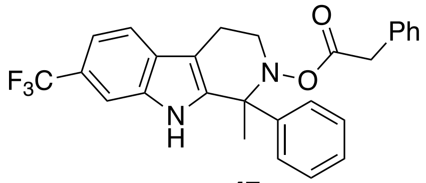

47

1-methyl-1-phenyl-7-(trifluoromethyl)-1,3,4,9-tetrahydro-2*H*-pyrido[3,4-*b*]indol-2-yl 2-phenylacetate (47). Prepared using the general procedure described above with **S44** and phenylacetyl chloride, ultimately yielding **47** (0.072 g, 62% yield) as a white solid. **47**: R_f = 0.44 (silica gel, hexanes/EtOAc = 4/1); IR (film) ν_{max} 3355, 1743, 1597, 1564, 1448, 1392, 1336, 1220, 1159, 1114, 1052, 699 cm^{-1}; ^{1}H NMR (500 MHz, $CDCl_3$) δ 7.95 (br s, 1 H, exchangeable), 7.66–7.59 (m, 2 H), 7.40 (d, *J* = 8.4 Hz, 1 H), 7.29–7.20 (m, 8 H), 7.18–7.12 (m, 2 H), 3.53 (s, 2 H), 3.49–3.42 (m, 1 H), 3.28–3.16 (m, 1 H), 3.01–2.91 (m, 1 H), 2.83–2.71 (m, 1 H), 1.79 (s, 3 H); ^{13}C NMR (101 MHz, $CDCl_3$) δ 170.4, 138.3, 135.1, 133.6, 129.2, 129.0, 128.7, 128.4, 128.3, 128.0, 127.8, 127.7, 127.3, 125.3 (q, *J* = 271.2 Hz), 124.1 (q, *J* = 31.9 Hz), 119.0, 116.6, 108.6, 65.7, 47.7, 40.3, 24.3, 18.3; HRMS (ESI) calcd for $C_{27}H_{24}F_3N_2O_2^+$ $[M + H]^+$ 465.1784, found 465.1784.

F. Identification of Hydrolysis By-product

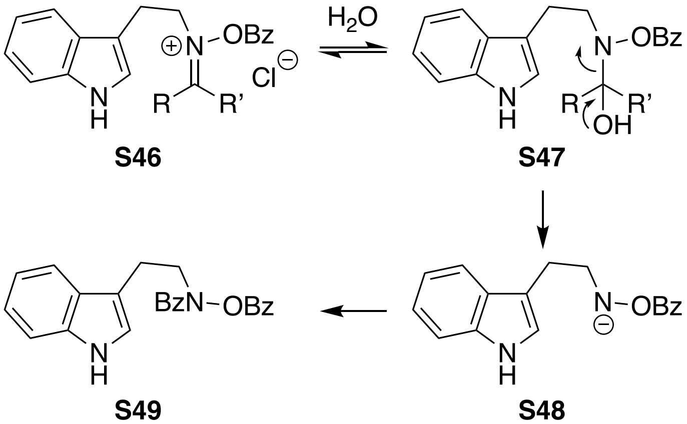

Figure S1. Proposed hydrolysis pathway.

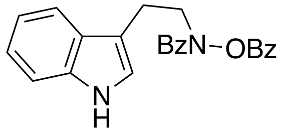

S49

***N*-(2-(1*H*-indol-3-yl)ethyl)-*N*-(benzoyloxy)benzamide (S49)**: R_f = 0.16 (silica gel, hexanes/EtOAc = 4/1); IR (film) ν_{max} 3406, 3059, 2925, 1762, 1654, 1451, 1239, 1010, 742, 704 cm^{-1}; ^{1}H NMR (400 MHz, $CDCl_3$) δ 8.16 (br s, 1 H, exchangeable), 7.93 (d, *J* = 7.5 Hz, 2 H), 7.64–7.57 (m, 1 H), 7.50–7.39 (m, 5 H), 7.37–7.30 (m, 2 H), 7.26–7.14 (m, 3 H), 7.09–7.02 (m, 2 H), 4.20 (t, *J* = 7.2 Hz, 2 H), 3.23 (t, *J* = 7.2 Hz, 2 H); ^{13}C NMR (101 MHz, $CDCl_3$) δ 170.5, 164.4, 136.4, 134.2, 133.7, 130.9, 130.0, 128.8, 128.3, 127.8, 127.4, 127.1, 122.6, 122.2, 119.6, 118.6, 112.1, 111.4, 51.2, 23.4; HRMS (ESI) calcd for $C_{24}H_{21}N_2O_3^+$ [M + H]$^+$ 385.1547, found 385.1547.

G. Catalyst Screening and Optimization

Table S1. Selected exploration of varied catalysts to achieve asymmetric cyclization of nitrone **19** using BzCl.[a]

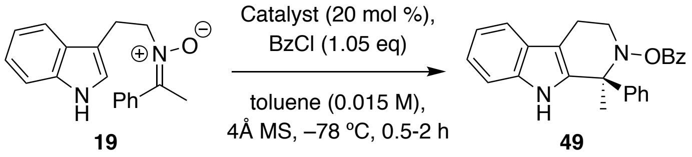

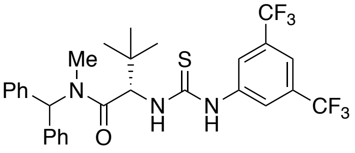

48
(CH_2Cl_2: 79%, 51% *ee*)
(Toluene: 52%, 64% *ee*)

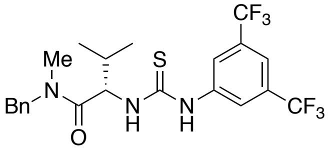

S50
(CH_2Cl_2: 77%, 36% *ee*)
(Toluene: 52%, 55% *ee*)

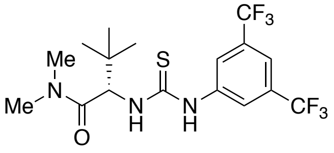

S51
(48% *ee*)

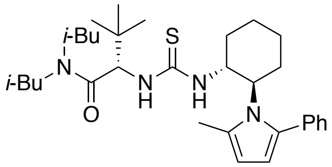

S52
(racemic)

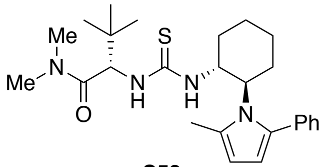

S53
(<20%, 76% *ee*)

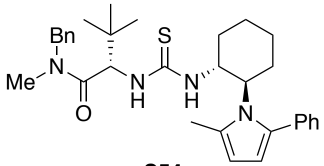

S54
(<20%, 81% *ee*)

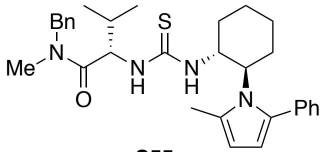

S55
(<20%, 77% *ee*)

[a] Final reactions were performed with **19** (0.09 mmol) under argon.

Note: All other acyl chlorides tested did not provide substantial improvements in enantioselectivity (4-OMeBzCl, 4-ClBzCl, 4-NO_2BzCl, 4-CF_3BzCl, 2-CF_3BzCl, 3,5-CF_3BzCl, AcCl, PivCl, methyl chloroformate, $PhCH_2COCl$, $Ph_2CHCOCl$, 9-anthracenecarbonyl chloride, 1-naphthoyl chloride), except 2-naphthoyl chloride that provided an ~10% *ee* increase with catalyst **S50**. Chiral auxiliary-based acyl chlorides were not able to induce good diastereoselectivity (~1:1). Bz_2O was unable to form the desired *N*-acyloxyiminium species, and thus no reaction occurred. All acid (HCl) and base (2,6-lutidine, 2,6-di-*tert*-butylpyridine, pyridine, 4-DMAP, *i*-Pr_2NEt, TMPDA) additives tested caused detrimental effects to the enantioselectivity of the reaction. Other solvents investigated led to no reaction (Et_2O or hexanes) or lower enantioselectivity (MTBE). Chiral phosphoric acids were not successful in promoting the desired cyclization with or without acyl chloride additive.

After exhaustive screening of catalysts under the BzCl system (only selected examples shown above in Table S1), we opted to change to BzBr based on the known effect halide counter ions can have on thiourea binding.[8,9b] We then rescreened our collection of catalysts that were purchased, donated by Merck, or synthesized according to literature procedures,[9] with key results shown in Table S2.

Table S2. Exploration of varied catalysts to achieve asymmetric cyclization of nitrone **19** using BzBr.[a]

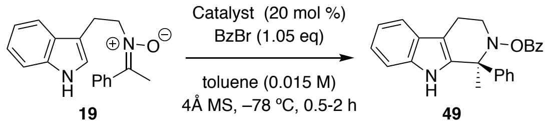

19 **49**

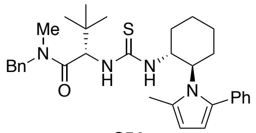

S54
(60%, 80% *ee*)

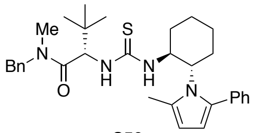

S56
(40% *ee*)

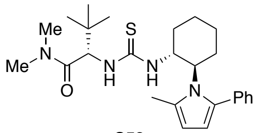

S53
(45%, 79% *ee*)

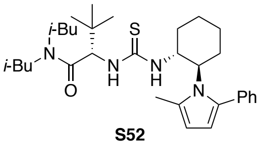

S52
(15% *ee*)

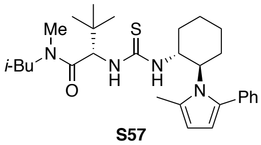

S57
(69% *ee*)

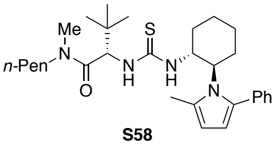

S58
(70% *ee*)

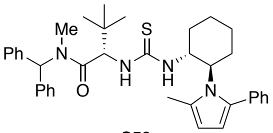

S59
(55%, 87% *ee*)[b][c]

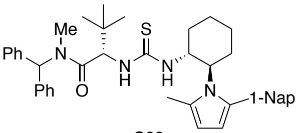

S60
(81% *ee*)[b]

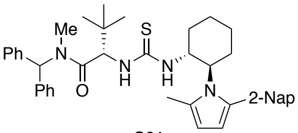

S61
(85% *ee*)[b][c]

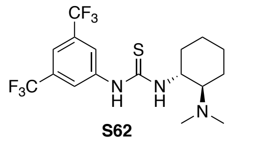

S62
(-41% *ee*)

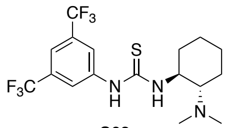

S63
(41% *ee*)

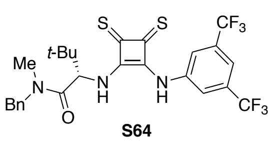

S64
(21% *ee*)

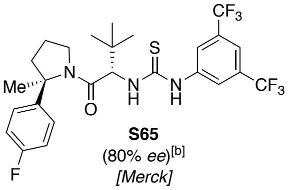

S65
(80% *ee*)[b]
[Merck]

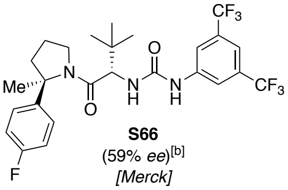

S66
(59% *ee*)[b]
[Merck]

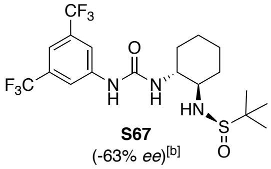

S67
(-63% *ee*)[b]
[Merck]

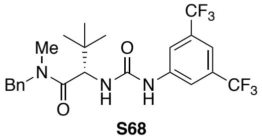

S68
(73% *ee*)

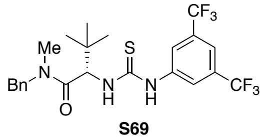

S69
(52%, 79% *ee*)

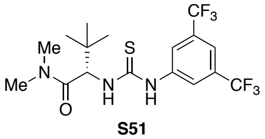

S51
(50%, 80% *ee*)

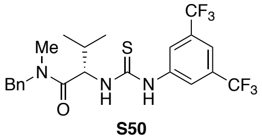

S50
(53%, 82% *ee*)

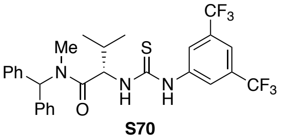

S70
(83% *ee*)

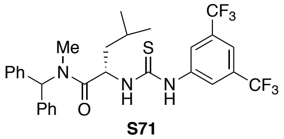

S71
(79% *ee*)

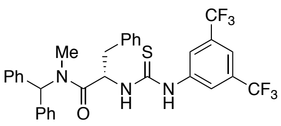

S72
(68% *ee*)

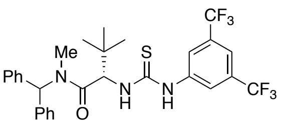

48
(75%, 83% *ee*)

[a] Final reactions were performed with **19** (0.09 mmol) under argon; [b] Catalyst (10 mol %); [c] very challenging or unsuccesful separation of catalyst and product.

Table S3. Optimization of reaction conditions with thiourea **48**.[a]

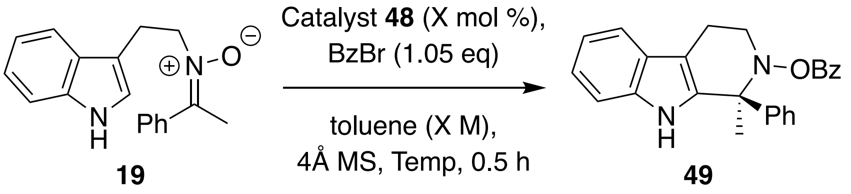

Entry	(X mol %)	M (mol/L)	Temp (ºC)	Yield [%]	*ee* [%]
1	20 mol %	0.015	–78 ºC	75	83
2	5 mol %	0.015	–78 ºC	59	77
3	10 mol %	0.015	–78 ºC	83	83
4	10 mol %	0.02	–78 ºC	83	78
5	10 mol %	0.005	–78 ºC	85	85
6	10 mol %	0.01	–78 ºC	85	84
7	10 mol %	0.01	–93 ºC	90	82

[a] Final reactions were performed with **19** (0.09 mmol) under argon.

Note: Entry 6 (Table S3) was chosen as the optimal conditions, because it provided the best results in combination with ease of reaction set-up. When the reaction was run on scale (0.25 mmol) the yield and enantioselectivity slightly decreased to 83% and 82% *ee*, respectively (see section H, below).

H. General Procedure for Enantioselective Pictet–Spengler Reactions of Nitrones.

Molecular sieves (4Å, powder 1.25 g) were flame-dried *in vacuo* and then allowed to cool to 23 °C before being suspended in toluene (25.0 mL) under an argon atmosphere. Nitrone (0.25 mmol, 1.0 equiv) and catalyst **48**[10] (0.015 g, 0.025 mmol, 0.1 equiv) were added sequentially. The reaction contents were then cooled to –78 ºC and stirred for 10 min before benzoyl bromide (0.031 mL, 0.26 mmol, 1.05 equiv) was added. The reaction mixture was then stirred for an additional 30 min at –78 ºC. Upon completion, the contents were quenched by the addition of saturated aqueous $NaHCO_3$ (25 mL), warmed to 23 ºC, poured into a separatory funnel, and extracted with EtOAc (3 × 75 mL). The combined organic extracts were then dried (Na_2SO_4), filtered, and concentrated. The resultant crude material was purified by flash column chromatography (silica gel, hexanes/Et_2O = 1/0→4/1) to yield **49**–**57**. *Note:* These reactions must be run under strictly anhydrous conditions to avoid hydrolysis of the *N*-acyloxyiminium species. In addition, the enantiomeric excess was determined by chiral HPLC (ChiralPak AD-H, 90:10 hexanes/*i*-PrOH, 1 mL/min, 254 nm).

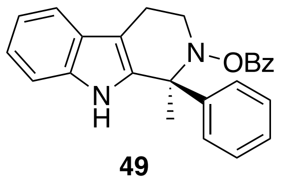

49

(*S*)-1-methyl-1-phenyl-1,3,4,9-tetrahydro-2*H*-pyrido[3,4-*b*]indol-2-yl benzoate (49). Prepared using the general procedure described above with **19**, ultimately yielding **49** (0.080 g, 83% yield, 82% *ee*) as a white solid. **49**: see above for data; $[\alpha]_D^{25}$ = –24.6º (c = 1.00, $CHCl_3$).

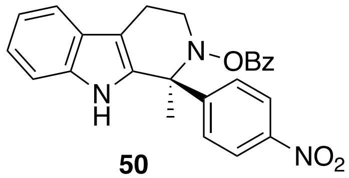

50

(*S*)-1-methyl-1-(4-nitrophenyl)-1,3,4,9-tetrahydro-2*H*-pyrido[3,4-*b*]indol-2-yl benzoate (50). Prepared using the general procedure described above with **S18**, ultimately yielding **50** (0.080 g, 75% yield, 81% *ee*) as a yellow solid. **50**: R_f = 0.38 (silica gel, hexanes/EtOAc = 4/1); $[\alpha]_D^{25}$ = –11.0º (c = 1.00, $CHCl_3$); IR (film) ν_{max} 3370, 1726, 1520, 1450, 1348, 1233, 1058, 1023, 746, 708 cm^{-1}; ^{1}H NMR (400 MHz, $CDCl_3$) δ 8.13 (d, *J* = 9.1 Hz, 2 H), 7.90 (d, *J* = 8.2 Hz, 2 H), 7.86 (br s, 1 H, exchangeable), 7.69–7.60 (m, 3 H), 7.58–7.51 (m, 1 H), 7.45–7.36 (m, 3 H), 7.31–7.26 (m, 1 H), 7.25–7.19 (m, 1 H), 3.71–3.61 (m, 1 H), 3.32–3.13 (m, 2 H), 2.95–2.82 (m, 1 H), 1.97 (s, 3 H); ^{13}C NMR (101 MHz, $CDCl_3$) δ 165.4, 147.4, 136.4, 133.7, 133.5, 129.6, 129.4, 128.9, 128.7, 126.9, 124.0, 123.5, 122.7, 120.1, 119.0, 111.4, 109.0, 65.7, 48.5, 24.8, 18.5; HRMS (ESI) calcd for $C_{25}H_{22}N_3O_4^+$ $[M + H]^+$ 428.1605, found 428.1604.

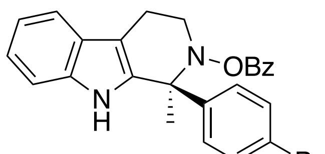

51

(*S*)-1-(4-bromophenyl)-1-methyl-1,3,4,9-tetrahydro-2*H*-pyrido[3,4-*b*]indol-2-yl benzoate (51). Prepared using the general procedure described above with **S45**, ultimately yielding **51** (0.093 g, 80% yield, 80% *ee*) as a white solid. **51:** R_f = 0.58 (silica gel, hexanes/EtOAc = 4/1); $[\alpha]_D^{25}$ = –9.6º (c = 1.00, $CHCl_3$); IR (film) ν_{max} 3361, 1725, 1485, 1450, 1271, 1177, 1059, 1009, 745, 709 cm^{-1}; ^{1}H NMR (400 MHz, $CDCl_3$) δ 7.90 (d, *J* = 8.1 Hz, 2 H), 7.80 (br s, 1 H, exchangeable), 7.61 (d, *J* = 7.8 Hz, 1 H), 7.57–7.49 (m, 1 H), 7.44–7.34 (m, 5 H), 7.31–7.27 (m, 2 H), 7.25–7.17 (m, 2 H), 3.68–3.56 (m, 1 H), 3.37–3.25 (m, 1 H), 3.21–3.11 (m, 1 H), 2.91–2.78 (m, 1 H), 1.93 (s, 3 H); ^{13}C NMR (101 MHz, $CDCl_3$) δ 165.5, 136.3, 134.6, 133.3, 131.5 (2 C), 129.6, 129.6, 129.1, 128.6, 127.0, 122.4, 122.0, 119.9, 118.9, 111.3, 108.7, 65.5, 48.2, 24.9, 18.5; HRMS (ESI) calcd for $C_{25}H_{22}BrN_2O_2^+$ $[M + H]^+$ 461.0859, found 461.0855.

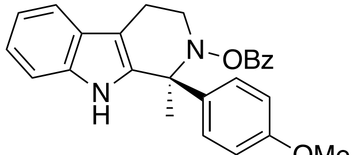

52

(*S*)-1-(4-methoxyphenyl)-1-methyl-1,3,4,9-tetrahydro-2*H*-pyrido[3,4-*b*]indol-2-yl benzoate (52). Prepared using the general procedure described above with **S19**, ultimately yielding **52** (0.079 g, 77% yield, 83% *ee*) as a white solid. **52**: R_f = 0.33 (silica gel, hexanes/EtOAc = 4/1); $[\alpha]_D^{25}$ = –20.8º (c = 1.00, $CHCl_3$); IR (film) ν_{max} 3364, 2934, 1726, 1508, 1451, 1248, 1179, 1024, 745, 710 cm^{-1}; ^{1}H NMR (400 MHz, $CDCl_3$) δ 7.91 (d, *J* = 8.2 Hz, 2 H), 7.82 (br s, 1 H, exchangeable), 7.61 (d, *J* = 7.5 Hz, 1 H), 7.56–7.49 (m, 1 H), 7.42–7.34 (m, 3 H), 7.32–7.27 (m, 2 H), 7.25–7.16 (m, 2 H), 6.81 (d, *J* = 9.1 Hz, 2 H), 3.77 (s, 3 H), 3.66–3.56 (m, 1 H), 3.42–3.29 (m, 1 H), 3.21–3.11 (m, 1 H), 2.91–2.78 (m, 1 H), 1.94 (s, 3 H); ^{13}C NMR (101 MHz, $CDCl_3$) δ 165.5, 159.1, 136.2, 135.5, 133.1, 129.6 (2 C), 129.4, 129.1, 128.5, 127.1, 122.1, 119.7, 118.7, 113.6, 111.2, 108.4, 65.4, 55.4, 48.0, 25.1, 18.5; HRMS (CI) calcd for $C_{26}H_{25}N_2O_3^+$ $[M + H]^+$ 413.1860, found 413.1860.

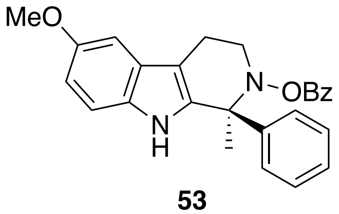

53

(*S*)-6-methoxy-1-methyl-1-phenyl-1,3,4,9-tetrahydro-2*H*-pyrido[3,4-*b*]indol-2-yl benzoate (53). Prepared using the general procedure described above with **S39** in toluene:CH_2Cl_2 (4:1), ultimately yielding **53** (0.083 g, 81% yield, 80% *ee*) as a white solid. **53**: R_f = 0.35 (silica gel, hexanes/EtOAc = 4/1); $[\alpha]_D^{25}$ = –37.0º (c = 1.00, $CHCl_3$); IR (film) ν_{max} 3362, 2936, 1728, 1486, 1451, 1246, 1166, 1062, 1023, 709 cm^{-1}; ^{1}H NMR (400 MHz, $CDCl_3$) δ 7.90 (d, *J* = 7.1 Hz, 2 H), 7.70 (br s,

1 H, exchangeable), 7.55–7.49 (m, 1 H), 7.41–7.34 (m, 4 H), 7.32–7.24 (m, 4 H), 7.06 (d, J = 2.4 Hz, 1 H), 6.90 (dd, J = 8.7, 2.4 Hz, 1 H), 3.90 (s, 3 H), 3.66–3.58 (m, 1 H), 3.38–3.27 (m, 1 H), 3.18–3.08 (m, 1 H), 2.86–2.74 (m, 1 H), 1.95 (s, 3 H); ^{13}C NMR (101 MHz, $CDCl_3$) δ 165.5, 154.3, 136.1, 133.2, 131.3, 129.6, 129.3, 128.5, 128.4 (2 C), 127.9 (2 C), 127.5, 112.0, 111.9, 108.4, 100.9, 66.0, 56.1, 48.1, 25.1, 18.5; HRMS (ESI) calcd for $C_{26}H_{25}N_2O_3^+$ $[M + H]^+$ 413.1860, found 413.1861.

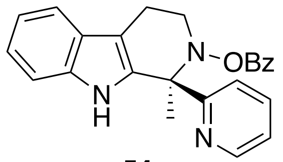

54

(*R*)-1-methyl-1-(pyridin-2-yl)-1,3,4,9-tetrahydro-2*H*-pyrido[3,4-*b*]indol-2-yl benzoate (54). Prepared using the general procedure described above with **S21** in toluene:CH_2Cl_2 (4:1), ultimately yielding **54** (0.069 g, 72% yield, 55% *ee*) as a white solid. **54**: R_f = 0.21 (silica gel, hexanes/EtOAc = 4/1); $[\alpha]_D^{25}$ = +21.2 (c = 1.00, $CHCl_3$); IR (film) ν_{max} 3395, 2991, 2937, 1739, 1588, 1451, 1245, 1058, 737, 709 cm^{-1}; ^{1}H NMR (400 MHz, $CDCl_3$) δ 8.62–8.55 (m, 1 H), 8.02–7.92 (m, 1 H), 7.91–7.70 (m, 2 H), 7.66–7.58 (m, 1 H), 7.57–7.46 (m, 2 H), 7.43–7.29 (m, 3 H), 7.21–7.06 (m, 4 H), 3.92–3.69 (m, 2 H), 3.22–3.10 (m, 1 H), 3.05–2.94 (m, 1 H), 2.05 (s, 3 H); ^{13}C NMR (101 MHz, $CDCl_3$) δ 165.3, 163.4, 148.5, 137.3, 136.6, 136.3, 133.2, 129.5, 129.2, 128.5, 126.7, 122.3, 121.8, 120.9, 119.4, 118.5, 111.3, 106.6, 67.5, 48.5, 24.0, 19.5; HRMS (ESI) calcd for $C_{24}H_{22}N_3O_2^+$ $[M + H]^+$ 384.1707, found 384.1707.

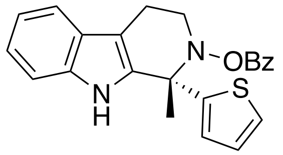

55

(*S*)-1-methyl-1-(thiophen-2-yl)-1,3,4,9-tetrahydro-2*H*-pyrido[3,4-*b*]indol-2-yl benzoate (55). Prepared using the general procedure described above with **S22**, ultimately yielding **55** (0.080 g, 82% yield, -53% *ee*) as a white solid. **55**: R_f = 0.43 (silica gel, hexanes/EtOAc = 4/1); $[\alpha]_D^{25}$ = +11.1° (c = 1.00, $CHCl_3$); IR (film) ν_{max} 3365, 2924, 1730, 1451, 1265, 1177, 1062, 1024, 740, 708 cm^{-1}; ^{1}H NMR (400 MHz, $CDCl_3$) δ 7.97 (d, J = 7.2 Hz, 2 H), 7.78 (br s, 1 H, exchangeable), 7.62–7.51 (m, 2 H), 7.44–7.33 (m, 3 H), 7.31 (d, J = 5.4 Hz, 1 H), 7.25–7.13 (m, 2 H), 6.94–6.87 (m, 1 H), 6.79 (s, 1 H), 3.68–3.49 (m, 2 H), 3.25–3.11 (m, 1 H), 3.01–2.91 (m, 1 H), 2.06 (s, 3 H); ^{13}C NMR (101 MHz, $CDCl_3$) δ 165.2, 136.4, 135.1, 133.2, 129.7 (2 C), 129.2, 128.5 (2 C), 126.7, 126.3, 126.2, 122.3, 119.8, 118.8, 111.3, 108.1, 63.9, 48.5, 26.3, 19.4; HRMS (CI) calcd for $C_{23}H_{21}N_2O_2S^+$ $[M + H]^+$ 389.1318, found 389.1317.

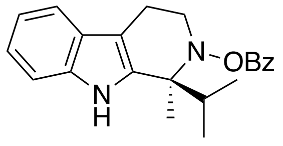

56

(*S*)-1-isopropyl-1-methyl-1,3,4,9-tetrahydro-2*H*-pyrido[3,4-*b*]indol-2-yl benzoate (56). Prepared using the general procedure described above with **S26**, ultimately yielding **56** (0.052 g, 60% yield, 62% *ee*) as a white solid. **56**: R_f = 0.50 (silica gel, hexanes/EtOAc = 4/1); $[\alpha]_D^{25}$ = –30.8° (c = 1.00, $CHCl_3$); IR (film) ν_{max} 3383, 2965, 1724, 1451, 1270, 1089, 1064, 1024, 738, 710 cm^{-1}; ^{1}H NMR (500 MHz, $CDCl_3$) δ 7.96–7.91 (m, 2 H), 7.76 (br s, 1 H, exchangeable), 7.57–7.50 (m, 2 H), 7.43–7.35 (m, 3 H), 7.23–7.18 (m, 1 H), 7.17–7.11 (m, 1 H), 3.75–3.62 (m, 2 H), 3.04–2.96 (m, 1 H), 2.96–2.88 (m, 1 H), 2.18–2.09 (m, 1 H), 1.54 (s, 3 H), 1.16 (d, J = 6.8 Hz, 3 H), 1.05 (d, J = 6.8 Hz, 3 H); ^{13}C NMR (101 MHz, $CDCl_3$) δ 165.3, 137.4, 136.0, 133.1, 129.6 (2 C), 128.6, 126.9, 121.8, 119.5, 118.5, 110.9, 107.6, 65.5, 48.1, 37.4, 18.9, 18.7, 18.1, 18.0; HRMS (CI) calcd for $C_{22}H_{25}N_2O_2^+$ $[M + H]^+$ 349.1911, found 349.1910.

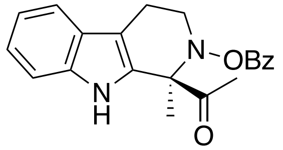

57

(*R*)-1-acetyl-1-methyl-1,3,4,9-tetrahydro-2*H*-pyrido[3,4-*b*]indol-2-yl benzoate (57). Prepared using the general procedure described above with **S31**, ultimately yielding **57** (0.064 g, 74% yield, 16% *ee*) as a white

solid. **57**: R_f = 0.38 (silica gel, hexanes/EtOAc = 4/1); $[\alpha]_D^{25}$ = –6.2º (c = 1.00, $CHCl_3$); IR (film) ν_{max} 3378, 2943, 1745, 1631, 1452, 1244, 1057, 1024, 737, 709 cm^{-1}; ^1H NMR (400 MHz, $CDCl_3$) δ 8.49 (br s, 1 H, exchangeable), 7.90 (d, *J* = 8.1 Hz, 2 H), 7.60–7.51 (m, 2 H), 7.45–7.35 (m, 3 H), 7.25–7.19 (m, 1 H), 7.17–7.11 (m, 1 H), 3.98–3.85 (m, 1 H), 3.65–3.52 (m, 1 H), 3.21–3.09 (m, 1 H), 2.96–2.83 (m, 1 H), 2.35 (s, 3 H), 1.77 (s, 3 H); ^{13}C NMR (101 MHz, $CDCl_3$) δ 210.0, 164.8, 136.7, 133.7, 132.2, 129.6, 128.7, 128.7, 126.5, 122.5, 119.8, 118.6, 111.5, 108.0, 72.1, 48.4, 24.9, 21.3, 19.0; HRMS (ESI) calcd for $C_{21}H_{21}N_2O_3^+$ $[M + H]^+$ 349.1547, found 349.1546.

I. Derivatizations of Pictet–Spengler Products.

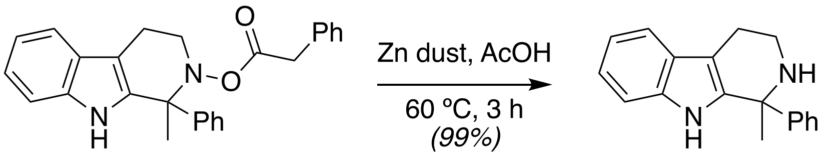

20e **58**

1-methyl-1-phenyl-2,3,4,9-tetrahydro-1*H*-pyrido[3,4-*b*]indole (58).[11,12] To a solution of **20e** (0.041 g, 0.10 mmol, 1.0 equiv) in AcOH (1.5 mL) at 23 ºC was added activated Zn powder (0.101 g, 1.55 mmol, 15.0 equiv) in a microwave tube open to air. The microwave tube was then sealed and the reaction was heated to 60 ºC with stirring for 3 h. Upon completion, the contents were cooled to 23 ºC, filtered through a pad of Celite, washed with MeOH, and concentrated. The crude product was then diluted with CH_2Cl_2 (10 mL) and washed with a 1:1 mixture of aqueous NH_3 (5 mL, 30 wt. %) and saturated aqueous $NaHCO_3$ (5 mL). The remaining aqueous layer was extracted with CH_2Cl_2 (3 × 10 mL). Next, the combined organic extracts were washed with saturated aqueous $NaHCO_3$ (10 mL) and the new aqueous layer was extracted with CH_2Cl_2 (2 × 10 mL). The combined organic extracts were then dried (Na_2SO_4), filtered, and concentrated to yield pure **58** (0.027 g, 99% yield) as a white solid. **58**: R_f = 0.29 (silica gel, CH_2Cl_2/MeOH = 9/1); IR (film) ν_{max} 3404, 1597, 1564, 1449, 1392, 1295, 1121, 858, 744, 700 cm^{-1}; ^1H NMR (400 MHz, $CDCl_3$) δ 7.94 (br s, 1 H, exchangeable), 7.56 (d, *J* = 6.8 Hz, 1 H), 7.32–7.22 (m, 5 H), 7.21–7.10 (m, 2 H), 3.19–3.07 (m, 1 H), 2.95–2.69 (m, 3 H), 1.91 (br s, 1 H, exchangeable), 1.84 (s, 3 H); ^{13}C NMR (101 MHz, $CDCl_3$) δ 146.2, 138.2, 135.8, 128.4, 127.4, 127.4, 127.0, 121.9, 119.5, 118.5, 111.0, 109.7, 57.0, 39.8, 28.3, 22.8; HRMS (ESI) calcd for $C_{18}H_{19}N_2^+$ $[M + H]^+$ 263.1543, found 263.1545.

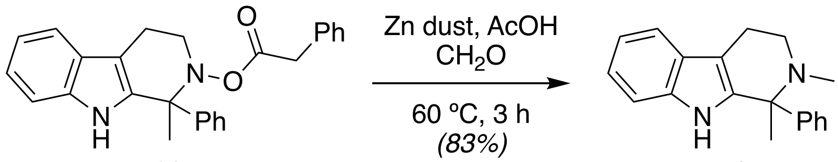

20e **59**

1,2-dimethyl-1-phenyl-2,3,4,9-tetrahydro-1*H*-pyrido[3,4-*b*]indole (59).[11,13] To a solution of **20e** (0.041 g, 0.10 mmol, 1.0 equiv) in AcOH (1.5 mL) at 23 ºC sequentially added CH_2O (0.050 mL, 37 wt. % in H_2O, 0.62 mmol, 6.0 equiv) and activated Zn powder (0.101 g, 1.55 mmol, 15.0 equiv) in a microwave tube open to air. The microwave tube was then sealed and the reaction was heated to 60 ºC with stirring for 3 h. Upon completion, the contents were cooled to 23 ºC, filtered through a pad of Celite, washed with MeOH, and concentrated. The

crude product was then diluted with CH_2Cl_2 (10 mL) and washed with a 1:1 mixture of aqueous NH_3 (5 mL, 30 wt. %) and saturated aqueous $NaHCO_3$ (5 mL). The remaining aqueous layer was extracted with CH_2Cl_2 (3 × 10 mL). Next, the combined organic extracts were washed with saturated aqueous $NaHCO_3$ (10 mL) and the new aqueous layer was extracted with CH_2Cl_2 (2 × 10 mL). The combined organic extracts were then dried (Na_2SO_4), filtered, and concentrated to yield pure **59** (0.024 g, 84% yield) as a yellow solid. **59**: R_f = 0.44 (silica gel, CH_2Cl_2/MeOH = 9/1); IR (film) ν_{max} 3412, 2942, 1447, 1297, 1251, 1179, 1026, 909, 741, 702 cm^{-1}; ^1H NMR (500 MHz, $CDCl_3$) δ 7.57–7.53 (m, 1 H), 7.45–7.40 (m, 2 H), 7.34–7.29 (m, 3 H), 7.29–7.24 (m, 1 H), 7.22–7.19 (m, 1 H), 7.16–7.09 (m, 2 H), 3.04–2.94 (m, 3 H), 2.94–2.86 (m, 1 H), 2.27 (s, 3 H), 1.79 (s, 3 H); ^{13}C NMR (101 MHz, $CDCl_3$) δ 144.5, 139.4, 136.3, 128.2, 127.9, 127.4, 127.2, 121.6, 119.4, 118.5, 110.9, 108.3, 61.0, 47.9, 37.7, 21.2, 19.3; HRMS (ESI) calcd for $C_{19}H_{21}N_2^+$ $[M + H]^+$ 277.1699, found 277.1698.

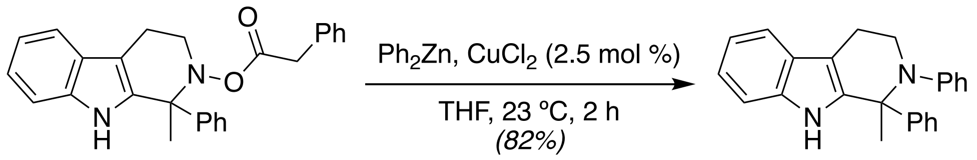

20e **60**

1-methyl-1,2-diphenyl-2,3,4,9-tetrahydro-1*H*-pyrido[3,4-*b*]indole (60).[14] To a solution of PhMgBr (1.0 M in THF, 1.50 mL, 1.5 mmol, 1.0 equiv) in THF (1.5 mL) at 0 ºC was added a freshly prepared solution of $ZnCl_2$ (1.0 M in THF, 0.75 mL, 0.75 mmol, 0.5 equiv) under an argon atmosphere. The resultant mixture warmed to 23 ºC and stirred for 15 min. Upon completion, the diorganozinc reagent was titrated using iodine to determine the concentration (here as 0.19 M).[15] Then, the freshly prepared solution of Ph_2Zn (0.19 M, 0.73 mL, 0.14 mmol, 1.1 equiv) was added to a solution of **20e** (0.050 g, 0.13 mmol, 1.0 equiv) and $CuCl_2$ (0.4 mg, 0.003 mmol, 0.025 equiv) in THF (1.0 mL) at 23 ºC under an argon atmosphere. The resultant mixture was stirred at 23 ºC for 2 h. Upon completion, the contents were quenched with saturated aqueous $NaHCO_3$ (5 mL), poured into a separatory funnel, and extracted with EtOAc (3 × 5 mL). The combined organic extracts were then dried (Na_2SO_4), filtered, and concentrated. The resultant crude material was purified by flash column chromatography (silica gel, hexanes/EtOAc = 1/0→4/1) to yield **60** (0.035 g, 82% yield) as a white solid. **60**: R_f = 0.74 (silica gel, hexanes/EtOAc = 4/1); IR (film) ν_{max} 3413, 2909, 1594, 1490, 1449, 1288, 1231, 908, 739, 701 cm^{-1}; ^1H NMR (500 MHz, $CDCl_3$) δ 7.65–7.57 (m, 1 H), 7.40 (br s, 1 H, exchangeable), 7.29–7.19 (m, 6 H), 7.19–7.08 (m, 4 H), 7.03 (t, J = 7.3 Hz, 1 H), 6.75 (d, J = 7.8 Hz, 2 H), 3.55 (t, J = 6.0 Hz, 2 H), 3.10–2.96 (m, 2 H), 1.80 (s, 3 H); ^{13}C NMR (101 MHz, $CDCl_3$) δ 149.5, 145.0, 139.8, 136.4, 128.8, 128.2, 128.0, 127.4, 127.1, 126.8, 123.9, 121.8, 119.5, 118.5, 111.0, 109.2, 62.1, 47.4, 22.3, 22.2; HRMS (ESI) calcd for $C_{24}H_{23}N_2^+$ $[M + H]^+$ 339.1856, found 339.1869.

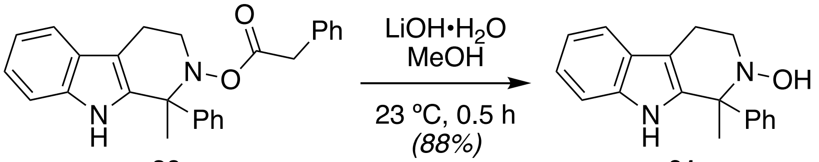

20e **61**

1-methyl-1-phenyl-1,3,4,9-tetrahydro-2*H*-pyrido[3,4-*b*]indol-2-ol (61).[16] To a solution of **20e** (0.100 g, 0.25 mmol, 1.0 equiv) in MeOH (2.0 mL) at 23 ºC was added LiOH•H_2O (0.012

mg, 0.28 mmol, 1.1 equiv) open to air. The resultant mixture was stirred at 23 ºC for 30 min, during which time the product crashed out as a white precipitate. Upon completion, the reaction contents were concentrated directly and then diluted with CH_2Cl_2/MeOH (9/1, 10 mL). Deionized H_2O (5 mL) was then added, the contents were poured into a separatory funnel, and the product was extracted with CH_2Cl_2/MeOH (9/1, 3 × 10 mL). The combined organic extracts were then dried (Na_2SO_4), filtered, and concentrated. The resultant crude material was purified by flash column chromatography (silica gel, hexanes/EtOAc = 1/0→2/1) or recrystallization (CH_2Cl_2) to yield **61** (0.062 mg, 88% yield) as a white solid. **61**: R_f = 0.59 (silica gel, CH_2Cl_2/MeOH = 9/1); IR (film) ν_{max} 3410, 1597, 1564, 1449, 1393, 1232, 858, 799, 735, 700 cm^{-1}; ^1H NMR (500 MHz, DMSO-d_6) δ 10.75 (br s, 1 H, exchangeable), 7.94 (br s, 1 H, exchangeable), 7.42 (d, J = 7.7 Hz, 1 H), 7.32–7.23 (m, 3 H), 7.22–7.12 (m, 3 H), 7.07–7.01 (m, 1 H), 7.00–6.94 (m, 1 H), 3.21–3.05 (m, 1 H), 2.99–2.84 (m, 2 H), 2.66–2.52 (m, 1 H), 1.81 (s, 3 H); ^{13}C NMR (101 MHz, DMSO-d_6) δ 137.3, 136.2, 127.8, 127.5, 127.1, 126.7, 126.3, 120.6, 118.3, 117.8, 111.0, 106.3, 64.6, 49.0, 24.1, 18.4; HRMS (ESI) calcd for $C_{18}H_{19}N_2O^+$ $[M + H]^+$ 279.1492, found 279.1495.

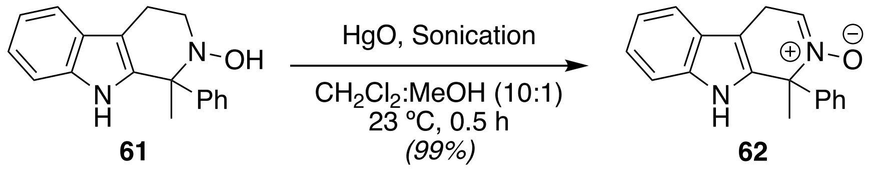

1-methyl-1-phenyl-4,9-dihydro-1*H*-pyrido[3,4-*b*]indole 2-oxide (62).[17] To a solution of **61** (0.042 g, 0.15 mmol, 1.0 equiv) in CH_2Cl_2/MeOH (10/1, 1.5 mL) at 23 ºC was added yellow HgO (0.098 g, 0.44 mmol, 3.0 equiv) under an argon atmosphere. The reaction contents were then sonicated for 0.5 h. Upon completion, $MgSO_4$ (0.055 g, 0.45 mmol, 3.0 equiv) was added and the reaction contents were stirred for an additional 10 min. The resulting grey heterogeneous mixture was filtered through a pad of Celite with a layer of $MgSO_4$ on top, washed with CH_2Cl_2 (15 mL), and then concentrated to afford nitrone **62** (0.041 g, 99% yield) that was carried forward without further purification.

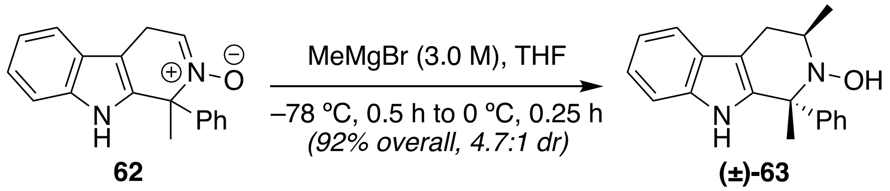

To a solution of **62** (0.041 g, 0.15 mmol, 1.0 equiv) in THF (3.0 mL) at –78 ºC was added dropwise MeMgBr (3.0 M in Et_2O, 0.49 mL, 1.49 mmol, 10 equiv) over the course of 15 min under an argon atmosphere. The resultant mixture was then stirred at –78 ºC for an additional 30 min before being warmed to 0 ºC and stirred for 15 min. Upon completion, the reaction contents were quenched with saturated aqueous NH_4Cl (5 mL) and extracted with EtOAc (3 × 5 mL). The combined organic extracts were then dried (Na_2SO_4), filtered, and concentrated. The resultant crude material was purified by flash column chromatography (silica gel, hexanes/EtOAc = 1/0→3/1) to yield **63a** (0.033 g, 76% yield) and **63b** (0.007 g, 16% yield) as white solids (0.040 g combined, 92% yield overall, 4.7:1 *dr*).[18]

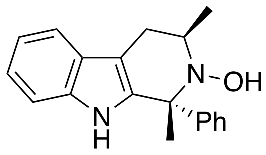

(±)-63a

(±)-(1*R*,3*R*)-1,3-dimethyl-1-phenyl-1,3,4,9-tetrahydro-2*H*-pyrido[3,4-*b*]indol-2-ol (63a): R_f = 0.25 (silica gel, hexanes/EtOAc = 4/1); IR (film) ν_{max} 3410, 2975, 2932, 1449, 1299, 1234, 1184, 910, 738, 700 cm^{-1}; ^{1}H NMR (500 MHz, $CDCl_3$) δ 7.89 (br s, 1 H, exchangeable), 7.56 (d, *J* = 7.8 Hz, 1 H), 7.39 (d, *J* = 8.1 Hz, 1 H), 7.26–7.18 (m, 6 H), 7.18–7.13 (m, 1 H), 4.52 (br s, 1 H, exchangeable), 3.20–3.09 (m, 1 H), 2.85–2.77 (m, 1 H), 2.70–2.56 (m, 1 H), 1.94 (s, 3 H), 1.26 (d, *J* = 6.6 Hz, 3 H); ^{13}C NMR (101 MHz, $CDCl_3$) δ 136.6, 133.7, 128.4, 127.8, 127.4, 127.3, 126.9, 122.2, 119.8, 118.8, 111.2, 109.1, 67.4, 52.7, 29.8, 26.1, 19.5; HRMS (ESI) calcd for $C_{19}H_{21}N_2O^+$ $[M + H]^+$ 293.1648, found 293.1658.

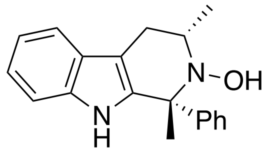

(±)-63b

(±)-(1*R*,3*S*)-1,3-dimethyl-1-phenyl-1,3,4,9-tetrahydro-2*H*-pyrido[3,4-*b*]indol-2-ol (63b): R_f = 0.37 (silica gel, hexanes/EtOAc = 4/1); IR (film) ν_{max} 3532, 3399, 2928, 1452, 1374, 1303, 1238, 908, 734, 701 cm^{-1}; ^{1}H NMR (500 MHz, $CDCl_3$) δ 7.56–7.47 (m, 3 H), 7.36–7.28 (m, 3 H), 7.25–7.15 (m, 2 H), 7.14–7.07 (m, 2 H), 4.48 (br s, 1 H, exchangeable), 3.63–3.52 (m, 1 H), 2.99–2.89 (m, 1 H), 2.85–2.76 (m, 1 H), 1.90 (s, 3 H), 1.43 (d, *J* = 6.1 Hz, 3 H); ^{13}C NMR (101 MHz, $CDCl_3$) δ 144.5, 138.9, 136.5, 130.6, 128.4, 127.6, 126.7, 121.7, 119.5, 118.5, 111.0, 107.3, 66.1, 53.5, 29.9, 29.3, 20.5; HRMS (ESI) calcd for $C_{19}H_{21}N_2O^+$ $[M + H]^+$ 293.1648, found 293.1648.

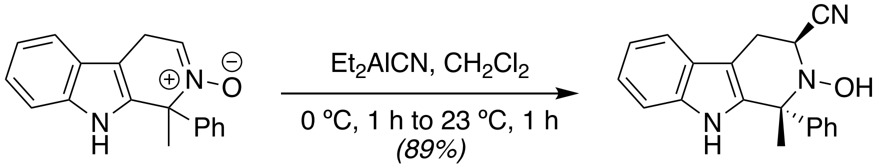

(±)-(1*R*,3*S*)-2-hydroxy-1-methyl-1-phenyl-2,3,4,9-tetrahydro-1*H*-pyrido[3,4-*b*]indole-3-carbonitrile (64).[19] To a solution of **62** (0.041 g, 0.15 mmol, 1.0 equiv) in CH_2Cl_2 (3.0 mL) at 0 ºC was added Et_2AlCN (1.0 M in toluene, 0.45 mL, 0.45 mmol, 3.0 equiv) under an argon atmosphere. The resultant mixture was stirred at 0 ºC for 1 h and then warmed to 23 ºC and stirred for an additional 1 h. Upon completion, the reaction contents were quenched with saturated aqueous $NaHCO_3$ (5 mL), poured into a separatory funnel, and extracted with CH_2Cl_2 (3 × 5 mL). The combined organic extracts were then dried (Na_2SO_4), filtered, and concentrated. The resultant crude material was purified by flash column chromatography (silica gel, hexanes/EtOAc = 1/0→3/1) to yield **64** (0.40 mg, 89% yield) as a white solid. **64**: R_f = 0.24 (silica gel, hexanes/EtOAc = 4/1); IR (film) ν_{max} 3404, 3058, 2920, 2258, 1453, 1300, 1183, 1028, 739, 701 cm^{-1}; ^{1}H NMR (500 MHz, $CDCl_3$) δ 7.93 (br s, 1 H, exchangeable), 7.56 (d, *J* = 7.9 Hz, 1 H), 7.40 (d, *J* = 8.1 Hz, 1 H), 7.30–7.26 (m, 3 H), 7.26–7.23 (m, 1 H), 7.21–7.12 (m, 3 H), 5.26 (br s, 1 H, exchangeable), 4.09–4.00 (m, 1 H), 3.57–3.47 (m, 1 H), 3.06–2.97 (m, 1 H), 1.96 (s, 3 H); ^{13}C NMR (101 MHz, $CDCl_3$) δ 136.2, 133.6, 128.7, 128.4, 127.3, 127.1, 126.4, 122.7, 120.3, 119.7, 118.7, 111.3, 106.2, 66.3, 50.2, 25.6, 22.1; HRMS (ESI) calcd for $C_{19}H_{18}N_3O^+$ $[M + H]^+$ 304.1444, found 304.1454.

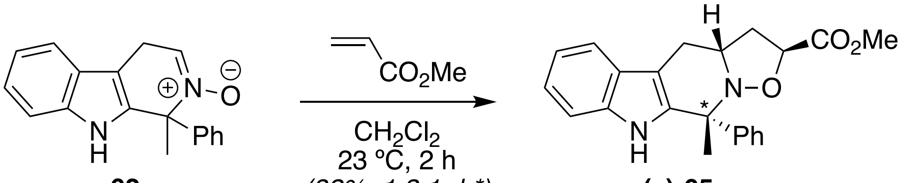

62 **(±)-65**

To a solution of **62** (0.041 g, 0.15 mmol, 1.0 equiv) in CH_2Cl_2 (3.0 mL) at 23 ºC was added methyl acrylate (0.13 mL, 1.5 mmol, 10 equiv) under an argon atmosphere and stirred for 2 h. Upon completion, the reaction contents were quenched with saturated aqueous $NaHCO_3$ (5 mL) and extracted with CH_2Cl_2 (3 × 5 mL). The combined organic extracts were then dried (Na_2SO_4), filtered, and concentrated. The resultant crude material was purified by preparative thin layer chromatography (silica gel, CH_2Cl_2/MeOH = 15/1) to yield **65a** (0.025 mg, 46% yield) and **65b** (0.019 mg, 35% yield) as yellow solids (0.044 mg combined, 82% yield overall, 1.3:1 *dr*).[20]

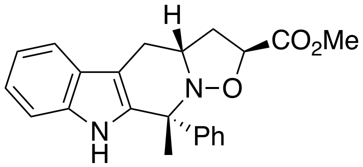

(±)-65a

(±)-methyl (2*S*,3a*R*,10*R*)-10-methyl-10-phenyl-2,3,3a,4,9,10-hexahydroisoxazolo[2',3':1,6]pyrido[3,4-*b*]indole-2-carboxylate (65a): R_f = 0.32 (silica gel, hexanes/EtOAc = 4/1); IR (film) ν_{max} 3386, 2924, 1741, 1454, 1373, 1213, 1102, 1028, 742, 703 cm^{-1}; ^1H NMR (500 MHz, $CDCl_3$) δ 7.61–7.54 (m, 2 H), 7.52–7.46 (m, 1 H), 7.38–7.26 (m, 4 H), 7.21–7.17 (m, 1 H), 7.16–7.07 (m, 2 H), 4.61 (dd, J = 8.0, 5.4 Hz, 1 H), 3.76 (s, 3 H), 3.65–3.56 (m, 1 H), 3.23–3.14 (m, 1 H), 2.89–2.79 (m, 1 H), 2.63–2.56 (m, 2 H), 1.99 (s, 3 H); ^{13}C NMR (101 MHz, $CDCl_3$) δ 172.3, 143.9, 139.6, 136.9, 128.6, 127.8, 127.5, 126.4, 122.0, 119.7, 118.4, 111.2, 107.8, 74.4, 64.7, 55.7, 52.3, 38.9, 26.1, 17.6; HRMS (ESI) calcd for $C_{22}H_{23}N_2O_3^+$ $[M + H]^+$ 363.1703, found 363.1717.

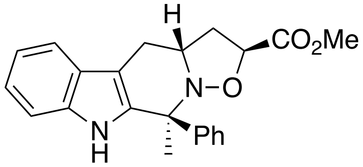

(±)-65b

(±)-methyl (2*S*,3a*R*,10*S*)-10-methyl-10-phenyl-2,3,3a,4,9,10-hexahydroisoxazolo[2',3':1,6]pyrido[3,4-*b*]indole-2-carboxylate (65b): R_f = 0.26 (silica gel, hexanes/EtOAc = 4/1); IR (film) ν_{max} 3373, 2924, 1738, 1453, 1376, 1210, 1103, 1029, 741, 700 cm^{-1}; ^1H NMR (500 MHz, $CDCl_3$) δ 7.75 (br s, 1 H, exchangeable), 7.54 (d, J = 8.7 Hz, 1 H), 7.34 (d, J = 7.9 Hz, 1 H), 7.26–7.11 (m, 7 H), 4.62 (dd, J = 9.2, 4.0 Hz, 1 H), 3.52 (s, 3 H), 3.22–3.01 (m, 2 H), 2.81–2.71 (m, 1 H), 2.52–2.36 (m, 2 H), 2.09 (s, 3 H); ^{13}C NMR (101 MHz, $CDCl_3$) δ 171.6, 137.0, 136.5, 130.1, 130.0, 127.7, 127.3, 126.4, 122.2, 119.9, 118.6, 111.3, 109.3, 74.4, 64.9, 55.0, 52.2, 38.4, 26.0, 25.2; HRMS (ESI) calcd for $C_{22}H_{23}N_2O_3^+$ $[M + H]^+$ 363.1703, found 363.1714.

J. Mechanistic Understanding

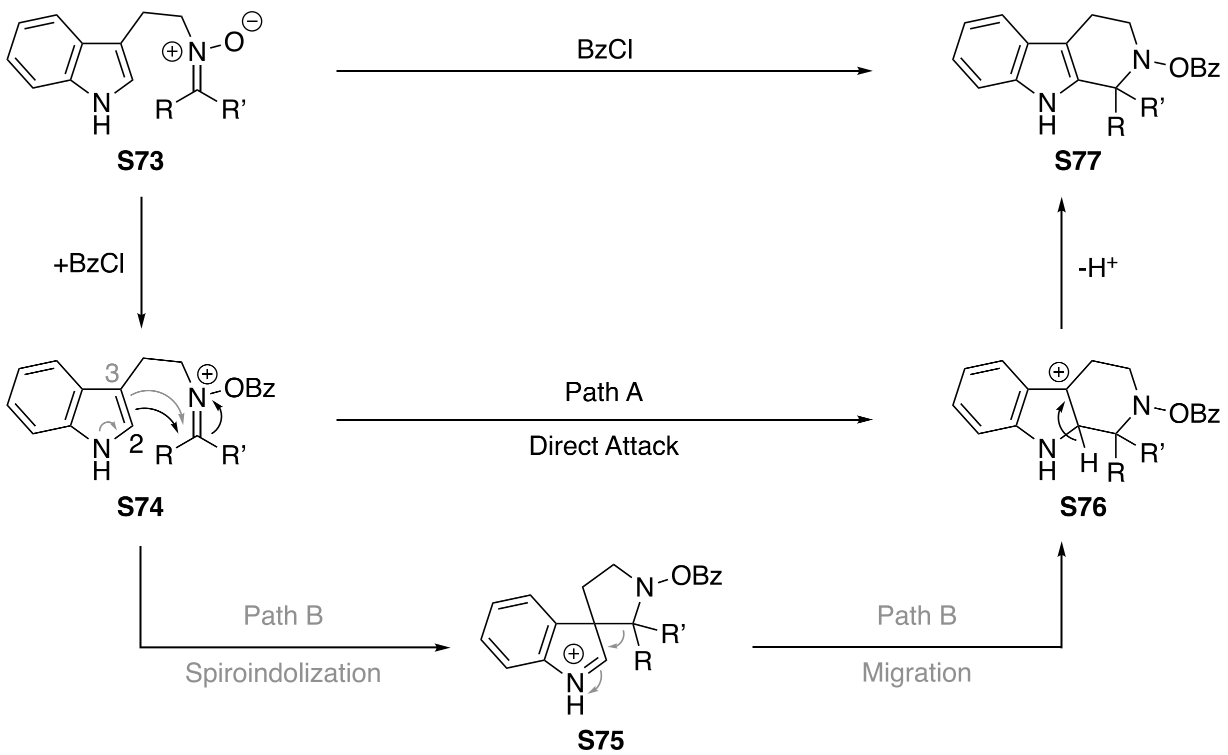

Figure S2. Current mechanistic understanding of the developed ketonitrone-based Pictet–Spengler reaction.

Investigations into the mechanism of Pictet–Spengler-type reactions have been of high interest for several decades. There are two possible reaction mechanisms (Figure S2): direct attack of the C2 position of indole to the *N*-acyloxyiminium species (Path A) or attack of the C3 position of the indole to undergo a stepwise spiroindolization/migration sequence (Path B). Although historically there has been experimental evidence supporting the fast and reversible formation of the spiroindolenine (like **S75**), it is still unclear if this intermediate ultimately rearranges to the carbonium ion (like **S76**).[21] Nonetheless, according to recent computational studies, there is a strong energetic preference for C2 addition over C3 addition.[9d,22] These studies also propose that rearomatization via deprotonation of the carbonium ion intermediate is both the rate- and enantioselective-determining step. Hence, it is challenging to propose which pathway our developed reaction undergoes without kinetic, computational, and/or structure–activity relationship studies, especially since it involves an *N*-acyloxyiminium species that has not been previously explored in this reaction type. Nonetheless, these mechanistic studies are of current interest.

K. References

1. J. K. Kerkouvius and M. A. Kerr, *J. Am. Chem. Soc.*, 2018, **140**, 8415.
2. C. Winter and N. Krause, *Angew. Chem.*, *Int. Ed.*, 2009, **48**, 6339.
3. a) M. Figerio, M. Santagostino and S. Sputore, *J. Org. Chem.*, 1999, **64**, 4537; b) G. Relevant, S. Dundand, S. Hesse and G. Kirsh, *Synthesis*, 2011, **18**, 2935.
4. W. Oppolzer, S. Siles, R. L. Snowden, B. H. Bakker and M. Petrzilka, *Tetrahedron*, 1985, **41**, 3497.
5. W. Liu, H. Wang, X. Li, Y. Xu, J. Zhang, W. Wang, Q. Gong, X. Qiu, J. Zhu, F. Mao, H. Zhang and J. Li, *Bioorg. Med. Chem*., 2018, **26**, 3117.
6. L. Fu and H. M. L. Davies, *Org. Lett*., 2017, **19**, 1504.
7. S.-Y. Han, M. V. Lakshmikantham and M. P. Cava, *Heterocycles*, 1985, **23**, 1671.
8. K. Brak and E. N. Jacobsen*, Angew. Chem.*, *Int. Ed.*, 2013, **52**, 534.
9. a) M. S. Taylor and E. N. Jacobsen, *J. Am. Chem. Soc.*, 2004, **126**, 10558; b) I. T. Raheem, P. S. Thiara, E. A. Peterson and E. N. Jacobsen, *J. Am. Chem. Soc.*, 2007, **129**, 13404; c) R. S. Klausen and E. N. Jacobsen, *Org. Lett.*, 2009, **11**, 887; d) R. S. Klausen, C. R. Kennedy, A. M. Hyde and E. N. Jacobsen, *J. Am. Chem. Soc.*, 2017, **139**, 12299.
10. E. N. Jacobsen and S. J. Zuend, US2013/66109, 2013, **A1**.
11. V. G. Lisnyak, T. Lynch-Colameta and S. A. Snyder, *Angew. Chem.*, *Int. Ed.*, 2018, **57**, 15162.
12. G. S. King, P. D. Magnus and H. S. Rzepa, *J. Chem. Soc., Perkin Trans. 1*, 1972, 437.
13. R. A. da Silva, I. H. S. Estevam and L. W. Bieber, *Tetrahedron Lett.*, 2007, **48**, 7680.
14. A. M. Berman and J. S. Johnson, *J. Am. Chem. Soc.*, 2004, **126**, 5680.
15. A. Krasovskiy and P. Knochel, *Synthesis*, 2006, 890.
16. A. Banerjee and H. Yamamoto, *Chem. Sci.*, 2019, **10**, 2124.
17. a) E. Gössinger, *Tetrahedron Lett.*, 1980, **21**, 2229; b) S. Cicchi, A. Goti and A. Brandi, *J. Org. Chem.*, 1995, **60**, 4743.
18. P. Merino, V. Mannucci and T. Tejero, *Eur. J. Org. Chem.*, 2008, 3943.
19. a) F. L. Merchan, P. Merino and T. Tejero, *Tetrahedron Lett.*, 1995, **36**, 6949; b) P. Merino, A. Lanaspa, F. L. Merchan and T. Tejero, *J. Org. Chem.*, 1996, **61**, 9028; c) A. Goti, S. Cicchi, V. Mannucci, F. Cardona, F. Guarna, P. Merino and T. Tejero, *Org. Lett.*, 2003, **5**, 4235.
20. a) S. A. Ali and M. I. M. Wazeer, *J. Chem. Soc., Perkin Trans. 2*, 1986, 1789; b) S. A. Ali and M. I. M. Wazeer, *J. Chem. Soc., Perkin Trans. 1*, 1988, 597; c) S. A. Ali and M. I. M. Wazeer, *Tetrahedron*, 1988, **44**, 187.
21. a) A. H. Jackson and A. E. Smith, *Tetrahedron*, 1968, **24**, 403; b) F. Ungemach and J. M. Cook, *Heterocycles*, 1978, **9**, 1089; c) P. D. Bailey, *J. Chem. Res*., 1987, 202; d) J. Liu, M. Nakagawa, K. Ogata and T. Hino, *Chem. Pharm. Bull.*, 1991, **39**, 1672; e) K. M. Czerwinski, L. Deng and J. M. Cook, *Tetrahedron Lett.*, 1992, **33**, 4721 f) C. Zheng, Z.-L. Xia and S.-L. You, *Chem*, 2018, **4**, 1952; g) C. Zheng and S.-L. You, *Acc. Chem. Res.*, 2020, **53**, 974.
22. a) P. Kowalski, A. J. Bojarski and J. L. Mokrosz, *Tetrahedron*, 1995, **51**, 2737; b) J. J. Maresh, L.-A. Giddings, A. Friedrich, E. A. Loris, S. Panjikar, B. L. Trout, J. Stöckigt, B. Peters and S. E. O'Connor, *J. Am. Chem. Soc.*, 2008, **130**, 710; c) L. M. Overvoorde, M. N. Grayson, Y. Luo and J. M. Goodman, *J. Org. Chem.*, 2015, **80**, 2634.

L. NMR Spectra

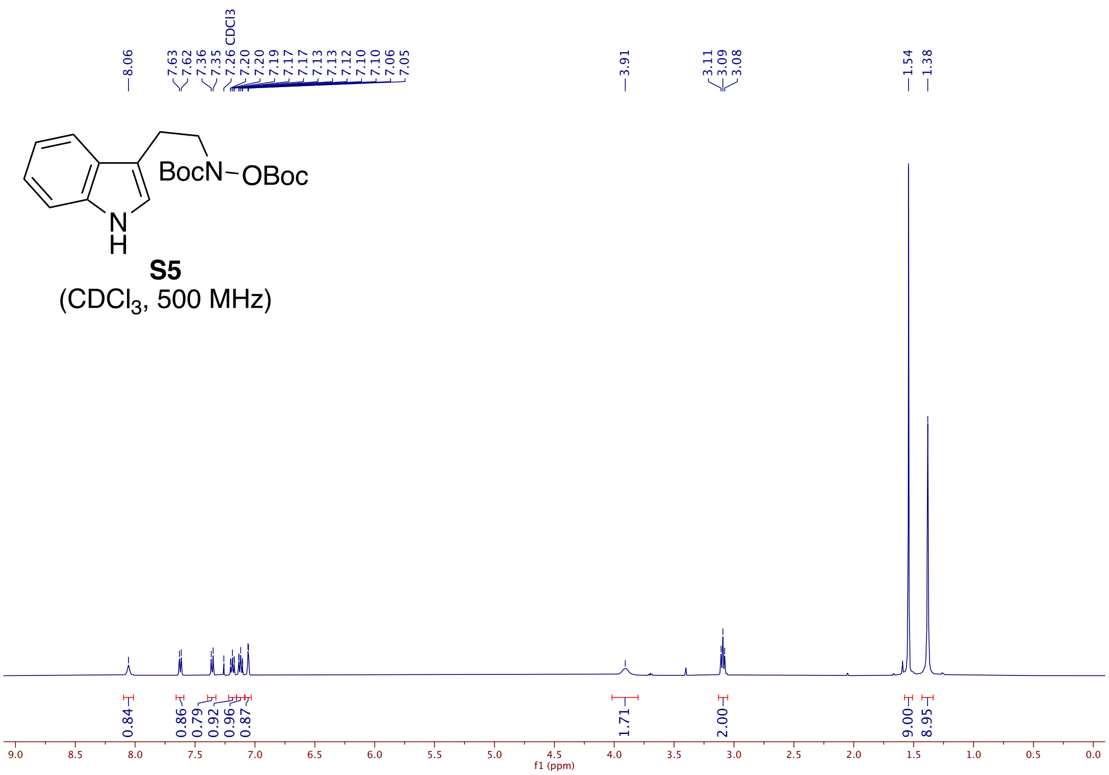

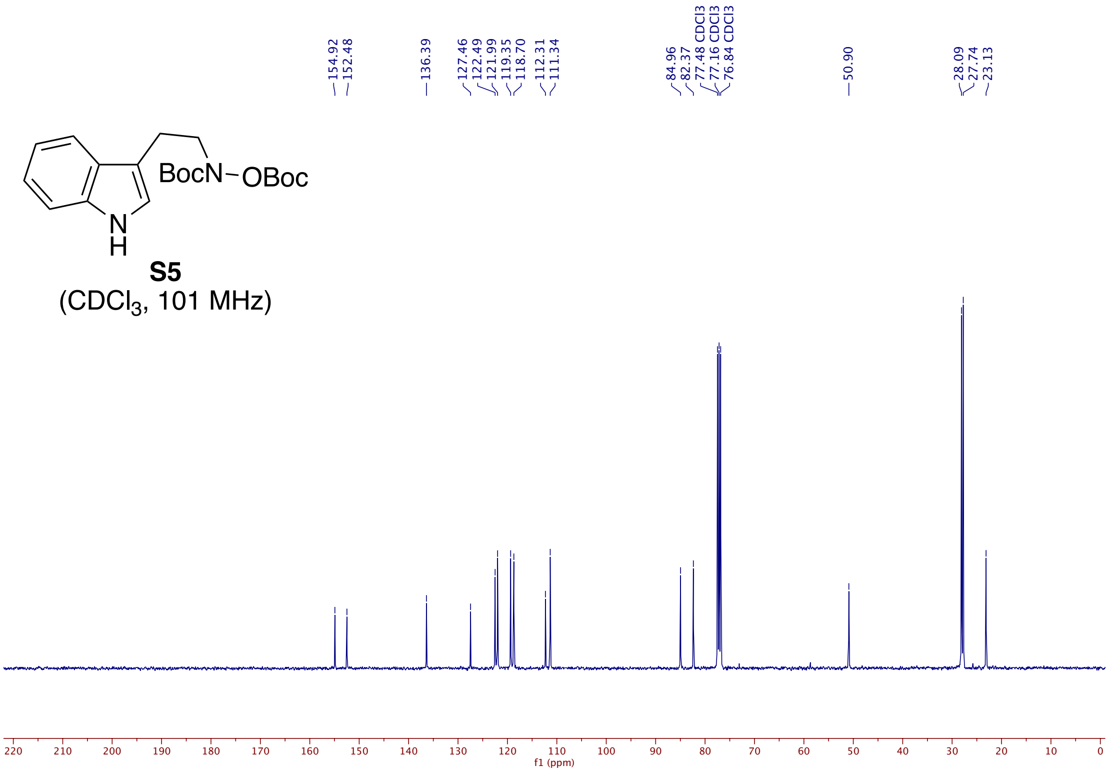

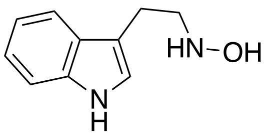

18
(DMSO-d_6, 500 MHz)

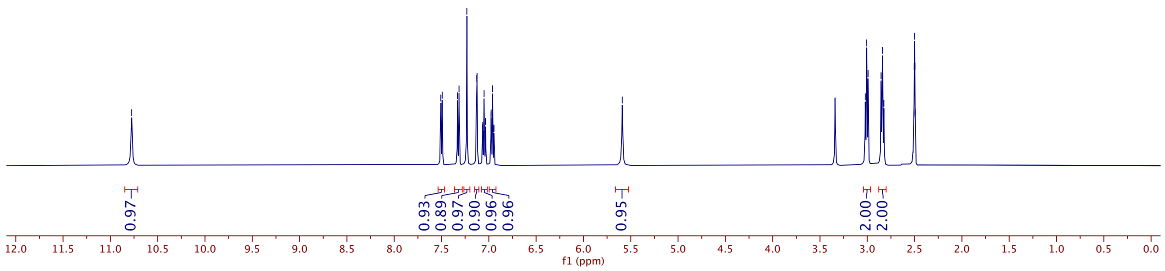

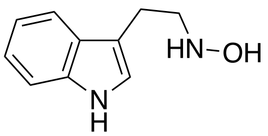

18
(DMSO-d_6, 101 MHz)

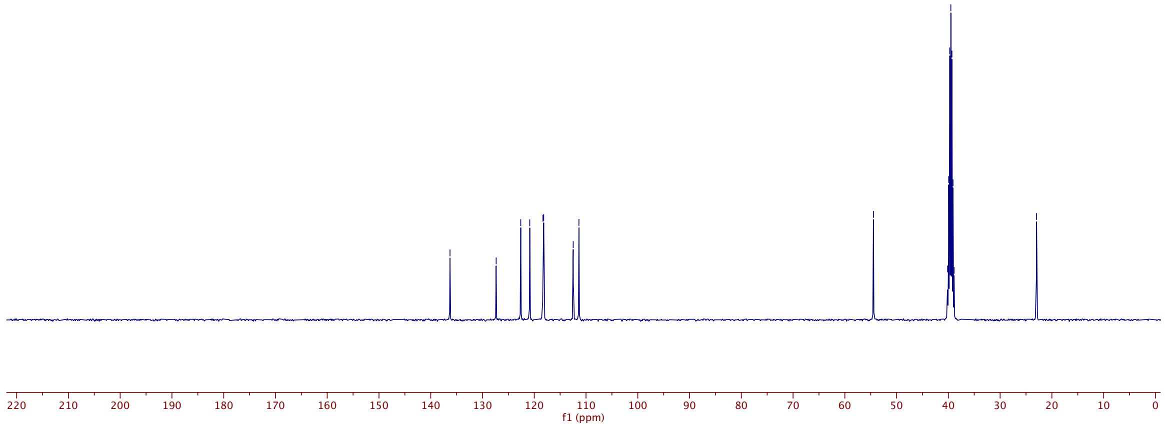

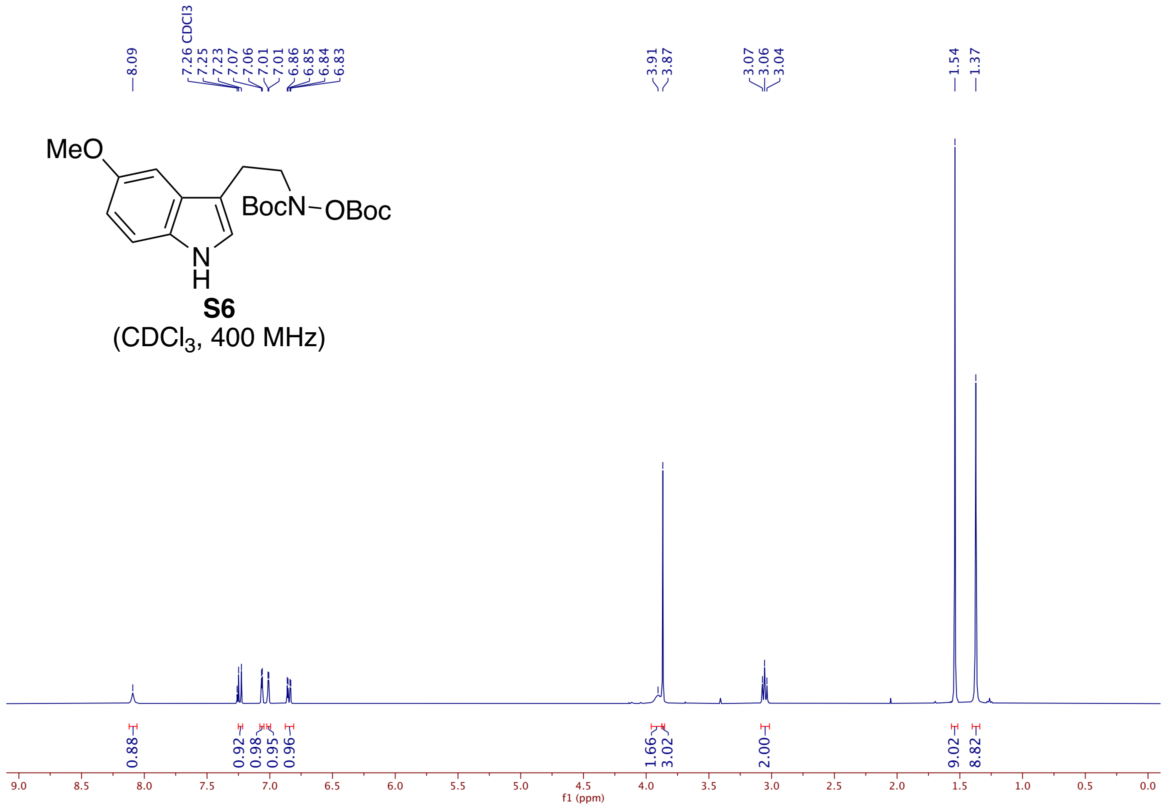
8.09
7.26 CDCl3
7.25
7.23
7.07
7.06
7.01
7.01
6.86
6.85
6.84
6.83
3.91
3.87
3.07
3.06
3.04
1.54
1.37
MeO
BocN
OBoc
N
H
S6
(CDCl3, 400 MHz)
0.88
0.92
0.98
0.95
0.96
1.66
3.02
2.00
9.02
8.82
f1 (ppm)

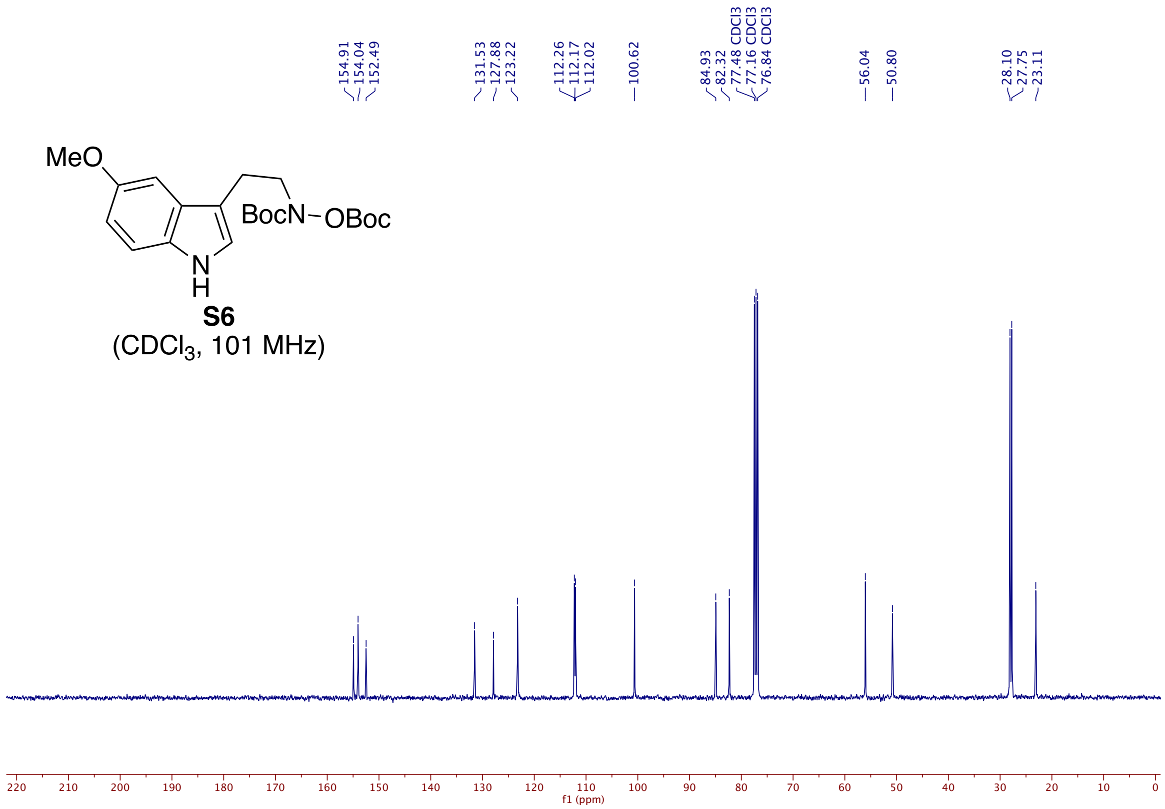
154.91
154.04
152.49
131.53
127.88
123.22
112.26
112.17
112.02
100.62
84.93
82.32
77.48 CDCl3
77.16 CDCl3
76.84 CDCl3
56.04
50.80
28.10
27.75
23.11
MeO
BocN
OBoc
N
H
S6
(CDCl3, 101 MHz)
f1 (ppm)

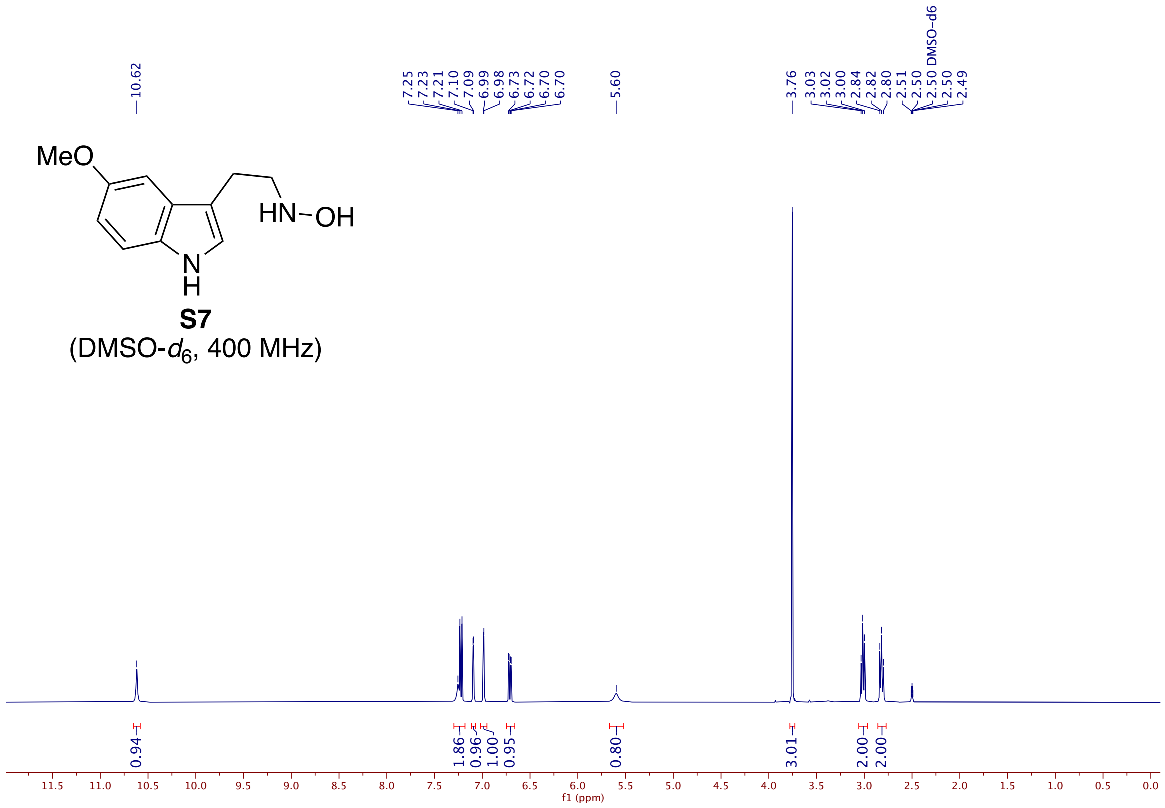
MeO
HN-OH
N
H
S7
(DMSO-d_6, 400 MHz)
f1 (ppm)

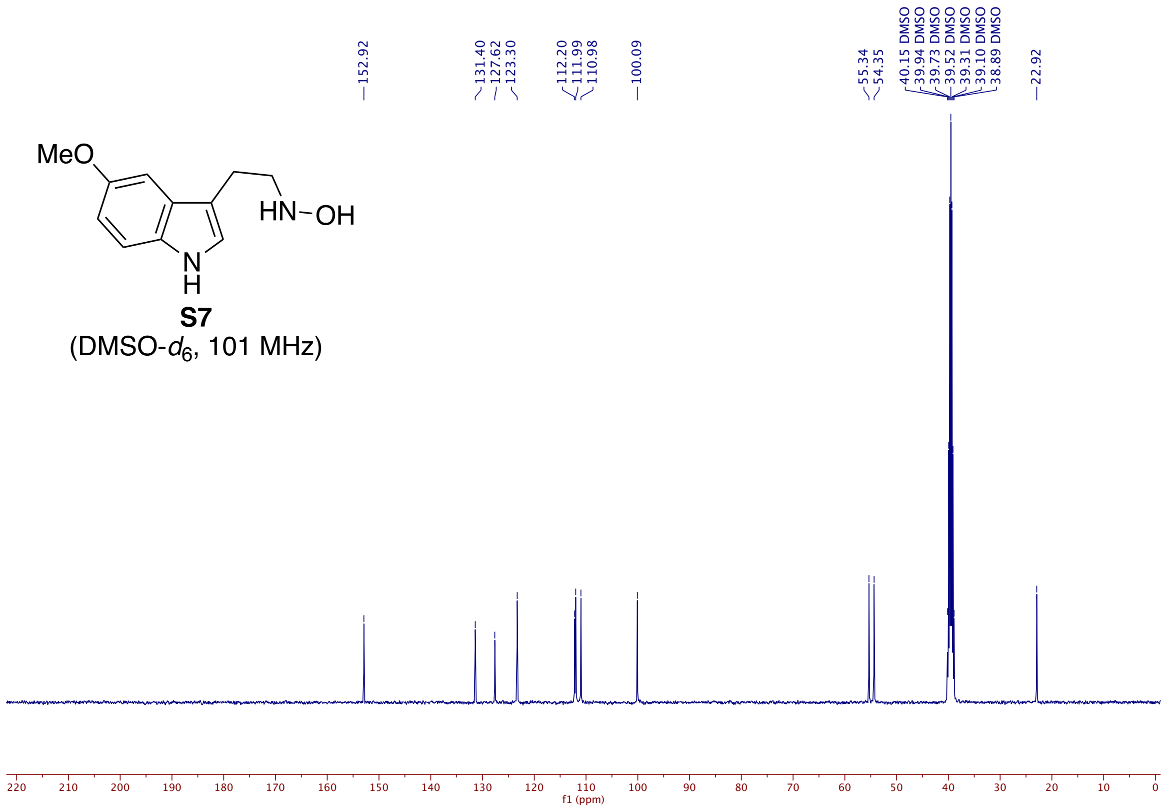
MeO
HN-OH
N
H
S7
(DMSO-d_6, 101 MHz)
f1 (ppm)

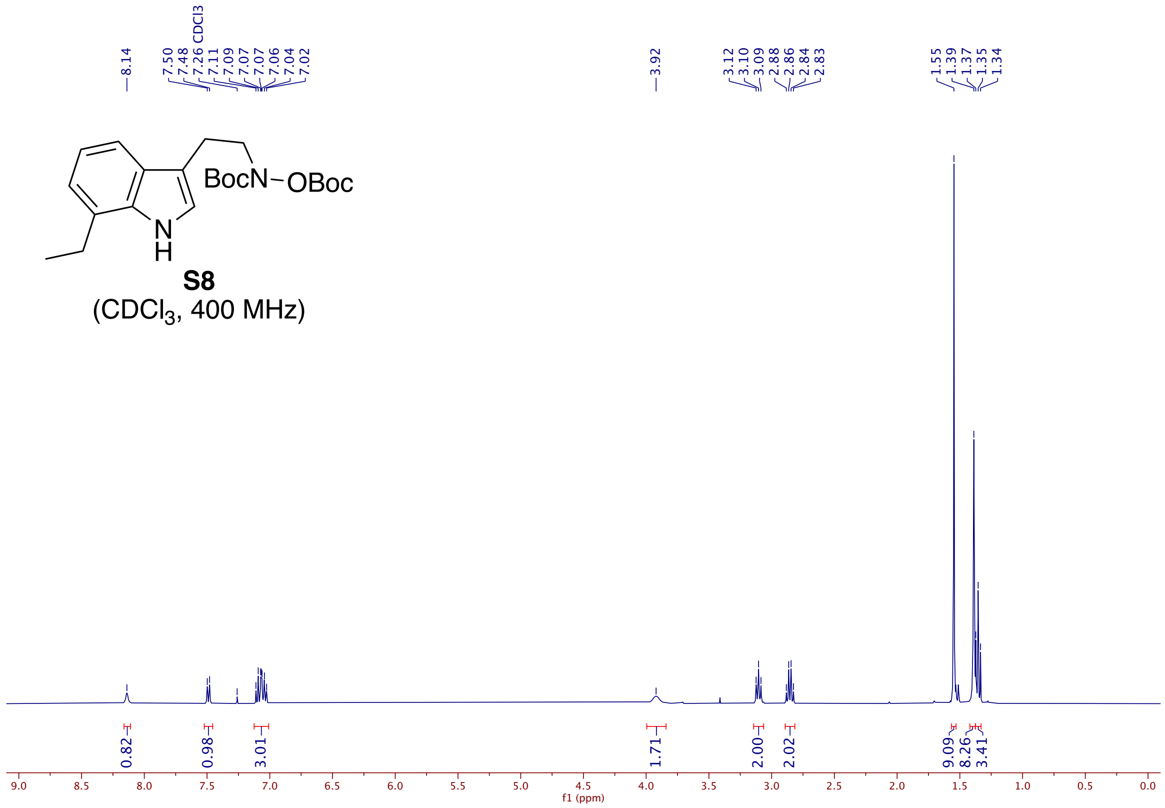
8.14
7.50
7.48
7.26 CDCl3
7.11
7.09
7.07
7.07
7.06
7.04
7.02
3.92
3.12
3.10
3.09
2.88
2.86
2.84
2.83
1.55
1.39
1.37
1.35
1.34
BocN–OBoc
N
H
S8
(CDCl3, 400 MHz)
0.82
0.98
3.01
1.71
2.00
2.02
9.09
8.26
3.41
f1 (ppm)

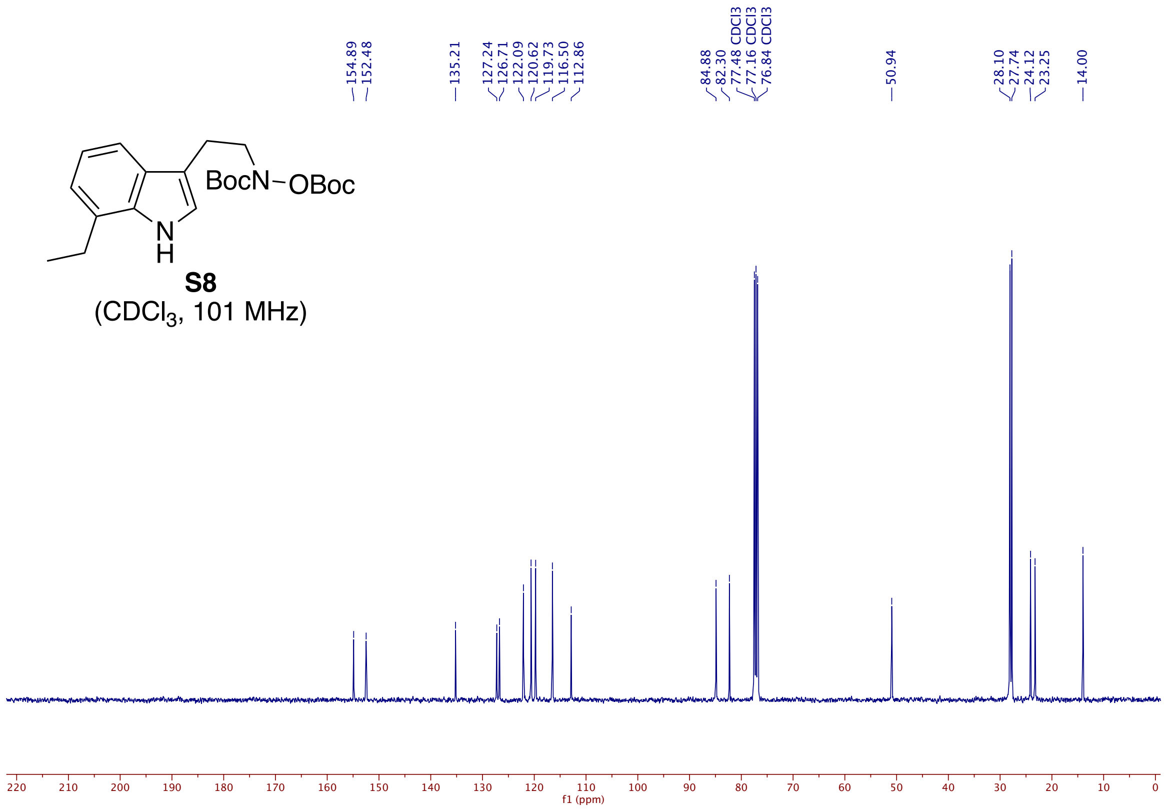
154.89
152.48
135.21
127.24
126.71
122.09
120.62
119.73
116.50
112.86
84.88
82.30
77.48 CDCl3
77.16 CDCl3
76.84 CDCl3
50.94
28.10
27.74
24.12
23.25
14.00
BocN–OBoc
N
H
S8
(CDCl3, 101 MHz)
f1 (ppm)

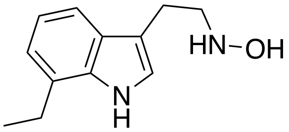

S9
(DMSO-d_6, 400 MHz)

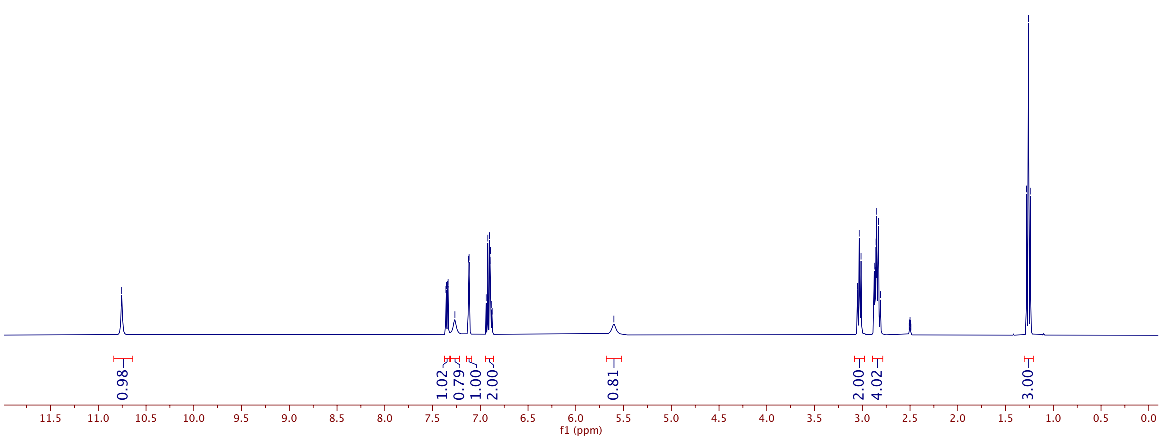

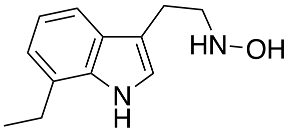

S9
(DMSO-d_6, 101 MHz)

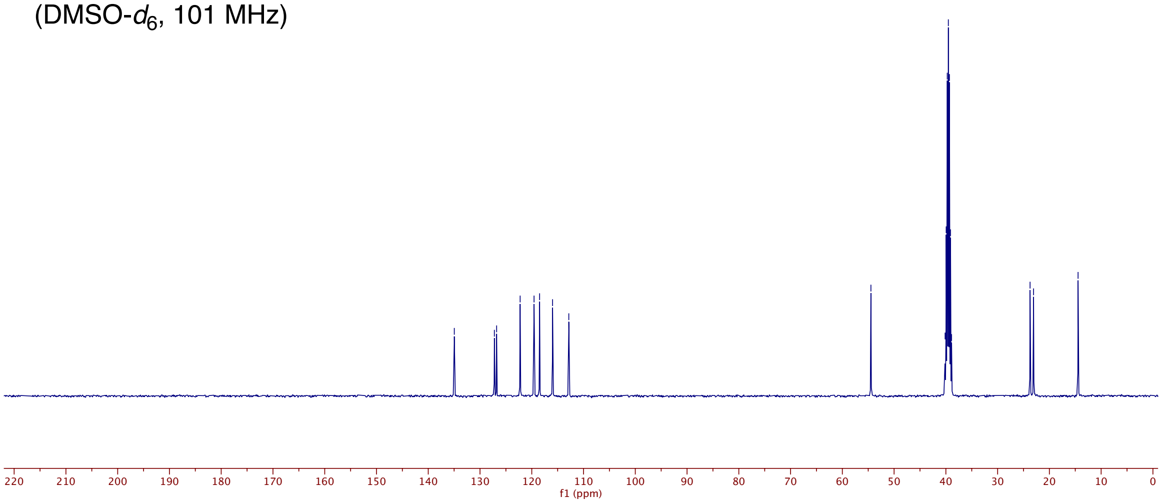

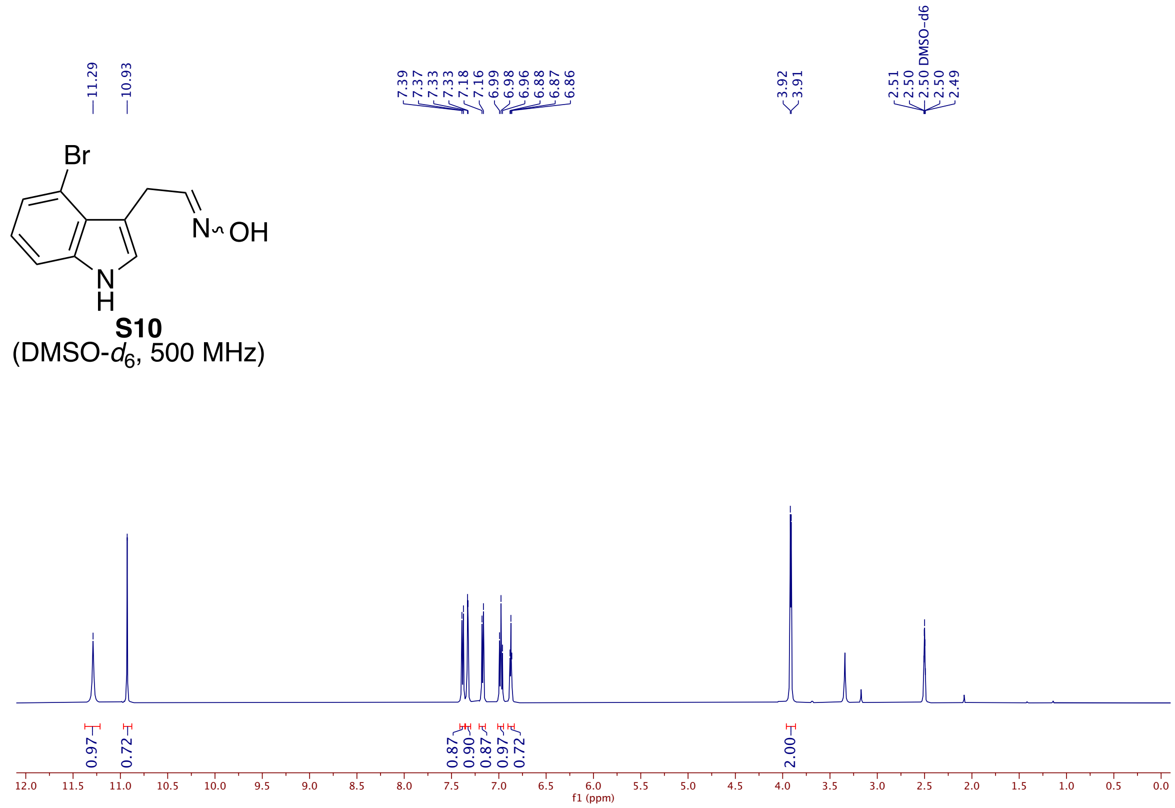
Br
N
H
OH
S10
(DMSO-d_6, 500 MHz)
11.29
10.93
7.39
7.37
7.33
7.33
7.18
7.16
6.99
6.98
6.96
6.88
6.87
6.86
3.92
3.91
2.51
2.50 DMSO-d6
2.50
2.49
0.97
0.72
0.87
0.90
0.87
0.97
0.72
2.00
f1 (ppm)

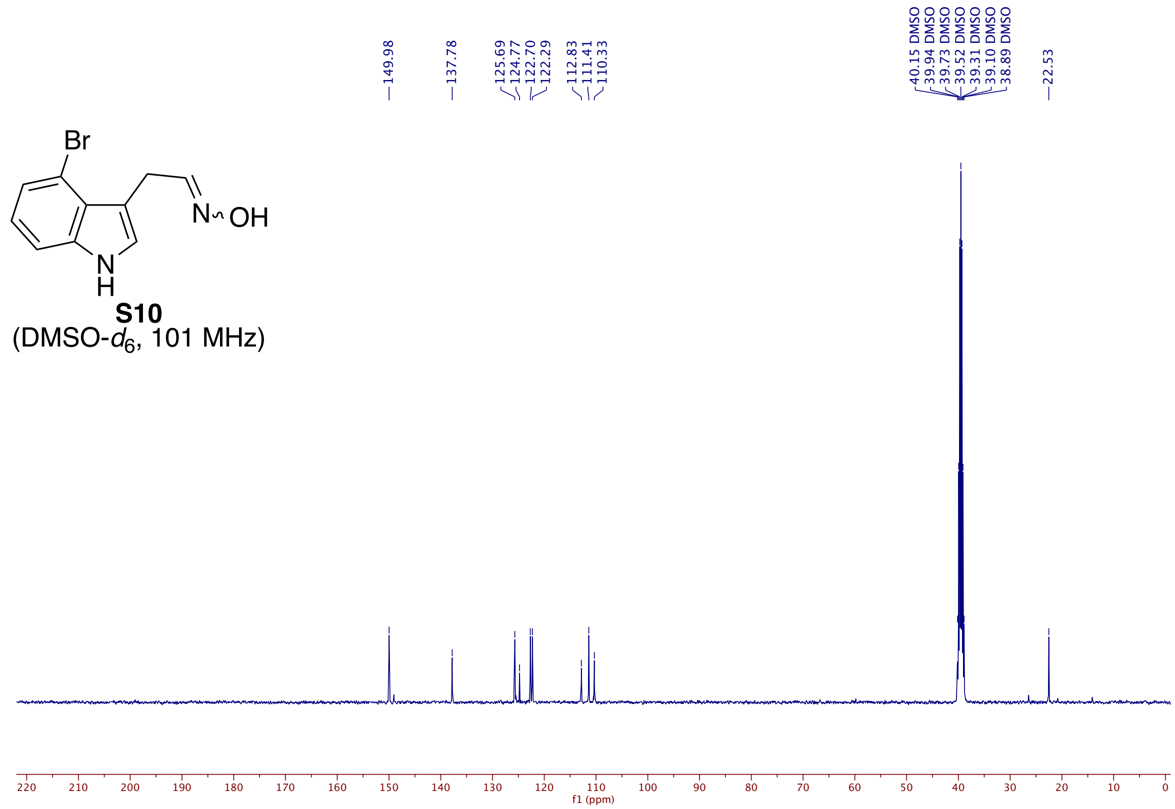
Br
N
H
OH
S10
(DMSO-d_6, 101 MHz)
149.98
137.78
125.69
124.77
122.70
122.29
112.83
111.41
110.33
40.15 DMSO
39.94 DMSO
39.73 DMSO
39.52 DMSO
39.31 DMSO
39.10 DMSO
38.89 DMSO
22.53
f1 (ppm)

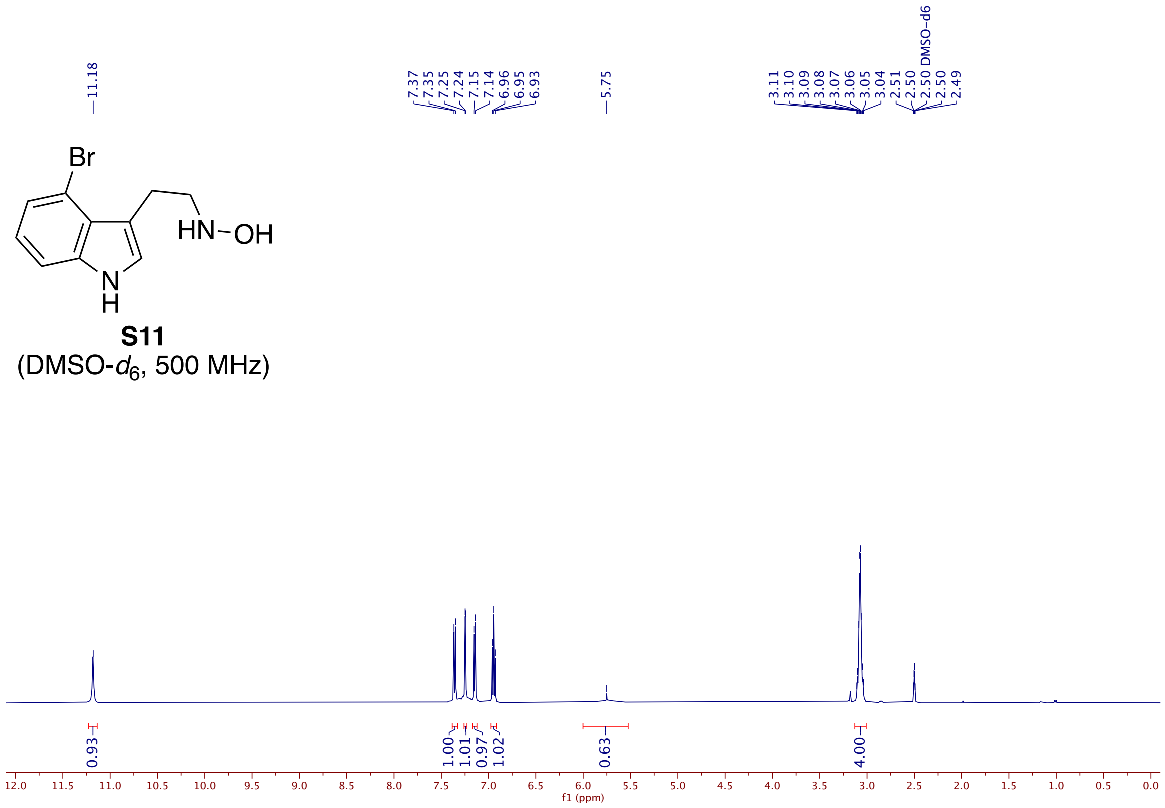
11.18
7.37
7.35
7.25
7.24
7.15
7.14
6.96
6.95
6.93
5.75
3.11
3.10
3.09
3.08
3.07
3.06
3.05
3.04
2.51
2.50 DMSO-d6
2.50
2.49
Br
HN–OH
N
H
S11
(DMSO-d_6, 500 MHz)
0.93
1.00
1.01
0.97
1.02
0.63
4.00
f1 (ppm)

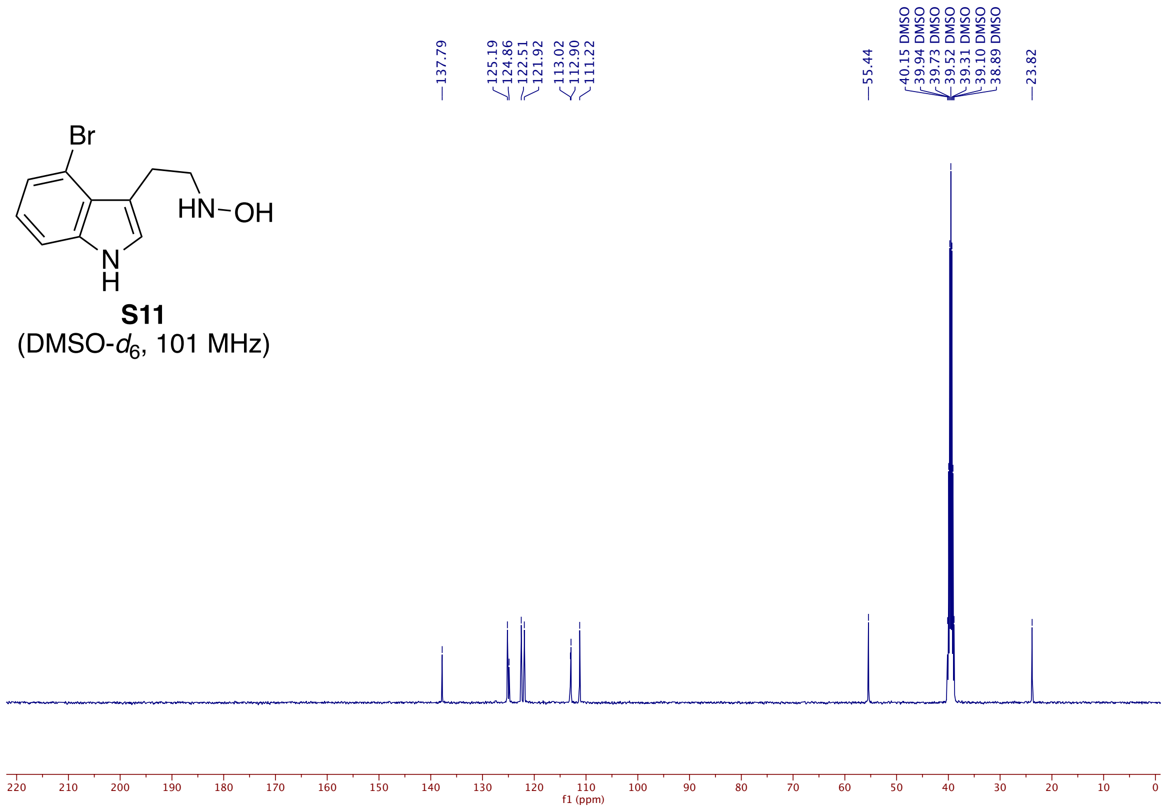
137.79
125.19
124.86
122.51
121.92
113.02
112.90
111.22
55.44
40.15 DMSO
39.94 DMSO
39.73 DMSO
39.52 DMSO
39.31 DMSO
39.10 DMSO
38.89 DMSO
23.82
Br
HN–OH
N
H
S11
(DMSO-d_6, 101 MHz)
f1 (ppm)

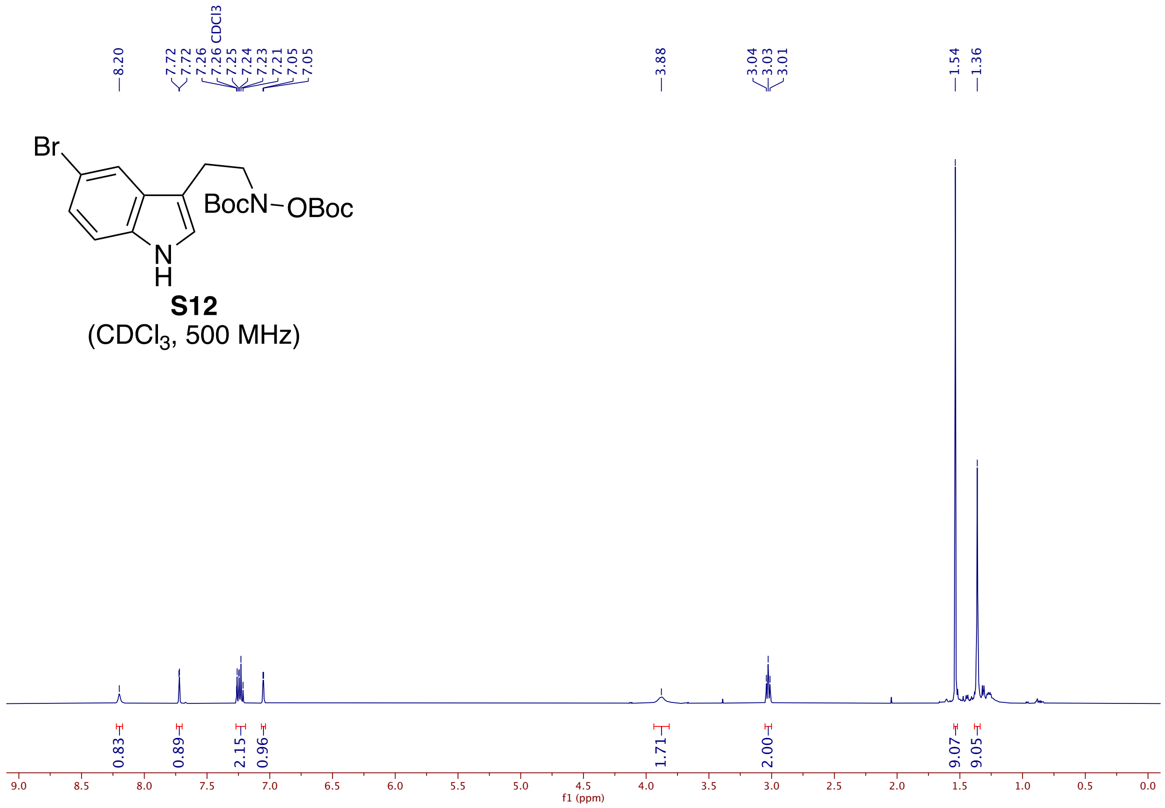

8.20
7.72
7.72
7.26 CDCl3
7.26
7.25
7.24
7.23
7.21
7.05
7.05
3.88
3.04
3.03
3.01
1.54
1.36
Br
BocN
OBoc
N
H
S12
(CDCl3, 500 MHz)
0.83
0.89
2.15
0.96
1.71
2.00
9.07
9.05
f1 (ppm)

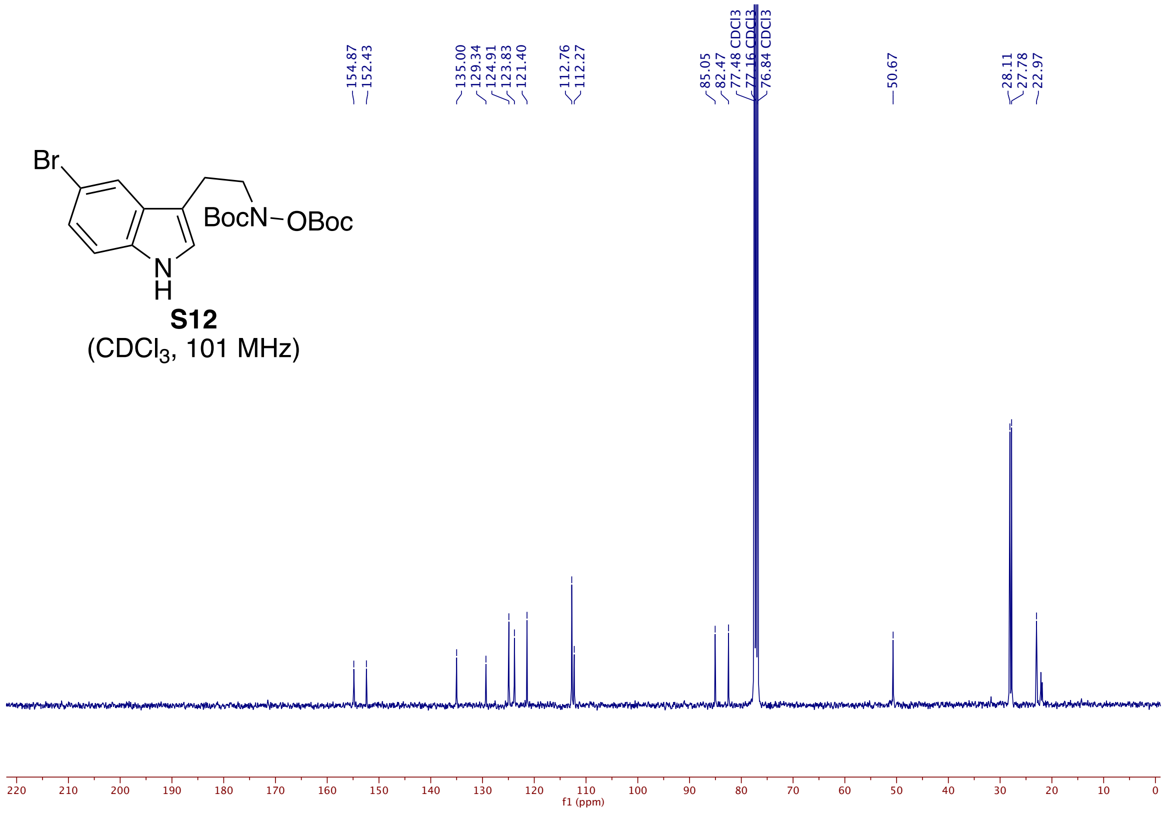

154.87
152.43
135.00
129.34
124.91
123.83
121.40
112.76
112.27
85.05
82.47
77.48 CDCl3
77.16 CDCl3
76.84 CDCl3
50.67
28.11
27.78
22.97
Br
BocN
OBoc
N
H
S12
(CDCl3, 101 MHz)
f1 (ppm)

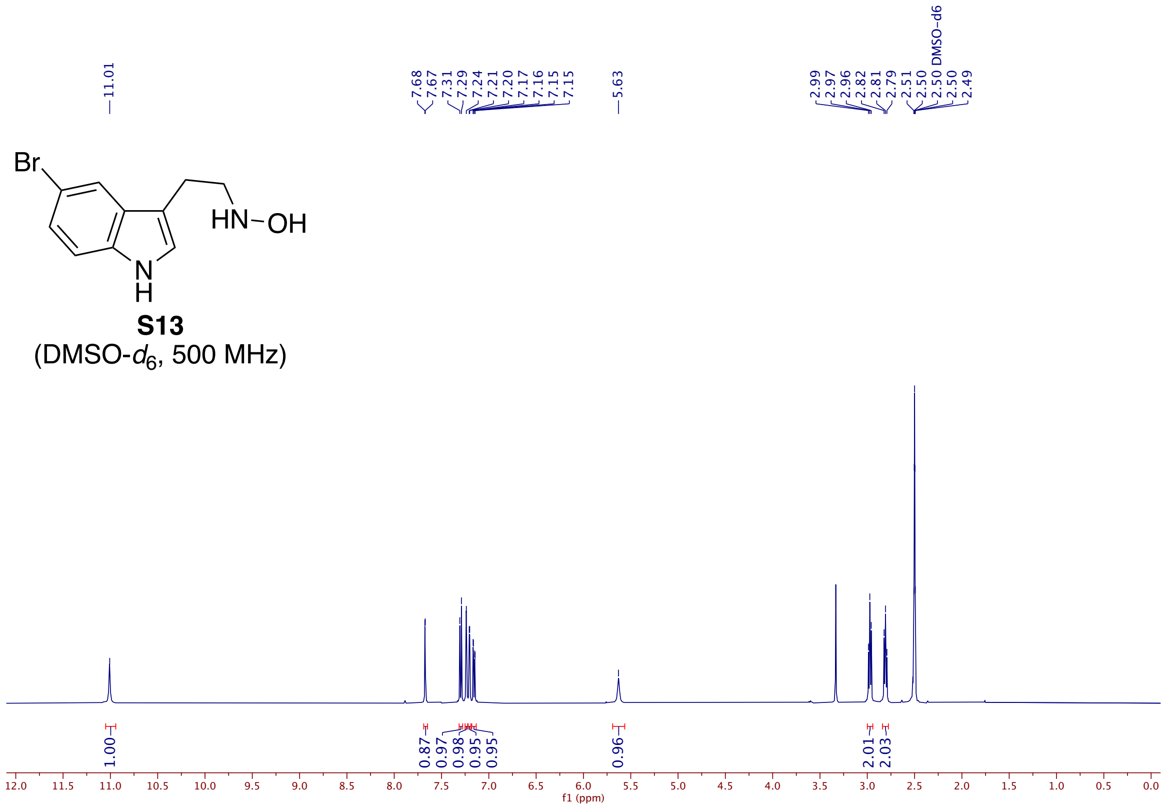

S13
(DMSO-d_6, 500 MHz)

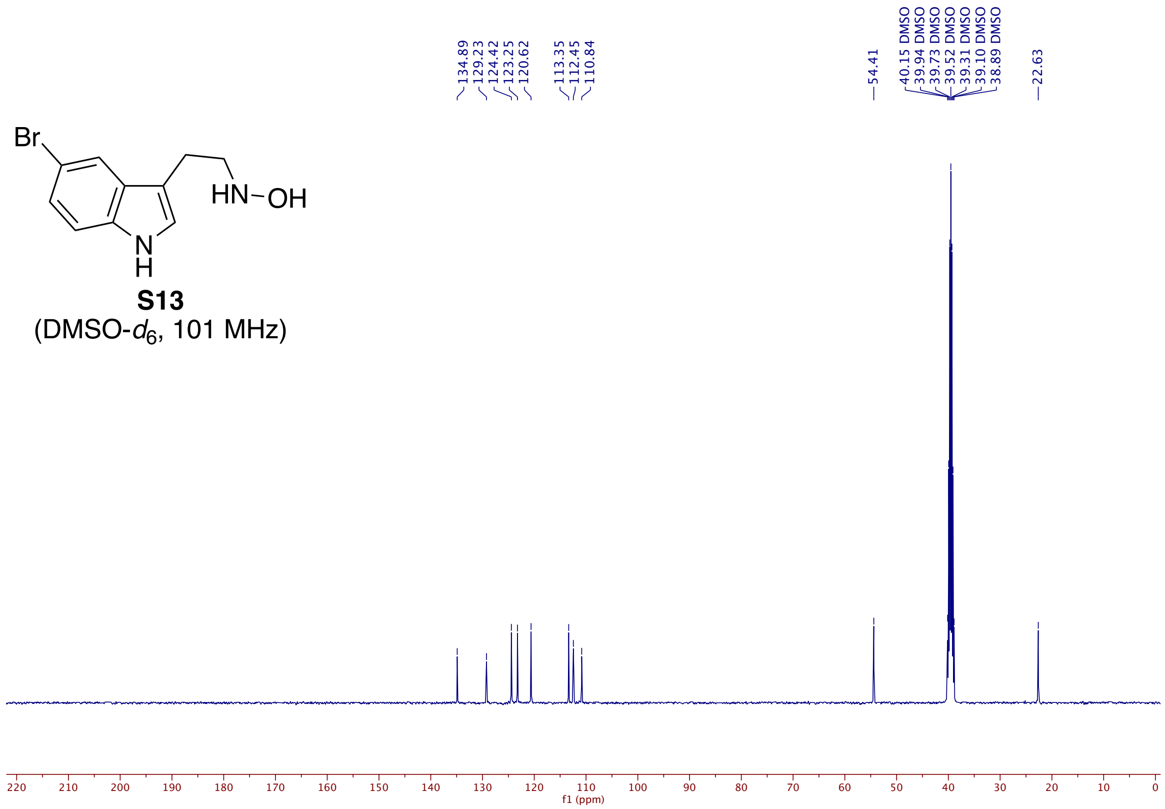

S13
(DMSO-d_6, 101 MHz)

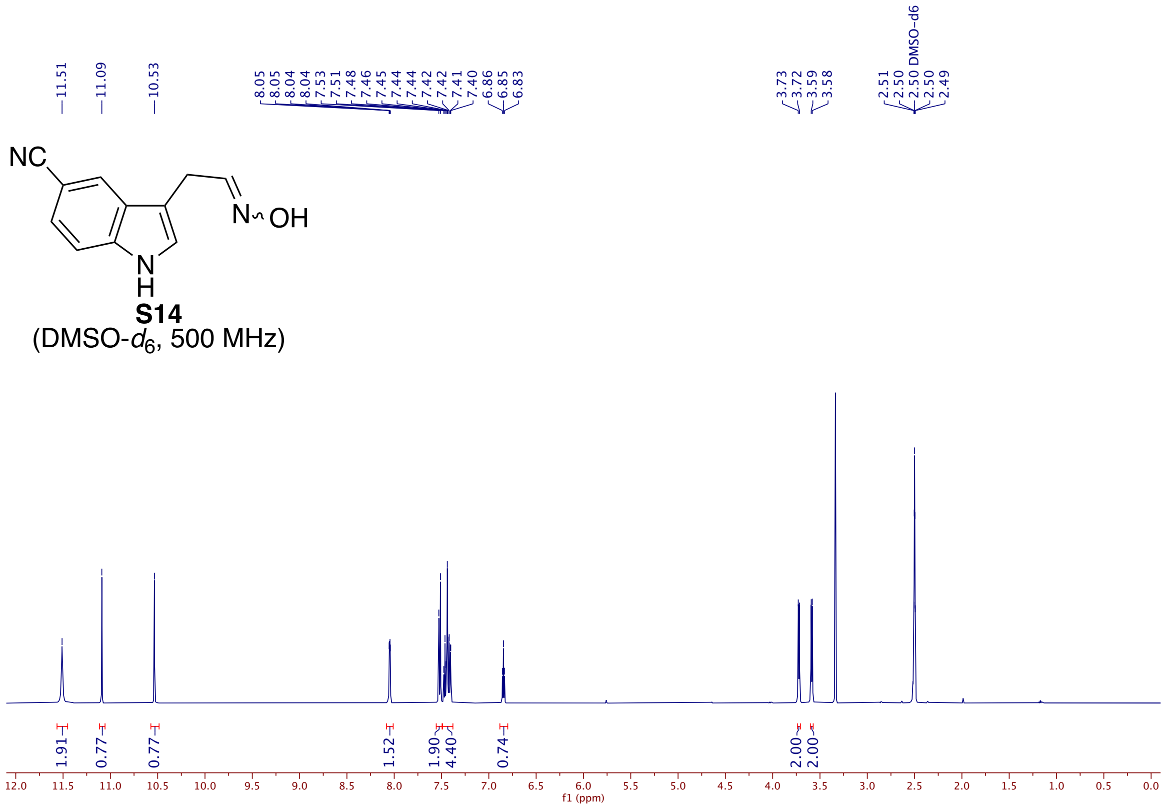
NC
N
OH
N
H
S14
(DMSO-d6, 500 MHz)
11.51
11.09
10.53
8.05
8.05
8.04
8.04
7.53
7.51
7.48
7.46
7.45
7.44
7.44
7.42
7.42
7.41
7.40
6.86
6.85
6.83
3.73
3.72
3.59
3.58
2.51
2.50 DMSO-d6
2.50
2.49
1.91
0.77
0.77
1.52
1.90
4.40
0.74
2.00
2.00
f1 (ppm)

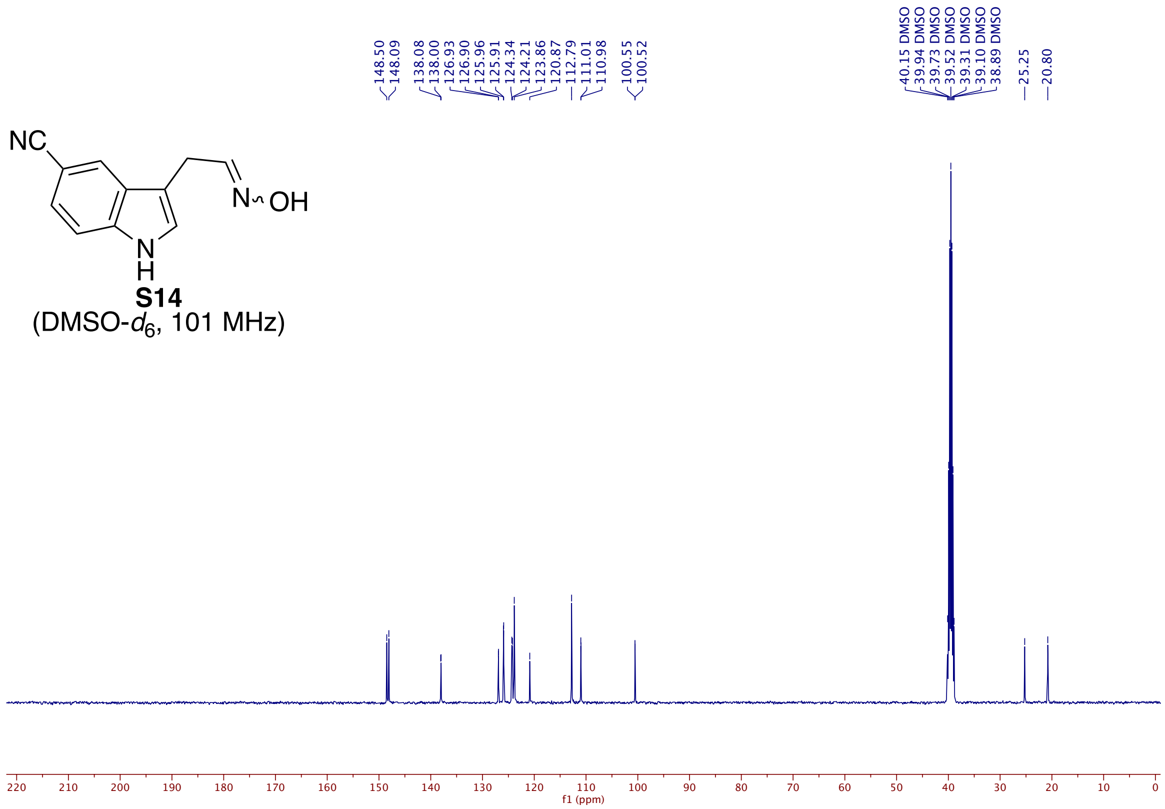
NC
N
OH
N
H
S14
(DMSO-d6, 101 MHz)
148.50
148.09
138.08
138.00
126.93
126.90
125.96
125.91
124.34
124.21
123.86
120.87
112.79
111.01
110.98
100.55
100.52
40.15 DMSO
39.94 DMSO
39.73 DMSO
39.52 DMSO
39.31 DMSO
39.10 DMSO
38.89 DMSO
25.25
20.80
f1 (ppm)

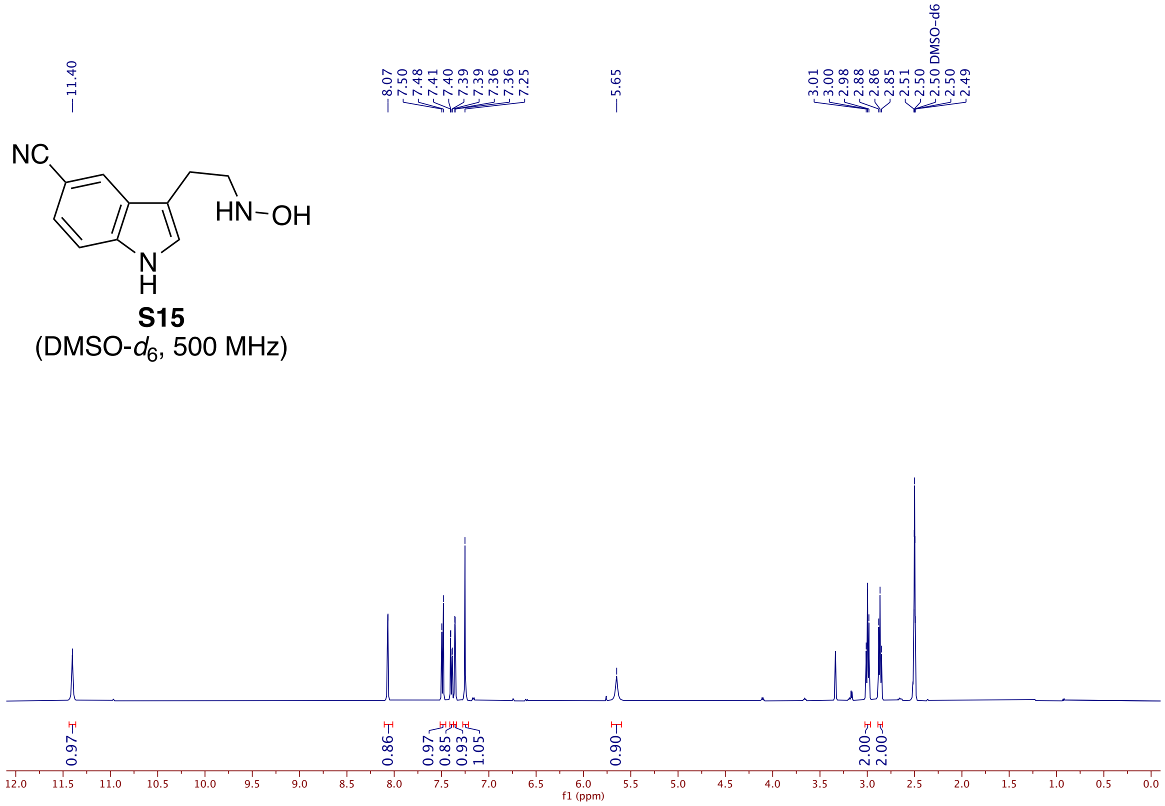
11.40
8.07
7.50
7.48
7.41
7.40
7.39
7.39
7.36
7.36
7.25
5.65
3.01
3.00
2.98
2.88
2.86
2.85
2.51
2.50 DMSO-d6
2.50
2.50
2.49
NC
HN-OH
N
H
S15
(DMSO-d6, 500 MHz)
0.97
0.86
0.97
0.85
0.93
1.05
0.90
2.00
2.00
f1 (ppm)

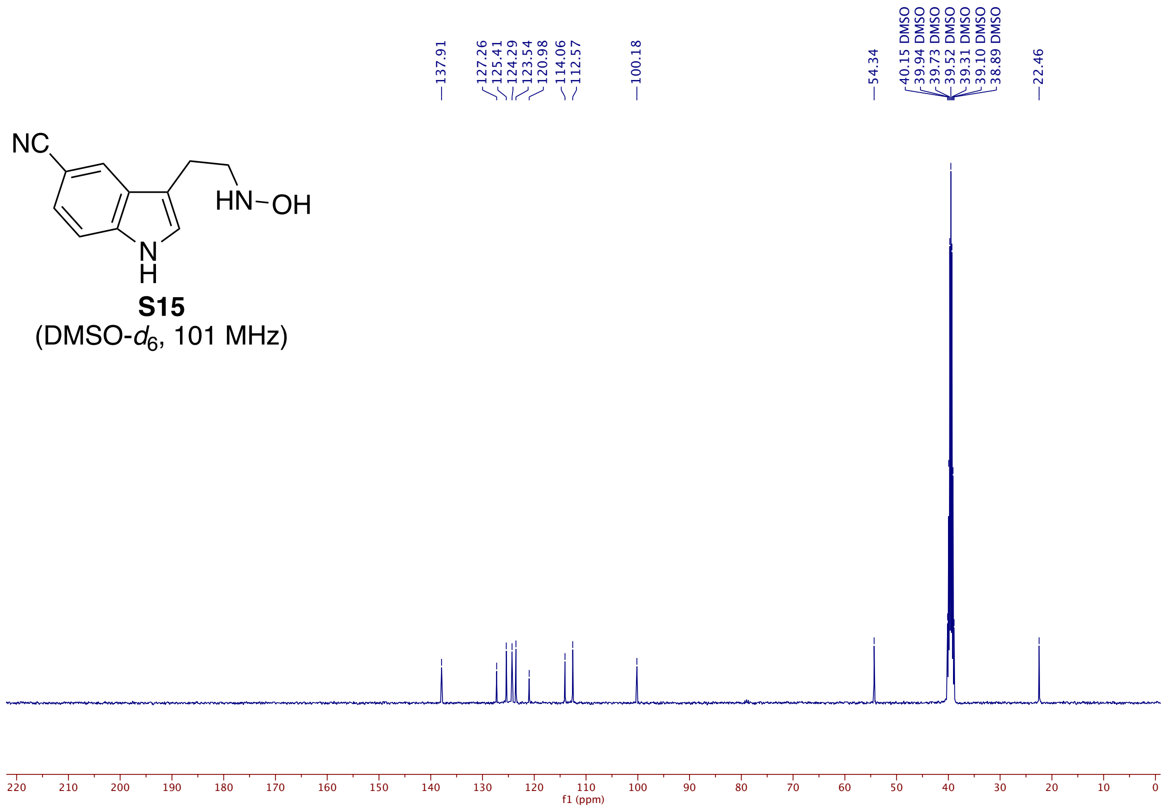
137.91
127.26
125.41
124.29
123.54
120.98
114.06
112.57
100.18
54.34
40.15 DMSO
39.94 DMSO
39.73 DMSO
39.52 DMSO
39.31 DMSO
39.10 DMSO
38.89 DMSO
22.46
NC
HN-OH
N
H
S15
(DMSO-d6, 101 MHz)
f1 (ppm)

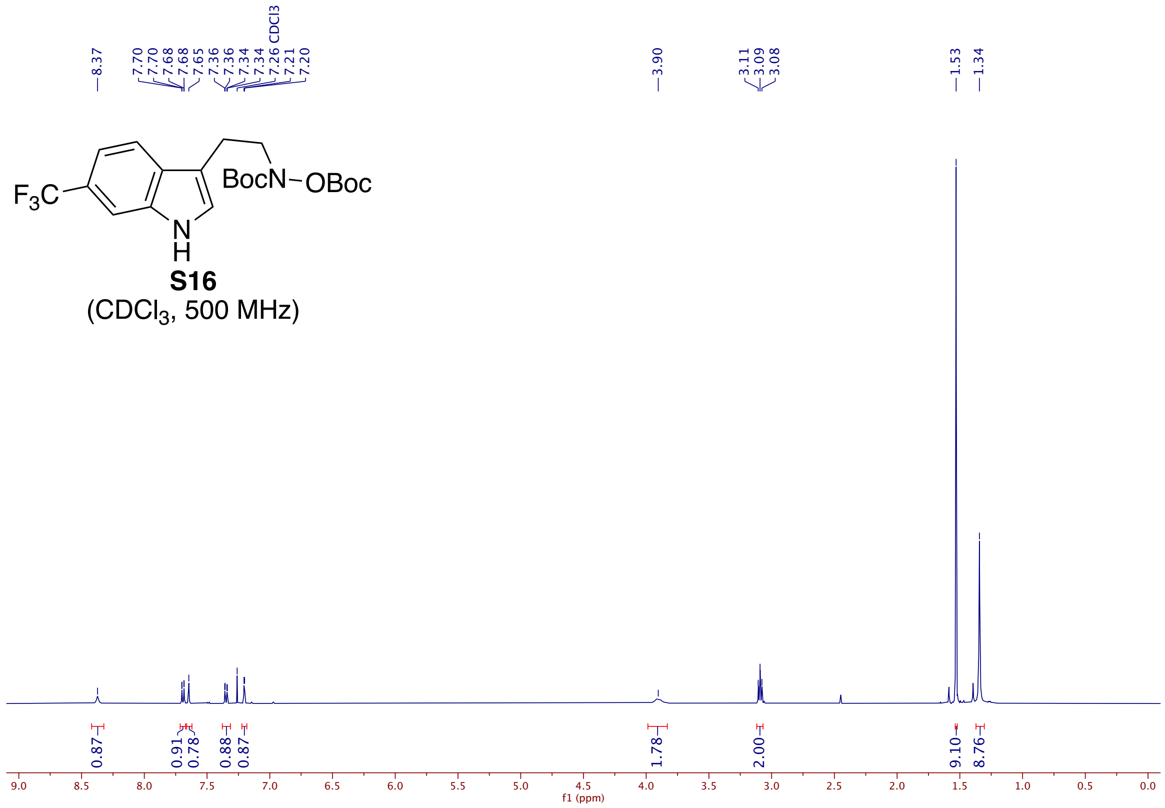
8.37
7.70
7.70
7.68
7.68
7.65
7.36
7.36
7.34
7.34
7.26 CDCl3
7.21
7.20
3.90
3.11
3.09
3.08
1.53
1.34
F3C
BocN
OBoc
N
H
S16
(CDCl3, 500 MHz)
0.87
0.91
0.78
0.88
0.87
1.78
2.00
9.10
8.76
f1 (ppm)

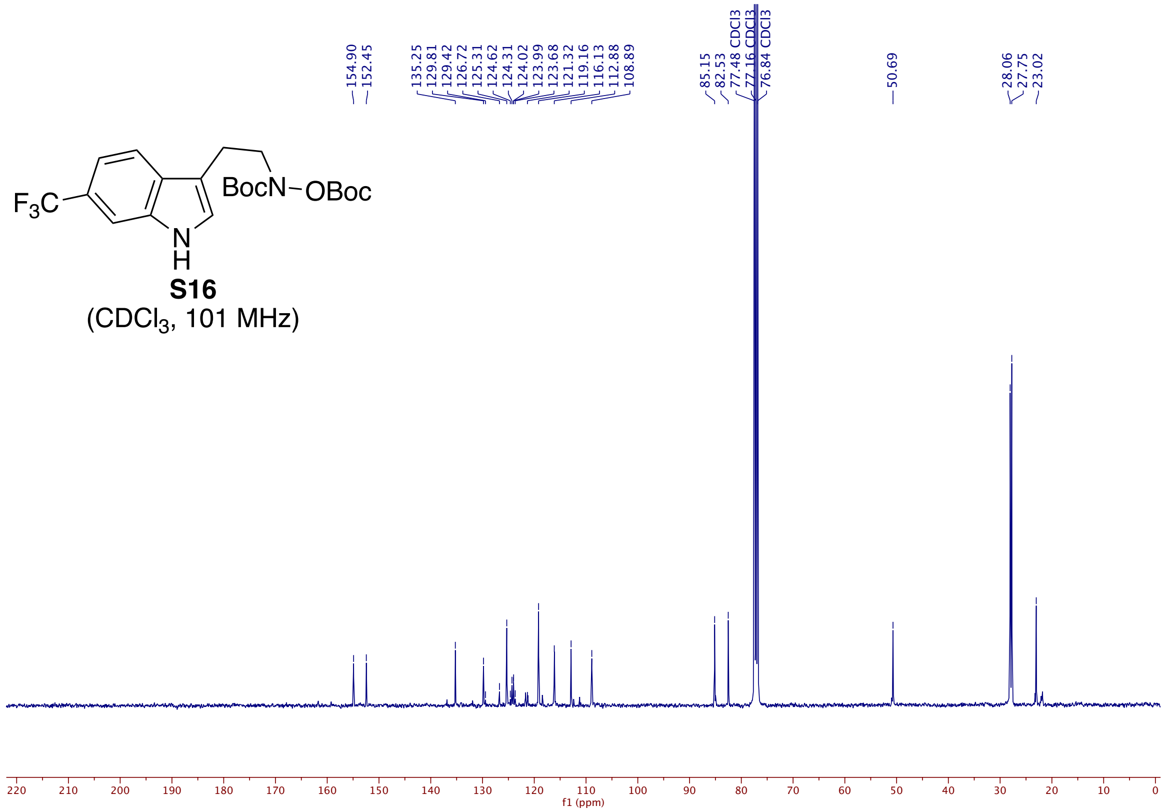
154.90
152.45
135.25
129.81
129.42
126.72
125.31
124.62
124.31
124.02
123.99
123.68
121.32
119.16
116.13
112.88
108.89
85.15
82.53
77.48 CDCl3
77.16 CDCl3
76.84 CDCl3
50.69
28.06
27.75
23.02
F3C
BocN
OBoc
N
H
S16
(CDCl3, 101 MHz)
f1 (ppm)

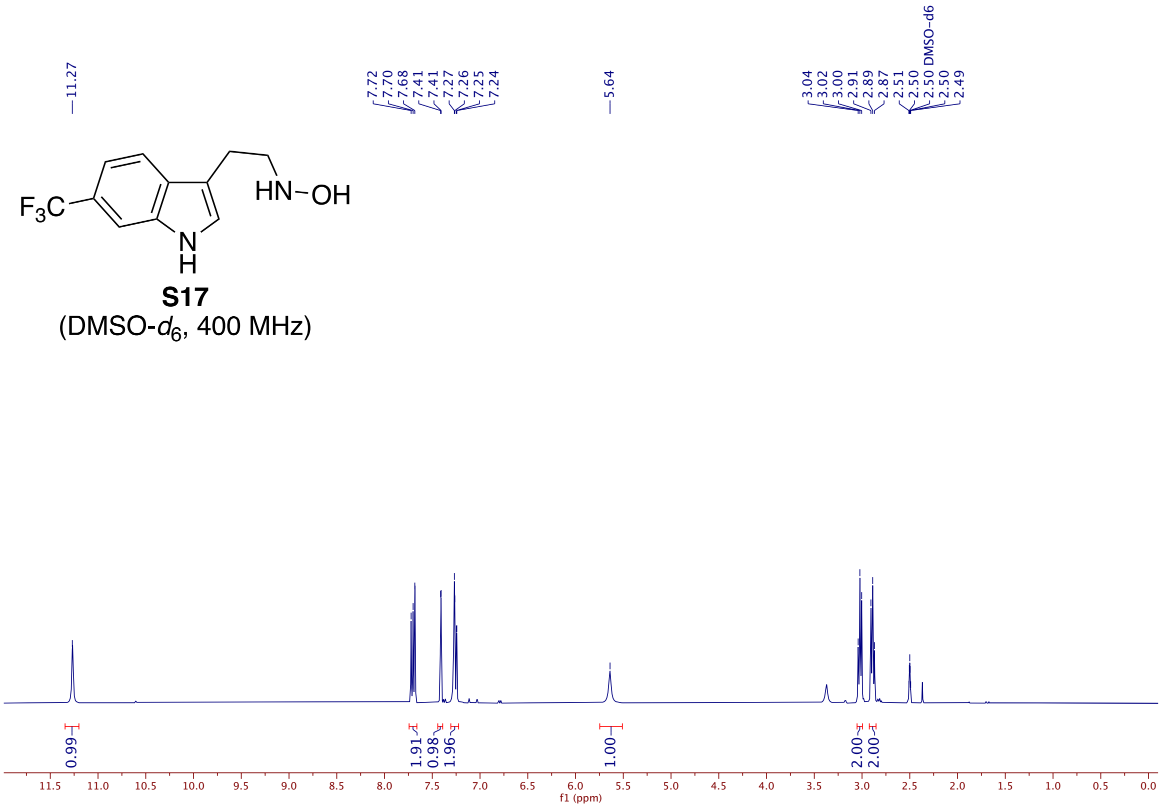

S17
(DMSO-d_6, 400 MHz)

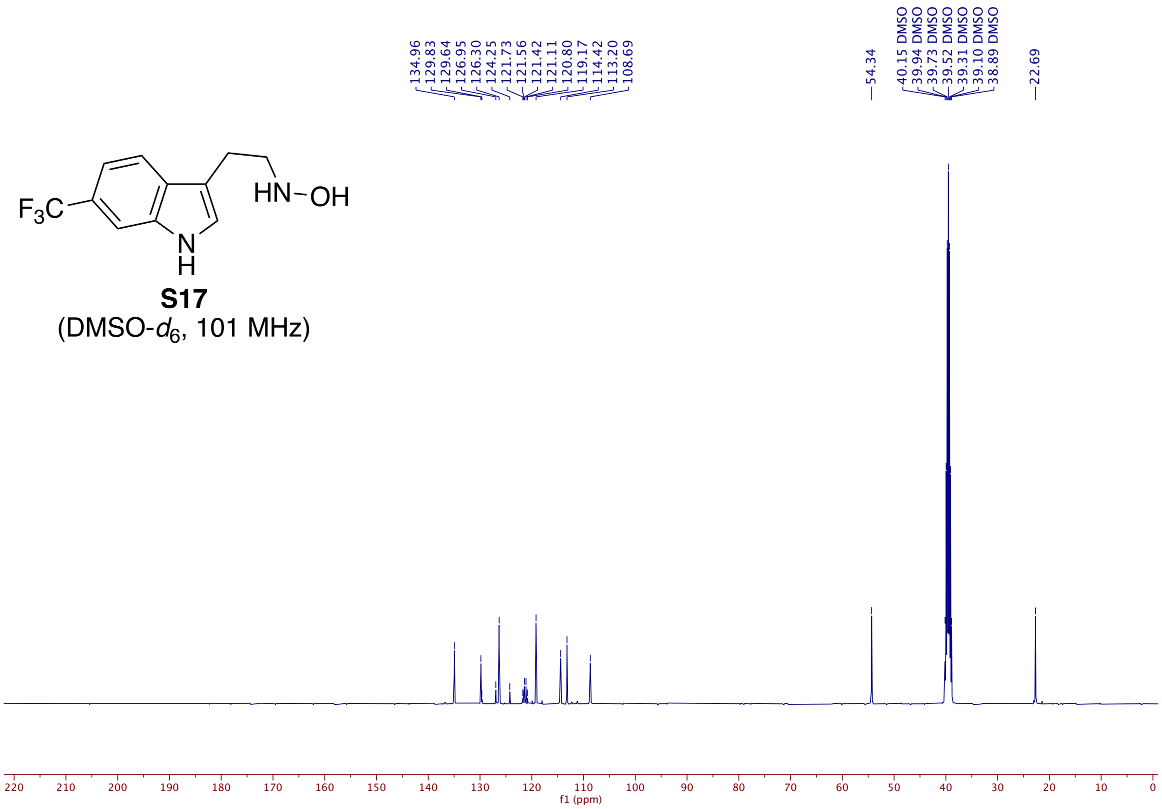

S17
(DMSO-d_6, 101 MHz)

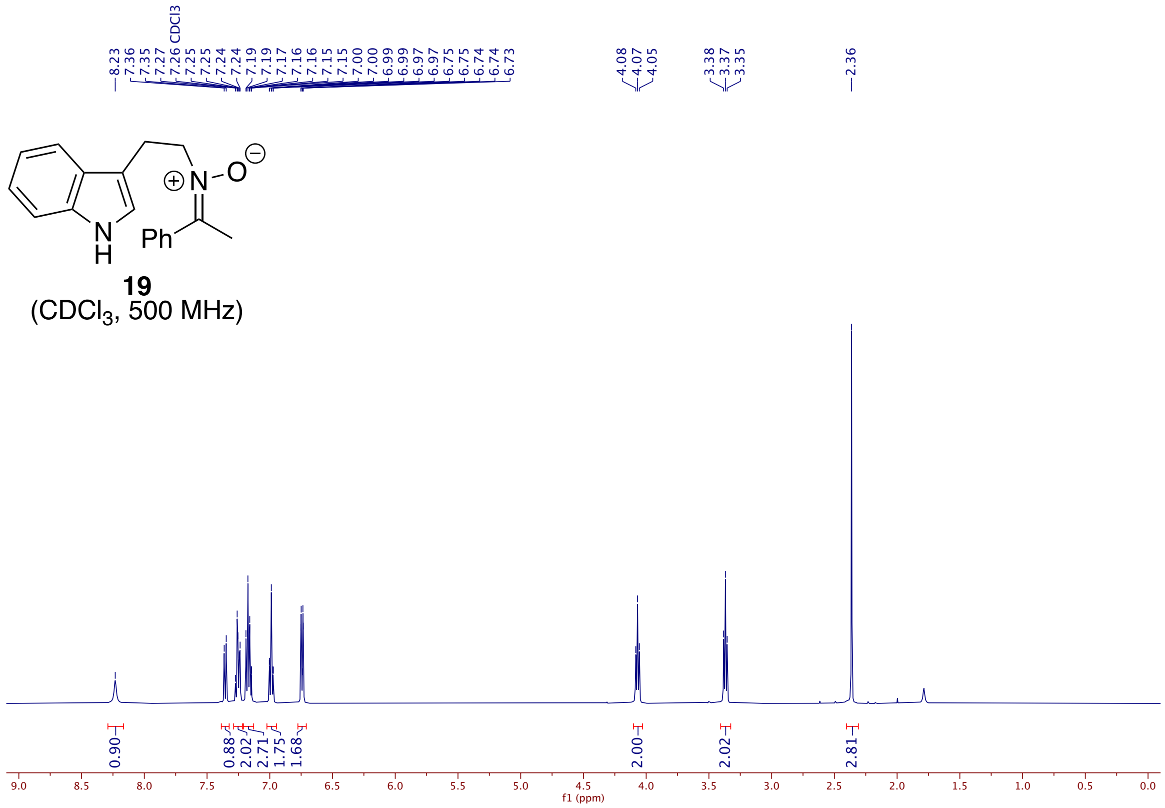
8.23
7.36
7.35
7.27
7.26 CDCl3
7.25
7.25
7.24
7.24
7.19
7.19
7.17
7.16
7.16
7.15
7.15
7.00
6.99
6.99
6.97
6.97
6.75
6.75
6.74
6.74
6.73
4.08
4.07
4.05
3.38
3.37
3.35
2.36
N
O
Ph
N
H
19
(CDCl3, 500 MHz)
0.90
0.88
2.02
2.71
1.75
1.68
2.00
2.02
2.81
f1 (ppm)

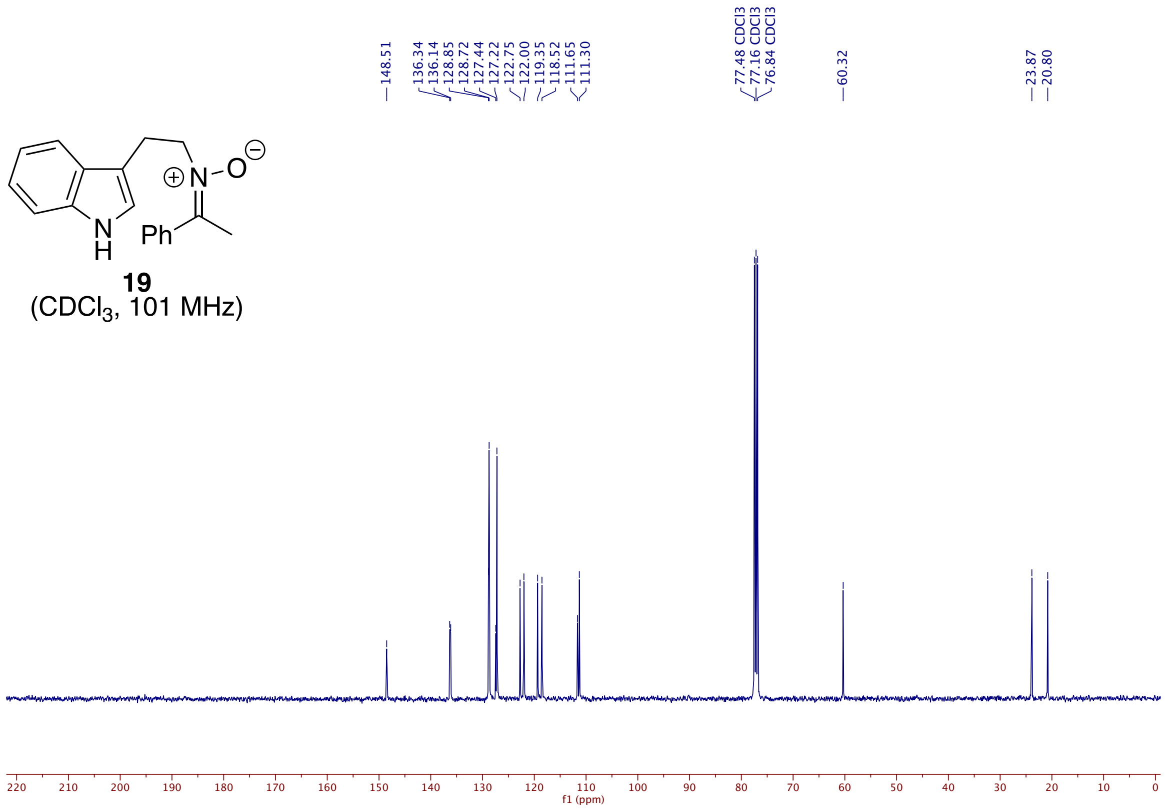
148.51
136.34
136.14
128.85
128.72
127.44
127.22
122.75
122.00
119.35
118.52
111.65
111.30
77.48 CDCl3
77.16 CDCl3
76.84 CDCl3
60.32
23.87
20.80
N
O
Ph
N
H
19
(CDCl3, 101 MHz)
f1 (ppm)

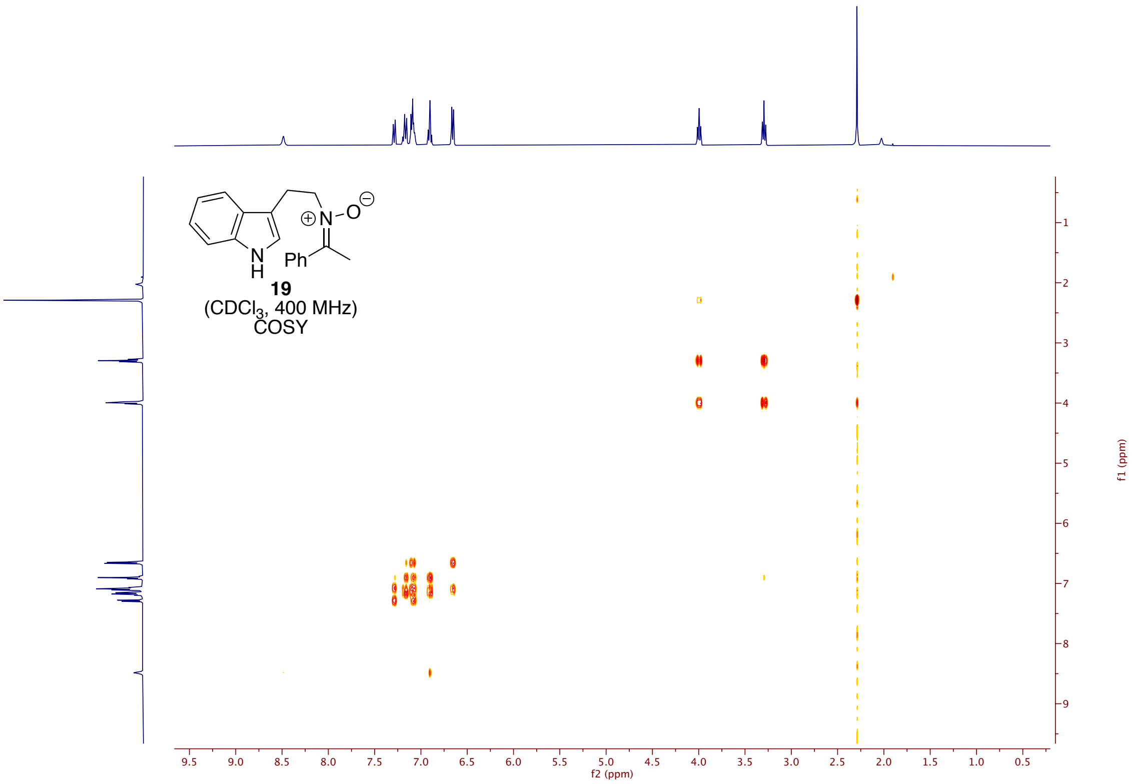
19
(CDCl3, 400 MHz)
COSY
f1 (ppm)
f2 (ppm)

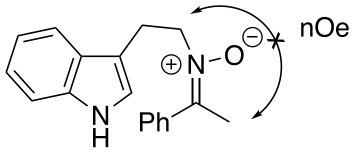
nOe

19

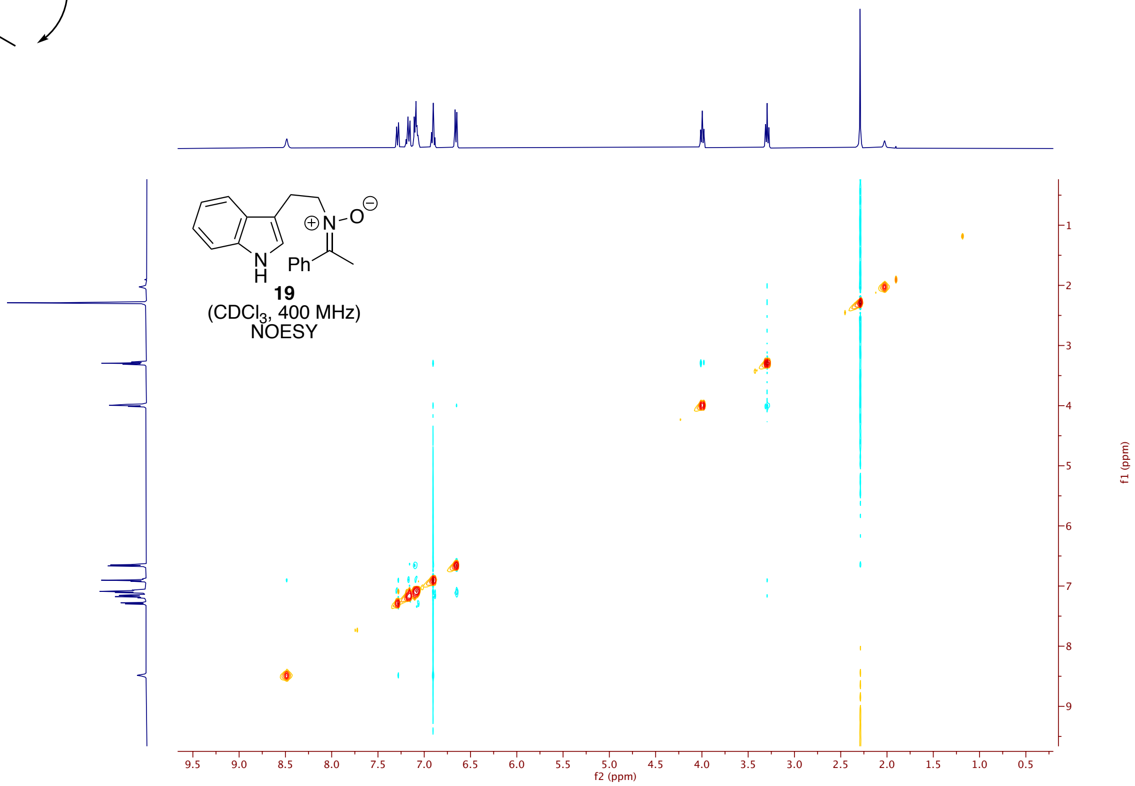
19
(CDCl3, 400 MHz)
NOESY
f1 (ppm)
f2 (ppm)

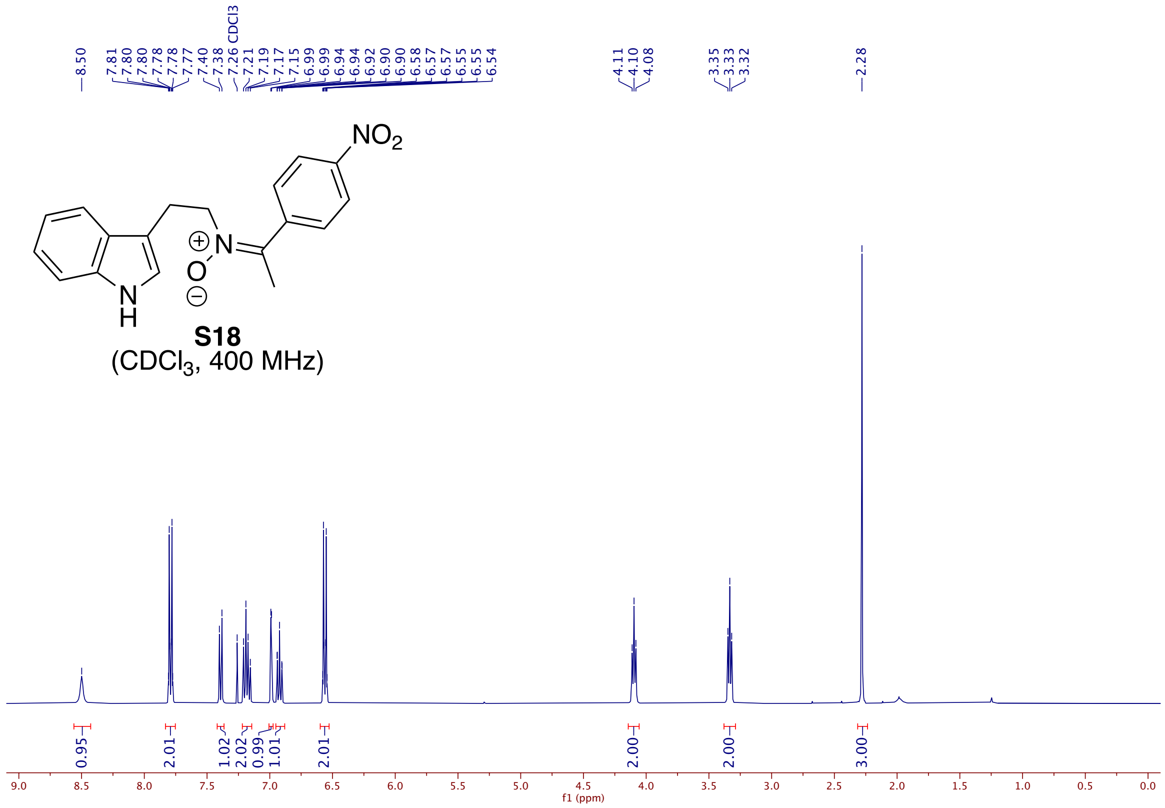

S18
(CDCl3, 400 MHz)
f1 (ppm)

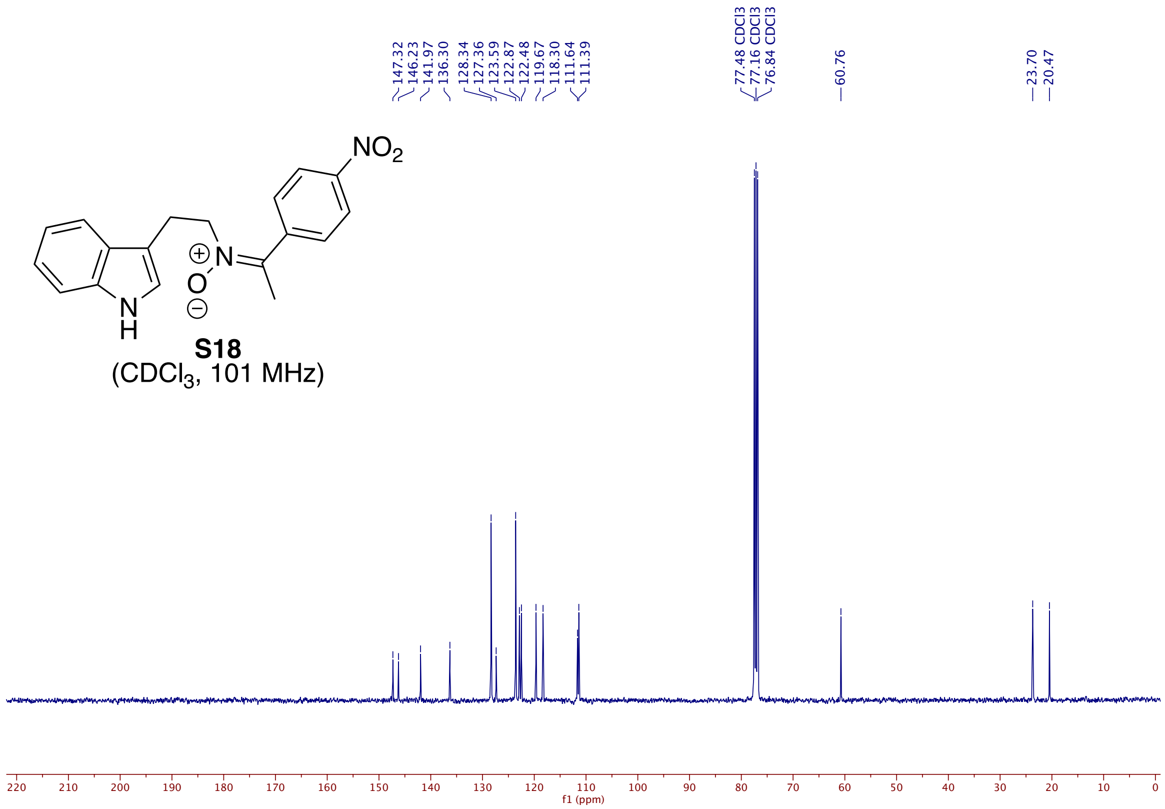

S18
(CDCl3, 101 MHz)
f1 (ppm)

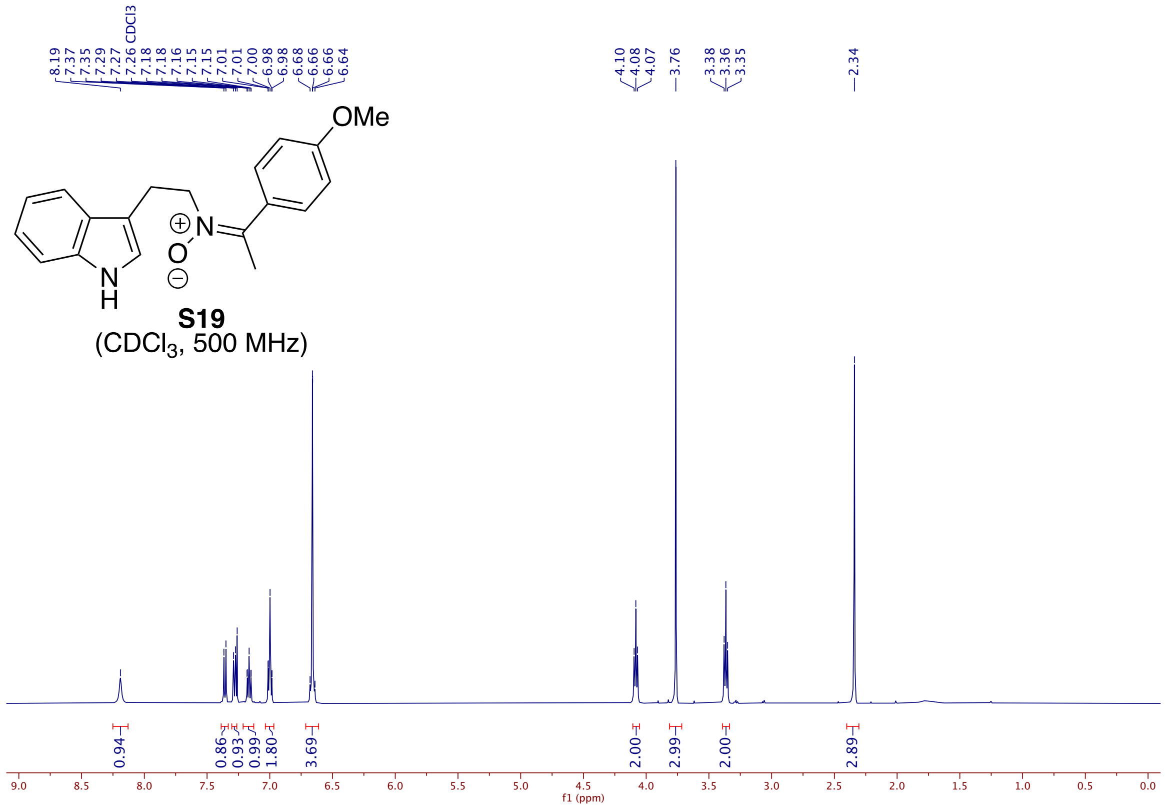
OMe
N
O
N
H
S19
(CDCl3, 500 MHz)

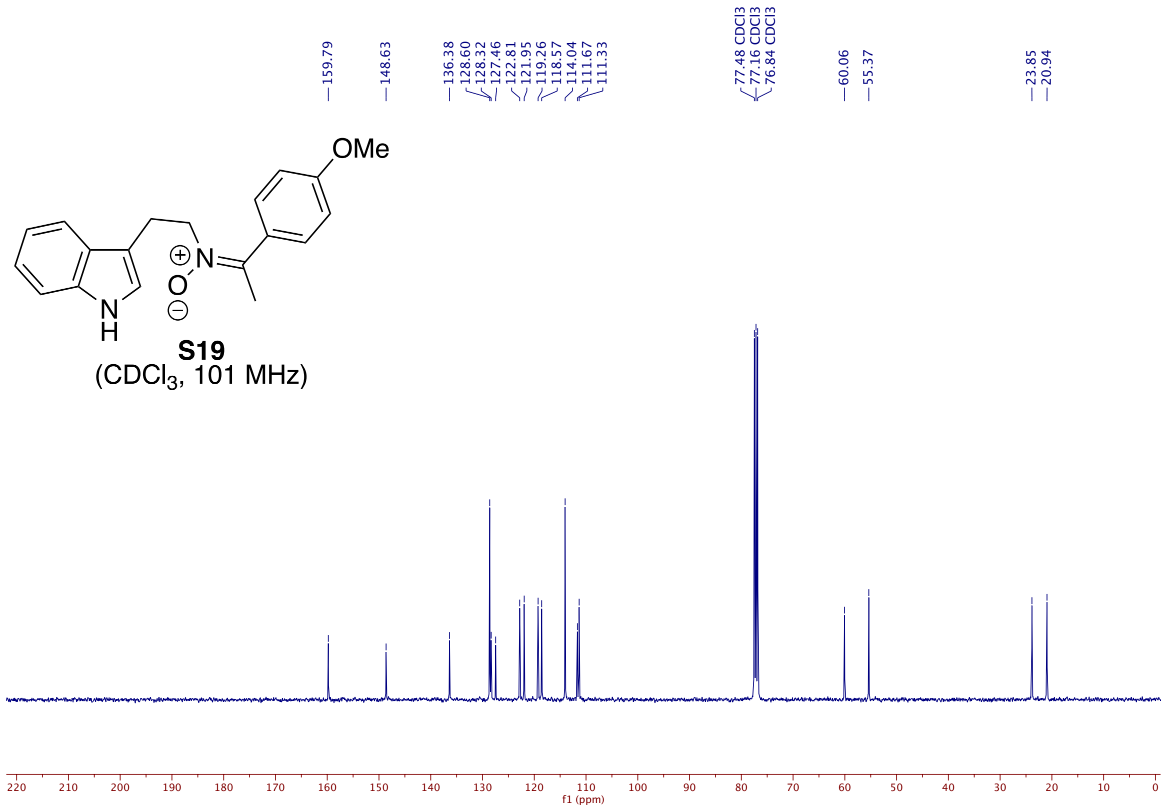
OMe
N
O
N
H
S19
(CDCl3, 101 MHz)

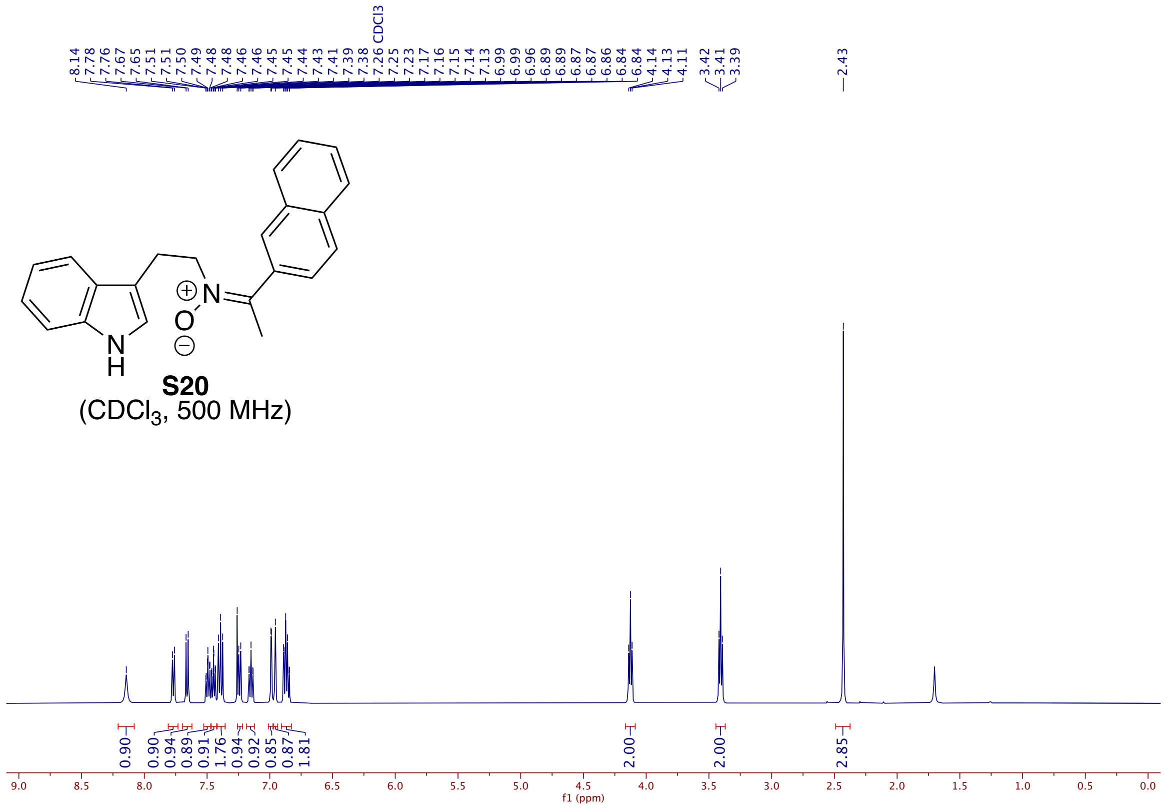
S20
(CDCl3, 500 MHz)
f1 (ppm)

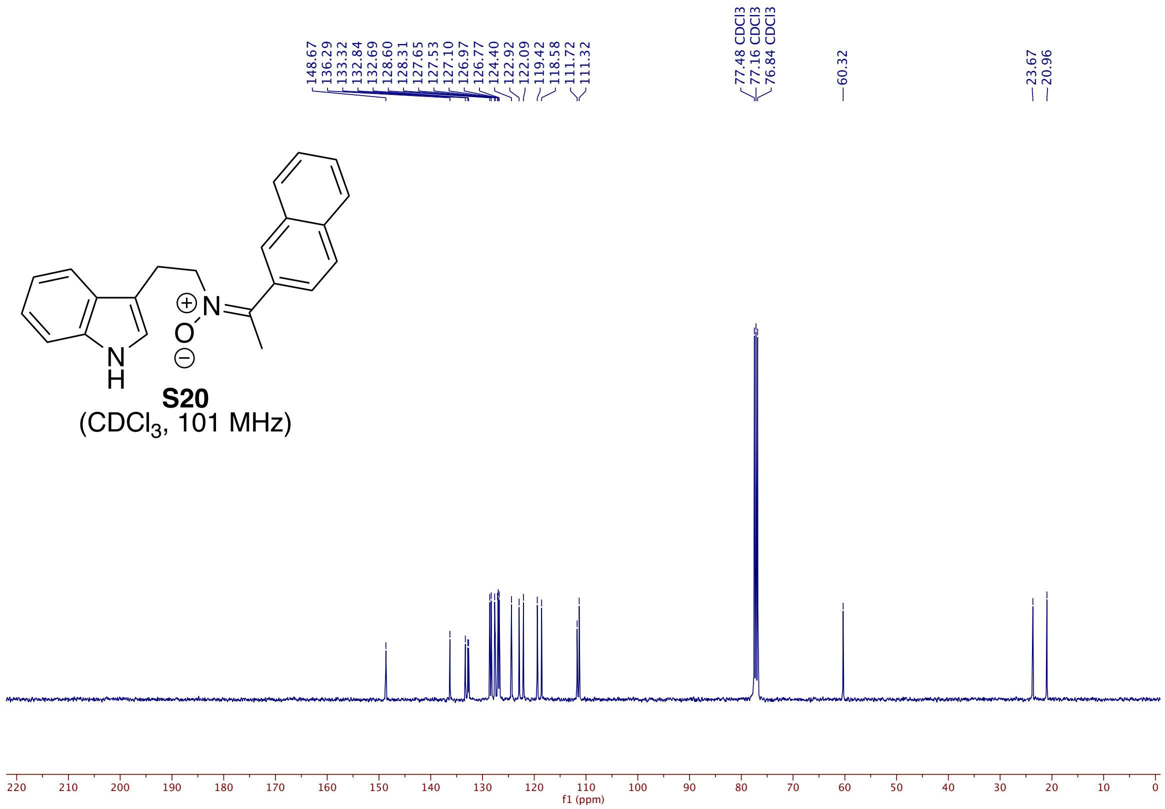
S20
(CDCl3, 101 MHz)
f1 (ppm)

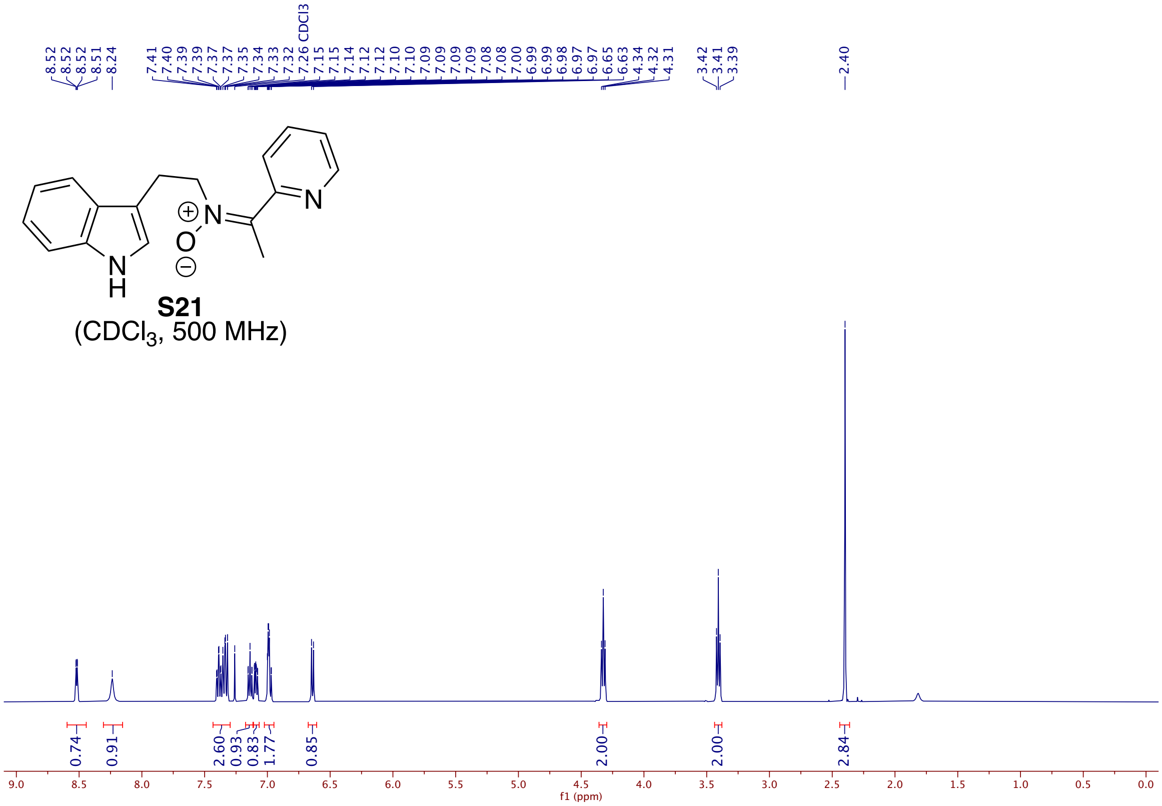
S21
(CDCl3, 500 MHz)
f1 (ppm)

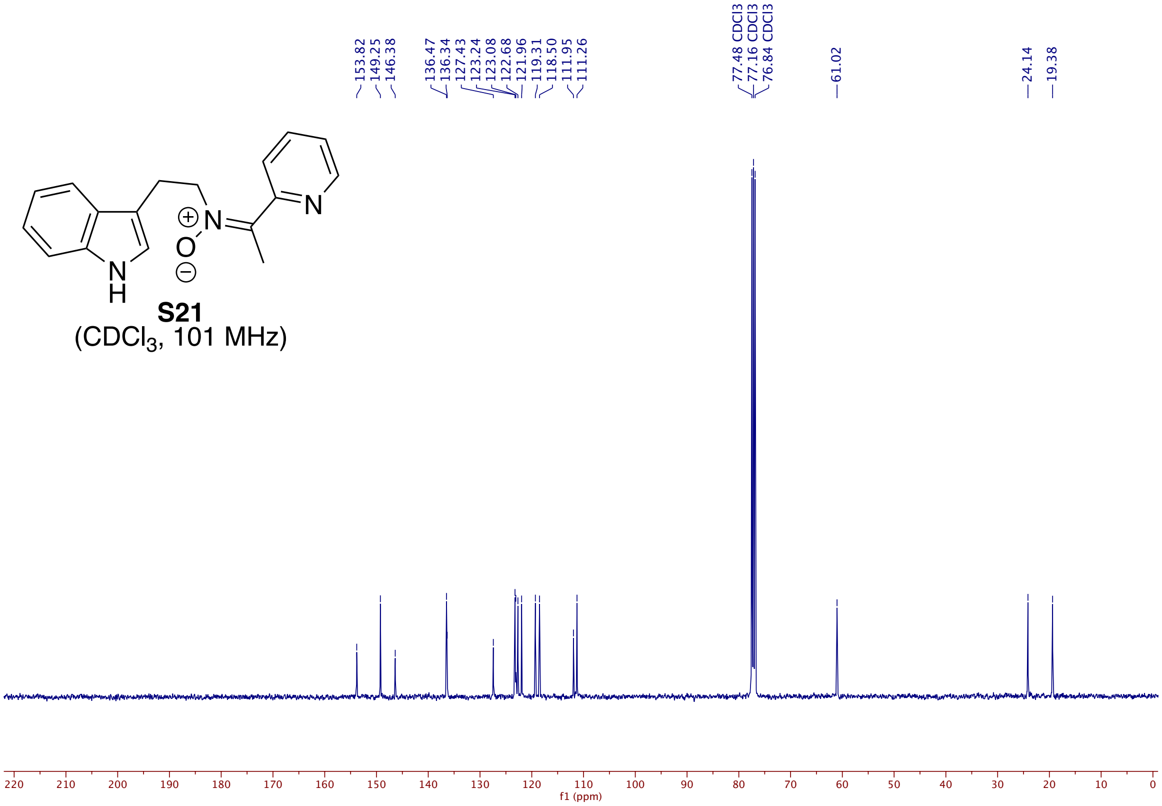
S21
(CDCl3, 101 MHz)
f1 (ppm)

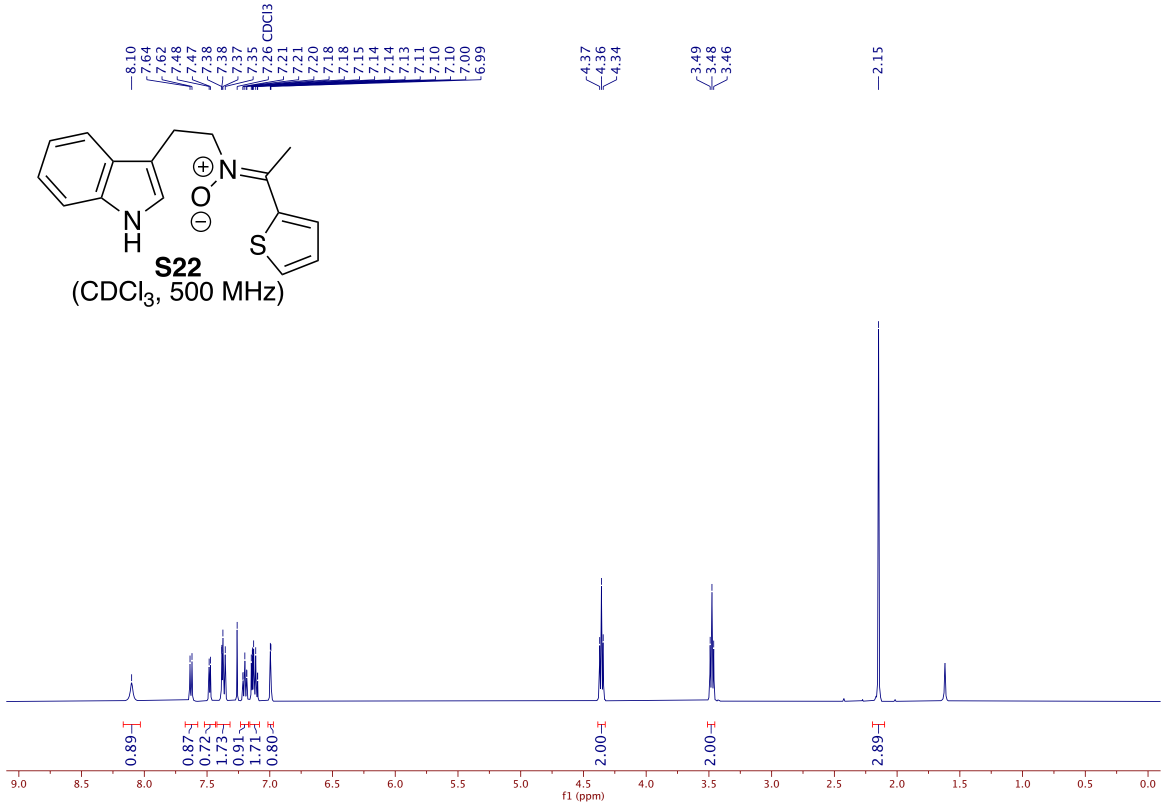
8.10
7.64
7.62
7.48
7.47
7.38
7.38
7.37
7.35
7.26 CDCl3
7.21
7.21
7.20
7.18
7.18
7.15
7.14
7.14
7.13
7.11
7.10
7.10
7.00
6.99
4.37
4.36
4.34
3.49
3.48
3.46
2.15
S22
(CDCl3, 500 MHz)
0.89
0.87
0.72
1.73
0.91
1.71
0.80
2.00
2.00
2.89
9.0
8.5
8.0
7.5
7.0
6.5
6.0
5.5
5.0
4.5
4.0
3.5
3.0
2.5
2.0
1.5
1.0
0.5
0.0
f1 (ppm)

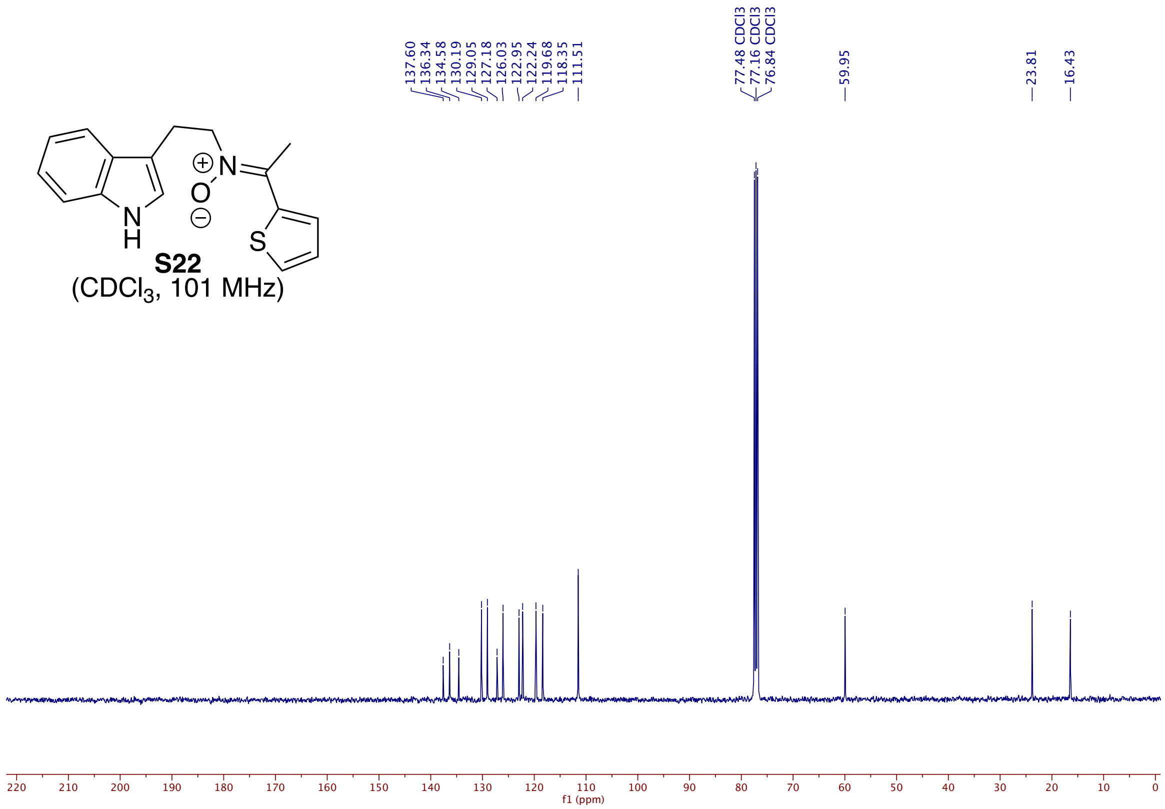
137.60
136.34
134.58
130.19
129.05
127.18
126.03
122.95
122.24
119.68
118.35
111.51
77.48 CDCl3
77.16 CDCl3
76.84 CDCl3
59.95
23.81
16.43
S22
(CDCl3, 101 MHz)
220
210
200
190
180
170
160
150
140
130
120
110
100
90
80
70
60
50
40
30
20
10
0
f1 (ppm)

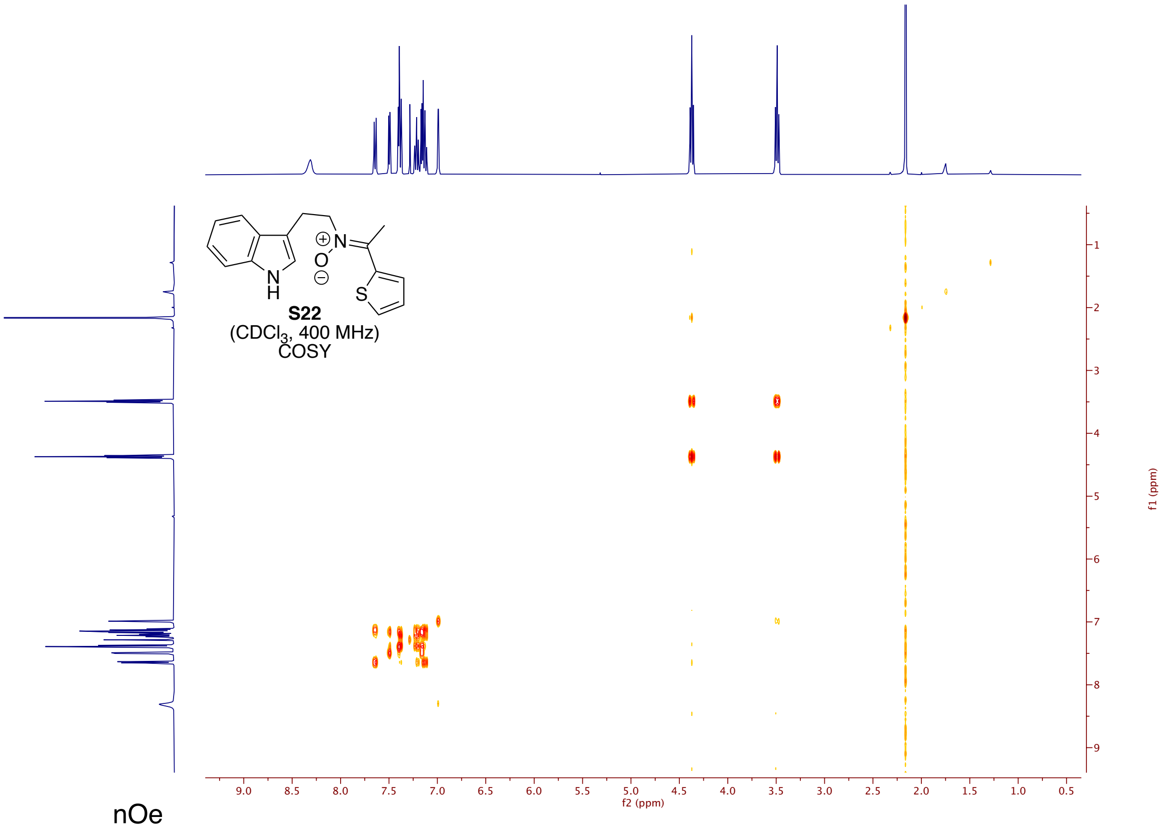
S22
(CDCl3, 400 MHz)
COSY
f2 (ppm)
f1 (ppm)

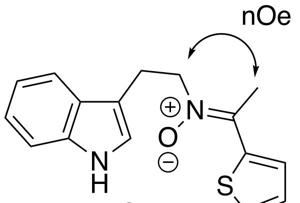
nOe

S22

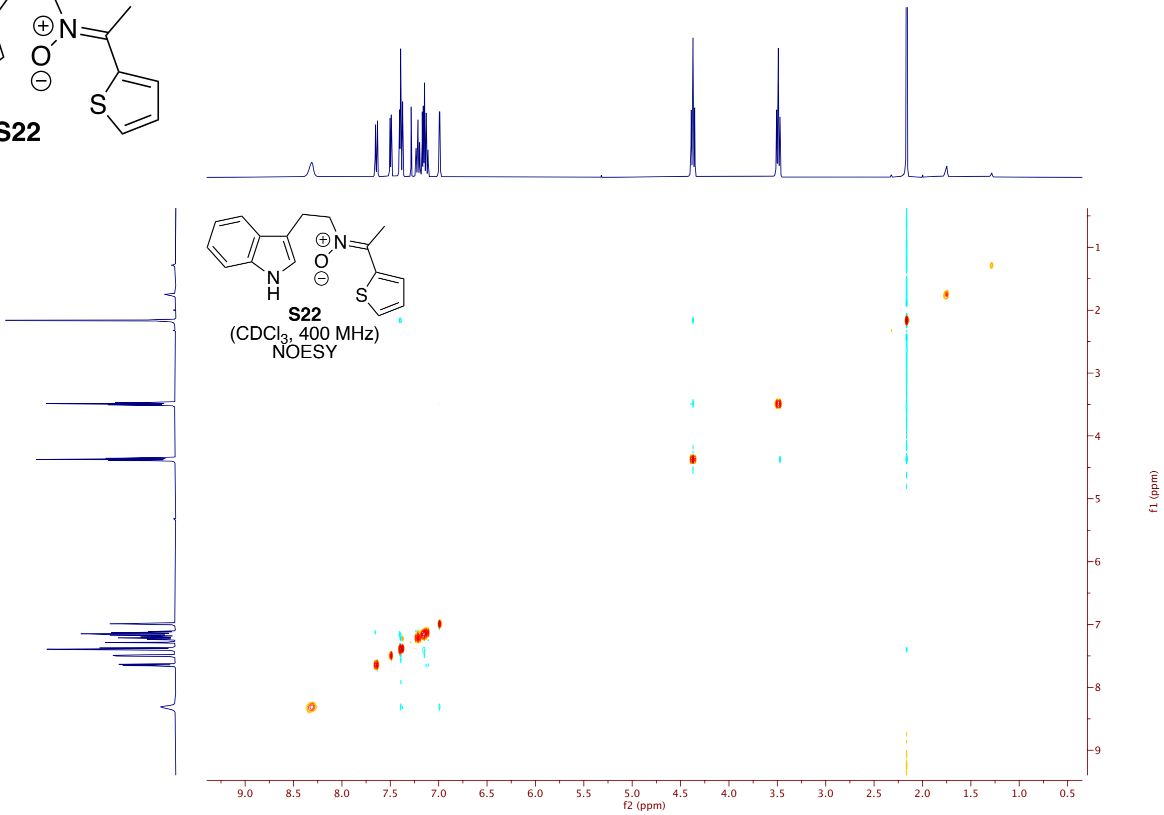
S22
(CDCl3, 400 MHz)
NOESY
f2 (ppm)
f1 (ppm)

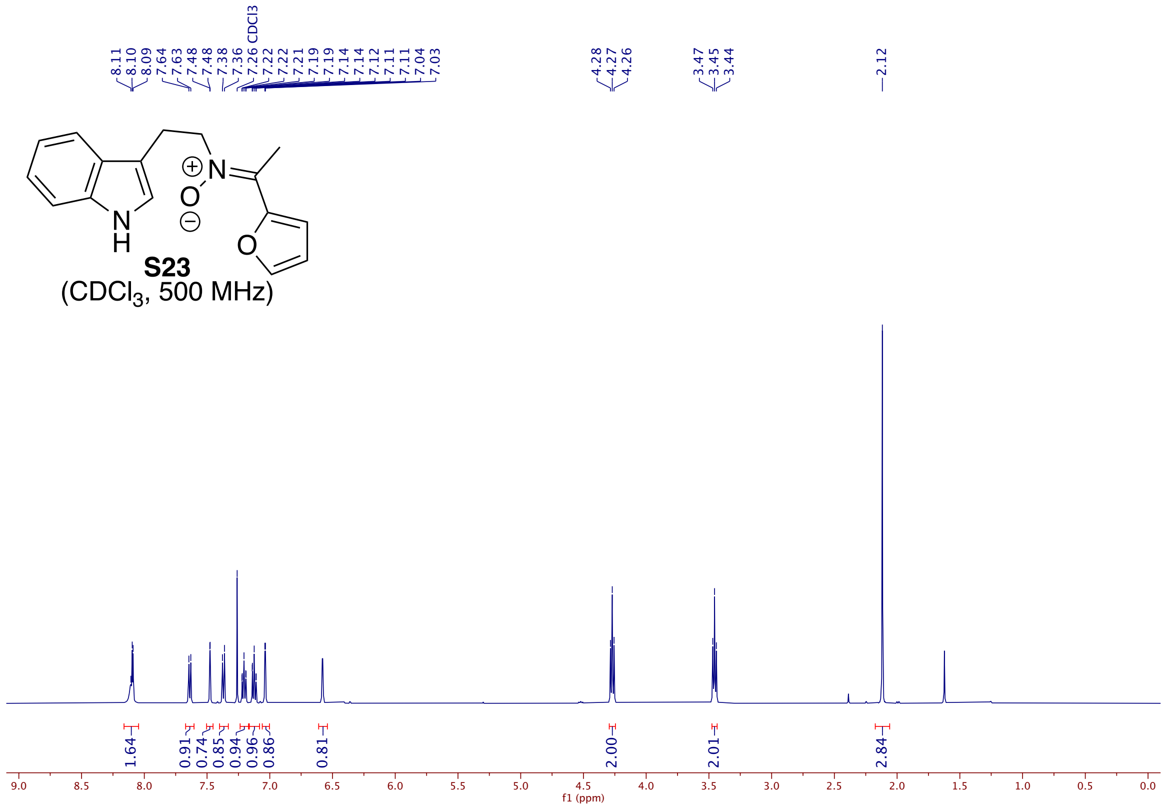

S23
($CDCl_3$, 500 MHz)

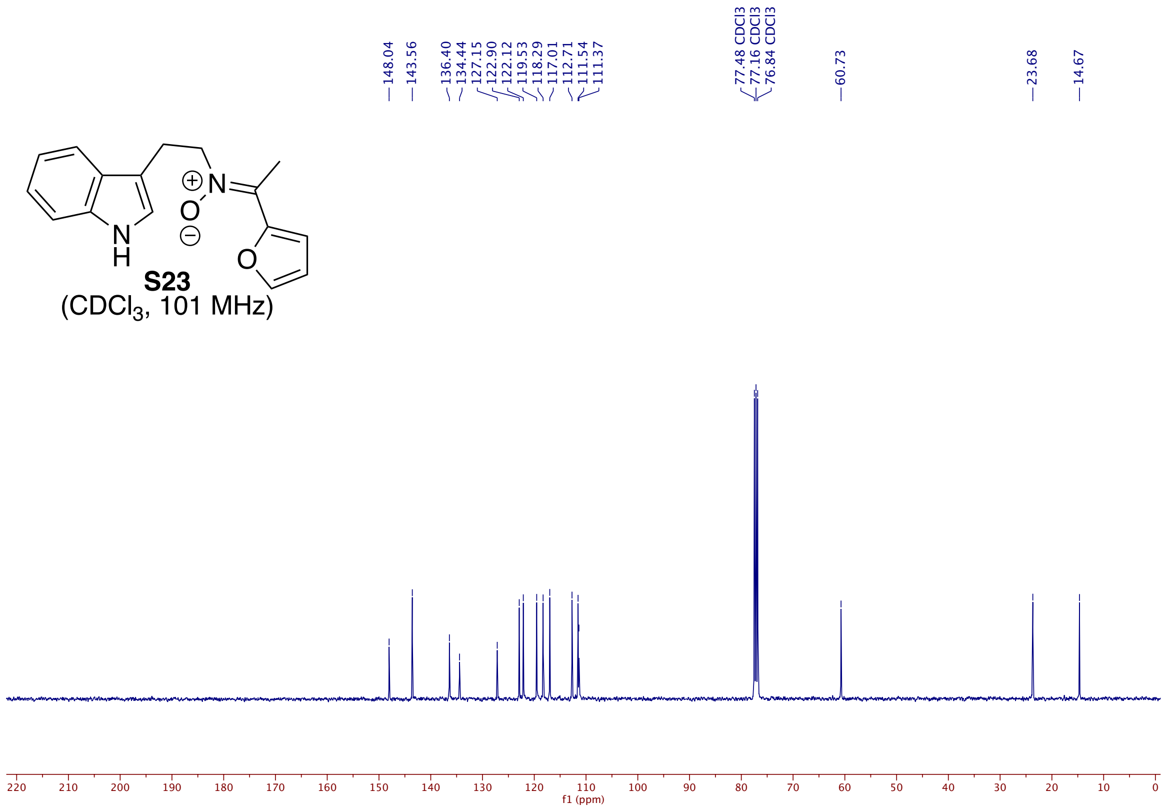

S23
($CDCl_3$, 101 MHz)

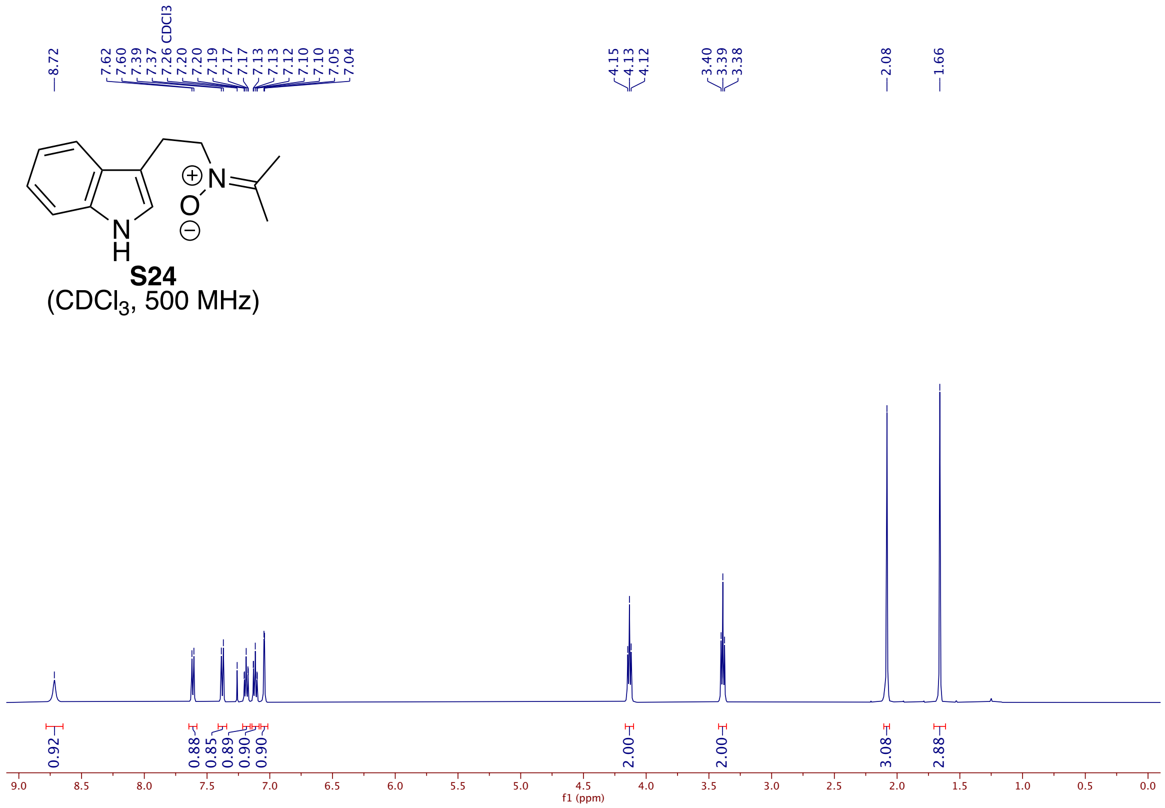

S24
($CDCl_3$, 500 MHz)

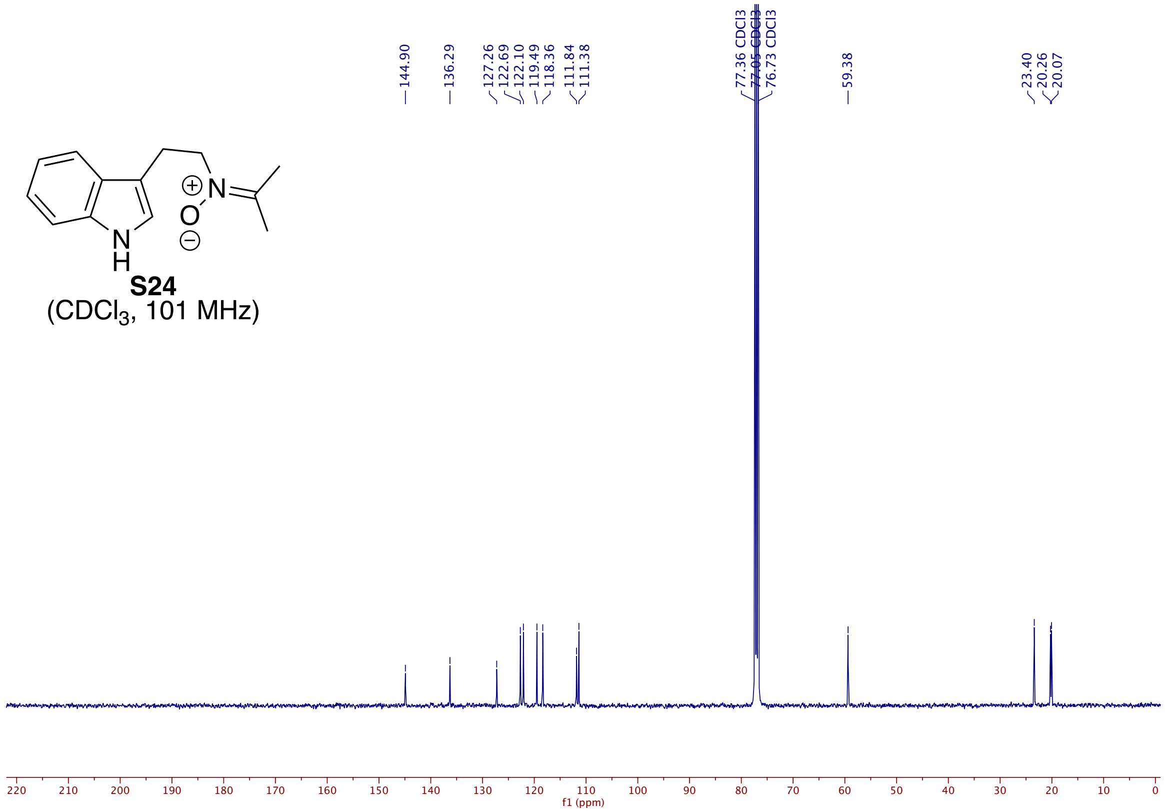

S24
($CDCl_3$, 101 MHz)

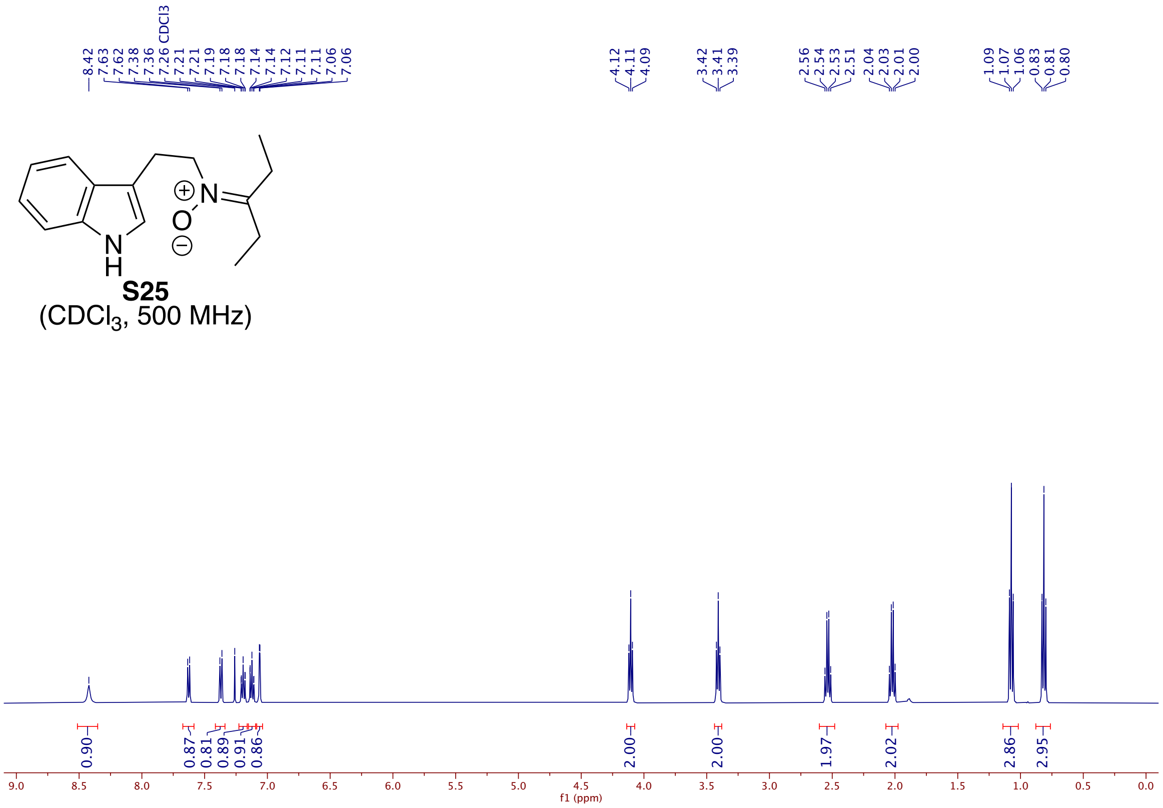

8.42
7.63
7.62
7.38
7.36
7.26 CDCl3
7.21
7.21
7.19
7.18
7.18
7.14
7.14
7.12
7.11
7.11
7.06
7.06
4.12
4.11
4.09
3.42
3.41
3.39
2.56
2.54
2.53
2.51
2.04
2.03
2.01
2.00
1.09
1.07
1.06
0.83
0.81
0.80
S25
(CDCl3, 500 MHz)
0.90
0.87
0.81
0.89
0.91
0.86
2.00
2.00
1.97
2.02
2.86
2.95
f1 (ppm)

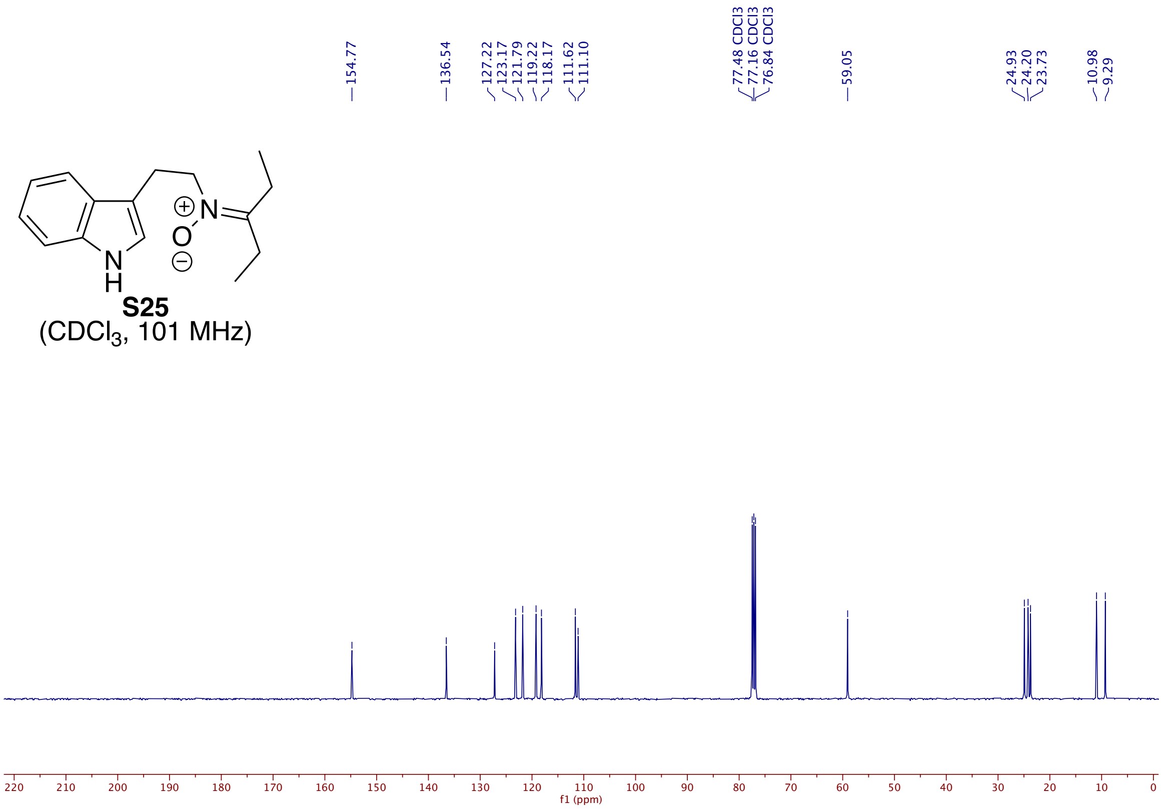

154.77
136.54
127.22
123.17
121.79
119.22
118.17
111.62
111.10
77.48 CDCl3
77.16 CDCl3
76.84 CDCl3
59.05
24.93
24.20
23.73
10.98
9.29
S25
(CDCl3, 101 MHz)
f1 (ppm)

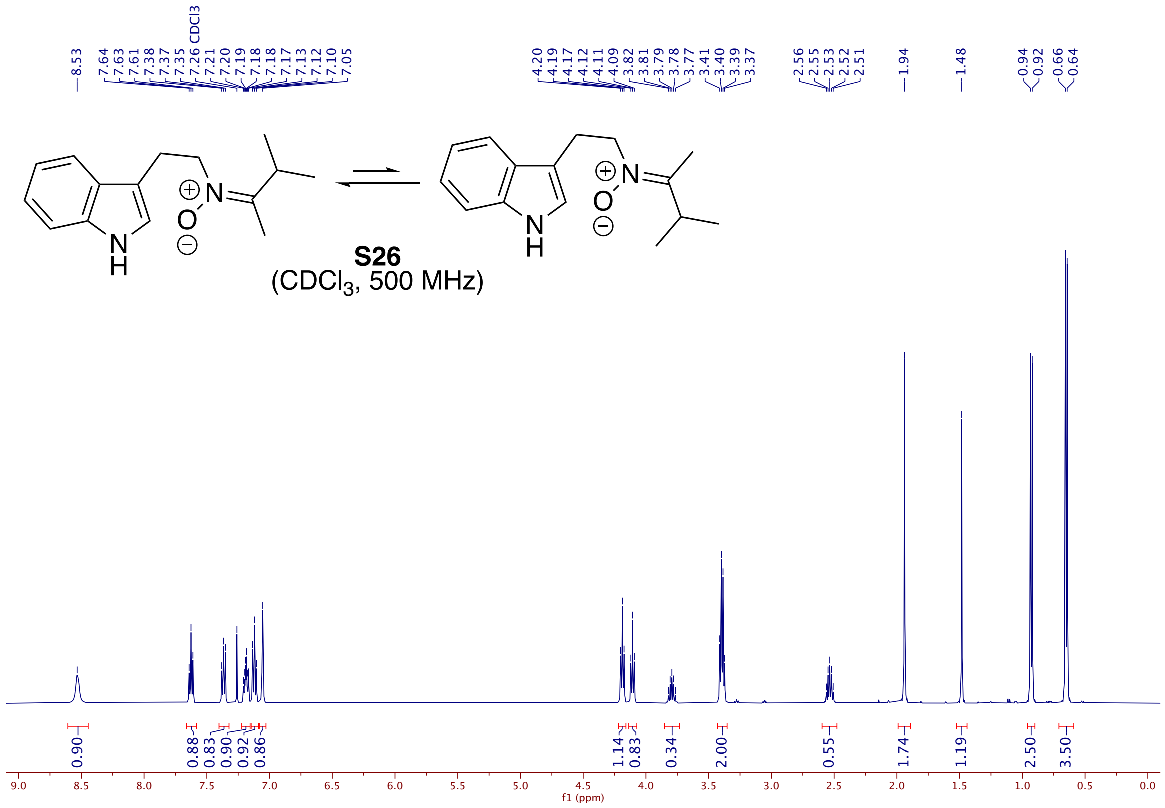
S26
(CDCl3, 500 MHz)
f1 (ppm)

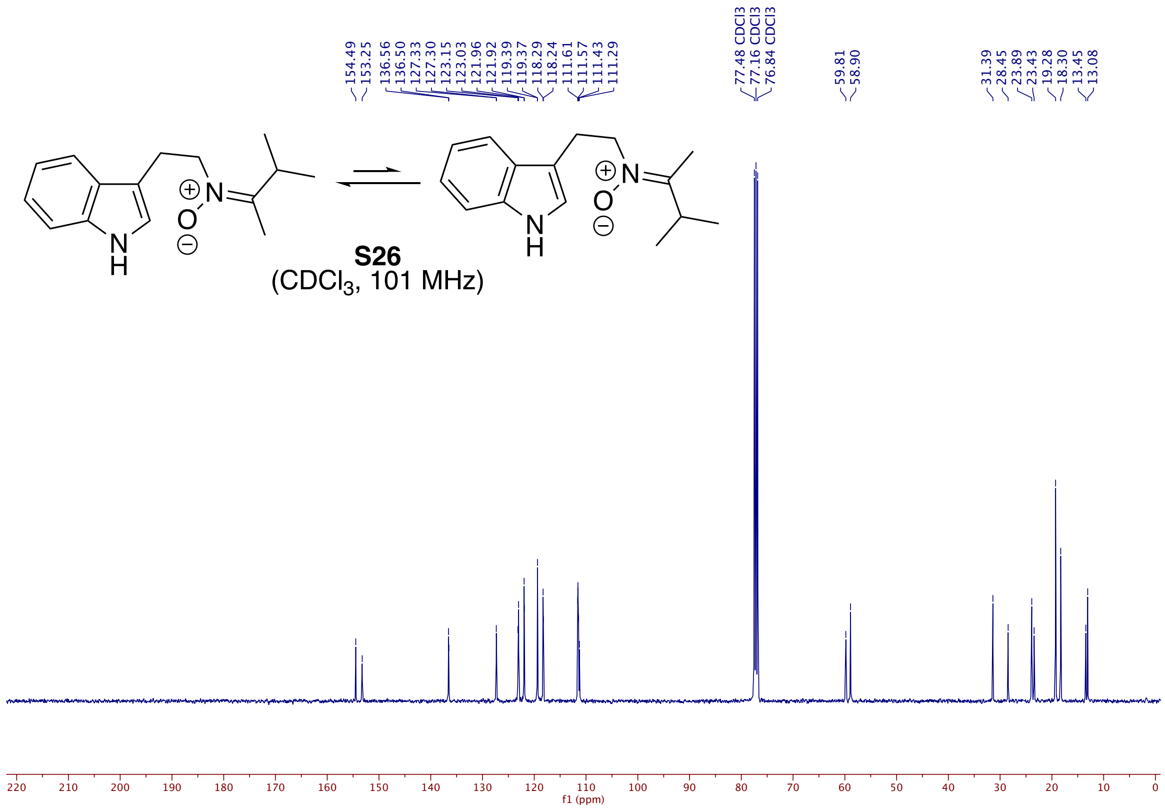
S26
(CDCl3, 101 MHz)
f1 (ppm)

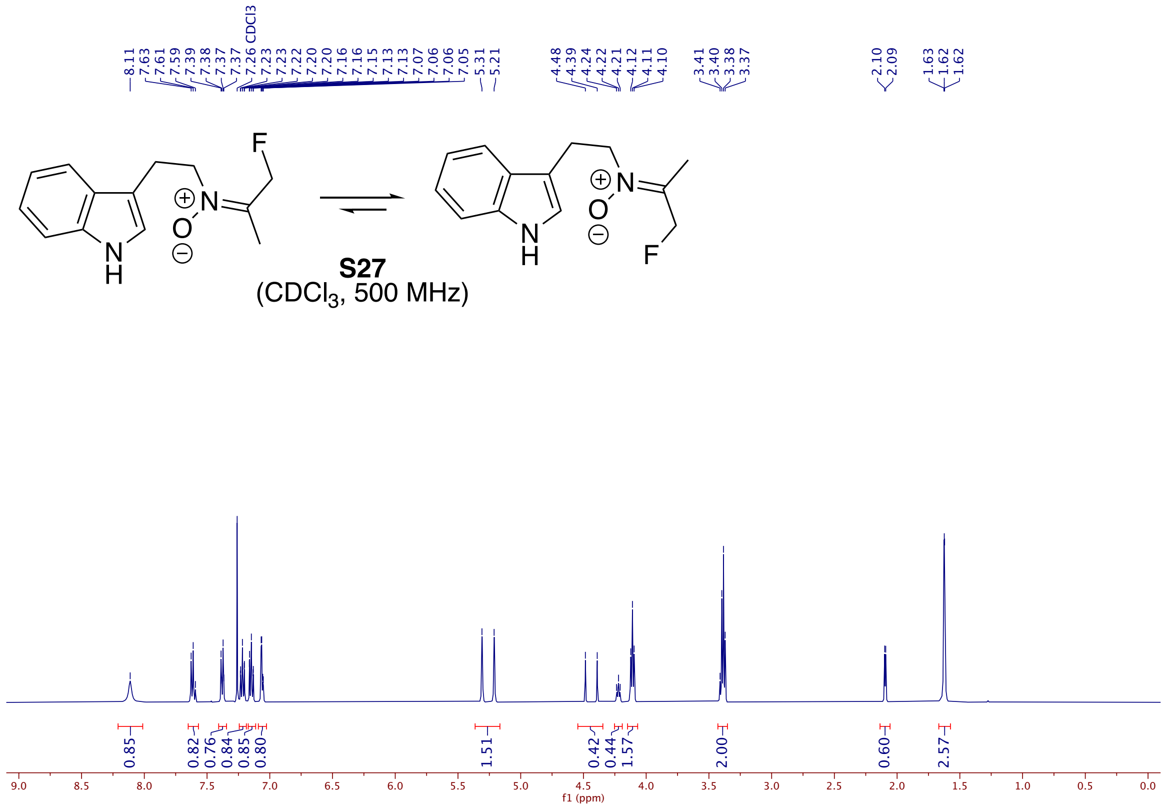
8.11 7.63 7.61 7.59 7.39 7.38 7.37 7.37 7.26 CDCl3 7.23 7.23 7.22 7.20 7.20 7.16 7.16 7.15 7.13 7.13 7.07 7.06 7.06 7.05 5.31 5.21
4.48 4.39 4.24 4.22 4.21 4.12 4.11 4.10
3.41 3.40 3.38 3.37
2.10 2.09
1.63 1.62 1.62
F
N
O
N
H
F
S27
(CDCl3, 500 MHz)
0.85 0.82 0.76 0.84 0.85 0.80 1.51 0.42 0.44 1.57 2.00 0.60 2.57
9.0 8.5 8.0 7.5 7.0 6.5 6.0 5.5 5.0 4.5 4.0 3.5 3.0 2.5 2.0 1.5 1.0 0.5 0.0
f1 (ppm)

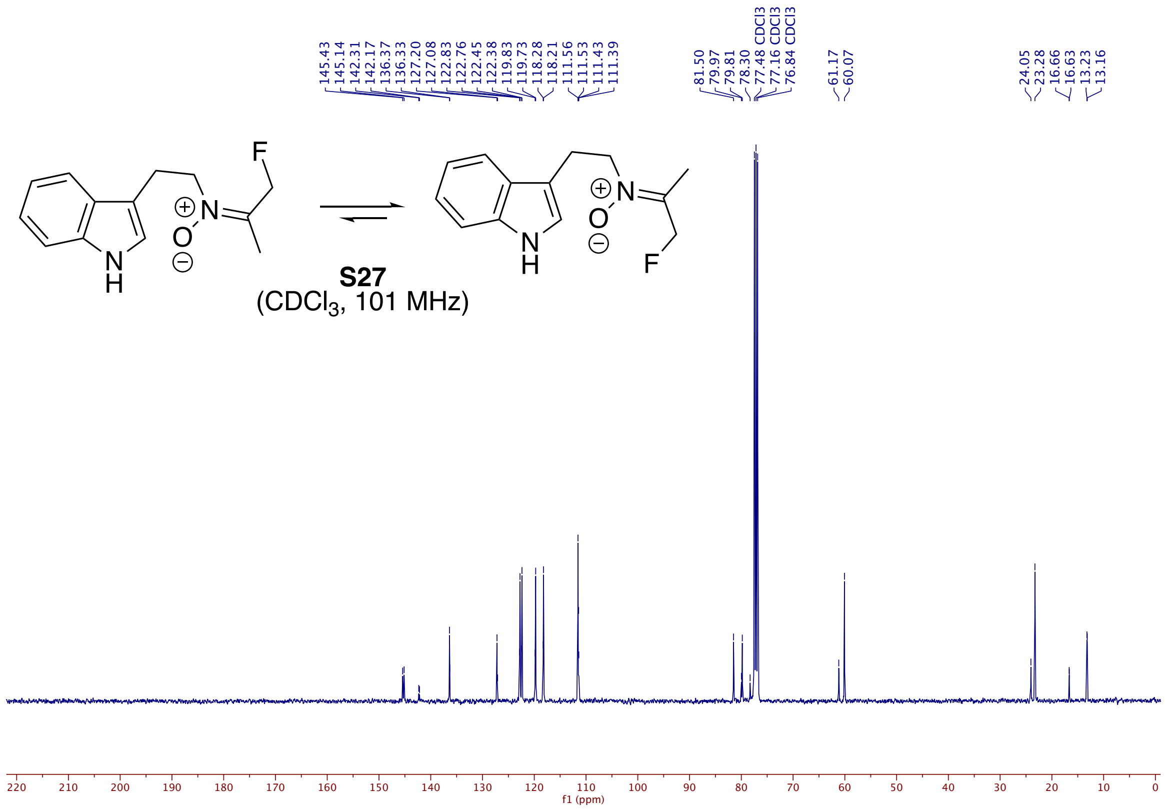
145.43 145.14 142.31 142.17 136.37 136.33 127.20 127.08 122.83 122.76 122.45 122.38 119.83 119.73 118.28 118.21 111.56 111.53 111.43 111.39
81.50 79.97 79.81 78.30 77.48 CDCl3 77.16 CDCl3 76.84 CDCl3
61.17 60.07
24.05 23.28 16.66 16.63 13.23 13.16
F
N
O
N
H
F
S27
(CDCl3, 101 MHz)
220 210 200 190 180 170 160 150 140 130 120 110 100 90 80 70 60 50 40 30 20 10 0
f1 (ppm)

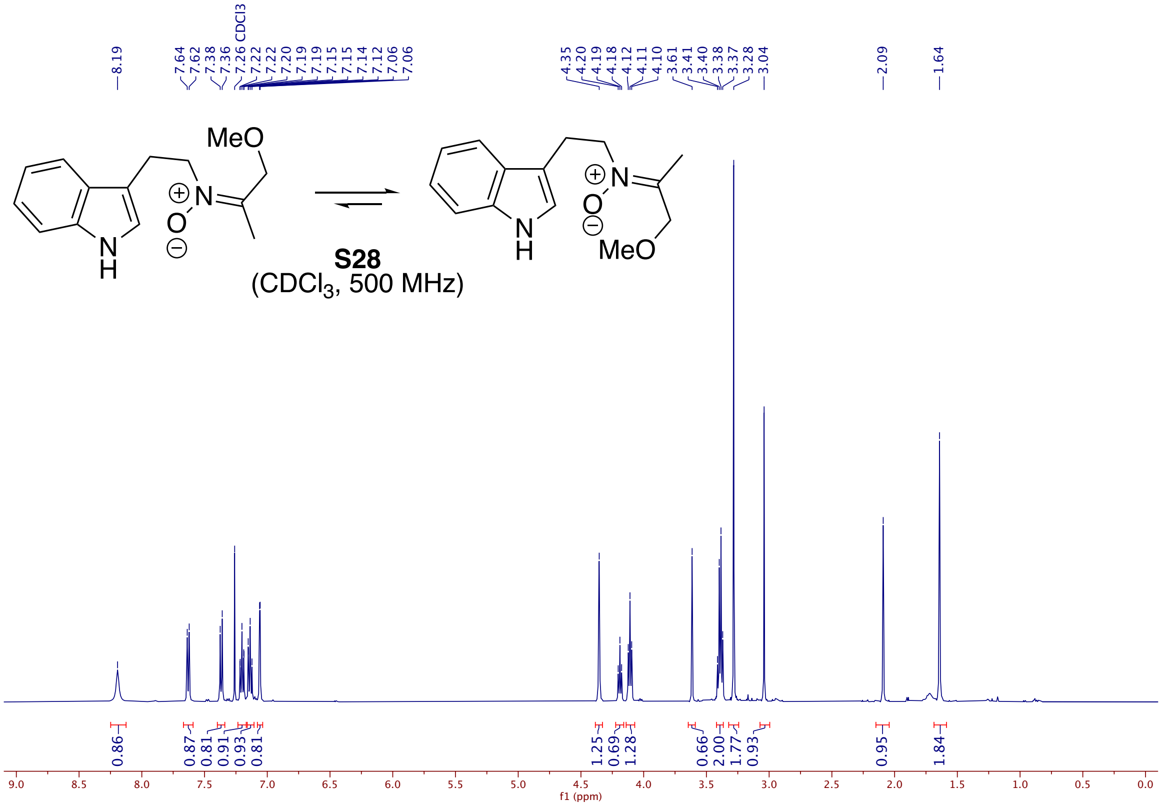

S28
($CDCl_3$, 500 MHz)

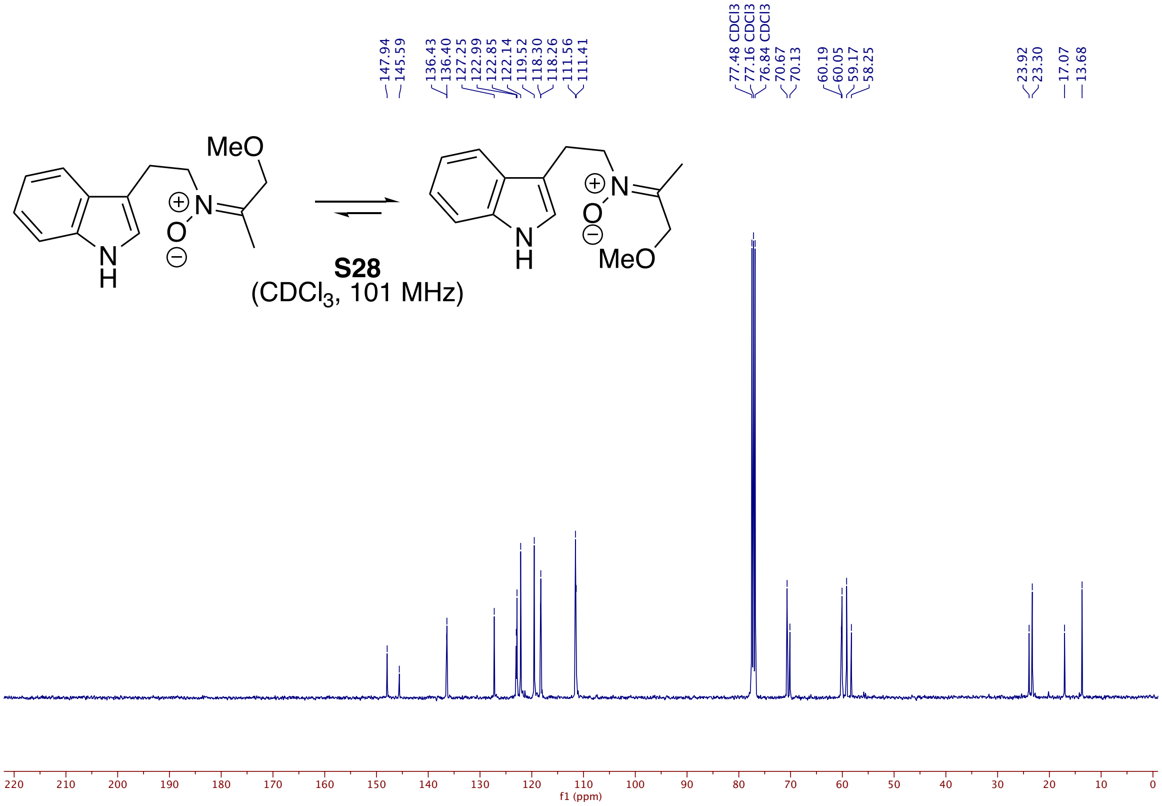

S28
($CDCl_3$, 101 MHz)

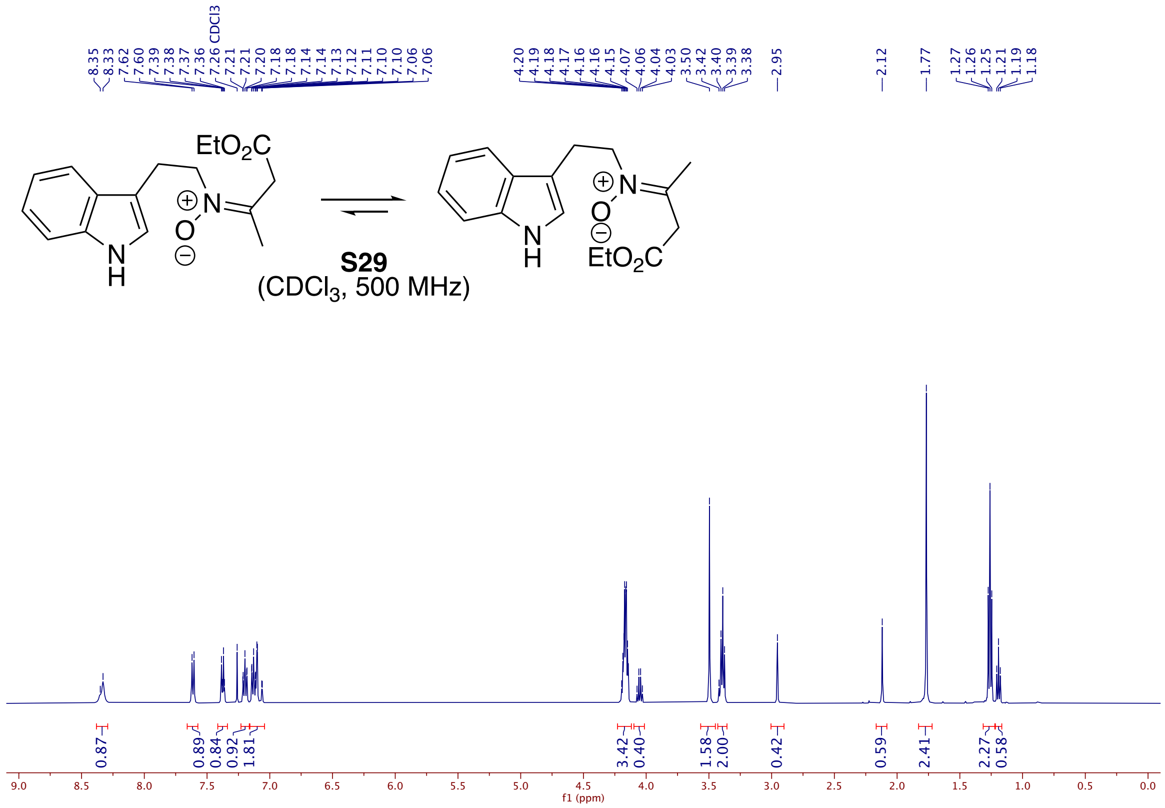
8.35
8.33
7.62
7.60
7.39
7.38
7.37
7.36
7.26 CDCl3
7.21
7.21
7.20
7.18
7.18
7.14
7.13
7.12
7.11
7.10
7.10
7.06
7.06
4.20
4.19
4.18
4.17
4.16
4.16
4.15
4.07
4.06
4.04
4.03
3.50
3.42
3.40
3.39
3.38
2.95
2.12
1.77
1.27
1.26
1.25
1.21
1.19
1.18
EtO2C
N
H
N
O
EtO2C
S29
(CDCl3, 500 MHz)
0.87
0.89
0.84
0.92
1.81
3.42
0.40
1.58
2.00
0.42
0.59
2.41
2.27
0.58
9.0
8.5
8.0
7.5
7.0
6.5
6.0
5.5
5.0
4.5
4.0
3.5
3.0
2.5
2.0
1.5
1.0
0.5
0.0
f1 (ppm)

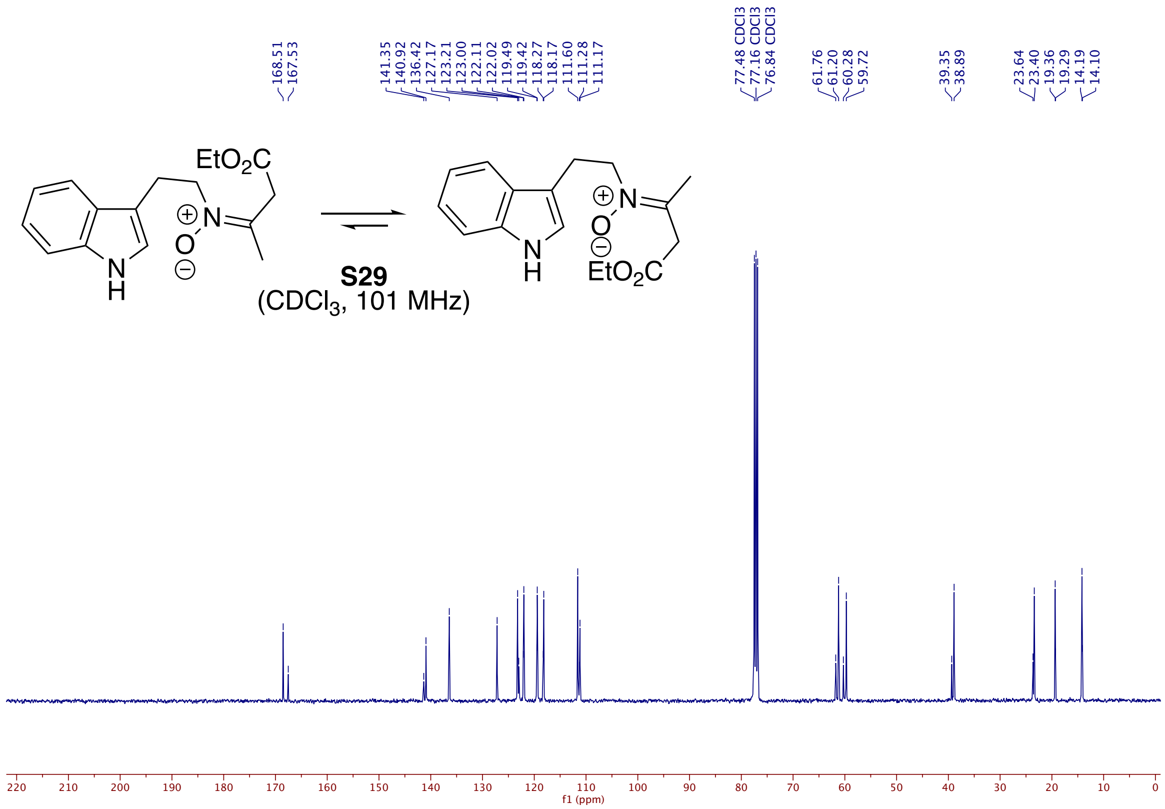
168.51
167.53
141.35
140.92
136.42
127.17
123.21
123.00
122.11
122.02
119.49
119.42
118.27
118.17
111.60
111.28
111.17
77.48 CDCl3
77.16 CDCl3
76.84 CDCl3
61.76
61.20
60.28
59.72
39.35
38.89
23.64
23.40
19.36
19.29
14.19
14.10
EtO2C
N
H
N
O
EtO2C
S29
(CDCl3, 101 MHz)
220
210
200
190
180
170
160
150
140
130
120
110
100
90
80
70
60
50
40
30
20
10
0
f1 (ppm)

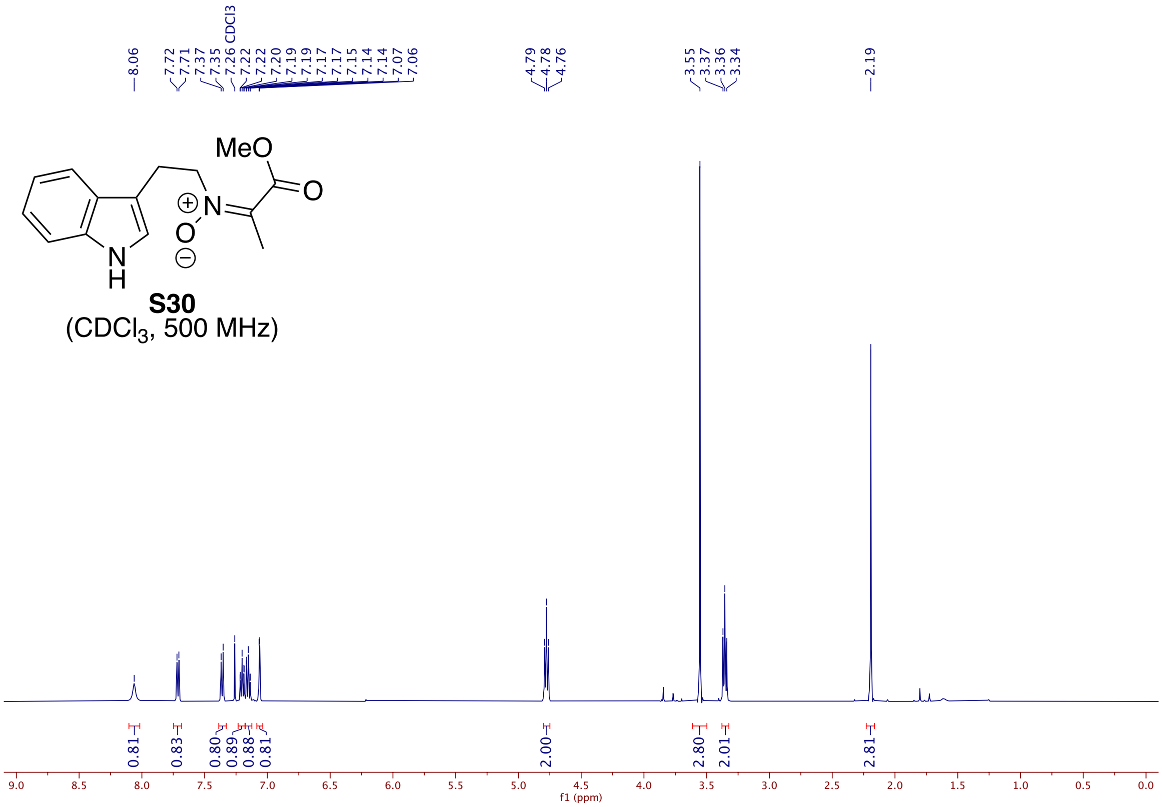
8.06
7.72
7.71
7.37
7.35
7.26 CDCl3
7.22
7.22
7.20
7.19
7.19
7.17
7.17
7.15
7.14
7.14
7.07
7.06
4.79
4.78
4.76
3.55
3.37
3.36
3.34
2.19
MeO
O
N
O
N
H
S30
(CDCl3, 500 MHz)
0.81
0.83
0.80
0.89
0.88
0.81
2.00
2.80
2.01
2.81
9.0 8.5 8.0 7.5 7.0 6.5 6.0 5.5 5.0 4.5 4.0 3.5 3.0 2.5 2.0 1.5 1.0 0.5 0.0
f1 (ppm)

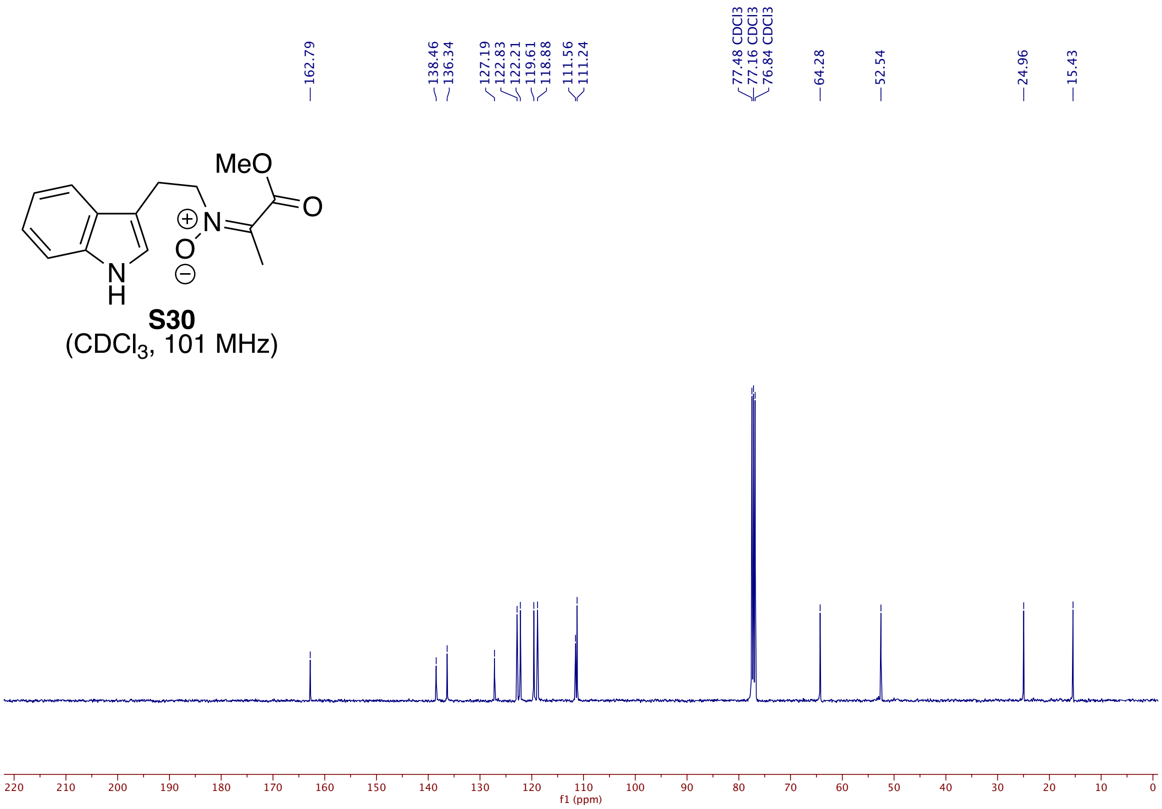
162.79
138.46
136.34
127.19
122.83
122.21
119.61
118.88
111.56
111.24
77.48 CDCl3
77.16 CDCl3
76.84 CDCl3
64.28
52.54
24.96
15.43
MeO
O
N
O
N
H
S30
(CDCl3, 101 MHz)
220 210 200 190 180 170 160 150 140 130 120 110 100 90 80 70 60 50 40 30 20 10 0
f1 (ppm)

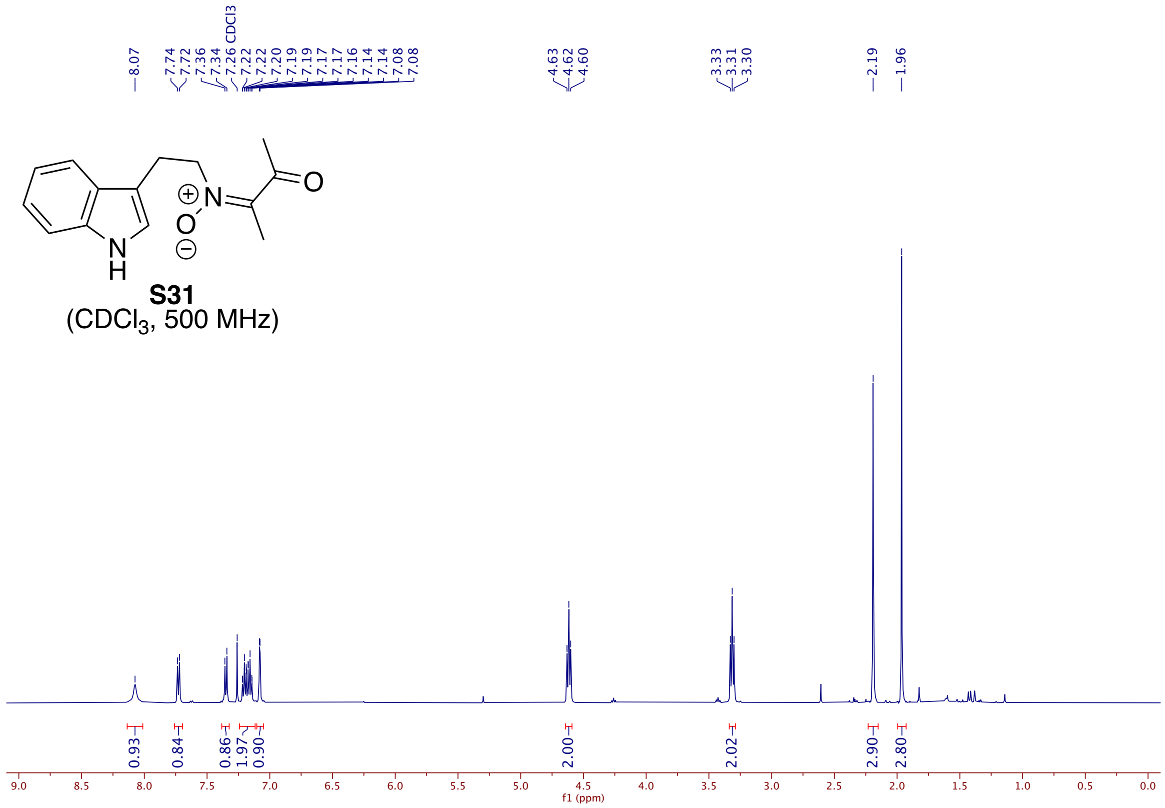
8.07
7.74
7.72
7.36
7.34
7.26 CDCl3
7.22
7.22
7.20
7.19
7.19
7.17
7.17
7.16
7.14
7.14
7.08
7.08
4.63
4.62
4.60
3.33
3.31
3.30
2.19
1.96
S31
(CDCl3, 500 MHz)
0.93
0.84
0.86
1.97
0.90
2.00
2.02
2.90
2.80
f1 (ppm)

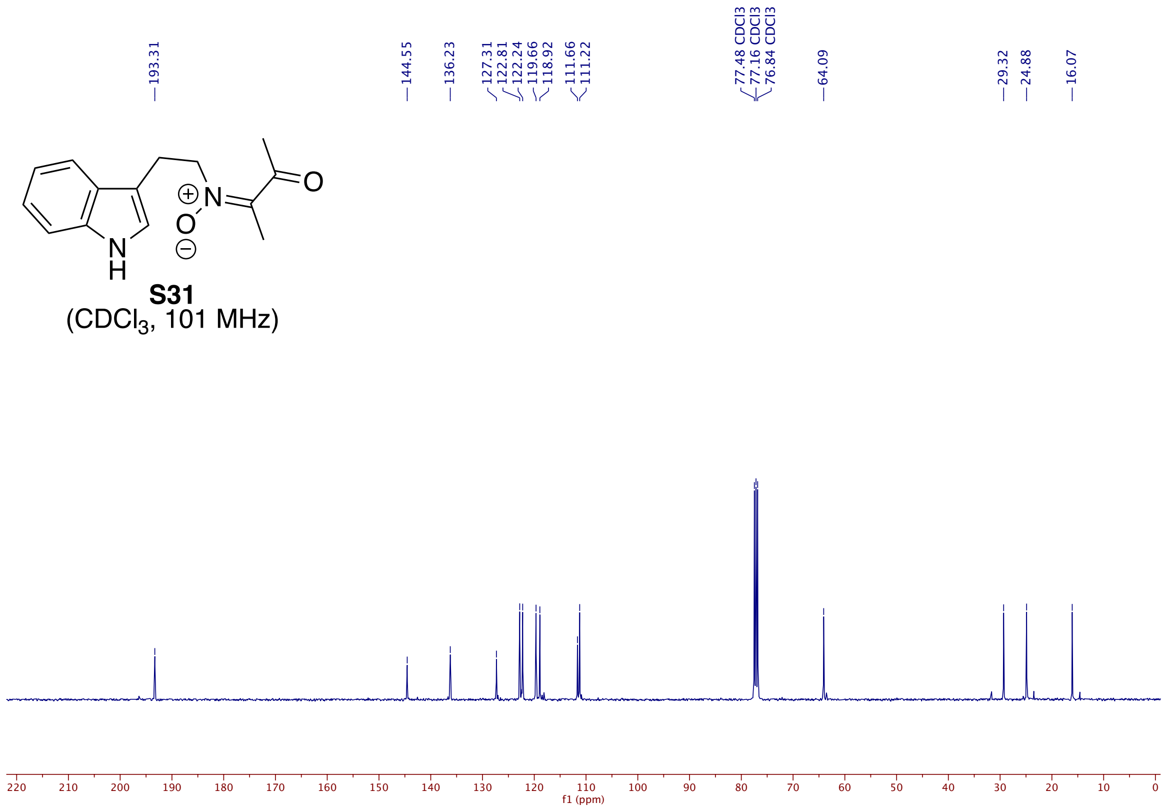
193.31
144.55
136.23
127.31
122.81
122.24
119.66
118.92
111.66
111.22
77.48 CDCl3
77.16 CDCl3
76.84 CDCl3
64.09
29.32
24.88
16.07
S31
(CDCl3, 101 MHz)
f1 (ppm)

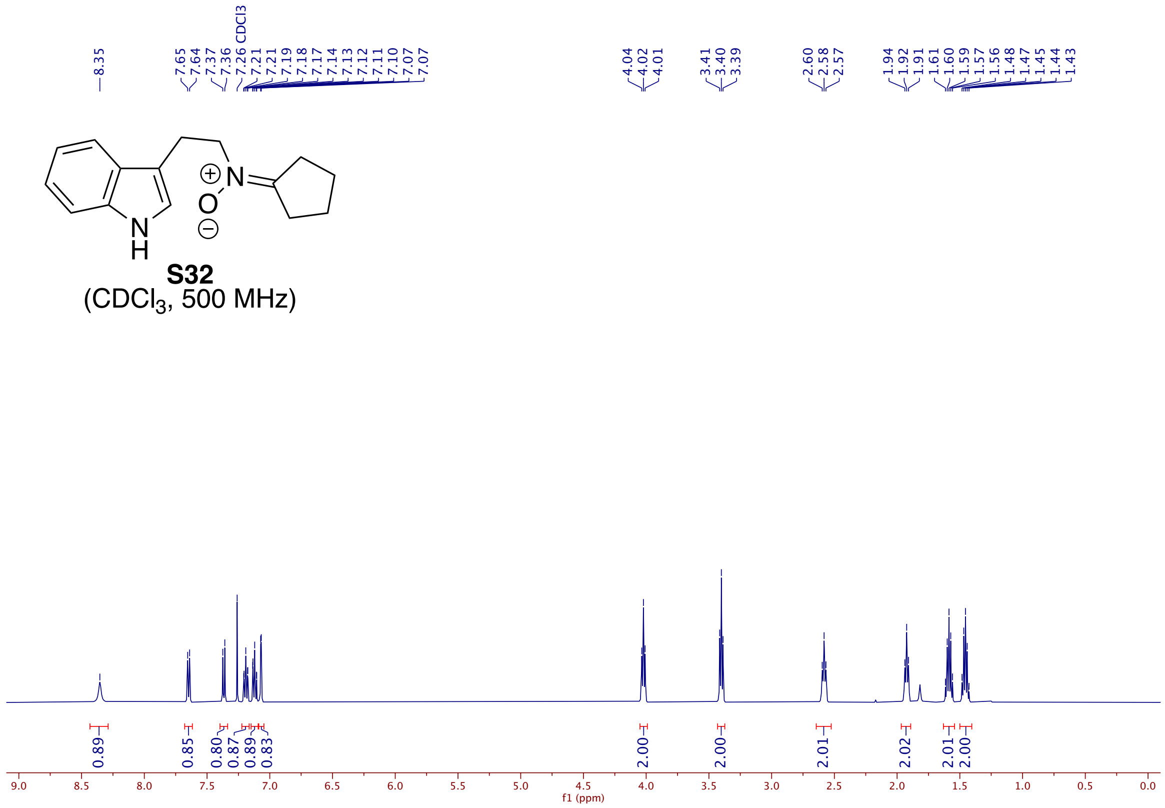
S32
(CDCl3, 500 MHz)
8.35
7.65
7.64
7.37
7.36
7.26 CDCl3
7.21
7.21
7.19
7.18
7.17
7.14
7.13
7.12
7.11
7.10
7.07
7.07
4.04
4.02
4.01
3.41
3.40
3.39
2.60
2.58
2.57
1.94
1.92
1.91
1.61
1.60
1.59
1.57
1.56
1.48
1.47
1.45
1.44
1.43
0.89
0.85
0.80
0.87
0.89
0.83
2.00
2.00
2.01
2.02
2.01
2.00
f1 (ppm)

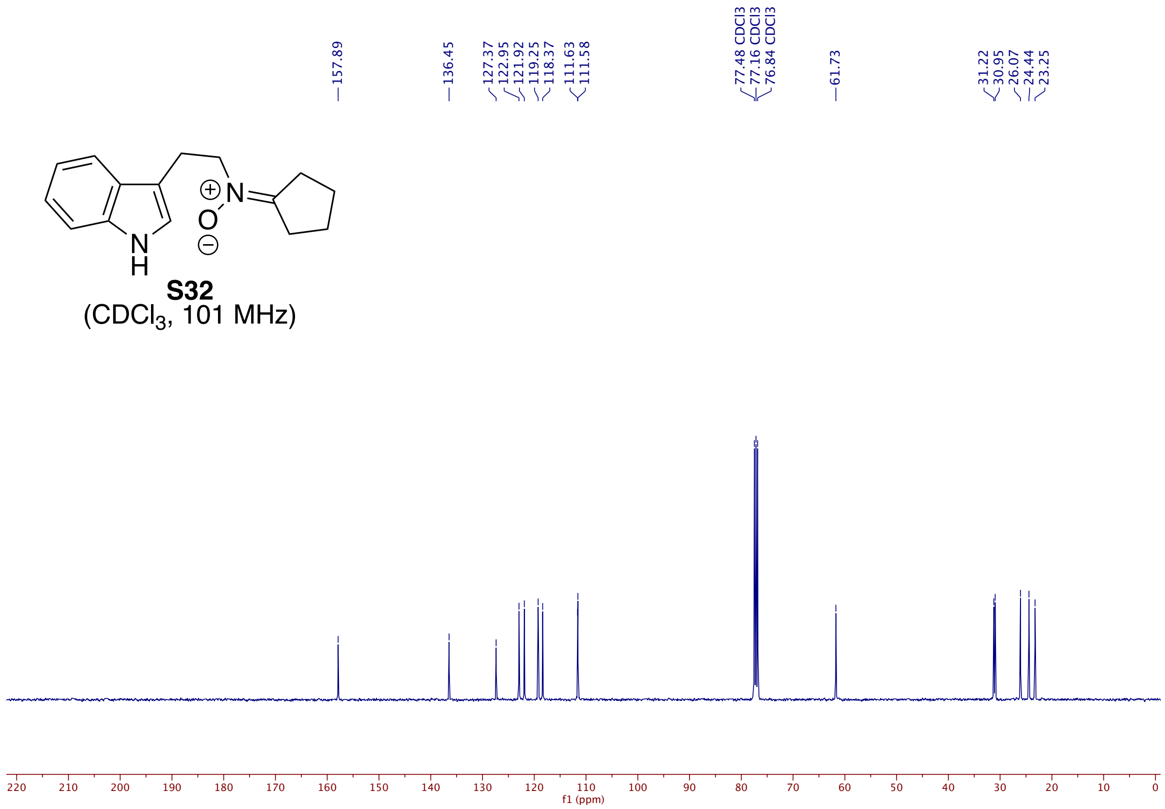
S32
(CDCl3, 101 MHz)
157.89
136.45
127.37
122.95
121.92
119.25
118.37
111.63
111.58
77.48 CDCl3
77.16 CDCl3
76.84 CDCl3
61.73
31.22
30.95
26.07
24.44
23.25
f1 (ppm)

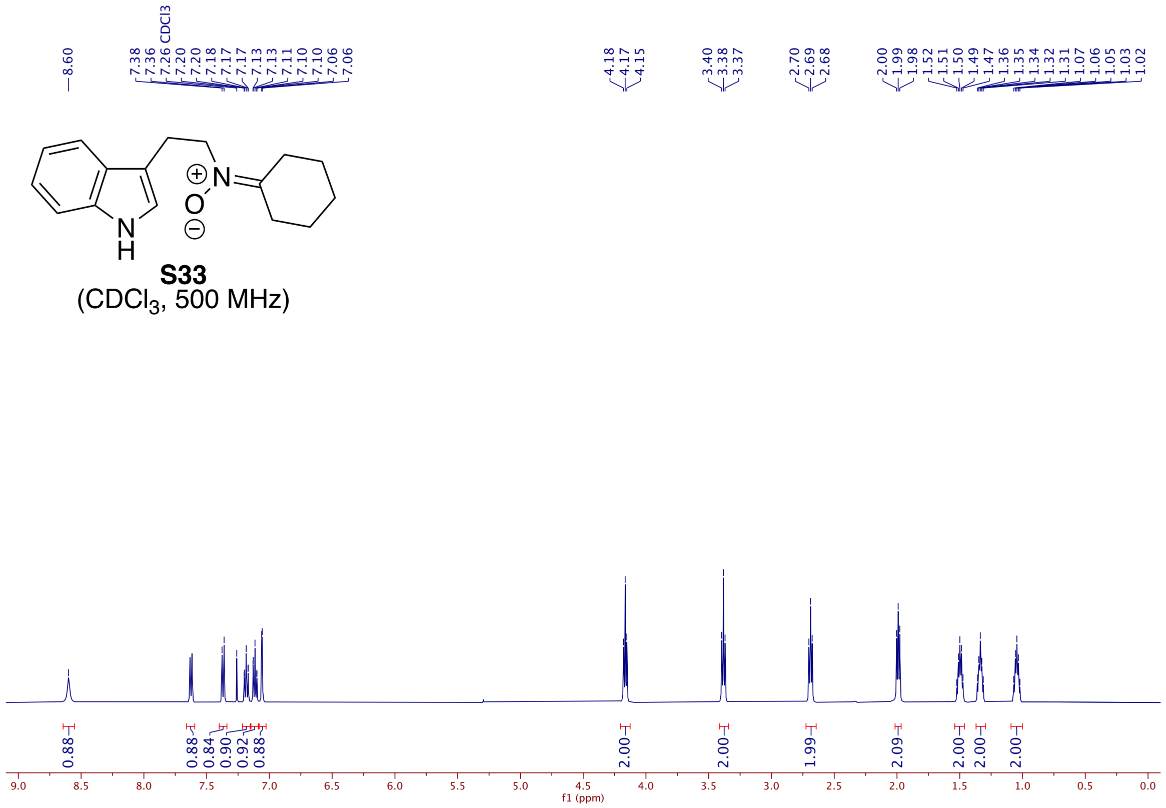

S33
($CDCl_3$, 500 MHz)

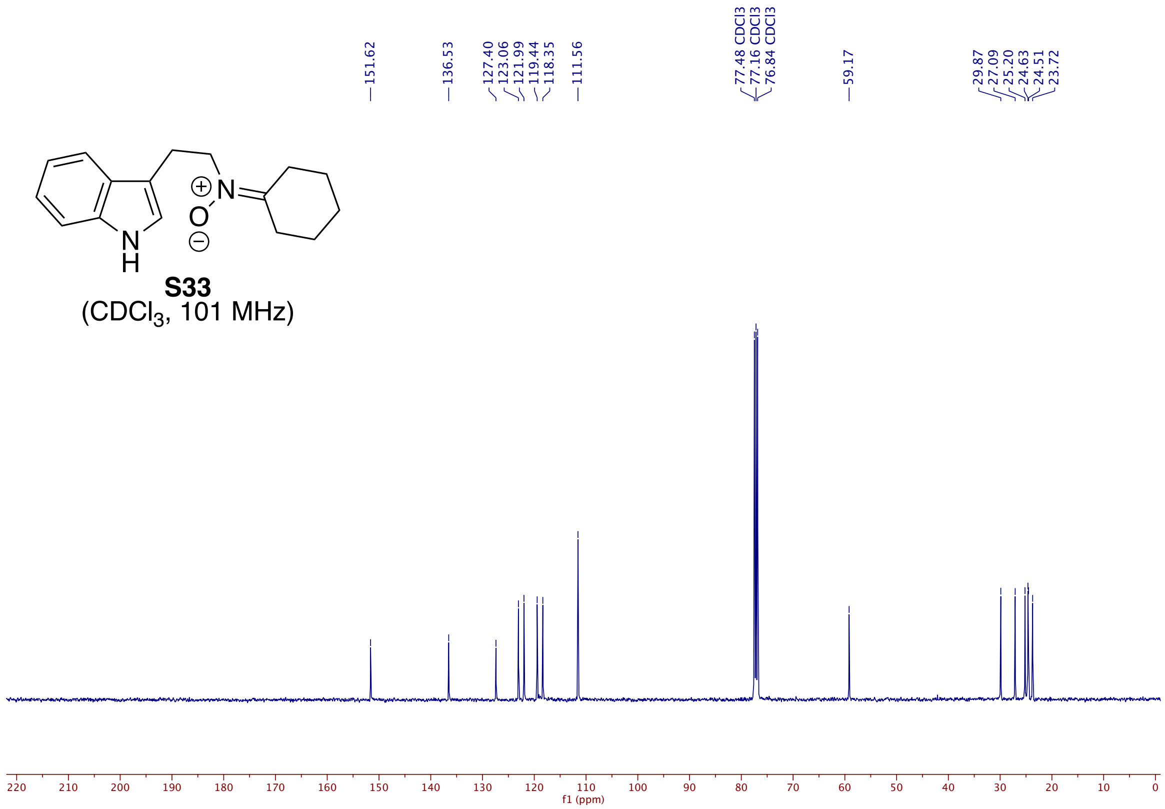

S33
($CDCl_3$, 101 MHz)

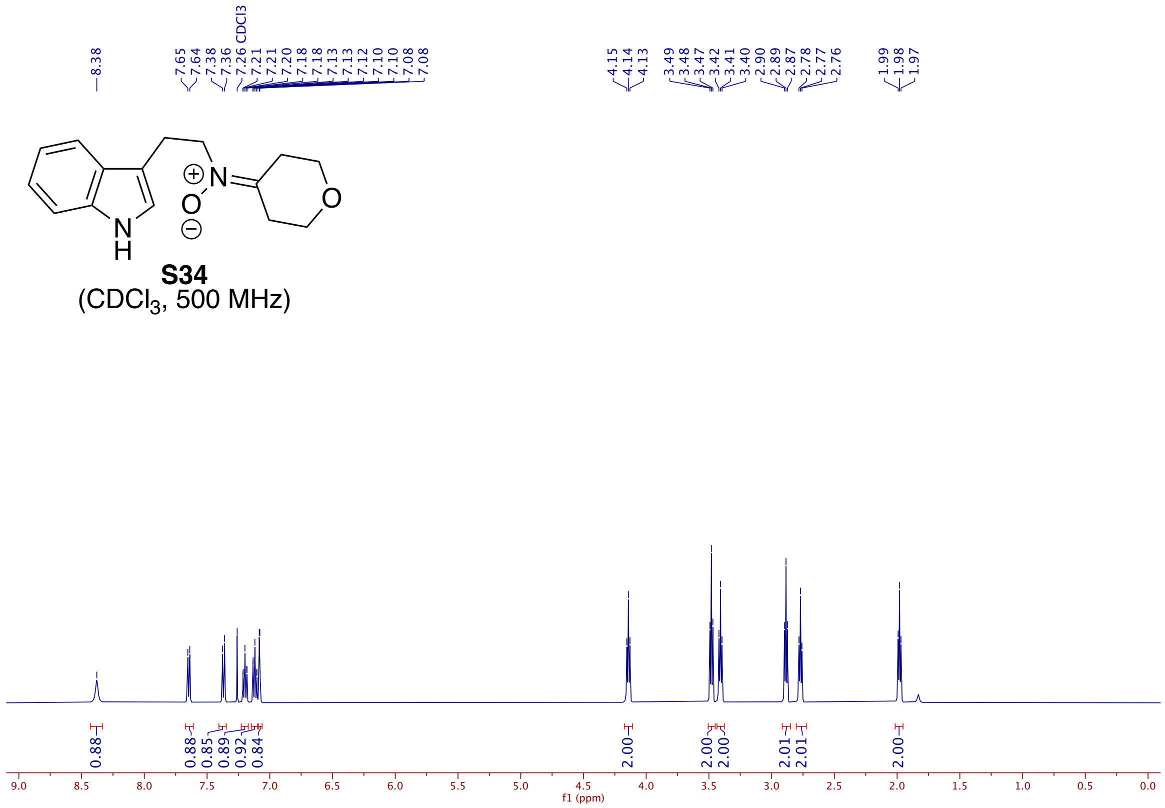
S34
(CDCl3, 500 MHz)
8.38
7.65
7.64
7.38
7.36
7.26 CDCl3
7.21
7.21
7.20
7.18
7.18
7.13
7.13
7.12
7.10
7.10
7.08
7.08
4.15
4.14
4.13
3.49
3.48
3.47
3.42
3.41
3.40
2.90
2.89
2.87
2.78
2.77
2.76
1.99
1.98
1.97
0.88
0.88
0.85
0.89
0.92
0.84
2.00
2.00
2.00
2.01
2.01
2.00
f1 (ppm)

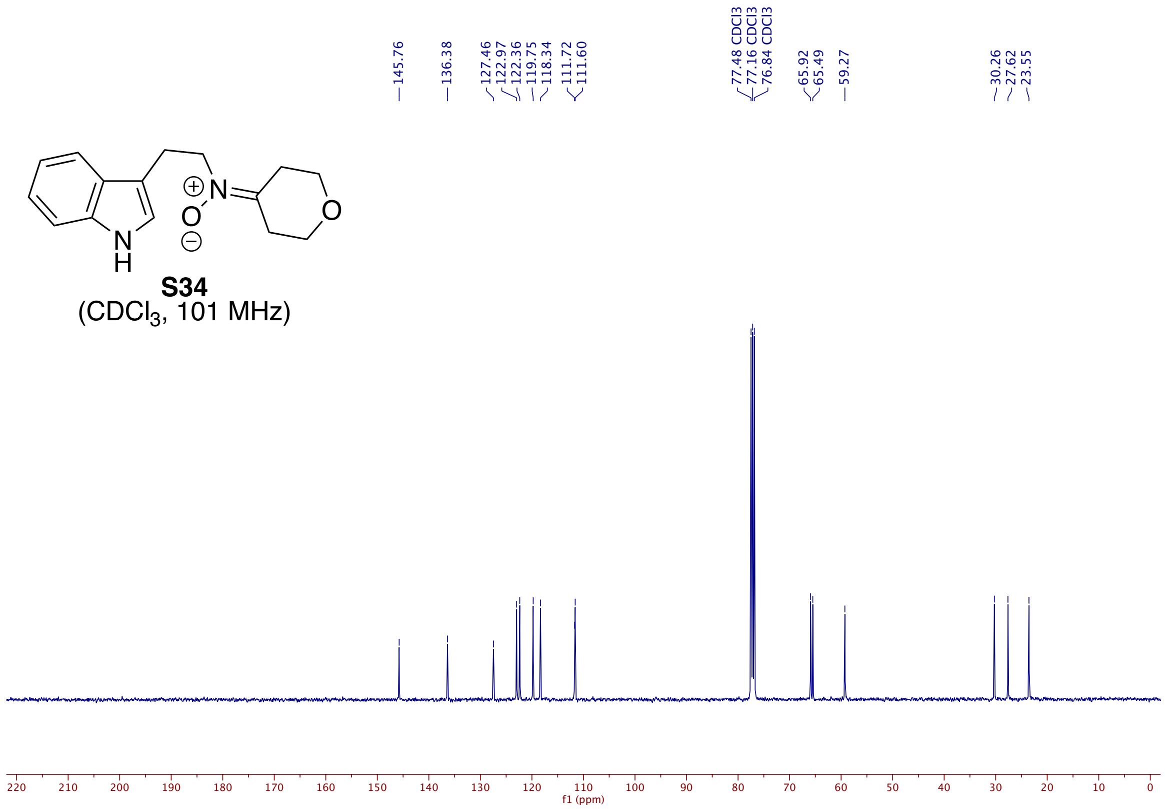
S34
(CDCl3, 101 MHz)
145.76
136.38
127.46
122.97
122.36
119.75
118.34
111.72
111.60
77.48 CDCl3
77.16 CDCl3
76.84 CDCl3
65.92
65.49
59.27
30.26
27.62
23.55
f1 (ppm)

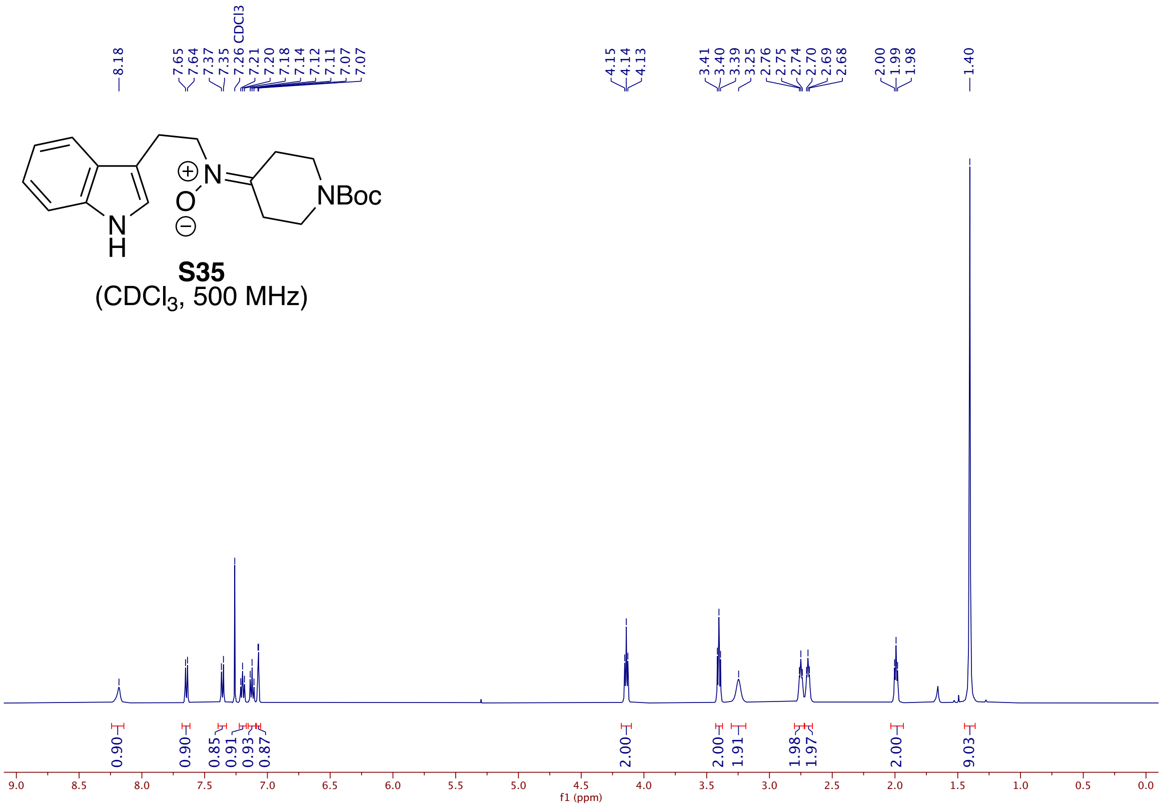
8.18
7.65
7.64
7.37
7.35
7.26 CDCl3
7.21
7.20
7.18
7.14
7.12
7.11
7.07
7.07
4.15
4.14
4.13
3.41
3.40
3.39
3.25
2.76
2.75
2.74
2.70
2.69
2.68
2.00
1.99
1.98
1.40
NBoc
N
O
N
H
S35
(CDCl3, 500 MHz)
0.90
0.90
0.85
0.91
0.93
0.87
2.00
2.00
1.91
1.98
1.97
2.00
9.03
f1 (ppm)

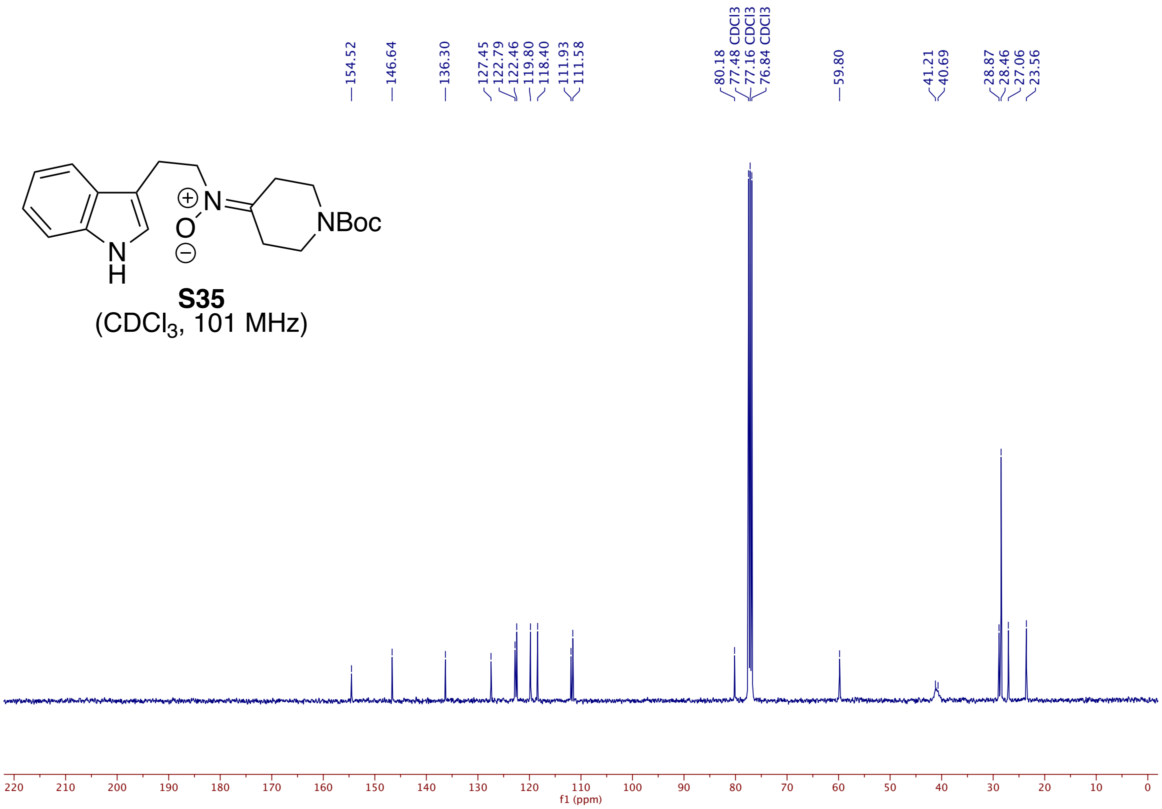
154.52
146.64
136.30
127.45
122.79
122.46
119.80
118.40
111.93
111.58
80.18
77.48 CDCl3
77.16 CDCl3
76.84 CDCl3
59.80
41.21
40.69
28.87
28.46
27.06
23.56
NBoc
N
O
N
H
S35
(CDCl3, 101 MHz)
f1 (ppm)

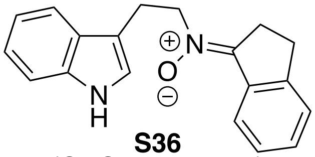

S36
($CDCl_3$, 500 MHz)

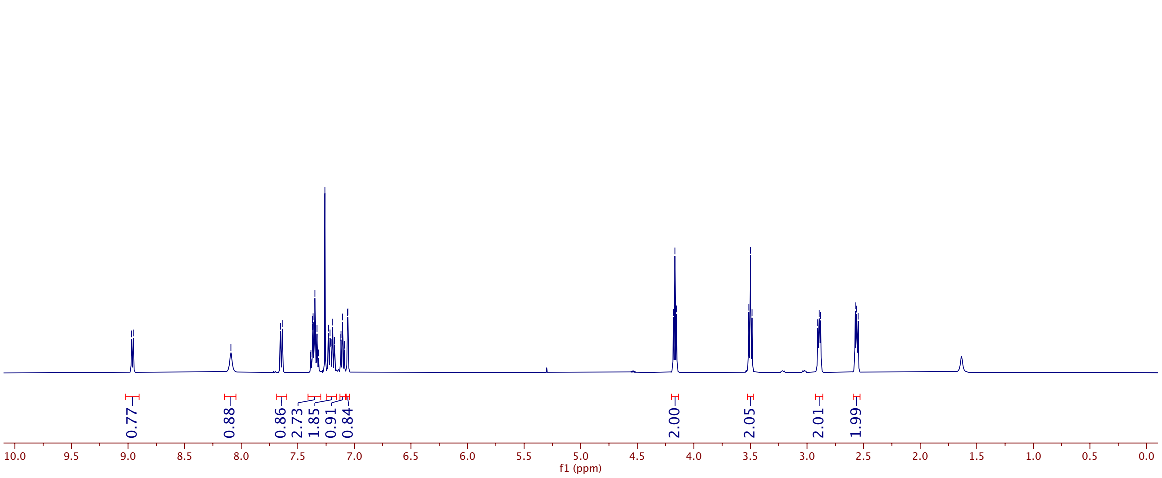

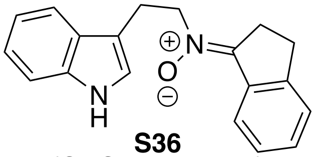

S36
($CDCl_3$, 101 MHz)

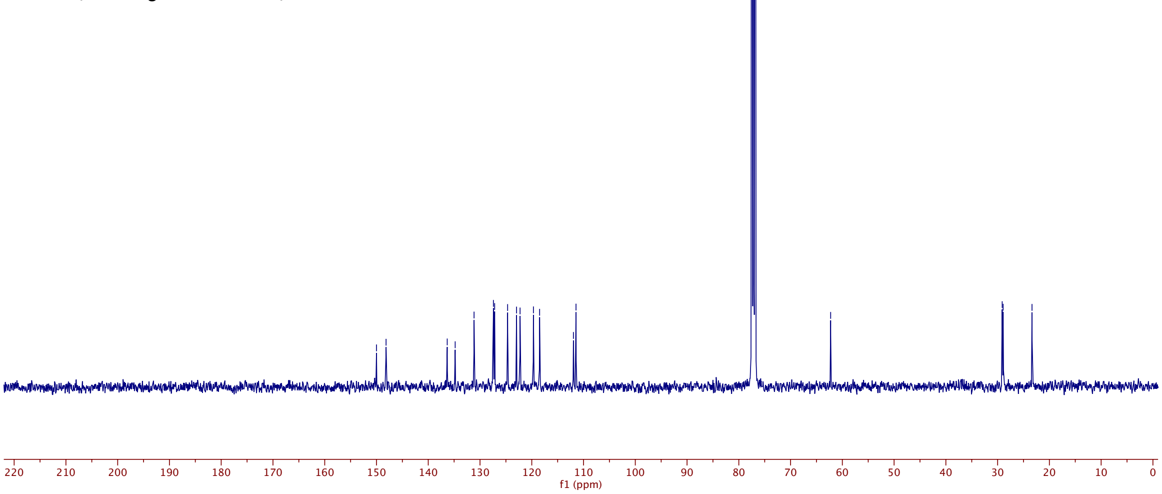

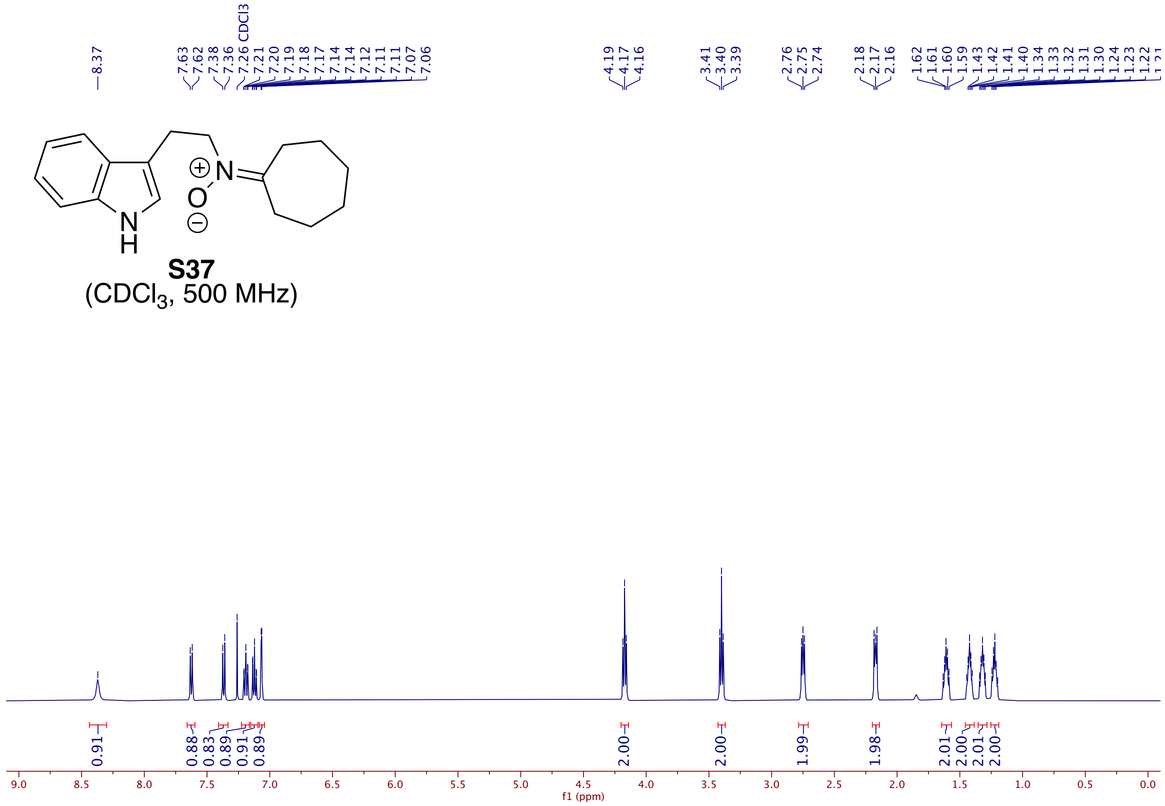

S37
($CDCl_3$, 500 MHz)

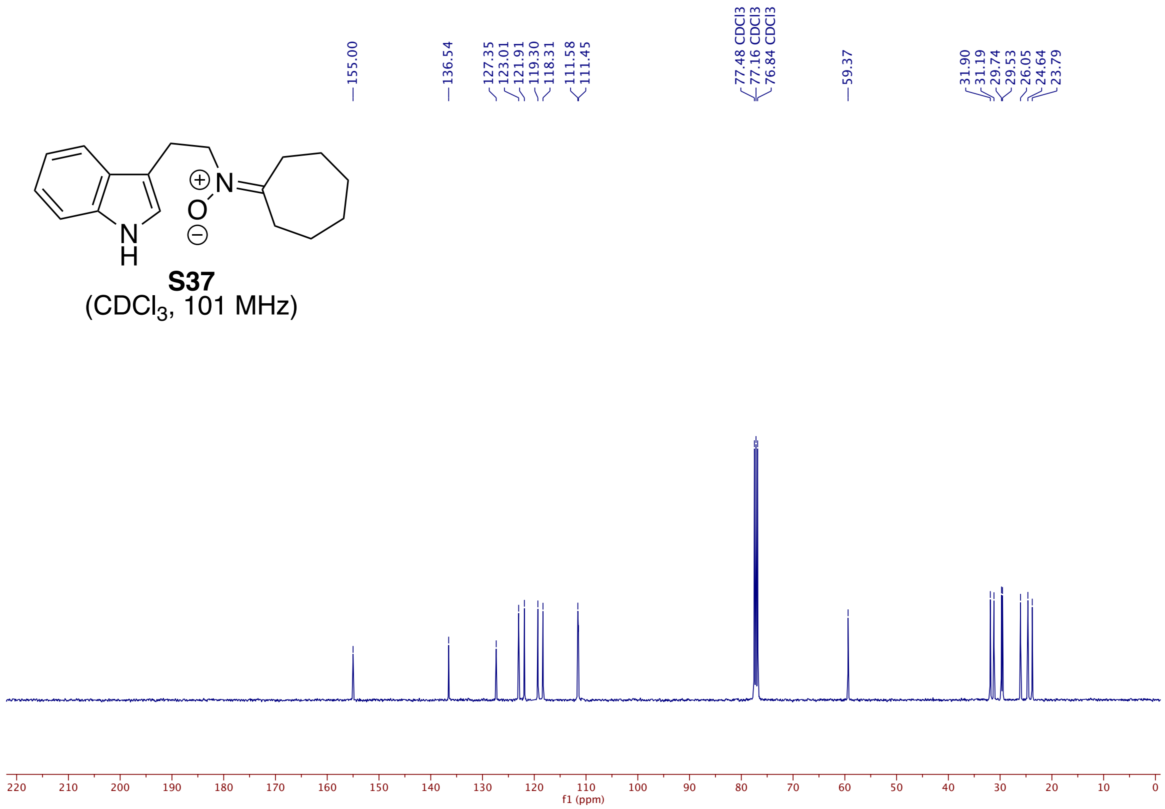

S37
($CDCl_3$, 101 MHz)

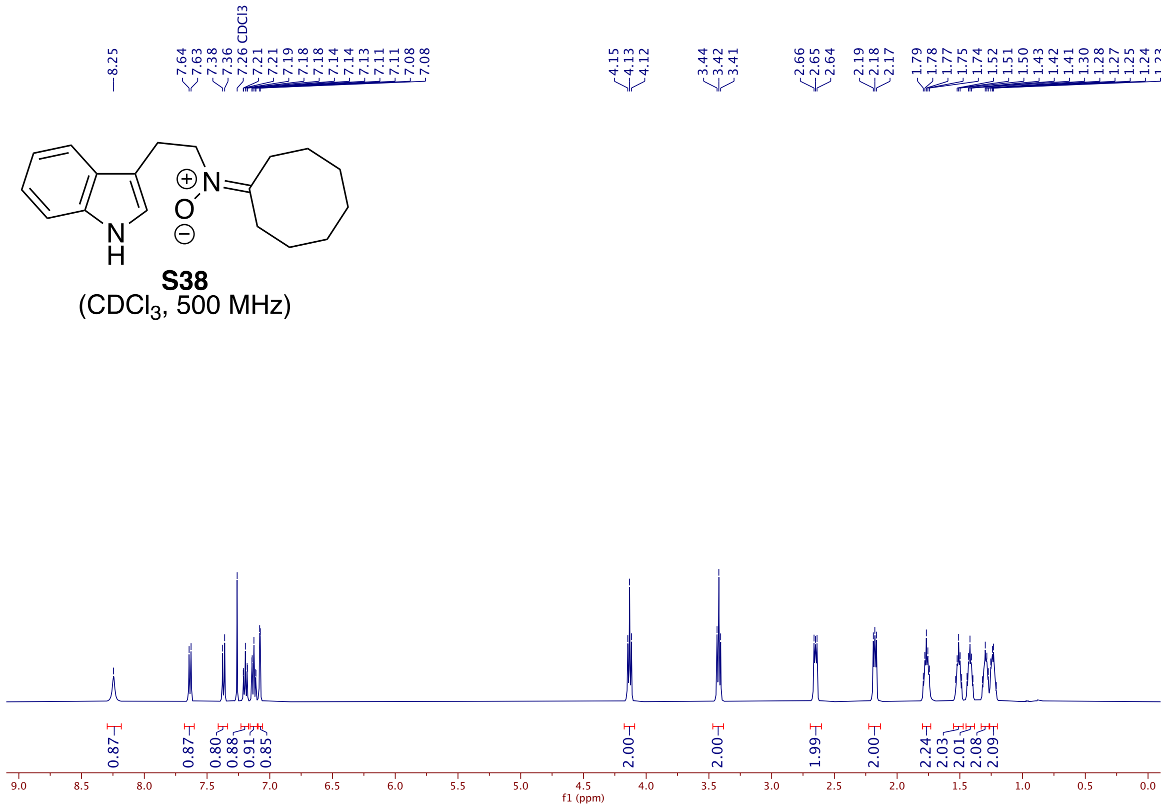

S38
(CDCl3, 500 MHz)

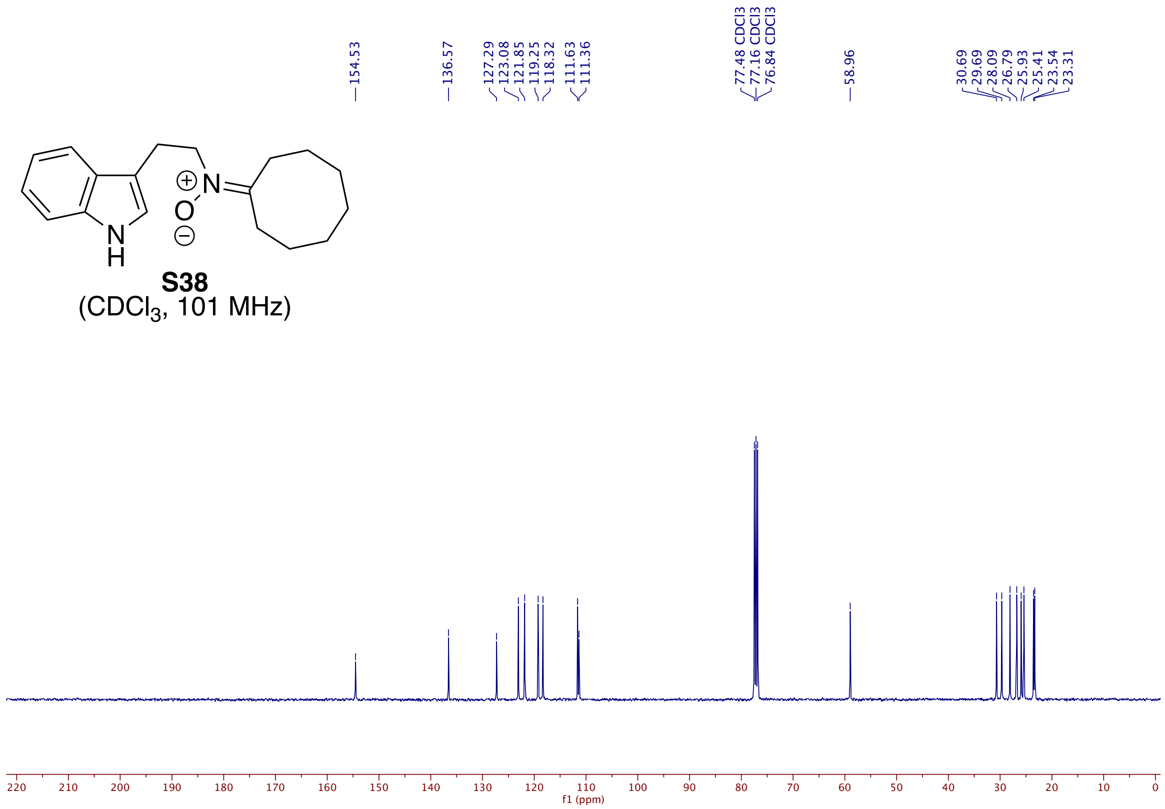

S38
(CDCl3, 101 MHz)

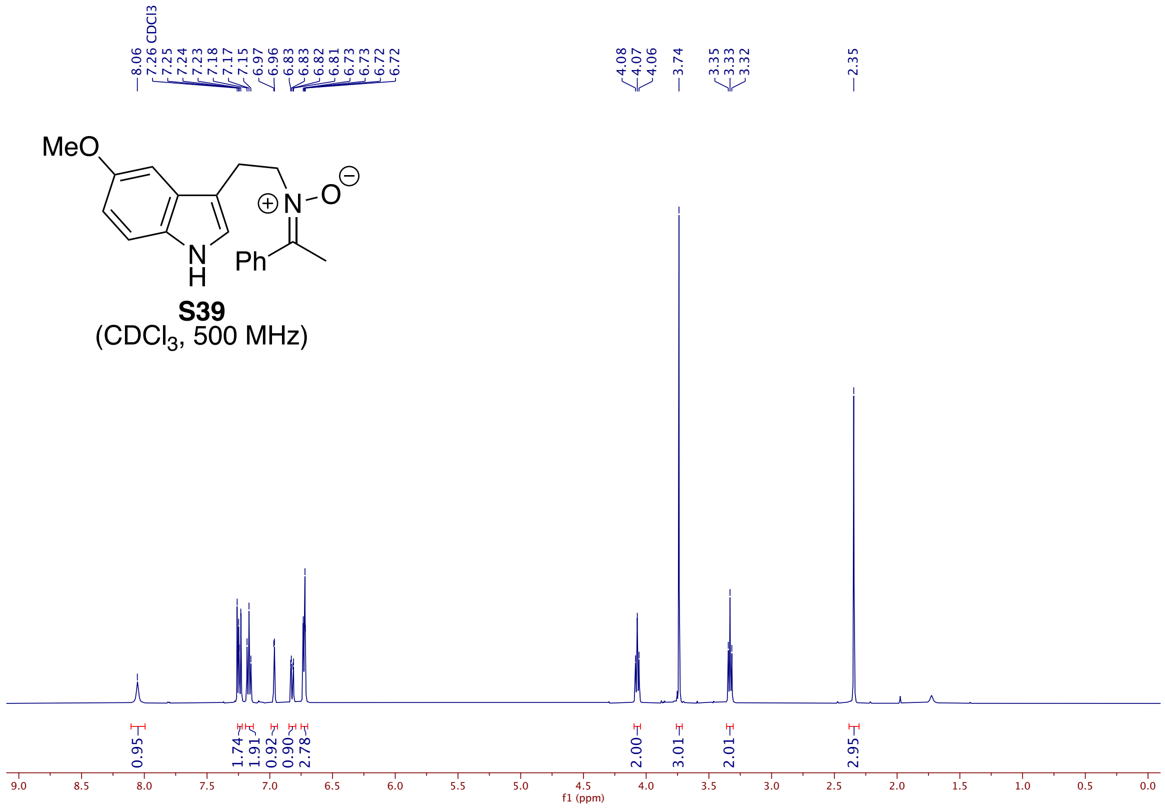
8.06
7.26 CDCl3
7.25
7.24
7.23
7.18
7.17
7.15
6.97
6.96
6.83
6.83
6.82
6.81
6.73
6.73
6.72
6.72
4.08
4.07
4.06
3.74
3.35
3.33
3.32
2.35
MeO
N
O
Ph
N
H
S39
(CDCl3, 500 MHz)
0.95
1.74
1.91
0.92
0.90
2.78
2.00
3.01
2.01
2.95
f1 (ppm)

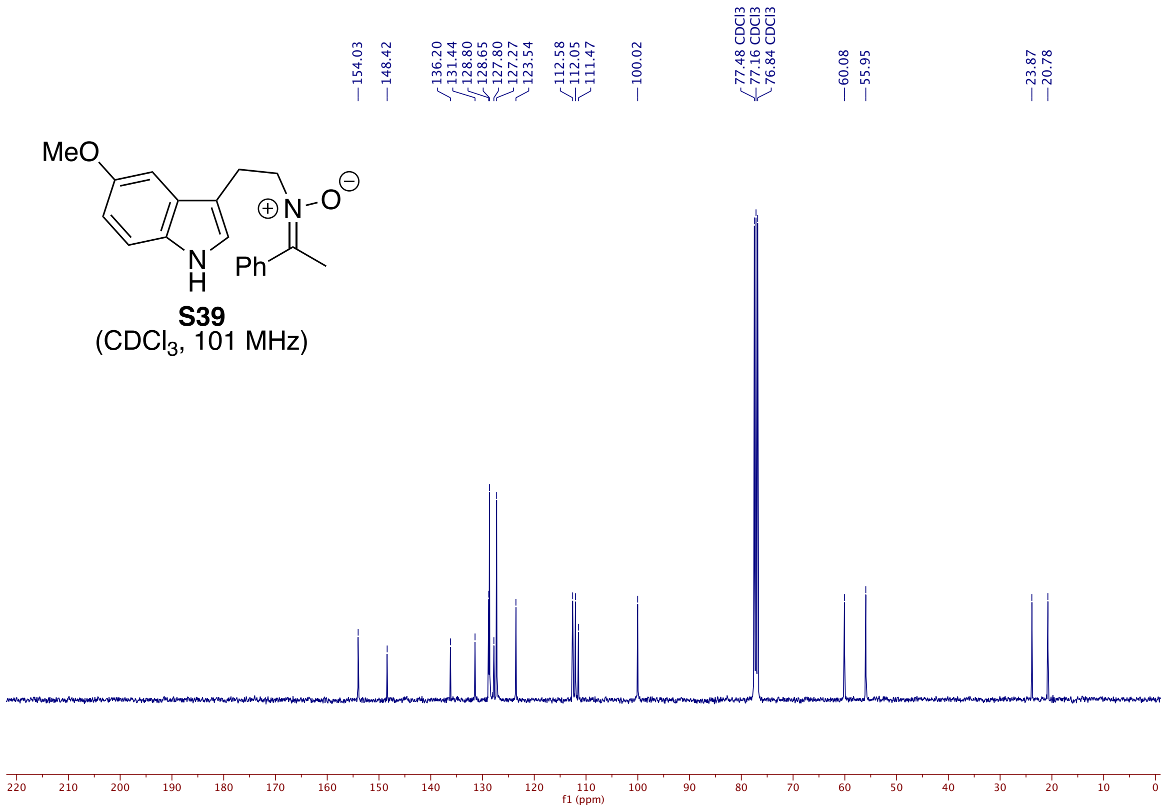
154.03
148.42
136.20
131.44
128.80
128.65
127.80
127.27
123.54
112.58
112.05
111.47
100.02
77.48 CDCl3
77.16 CDCl3
76.84 CDCl3
60.08
55.95
23.87
20.78
MeO
N
O
Ph
N
H
S39
(CDCl3, 101 MHz)
f1 (ppm)

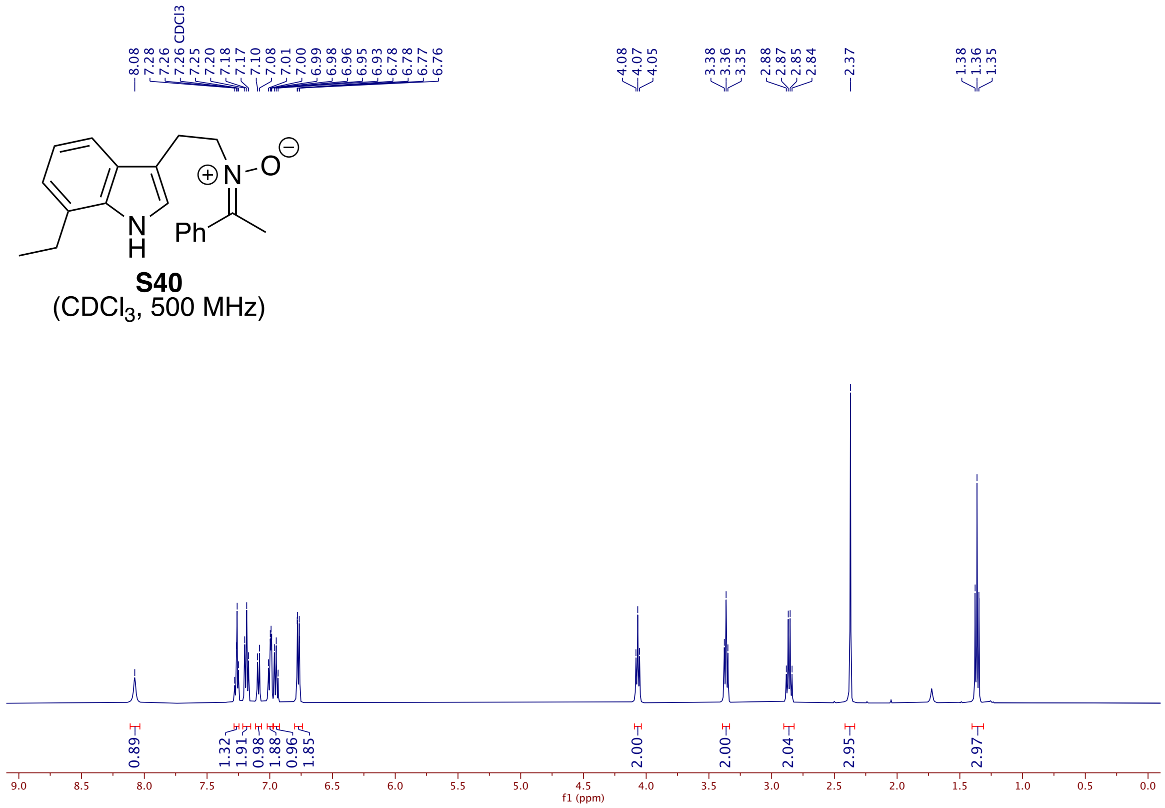
S40
(CDCl3, 500 MHz)

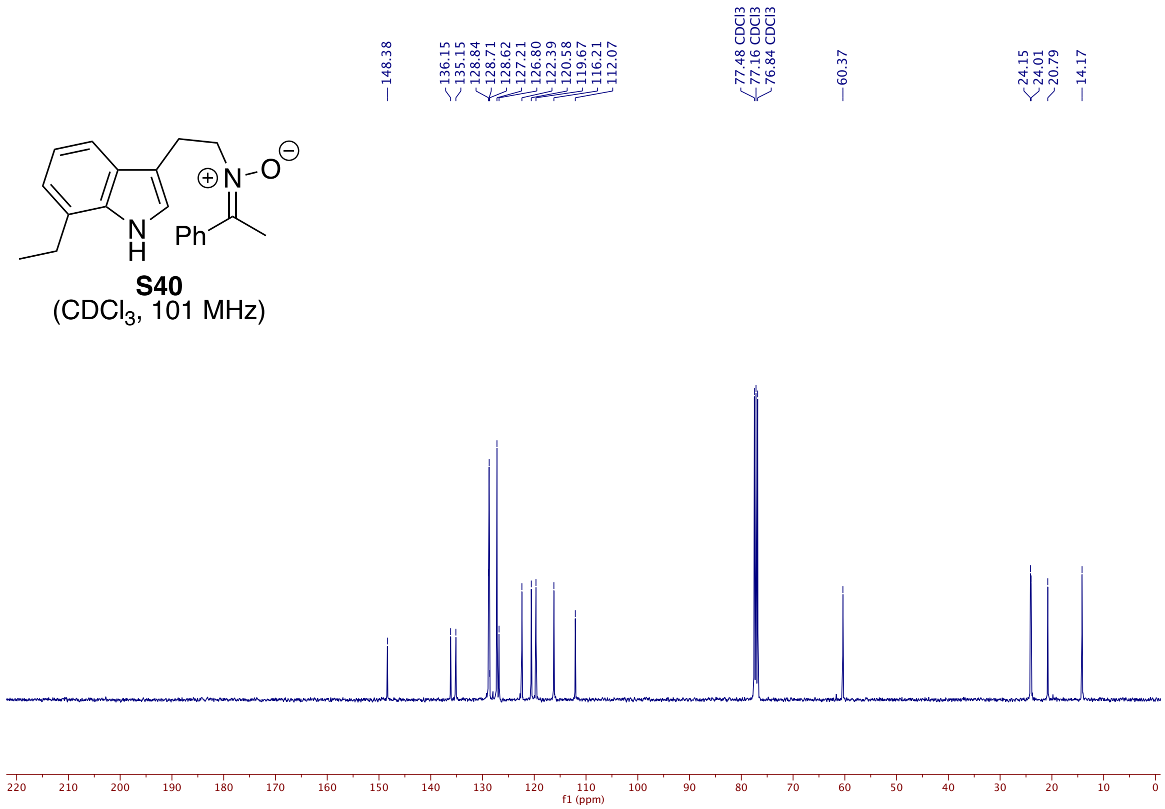
S40
(CDCl3, 101 MHz)

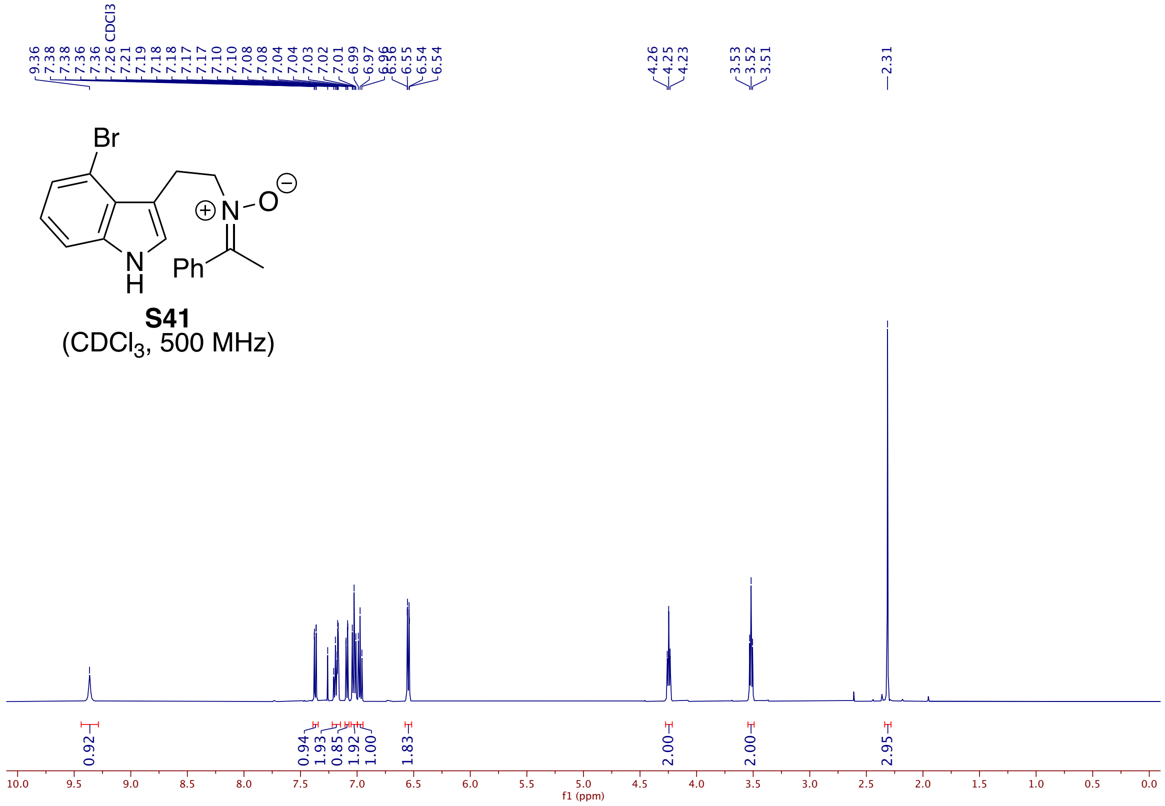

S41
($CDCl_3$, 500 MHz)

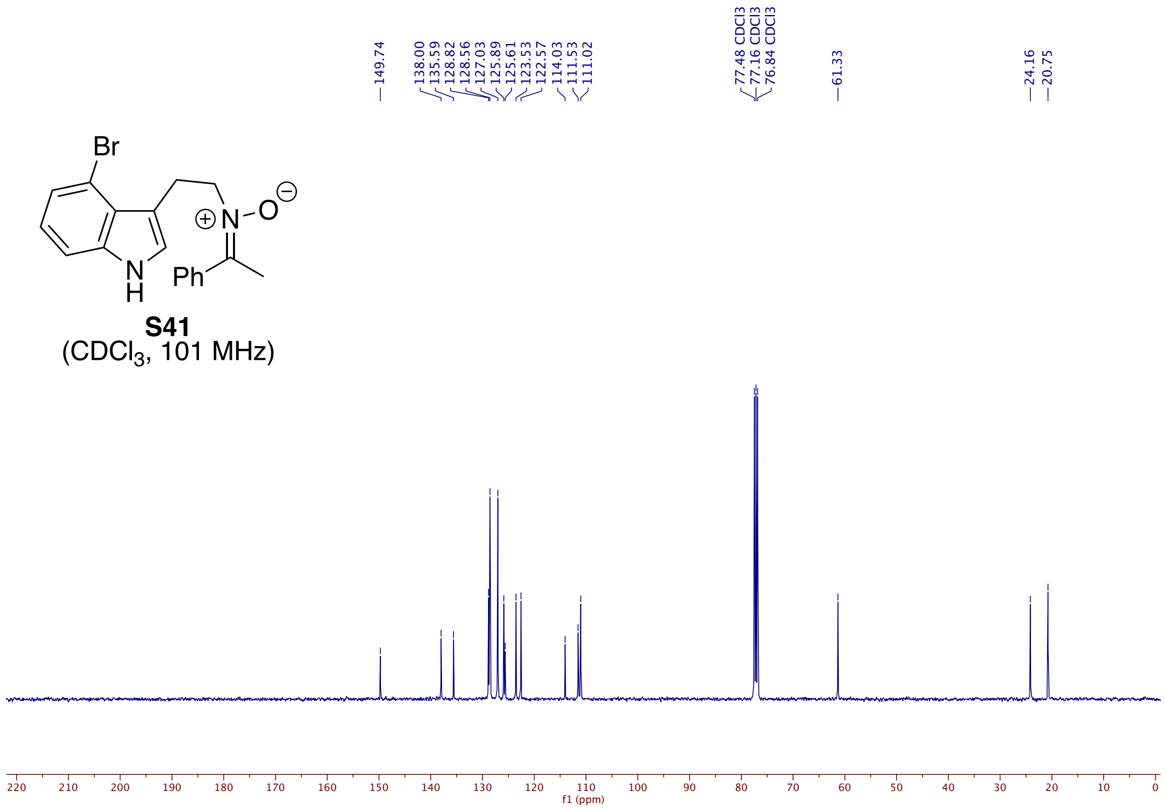

S41
($CDCl_3$, 101 MHz)

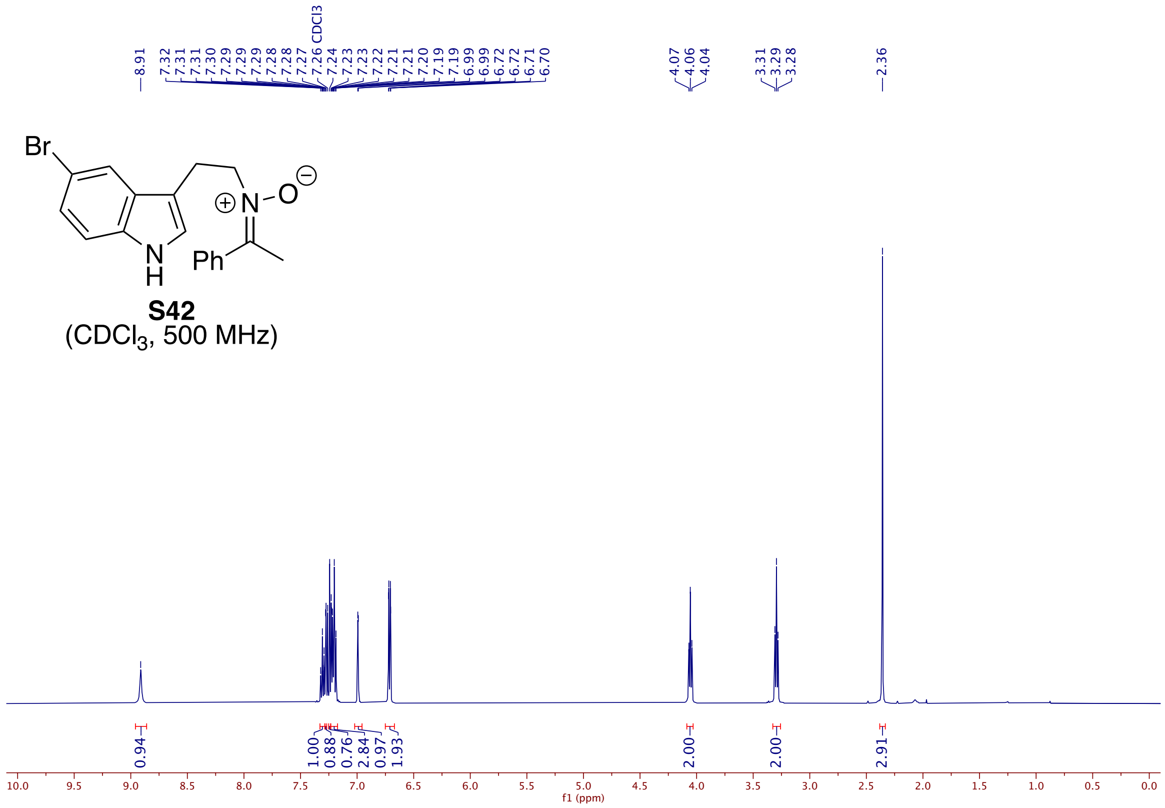
8.91
7.32
7.31
7.31
7.30
7.29
7.29
7.29
7.28
7.28
7.27
7.26 CDCl3
7.24
7.23
7.23
7.22
7.21
7.21
7.20
7.19
7.19
6.99
6.99
6.72
6.72
6.71
6.70
4.07
4.06
4.04
3.31
3.29
3.28
2.36
Br
N
O
N
H
Ph
S42
(CDCl3, 500 MHz)
0.94
1.00
0.88
0.76
2.84
0.97
1.93
2.00
2.00
2.91
f1 (ppm)

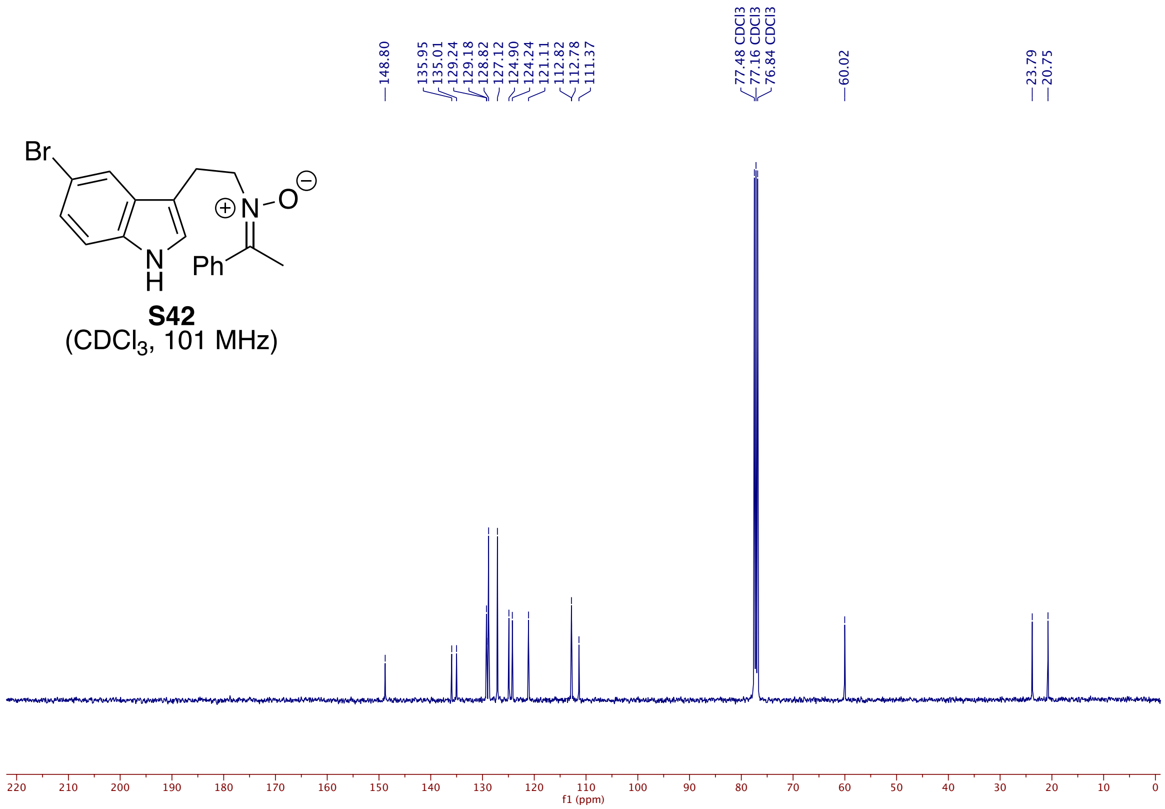
148.80
135.95
135.01
129.24
129.18
128.82
127.12
124.90
124.24
121.11
112.82
112.78
111.37
77.48 CDCl3
77.16 CDCl3
76.84 CDCl3
60.02
23.79
20.75
Br
N
O
N
H
Ph
S42
(CDCl3, 101 MHz)
f1 (ppm)

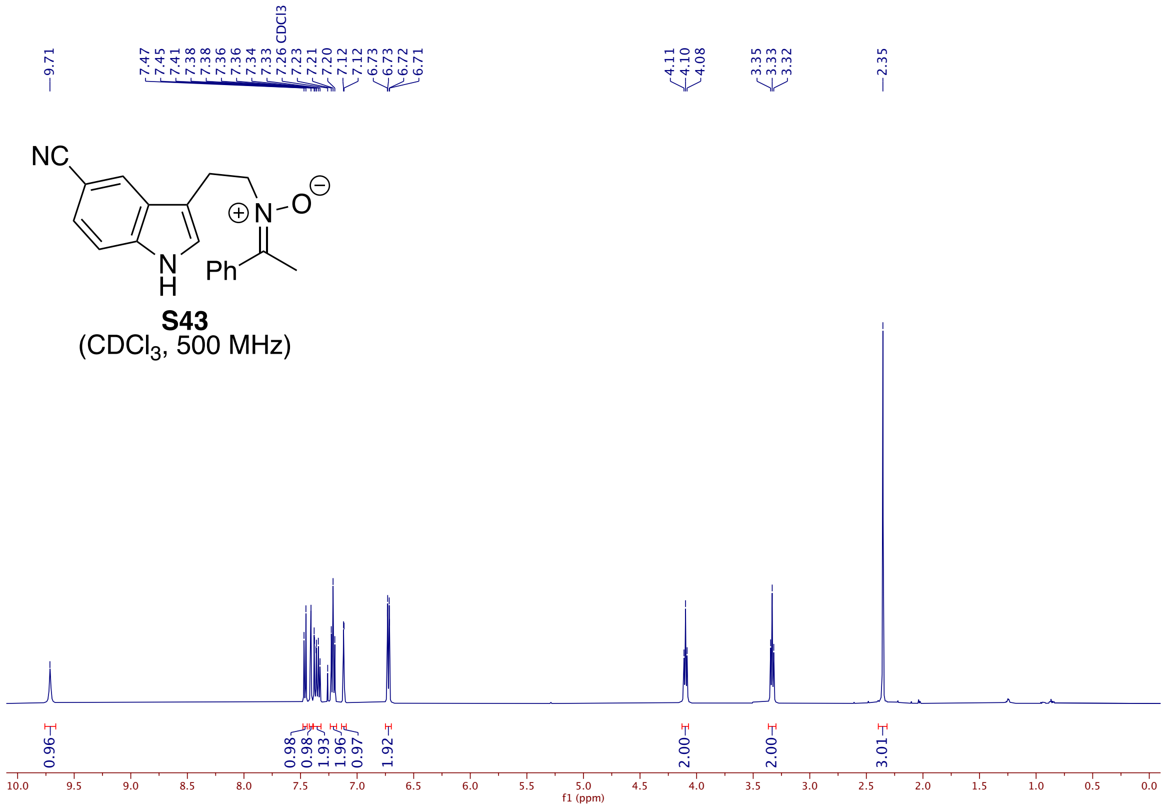
9.71
7.47
7.45
7.41
7.38
7.38
7.36
7.36
7.34
7.33
7.26 CDCl3
7.23
7.21
7.20
7.12
7.12
6.73
6.73
6.72
6.71
4.11
4.10
4.08
3.35
3.33
3.32
2.35
NC
N
H
N
O
Ph
S43
(CDCl$_3$, 500 MHz)
0.96
0.98
0.98
1.93
1.96
0.97
1.92
2.00
2.00
3.01
10.0 9.5 9.0 8.5 8.0 7.5 7.0 6.5 6.0 5.5 5.0 4.5 4.0 3.5 3.0 2.5 2.0 1.5 1.0 0.5 0.0
f1 (ppm)

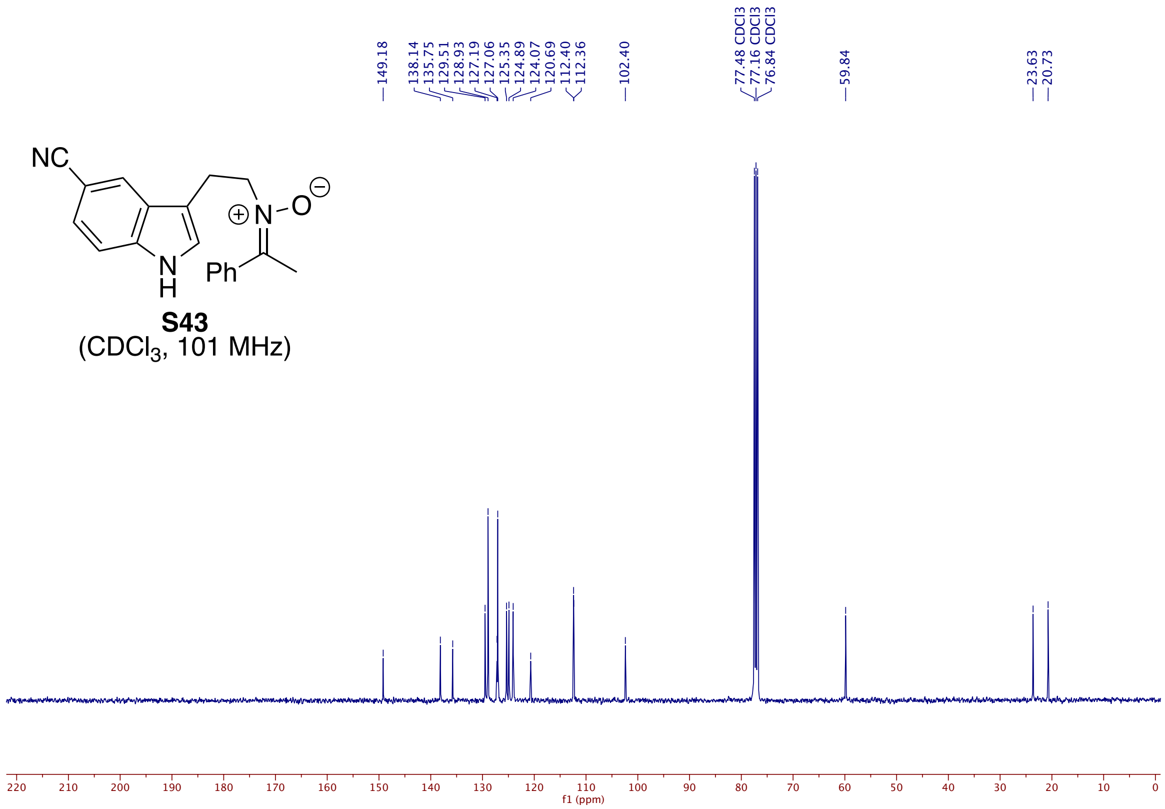
149.18
138.14
135.75
129.51
128.93
127.19
127.06
125.35
124.89
124.07
120.69
112.40
112.36
102.40
77.48 CDCl3
77.16 CDCl3
76.84 CDCl3
59.84
23.63
20.73
NC
N
H
N
O
Ph
S43
(CDCl$_3$, 101 MHz)
220 210 200 190 180 170 160 150 140 130 120 110 100 90 80 70 60 50 40 30 20 10 0
f1 (ppm)

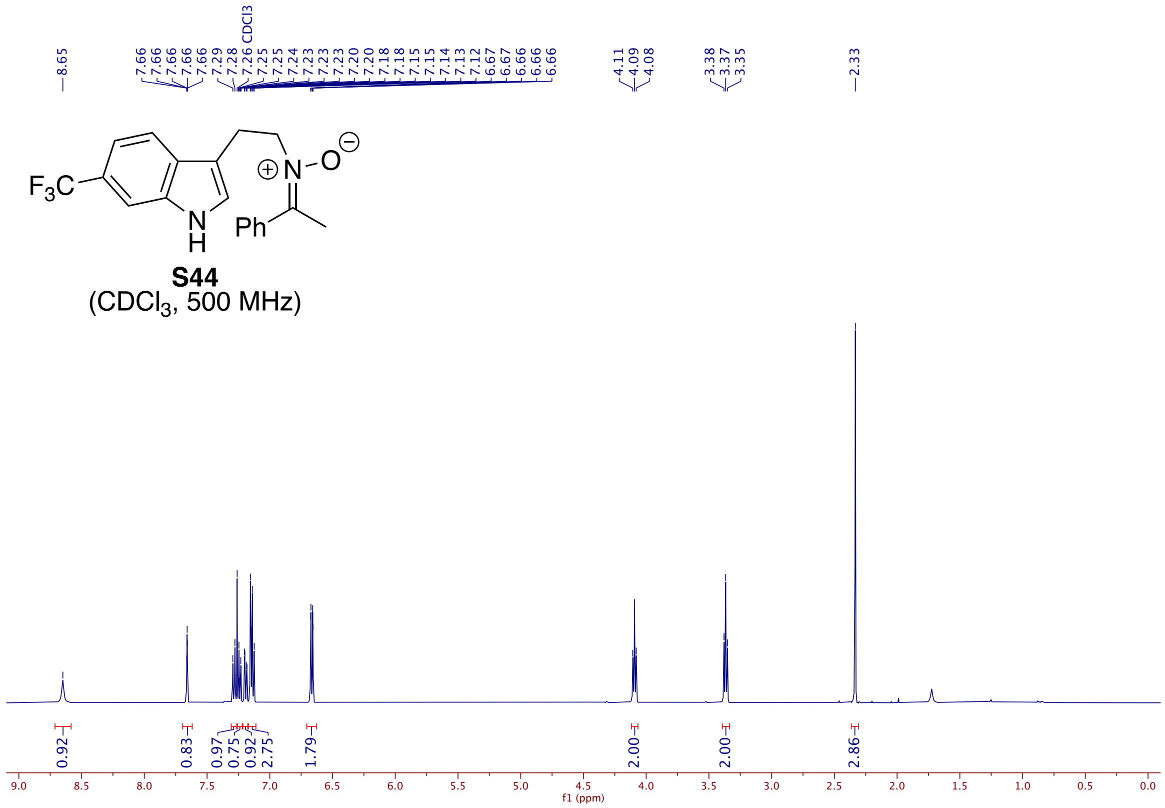
8.65
7.66 7.66 7.66 7.66 7.66 7.29 7.28 7.26 CDCl3 7.25 7.25 7.24 7.23 7.23 7.23 7.20 7.20 7.18 7.18 7.15 7.15 7.14 7.13 7.12 6.67 6.67 6.66 6.66 6.66
4.11 4.09 4.08
3.38 3.37 3.35
2.33
F3C
N
H
Ph
N
O
S44
(CDCl3, 500 MHz)
0.92 0.83 0.97 0.75 0.92 2.75 1.79 2.00 2.00 2.86
9.0 8.5 8.0 7.5 7.0 6.5 6.0 5.5 5.0 4.5 4.0 3.5 3.0 2.5 2.0 1.5 1.0 0.5 0.0
f1 (ppm)

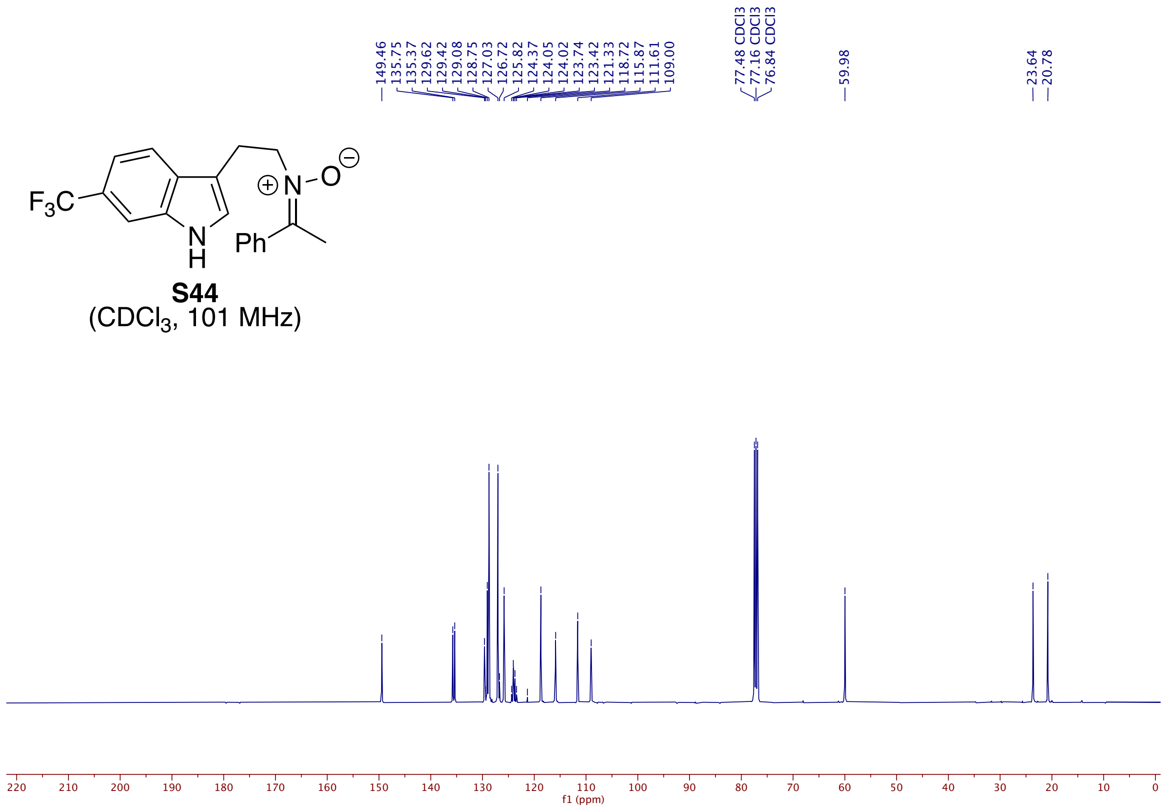
149.46 135.75 135.37 129.62 129.42 129.08 128.75 127.03 126.72 125.82 124.37 124.05 124.02 123.74 123.42 121.33 118.72 115.87 111.61 109.00
77.48 CDCl3 77.16 CDCl3 76.84 CDCl3
59.98
23.64 20.78
F3C
N
H
Ph
N
O
S44
(CDCl3, 101 MHz)
220 210 200 190 180 170 160 150 140 130 120 110 100 90 80 70 60 50 40 30 20 10 0
f1 (ppm)

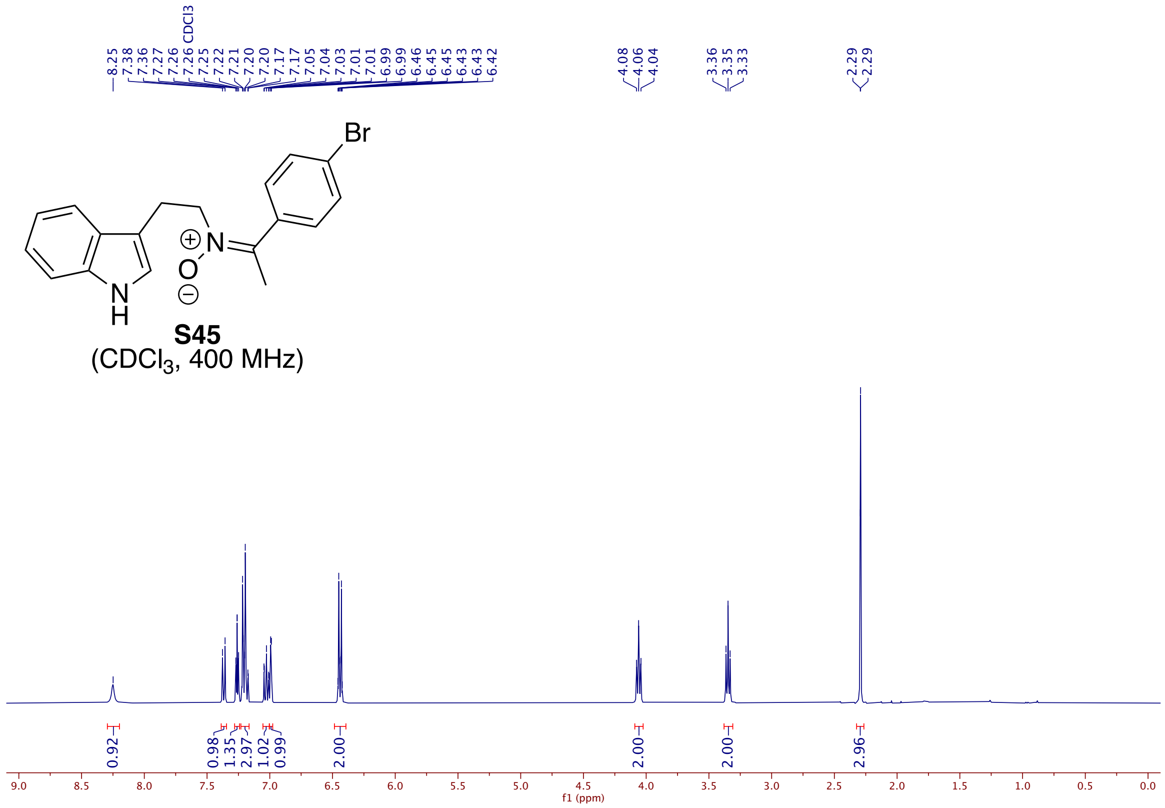
8.25
7.38
7.36
7.27
7.26 CDCl3
7.25
7.22
7.21
7.20
7.20
7.17
7.17
7.05
7.04
7.03
7.01
6.99
6.99
6.46
6.45
6.45
6.43
6.43
6.42
4.08
4.06
4.04
3.36
3.35
3.33
2.29
2.29
Br
N
O
N
H
S45
(CDCl3, 400 MHz)
0.92
0.98
1.35
2.97
1.02
0.99
2.00
2.00
2.00
2.96
f1 (ppm)

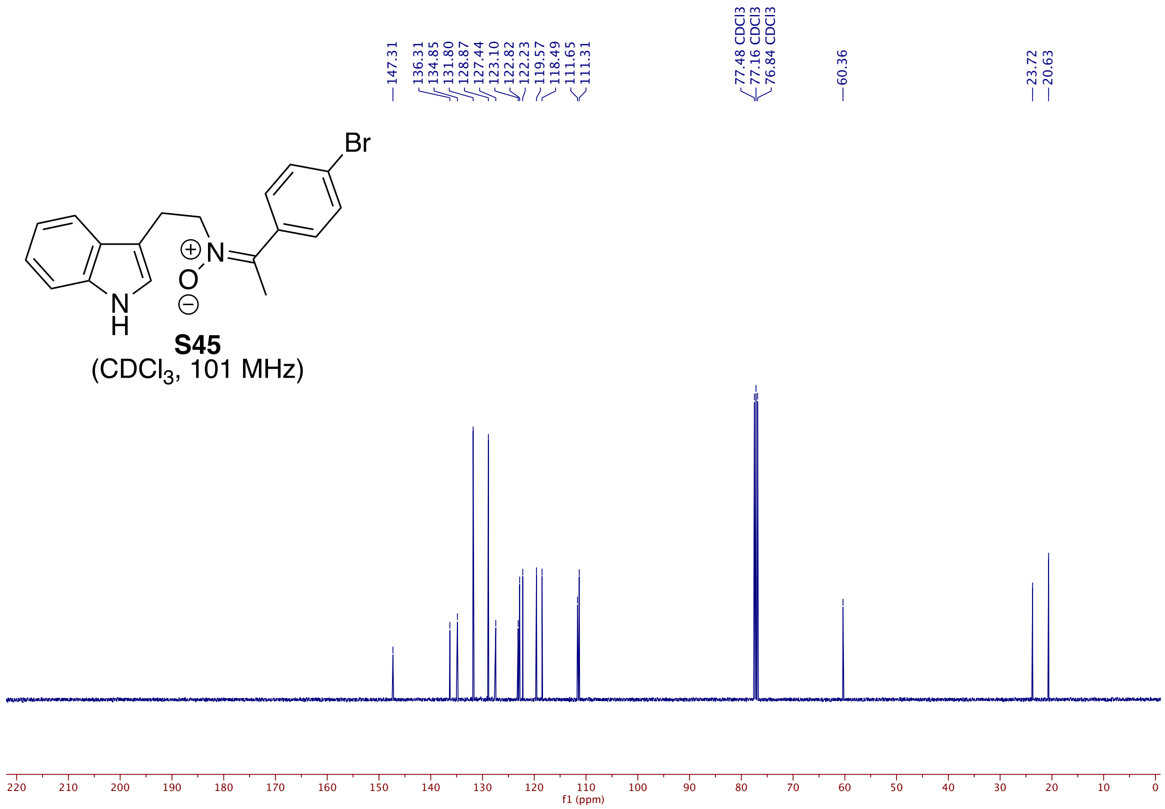
147.31
136.31
134.85
131.80
128.87
127.44
123.10
122.82
122.23
119.57
118.49
111.65
111.31
77.48 CDCl3
77.16 CDCl3
76.84 CDCl3
60.36
23.72
20.63
Br
N
O
N
H
S45
(CDCl3, 101 MHz)
f1 (ppm)

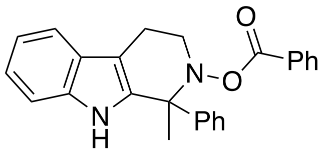

20a
($CDCl_3$, 500 MHz)

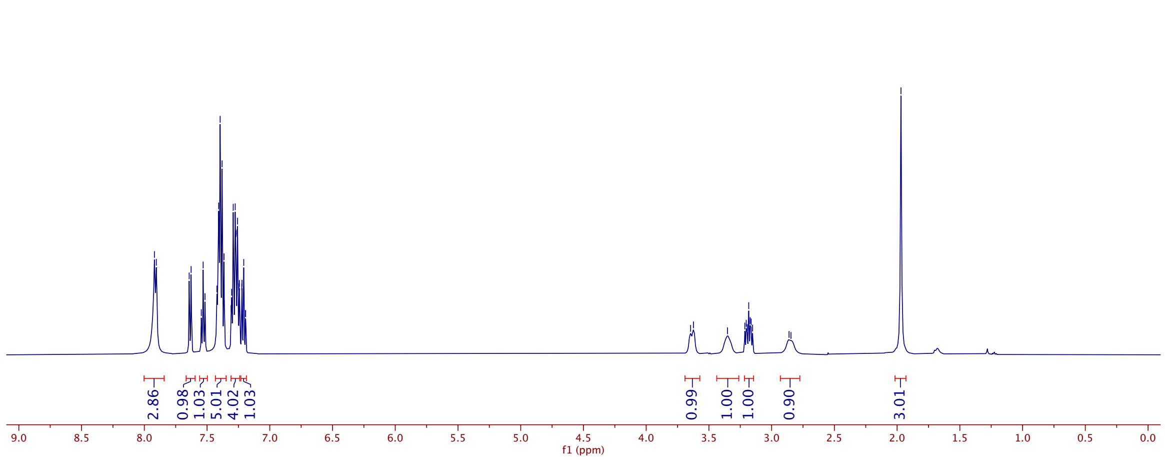

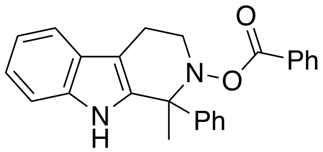

20a
($CDCl_3$, 101 MHz)

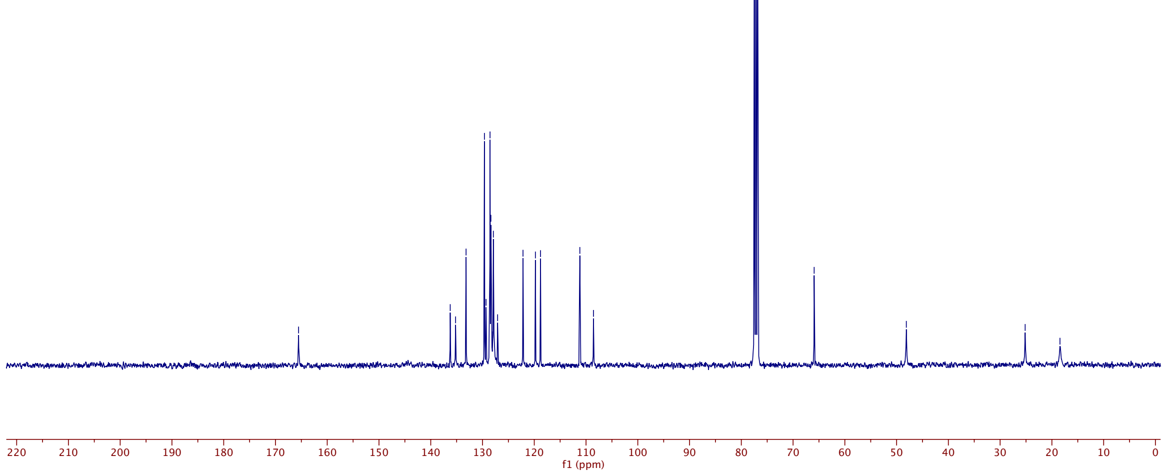

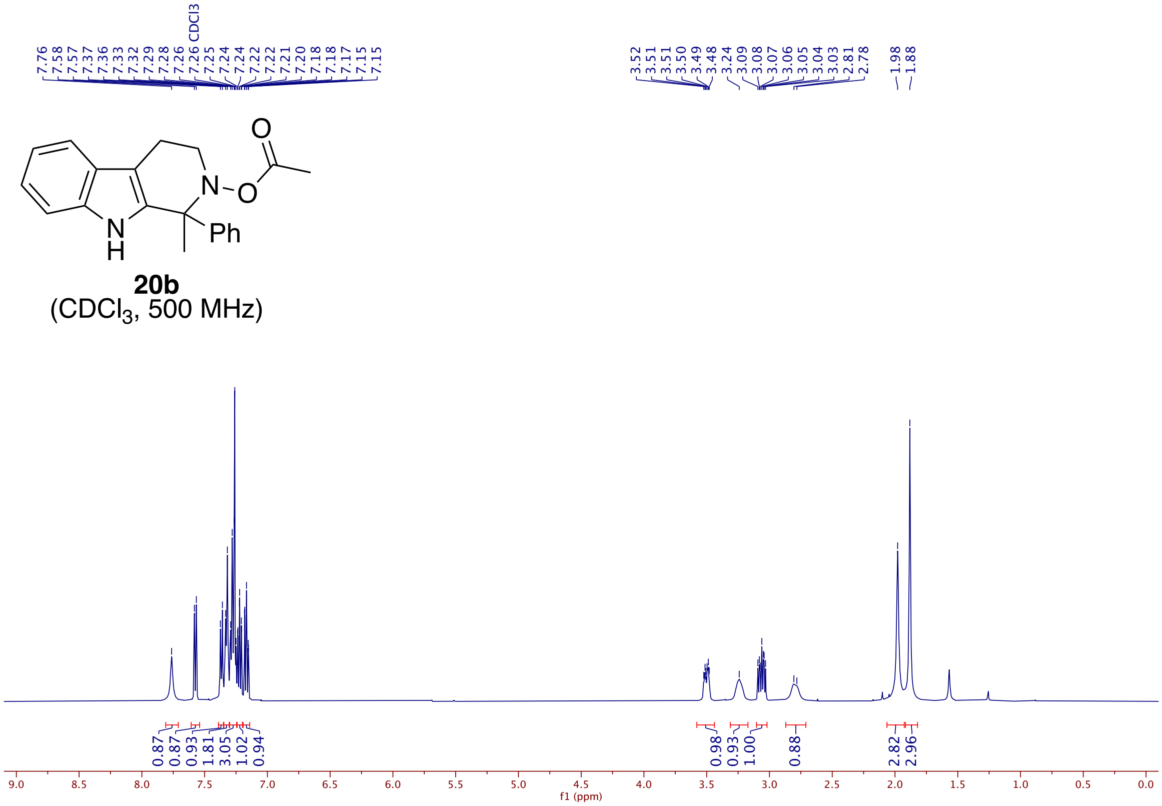
7.76
7.58
7.57
7.37
7.36
7.33
7.32
7.29
7.28
7.26 CDCl3
7.25
7.24
7.22
7.22
7.21
7.20
7.18
7.18
7.17
7.15
7.15
3.52
3.51
3.51
3.50
3.49
3.48
3.24
3.09
3.08
3.07
3.06
3.05
3.04
3.03
2.81
2.78
1.98
1.88
O
N
O
N
H
Ph
20b
(CDCl3, 500 MHz)
0.87
0.87
0.93
1.81
3.05
1.02
0.94
0.98
0.93
1.00
0.88
2.82
2.96
9.0
8.5
8.0
7.5
7.0
6.5
6.0
5.5
5.0
4.5
4.0
3.5
3.0
2.5
2.0
1.5
1.0
0.5
0.0
f1 (ppm)

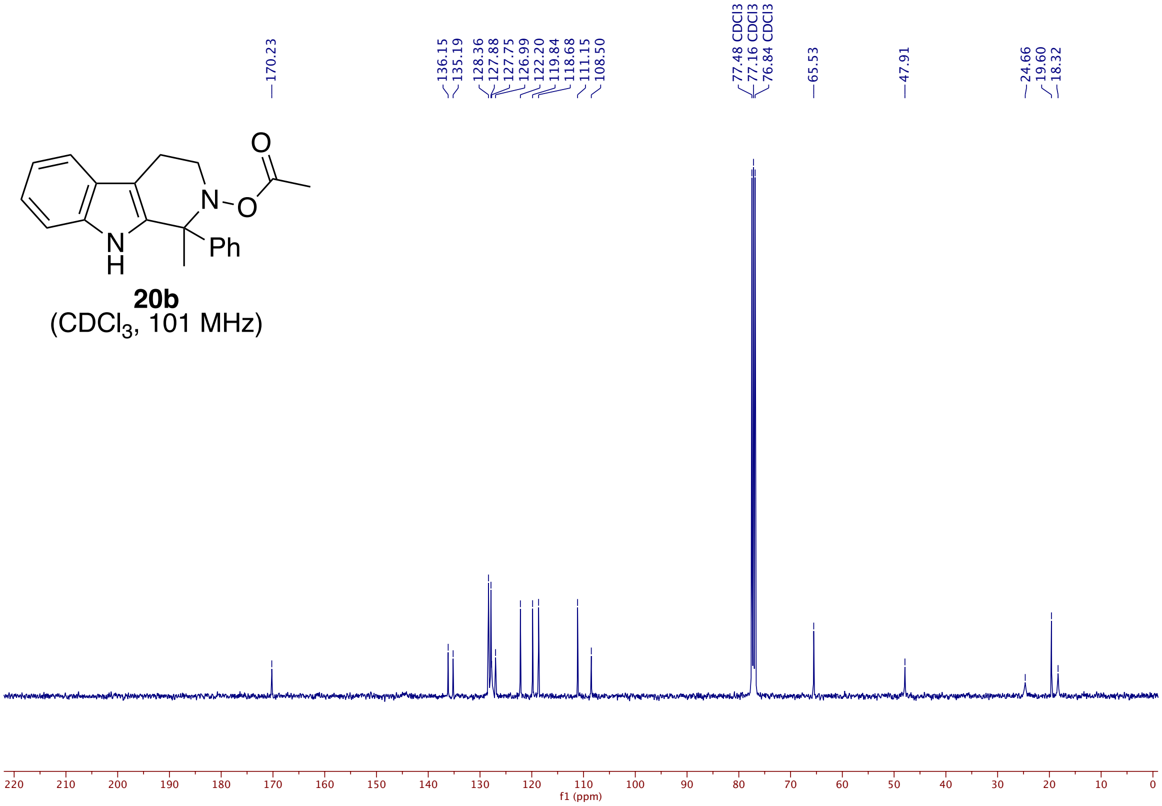
170.23
136.15
135.19
128.36
127.88
127.75
126.99
122.20
119.84
118.68
111.15
108.50
77.48 CDCl3
77.16 CDCl3
76.84 CDCl3
65.53
47.91
24.66
19.60
18.32
O
N
O
N
H
Ph
20b
(CDCl3, 101 MHz)
220
210
200
190
180
170
160
150
140
130
120
110
100
90
80
70
60
50
40
30
20
10
0
f1 (ppm)

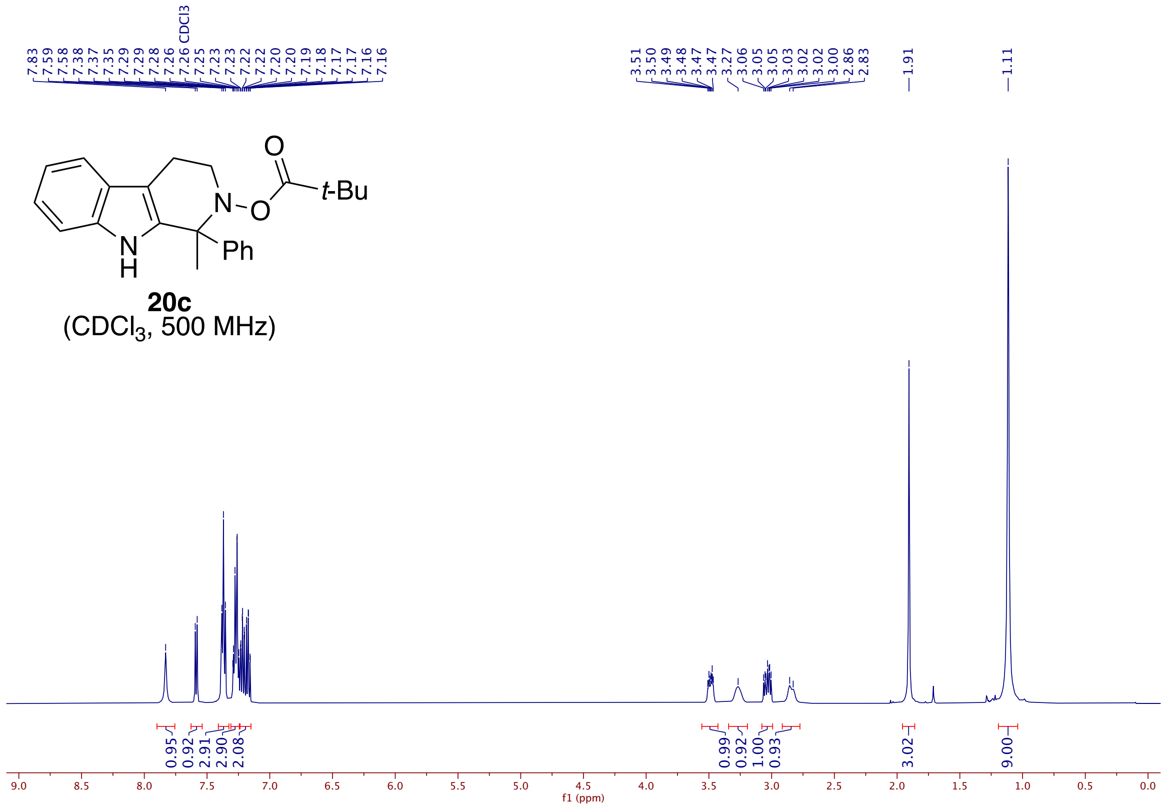

7.83
7.59
7.58
7.38
7.37
7.35
7.29
7.29
7.28
7.26
7.26 CDCl3
7.25
7.23
7.23
7.22
7.22
7.20
7.20
7.19
7.18
7.17
7.17
7.16
7.16
3.51
3.50
3.49
3.48
3.47
3.47
3.27
3.06
3.05
3.05
3.03
3.02
3.02
3.00
2.86
2.83
1.91
1.11
O
t-Bu
N
O
N
H
Ph
20c
(CDCl3, 500 MHz)
0.95
0.92
2.91
2.90
2.08
0.99
0.92
1.00
0.93
3.02
9.00
f1 (ppm)

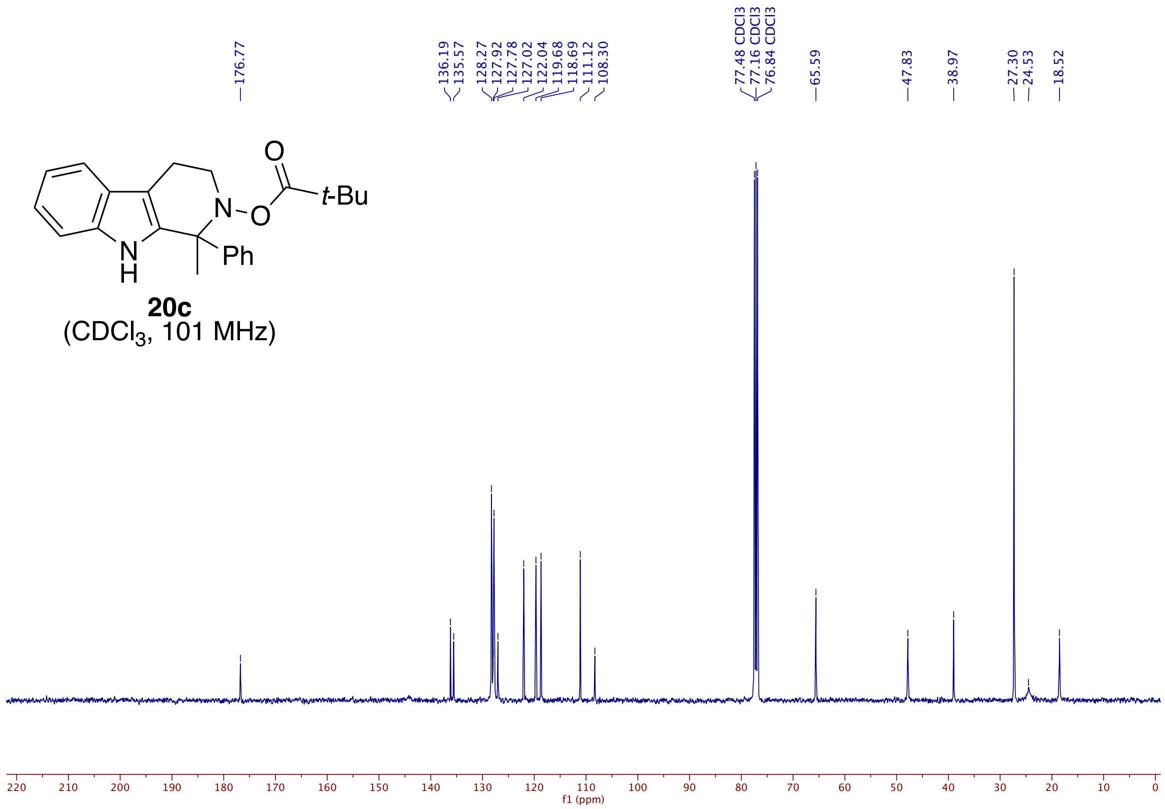

176.77
136.19
135.57
128.27
127.92
127.78
127.02
122.04
119.68
118.69
111.12
108.30
77.48 CDCl3
77.16 CDCl3
76.84 CDCl3
65.59
47.83
38.97
27.30
24.53
18.52
O
t-Bu
N
O
N
H
Ph
20c
(CDCl3, 101 MHz)
f1 (ppm)

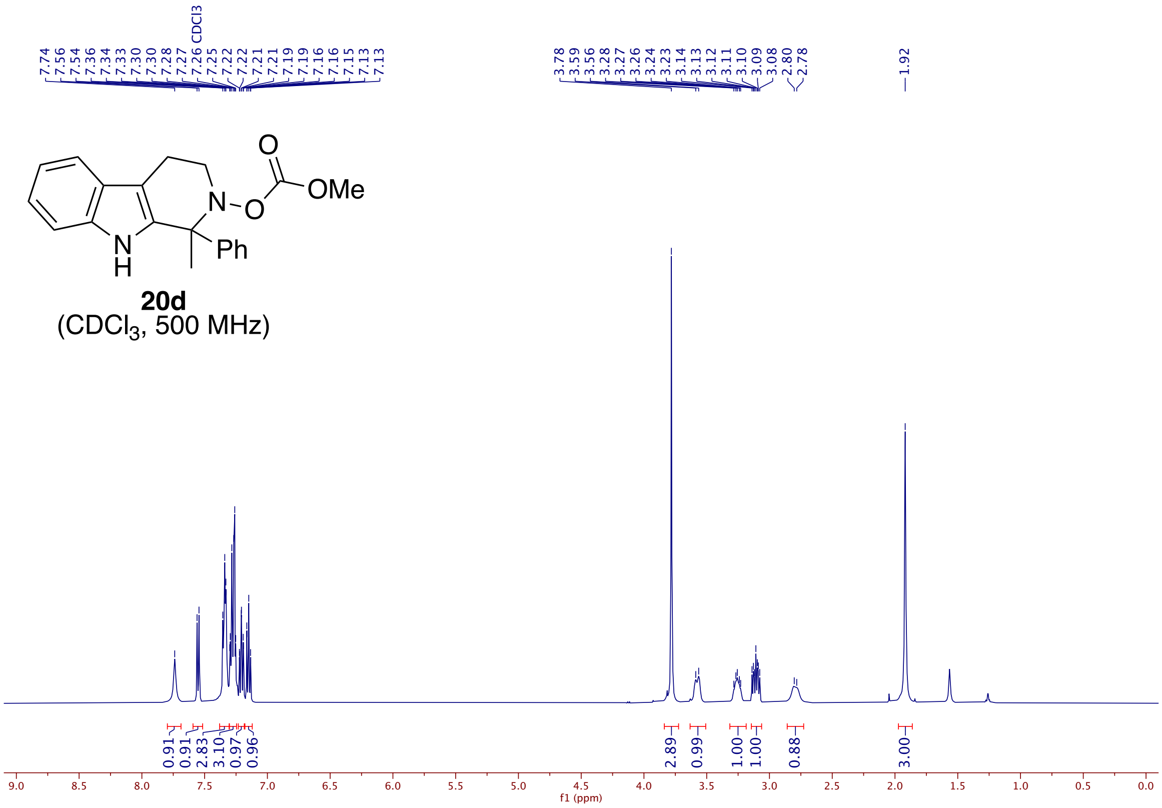

20d
($CDCl_3$, 500 MHz)

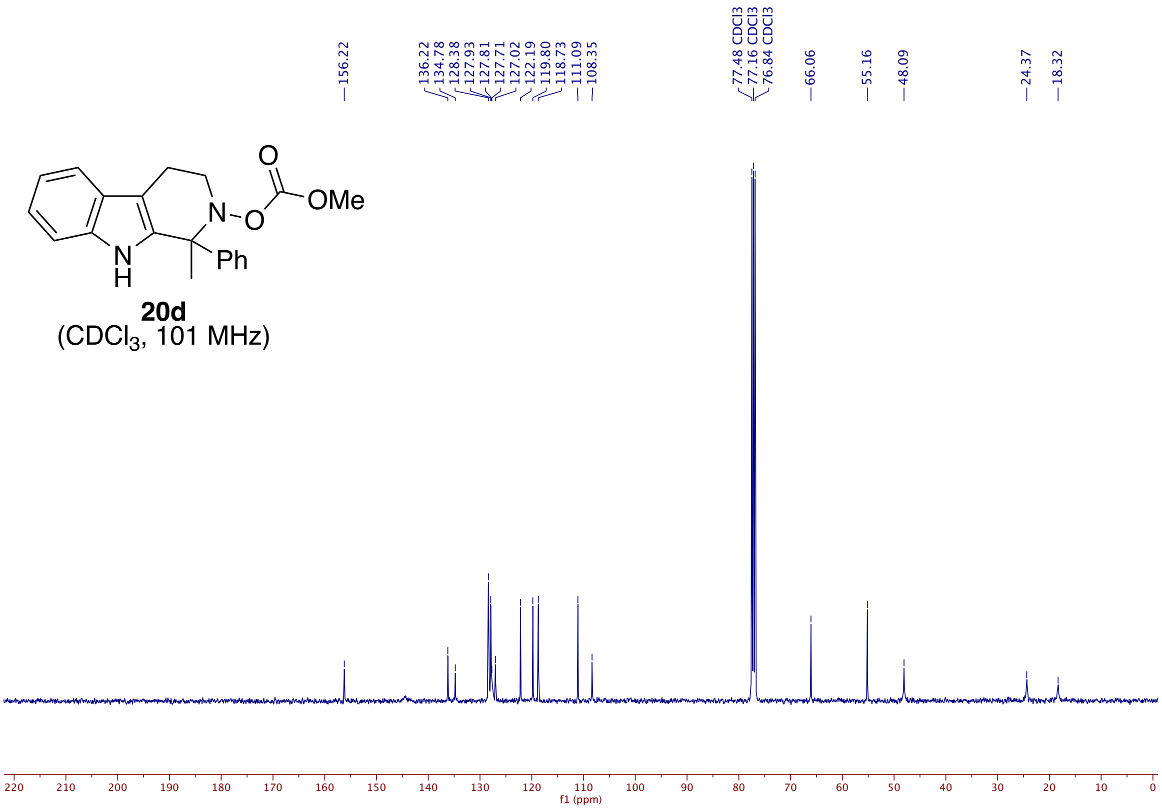

20d
($CDCl_3$, 101 MHz)

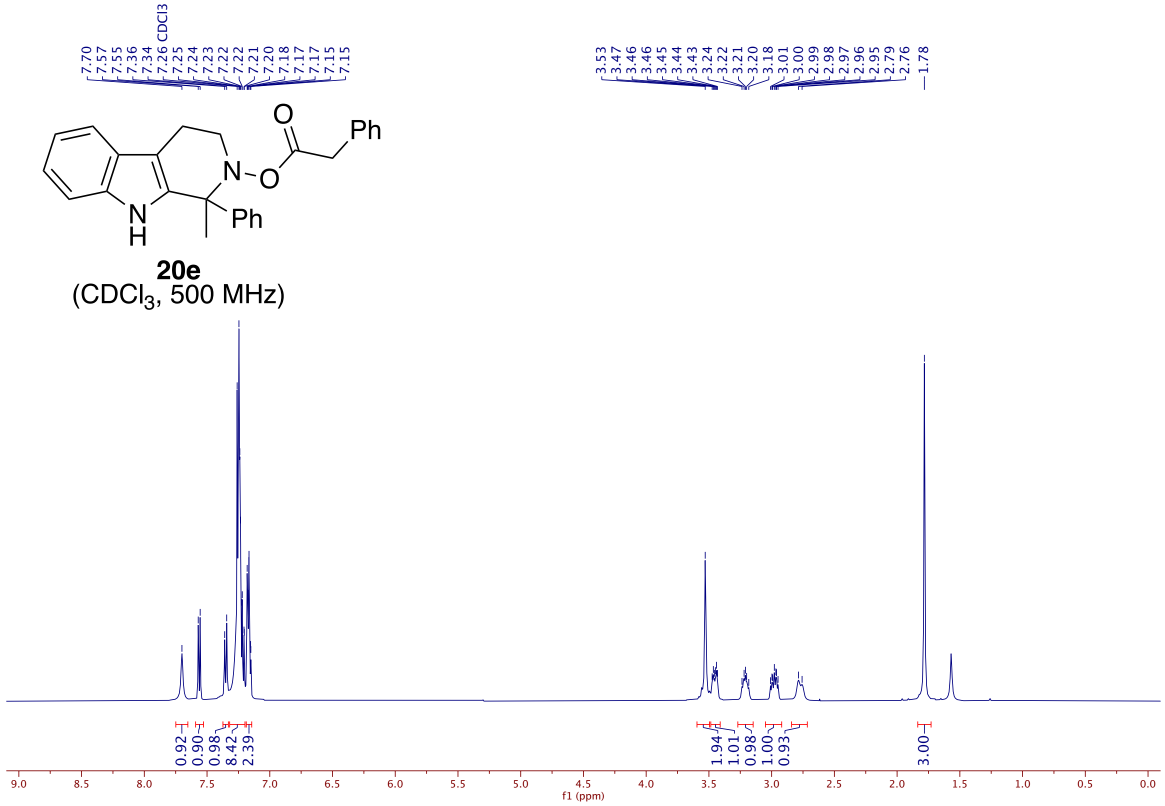
7.70 7.57 7.55 7.36 7.34 7.26 CDCl3 7.25 7.24 7.23 7.22 7.22 7.21 7.20 7.18 7.17 7.17 7.15
3.53 3.47 3.46 3.45 3.44 3.43 3.24 3.22 3.21 3.20 3.18 3.01 3.00 2.99 2.98 2.97 2.96 2.95 2.79 2.76
1.78
Ph
N
O
N
H
Ph
20e
(CDCl3, 500 MHz)
0.92 0.90 0.98 8.42 2.39
1.94 1.01 0.98 1.00 0.93
3.00
f1 (ppm)

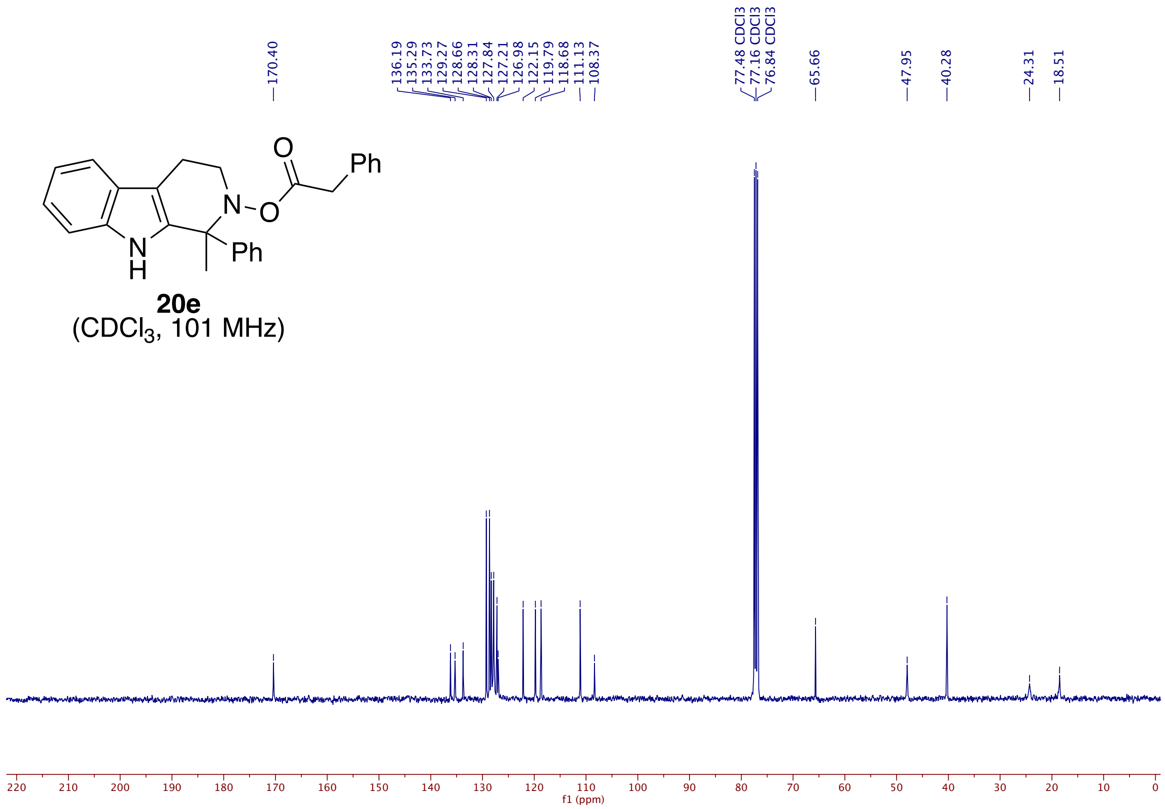
170.40
136.19 135.29 133.73 129.27 128.66 128.31 127.84 127.21 126.98 122.15 119.79 118.68 111.13 108.37
77.48 CDCl3 77.16 CDCl3 76.84 CDCl3
65.66
47.95
40.28
24.31
18.51
Ph
N
O
N
H
Ph
20e
(CDCl3, 101 MHz)
f1 (ppm)

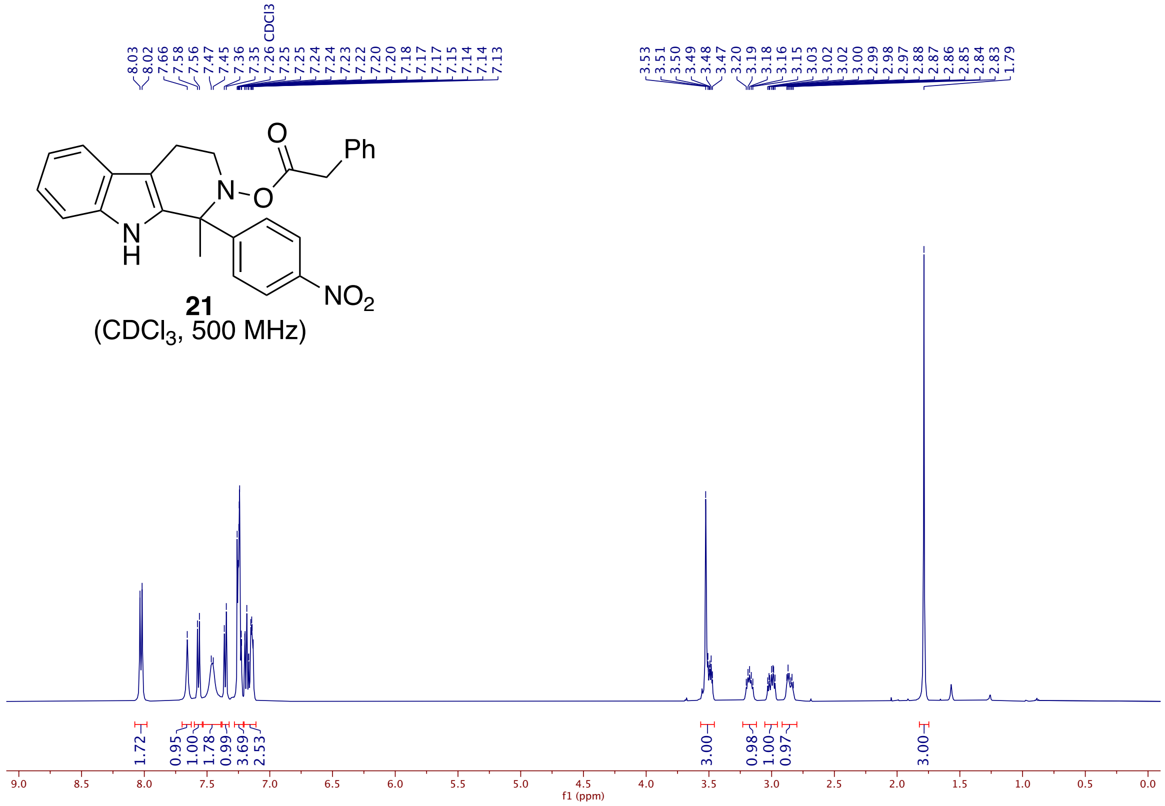
8.03 8.02 7.66 7.58 7.56 7.47 7.45 7.36 7.35 7.26 CDCl3 7.25 7.25 7.24 7.24 7.23 7.22 7.20 7.20 7.18 7.17 7.17 7.15 7.14 7.14 7.13
3.53 3.51 3.50 3.49 3.48 3.47 3.20 3.19 3.18 3.16 3.15 3.03 3.02 3.02 3.00 2.99 2.98 2.97 2.88 2.87 2.86 2.85 2.84 2.83 1.79
O
Ph
N
O
N
H
NO2
21
(CDCl3, 500 MHz)
1.72 0.95 1.00 1.78 0.99 3.69 2.53 3.00 0.98 1.00 0.97 3.00
f1 (ppm)

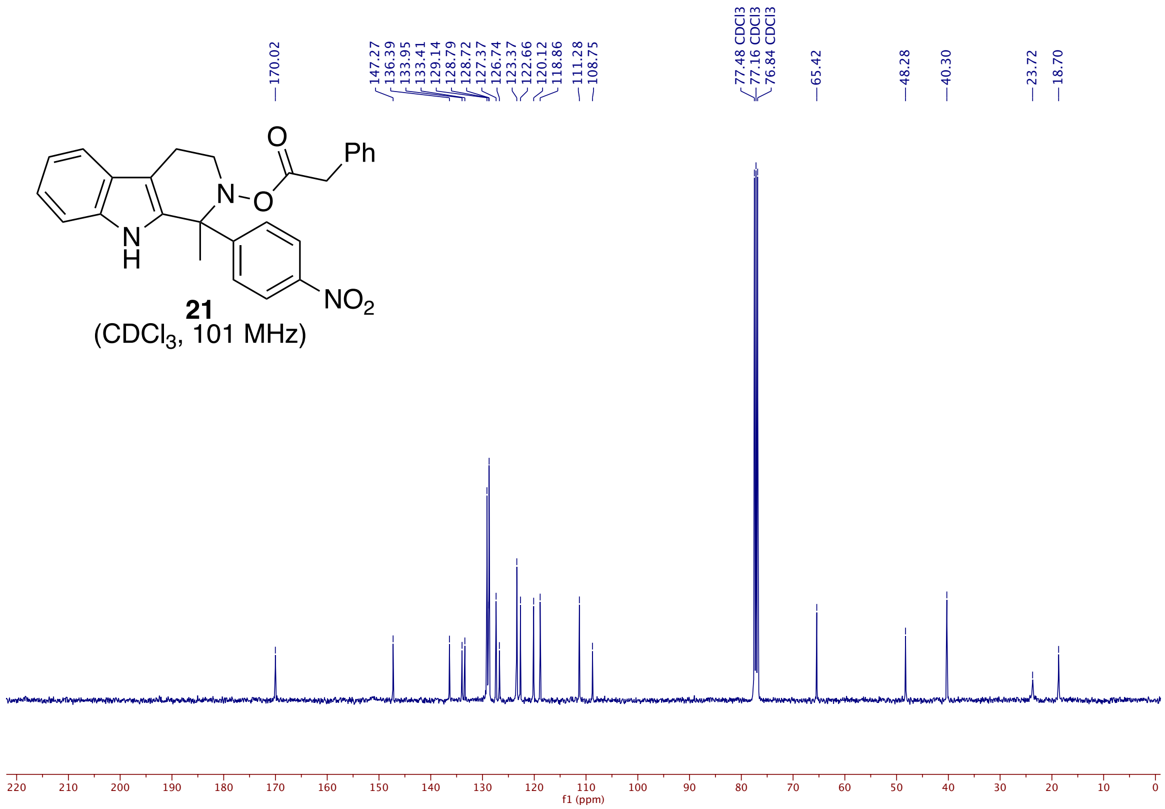
170.02
147.27 136.39 133.95 133.41 129.14 128.79 128.72 127.37 126.74 123.37 122.66 120.12 118.86 111.28 108.75
77.48 CDCl3 77.16 CDCl3 76.84 CDCl3
65.42
48.28
40.30
23.72
18.70
O
Ph
N
O
N
H
NO2
21
(CDCl3, 101 MHz)
f1 (ppm)

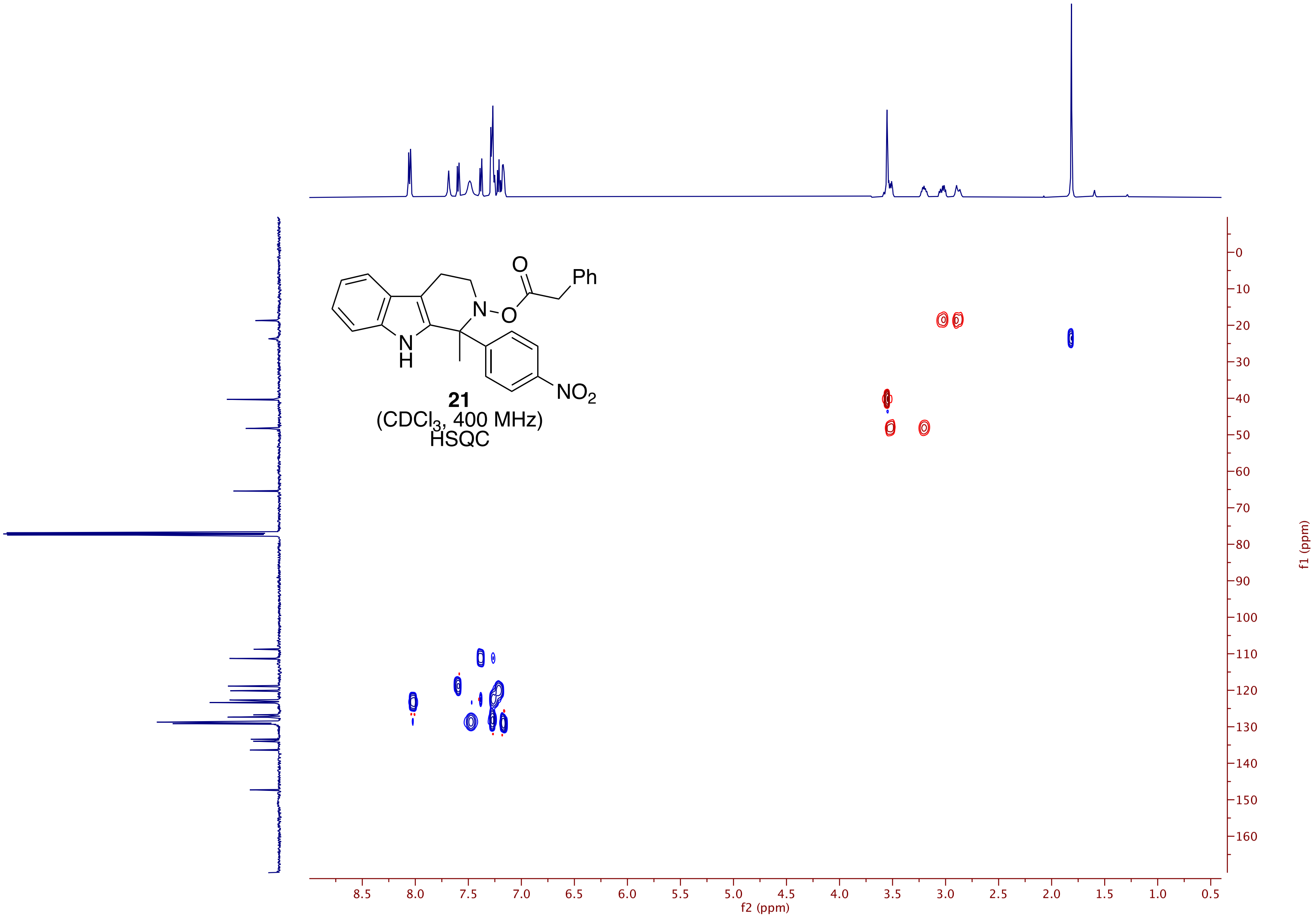
Ph
O
N
O
N
H
NO2
21
(CDCl3, 400 MHz)
HSQC
f1 (ppm)
f2 (ppm)

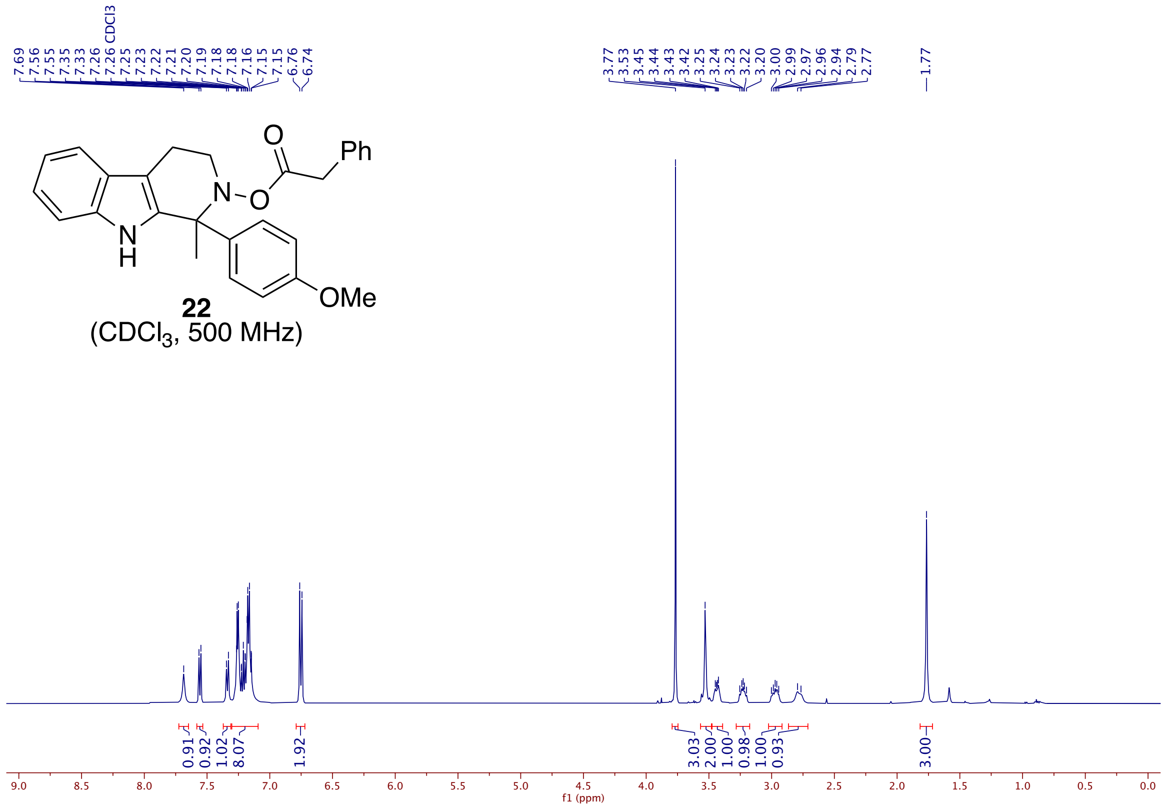

22

(CDCl$_3$, 500 MHz)

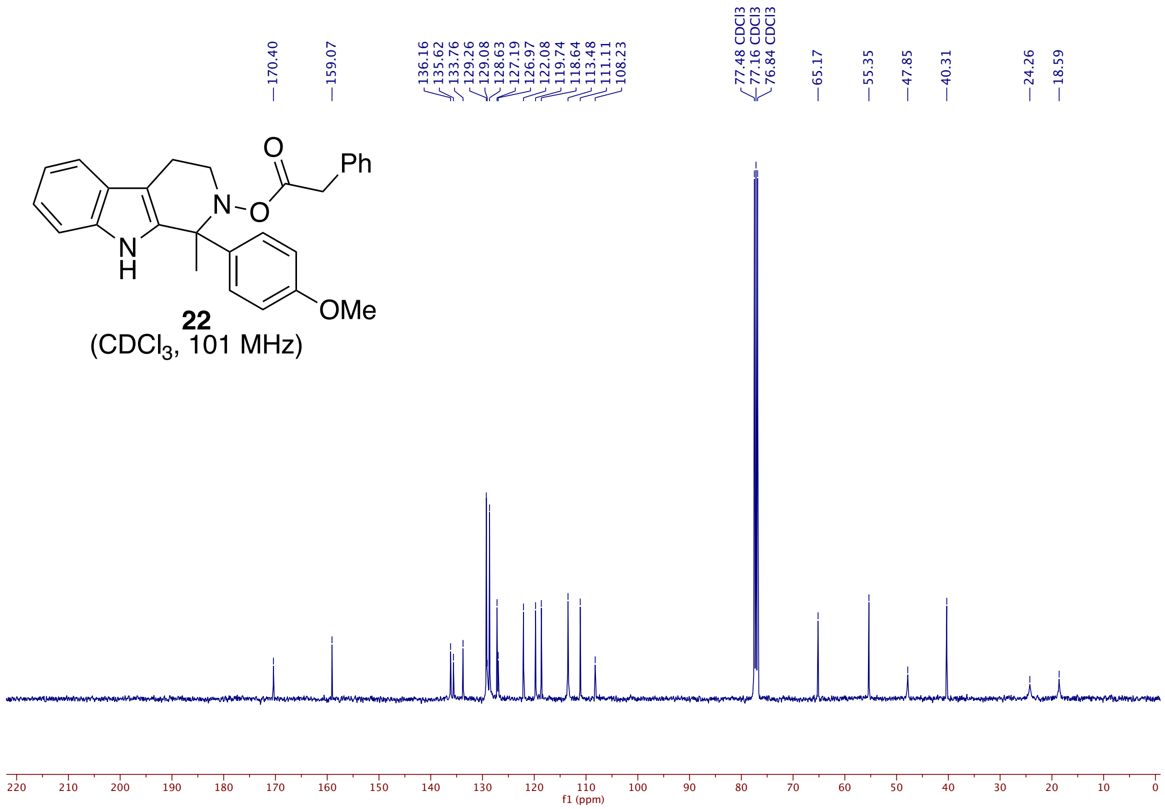

22

(CDCl$_3$, 101 MHz)

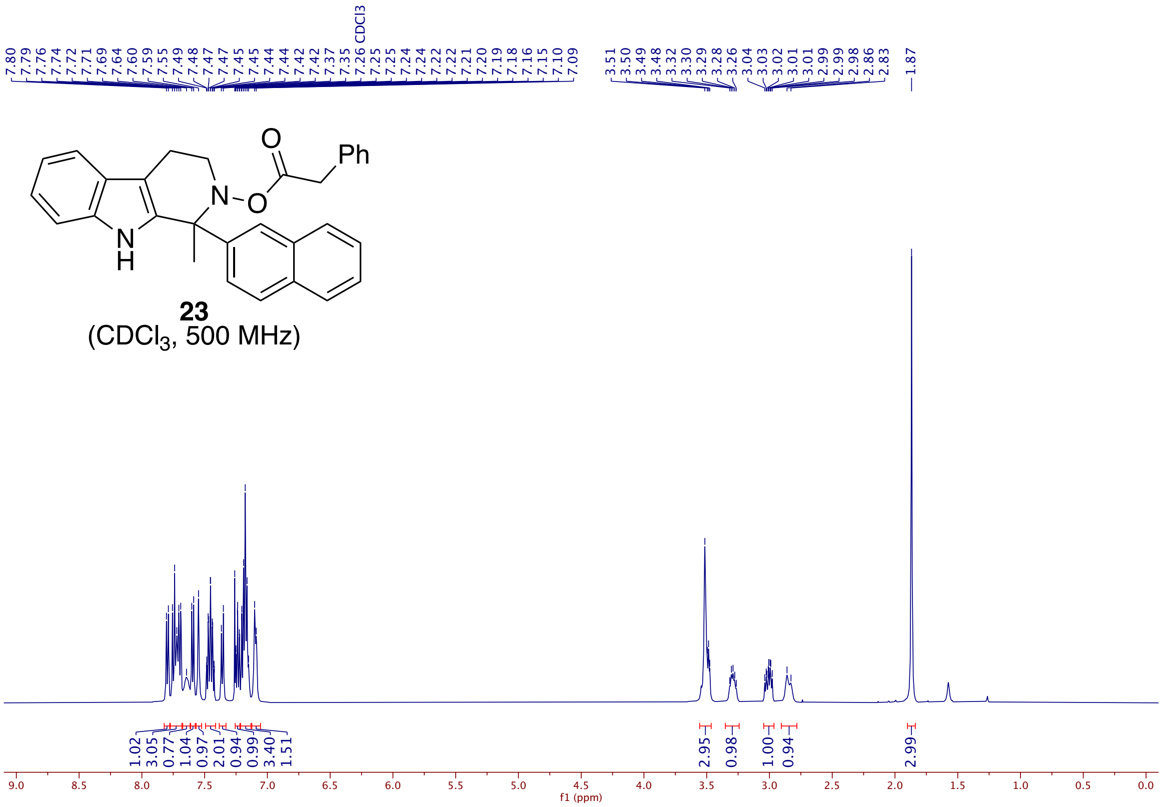

23
(CDCl$_3$, 500 MHz)

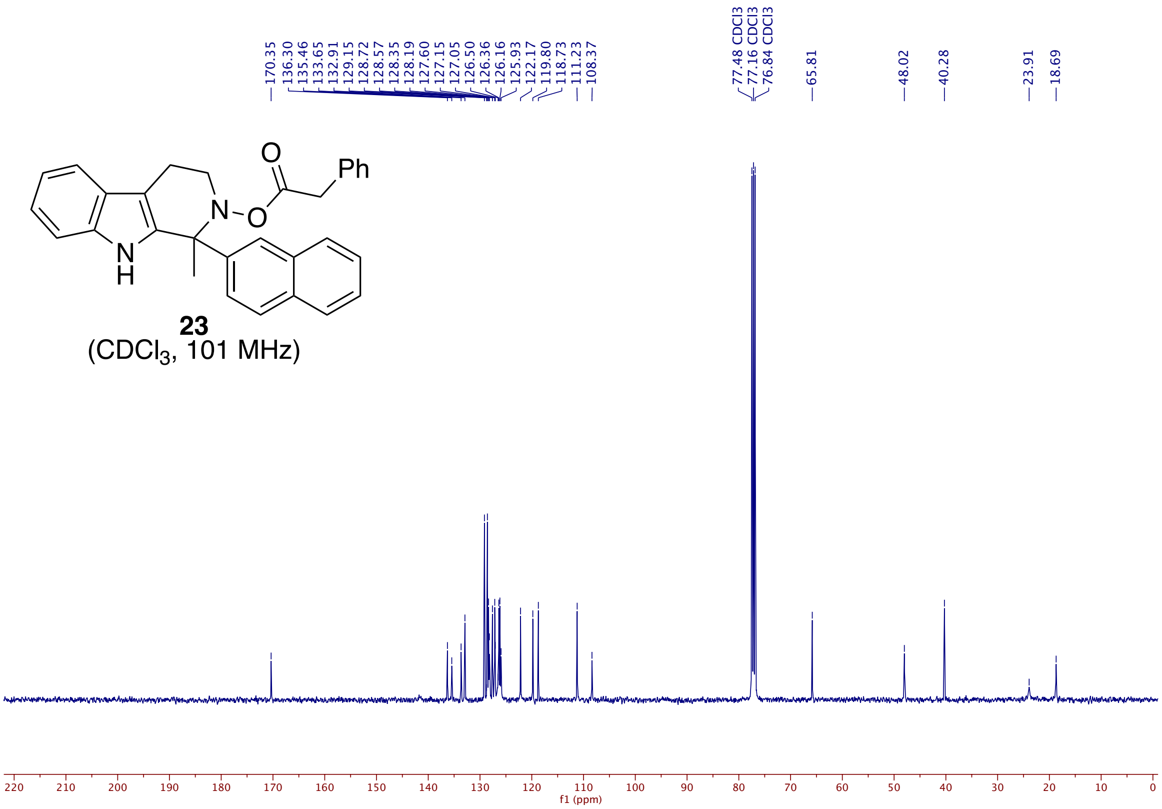

23
(CDCl$_3$, 101 MHz)

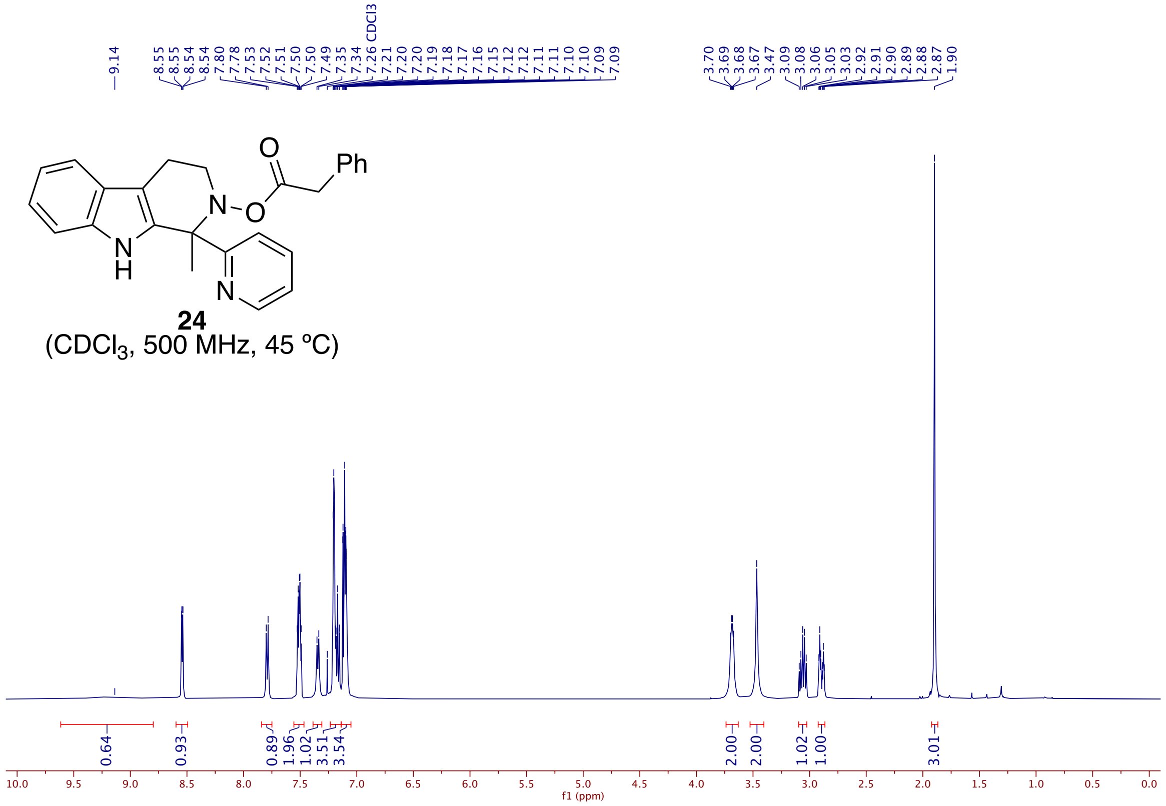
9.14
8.55 8.55 8.54 8.54 7.80 7.78 7.53 7.52 7.51 7.50 7.50 7.49 7.35 7.34 7.26 CDCl3 7.21 7.20 7.20 7.19 7.18 7.17 7.16 7.15 7.12 7.12 7.11 7.11 7.10 7.10 7.09 7.09
3.70 3.69 3.68 3.67 3.47 3.09 3.08 3.06 3.05 3.03 2.92 2.91 2.90 2.89 2.88 2.87 1.90
O
Ph
N
O
N
H
N
24
(CDCl3, 500 MHz, 45 ºC)
0.64 0.93 0.89 1.96 1.02 3.51 3.54 2.00 2.00 1.02 1.00 3.01
10.0 9.5 9.0 8.5 8.0 7.5 7.0 6.5 6.0 5.5 5.0 4.5 4.0 3.5 3.0 2.5 2.0 1.5 1.0 0.5 0.0
f1 (ppm)

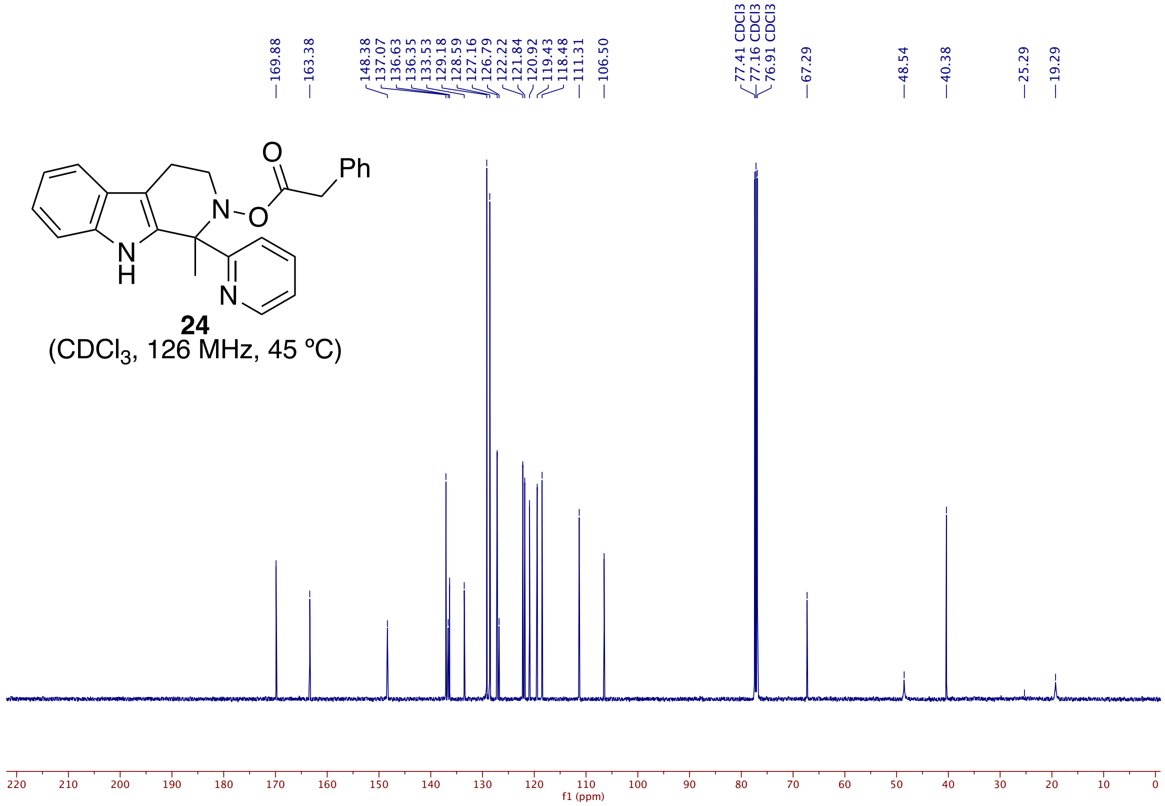
169.88 163.38 148.38 137.07 136.63 136.35 133.53 129.18 128.59 127.16 126.79 122.22 121.84 120.92 119.43 118.48 111.31 106.50
77.41 CDCl3 77.16 CDCl3 76.91 CDCl3 67.29 48.54 40.38 25.29 19.29
O
Ph
N
O
N
H
N
24
(CDCl3, 126 MHz, 45 ºC)
220 210 200 190 180 170 160 150 140 130 120 110 100 90 80 70 60 50 40 30 20 10 0
f1 (ppm)

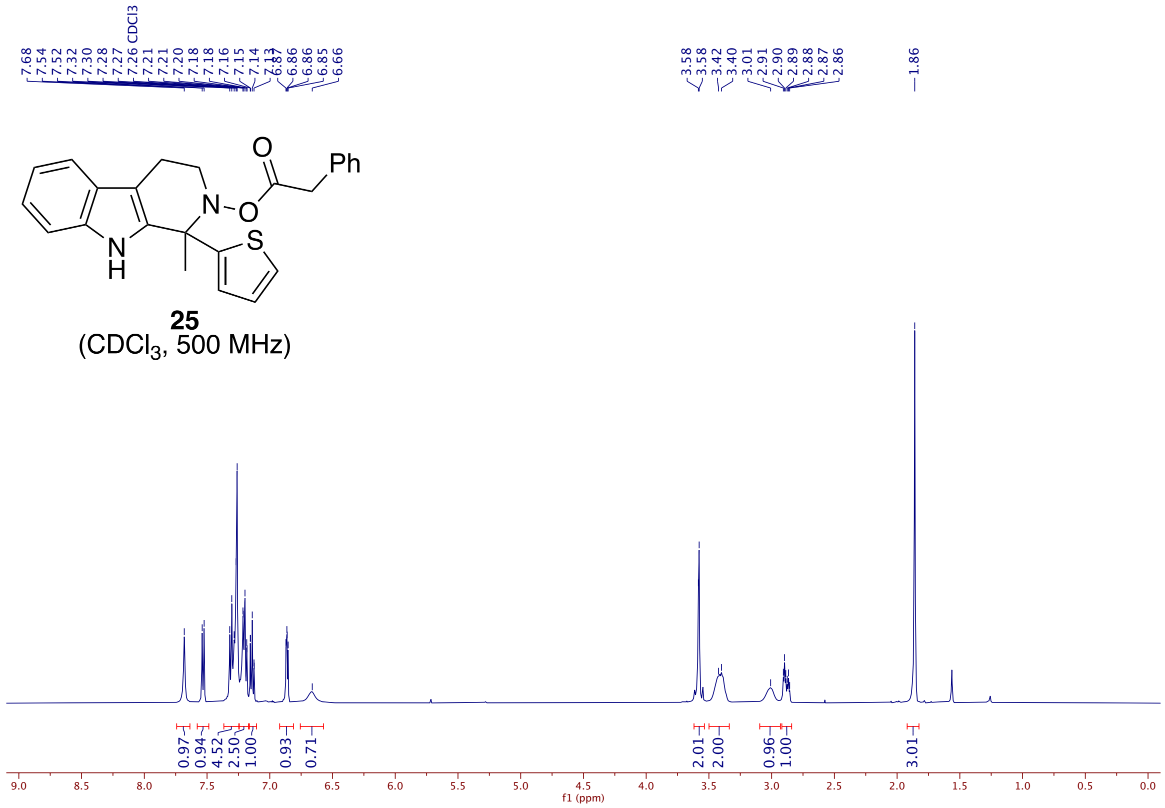
25
(CDCl3, 500 MHz)
f1 (ppm)

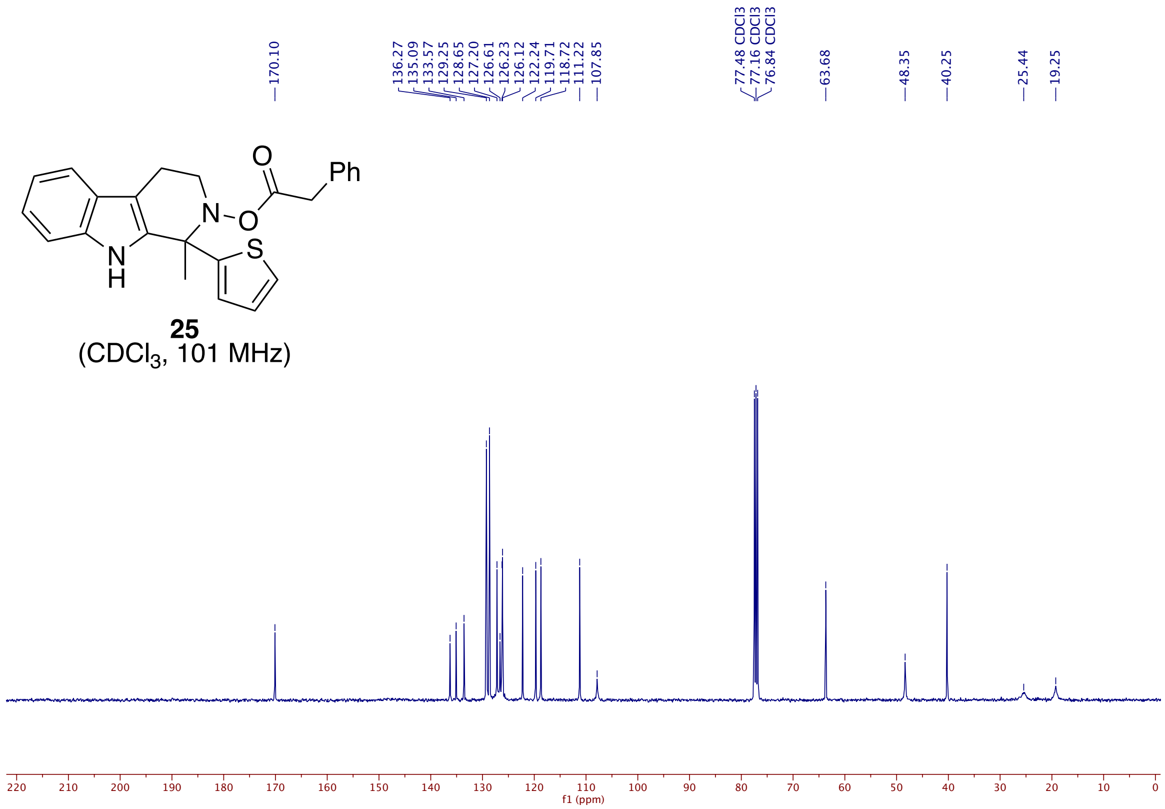
25
(CDCl3, 101 MHz)
f1 (ppm)

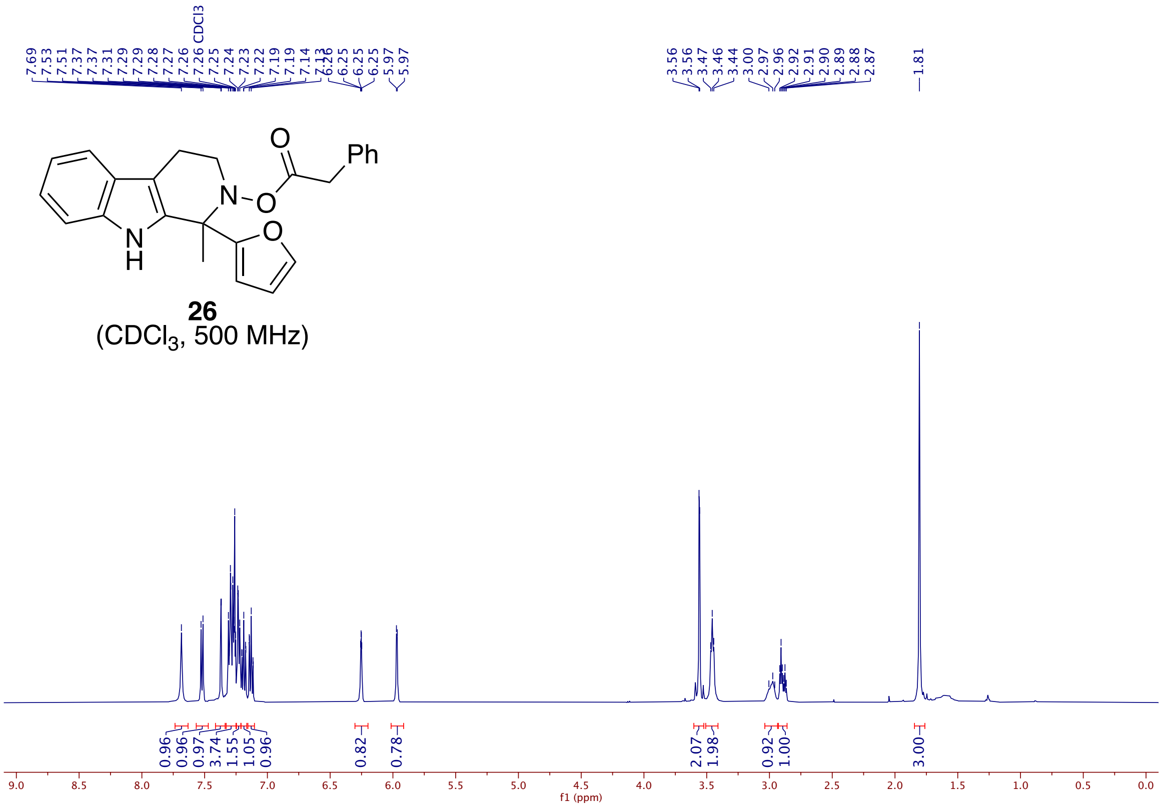

26
($CDCl_3$, 500 MHz)

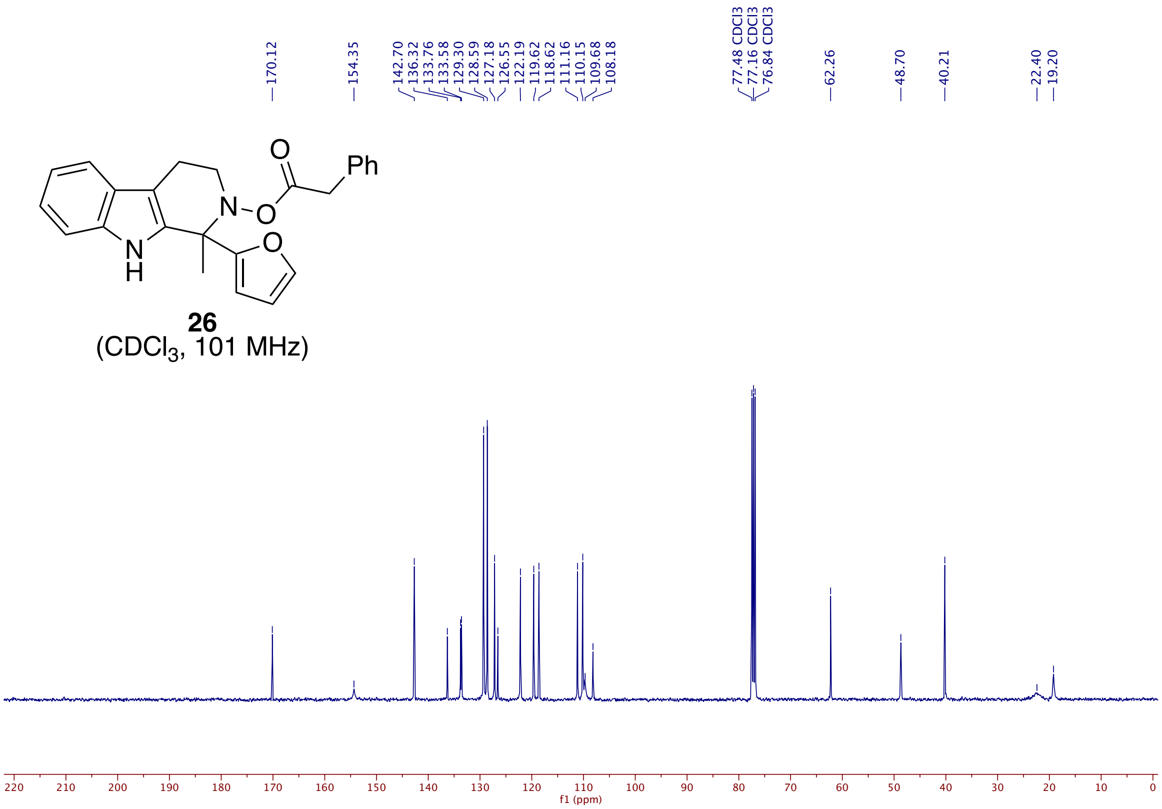

26
($CDCl_3$, 101 MHz)

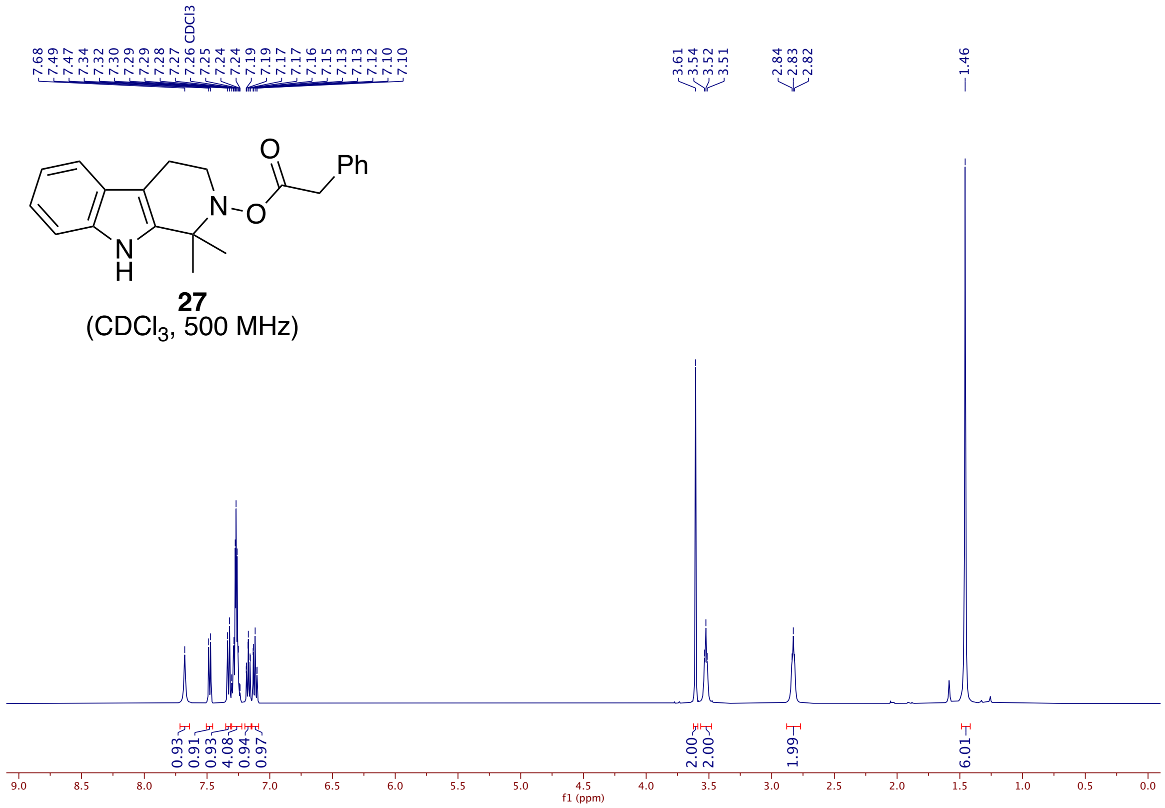
Ph
O
N
O
N
H
27
(CDCl3, 500 MHz)
7.68 7.49 7.47 7.34 7.32 7.30 7.29 7.29 7.28 7.27 7.26 CDCl3 7.25 7.24 7.24 7.19 7.19 7.17 7.17 7.16 7.15 7.13 7.13 7.12 7.10 7.10
3.61 3.54 3.52 3.51
2.84 2.83 2.82
1.46
0.93 0.91 0.93 4.08 0.94 0.97
2.00 2.00
1.99
6.01
f1 (ppm)

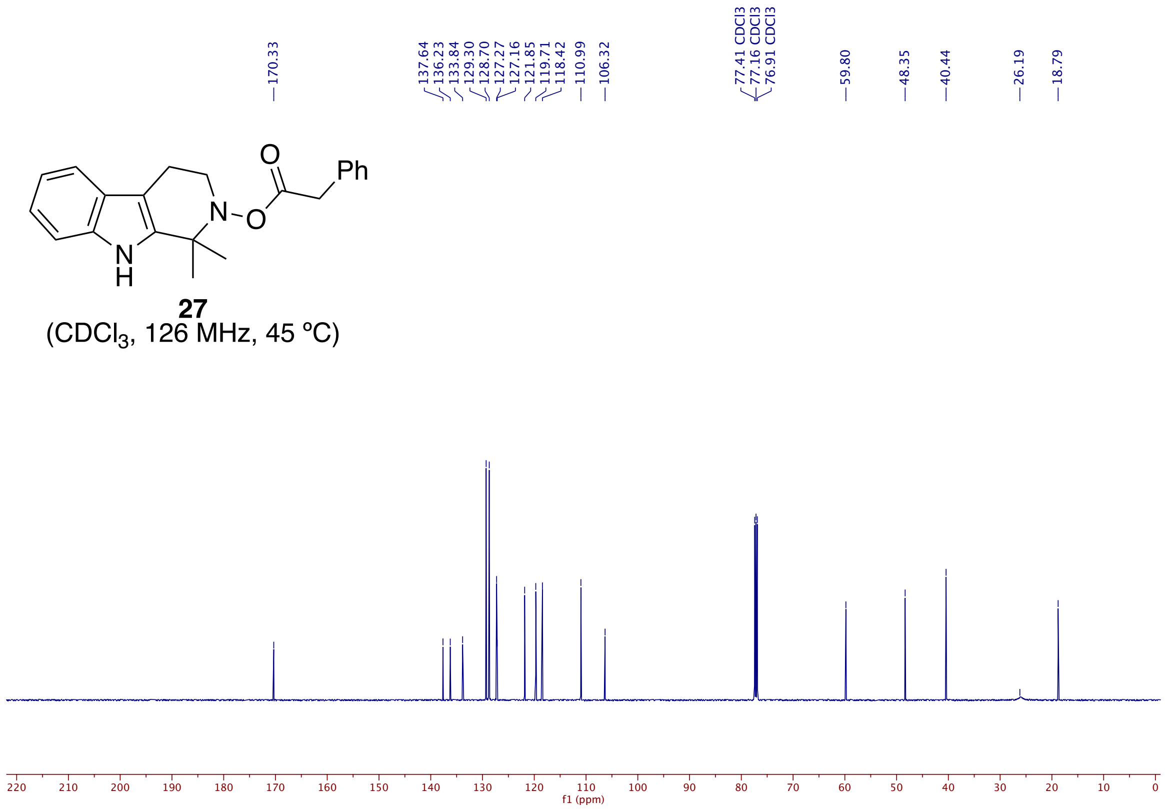
Ph
O
N
O
N
H
27
(CDCl3, 126 MHz, 45 ºC)
170.33
137.64 136.23 133.84 129.30 128.70 127.27 127.16 121.85 119.71 118.42 110.99 106.32
77.41 CDCl3 77.16 CDCl3 76.91 CDCl3
59.80
48.35
40.44
26.19
18.79
f1 (ppm)

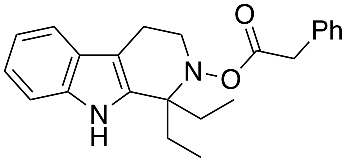

28
($CDCl_3$, 500 MHz)

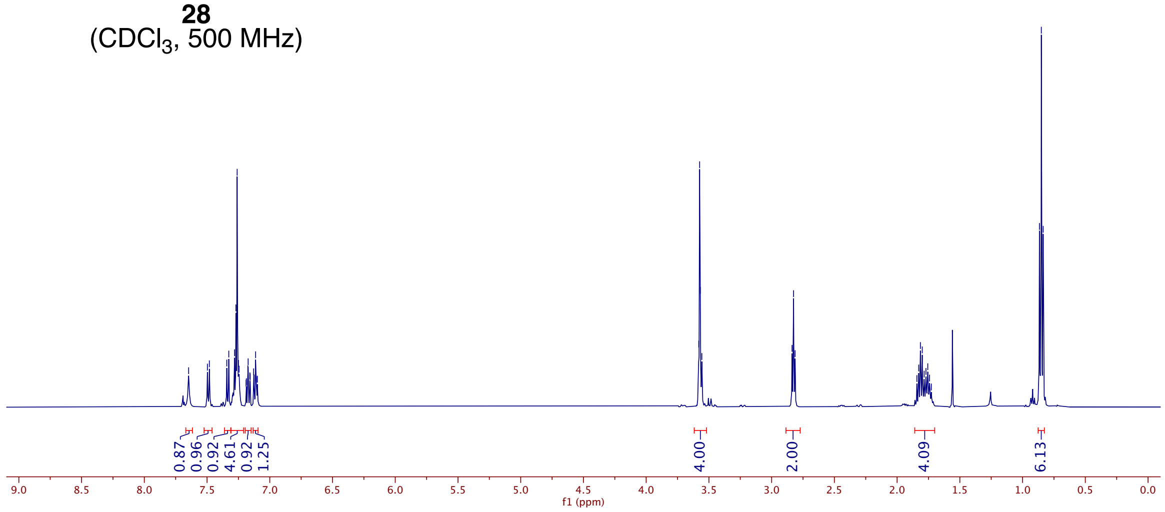

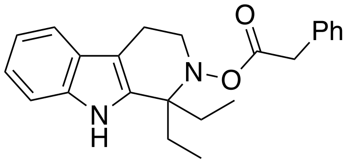

28
($CDCl_3$, 101 MHz)

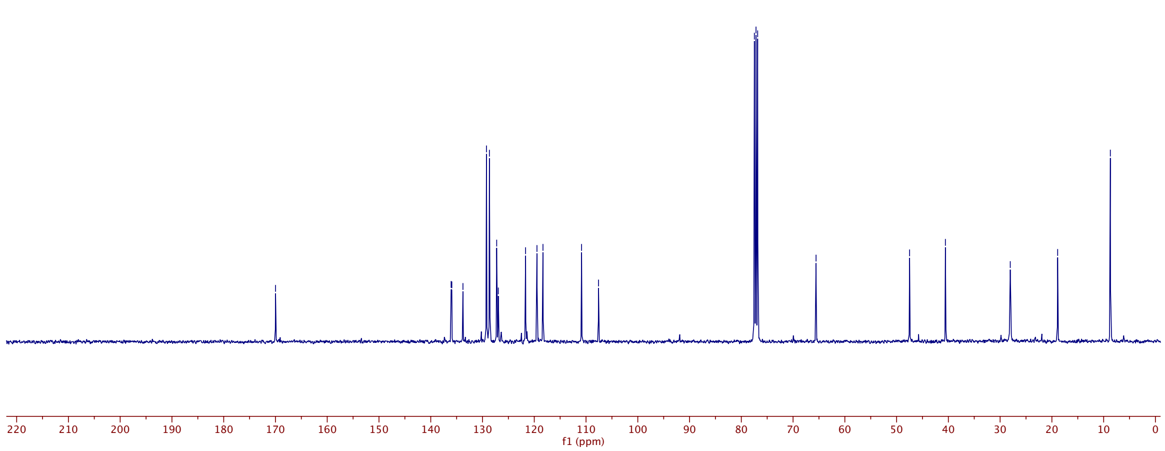

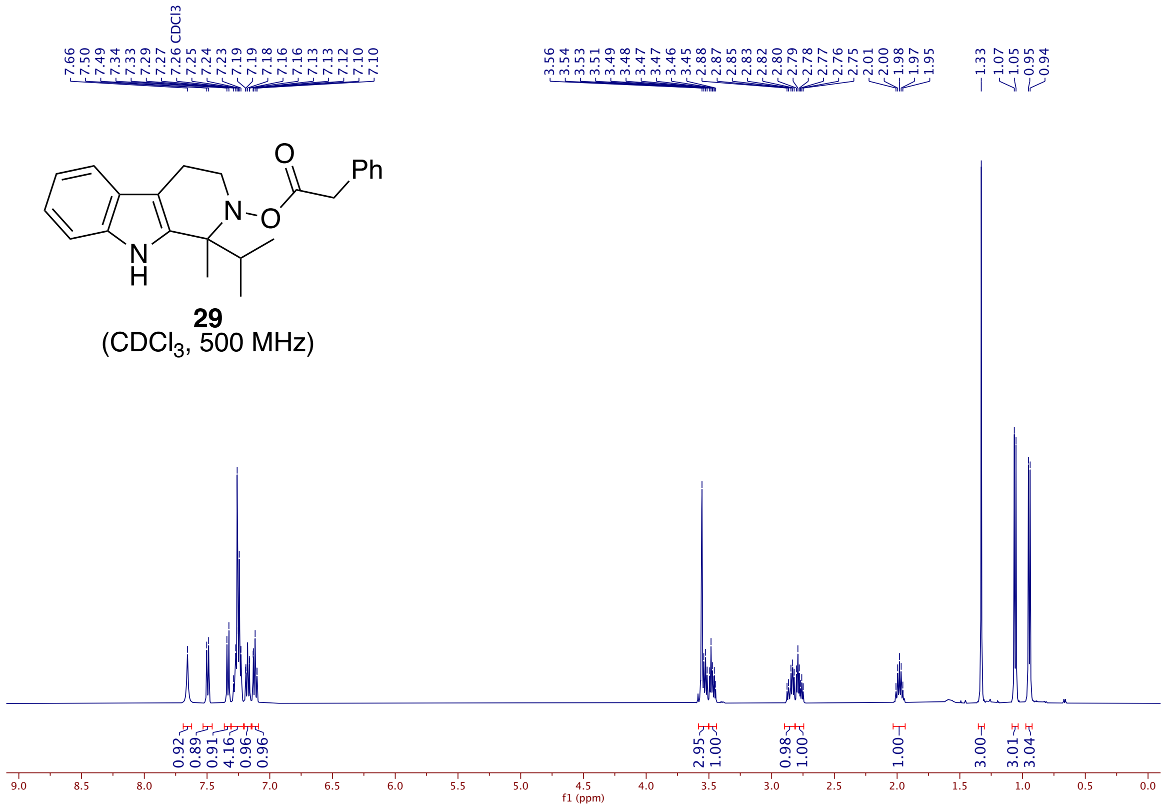

29
($CDCl_3$, 500 MHz)

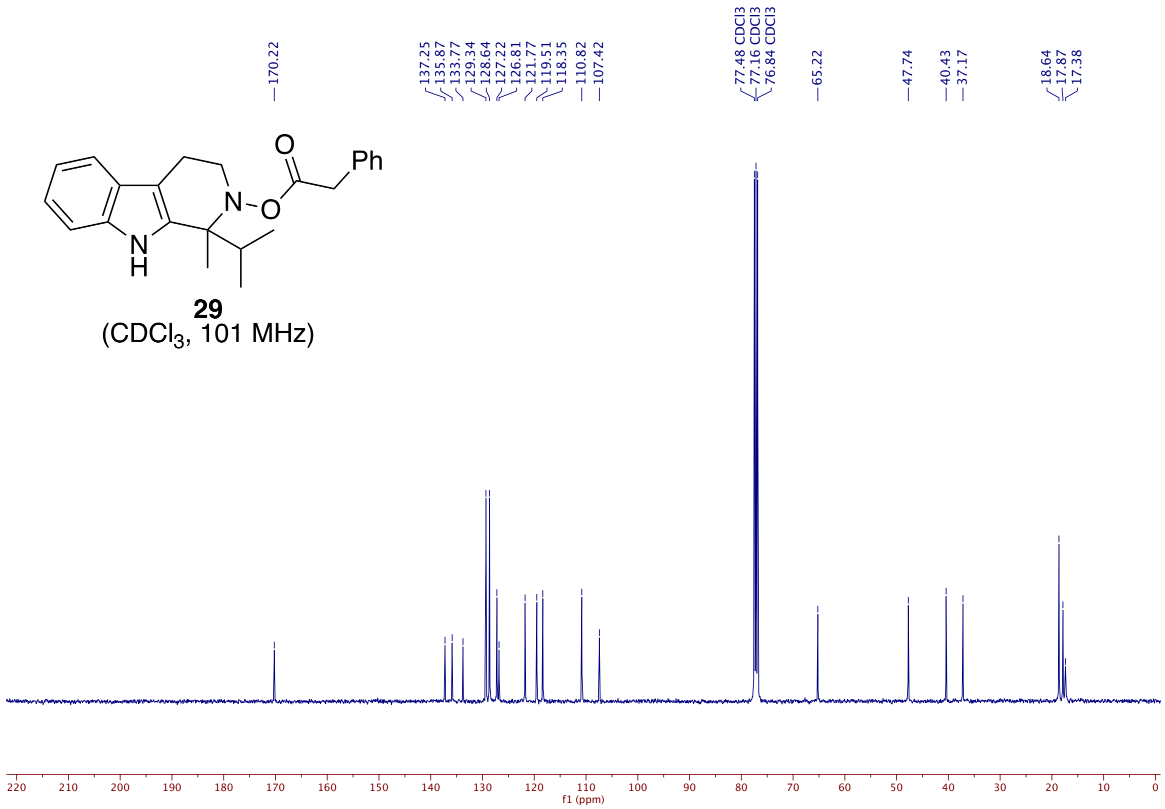

29
($CDCl_3$, 101 MHz)

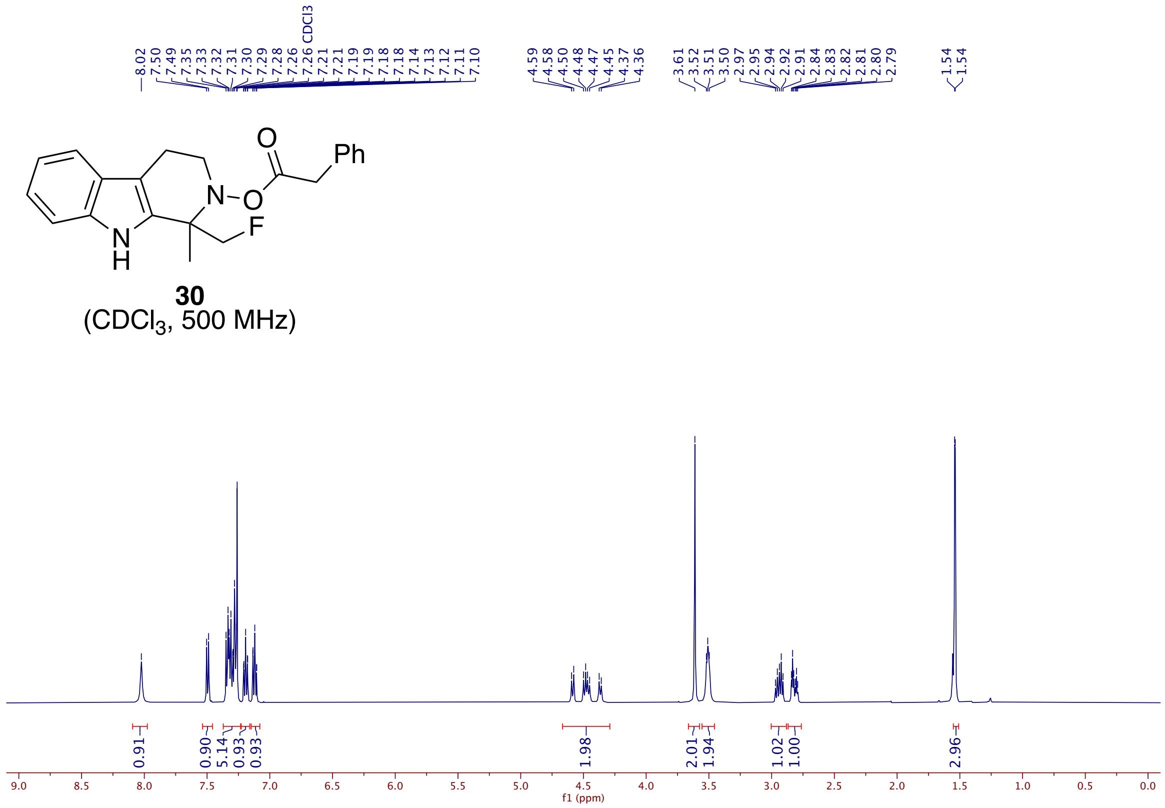

30
($CDCl_3$, 500 MHz)

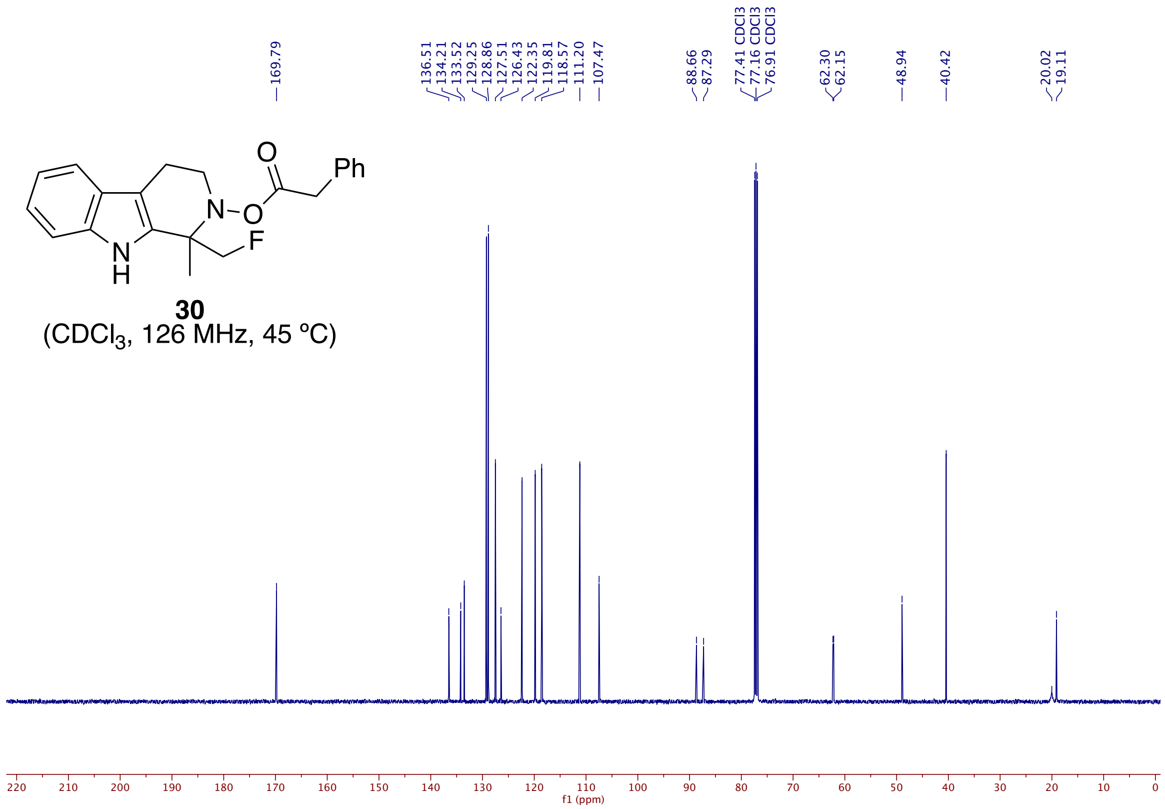

30
($CDCl_3$, 126 MHz, 45 ºC)

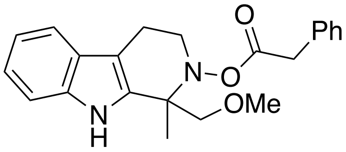

31
($CDCl_3$, 500 MHz)

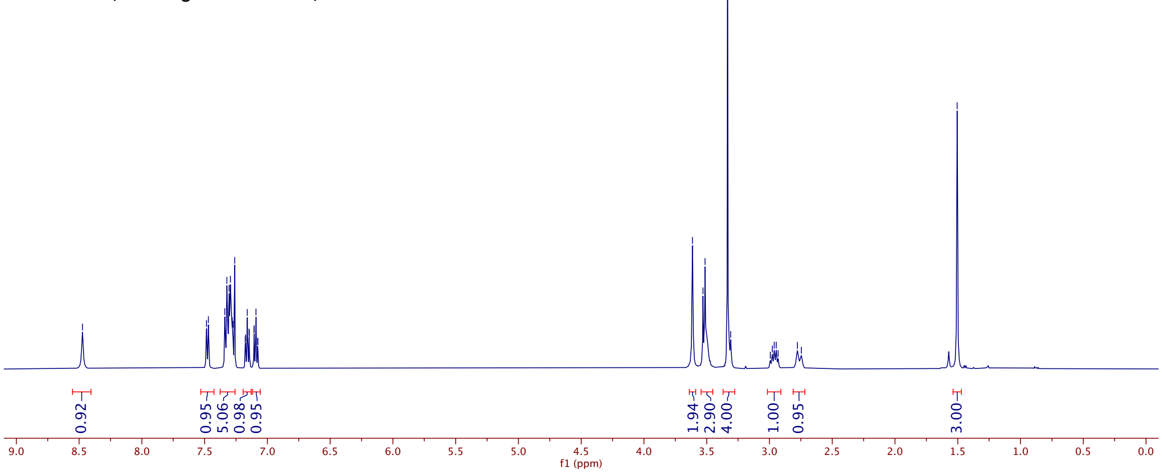

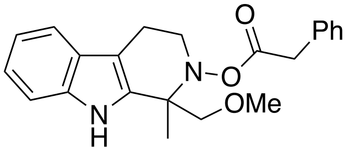

31
($CDCl_3$, 126 MHz, 45 °C)

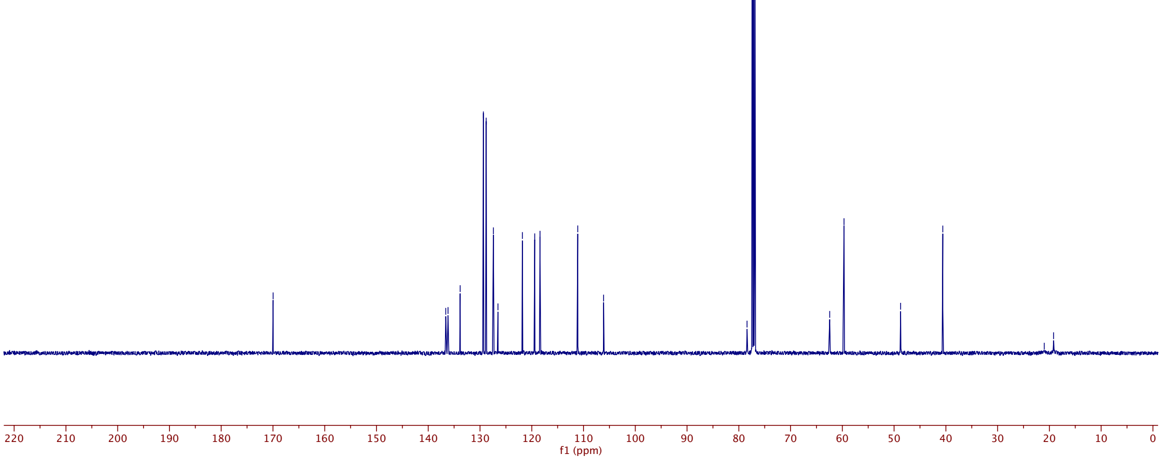

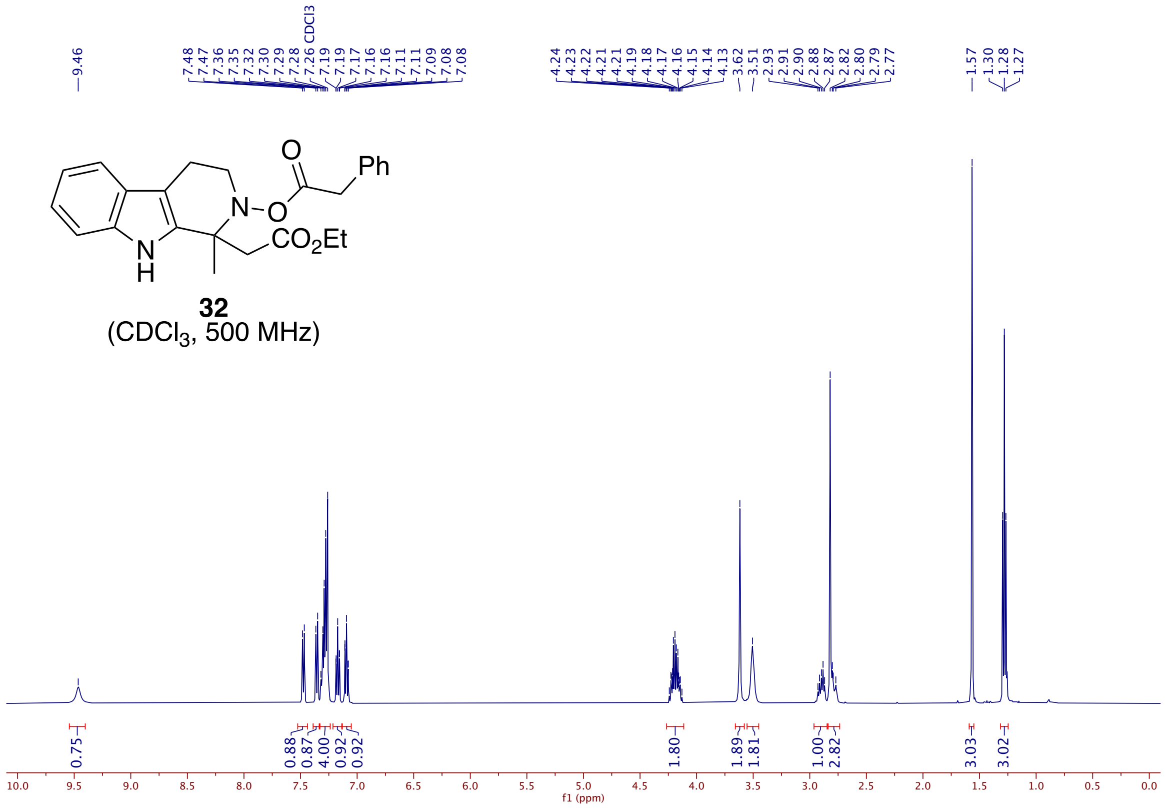
9.46
7.48 7.47 7.36 7.35 7.32 7.30 7.29 7.28 7.26 CDCl3 7.19 7.19 7.17 7.16 7.11 7.11 7.09 7.08 7.08
4.24 4.23 4.22 4.21 4.21 4.19 4.18 4.17 4.16 4.15 4.14 4.13 3.62 3.51 2.93 2.91 2.90 2.88 2.87 2.82 2.80 2.79 2.77
1.57 1.30 1.28 1.27
O
Ph
N
O
N
H
CO2Et
32
(CDCl3, 500 MHz)
0.75
0.88 0.87 4.00 0.92 0.92
1.80
1.89 1.81
1.00 2.82
3.03
3.02
10.0 9.5 9.0 8.5 8.0 7.5 7.0 6.5 6.0 5.5 5.0 4.5 4.0 3.5 3.0 2.5 2.0 1.5 1.0 0.5 0.0
f1 (ppm)

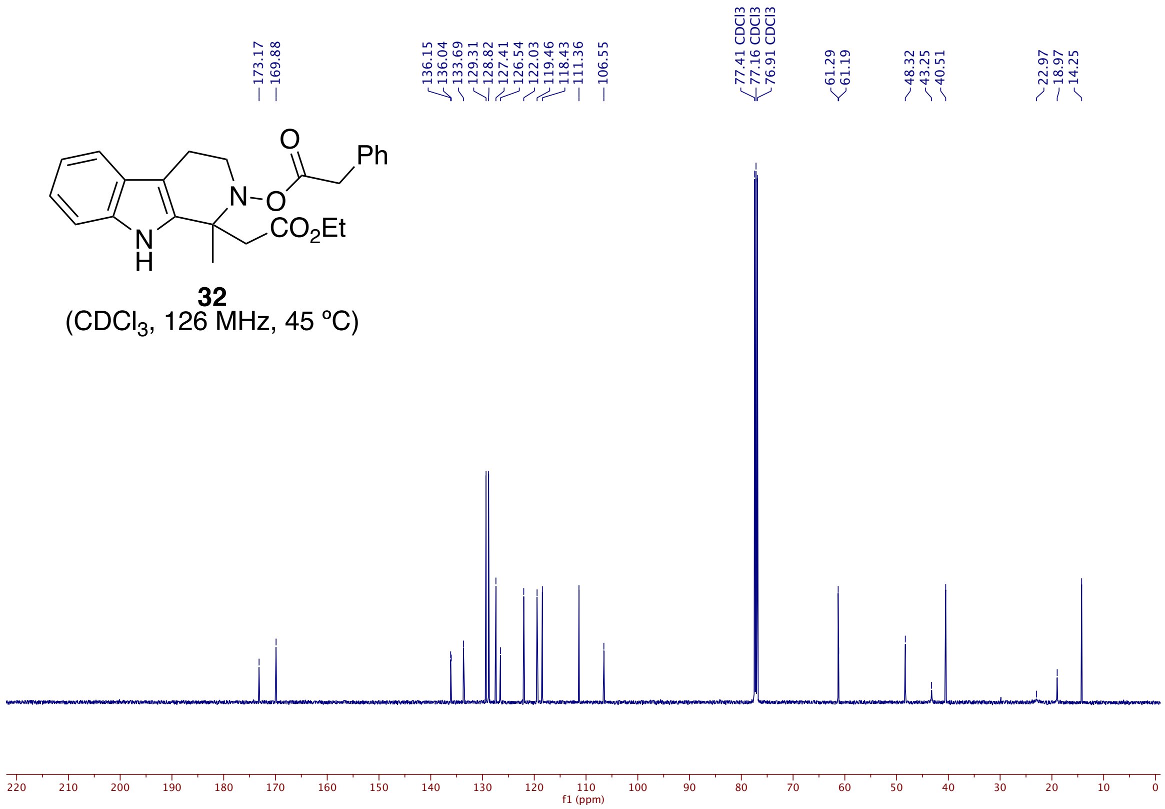
173.17
169.88
136.15 136.04 133.69 129.31 128.82 127.41 126.54 122.03 119.46 118.43 111.36
106.55
77.41 CDCl3 77.16 CDCl3 76.91 CDCl3
61.29 61.19
48.32 43.25 40.51
22.97 18.97 14.25
O
Ph
N
O
N
H
CO2Et
32
(CDCl3, 126 MHz, 45 °C)
220 210 200 190 180 170 160 150 140 130 120 110 100 90 80 70 60 50 40 30 20 10 0
f1 (ppm)

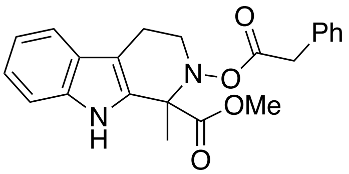

33
($CDCl_3$, 500 MHz)

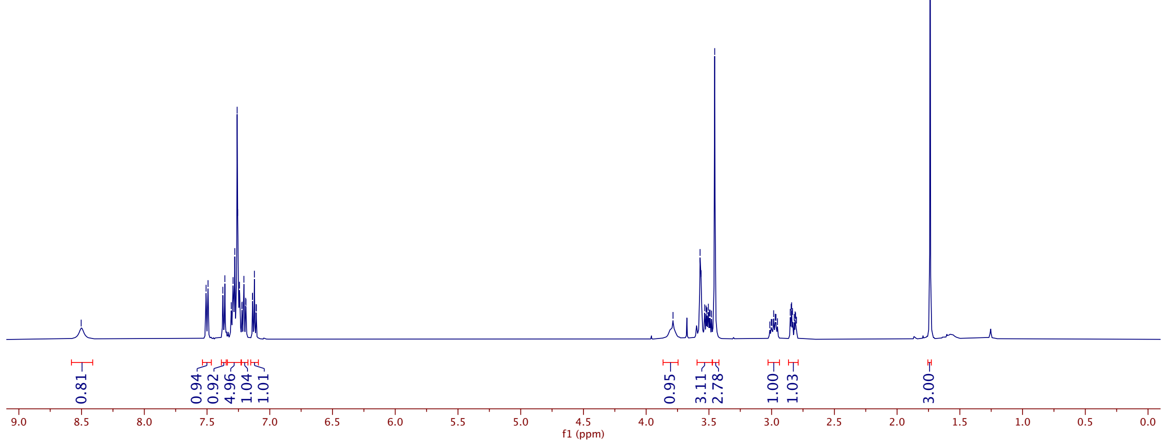

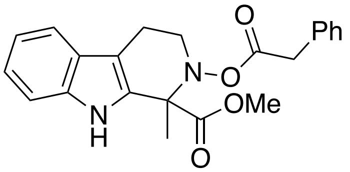

33
($CDCl_3$, 126 MHz, 45 ºC)

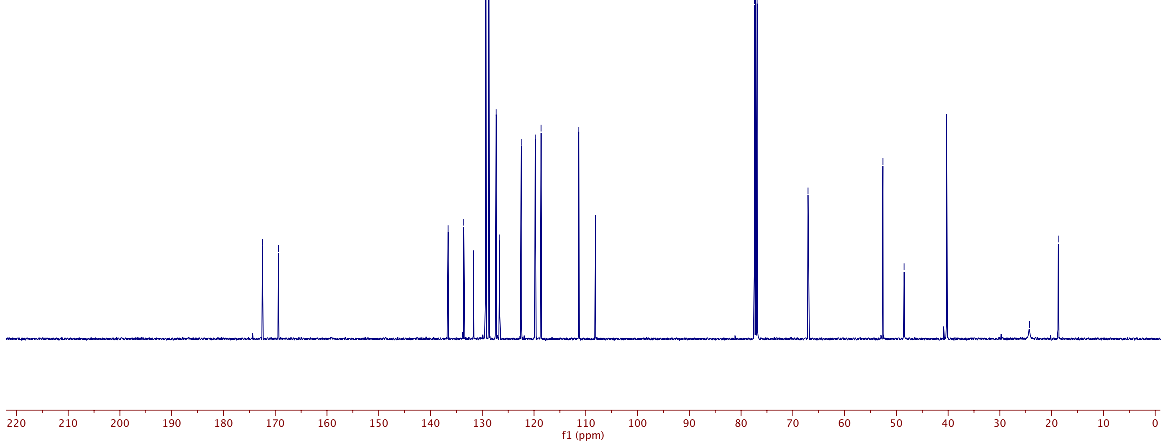

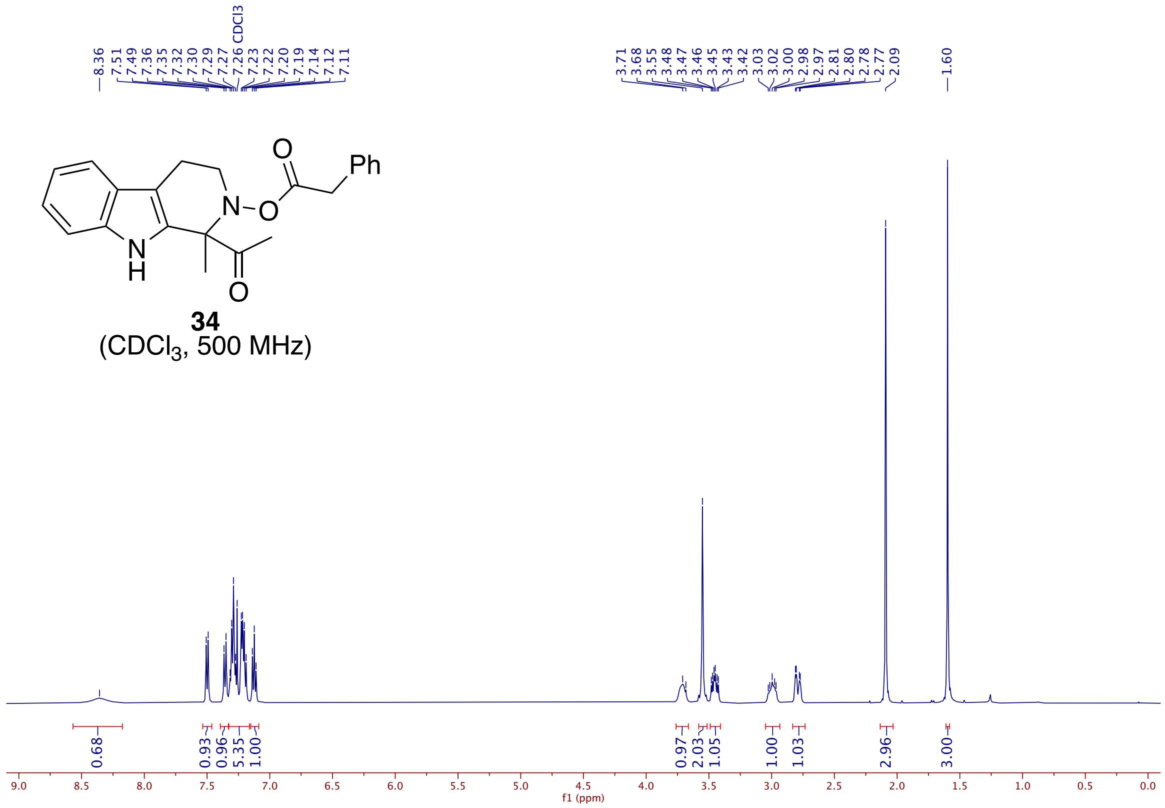

34
($CDCl_3$, 500 MHz)

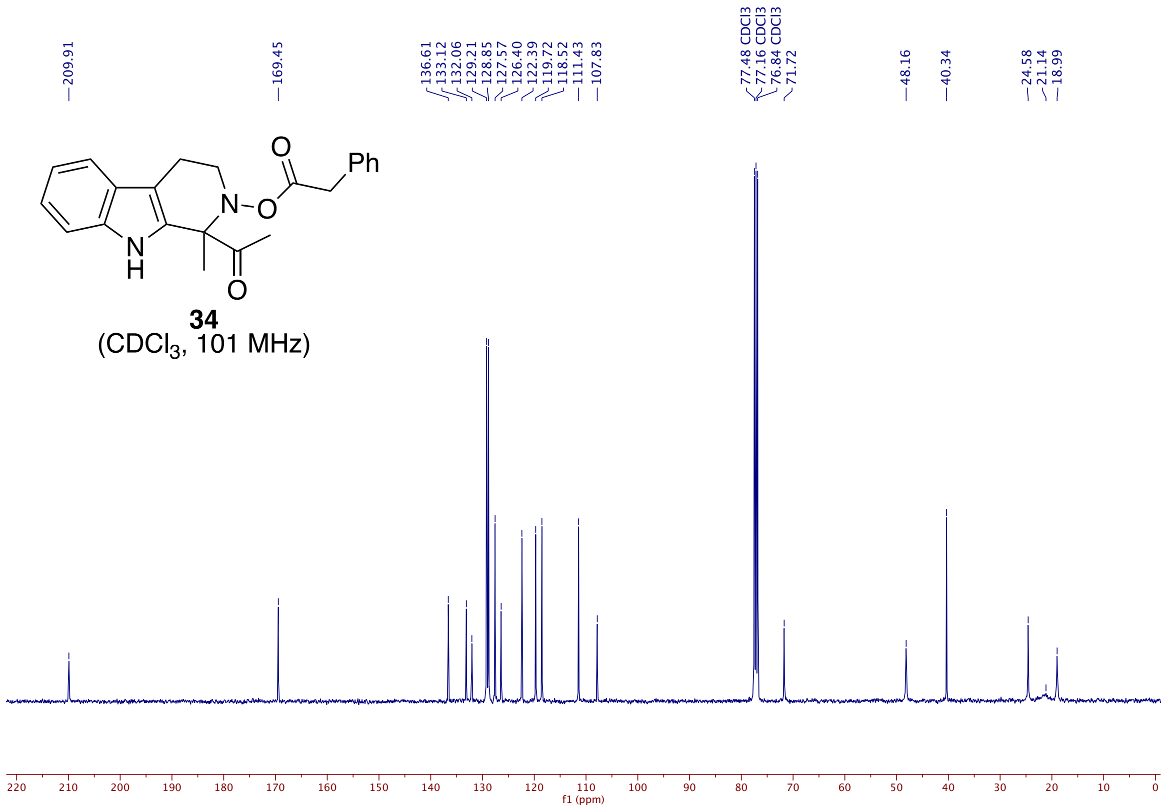

34
($CDCl_3$, 101 MHz)

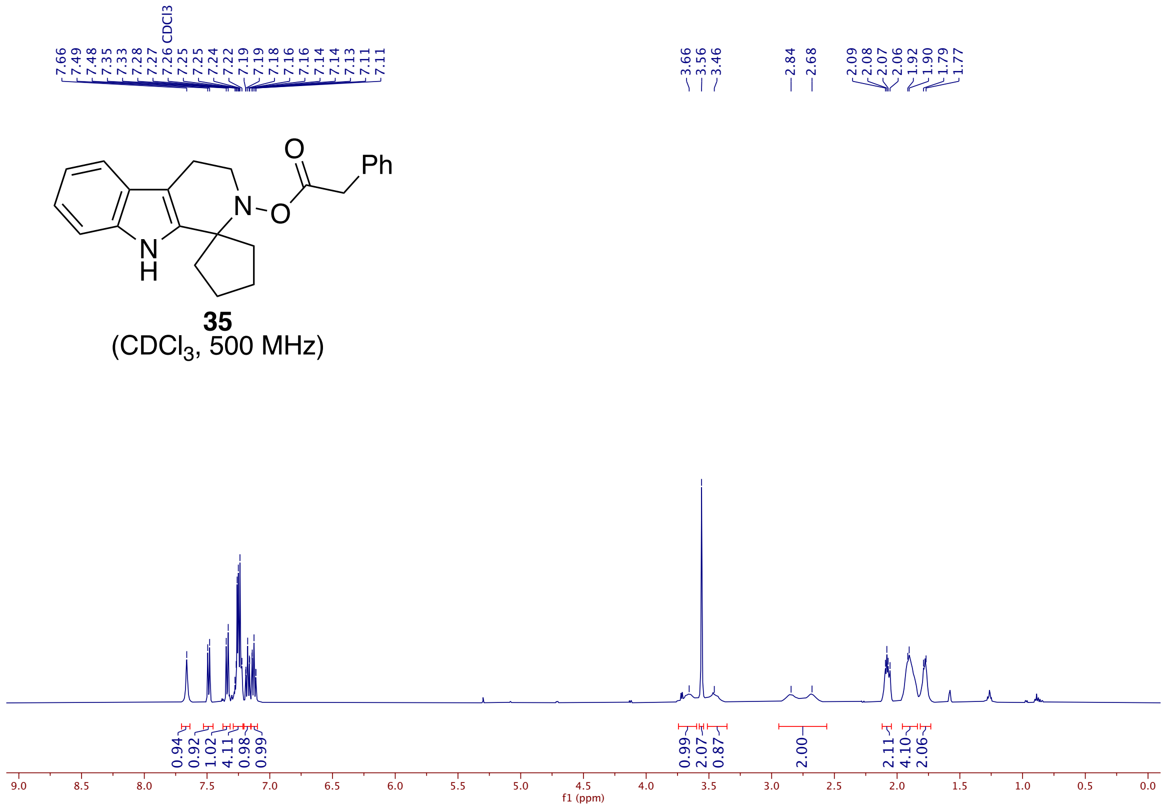
7.66 7.49 7.48 7.35 7.33 7.28 7.27 7.26 CDCl3 7.25 7.25 7.24 7.22 7.19 7.19 7.18 7.16 7.16 7.14 7.14 7.13 7.11 7.11
3.66 3.56 3.46
2.84 2.68
2.09 2.08 2.07 2.06 1.92 1.90 1.79 1.77
O
Ph
N
O
N
H
35
(CDCl3, 500 MHz)
0.94 0.92 1.02 4.11 0.98 0.99
0.99 2.07 0.87
2.00
2.11 4.10 2.06
9.0 8.5 8.0 7.5 7.0 6.5 6.0 5.5 5.0 4.5 4.0 3.5 3.0 2.5 2.0 1.5 1.0 0.5 0.0
f1 (ppm)

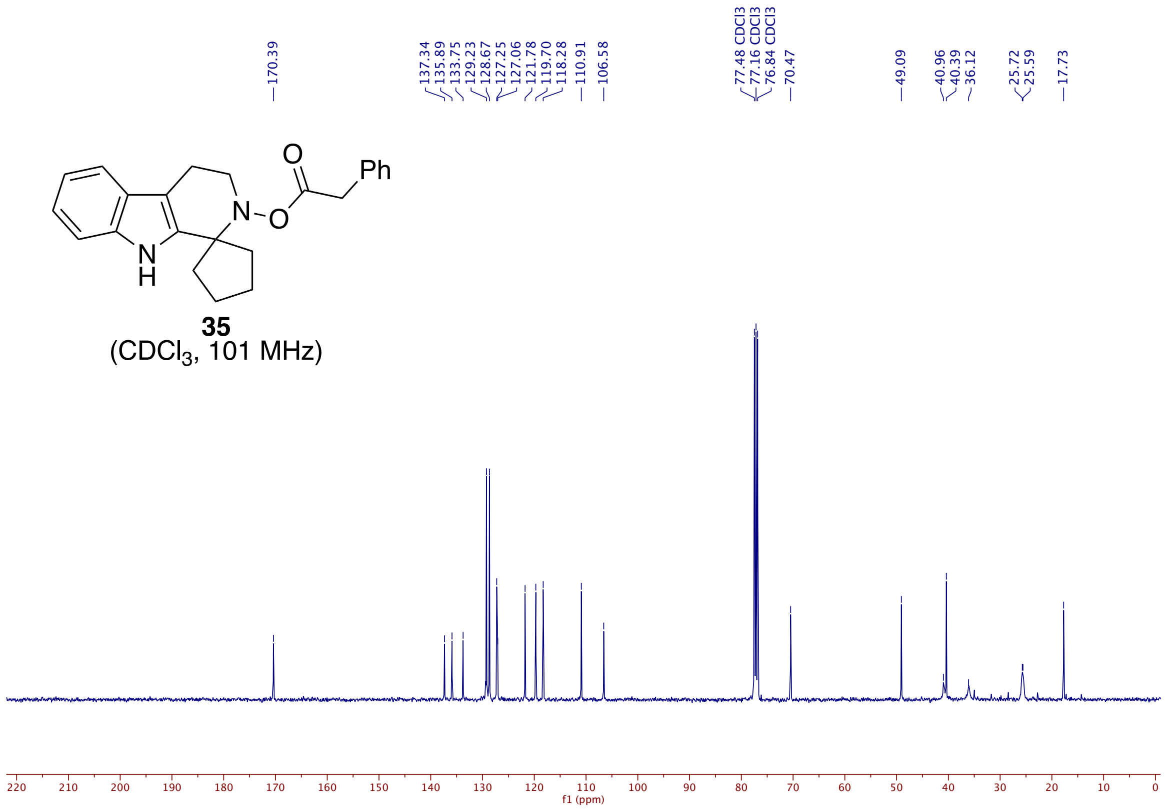
170.39
137.34 135.89 133.75 129.23 128.67 127.25 127.06 121.78 119.70 118.28 110.91 106.58
77.48 CDCl3 77.16 CDCl3 76.84 CDCl3 70.47
49.09
40.96 40.39 36.12
25.72 25.59
17.73
O
Ph
N
O
N
H
35
(CDCl3, 101 MHz)
220 210 200 190 180 170 160 150 140 130 120 110 100 90 80 70 60 50 40 30 20 10 0
f1 (ppm)

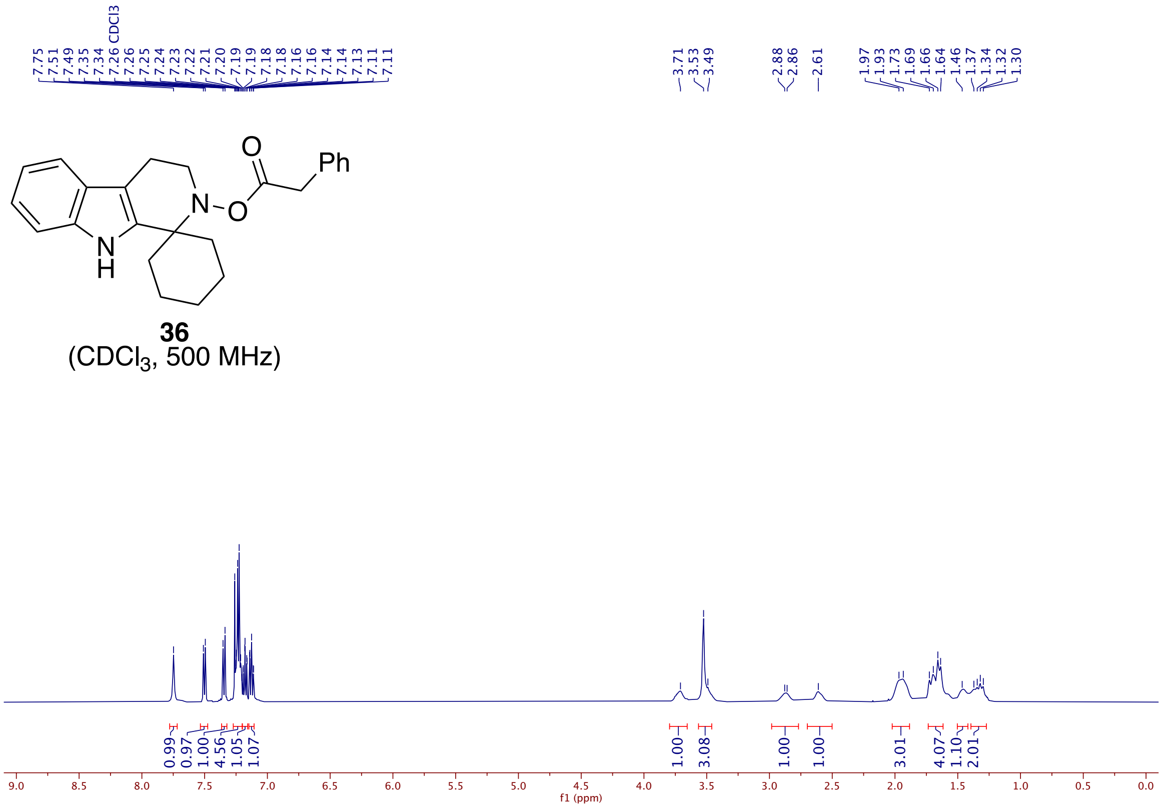
Ph
36
(CDCl3, 500 MHz)
f1 (ppm)

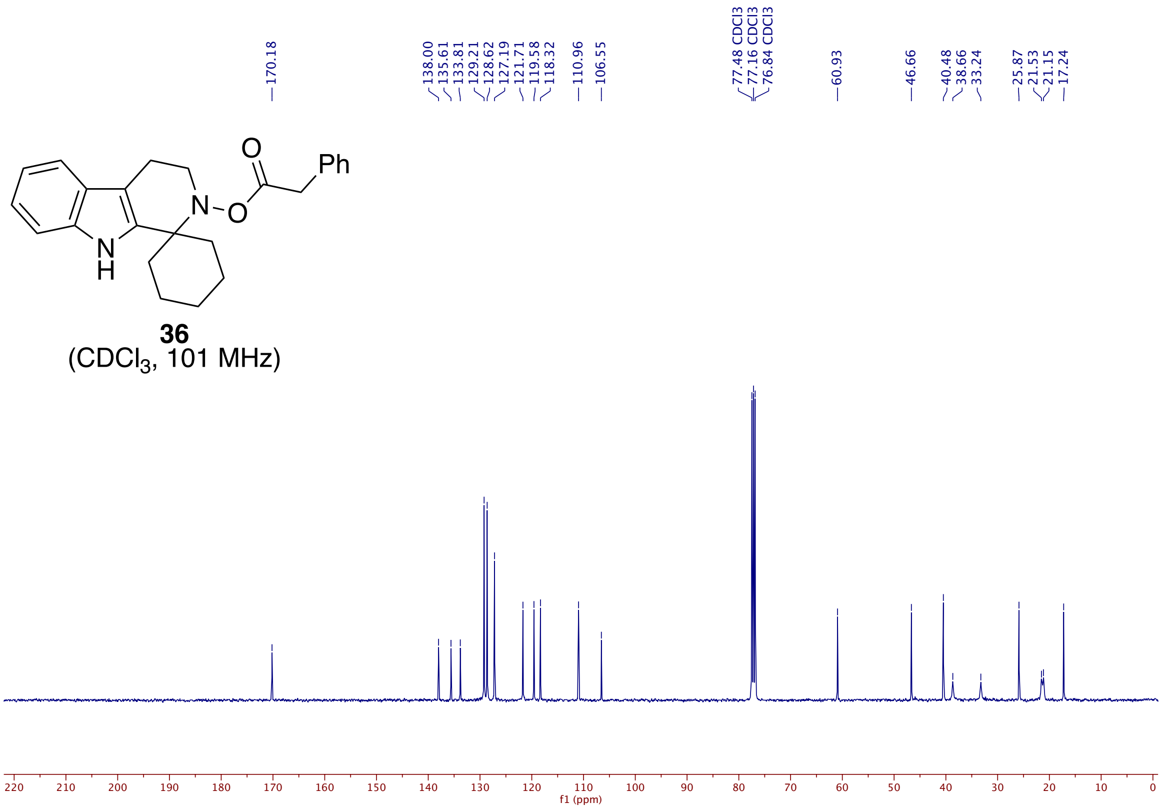
Ph
36
(CDCl3, 101 MHz)
f1 (ppm)

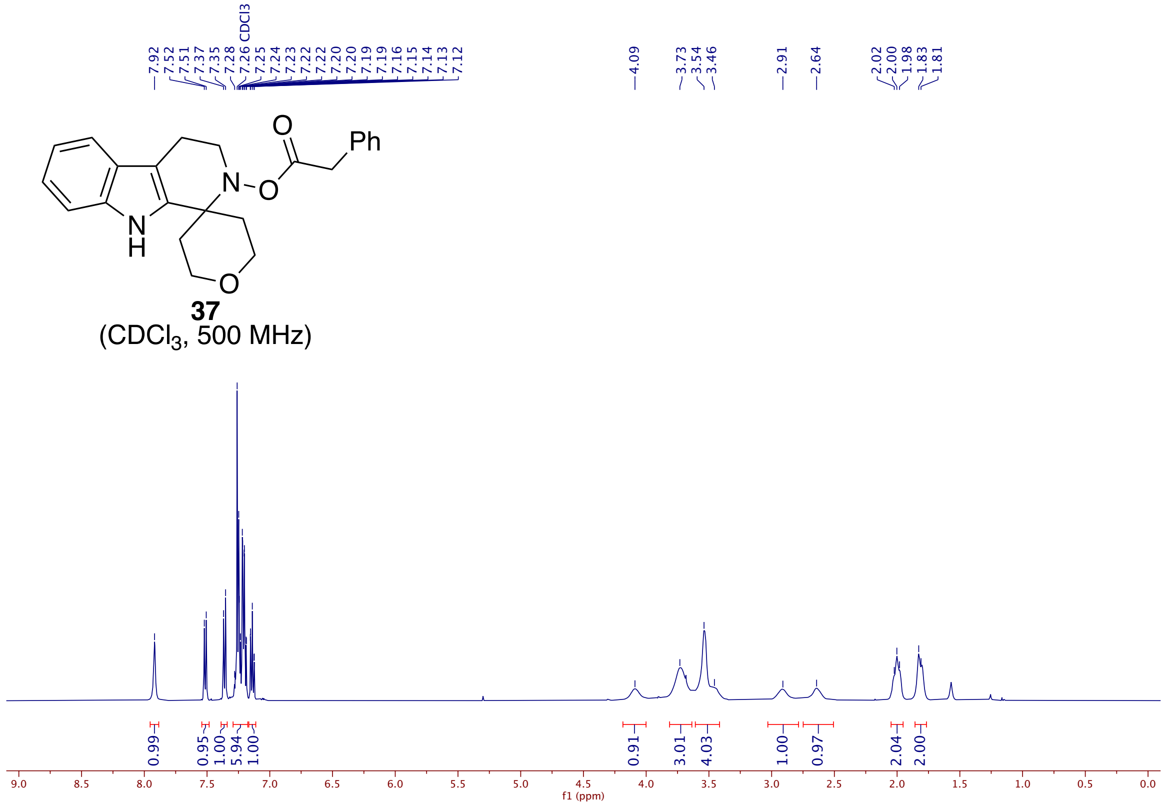

37
($CDCl_3$, 500 MHz)

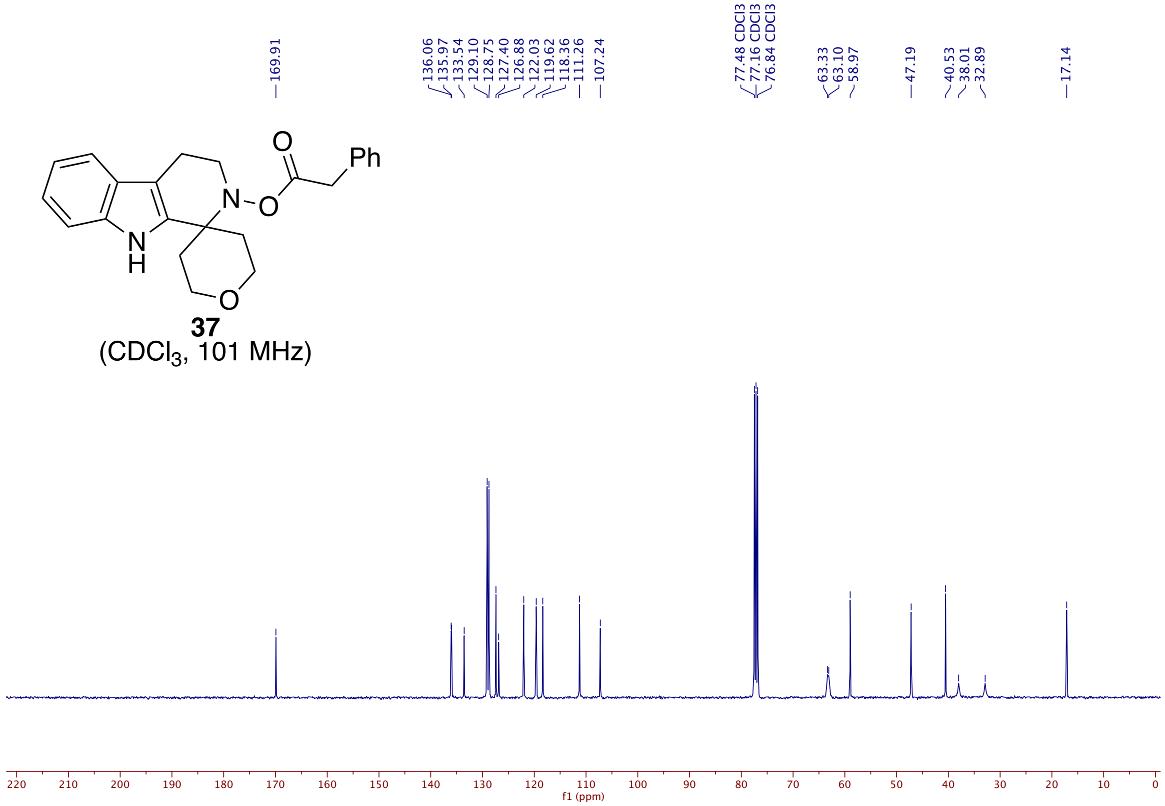

37
($CDCl_3$, 101 MHz)

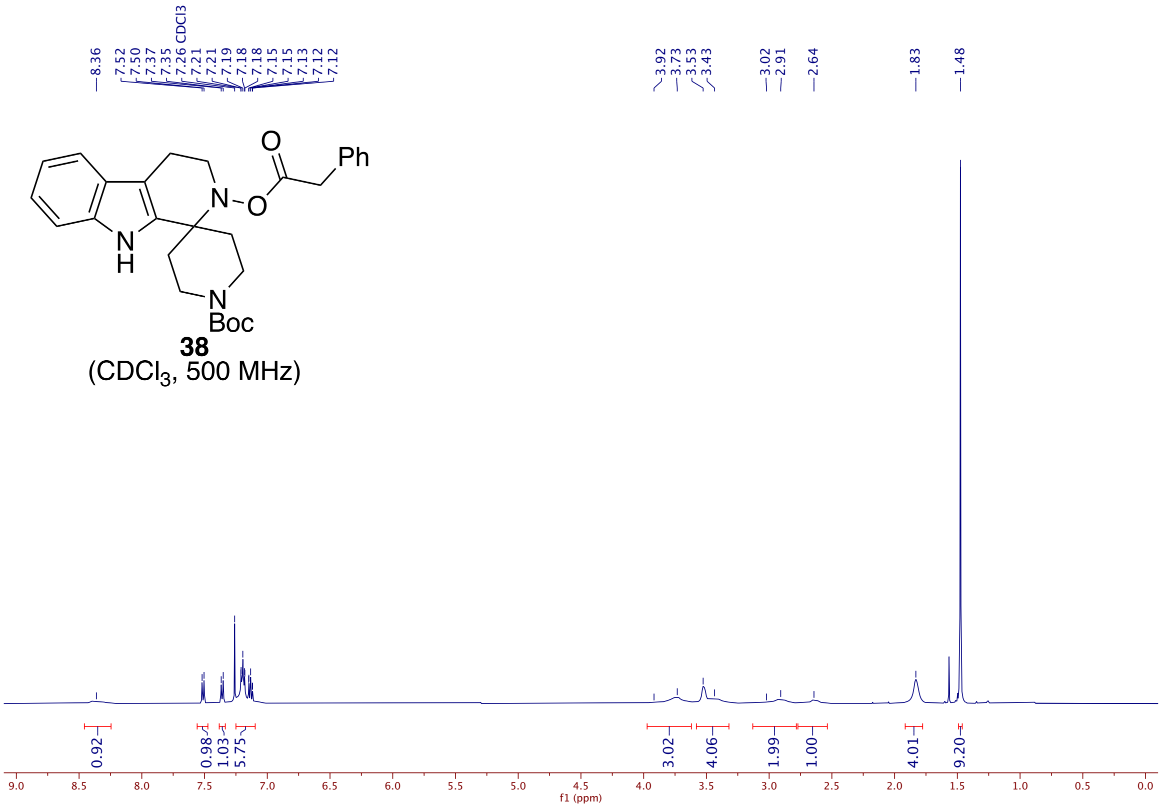
8.36
7.52
7.50
7.37
7.35
7.26 CDCl3
7.21
7.21
7.19
7.18
7.18
7.15
7.15
7.13
7.12
7.12
3.92
3.73
3.53
3.43
3.02
2.91
2.64
1.83
1.48
O
Ph
N
O
N
H
N
Boc
38
(CDCl3, 500 MHz)
0.92
0.98
1.03
5.75
3.02
4.06
1.99
1.00
4.01
9.20
f1 (ppm)

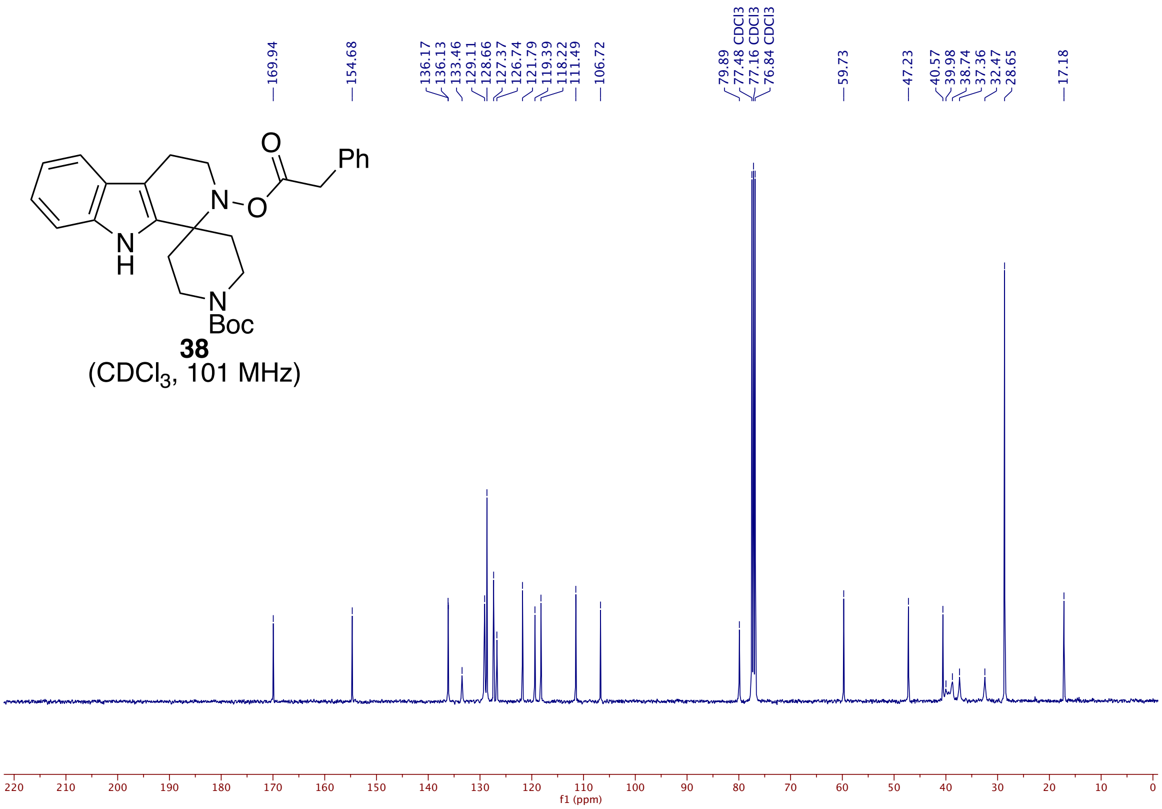
169.94
154.68
136.17
136.13
133.46
129.11
128.66
127.37
126.74
121.79
119.39
118.22
111.49
106.72
79.89
77.48 CDCl3
77.16 CDCl3
76.84 CDCl3
59.73
47.23
40.57
39.98
38.74
37.36
32.47
28.65
17.18
O
Ph
N
O
N
H
N
Boc
38
(CDCl3, 101 MHz)
f1 (ppm)

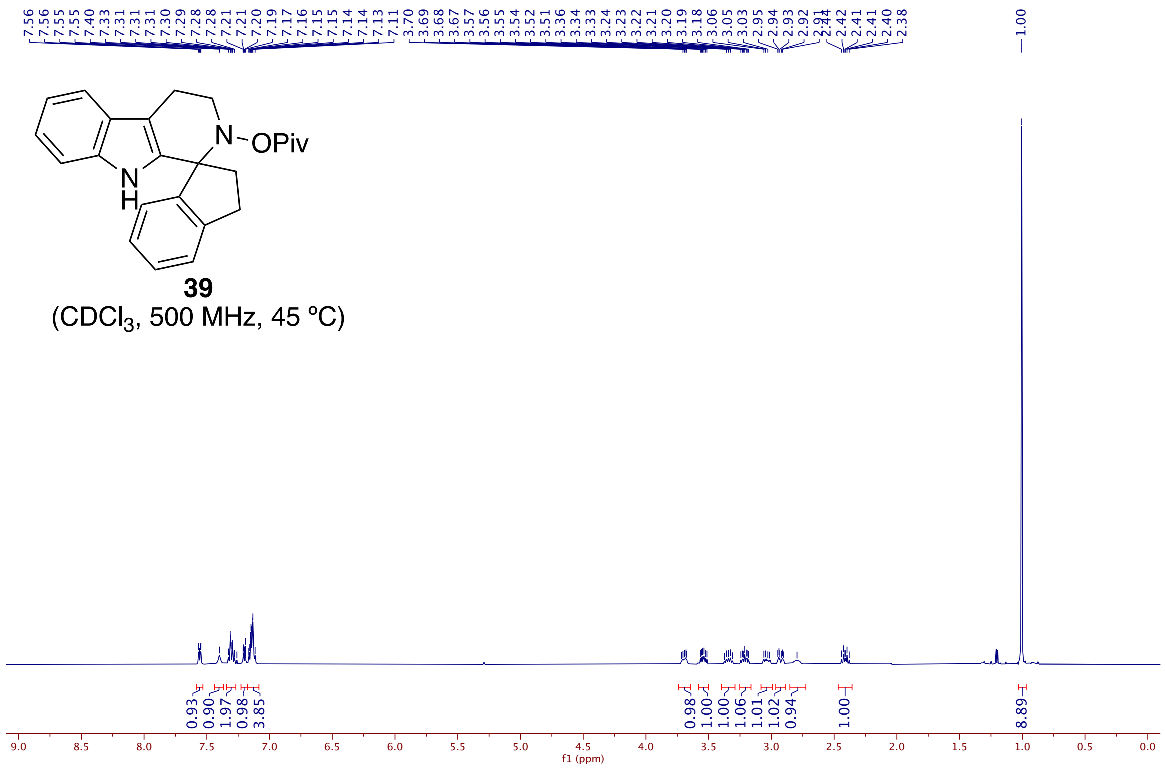
N-OPiv
N
H
39
(CDCl3, 500 MHz, 45 ºC)
f1 (ppm)

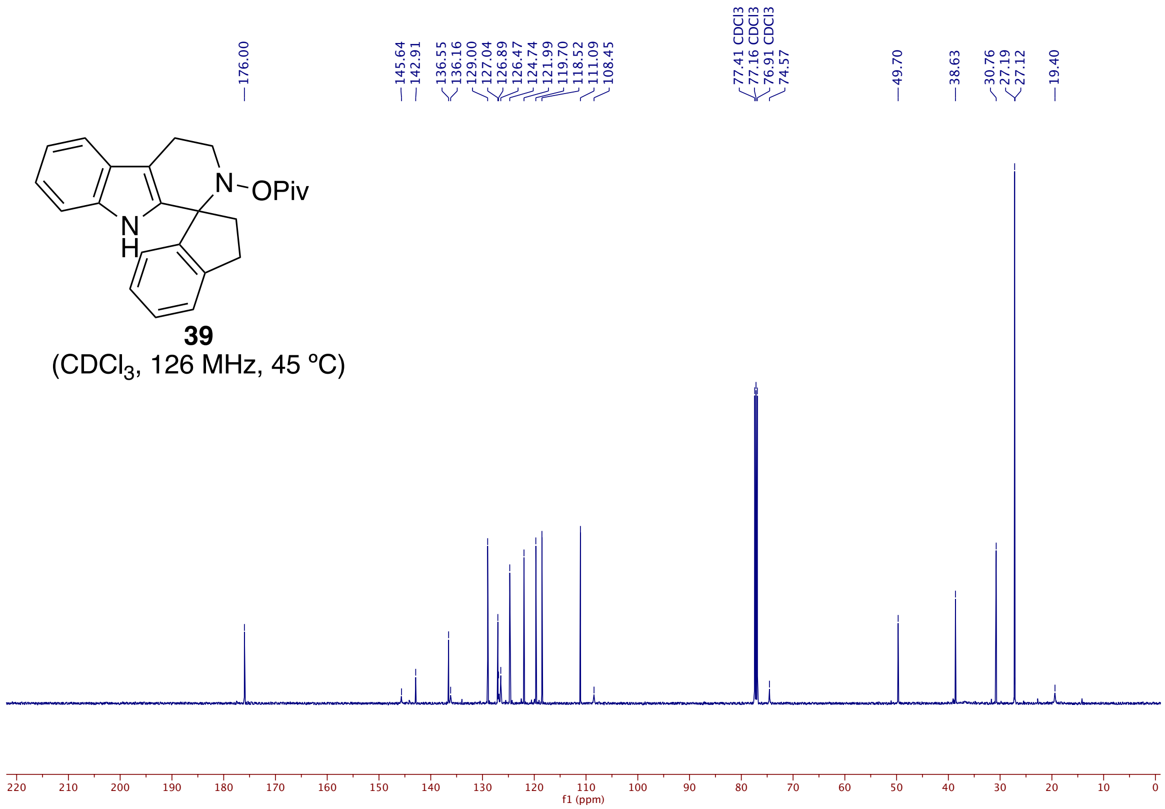
N-OPiv
N
H
39
(CDCl3, 126 MHz, 45 ºC)
77.41 CDCl3
77.16 CDCl3
76.91 CDCl3
f1 (ppm)

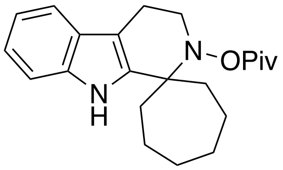

40
($CDCl_3$, 500 MHz, 45 ºC)

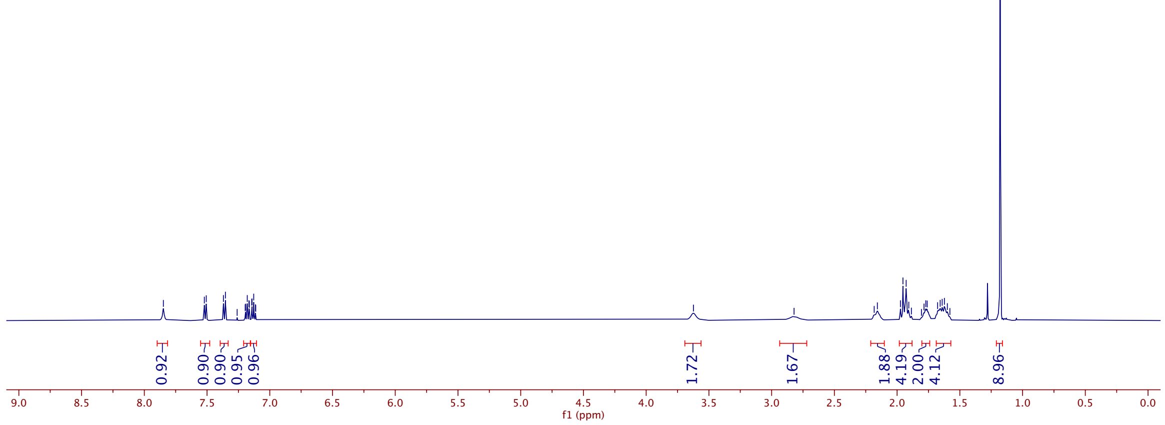

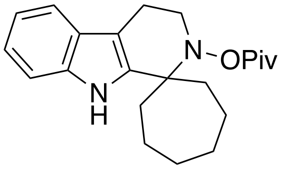

40
($CDCl_3$, 126 MHz, 45 ºC)

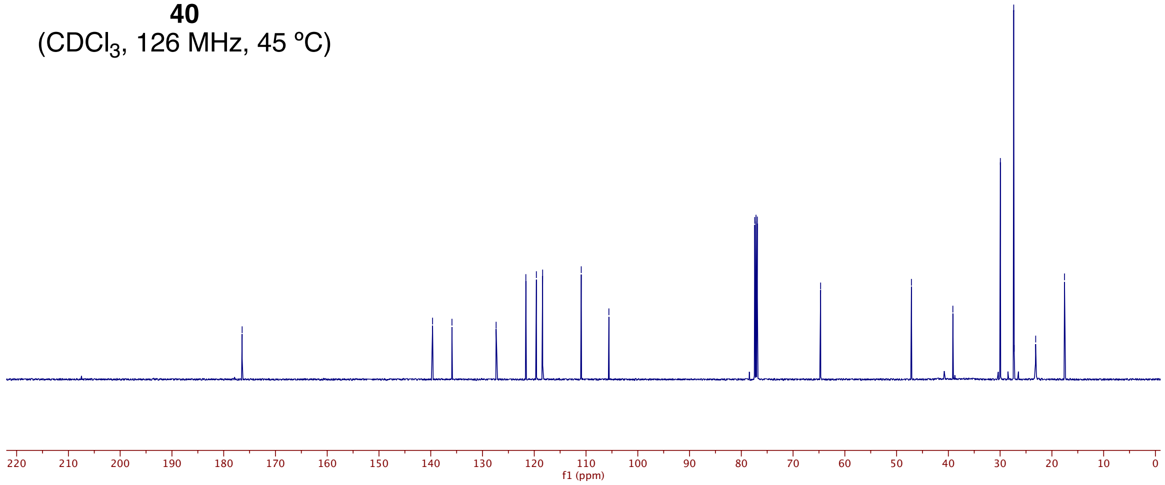

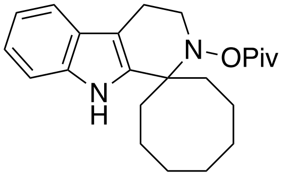

41
($CDCl_3$, 500 MHz, 45 ºC)

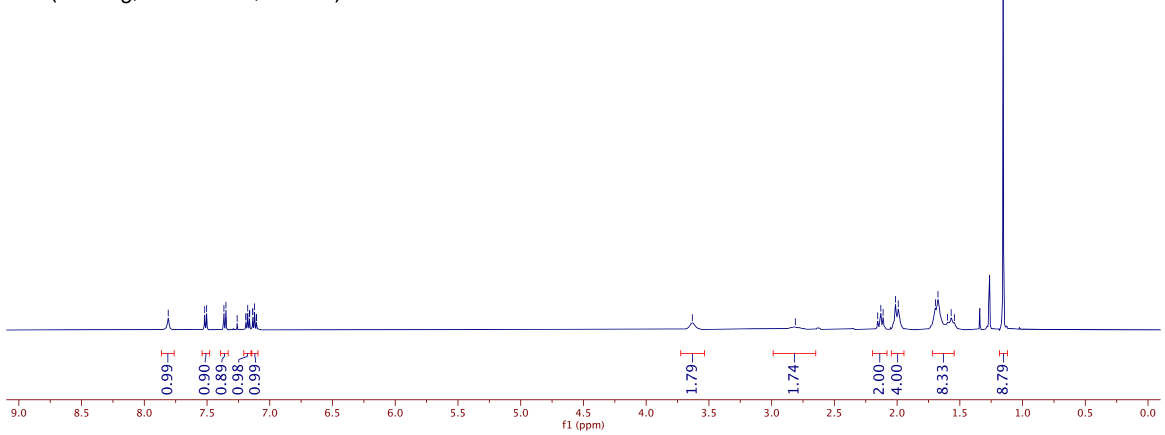

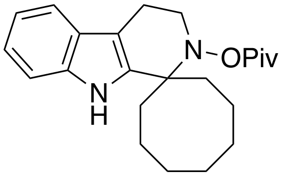

41
($CDCl_3$, 126 MHz, 45 ºC)

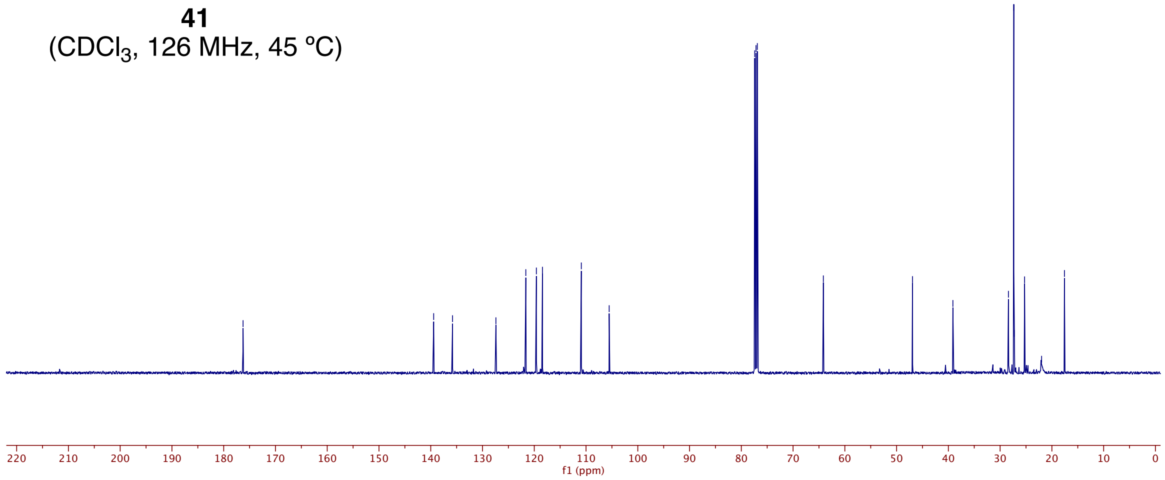

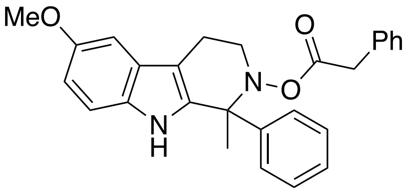

42
($CDCl_3$, 500 MHz)

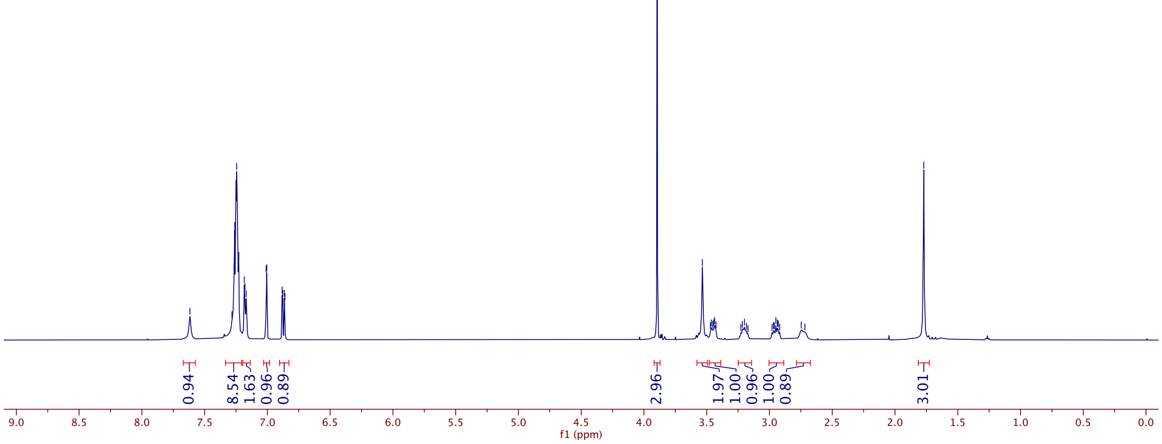

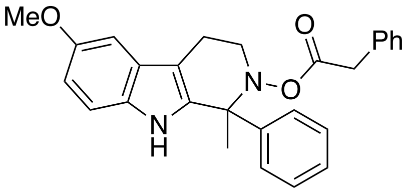

42
($CDCl_3$, 101 MHz)

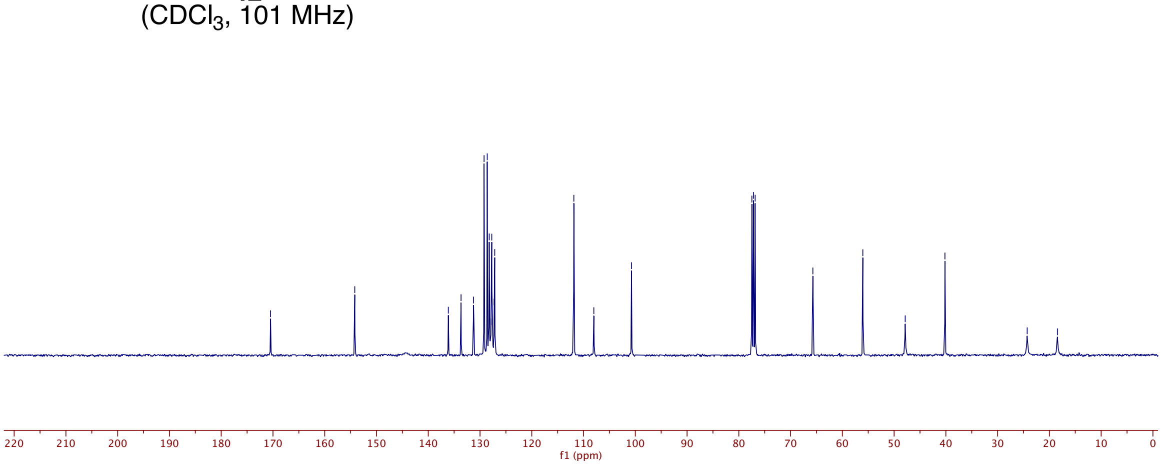

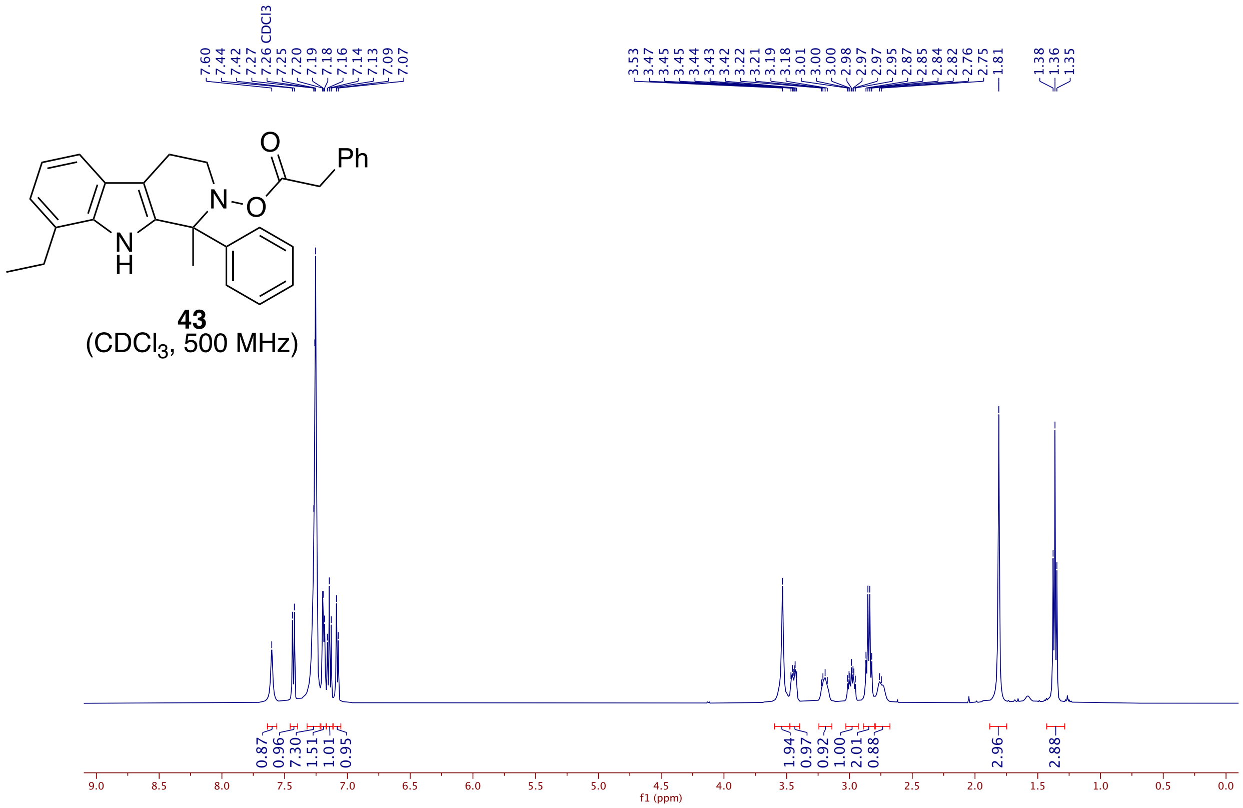

43
($CDCl_3$, 500 MHz)

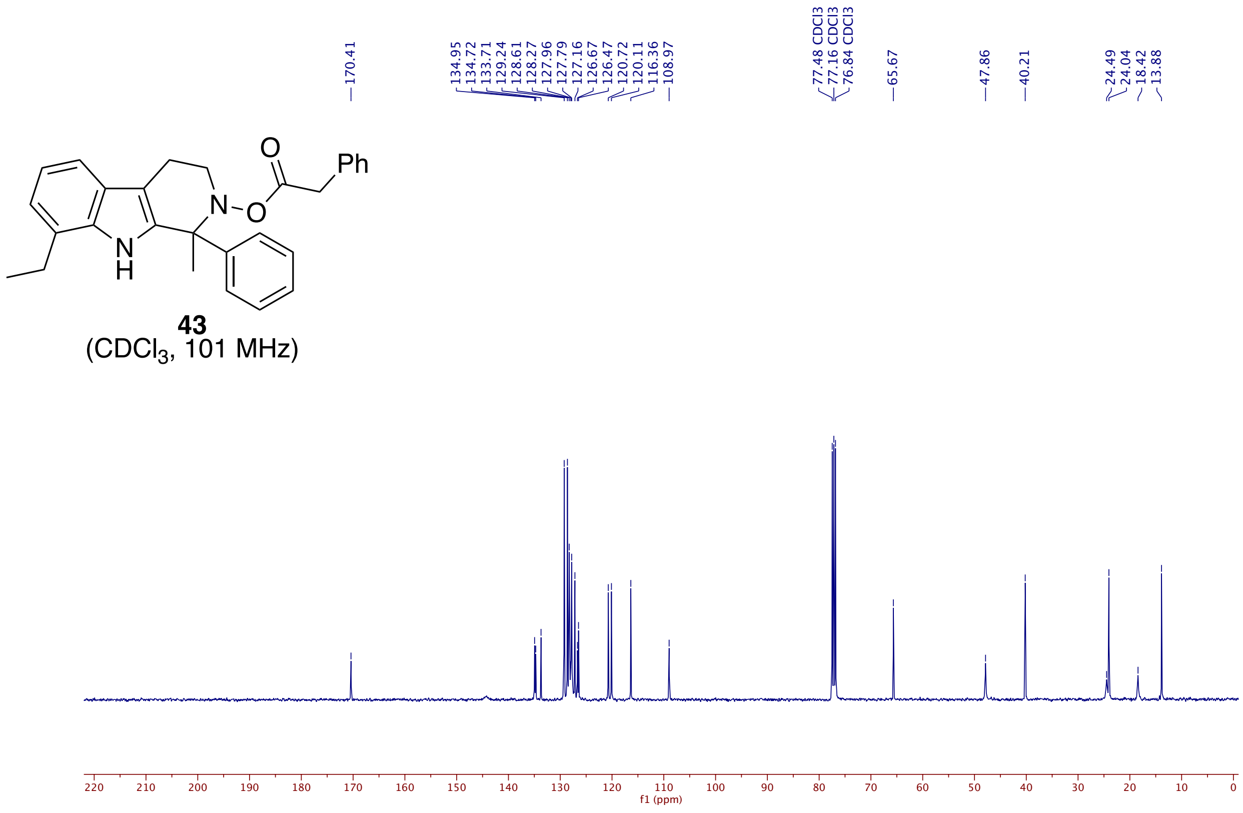

43
($CDCl_3$, 101 MHz)

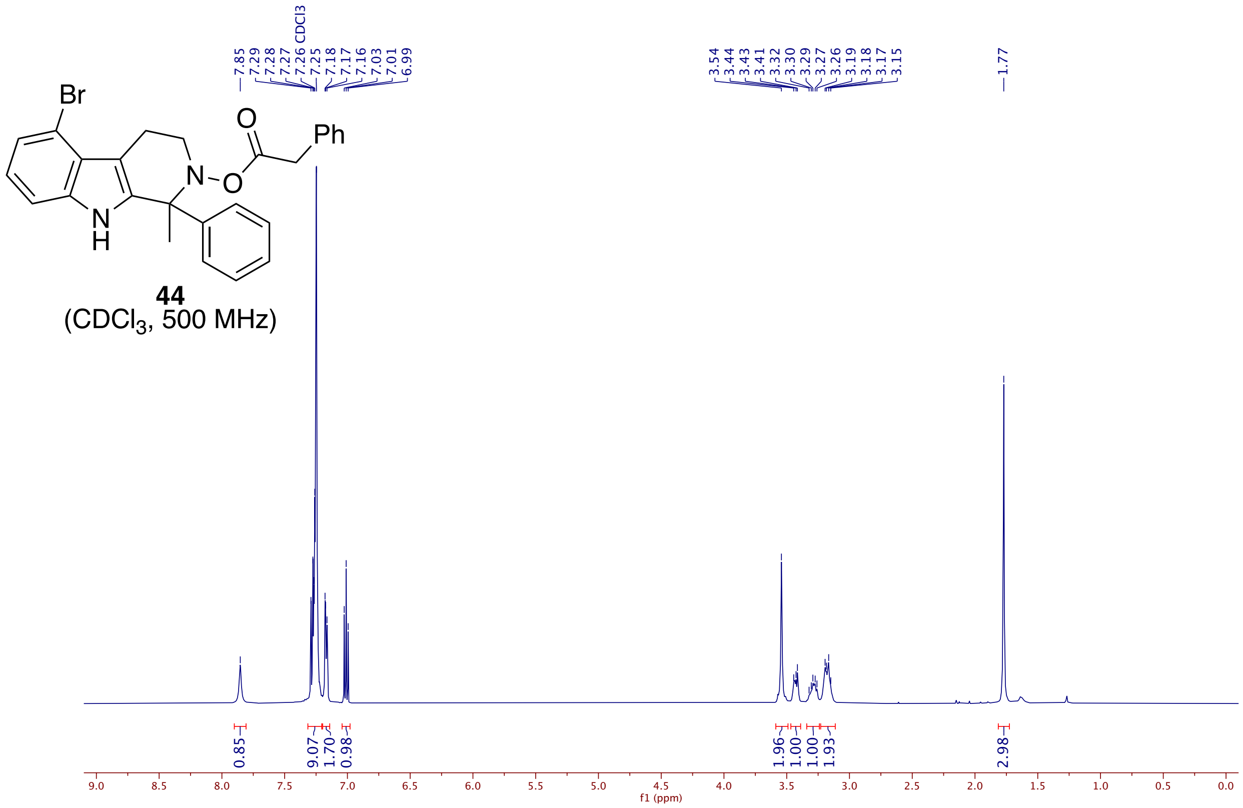

44
($CDCl_3$, 500 MHz)

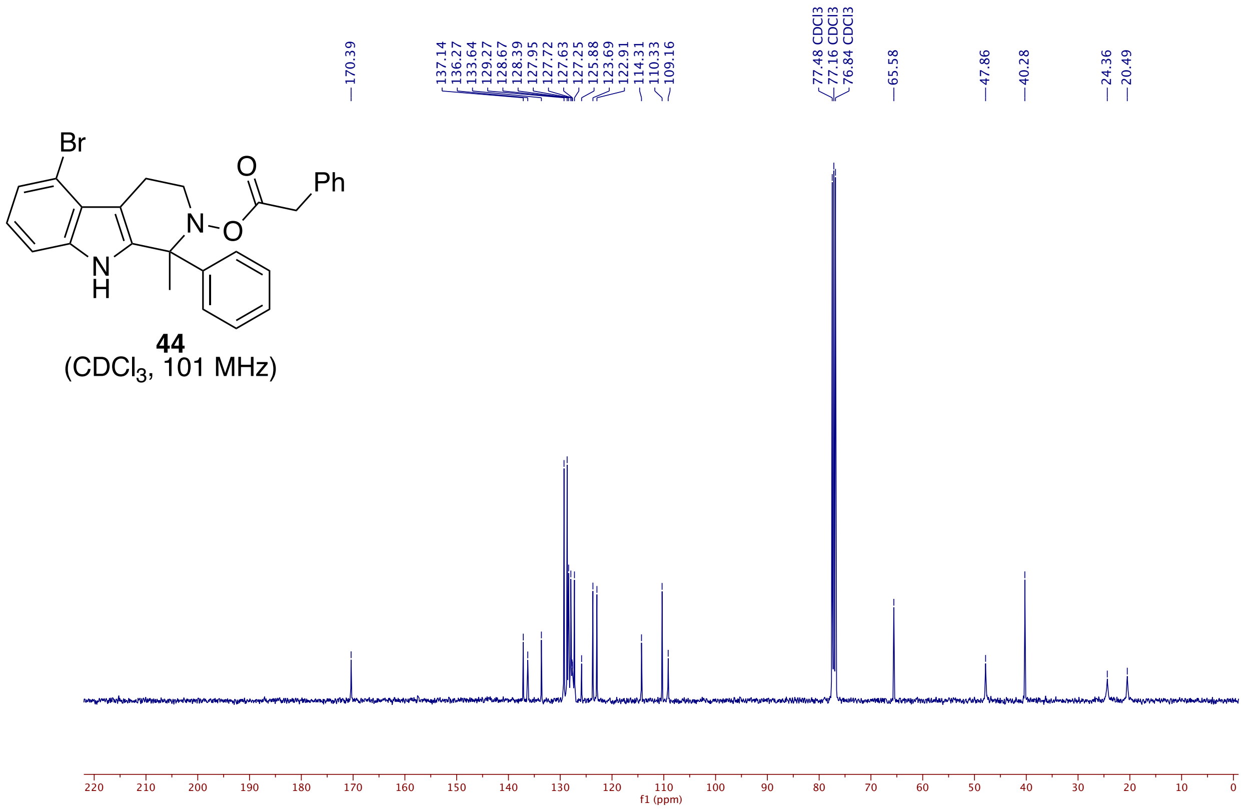

44
($CDCl_3$, 101 MHz)

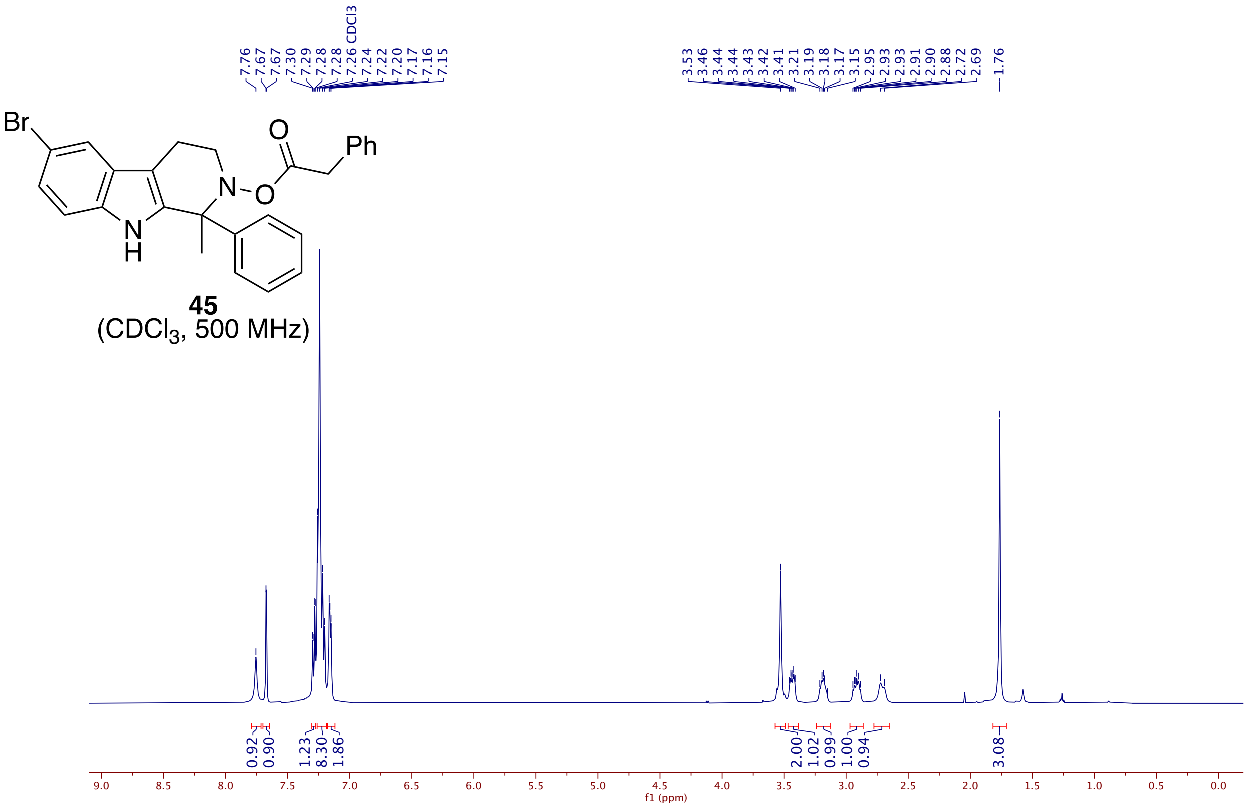

45
(CDCl$_3$, 500 MHz)

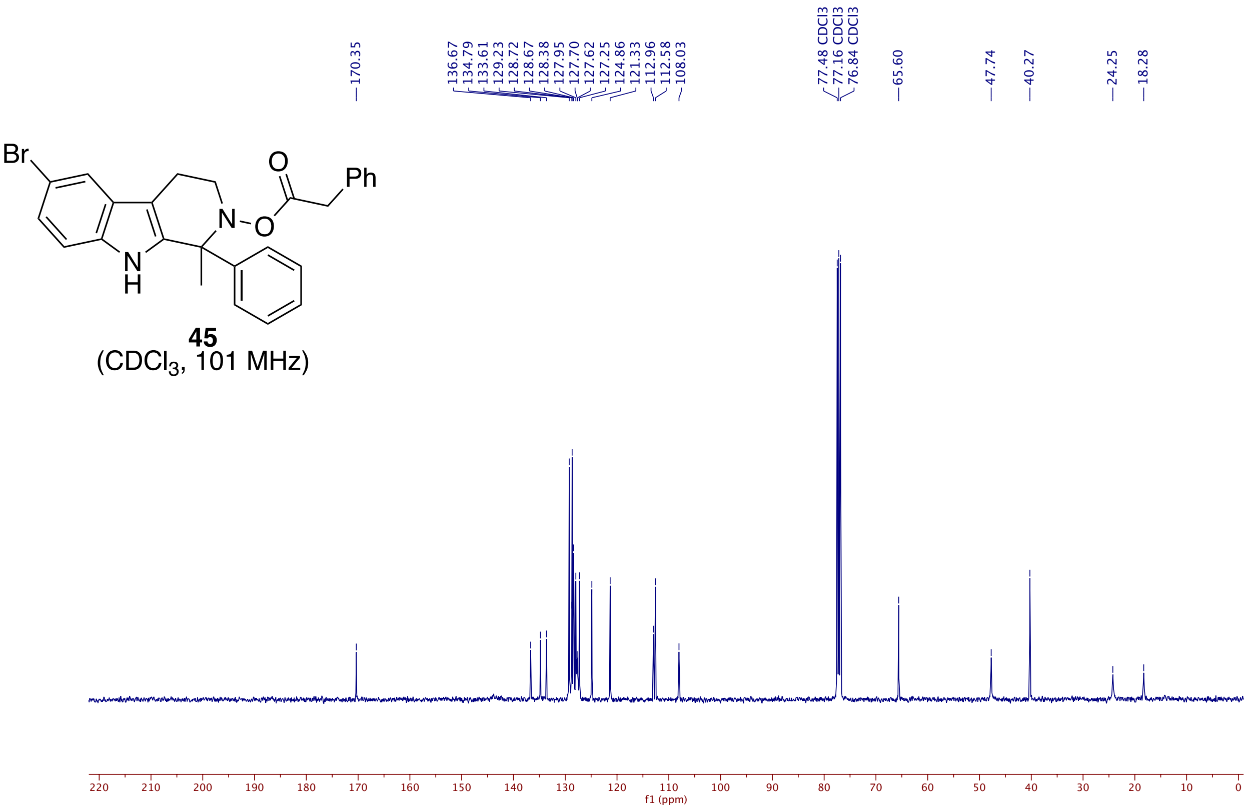

45
(CDCl$_3$, 101 MHz)

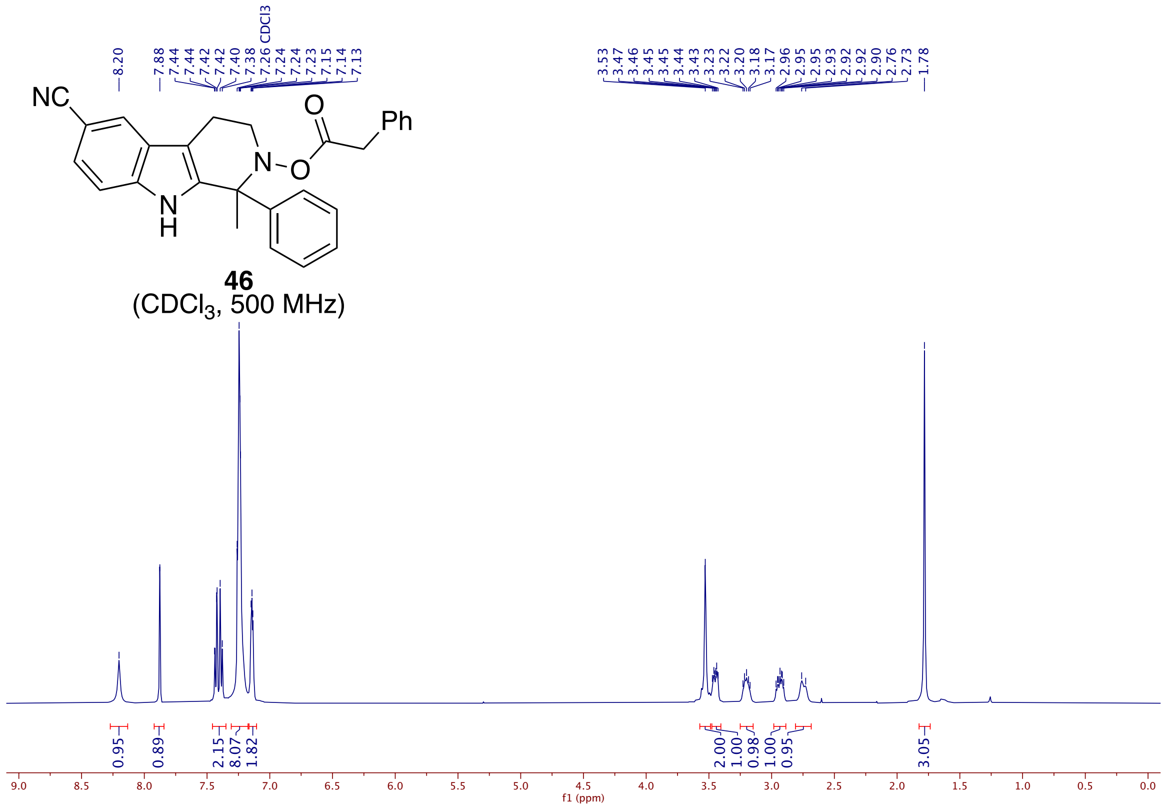
8.20
7.88
7.44
7.44
7.42
7.42
7.40
7.38
7.26 CDCl3
7.24
7.24
7.23
7.15
7.14
7.13
3.53
3.47
3.46
3.45
3.45
3.44
3.43
3.23
3.22
3.20
3.18
3.17
2.96
2.95
2.95
2.93
2.92
2.92
2.90
2.76
2.73
1.78
NC
O
Ph
N
O
N
H
46
(CDCl3, 500 MHz)
0.95
0.89
2.15
8.07
1.82
2.00
1.00
0.98
1.00
0.95
3.05
f1 (ppm)

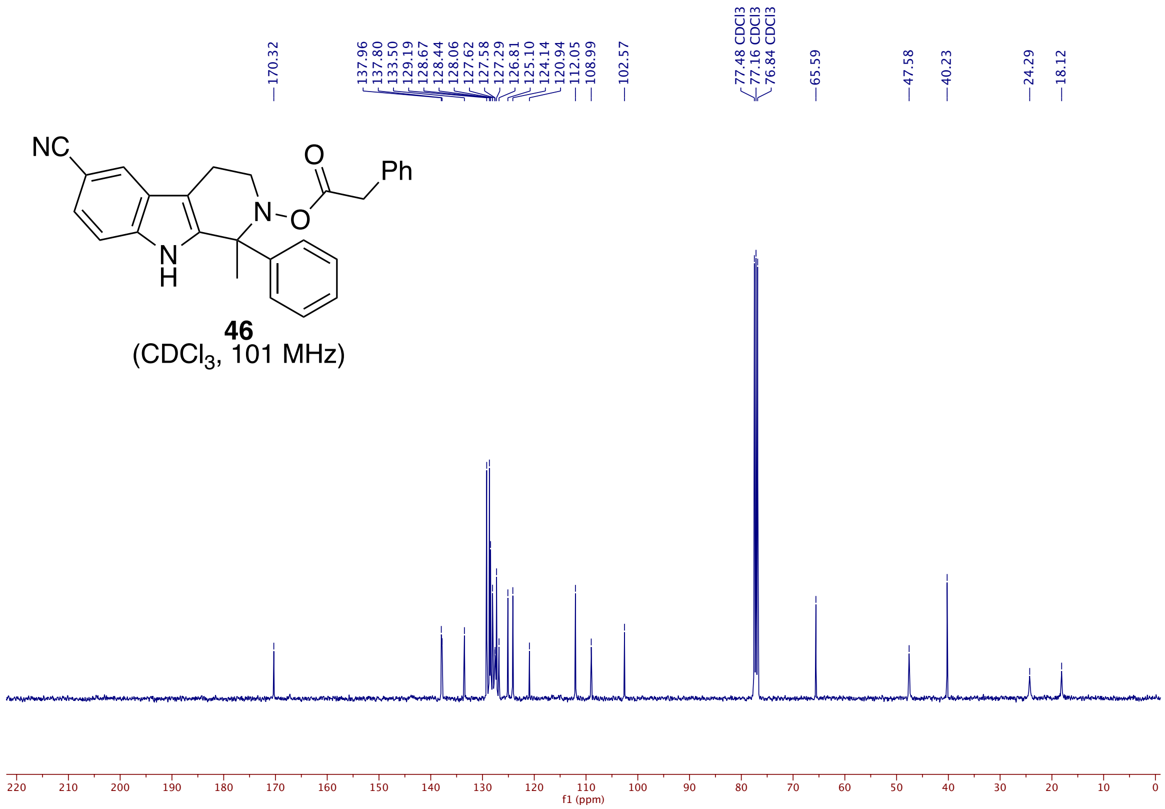
170.32
137.96
137.80
133.50
129.19
128.67
128.44
128.06
127.62
127.58
127.29
126.81
125.10
124.14
120.94
112.05
108.99
102.57
77.48 CDCl3
77.16 CDCl3
76.84 CDCl3
65.59
47.58
40.23
24.29
18.12
NC
O
Ph
N
O
N
H
46
(CDCl3, 101 MHz)
f1 (ppm)

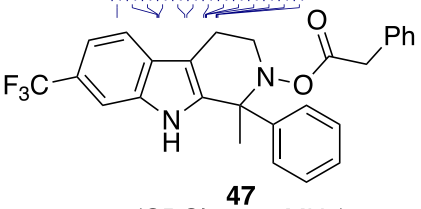

47
($CDCl_3$, 500 MHz)

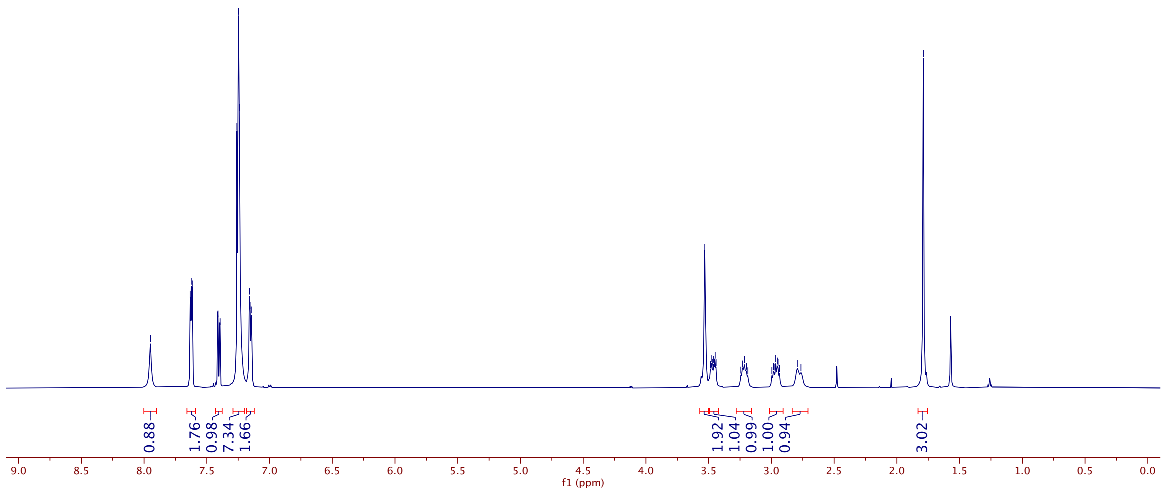

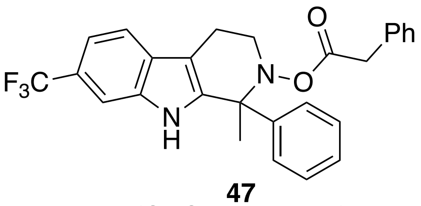

47
($CDCl_3$, 101 MHz)

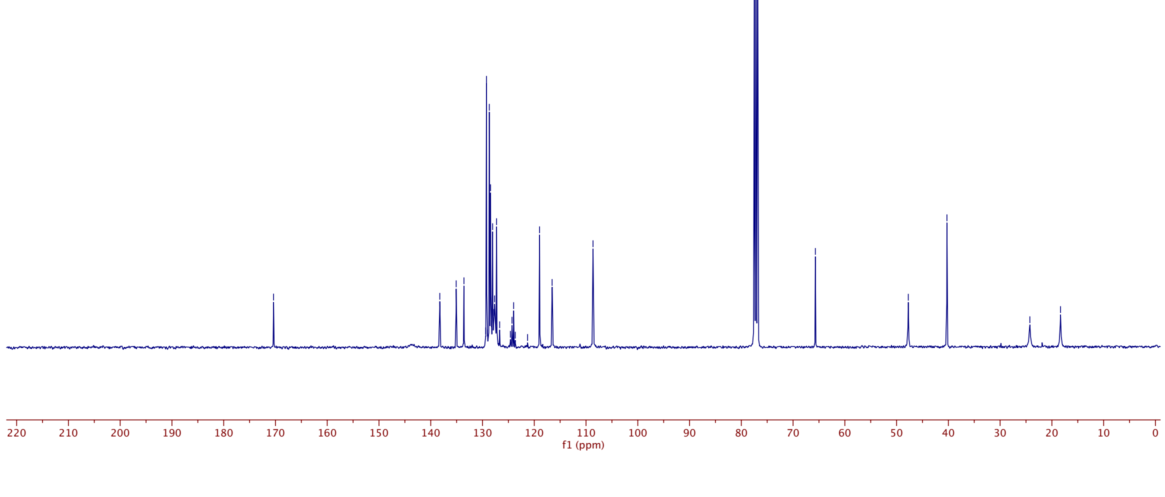

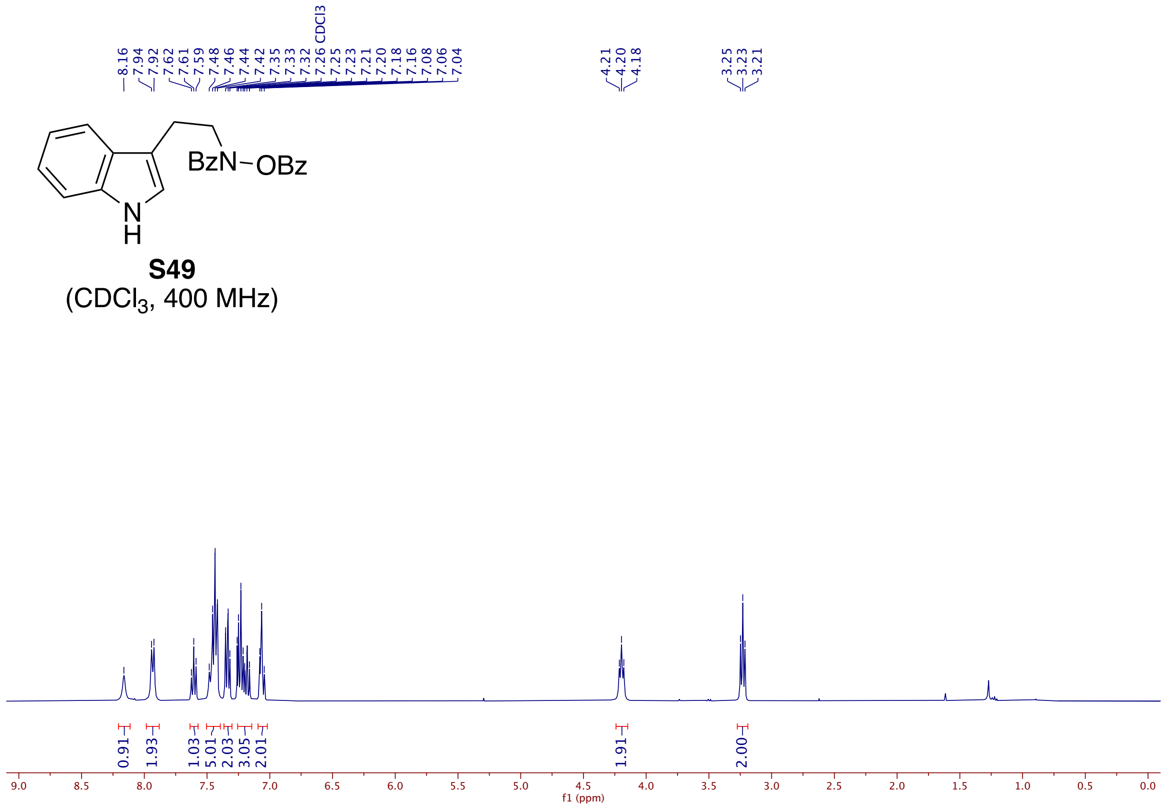

S49
($CDCl_3$, 400 MHz)

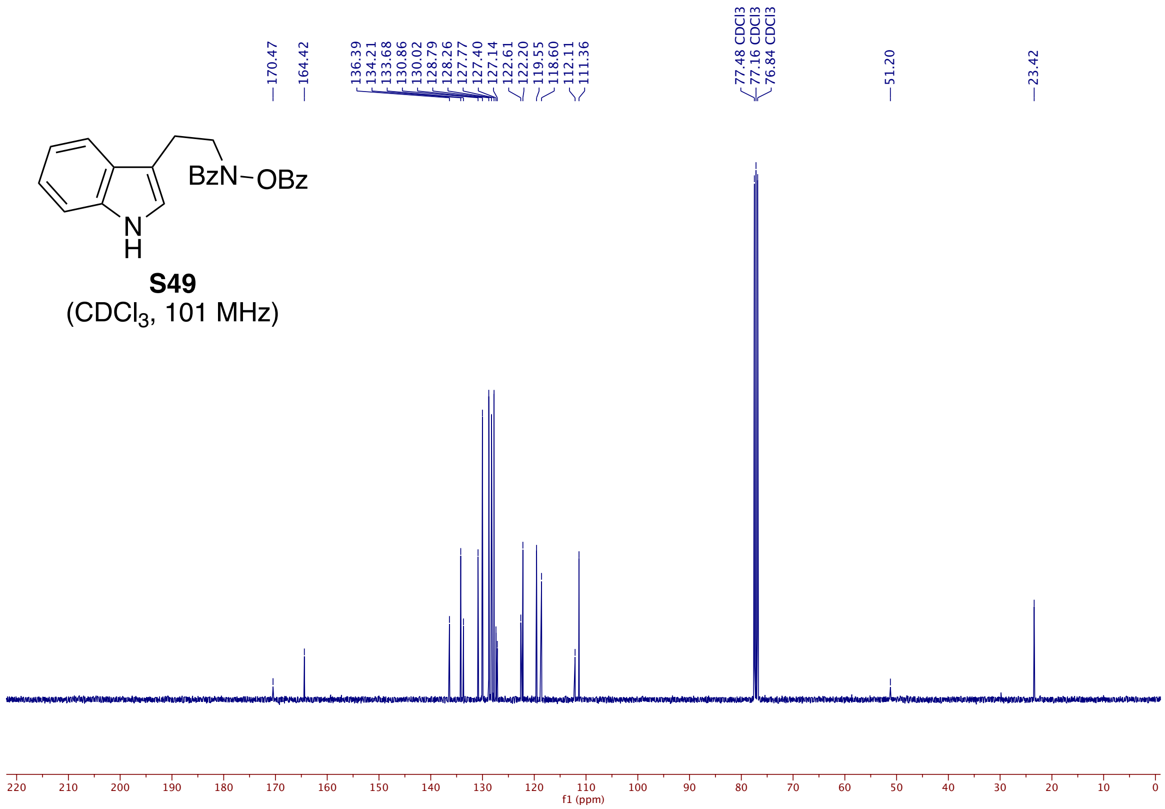

S49
($CDCl_3$, 101 MHz)

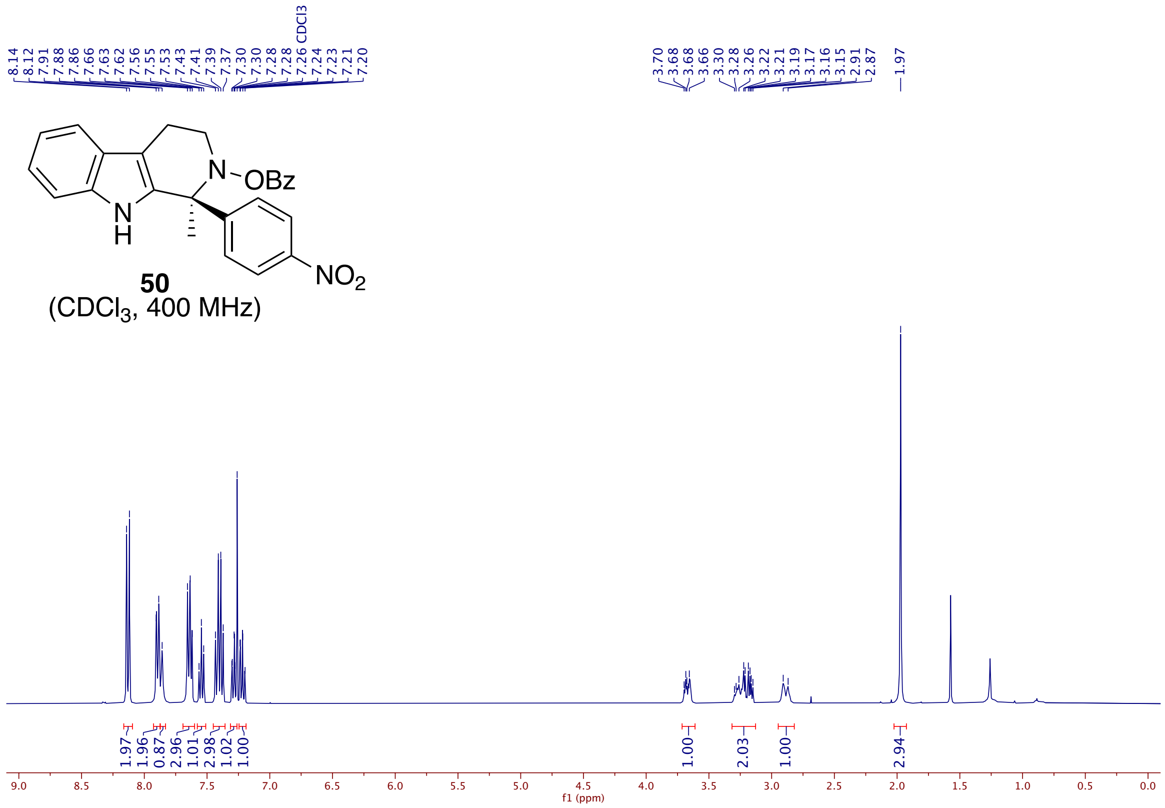
8.14 8.12 7.91 7.88 7.86 7.66 7.63 7.62 7.56 7.55 7.53 7.43 7.41 7.39 7.37 7.30 7.30 7.28 7.28 7.26 CDCl3 7.24 7.23 7.21 7.20
3.70 3.68 3.68 3.66 3.30 3.28 3.26 3.22 3.21 3.19 3.17 3.16 3.15 2.91 2.87
1.97
N
OBz
N
H
NO2
50
(CDCl3, 400 MHz)
1.97 1.96 0.87 2.96 1.01 2.98 1.02 1.00
1.00 2.03 1.00 2.94
f1 (ppm)

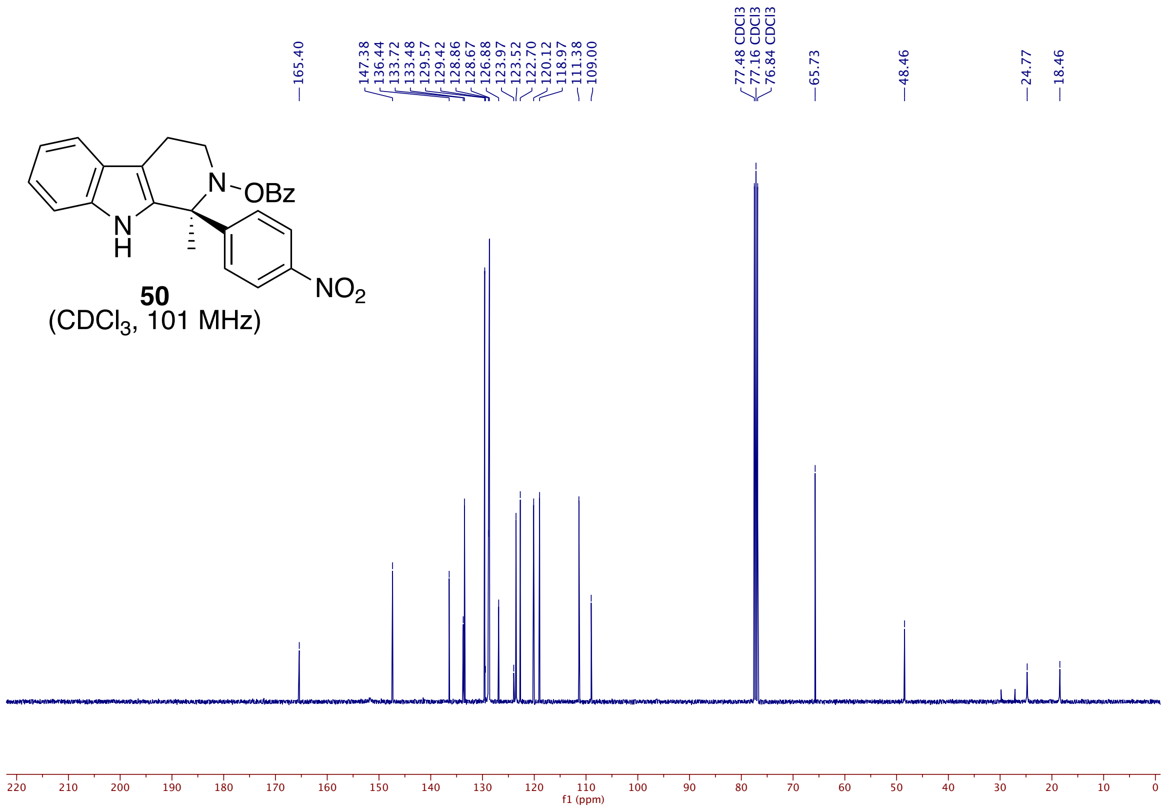
165.40
147.38 136.44 133.72 133.48 129.57 129.42 128.86 128.67 126.88 123.97 123.52 122.70 120.12 118.97 111.38 109.00
77.48 CDCl3 77.16 CDCl3 76.84 CDCl3
65.73
48.46
24.77
18.46
N
OBz
N
H
NO2
50
(CDCl3, 101 MHz)
f1 (ppm)

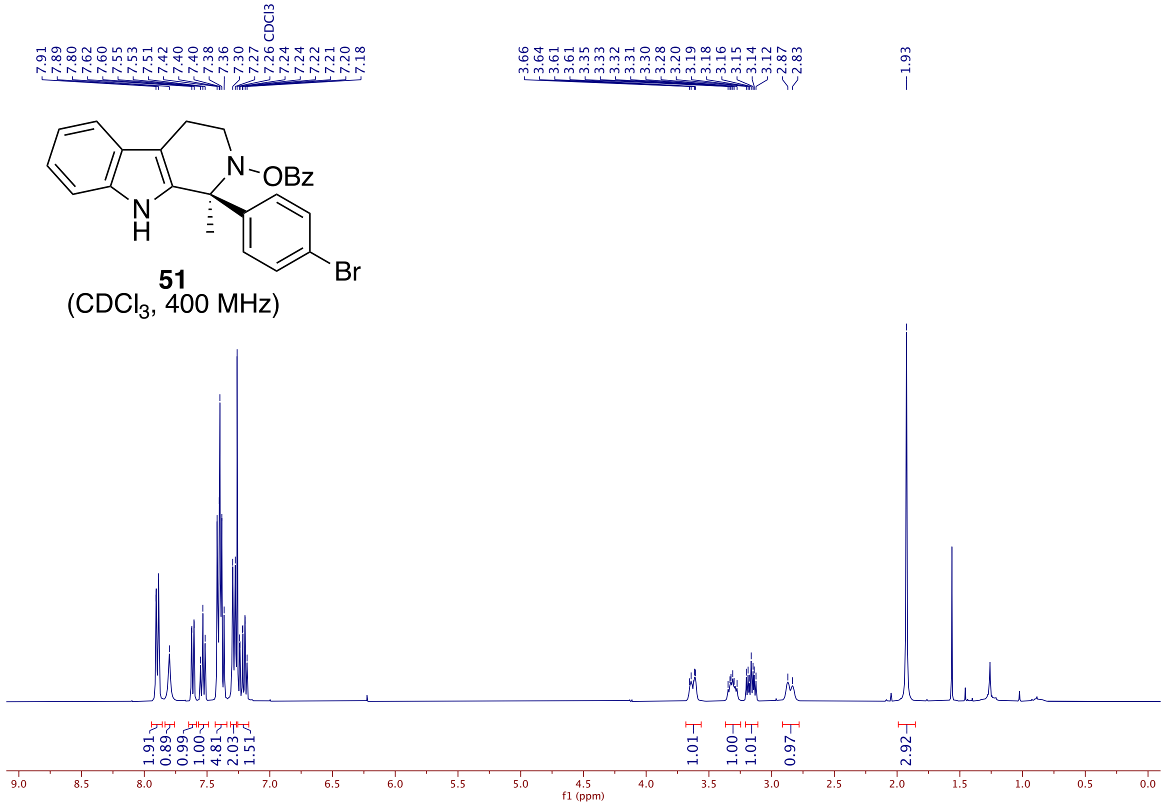
7.91 7.89 7.80 7.62 7.60 7.55 7.53 7.51 7.42 7.40 7.40 7.38 7.36 7.30 7.27 7.26 CDCl3 7.24 7.24 7.22 7.21 7.20 7.18
3.66 3.64 3.61 3.61 3.35 3.33 3.32 3.31 3.30 3.28 3.20 3.19 3.18 3.16 3.15 3.14 3.12 2.87 2.83
1.93
N
OBz
N
H
Br
51
(CDCl3, 400 MHz)
1.91 0.89 0.99 1.00 4.81 2.03 1.51
1.01 1.00 1.01 0.97 2.92
9.0 8.5 8.0 7.5 7.0 6.5 6.0 5.5 5.0 4.5 4.0 3.5 3.0 2.5 2.0 1.5 1.0 0.5 0.0
f1 (ppm)

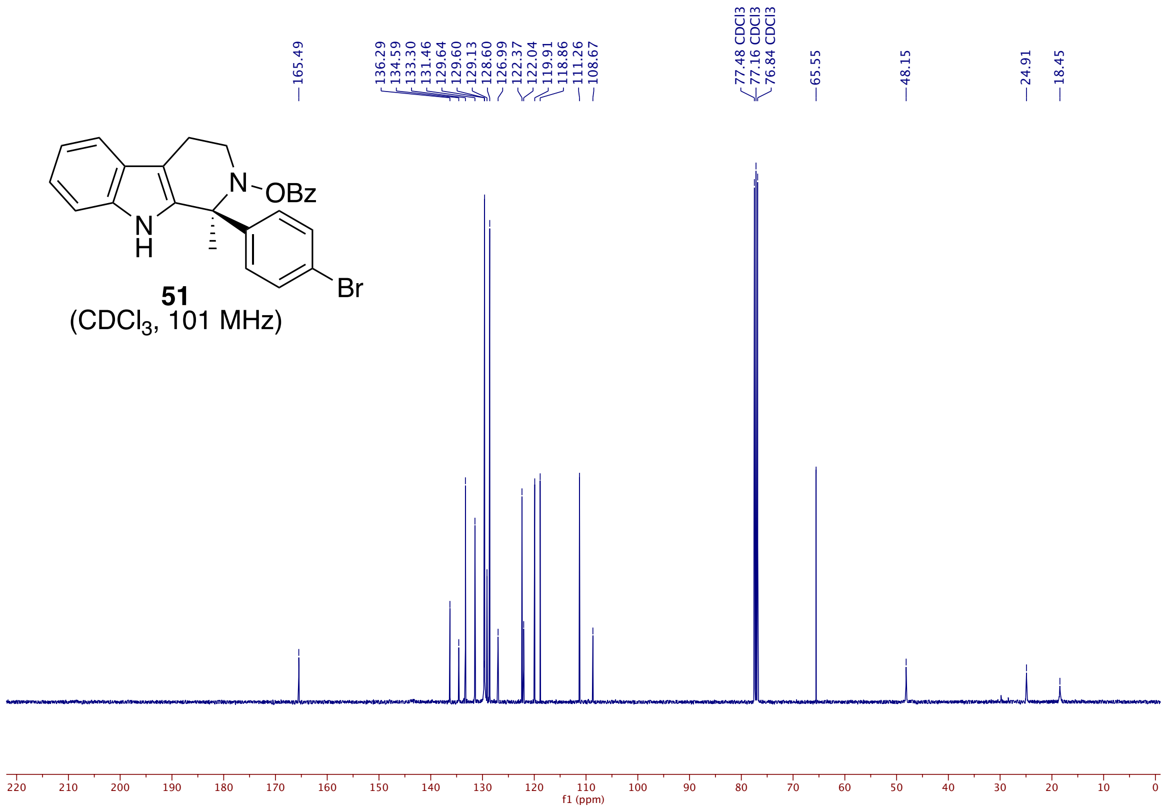
165.49
136.29 134.59 133.30 131.46 129.64 129.60 129.13 128.60 126.99 122.37 122.04 119.91 118.86 111.26 108.67
77.48 CDCl3 77.16 CDCl3 76.84 CDCl3
65.55
48.15
24.91
18.45
N
OBz
N
H
Br
51
(CDCl3, 101 MHz)
220 210 200 190 180 170 160 150 140 130 120 110 100 90 80 70 60 50 40 30 20 10 0
f1 (ppm)

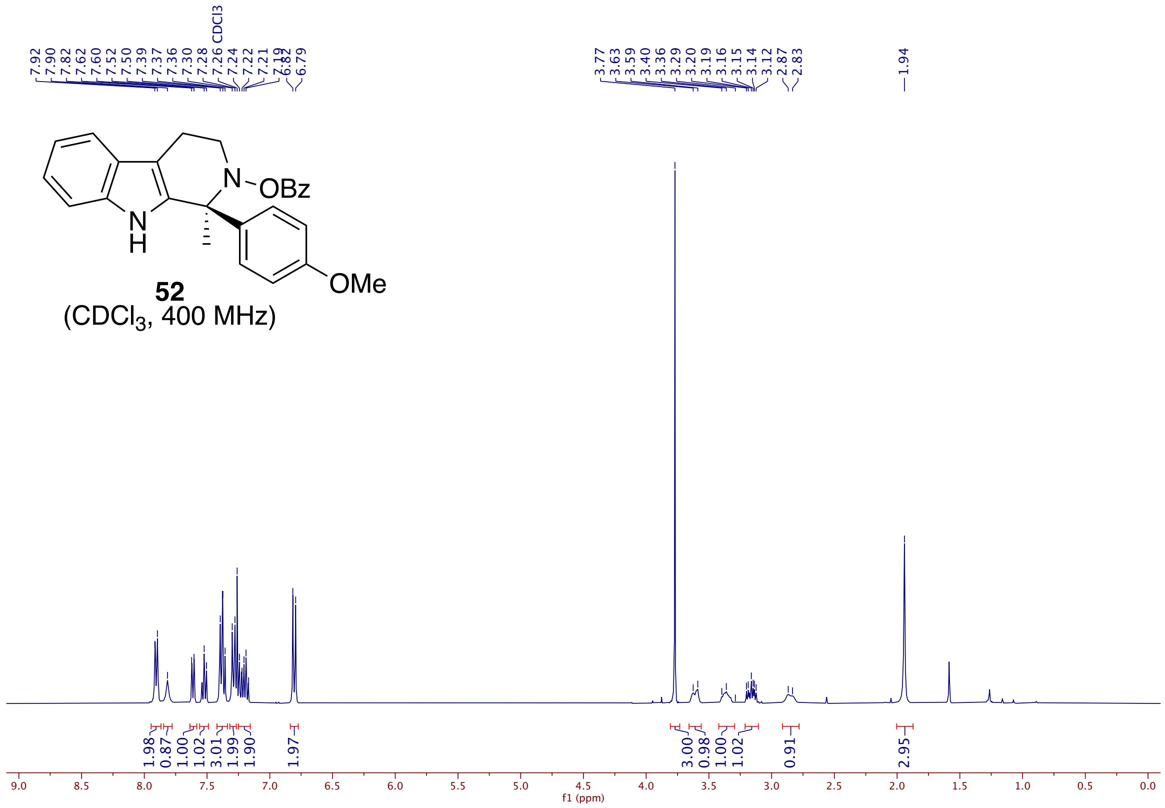
7.92
7.90
7.82
7.62
7.60
7.52
7.50
7.39
7.37
7.36
7.30
7.28
7.26 CDCl3
7.24
7.22
7.21
7.19
6.82
6.79
3.77
3.63
3.59
3.40
3.36
3.29
3.20
3.19
3.16
3.15
3.14
3.12
2.87
2.83
1.94
N
OBz
N
H
OMe
52
(CDCl3, 400 MHz)
1.98
0.87
1.00
1.02
3.01
1.99
1.90
1.97
3.00
0.98
1.00
1.02
0.91
2.95
f1 (ppm)

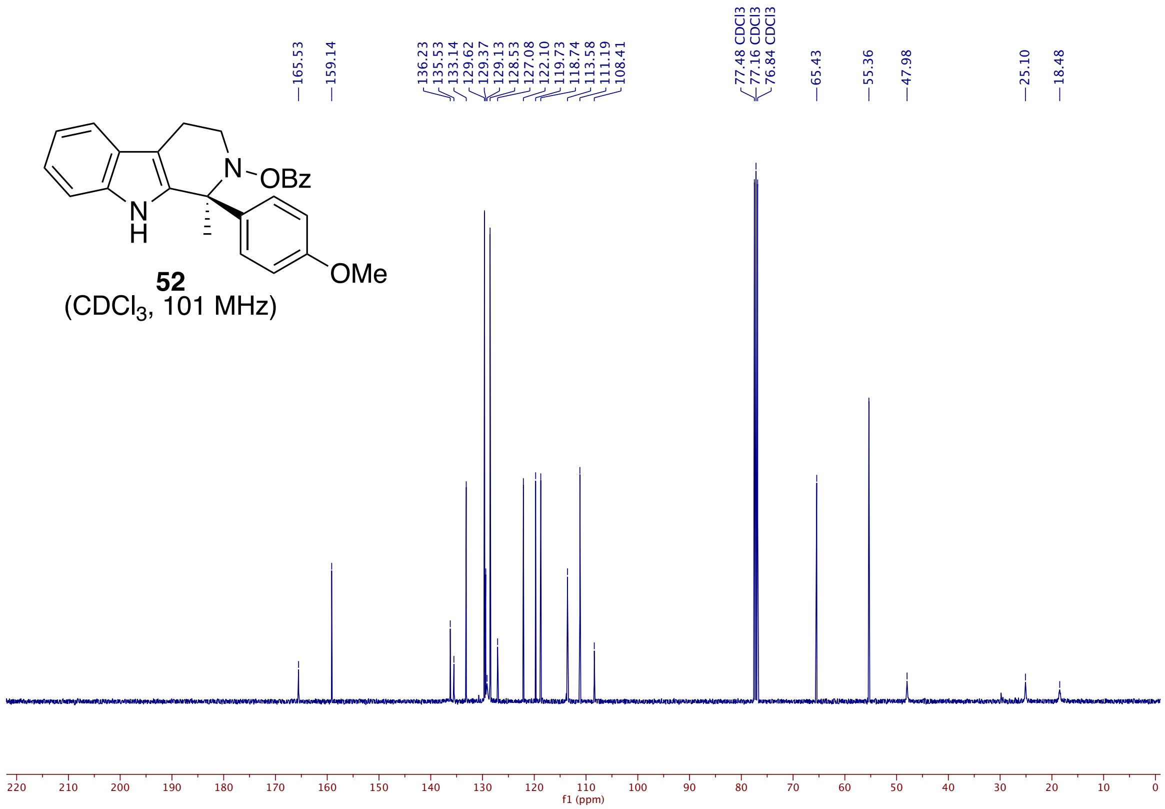
165.53
159.14
136.23
135.53
133.14
129.62
129.37
129.13
128.53
127.08
122.10
119.73
118.74
113.58
111.19
108.41
77.48 CDCl3
77.16 CDCl3
76.84 CDCl3
65.43
55.36
47.98
25.10
18.48
N
OBz
N
H
OMe
52
(CDCl3, 101 MHz)
f1 (ppm)

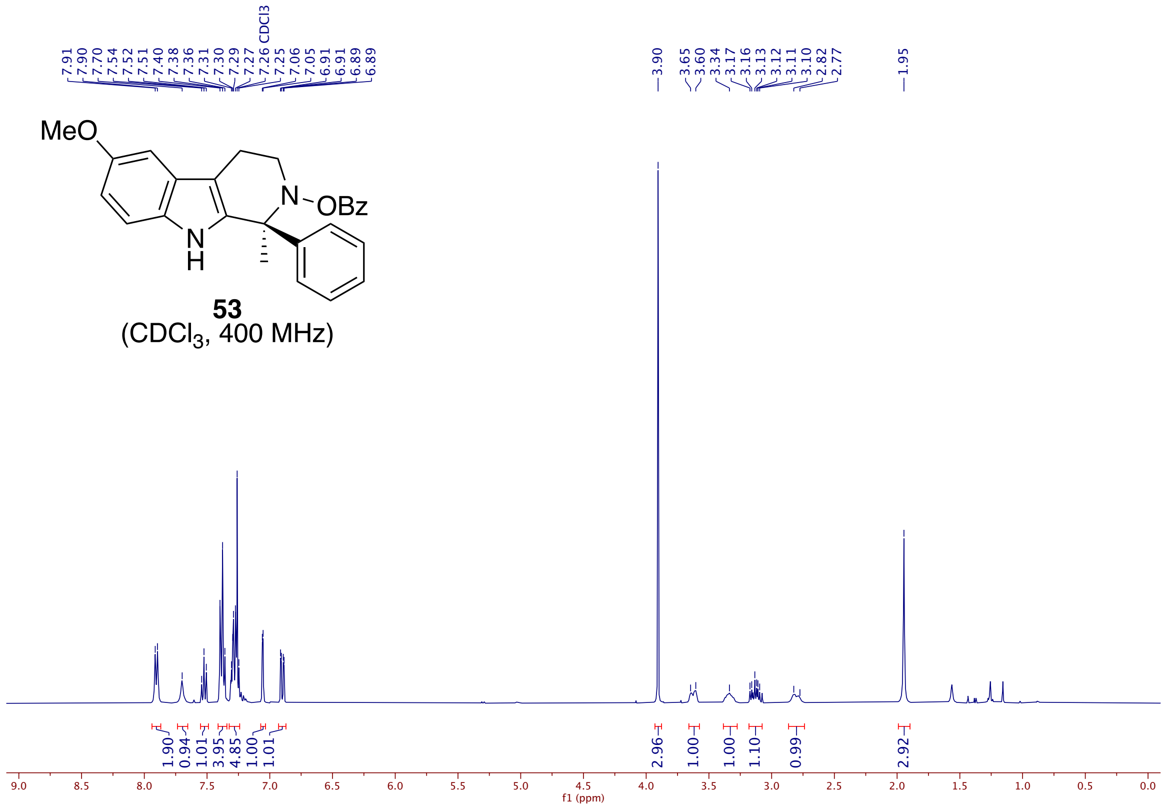
7.91
7.90
7.70
7.54
7.52
7.51
7.40
7.38
7.36
7.31
7.30
7.29
7.27
7.26 CDCl3
7.25
7.06
7.05
6.91
6.91
6.89
6.89
3.90
3.65
3.60
3.34
3.17
3.16
3.13
3.12
3.11
3.10
2.82
2.77
1.95
MeO
N
OBz
N
H
53
(CDCl3, 400 MHz)
1.90
0.94
1.01
3.95
4.85
1.00
1.01
2.96
1.00
1.00
1.10
0.99
2.92
f1 (ppm)

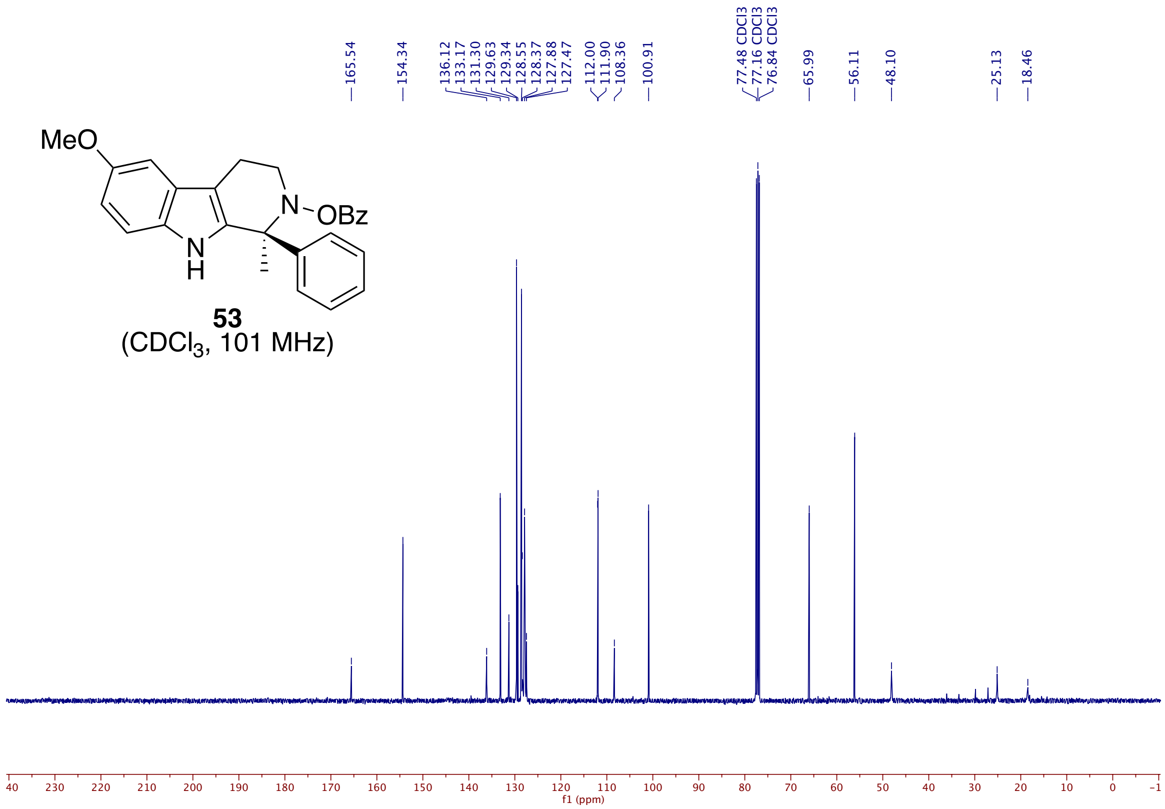
165.54
154.34
136.12
133.17
131.30
129.63
129.34
128.55
128.37
127.88
127.47
112.00
111.90
108.36
100.91
77.48 CDCl3
77.16 CDCl3
76.84 CDCl3
65.99
56.11
48.10
25.13
18.46
MeO
N
OBz
N
H
53
(CDCl3, 101 MHz)
f1 (ppm)

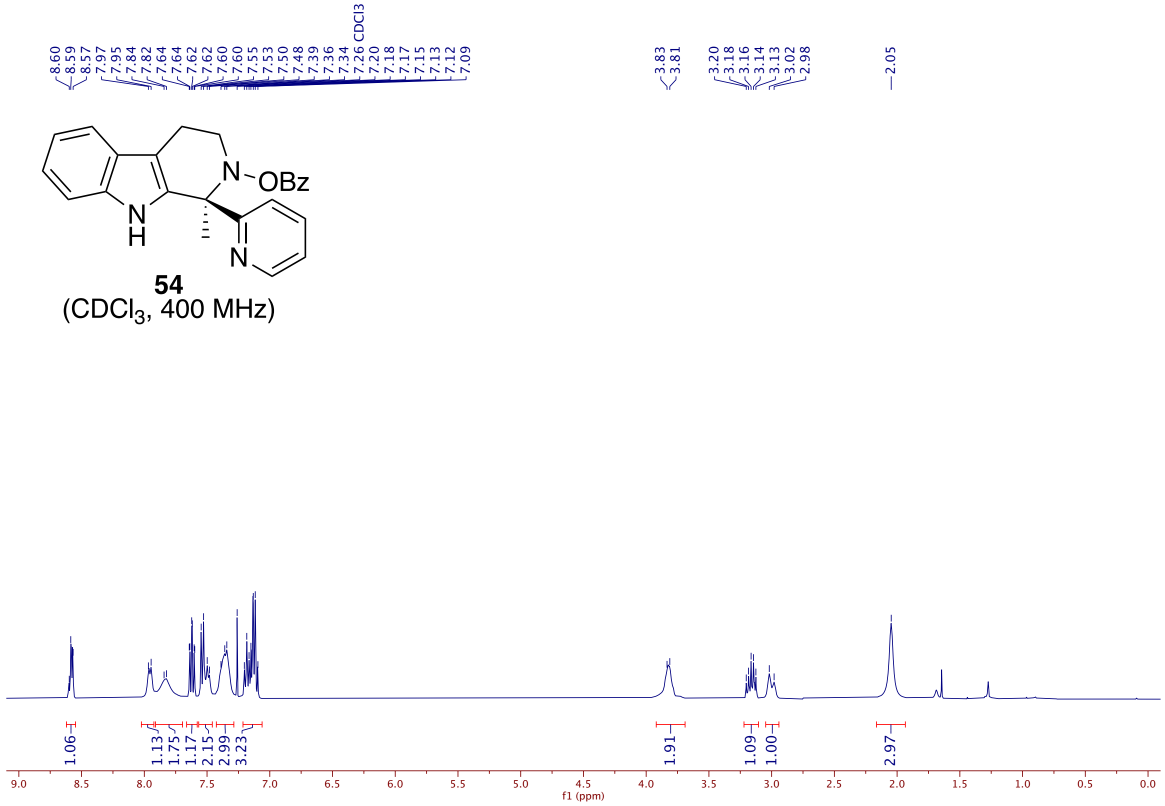
8.60 8.59 8.57 7.97 7.95 7.84 7.82 7.64 7.62 7.62 7.60 7.60 7.55 7.53 7.50 7.48 7.39 7.36 7.34 7.26 CDCl3 7.20 7.18 7.17 7.15 7.13 7.12 7.09
3.83 3.81
3.20 3.18 3.16 3.14 3.13 3.02 2.98
2.05
N-OBz
N
H
N
54
(CDCl3, 400 MHz)
1.06 1.13 1.75 1.17 2.15 2.99 3.23 1.91 1.09 1.00 2.97
f1 (ppm)

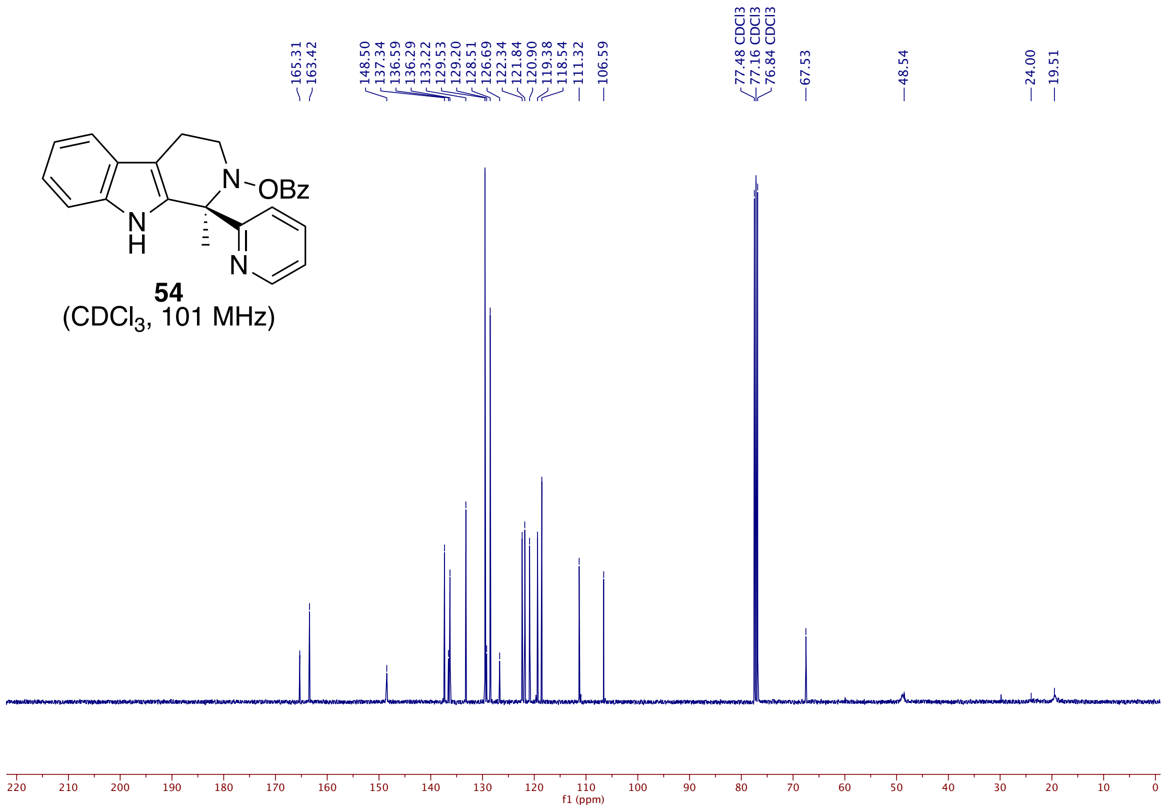
165.31 163.42 148.50 137.34 136.59 136.29 133.22 129.53 129.20 128.51 126.69 122.34 121.84 120.90 119.38 118.54 111.32 106.59
77.48 CDCl3 77.16 CDCl3 76.84 CDCl3 67.53 48.54 24.00 19.51
N-OBz
N
H
N
54
(CDCl3, 101 MHz)
f1 (ppm)

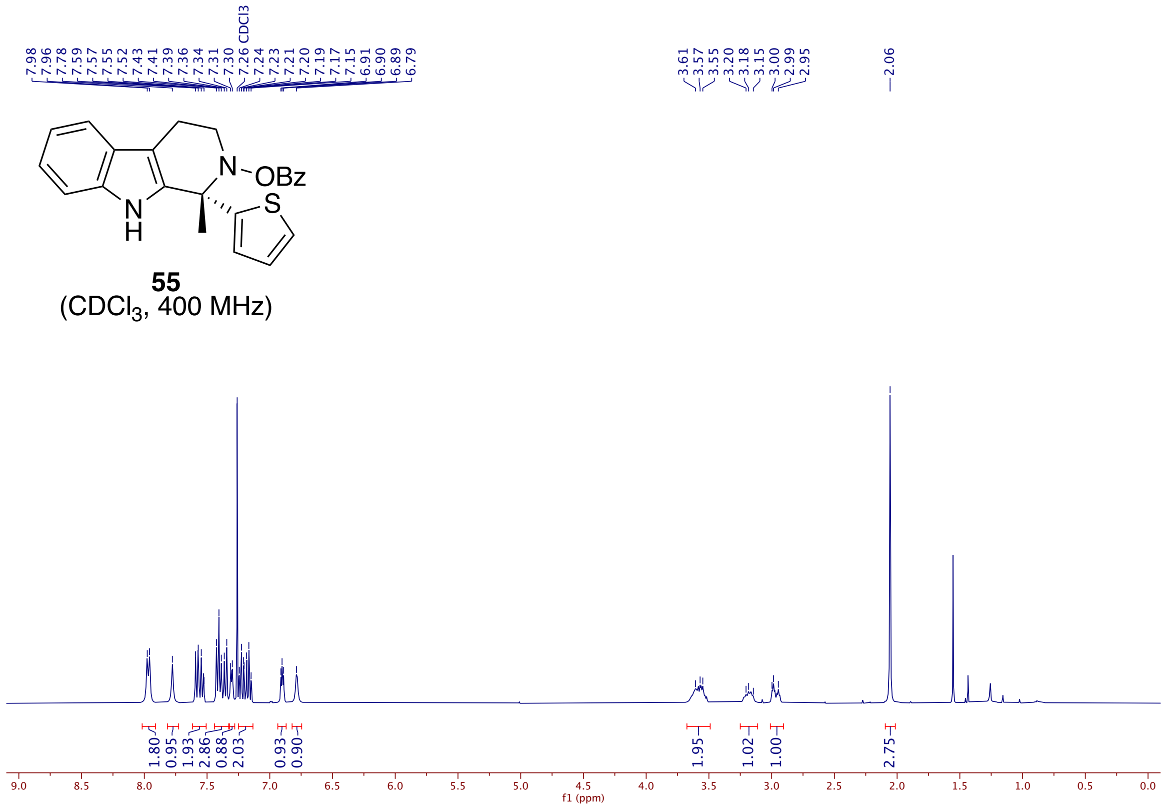
7.98 7.96 7.78 7.59 7.57 7.55 7.52 7.43 7.41 7.39 7.36 7.34 7.31 7.30 7.26 CDCl3 7.24 7.23 7.21 7.20 7.19 7.17 7.15 6.91 6.90 6.89 6.79
3.61 3.57 3.55 3.20 3.18 3.15 3.00 2.99 2.95
2.06
N-OBz
S
N
H
55
(CDCl3, 400 MHz)
1.80 0.95 1.93 2.86 0.88 2.03 0.93 0.90
1.95 1.02 1.00 2.75
9.0 8.5 8.0 7.5 7.0 6.5 6.0 5.5 5.0 4.5 4.0 3.5 3.0 2.5 2.0 1.5 1.0 0.5 0.0
f1 (ppm)

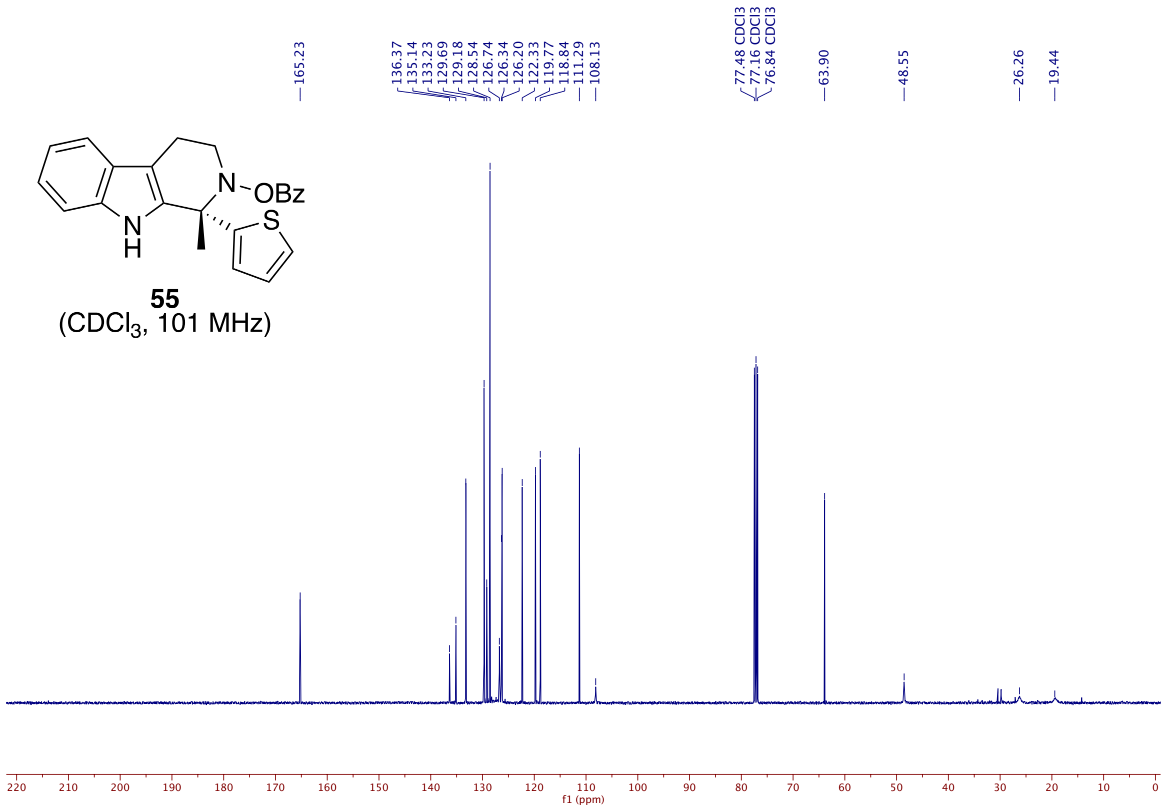
165.23
136.37 135.14 133.23 129.69 129.18 128.54 126.74 126.34 126.20 122.33 119.77 118.84 111.29 108.13
77.48 CDCl3 77.16 CDCl3 76.84 CDCl3
63.90
48.55
26.26
19.44
N-OBz
S
N
H
55
(CDCl3, 101 MHz)
220 210 200 190 180 170 160 150 140 130 120 110 100 90 80 70 60 50 40 30 20 10 0
f1 (ppm)

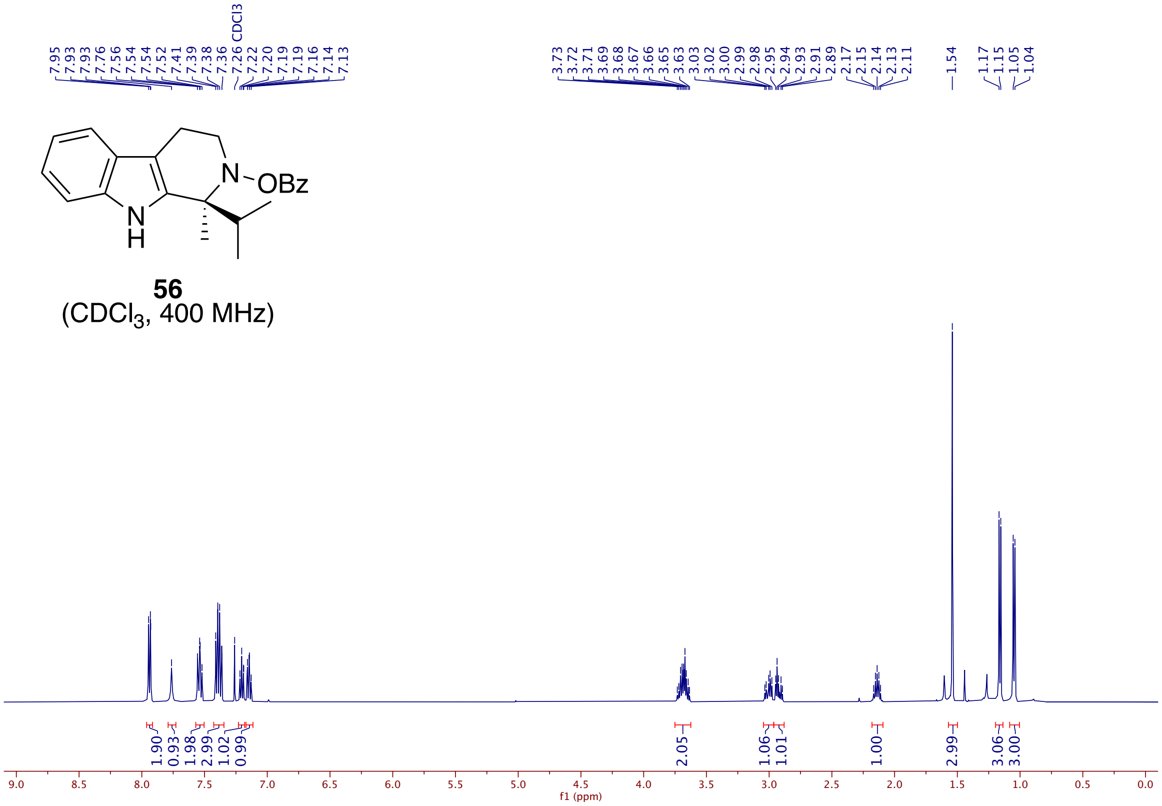
7.95 7.93 7.93 7.76 7.56 7.54 7.54 7.52 7.41 7.39 7.38 7.36 7.26 CDCl3 7.22 7.20 7.19 7.19 7.16 7.14 7.13
3.73 3.72 3.71 3.69 3.68 3.67 3.66 3.65 3.63 3.03 3.02 3.00 2.99 2.98 2.95 2.94 2.93 2.91 2.89 2.17 2.15 2.14 2.13 2.11
1.54
1.17 1.15 1.05 1.04
N
OBz
N
H
56
(CDCl3, 400 MHz)
1.90 0.93 1.98 2.99 1.02 0.99
2.05
1.06 1.01
1.00
2.99
3.06 3.00
9.0 8.5 8.0 7.5 7.0 6.5 6.0 5.5 5.0 4.5 4.0 3.5 3.0 2.5 2.0 1.5 1.0 0.5 0.0
f1 (ppm)

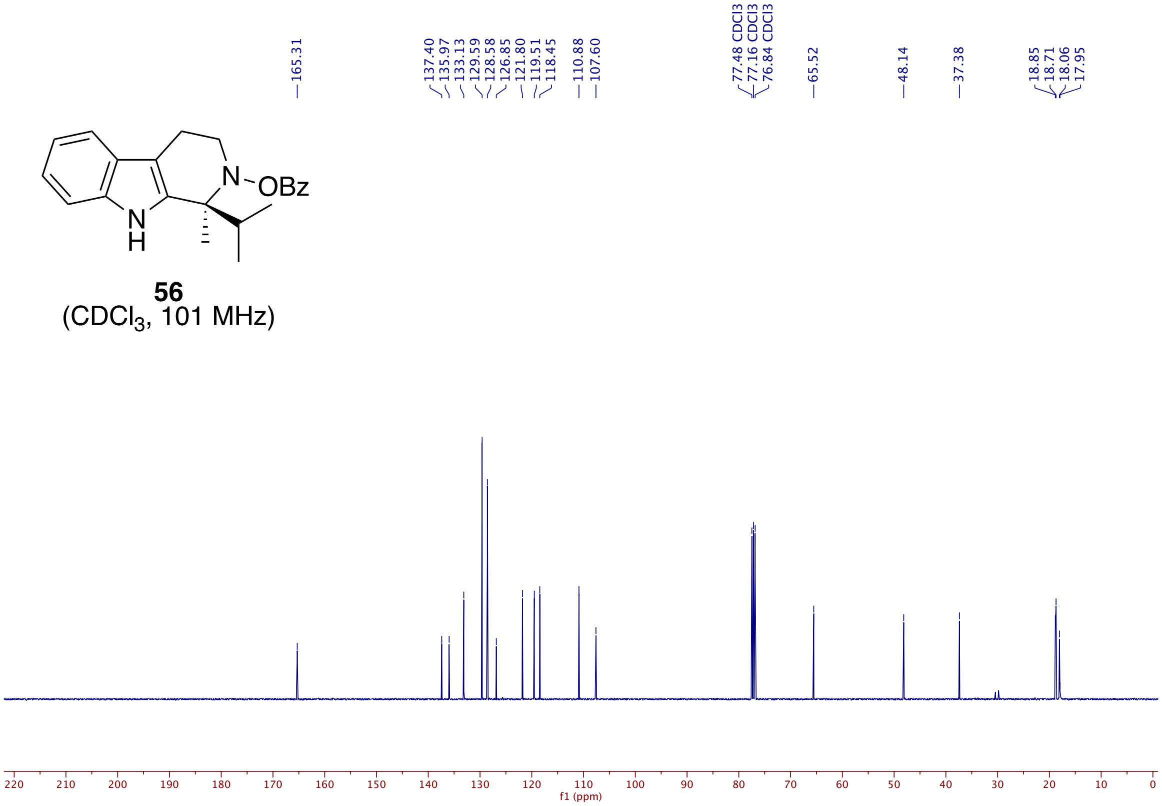
165.31
137.40 135.97 133.13 129.59 128.58 126.85 121.80 119.51 118.45
110.88 107.60
77.48 CDCl3 77.16 CDCl3 76.84 CDCl3
65.52
48.14
37.38
18.85 18.71 18.06 17.95
N
OBz
N
H
56
(CDCl3, 101 MHz)
220 210 200 190 180 170 160 150 140 130 120 110 100 90 80 70 60 50 40 30 20 10 0
f1 (ppm)

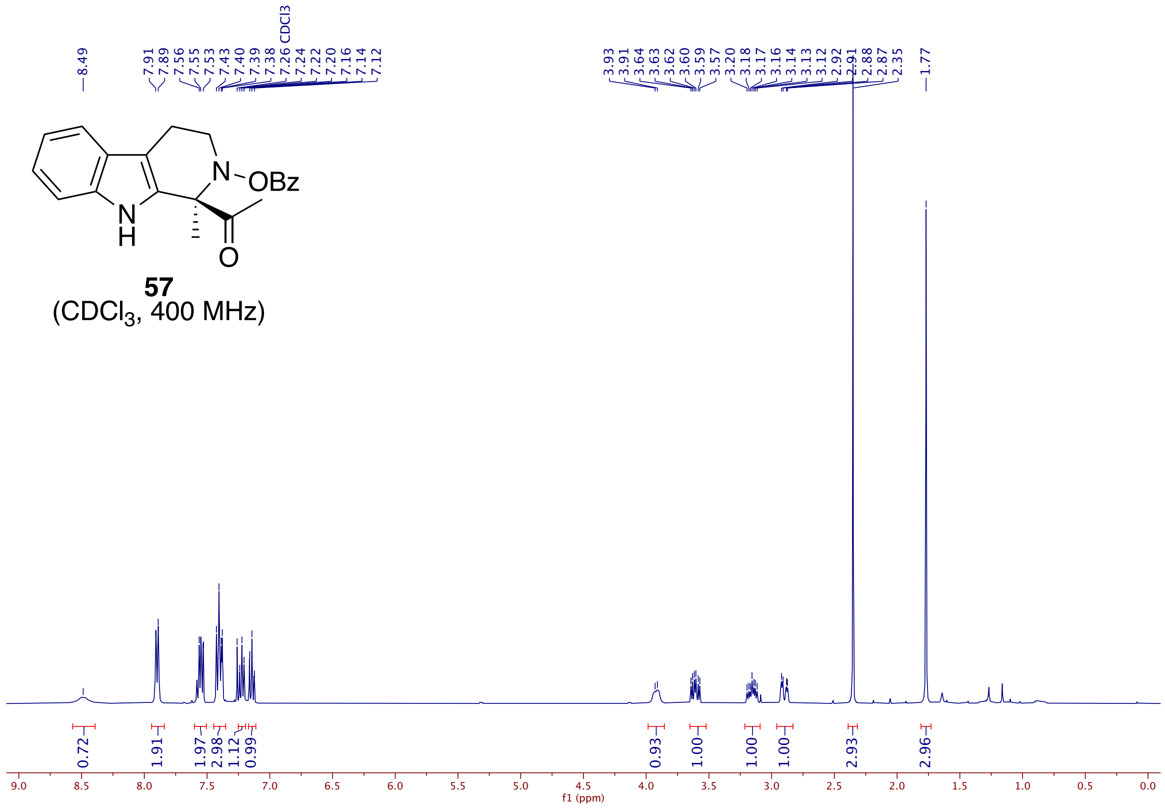

8.49
7.91
7.89
7.56
7.55
7.53
7.43
7.40
7.39
7.38
7.26 CDCl3
7.24
7.22
7.20
7.16
7.14
7.12
3.93
3.91
3.64
3.63
3.62
3.60
3.59
3.57
3.20
3.18
3.17
3.16
3.14
3.13
3.12
2.92
2.91
2.88
2.87
2.35
1.77
N
OBz
N
H
O
57
(CDCl$_3$, 400 MHz)
0.72
1.91
1.97
2.98
1.12
0.99
0.93
1.00
1.00
1.00
2.93
2.96
f1 (ppm)

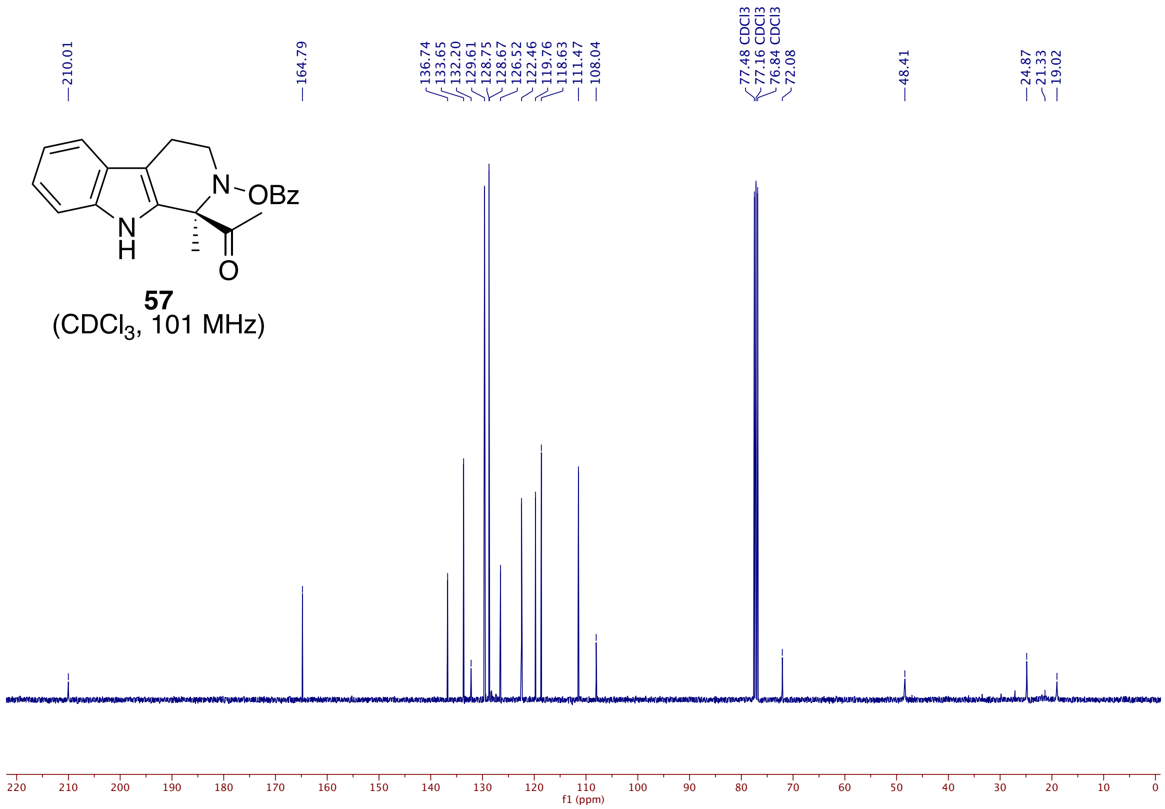

210.01
164.79
136.74
133.65
132.20
129.61
128.75
128.67
126.52
122.46
119.76
118.63
111.47
108.04
77.48 CDCl3
77.16 CDCl3
76.84 CDCl3
72.08
48.41
24.87
21.33
19.02
N
OBz
N
H
O
57
(CDCl$_3$, 101 MHz)
f1 (ppm)

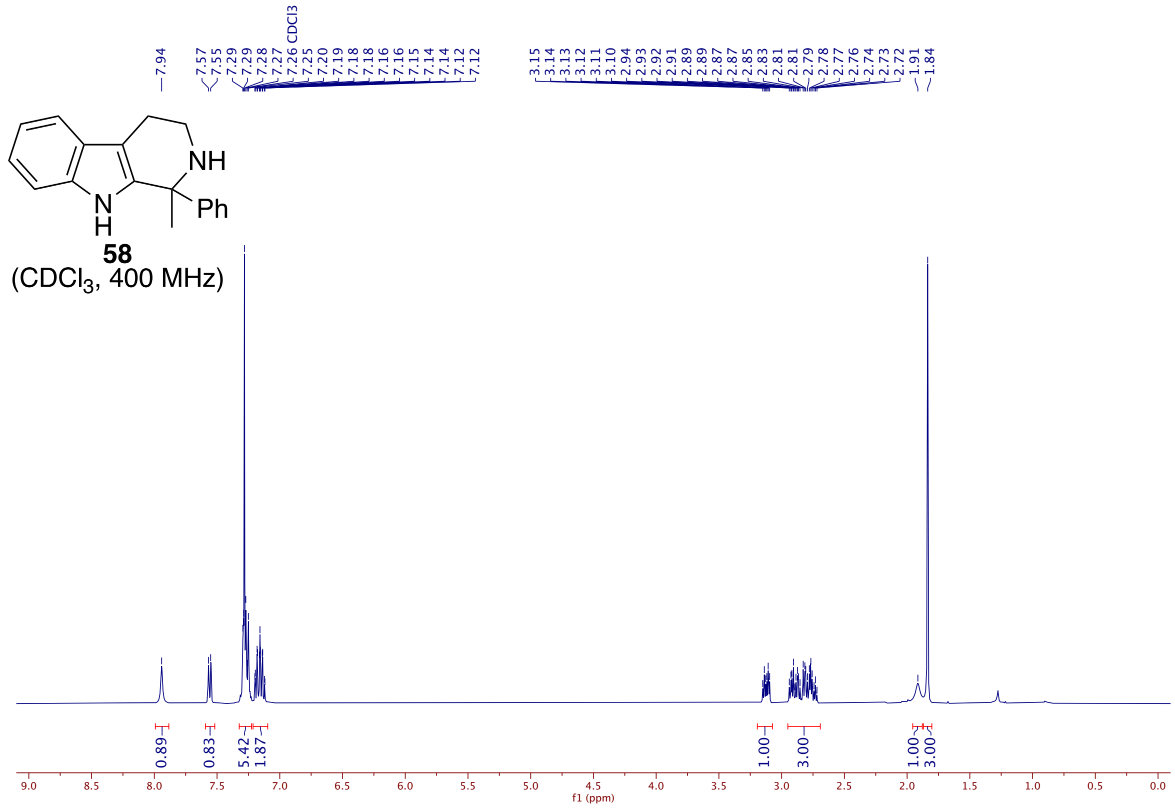
7.94
7.57
7.55
7.29
7.29
7.28
7.27
7.26 CDCl3
7.25
7.20
7.19
7.18
7.18
7.16
7.16
7.15
7.14
7.14
7.12
7.12
3.15
3.14
3.13
3.12
3.11
3.10
2.94
2.93
2.92
2.91
2.89
2.89
2.87
2.87
2.85
2.83
2.81
2.81
2.79
2.78
2.77
2.76
2.74
2.73
2.72
1.91
1.84
NH
N
H
Ph
58
(CDCl3, 400 MHz)
0.89
0.83
5.42
1.87
1.00
3.00
1.00
3.00
f1 (ppm)

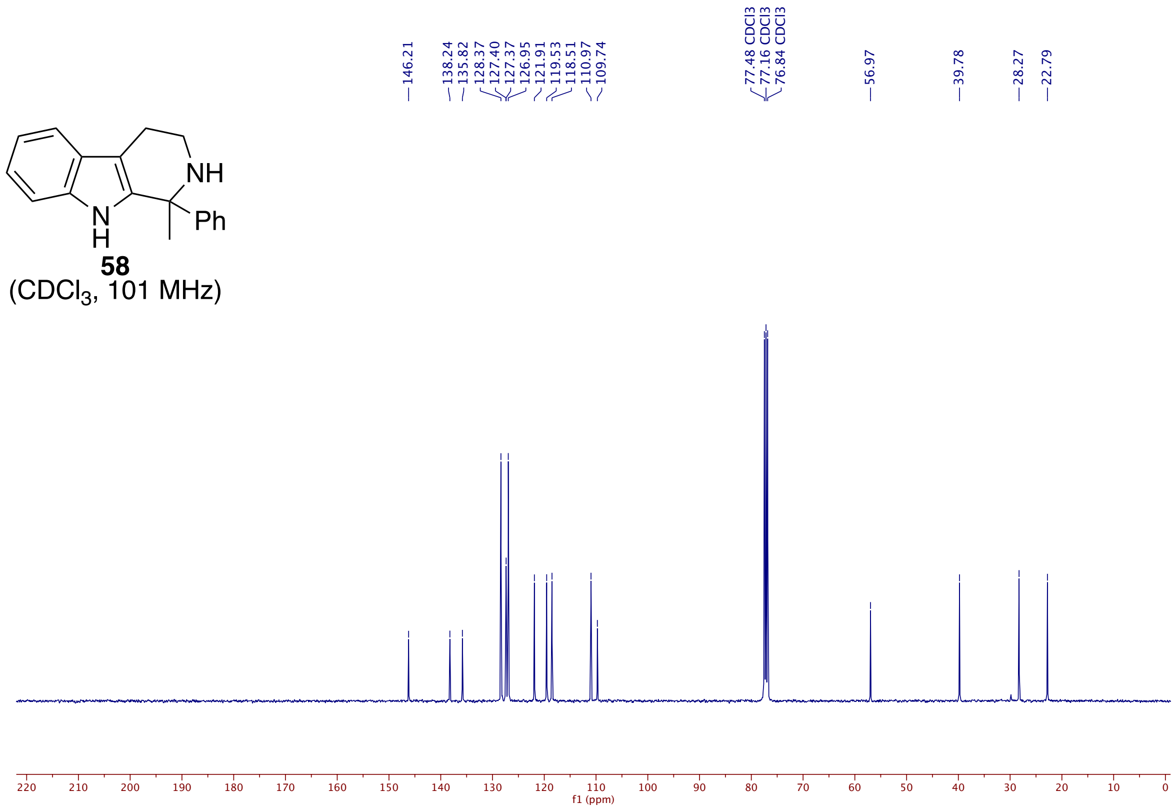
146.21
138.24
135.82
128.37
127.40
127.37
126.95
121.91
119.53
118.51
110.97
109.74
77.48 CDCl3
77.16 CDCl3
76.84 CDCl3
56.97
39.78
28.27
22.79
NH
N
H
Ph
58
(CDCl3, 101 MHz)
f1 (ppm)

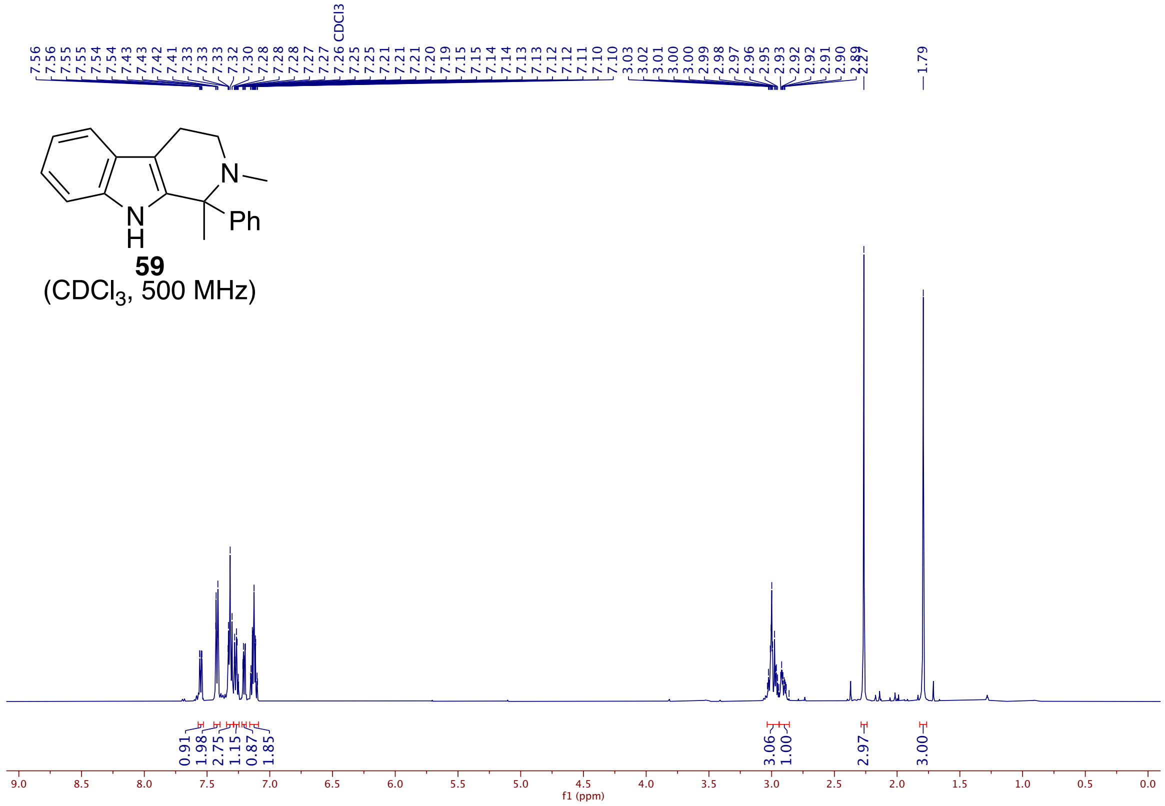

59
($CDCl_3$, 500 MHz)

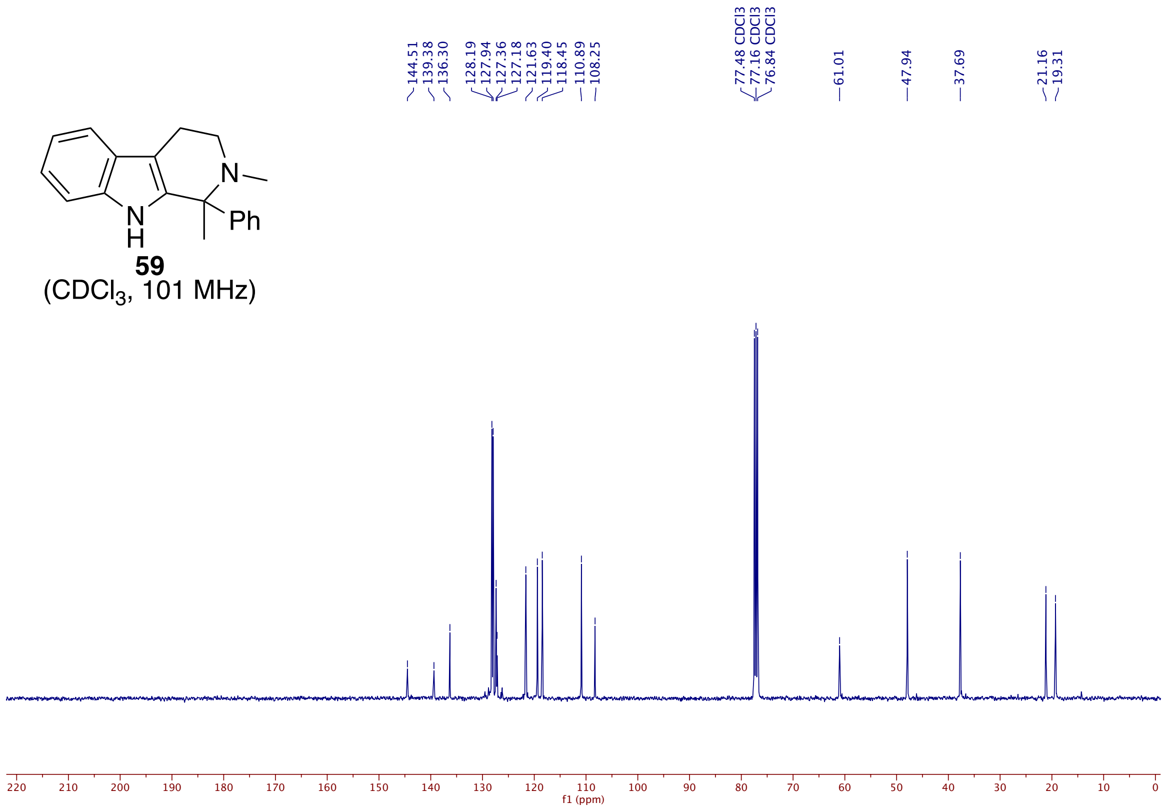

59
($CDCl_3$, 101 MHz)

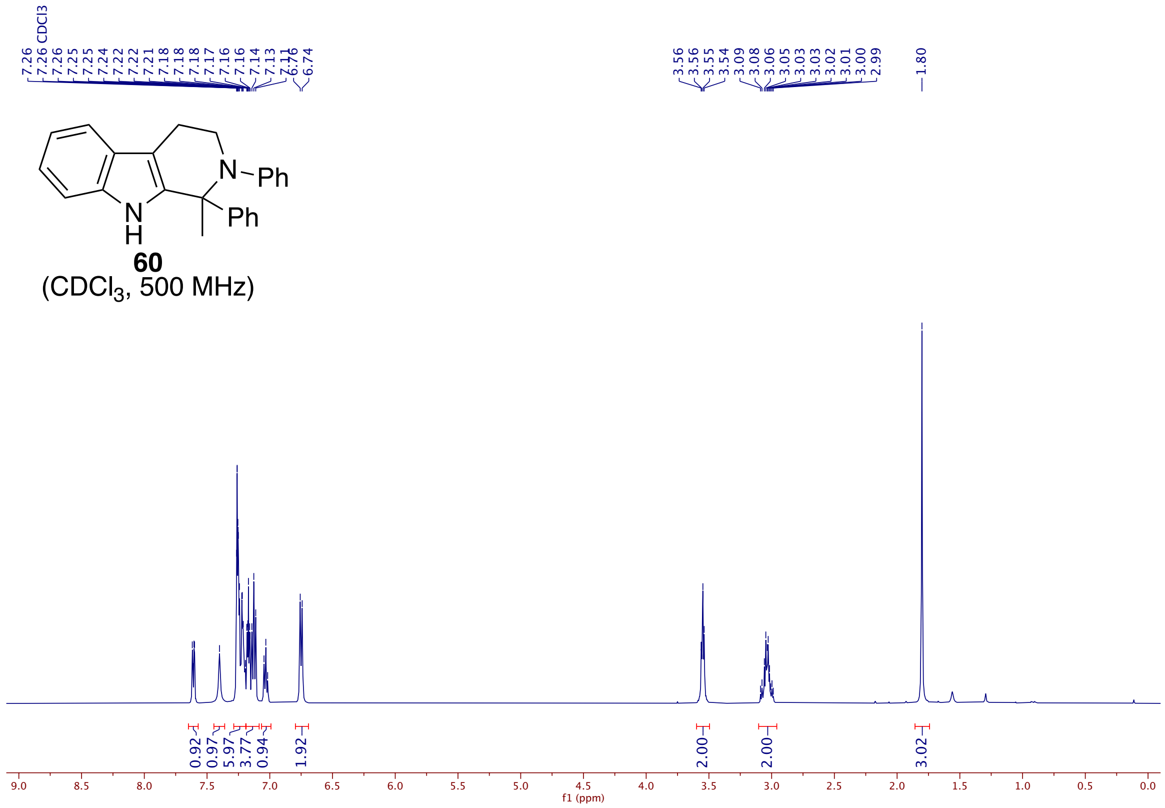
7.26 7.26 CDCl3 7.26 7.25 7.25 7.24 7.22 7.22 7.21 7.18 7.18 7.18 7.17 7.16 7.16 7.14 7.13 7.11 6.76 6.74
3.56 3.56 3.55 3.54 3.09 3.08 3.06 3.05 3.03 3.03 3.02 3.01 3.00 2.99
1.80
N
Ph
N
H
Ph
60
(CDCl3, 500 MHz)
0.92 0.97 5.97 3.77 0.94 1.92 2.00 2.00 3.02
f1 (ppm)

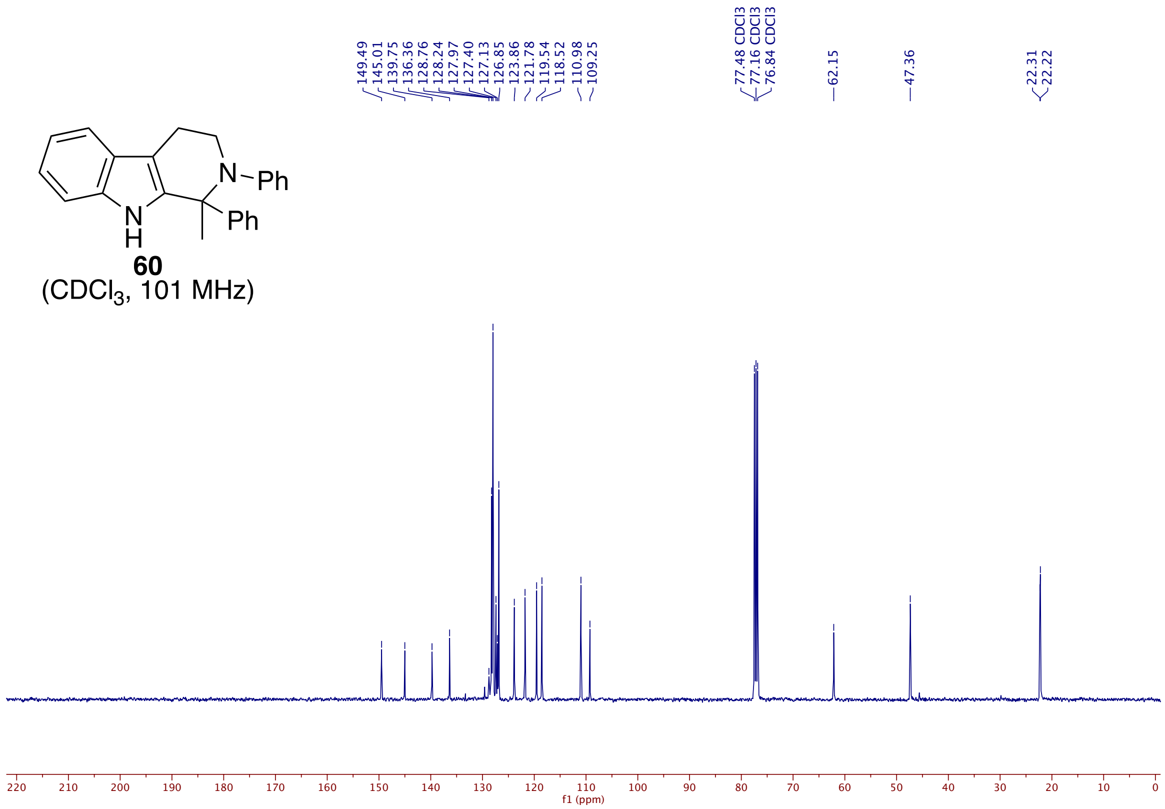
149.49 145.01 139.75 136.36 128.76 128.24 127.97 127.40 127.13 126.85 123.86 121.78 119.54 118.52 110.98 109.25
77.48 CDCl3 77.16 CDCl3 76.84 CDCl3
62.15
47.36
22.31 22.22
N
Ph
N
H
Ph
60
(CDCl3, 101 MHz)
f1 (ppm)

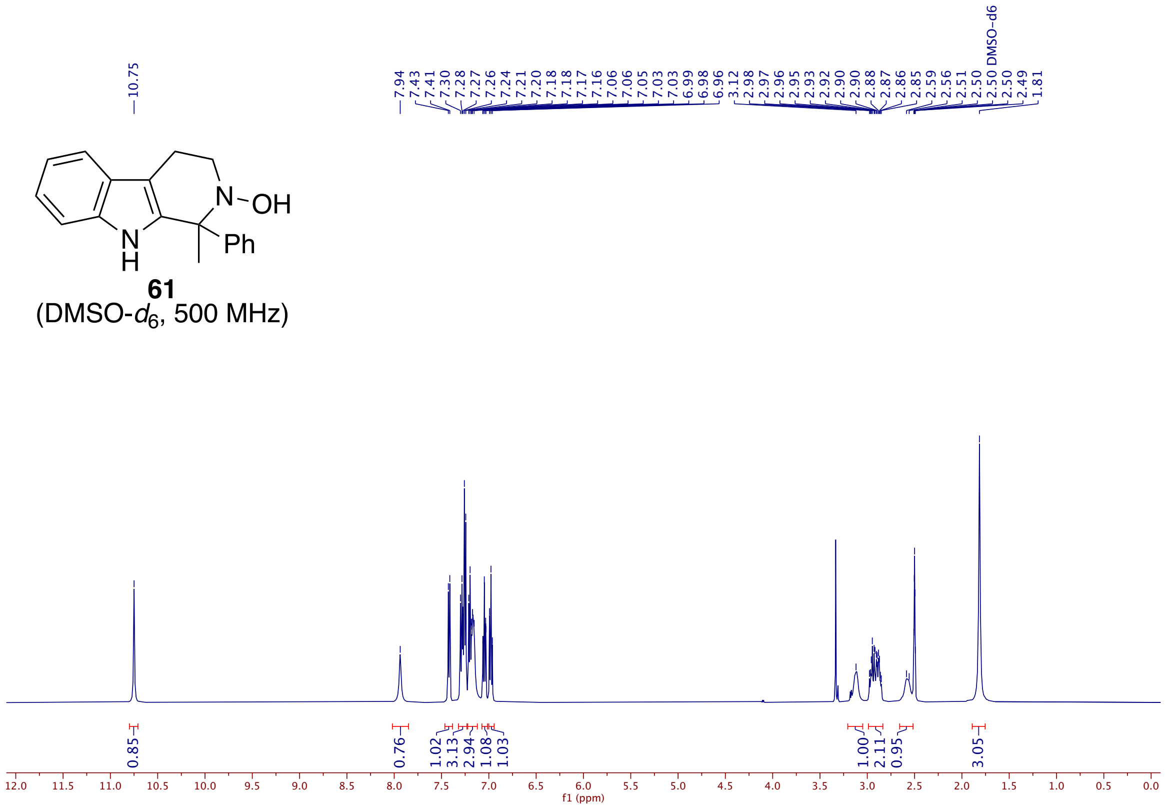

61
(DMSO-d_6, 500 MHz)

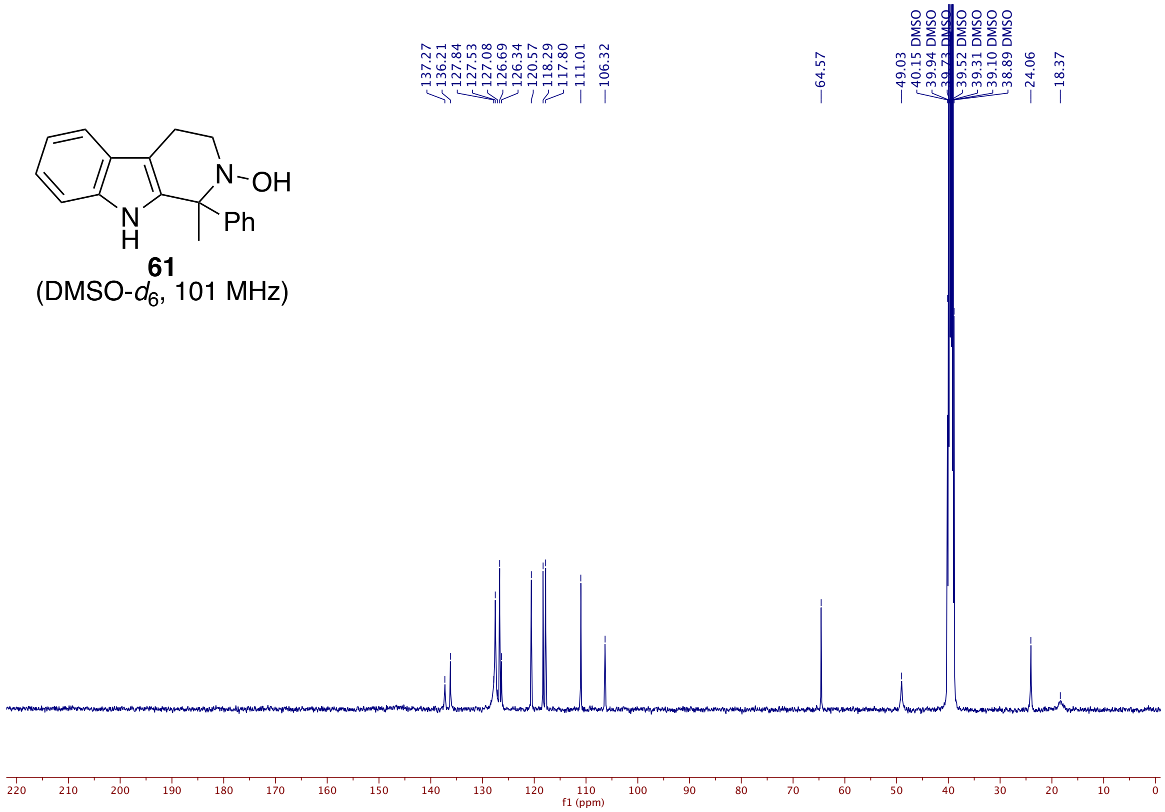

61
(DMSO-d_6, 101 MHz)

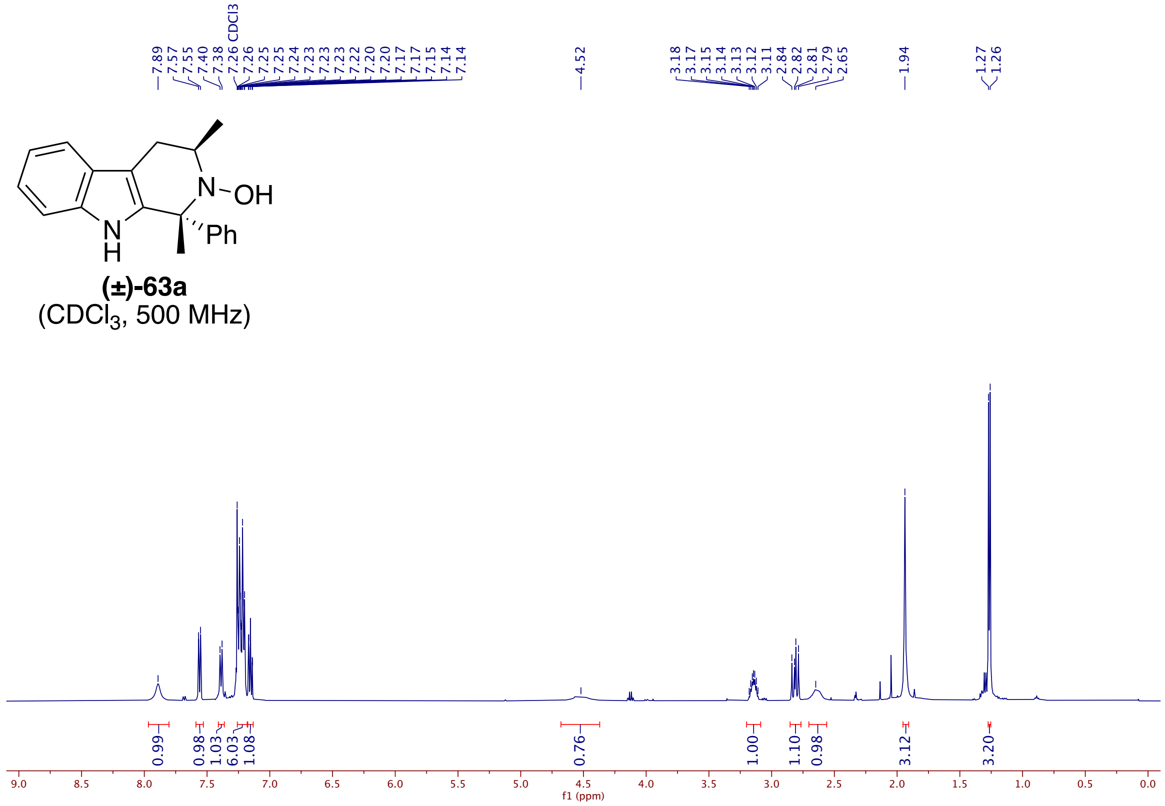

(±)-63a
($CDCl_3$, 500 MHz)

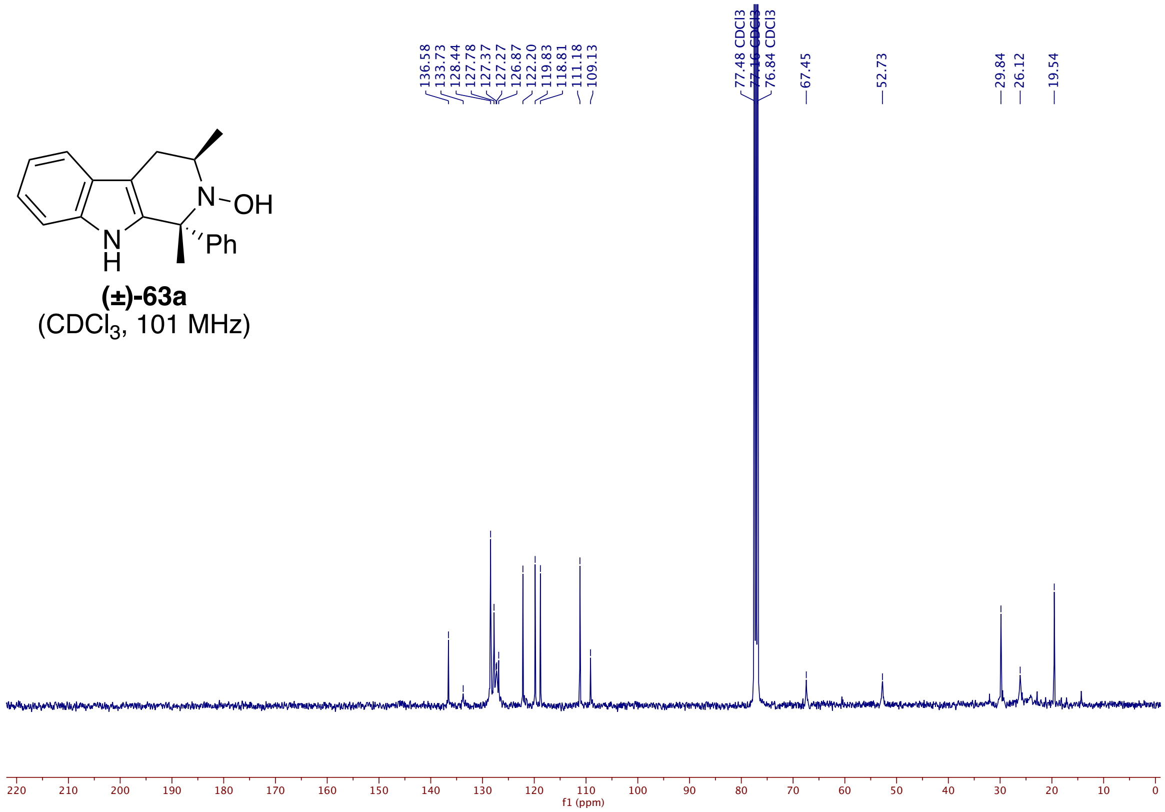

(±)-63a
($CDCl_3$, 101 MHz)

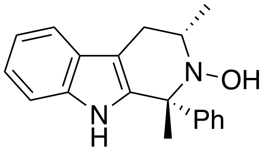

(±)-63b
($CDCl_3$, 500 MHz)

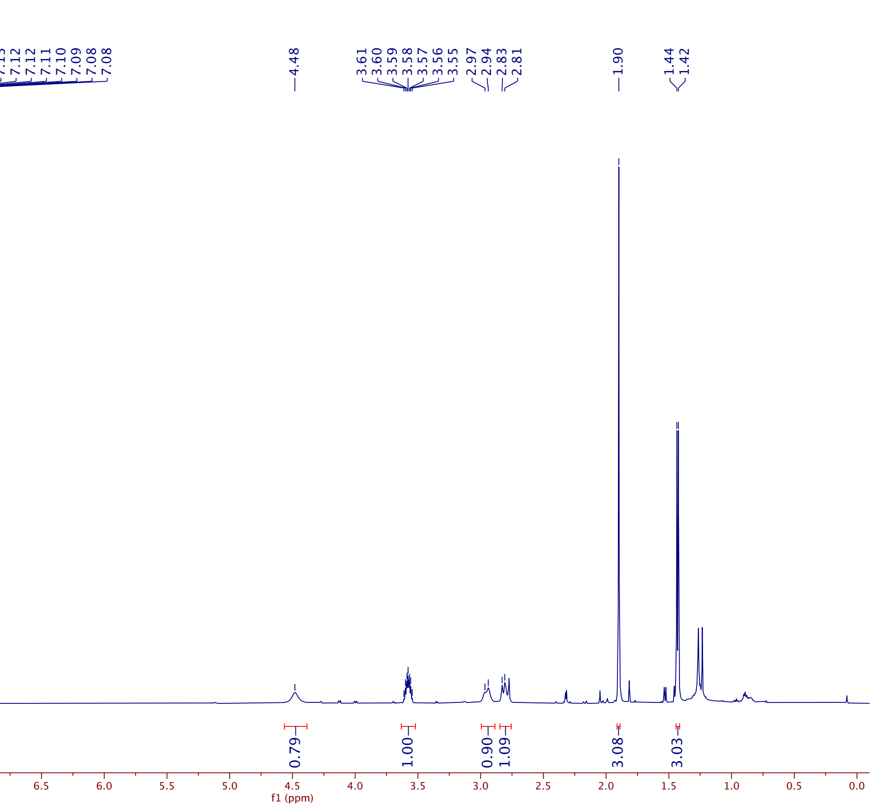

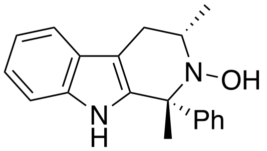

(±)-63b
($CDCl_3$, 101 MHz)

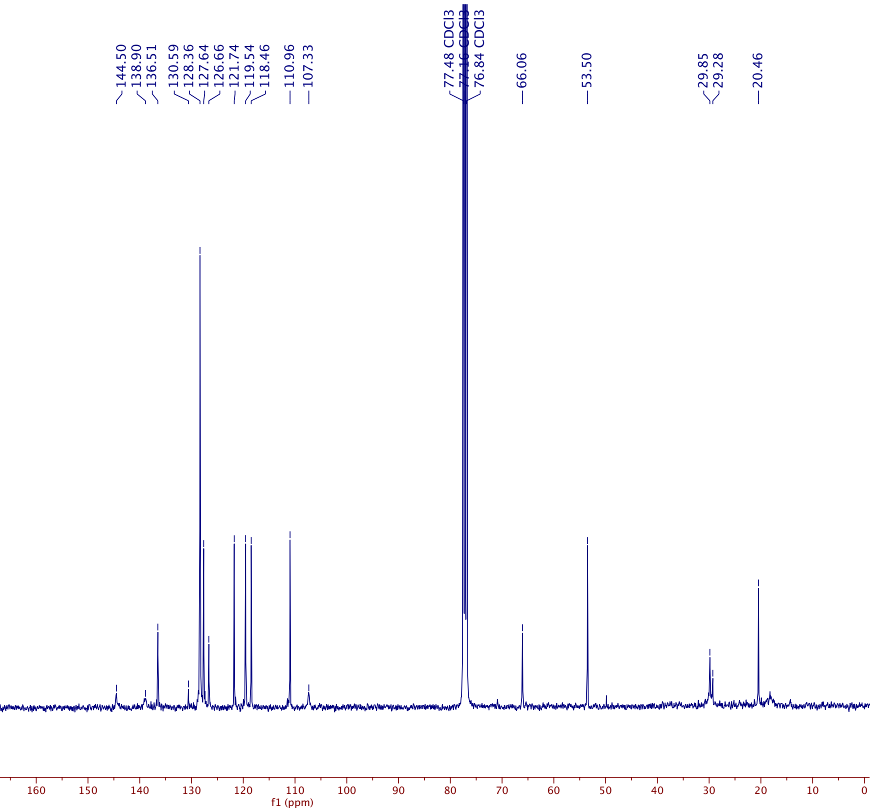

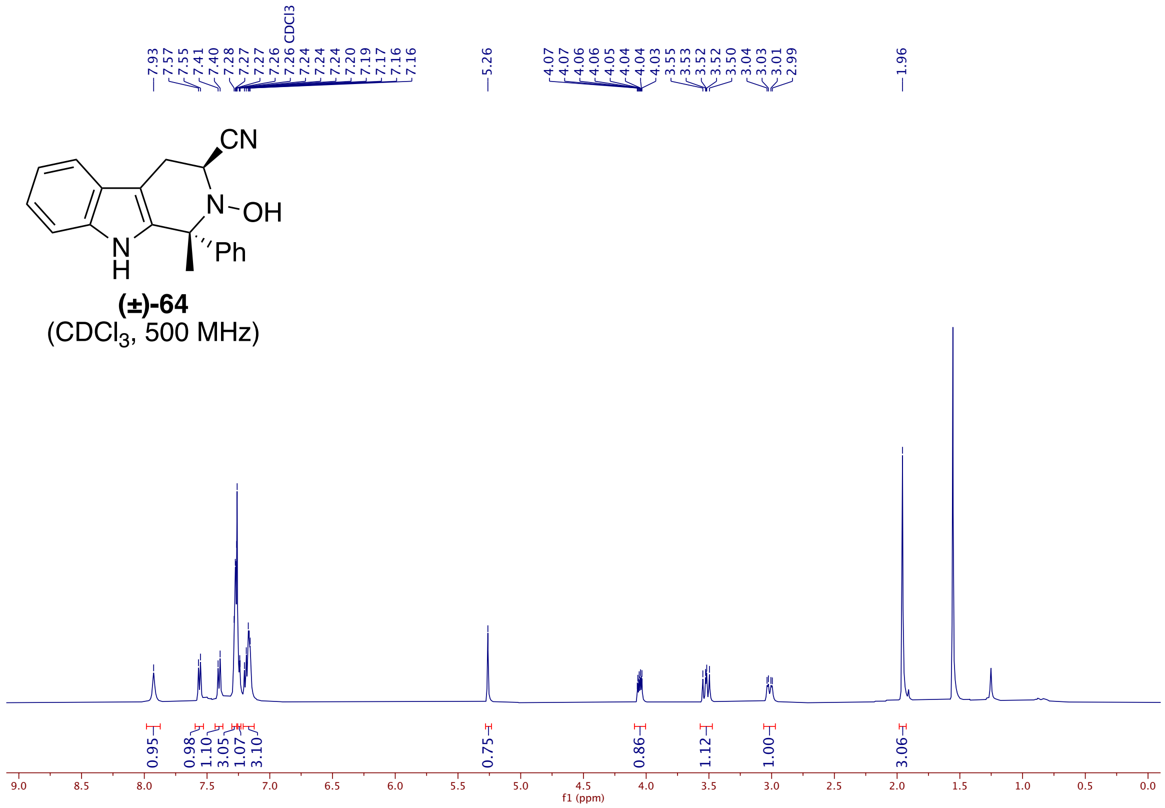
7.93
7.57
7.55
7.41
7.40
7.28
7.27
7.27
7.26 CDCl3
7.26
7.24
7.24
7.24
7.20
7.19
7.17
7.16
7.16
5.26
4.07
4.07
4.06
4.06
4.05
4.04
4.04
4.03
3.55
3.53
3.52
3.52
3.50
3.04
3.03
3.01
2.99
1.96
CN
N
OH
N
H
Ph
(±)-64
(CDCl3, 500 MHz)
0.95
0.98
1.10
3.05
1.07
3.10
0.75
0.86
1.12
1.00
3.06
f1 (ppm)

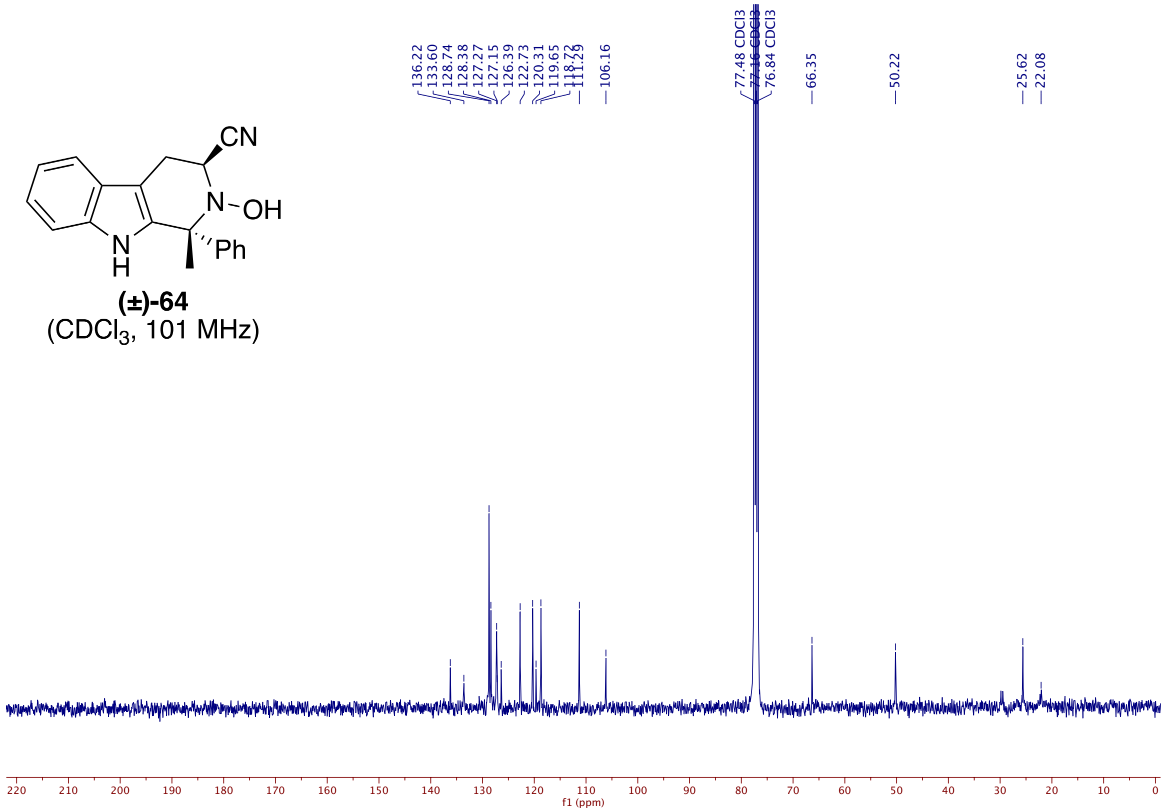
136.22
133.60
128.74
128.38
127.27
127.15
126.39
122.73
120.31
119.65
106.16
77.48 CDCl3
76.84 CDCl3
66.35
50.22
25.62
22.08
CN
N
OH
N
H
Ph
(±)-64
(CDCl3, 101 MHz)
f1 (ppm)

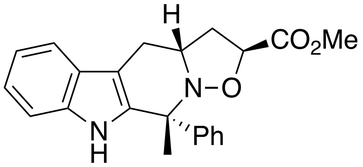

(±)-65a
($CDCl_3$, 500 MHz)

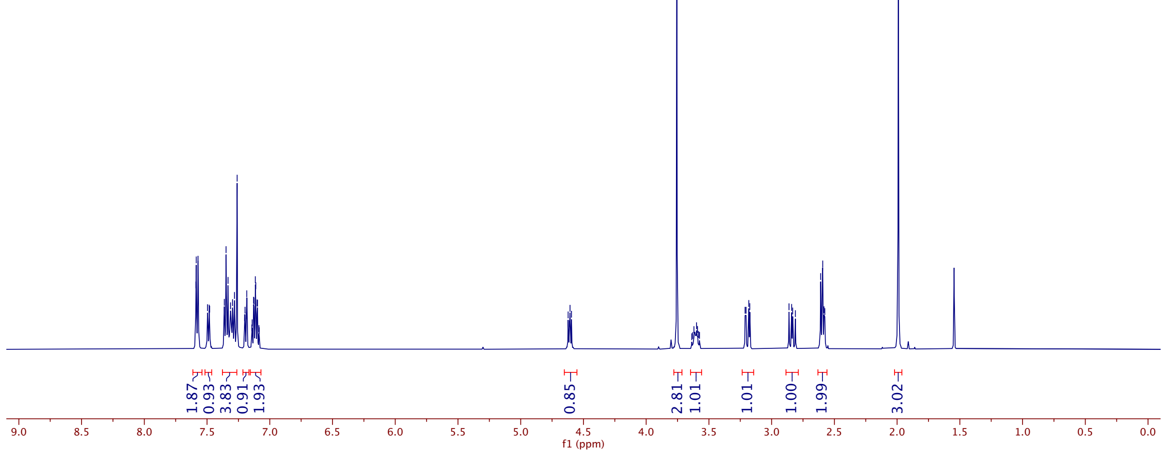

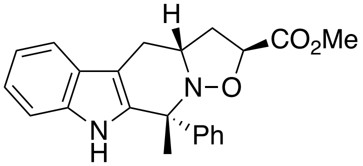

(±)-65a
($CDCl_3$, 101 MHz)

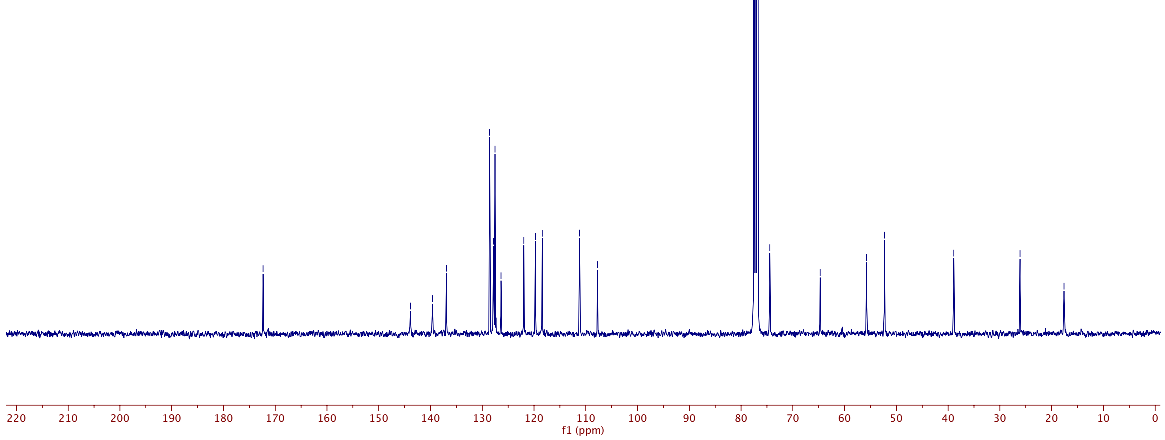

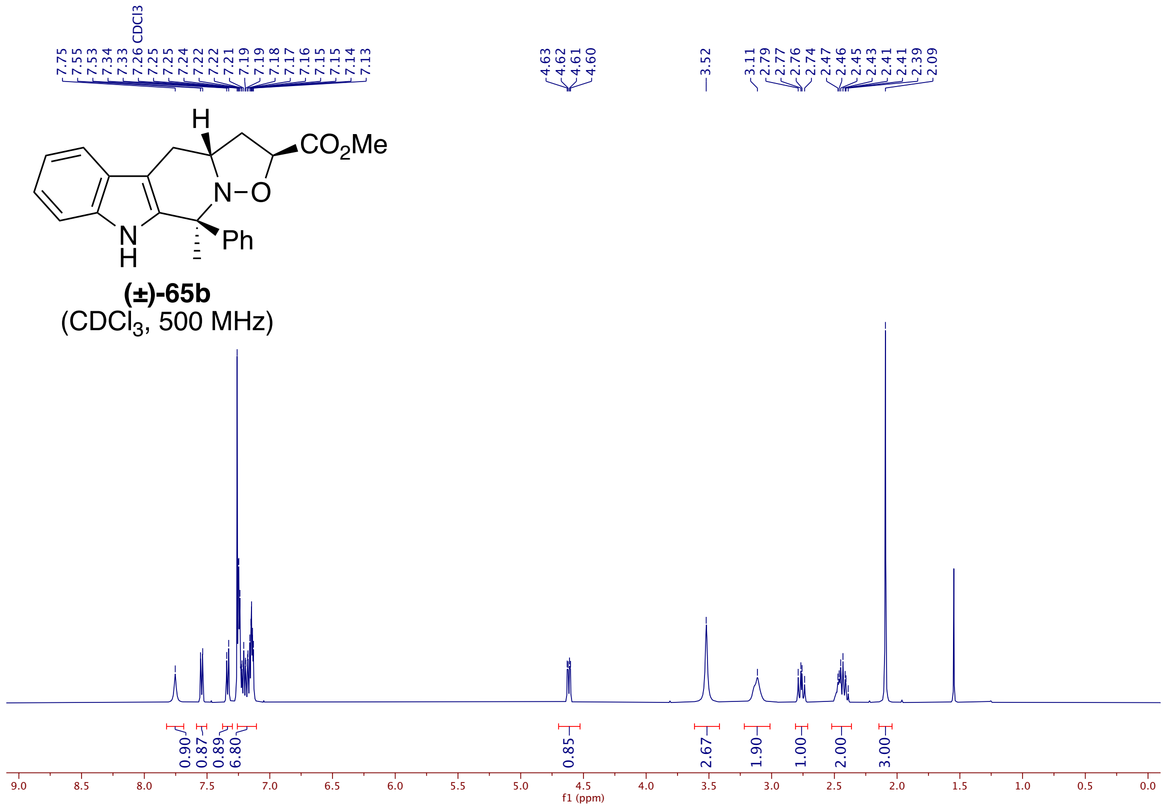

7.75 7.55 7.53 7.34 7.33 7.26 CDCl3 7.25 7.25 7.24 7.22 7.22 7.21 7.19 7.19 7.18 7.17 7.16 7.15 7.15 7.14 7.13
4.63 4.62 4.61 4.60
3.52
3.11 2.79 2.77 2.76 2.74 2.47 2.46 2.45 2.43 2.41 2.41 2.39 2.09
H
CO2Me
N–O
N
H
Ph
(±)-65b
(CDCl3, 500 MHz)
0.90 0.87 0.89 6.80 0.85 2.67 1.90 1.00 2.00 3.00
f1 (ppm)

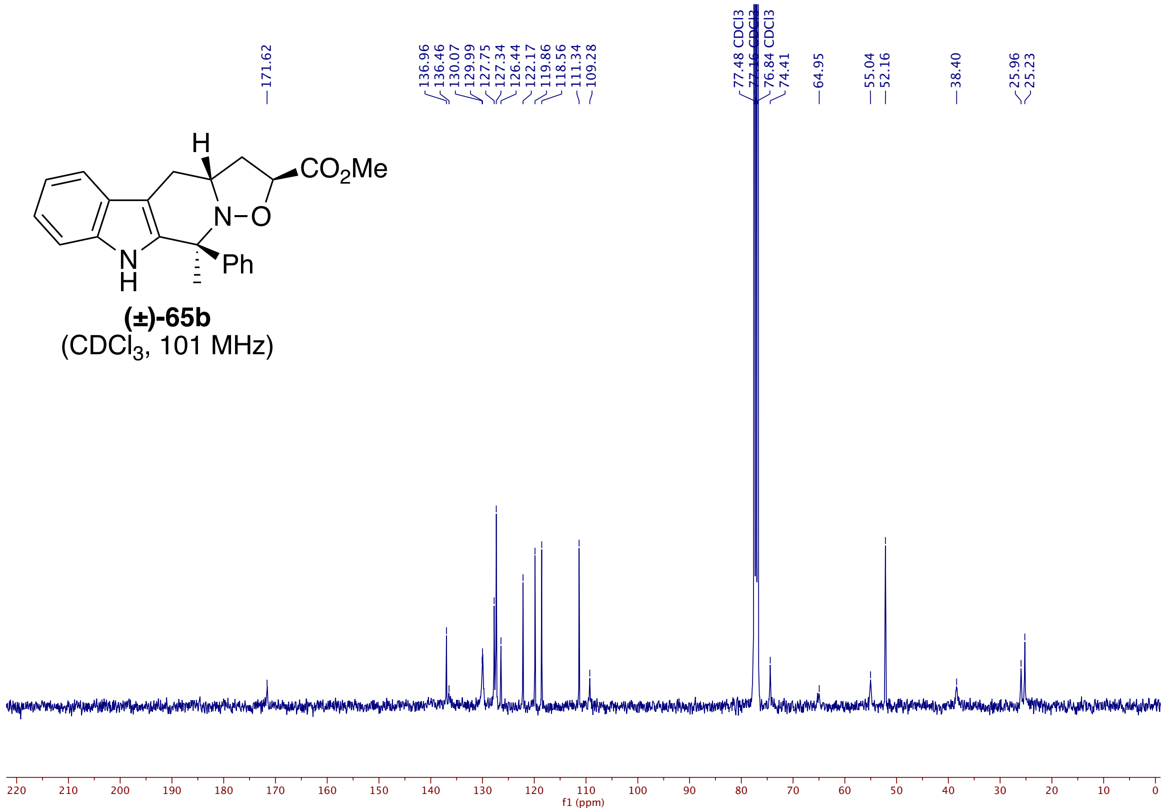

171.62
136.96 136.46 130.07 129.99 127.75 127.34 126.44 122.17 119.86 118.56 111.34 109.28
77.48 CDCl3 76.84 CDCl3 74.41
64.95
55.04 52.16
38.40
25.96 25.23
H
CO2Me
N–O
N
H
Ph
(±)-65b
(CDCl3, 101 MHz)
f1 (ppm)

M. HPLC Traces

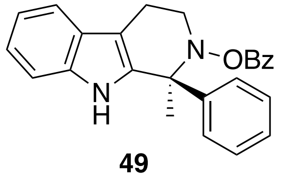

49

Conditions: HPLC (ChiralPak AD-H, 90:10 hexanes/i-PrOH, 1 mL/min, 254 nm)

Racemic Sample:

<Chromatogram>

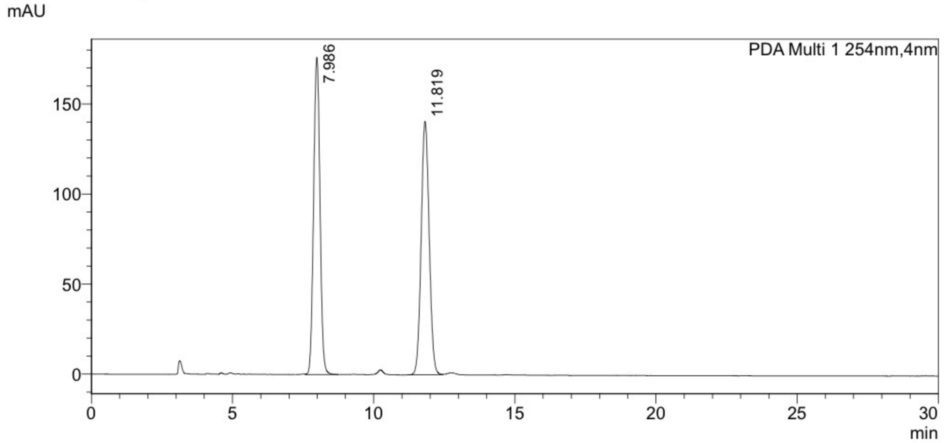

<Peak Table>

PDA Ch1 254nm

Peak#	Ret. Time	Height	Area	Height%	Area%
1	7.986	176267	2749666	55.582	49.673
2	11.819	140861	2785819	44.418	50.327
Total		317128	5535485	100.000	100.000

Enantioenriched Sample:

<Chromatogram>

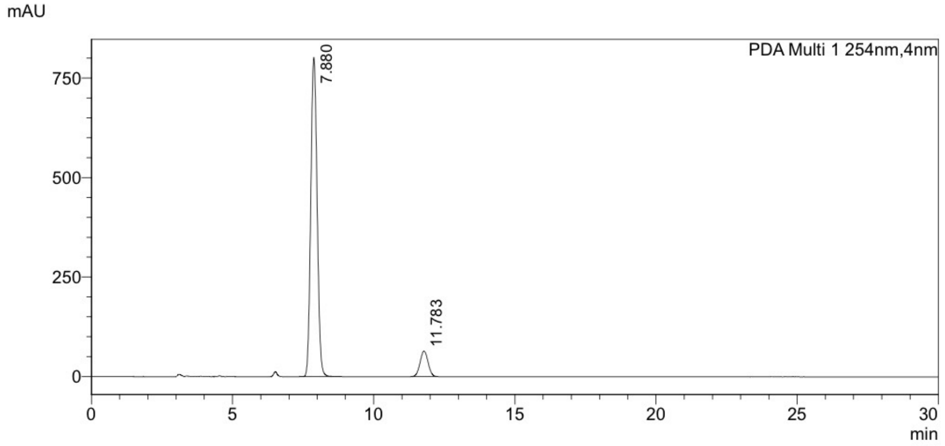

<Peak Table>

PDA Ch1 254nm

Peak#	Ret. Time	Height	Area	Height%	Area%
1	7.880	802274	12518070	92.580	90.783
2	11.783	64304	1270991	7.420	9.217
Total		866578	13789061	100.000	100.000

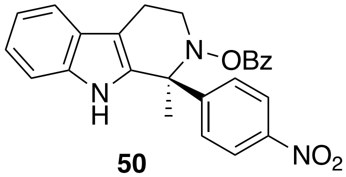

50

Conditions: HPLC (ChiralPak AD-H, 90:10 hexanes/i-PrOH, 1 mL/min, 254 nm)

Racemic Sample:

<Chromatogram>

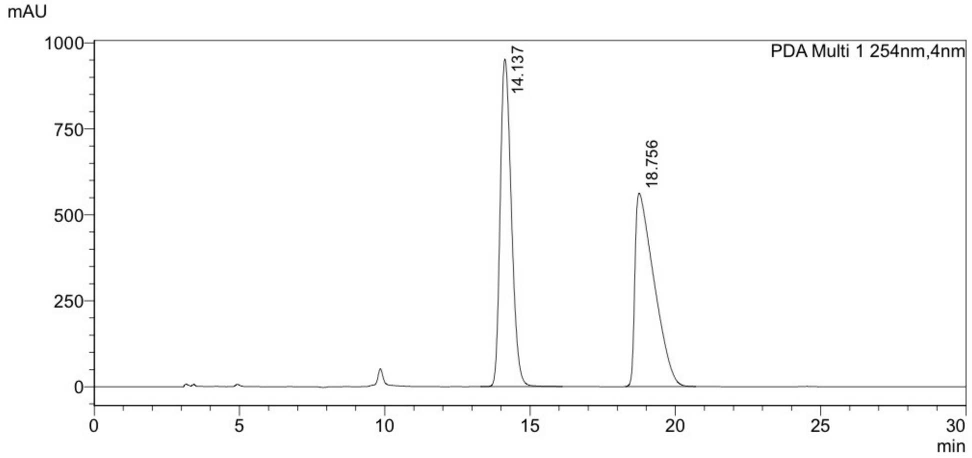

<Peak Table>

PDA Ch1 254nm

Peak#	Ret. Time	Height	Area	Height%	Area%
1	14.137	952894	25421779	62.900	49.773
2	18.756	562034	25654098	37.100	50.227
Total		1514928	51075878	100.000	100.000

Enantioenriched Sample:

<Chromatogram>

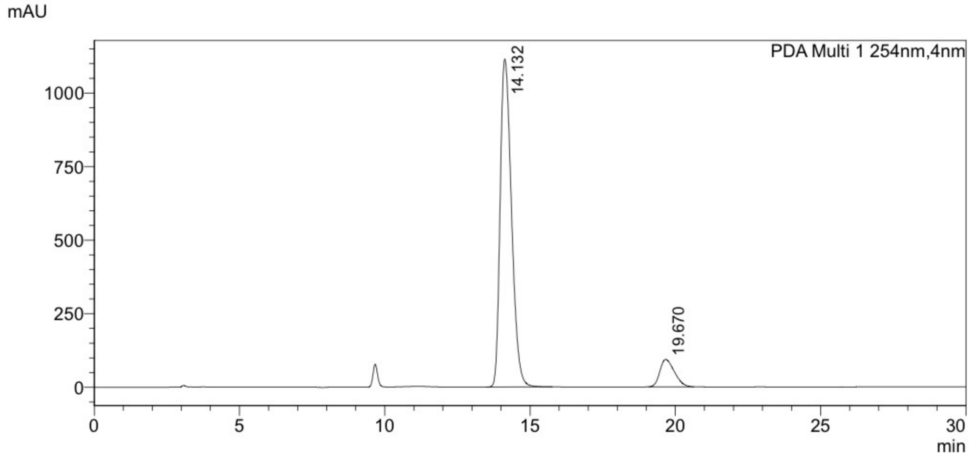

<Peak Table>

PDA Ch1 254nm

Peak#	Ret. Time	Height	Area	Height%	Area%
1	14.132	1115363	30219406	92.306	90.376
2	19.670	92969	3218078	7.694	9.624
Total		1208332	33437484	100.000	100.000

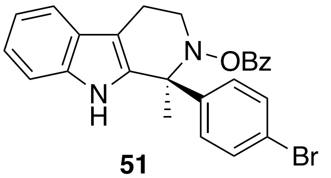

51

Conditions: HPLC (ChiralPak AD-H, 90:10 hexanes/i-PrOH, 1 mL/min, 254 nm)

Racemic Sample:

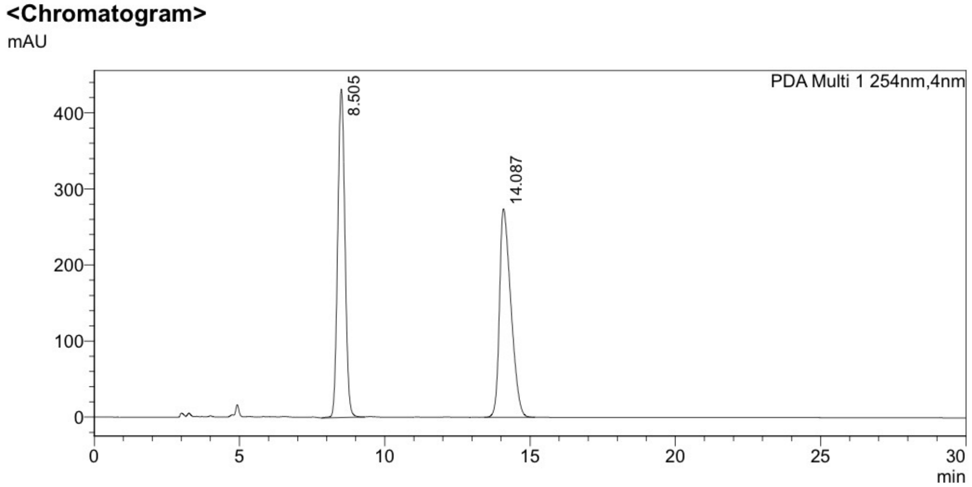

<Peak Table>

PDA Ch1 254nm

Peak#	Ret. Time	Height	Area	Height%	Area%
1	8.505	431813	7476611	61.157	50.000
2	14.087	274260	7476740	38.843	50.000
Total		706073	14953351	100.000	100.000

Enantioenriched Sample:

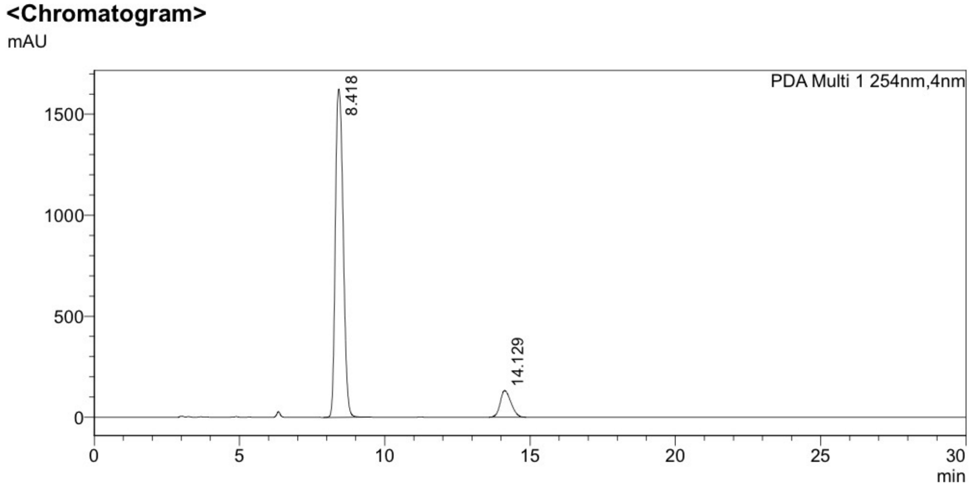

<Peak Table>

PDA Ch1 254nm

Peak#	Ret. Time	Height	Area	Height%	Area%
1	8.418	1625324	30373382	92.499	89.951
2	14.129	131804	3393250	7.501	10.049
Total		1757128	33766632	100.000	100.000

Crystallization Sample:

<Chromatogram>

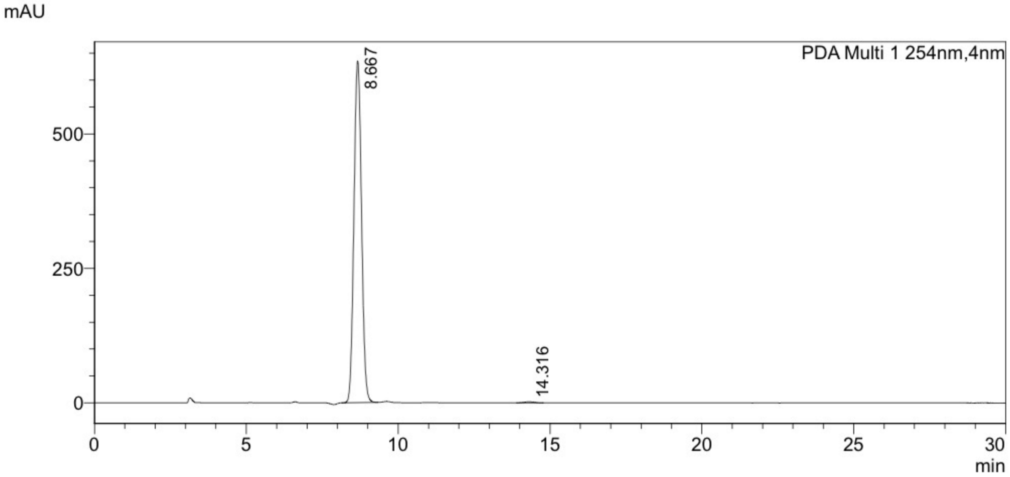

<Peak Table>

PDA Ch1 254nm

Peak#	Ret. Time	Height	Area	Height%	Area%
1	8.667	635186	10994403	99.703	99.590
2	14.316	1893	45287	0.297	0.410
Total		637078	11039691	100.000	100.000

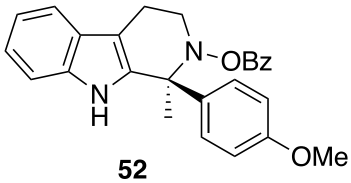

52

Conditions: HPLC (ChiralPak AD-H, 90:10 hexanes/i-PrOH, 1 mL/min, 254 nm)

Racemic Sample:

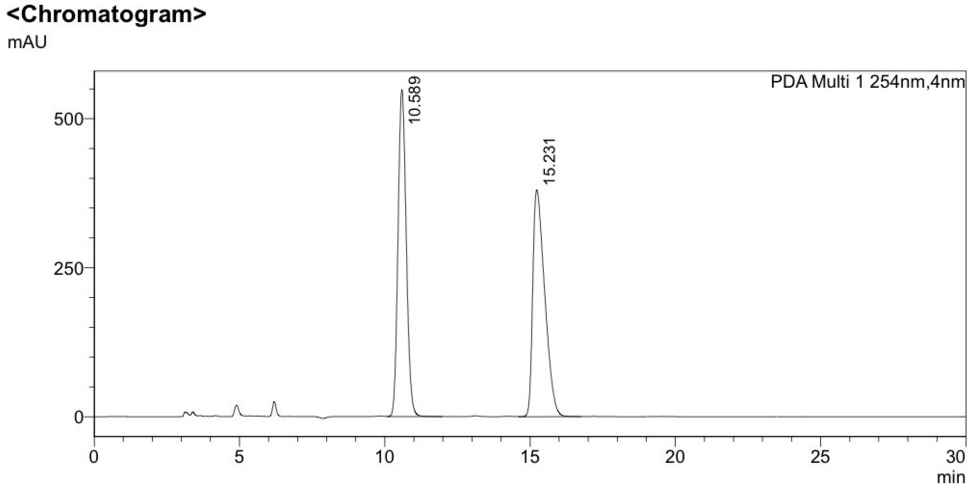

<Peak Table>

PDA Ch1 254nm

Peak#	Ret. Time	Height	Area	Height%	Area%
1	10.589	548849	10821368	59.038	49.840
2	15.231	380801	10890847	40.962	50.160
Total		929651	21712215	100.000	100.000

Enantioenriched Sample:

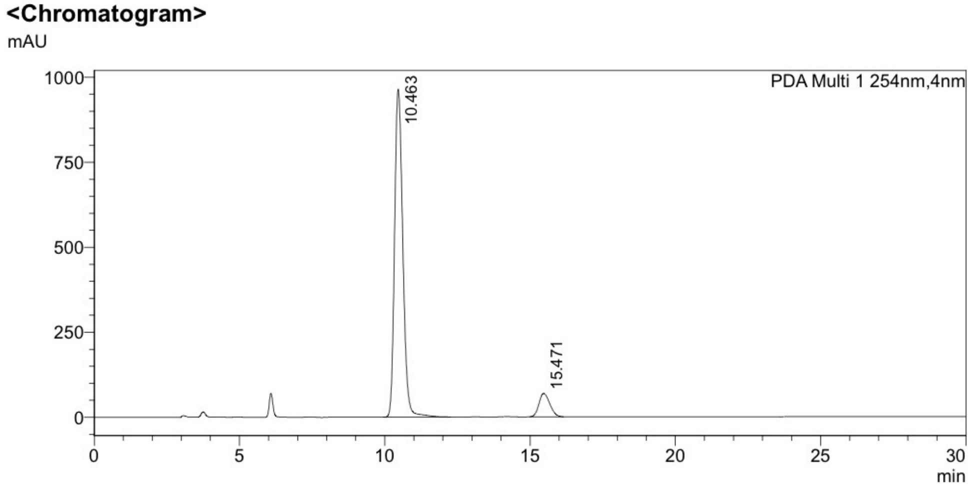

<Peak Table>

PDA Ch1 254nm

Peak#	Ret. Time	Height	Area	Height%	Area%
1	10.463	964866	19025086	93.357	91.455
2	15.471	68658	1777665	6.643	8.545
Total		1033524	20802751	100.000	100.000

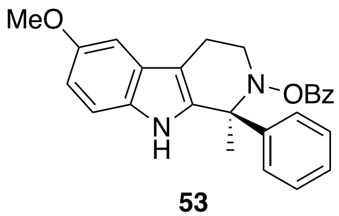

53

Conditions: HPLC (ChiralPak AD-H, 90:10 hexanes/i-PrOH, 1 mL/min, 254 nm)

Racemic Sample:

<Chromatogram>

mAU

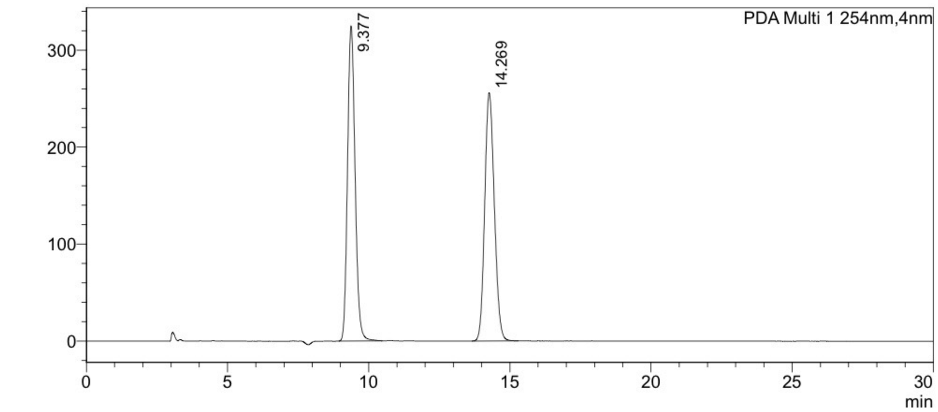

<Peak Table>

PDA Ch1 254nm

Peak#	Ret. Time	Height	Area	Height%	Area%
1	9.377	325055	6031388	55.957	49.822
2	14.269	255845	6074544	44.043	50.178
Total		580900	12105932	100.000	100.000

Enantioenriched Sample:

<Chromatogram>

mAU

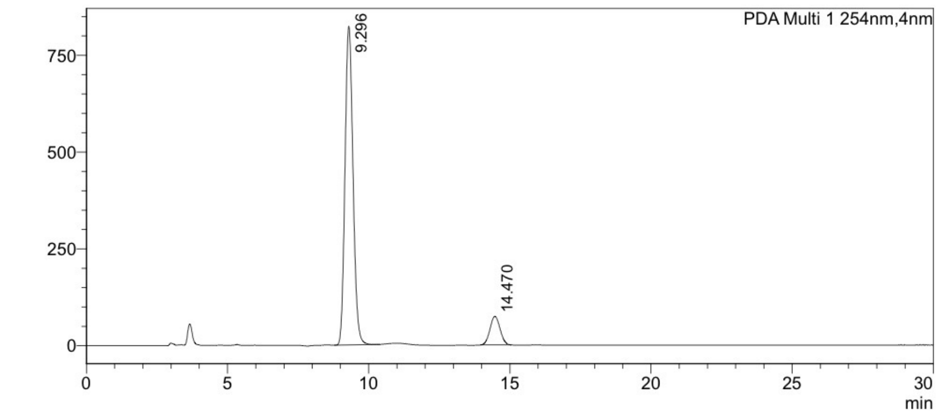

<Peak Table>

PDA Ch1 254nm

Peak#	Ret. Time	Height	Area	Height%	Area%
1	9.296	822941	16240677	91.771	89.871
2	14.470	73796	1830399	8.229	10.129
Total		896737	18071077	100.000	100.000

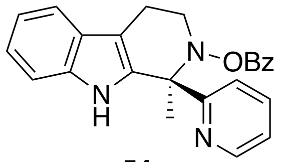

54

Conditions: HPLC (ChiralPak AD-H, 90:10 hexanes/i-PrOH, 1 mL/min, 254 nm)

Racemic Sample:

<Chromatogram>

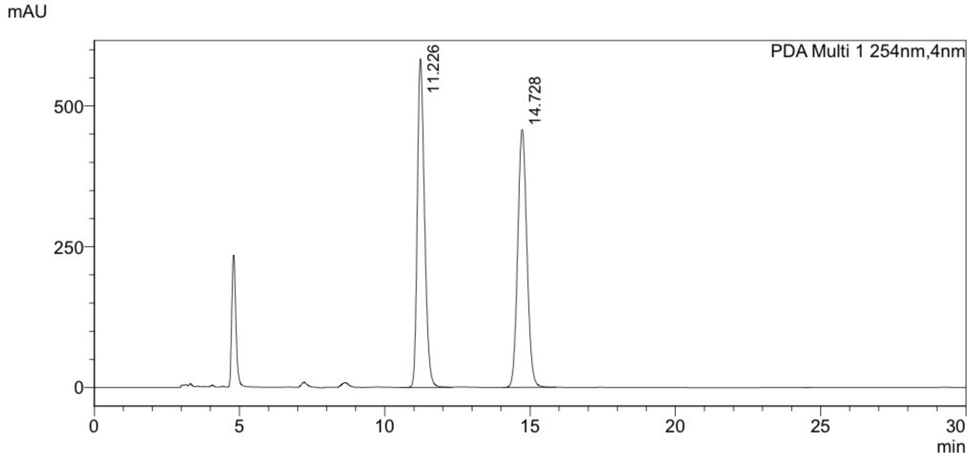

<Peak Table>

PDA Ch1 254nm

Peak#	Ret. Time	Height	Area	Height%	Area%
1	11.226	583381	10071262	55.997	49.998
2	14.728	458435	10072209	44.003	50.002
Total		1041816	20143471	100.000	100.000

Enantioenriched Sample:

<Chromatogram>

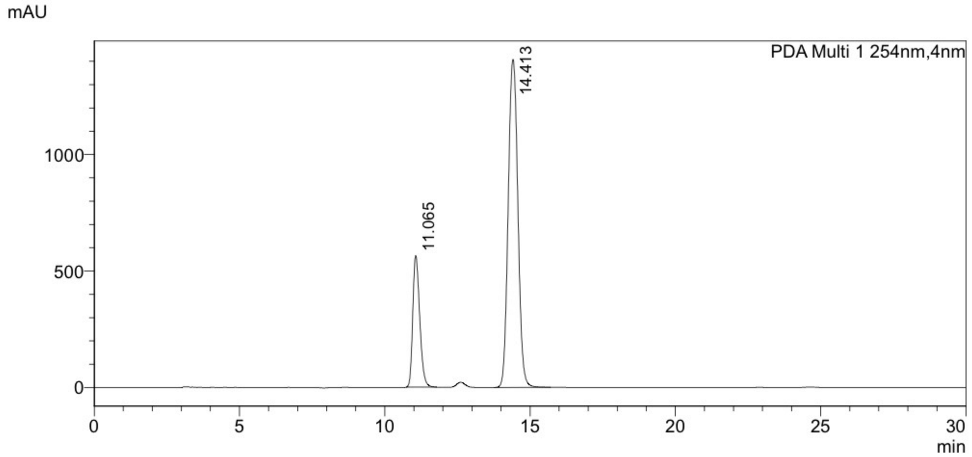

<Peak Table>

PDA Ch1 254nm

Peak#	Ret. Time	Height	Area	Height%	Area%
1	11.065	564622	9387200	28.623	22.444
2	14.413	1407970	32437761	71.377	77.556
Total		1972593	41824961	100.000	100.000

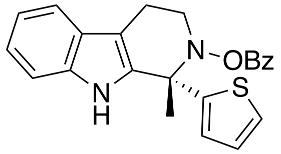

55

Conditions: HPLC (ChiralPak AD-H, 90:10 hexanes/i-PrOH, 1 mL/min, 254 nm)

Racemic Sample:

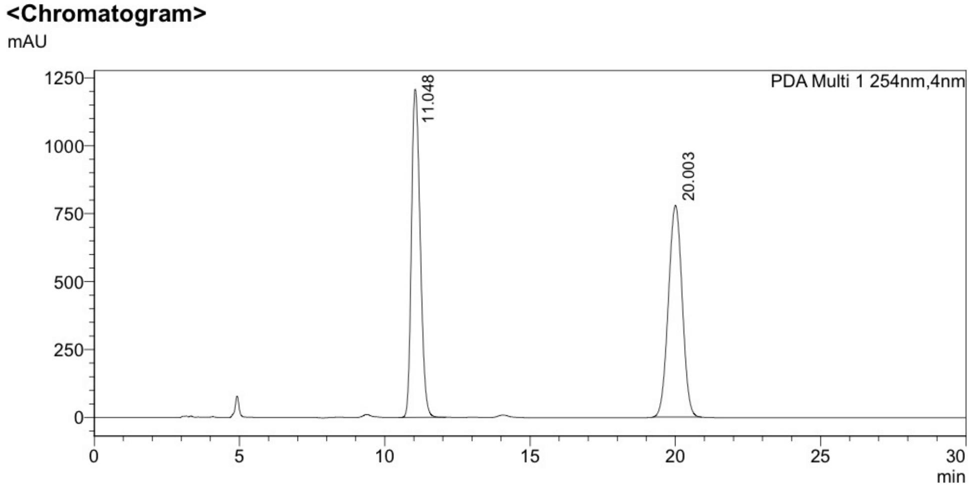

<Peak Table>

PDA Ch1 254nm

Peak#	Ret. Time	Height	Area	Height%	Area%
1	11.048	1208916	25351279	60.817	49.617
2	20.003	778873	25742938	39.183	50.383
Total		1987789	51094216	100.000	100.000

Enantioenriched Sample:

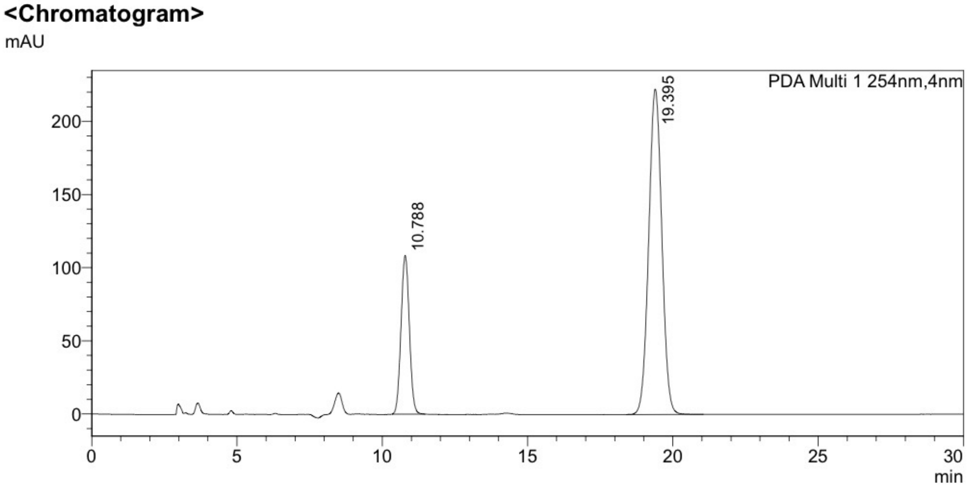

<Peak Table>

PDA Ch1 254nm

Peak#	Ret. Time	Height	Area	Height%	Area%
1	10.788	108314	2152604	32.748	23.595
2	19.395	222432	6970402	67.252	76.405
Total		330746	9123006	100.000	100.000

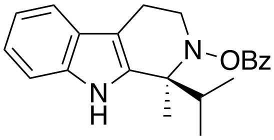

56

Conditions: HPLC (ChiralPak AD-H, 90:10 hexanes/i-PrOH, 1 mL/min, 254 nm)

Racemic Sample:

<Chromatogram>

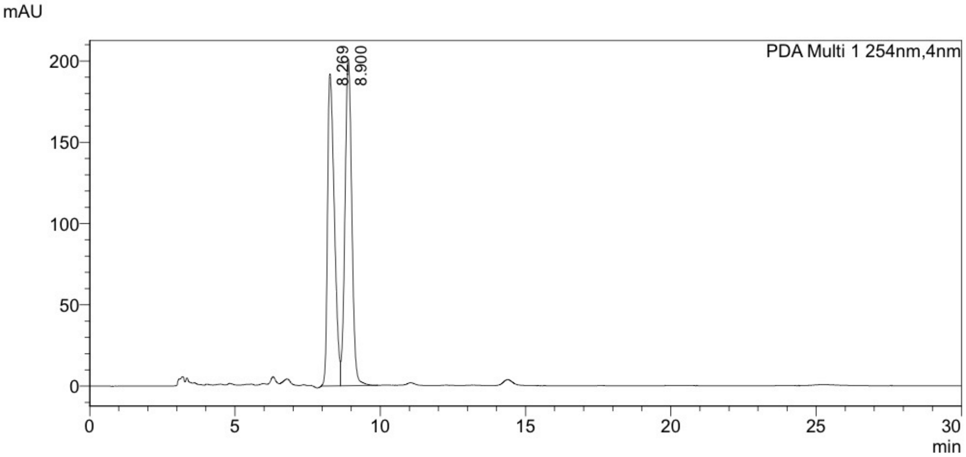

<Peak Table>

PDA Ch1 254nm

Peak#	Ret. Time	Height	Area	Height%	Area%
1	8.269	192083	3130806	48.871	48.895
2	8.900	200956	3272371	51.129	51.105
Total		393039	6403177	100.000	100.000

Enantioenriched Sample:

<Chromatogram>

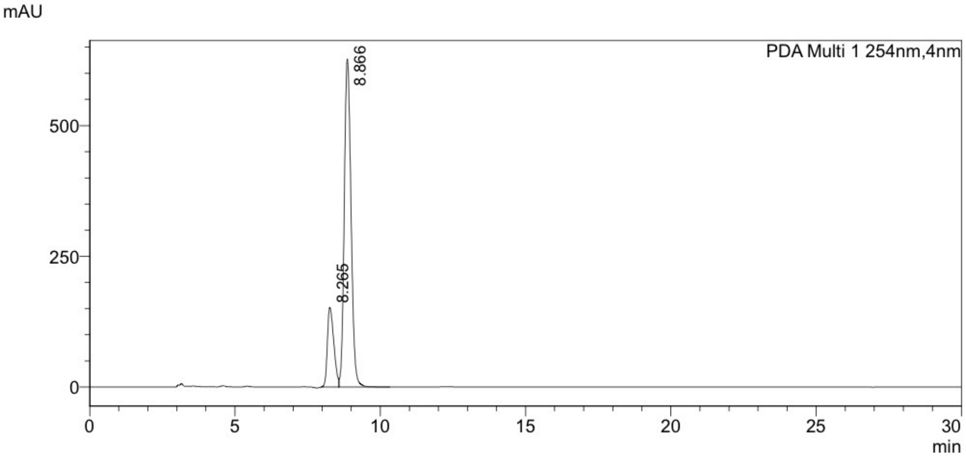

<Peak Table>

PDA Ch1 254nm

Peak#	Ret. Time	Height	Area	Height%	Area%
1	8.265	152396	2350053	19.545	18.775
2	8.866	627305	10166924	80.455	81.225
Total		779701	12516977	100.000	100.000

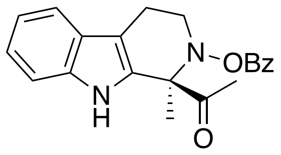

57

Conditions: HPLC (ChiralPak AD-H, 90:10 hexanes/i-PrOH, 1 mL/min, 254 nm)

Racemic Sample:

<Chromatogram>

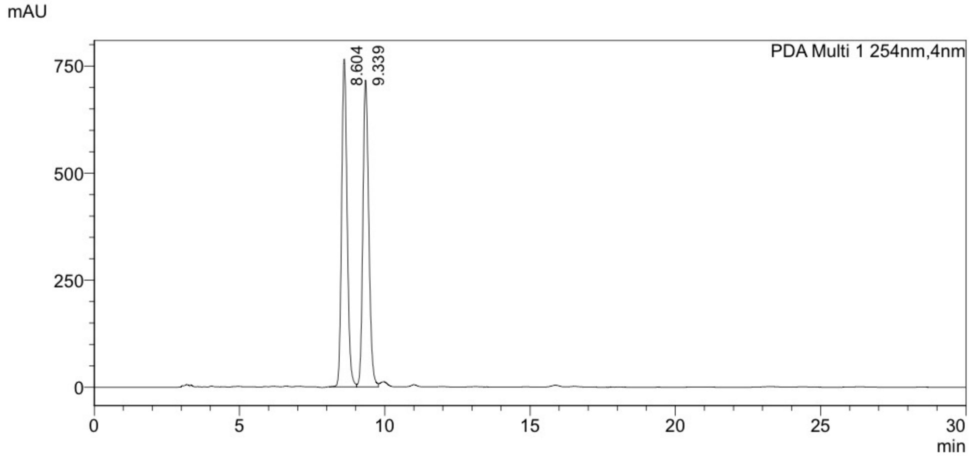

<Peak Table>

PDA Ch1 254nm

Peak#	Ret. Time	Height	Area	Height%	Area%
1	8.604	765554	10079971	51.669	49.982
2	9.339	716089	10087227	48.331	50.018
Total		1481642	20167198	100.000	100.000

Enantioenriched Sample:

<Chromatogram>

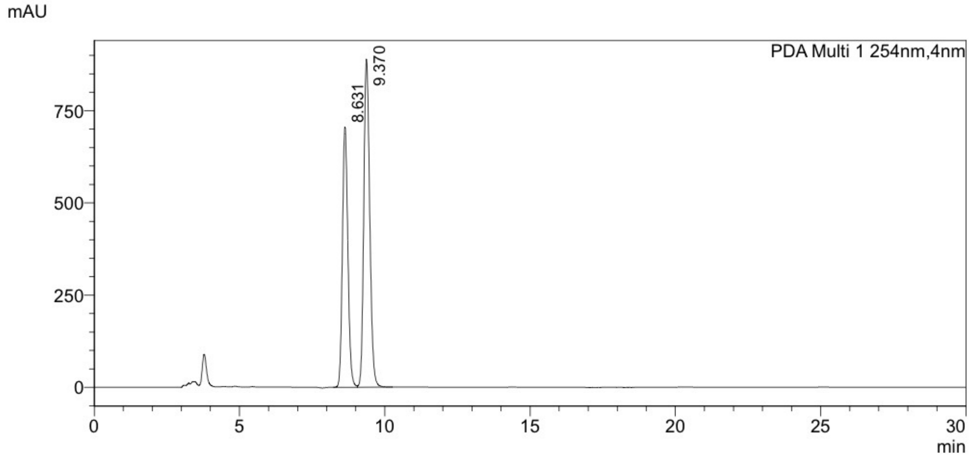

<Peak Table>

PDA Ch1 254nm

Peak#	Ret. Time	Height	Area	Height%	Area%
1	8.631	705922	9185569	44.244	42.134
2	9.370	889606	12615307	55.756	57.866
Total		1595528	21800876	100.000	100.000

N. X-Ray Crystallography Data

Crystal Structure of 20a

General information: The diffraction data were measured at 100 K on a Bruker D8 VENTURE diffractometer equipped with a microfocus Mo-target X-ray tube (λ = 0.71073 Å) and PHOTON 100 CMOS detector. Data were collected using ϕ and ω scans to survey a hemisphere of reciprocal space. Data reduction and integration were performed with the Bruker APEX3 software package (Bruker AXS, version 2017.3-0, 2018). Data were scaled and corrected for absorption effects using the multi-scan procedure as implemented in SADABS (Bruker AXS, version 2014/5, Krause, Herbst-Irmer, Sheldrick & Stalke, *J. Appl. Cryst.* **2015**, *48*, 3-10). The structure was solved by SHELXT (Version 2014/5: Sheldrick, G. M. *Acta Crystallogr*. **2015**, ***A71***, 3-8) and refined by a full-matrix least-squares procedure using OLEX2 (O. V. Dolomanov, L. J. Bourhis, R. J. Gildea, J. A. K. Howard and H. Puschmann. J. Appl. Crystallogr. **2009**, *42*, 339-341) (XL refinement program version 2018/3, *Sheldrick, G. M. Acta Crystallogr.* **2015**, *C71*, 3-8). Crystallographic data and details of the data collection and structure refinement are listed in Table S4.

Specific details for structure refinement: All atoms were refined with anisotropic thermal parameters. Hydrogen atoms were included in idealized positions for structure factor calculations except atom H2 attached to nitrogen atom N2. This hydrogen atom was located in the difference Fourier map and allowed to be refined without any additional restraints. All structures are drawn with thermal ellipsoids at 50% probability.

Table S4 Crystal data and structure refinement for 0732_LC.

Identification code	0732_LC
Empirical formula	$C_{25}H_{22}N_2O_2$
Formula weight	382.44
Temperature/K	100(2)
Crystal system	monoclinic
Space group	$P2_1/c$
a/Å	9.5752(5)
b/Å	12.0481(7)
c/Å	17.8268(10)
α/°	90
β/°	101.373(2)
γ/°	90
Volume/Å^3	2016.17(19)
Z	4
ρ_{calc}g/cm^3	1.260
μ/mm^{-1}	0.080
F(000)	808.0
Crystal size/mm^3	0.307 × 0.108 × 0.107
Radiation	MoKα (λ = 0.71073)
2Θ range for data collection/°	4.662 to 53.01
Index ranges	$-12 \le h \le 12$, $-15 \le k \le 15$, $-22 \le l \le 22$
Reflections collected	50007
Independent reflections	4167 [R_{int} = 0.0400, R_{sigma} = 0.0184]
Data/restraints/parameters	4167/0/267
Goodness-of-fit on F^2	1.023
Final R indexes [I>=2σ (I)]	R_1 = 0.0357, wR_2 = 0.0812
Final R indexes [all data]	R_1 = 0.0481, wR_2 = 0.0879
Largest diff. peak/hole / e Å^{-3}	0.27/-0.18

$R_{int} = \Sigma \mid F_o^2 - \langle F_o^2 \rangle \mid / \Sigma \mid F_o^2 \mid$

$R1 = \Sigma \mid \mid F_o \mid - \mid F_c \mid \mid / \Sigma \mid F_o \mid$

$wR2 = [\Sigma [w (F_o^2 - F_c^2)^2] / \Sigma [w (F_o^2)^2]]^{1/2}$

Goodness-of-fit = $[\Sigma [w (F_o^2 - F_c^2)^2] / (n-p)^{1/2}$

n: number of independent reflections; p: number of refined parameters

Figure S2.

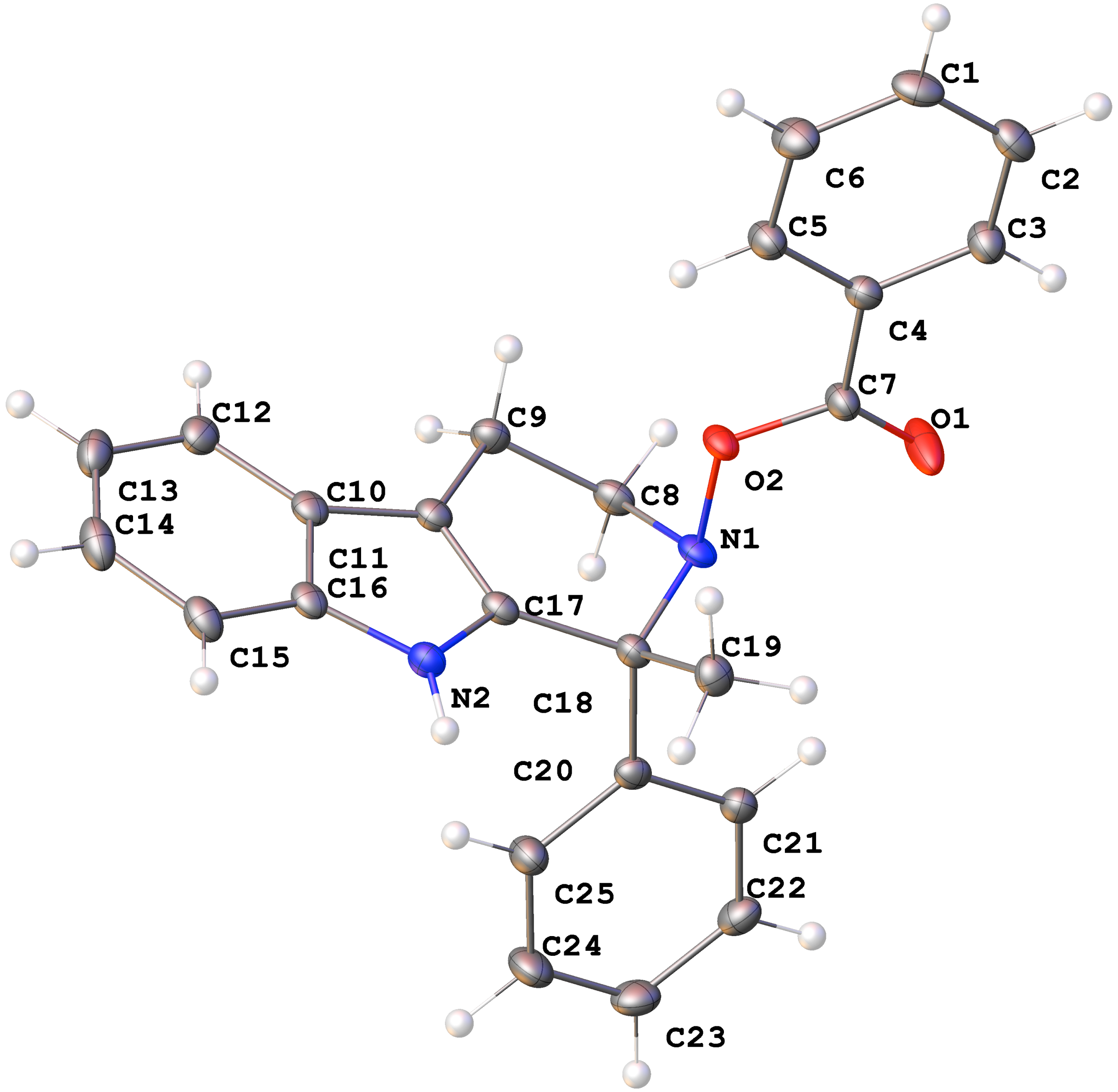
C1
C6
C2
C5
C3
C4
C7
O1
C12
C9
O2
C13
C10
C8
C14
N1
C11
C16
C17
C19
C15
N2
C18
C20
C21
C25
C22
C24
C23

Figure S3.

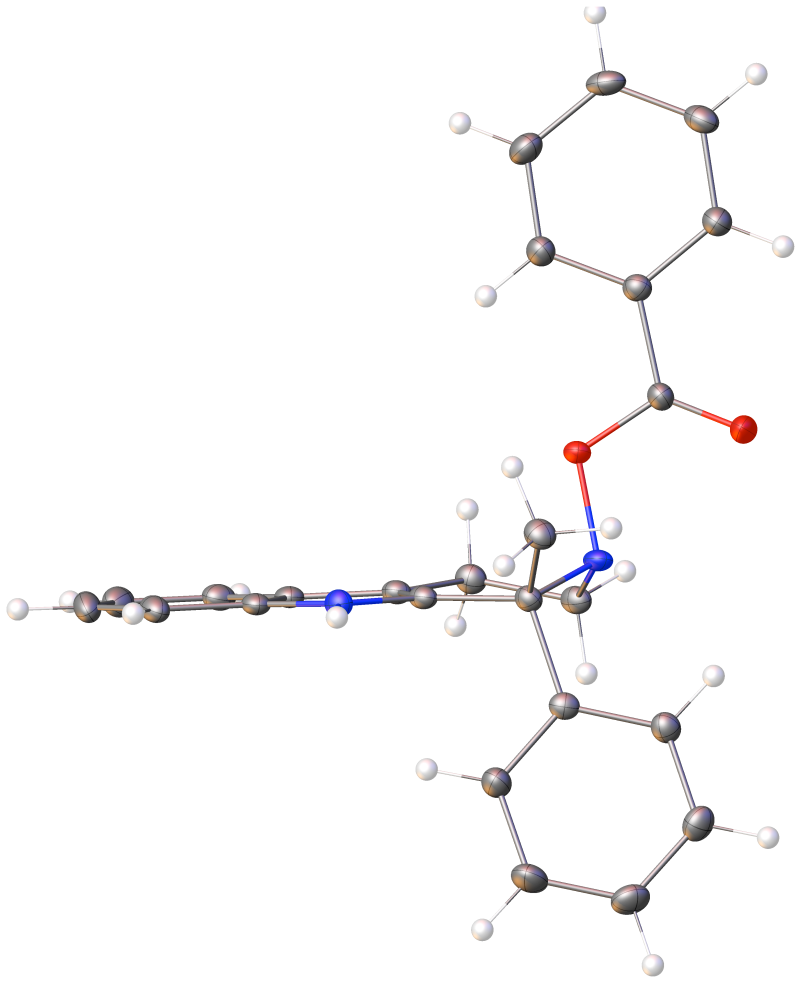

Figure S4.

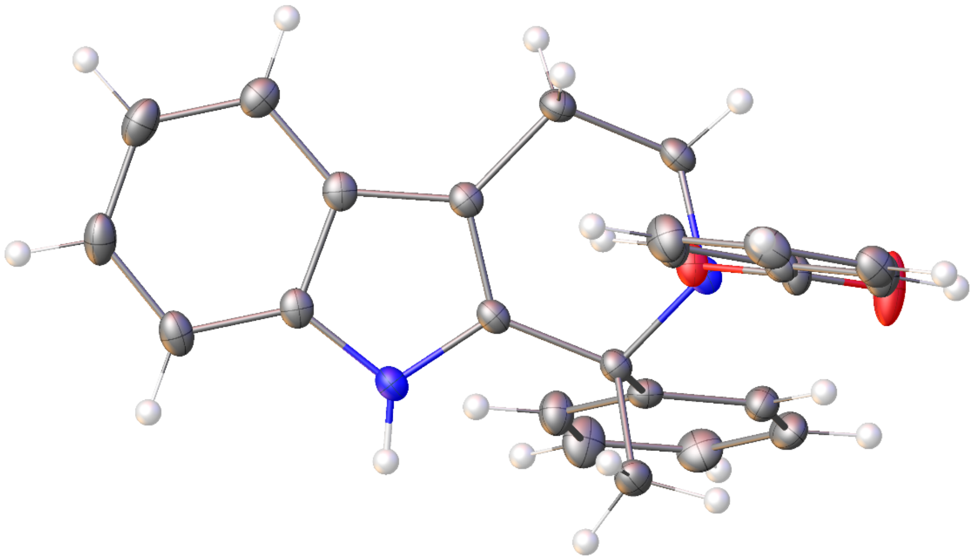

Figure S5.

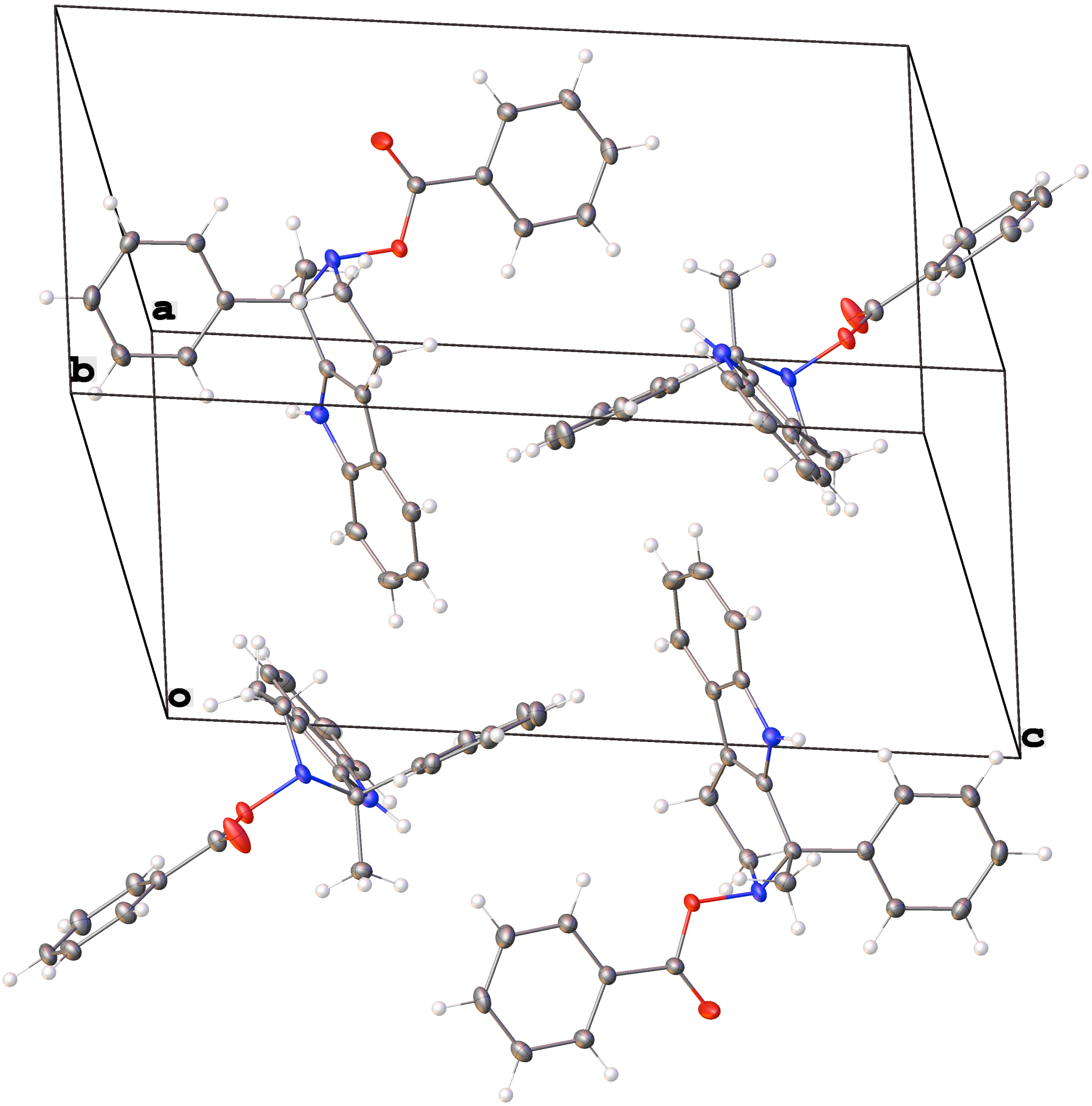

Table S5 Fractional Atomic Coordinates (×10[4]) and Equivalent Isotropic Displacement Parameters (Å[2]×10[3]) for 0732_LC. U_{eq} is defined as 1/3 of of the trace of the orthogonalised U_{IJ} tensor.

Atom	*x*	*y*	*z*	U(eq)
O1	6999.6(11)	9878.1(9)	3763.2(6)	44.7(3)
O2	5319.8(9)	8576.2(7)	3788.4(4)	21.14(19)
N1	4605.3(10)	9014.0(8)	3041.3(5)	19.6(2)
N2	2857.5(11)	6247.6(8)	2543.7(6)	19.7(2)
C1	8660.9(15)	7893.7(11)	6232.5(7)	28.2(3)
C2	9230.2(14)	8749.2(11)	5878.8(7)	26.7(3)
C3	8516.7(13)	9131.1(11)	5176.5(7)	25.5(3)
C4	7235.5(13)	8654.7(10)	4824.9(7)	20.0(3)
C5	6664.0(14)	7788.5(11)	5180.9(7)	24.9(3)
C6	7388.1(15)	7409.4(12)	5883.4(7)	30.6(3)
C7	6534.7(13)	9117.4(10)	4071.8(7)	21.8(3)
C8	3314.0(13)	9552.4(10)	3198.6(7)	21.3(3)
C9	2336.9(13)	8814.1(10)	3563.5(7)	21.8(3)
C10	2234.2(12)	7693.4(10)	3203.8(6)	19.2(3)
C11	1283.0(12)	6792.8(10)	3261.5(6)	20.2(3)
C12	113.0(13)	6652.2(11)	3616.3(7)	24.6(3)
C13	-566.8(14)	5638.2(12)	3559.1(8)	30.2(3)
C14	-110.9(14)	4763.9(11)	3151.5(8)	30.2(3)
C15	1018.2(13)	4879.7(11)	2782.5(7)	25.5(3)
C16	1707.2(12)	5903.2(10)	2841.2(7)	20.3(3)
C17	3152.9(12)	7336.2(10)	2761.2(6)	17.9(2)
C18	4301.6(12)	8025.4(10)	2526.3(6)	18.5(3)
C19	5678.6(13)	7361.2(11)	2562.6(7)	24.0(3)
C20	3827.8(12)	8499.1(10)	1714.9(6)	18.9(2)
C21	4516.4(13)	9414.3(10)	1481.4(7)	22.5(3)
C22	4110.7(14)	9831.7(11)	747.6(7)	26.8(3)
C23	3033.0(15)	9332.9(12)	227.3(7)	30.8(3)
C24	2358.0(15)	8417.2(13)	451.0(7)	33.9(3)
C25	2743.7(14)	8003.9(11)	1189.4(7)	26.5(3)

Table S6 Anisotropic Displacement Parameters (Å[2]×10[3]) for 0732_LC. The Anisotropic displacement factor exponent takes the form: $-2\pi^2[h^2a^{*2}U_{11}+2hka^*b^*U_{12}+\ldots]$.

Atom	U_{11}	U_{22}	U_{33}	U_{23}	U_{13}	U_{12}
O1	41.8(6)	49.2(7)	34.0(6)	22.7(5)	-14.7(5)	-27.1(5)
O2	22.2(4)	22.5(4)	15.7(4)	3.6(3)	-3.5(3)	-4.7(4)
N1	21.7(5)	20.0(5)	14.1(5)	1.5(4)	-3.6(4)	-0.1(4)

N2	22.2(5)	16.6(5)	19.6(5)	-1.3(4)	2.0(4)	-0.2(4)
C1	35.6(7)	28.0(7)	17.5(6)	1.8(5)	-3.4(5)	7.0(6)
C2	23.3(6)	27.4(7)	25.7(7)	-2.3(5)	-4.0(5)	1.0(5)
C3	23.6(6)	25.9(7)	25.2(6)	3.5(5)	-0.2(5)	-3.7(5)
C4	22.0(6)	19.8(6)	17.5(6)	-0.8(5)	2.1(5)	-0.4(5)
C5	27.1(7)	24.2(7)	21.7(6)	0.8(5)	1.0(5)	-5.5(5)
C6	39.4(8)	27.6(7)	23.6(7)	6.7(6)	3.1(6)	-3.4(6)
C7	22.0(6)	21.8(6)	20.1(6)	0.9(5)	0.0(5)	-5.3(5)
C8	25.1(6)	17.4(6)	19.7(6)	-3.2(5)	0.1(5)	1.2(5)
C9	23.4(6)	21.8(6)	20.0(6)	-3.4(5)	3.9(5)	0.8(5)
C10	19.7(6)	20.6(6)	15.6(6)	0.3(5)	-0.7(4)	0.4(5)
C11	19.6(6)	22.4(6)	15.9(6)	3.3(5)	-3.0(4)	-0.1(5)
C12	19.7(6)	31.7(7)	20.7(6)	3.1(5)	0.1(5)	0.8(5)
C13	19.3(6)	38.5(8)	31.3(7)	9.3(6)	1.4(5)	-3.5(6)
C14	22.5(6)	25.9(7)	38.0(8)	7.9(6)	-4.1(6)	-6.1(5)
C15	23.4(6)	20.8(6)	28.0(7)	2.5(5)	-5.2(5)	-1.4(5)
C16	19.0(6)	21.1(6)	17.7(6)	3.4(5)	-3.7(4)	-0.2(5)
C17	19.5(6)	16.4(6)	15.7(5)	-0.2(4)	-1.6(4)	1.3(5)
C18	19.3(6)	17.9(6)	17.3(6)	-1.7(5)	1.0(5)	-1.0(5)
C19	20.1(6)	24.5(7)	26.8(6)	-0.6(5)	2.8(5)	1.9(5)
C20	18.4(6)	21.1(6)	17.3(6)	-1.6(5)	3.8(4)	2.7(5)
C21	22.9(6)	24.5(7)	20.2(6)	-2.3(5)	4.8(5)	-2.7(5)
C22	32.4(7)	26.5(7)	24.1(7)	2.4(5)	11.7(5)	-1.5(6)
C23	36.5(8)	37.9(8)	18.0(6)	4.9(6)	5.0(5)	1.5(6)
C24	33.8(7)	42.6(9)	21.0(7)	0.4(6)	-4.9(6)	-8.4(6)
C25	28.0(7)	28.2(7)	21.8(6)	1.0(5)	0.8(5)	-6.8(5)

Table S7 Bond Lengths for 0732_LC.

Atom	Atom	Length/Å	Atom	Atom	Length/Å
O1	C7	1.1988(15)	C10	C17	1.3621(16)
O2	N1	1.4699(12)	C11	C12	1.4011(17)
O2	C7	1.3423(14)	C11	C16	1.4125(17)
N1	C8	1.4715(16)	C12	C13	1.3785(19)
N1	C18	1.4970(15)	C13	C14	1.398(2)
N2	C16	1.3774(16)	C14	C15	1.3789(19)
N2	C17	1.3811(15)	C15	C16	1.3927(17)
C1	C2	1.3757(19)	C17	C18	1.5020(16)
C1	C6	1.3835(19)	C18	C19	1.5327(16)
C2	C3	1.3816(17)	C18	C20	1.5379(16)
C3	C4	1.3871(17)	C20	C21	1.3899(17)

C4	C5	1.3886(17)	C20	C25	1.3884(17)
C4	C7	1.4861(16)	C21	C22	1.3838(17)
C5	C6	1.3839(18)	C22	C23	1.3816(19)
C8	C9	1.5263(17)	C23	C24	1.377(2)
C9	C10	1.4897(17)	C24	C25	1.3875(18)
C10	C11	1.4333(17)			

Table S8 Bond Angles for 0732_LC.

Atom	Atom	Atom	Angle/°	Atom	Atom	Atom	Angle/°
C7	O2	N1	112.37(9)	C13	C12	C11	118.79(12)
O2	N1	C8	104.12(8)	C12	C13	C14	121.00(12)
O2	N1	C18	105.64(8)	C15	C14	C13	121.77(12)
C8	N1	C18	113.50(9)	C14	C15	C16	117.26(13)
C16	N2	C17	108.24(10)	N2	C16	C11	108.12(10)
C2	C1	C6	120.22(12)	N2	C16	C15	129.85(12)
C1	C2	C3	119.68(12)	C15	C16	C11	122.02(12)
C2	C3	C4	120.45(12)	N2	C17	C18	124.57(10)
C3	C4	C5	119.84(11)	C10	C17	N2	110.05(11)
C3	C4	C7	117.03(11)	C10	C17	C18	125.37(11)
C5	C4	C7	123.12(11)	N1	C18	C17	109.47(9)
C6	C5	C4	119.30(12)	N1	C18	C19	109.15(9)
C1	C6	C5	120.51(12)	N1	C18	C20	105.49(9)
O1	C7	O2	124.84(11)	C17	C18	C19	111.76(10)
O1	C7	C4	123.99(11)	C17	C18	C20	111.78(9)
O2	C7	C4	111.17(10)	C19	C18	C20	108.98(9)
N1	C8	C9	115.66(10)	C21	C20	C18	120.44(10)
C10	C9	C8	109.80(10)	C25	C20	C18	121.18(11)
C11	C10	C9	130.11(11)	C25	C20	C21	118.34(11)
C17	C10	C9	122.71(11)	C22	C21	C20	120.67(12)
C17	C10	C11	107.13(11)	C23	C22	C21	120.65(12)
C12	C11	C10	134.41(12)	C24	C23	C22	119.04(12)
C12	C11	C16	119.13(11)	C23	C24	C25	120.66(12)
C16	C11	C10	106.45(10)	C24	C25	C20	120.64(12)

Table S9 Hydrogen Atom Coordinates (Å×10^4) and Isotropic Displacement Parameters (Å^2×10^3) for 0732_LC.

Atom	*x*	*y*	*z*	U(eq)
H2	3224(17)	5863(13)	2194(9)	38(4)

H1	9143.41	7634.58	6718.1	34
H2A	10109.62	9075.67	6116.31	32
H3	8906.19	9724.18	4932.64	31
H5	5785.19	7459.68	4944.77	30
H6	7008.29	6812.02	6127.7	37
H8A	2759.99	9845.98	2711.07	26
H8B	3606.64	10193.68	3540.8	26
H9A	2719.61	8747.24	4119.57	26
H9B	1377.46	9152.23	3492.66	26
H12	-206.01	7244.91	3891.22	30
H13	-1357.14	5532.15	3800.41	36
H14	-591.76	4071.31	3127.98	36
H15	1313.99	4285.65	2499.87	31
H19A	6443.37	7862.04	2484.15	36
H19B	5528.51	6791.82	2162.19	36
H19C	5944.22	7005.1	3064.82	36
H21	5273.26	9756.64	1829.22	27
H22	4577.82	10467.61	600.06	32
H23	2761.7	9617.57	-277.38	37
H24	1620.69	8064.81	96.26	41
H25	2261.41	7375.99	1336.77	32

Crystal Structure of 24

General information: The diffraction data were measured at 100 K on a Bruker D8 VENTURE diffractometer equipped with a microfocus Mo-target X-ray tube (λ = 0.71073 Å) and PHOTON 100 CMOS detector. Data were collected using ϕ and ω scans to survey a hemisphere of reciprocal space. Data reduction and integration were performed with the Bruker APEX3 software package (Bruker AXS, version 2017.3-0, 2018). Data were scaled and corrected for absorption effects using the multi-scan procedure as implemented in SADABS (Bruker AXS, version 2014/5, Krause, Herbst-Irmer, Sheldrick & Stalke, *J. Appl. Cryst.* **2015**, *48*, 3-10). The structure was solved by SHELXT (Version 2014/5: Sheldrick, G. M. *Acta Crystallogr*. **2015**, *A**71***, 3-8) and refined by a full-matrix least-squares procedure using OLEX2 (O. V. Dolomanov, L. J. Bourhis, R. J. Gildea, J. A. K. Howard and H. Puschmann. J. Appl. Crystallogr. **2009**, *42*, 339-341) (XL refinement program version 2018/3, *Sheldrick, G. M. Acta Crystallogr.* **2015**, *C71*, 3-8). Crystallographic data and details of the data collection and structure refinement are listed in Table S10.

Specific details for structure refinement: All atoms were refined with anisotropic thermal parameters. Hydrogen atoms were included in idealized positions for structure factor calculations except an H-atom attached to an indole nitrogen atom. This hydrogen atom was located in the difference Fourier map and allowed to be freely refined with the thermal parameter being constrained to be 1.2 times of the Ueq value of the N atom. All structures are drawn with thermal ellipsoids at 50% probability.

Table S10 Crystal data and structure refinement for mo_0877_tes.

Identification code	mo_0877_tes
Empirical formula	$C_{25}H_{23}N_3O_2$
Formula weight	397.46
Temperature/K	100(2)
Crystal system	triclinic
Space group	P-1
a/Å	7.7539(3)
b/Å	10.3038(5)
c/Å	13.4620(6)
α/°	107.202(2)
β/°	98.294(2)
γ/°	96.619(2)
Volume/Å^3	1002.39(8)
Z	2
ρ_{calc}g/cm^3	1.317
μ/mm^{-1}	0.085
F(000)	420.0
Crystal size/mm^3	0.41 × 0.28 × 0.14
Radiation	MoKα (λ = 0.71073)
2Θ range for data collection/°	4.406 to 56.86
Index ranges	-10 ≤ h ≤ 10, -13 ≤ k ≤ 13, -17 ≤ l ≤ 18
Reflections collected	32154
Independent reflections	5032 [R_{int} = 0.0383, R_{sigma} = 0.0309]
Data/restraints/parameters	5032/0/275
Goodness-of-fit on F^2	1.032
Final R indexes [I>=2σ (I)]	R_1 = 0.0425, wR_2 = 0.0965
Final R indexes [all data]	R_1 = 0.0618, wR_2 = 0.1050
Largest diff. peak/hole / e Å^{-3}	0.35/-0.21

$R_{int} = \Sigma | F_o^2 - \langle F_o^2 \rangle | / \Sigma | F_o^2|$

$R1 = \Sigma || F_o| - | F_c|| / \Sigma| F_o|$

$wR2 = [\Sigma [w (F_o^2 - F_c^2)^2] / \Sigma [w (F_o^2)^2]]^{1/2}$

Goodness-of-fit = $[\Sigma [w (F_o^2 - F_c^2)^2] / (n-p)]^{1/2}$

n: number of independent reflections; p: number of refined parameters

Figure S6.

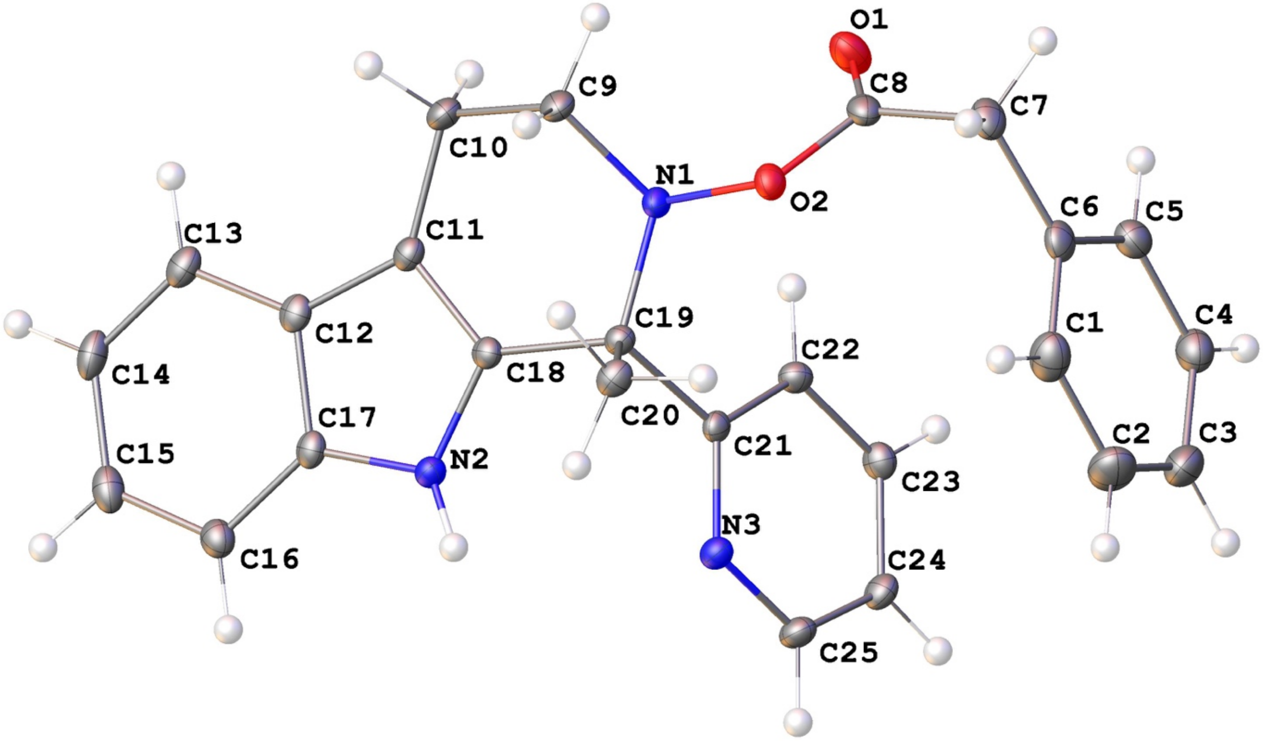

Figure S7.

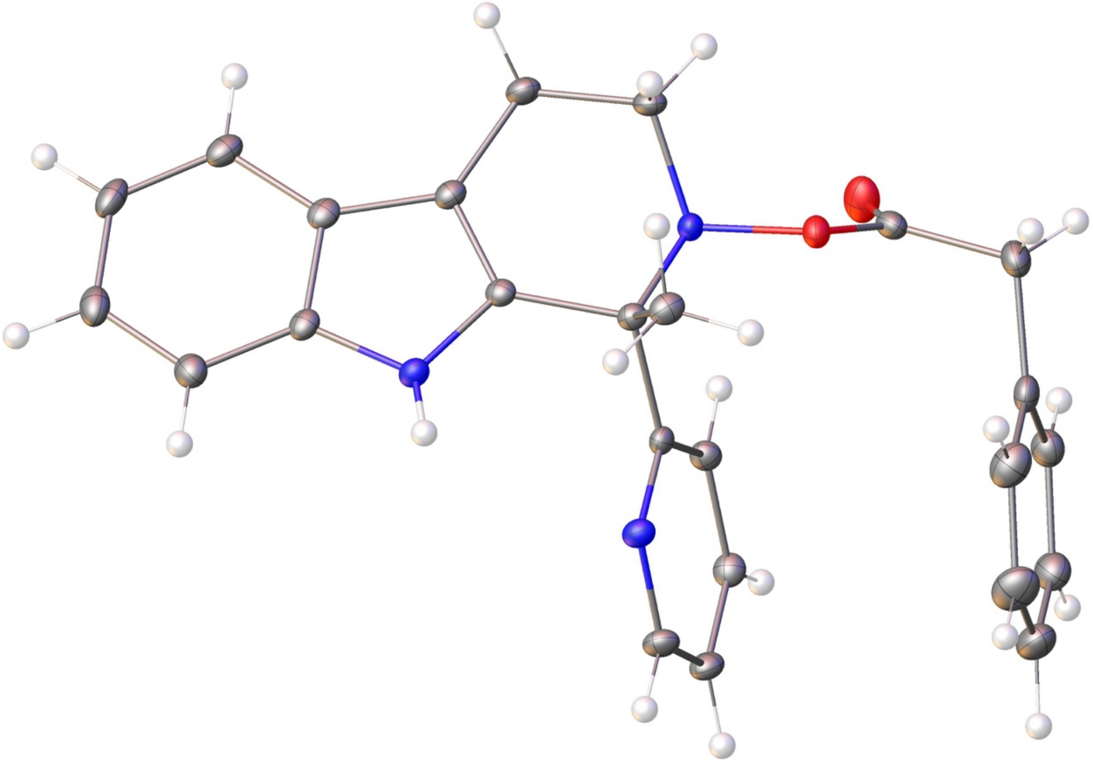

Figure S8.

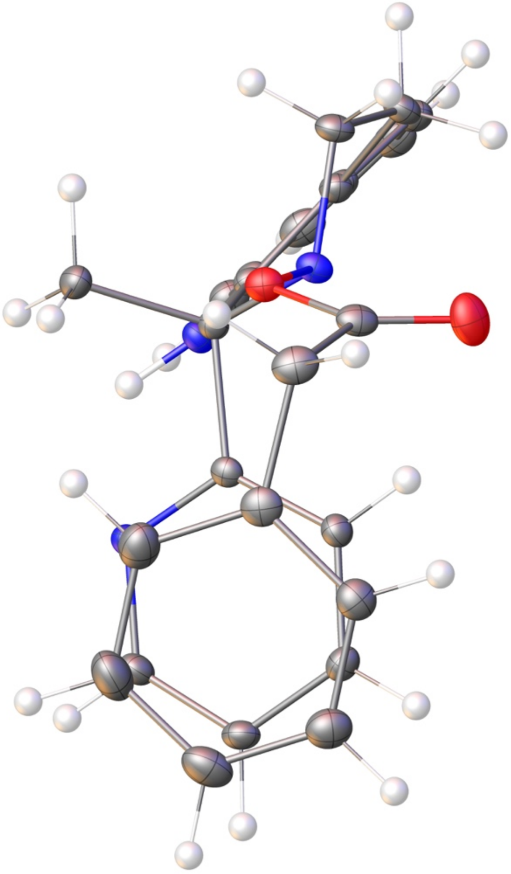

Figure S9.

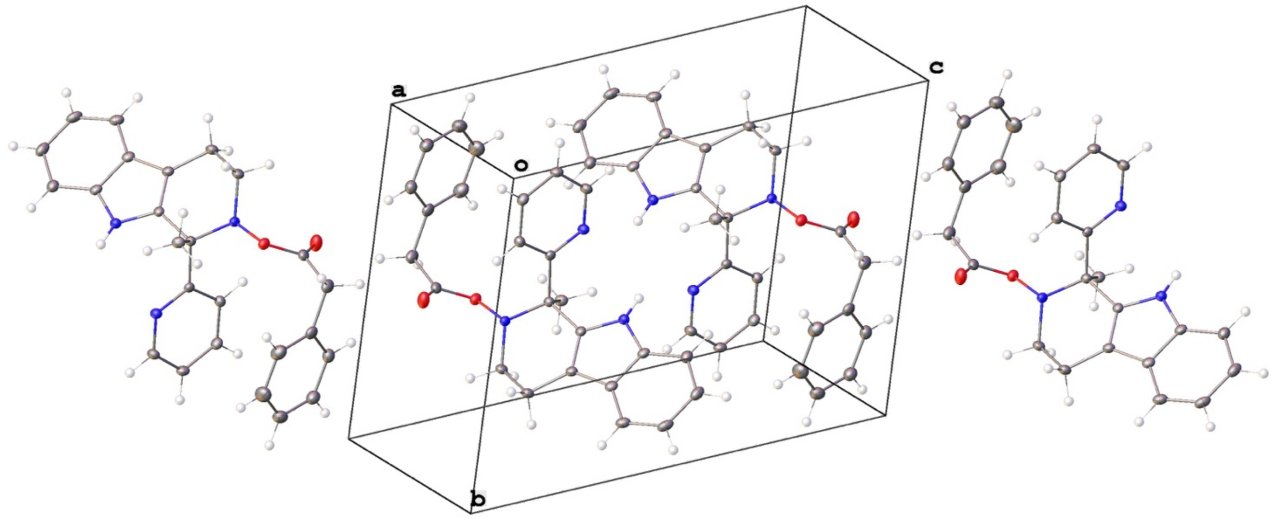

Table S11 Fractional Atomic Coordinates (×10^4) and Equivalent Isotropic Displacement Parameters (Å^2×10^3) for mo_0877_tes. U_{eq} is defined as 1/3 of of the trace of the orthogonalised U_{IJ} tensor.

Atom	*x*	*y*	*z*	U(eq)
O1	852.0(13)	4003.5(10)	8854.2(8)	29.0(2)
O2	3530.4(11)	4503.0(8)	8440.0(6)	17.86(19)
N1	2885.3(13)	3408.5(10)	7426.2(8)	15.5(2)
N2	3566.4(13)	2809.6(10)	4698.6(8)	15.8(2)
N3	3409.8(13)	5829.6(10)	5940.4(8)	16.9(2)
C1	3639.7(19)	7995.5(15)	9185.7(12)	30.3(3)
C2	3165(2)	9020.7(15)	8777.9(13)	35.2(4)
C3	1459(2)	9286.9(14)	8712.2(12)	31.0(3)
C4	225.6(19)	8538.1(14)	9063.5(11)	26.7(3)
C5	694.6(18)	7515.8(13)	9470.8(10)	23.1(3)
C6	2408.4(17)	7232.8(13)	9534.9(10)	20.9(3)
C7	2867.4(18)	6063.8(13)	9937.3(10)	23.6(3)
C8	2255.6(17)	4732.0(13)	9038.4(10)	19.4(3)
C9	3464.3(17)	2140.1(12)	7550.3(10)	18.5(3)
C10	2623.3(17)	940.2(12)	6551.9(10)	19.3(3)
C11	2855.7(15)	1364.0(12)	5603.3(10)	16.0(2)
C12	2675.4(15)	568.6(12)	4510.0(10)	17.1(2)
C13	2106.3(16)	-826.8(12)	3916.7(10)	20.7(3)
C14	2068.3(17)	-1240.1(13)	2840.1(11)	23.3(3)
C15	2625.3(17)	-300.6(13)	2335.6(10)	23.6(3)
C16	3188.4(17)	1084.1(13)	2895.6(10)	20.5(3)
C17	3168.9(15)	1502.5(12)	3974.3(9)	16.2(2)
C18	3383.7(15)	2701.0(12)	5676.2(9)	14.5(2)
C19	3697.5(15)	3937.9(11)	6659.2(9)	14.3(2)
C20	5688.4(16)	4527.1(12)	7017.4(10)	18.6(3)
C21	2647.7(15)	5027.1(11)	6433.7(9)	13.7(2)
C22	984.6(16)	5141.2(12)	6693.4(9)	16.6(2)
C23	106.5(16)	6140.5(12)	6459.6(10)	18.2(3)
C24	880.6(16)	6977.4(12)	5956.6(10)	18.8(3)
C25	2517.3(17)	6777.2(12)	5710.2(10)	19.3(3)

Table S12 Anisotropic Displacement Parameters (Å^2×10^3) for mo_0877_tes. The Anisotropic displacement factor exponent takes the form: $-2\pi^2[h^2a^{*2}U_{11}+2hka^*b^*U_{12}+\ldots]$.

Atom	U_{11}	U_{22}	U_{33}	U_{23}	U_{13}	U_{12}
O1	32.2(5)	28.3(5)	25.0(5)	4.7(4)	13.3(4)	-0.9(4)
O2	20.9(4)	17.3(4)	13.9(4)	2.8(3)	2.4(3)	4.6(3)

N1	21.2(5)	12.8(5)	12.4(5)	3.3(4)	3.5(4)	4.1(4)
N2	17.7(5)	13.3(5)	17.3(5)	5.2(4)	5.5(4)	2.7(4)
N3	17.5(5)	15.2(5)	19.8(5)	7.0(4)	5.6(4)	4.0(4)
C1	26.2(7)	26.8(7)	36.5(8)	6.0(6)	9.8(6)	5.2(6)
C2	37.0(8)	27.2(7)	44.4(9)	13.6(7)	16.0(7)	1.2(6)
C3	43.9(9)	20.7(7)	31.3(8)	9.4(6)	11.1(7)	8.6(6)
C4	29.8(7)	26.6(7)	24.8(7)	7.2(5)	7.0(6)	9.9(6)
C5	25.9(7)	23.7(6)	19.9(6)	5.9(5)	6.8(5)	4.3(5)
C6	26.0(7)	18.2(6)	14.4(6)	-0.6(5)	2.8(5)	4.1(5)
C7	29.1(7)	25.4(7)	14.9(6)	3.7(5)	2.4(5)	8.9(5)
C8	26.2(7)	20.3(6)	15.5(6)	8.5(5)	6.0(5)	8.5(5)
C9	23.3(6)	16.4(6)	19.3(6)	9.1(5)	4.7(5)	7.1(5)
C10	24.0(6)	14.2(6)	22.2(6)	8.3(5)	6.0(5)	4.8(5)
C11	15.7(6)	13.7(5)	19.0(6)	5.1(5)	3.4(5)	4.4(4)
C12	14.0(6)	16.6(6)	20.6(6)	5.0(5)	2.3(5)	5.7(4)
C13	19.1(6)	15.2(6)	26.9(7)	5.7(5)	2.3(5)	5.1(5)
C14	21.7(6)	15.6(6)	26.5(7)	-1.4(5)	-0.3(5)	6.2(5)
C15	23.7(7)	24.9(7)	18.9(6)	0.6(5)	2.1(5)	10.0(5)
C16	21.4(6)	21.8(6)	19.3(6)	6.0(5)	4.9(5)	7.6(5)
C17	13.9(6)	15.0(5)	19.1(6)	3.4(5)	2.8(5)	5.3(4)
C18	13.9(5)	15.2(5)	16.3(6)	6.0(4)	4.3(4)	5.4(4)
C19	14.8(5)	13.5(5)	15.8(6)	5.5(4)	3.7(4)	3.2(4)
C20	15.2(6)	17.1(6)	23.1(6)	6.4(5)	2.5(5)	2.5(5)
C21	16.0(6)	11.2(5)	12.3(5)	1.8(4)	1.8(4)	1.9(4)
C22	17.0(6)	16.5(6)	17.4(6)	6.7(5)	4.2(5)	2.0(5)
C23	15.0(6)	20.1(6)	19.6(6)	5.3(5)	4.4(5)	4.8(5)
C24	20.4(6)	15.7(6)	21.9(6)	7.3(5)	3.0(5)	6.5(5)
C25	21.7(6)	16.1(6)	23.7(6)	10.3(5)	6.4(5)	4.0(5)

Table S13 Bond Lengths for mo_0877_tes.

Atom	Atom	Length/Å	Atom	Atom	Length/Å
O1	C8	1.1971(16)	C9	C10	1.5293(17)
O2	N1	1.4654(12)	C10	C11	1.4939(17)
O2	C8	1.3613(15)	C11	C12	1.4347(17)
N1	C9	1.4766(15)	C11	C18	1.3614(16)
N1	C19	1.4838(15)	C12	C13	1.4046(17)
N2	C17	1.3785(15)	C12	C17	1.4135(17)
N2	C18	1.3792(15)	C13	C14	1.3793(19)
N3	C21	1.3414(15)	C14	C15	1.401(2)
N3	C25	1.3420(15)	C15	C16	1.3855(18)

C1	C2	1.388(2)	C16	C17	1.3901(17)
C1	C6	1.3874(19)	C18	C19	1.5076(16)
C2	C3	1.378(2)	C19	C20	1.5428(16)
C3	C4	1.382(2)	C19	C21	1.5347(16)
C4	C5	1.3837(19)	C21	C22	1.3927(16)
C5	C6	1.3891(19)	C22	C23	1.3835(17)
C6	C7	1.5166(18)	C23	C24	1.3805(17)
C7	C8	1.5081(17)	C24	C25	1.3801(17)

Table S14 Bond Angles for mo_0877_tes.

Atom	Atom	Atom	Angle/°	Atom	Atom	Atom	Angle/°
C8	O2	N1	112.35(9)	C17	C12	C11	106.67(10)
O2	N1	C9	107.01(8)	C14	C13	C12	119.16(12)
O2	N1	C19	103.77(8)	C13	C14	C15	121.19(12)
C9	N1	C19	114.55(9)	C16	C15	C14	121.17(12)
C17	N2	C18	107.90(10)	C15	C16	C17	117.37(12)
C21	N3	C25	117.50(10)	N2	C17	C12	108.10(10)
C6	C1	C2	120.57(14)	N2	C17	C16	129.30(11)
C3	C2	C1	120.14(14)	C16	C17	C12	122.60(11)
C2	C3	C4	119.72(14)	N2	C18	C19	122.50(10)
C3	C4	C5	120.27(13)	C11	C18	N2	110.71(10)
C4	C5	C6	120.51(13)	C11	C18	C19	126.78(11)
C1	C6	C5	118.79(12)	N1	C19	C18	103.81(9)
C1	C6	C7	121.68(12)	N1	C19	C20	114.74(10)
C5	C6	C7	119.48(12)	N1	C19	C21	107.39(9)
C8	C7	C6	108.00(10)	C18	C19	C20	110.45(9)
O1	C8	O2	124.81(11)	C18	C19	C21	109.18(9)
O1	C8	C7	125.84(12)	C21	C19	C20	110.93(9)
O2	C8	C7	109.32(11)	N3	C21	C19	115.69(10)
N1	C9	C10	108.29(10)	N3	C21	C22	122.28(11)
C11	C10	C9	108.87(10)	C22	C21	C19	121.99(10)
C12	C11	C10	131.26(11)	C23	C22	C21	118.91(11)
C18	C11	C10	122.12(11)	C24	C23	C22	119.34(11)
C18	C11	C12	106.59(11)	C25	C24	C23	117.94(11)
C13	C12	C11	134.87(12)	N3	C25	C24	124.00(11)
C13	C12	C17	118.43(11)				

Crystal Structure of 51

General information: The diffraction data were measured at 100 K on a Bruker D8 VENTURE diffractometer equipped with a microfocus Mo-target X-ray tube (λ = 0.71073 Å) and PHOTON 100 CMOS detector. Data were collected using ω scans to survey a sphere of reciprocal space. Data reduction and integration were performed with the Bruker APEX3 software package (Bruker AXS, version 2017.3-0, 2018). Data were scaled and corrected for absorption effects using the multi-scan procedure as implemented in SADABS (Bruker AXS, version 2014/5, Krause, Herbst-Irmer, Sheldrick & Stalke, *J. Appl. Cryst.* **2015**, *48*, 3-10). The structure was solved by SHELXT (Version 2018/2: Sheldrick, G. M. *Acta Crystallogr*. **2015**, ***A71***, 3-8) and refined by a full-matrix least-squares procedure using OLEX2 (O. V. Dolomanov, L. J. Bourhis, R. J. Gildea, J. A. K. Howard and H. Puschmann. J. Appl. Crystallogr. **2009**, *42*, 339-341) (XL refinement program version 2018/3, Sheldrick, G. M. Acta Crystallogr. **2015**, *C71*, 3-8). Crystallographic data and details of the data collection and structure refinement are listed in Table S15.

Specific details for structure refinement: All atoms were refined with anisotropic thermal parameters. All hydrogen atoms were included in idealized positions for structure factor calculations except the hydrogen atom of the NH group. This atom was found in the difference Fourier map and refined without geometric restraints. All structures are drawn with thermal ellipsoids at 50% probability.

Table S15 Crystal data and structure refinement for 0953_TLC_Snyder_2.

Identification code	0953_TLC_Snyder_2
Empirical formula	$C_{25}H_{21}BrN_2O_2$
Formula weight	461.35
Temperature/K	100(2)
Crystal system	orthorhombic
Space group	$P2_12_12_1$
a/Å	9.8948(6)
b/Å	10.3781(6)
c/Å	20.5618(13)
α/°	90
β/°	90
γ/°	90
Volume/Å^3	2111.5(2)
Z	4
ρ_{calc}g/cm^3	1.451
μ/mm^{-1}	1.971
F(000)	944.0
Crystal size/mm^3	0.12 × 0.051 × 0.045
Radiation	MoKα (λ = 0.71073)
2Θ range for data collection/°	4.396 to 57.446
Index ranges	-13 ≤ h ≤ 12, -14 ≤ k ≤ 13, -27 ≤ l ≤ 27
Reflections collected	43364
Independent reflections	5432 [R_{int} = 0.0474, R_{sigma} = 0.0470]
Data/restraints/parameters	5432/0/275
Goodness-of-fit on F^2	1.056
Final R indexes [I>=2σ (I)]	R_1 = 0.0338, wR_2 = 0.0616
Final R indexes [all data]	R_1 = 0.0468, wR_2 = 0.0646
Largest diff. peak/hole / e Å^{-3}	0.73/-0.36
Flack parameter	0.012(3)

$R_{int} = \Sigma \mid F_o^2 - \langle F_o^2 \rangle \mid / \Sigma \mid F_o^2 \mid$

$R1 = \Sigma \mid \mid F_o \mid - \mid F_c \mid \mid / \Sigma \mid F_o \mid$

$wR2 = [\Sigma [w (F_o^2 - F_c^2)^2] / \Sigma [w (F_o^2)^2]]^{1/2}$

Goodness-of-fit = $[\Sigma [w (F_o^2 - F_c^2)^2] / (n-p)]^{1/2}$

n: number of independent reflections; p: number of refined parameters

Figure S10.

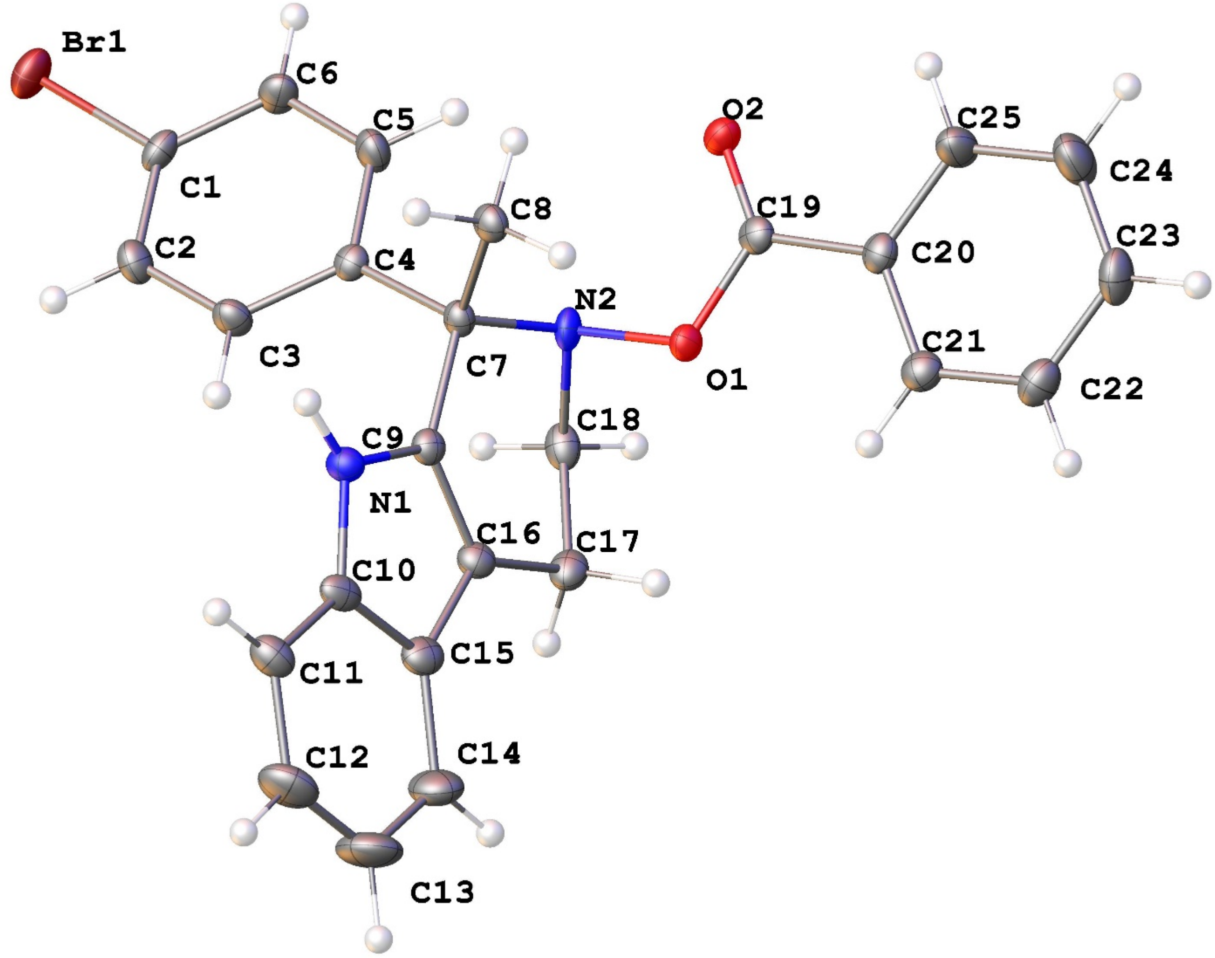

Br1
C6
C5
C1
C8
C2
C4
O2
C19
C25
C24
C23
C20
N2
C3
C7
O1
C21
C22
C18
C9
N1
C16
C17
C10
C11
C15
C12
C14
C13

Figure S11.

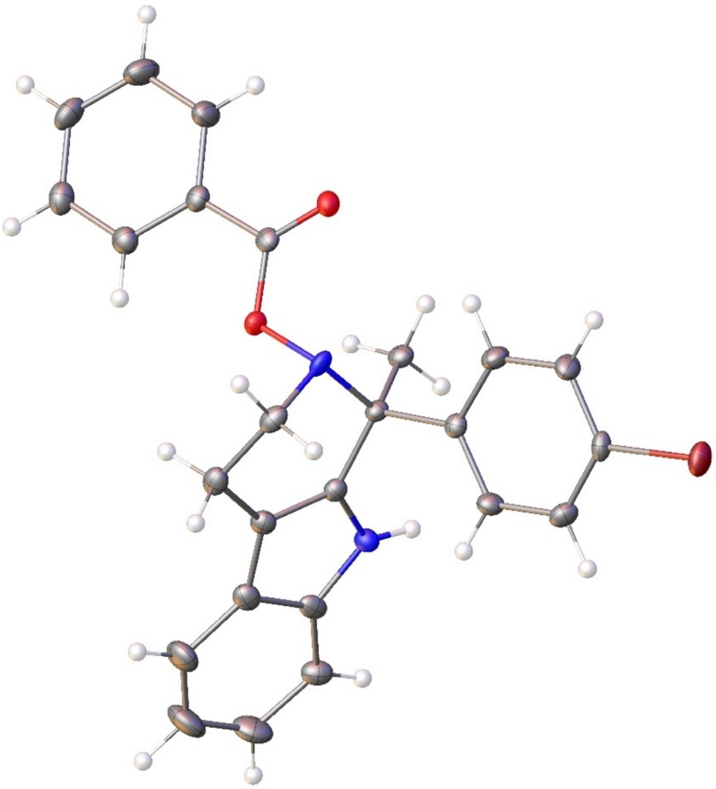

Figure S12.

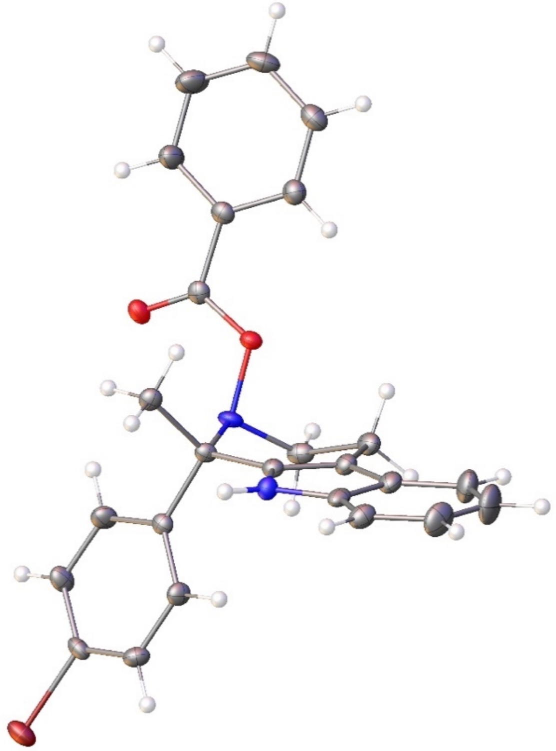

Figure S13.

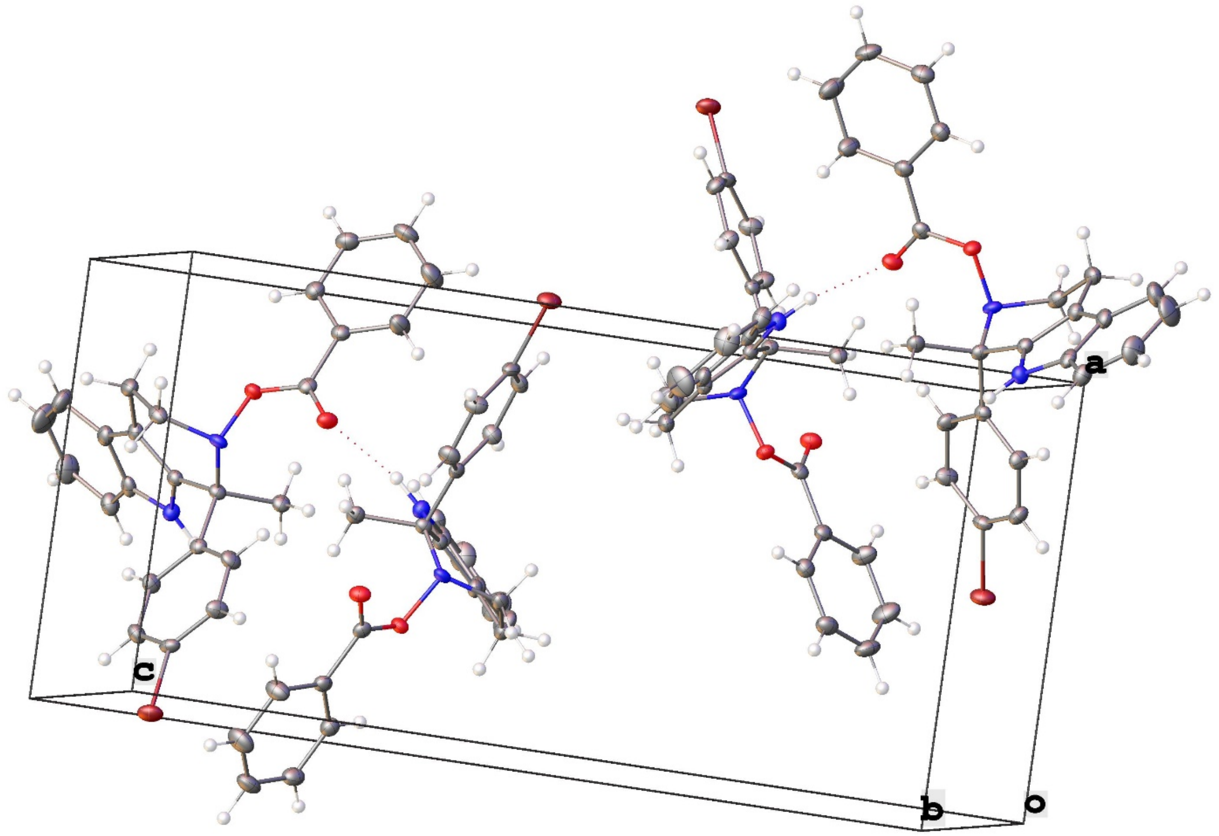

Table S16 Fractional Atomic Coordinates (×10^4) and Equivalent Isotropic Displacement Parameters (Å^2×10^3) for 0953_TLC_Snyder_2. U_{eq} is defined as 1/3 of of the trace of the orthogonalised U_{IJ} tensor.

Atom	*x*	*y*	*z*	U(eq)
Br1	10135.5(2)	962.6(3)	5898.3(2)	25.65(9)
N1	5321(2)	6189(2)	6408.1(10)	16.3(5)
N2	3748(2)	2879(2)	6445.2(11)	16.1(5)
O1	2509.8(17)	3232.6(17)	6796.2(9)	15.8(4)
O2	2994.1(19)	1567.4(16)	7467.1(9)	19.3(4)
C1	8525(3)	1865(3)	6112.8(13)	17.5(6)
C2	8302(3)	3080(3)	5866.3(14)	19.9(6)
C3	7099(3)	3706(3)	6019.2(13)	18.5(6)
C4	6147(3)	3135(3)	6417.0(13)	14.6(6)
C5	6404(3)	1909(3)	6656.8(14)	21.1(6)
C6	7585(3)	1268(3)	6509.1(14)	20.8(6)
C7	4833(3)	3820(2)	6620.3(11)	14.3(5)
C8	4871(3)	4029(2)	7360.6(12)	17.1(5)
C9	4605(3)	5080(2)	6273.3(12)	14.6(5)
C10	4844(3)	7161(2)	6009.2(11)	17.8(5)
C11	5234(3)	8443(2)	5973.3(13)	21.8(6)
C12	4554(4)	9215(3)	5537.5(15)	33.6(8)
C13	3527(4)	8717(3)	5148.3(17)	40.5(9)
C14	3148(3)	7443(3)	5177.3(15)	30.7(7)
C15	3812(3)	6636(3)	5617.8(13)	20.0(6)
C16	3692(3)	5305(3)	5794.9(13)	16.6(6)
C17	2776(3)	4269(3)	5545.8(14)	19.9(6)
C18	3353(3)	2969(3)	5754.7(13)	19.4(6)
C19	2245(3)	2418(2)	7293.2(13)	16.0(6)
C20	935(3)	2716(3)	7610.7(13)	17.4(6)
C21	37(3)	3631(2)	7366.6(13)	20.5(6)
C22	-1180(3)	3837(3)	7669.9(15)	24.1(6)
C23	-1520(3)	3148(3)	8225.5(16)	27.6(7)
C24	-631(3)	2260(3)	8475.7(16)	33.1(8)
C25	598(3)	2042(3)	8171.8(15)	26.0(7)

Table S17 Anisotropic Displacement Parameters (Å^2×10^3) for 0953_TLC_Snyder_2. The Anisotropic displacement factor exponent takes the form: $-2\pi^2[h^2a^{*2}U_{11}+2hka^*b^*U_{12}+\ldots]$.

Atom	U_{11}	U_{22}	U_{33}	U_{23}	U_{13}	U_{12}
Br1	14.75(13)	27.10(15)	35.09(16)	-3.25(13)	0.49(12)	4.61(12)
N1	18.1(11)	14.9(11)	15.9(11)	0.4(9)	-1.4(9)	-0.5(9)
N2	10.1(11)	19.2(12)	18.9(12)	-2.9(10)	4.4(9)	-1.4(9)
O1	13.0(9)	17.2(10)	17.2(9)	0.3(8)	2.8(7)	2.0(8)
O2	14.8(10)	17.4(10)	25.6(11)	3.5(8)	-0.7(8)	1.6(8)
C1	11.6(12)	20.1(14)	20.7(14)	-5.1(12)	-2.2(10)	3.1(11)
C2	19.7(13)	21.2(14)	18.7(14)	-0.4(13)	5.4(13)	-2.4(11)
C3	21.2(13)	13.7(13)	20.5(15)	2.7(11)	2.0(11)	0.4(10)
C4	14.1(13)	14.6(14)	15.1(14)	-2.6(12)	-1.6(11)	-1.4(11)
C5	18.7(14)	19.2(15)	25.5(16)	3.8(13)	3.9(12)	-3.6(12)
C6	18.9(14)	13.2(14)	30.4(16)	3.6(12)	0.1(12)	1.1(10)
C7	13.7(12)	12.3(12)	17.1(12)	0.0(9)	1.5(10)	-1.6(11)
C8	17.0(12)	15.3(11)	18.9(12)	0.7(11)	0.1(11)	-3.8(14)
C9	14.8(13)	15.5(13)	13.5(13)	-1.5(11)	1.5(10)	-0.3(10)
C10	22.2(13)	17.6(12)	13.7(12)	0.4(10)	5.9(12)	5.9(11)
C11	28.1(14)	17.8(13)	19.4(14)	0.2(11)	3.4(14)	-1.1(11)
C12	48(2)	19.2(15)	33.6(17)	8.3(14)	1.1(15)	1.7(15)
C13	51(2)	33(2)	38(2)	16.9(16)	-11.2(18)	5.7(16)
C14	34.1(19)	34.7(19)	23.4(17)	8.3(14)	-9.1(14)	1.1(15)
C15	22.2(15)	23.6(16)	14.1(14)	-0.5(12)	1.8(12)	2.8(12)
C16	18.7(13)	18.1(13)	13.1(14)	-0.5(11)	0.7(11)	0.1(11)
C17	18.0(13)	26.4(17)	15.4(14)	-1.7(12)	-1.3(11)	-1.1(12)
C18	18.3(13)	21.3(15)	18.5(15)	-5.3(12)	2.3(11)	-5.6(11)
C19	15.3(13)	14.2(14)	18.7(14)	-1.1(11)	-2.5(11)	-3.5(11)
C20	14.8(14)	17.9(14)	19.7(14)	-2.0(12)	-1.1(11)	-2.1(11)
C21	19.9(14)	21.2(13)	20.4(13)	-0.8(10)	-2.2(13)	-0.7(12)
C22	18.7(14)	23.6(16)	30.2(16)	-3.8(14)	-1.4(12)	2.8(13)
C23	17.9(15)	29.5(17)	35.4(18)	-4.3(15)	9.3(13)	-1.5(13)
C24	30.5(18)	33.0(18)	35.7(19)	9.4(16)	13.5(15)	0.4(14)
C25	22.6(16)	26.1(16)	29.4(17)	9.0(14)	4.9(13)	5.4(13)

Table S18 Bond Lengths for 0953_TLC_Snyder_2.

Atom	Atom	Length/Å	Atom	Atom	Length/Å
Br1	C1	1.900(3)	C9	C16	1.356(4)
N1	C9	1.379(3)	C10	C11	1.388(4)

Table S18 Bond Lengths for 0953_TLC_Snyder_2.

Atom	Atom	Length/Å	Atom	Atom	Length/Å
N1	C10	1.383(3)	C10	C15	1.410(4)
N2	O1	1.468(3)	C11	C12	1.377(4)
N2	C7	1.496(3)	C12	C13	1.393(5)
N2	C18	1.475(4)	C13	C14	1.376(5)
O1	C19	1.352(3)	C14	C15	1.397(4)
O2	C19	1.207(3)	C15	C16	1.434(4)
C1	C2	1.378(4)	C16	C17	1.497(4)
C1	C6	1.383(4)	C17	C18	1.526(4)
C2	C3	1.391(4)	C19	C20	1.484(4)
C3	C4	1.381(4)	C20	C21	1.393(4)
C4	C5	1.387(4)	C20	C25	1.390(4)
C4	C7	1.540(4)	C21	C22	1.373(4)
C5	C6	1.379(4)	C22	C23	1.389(4)
C7	C8	1.538(3)	C23	C24	1.374(5)
C7	C9	1.506(3)	C24	C25	1.386(4)

Table S19 Bond Angles for 0953_TLC_Snyder_2.

Atom	Atom	Atom	Angle/°	Atom	Atom	Atom	Angle/°
C9	N1	C10	108.3(2)	N1	C10	C15	107.7(2)
O1	N2	C7	108.50(19)	C11	C10	C15	122.8(2)
O1	N2	C18	103.68(19)	C12	C11	C10	117.2(3)
C18	N2	C7	112.3(2)	C11	C12	C13	121.0(3)
C19	O1	N2	112.14(19)	C14	C13	C12	122.1(3)
C2	C1	Br1	120.0(2)	C13	C14	C15	118.4(3)
C2	C1	C6	121.3(2)	C10	C15	C16	106.7(2)
C6	C1	Br1	118.7(2)	C14	C15	C10	118.7(3)
C1	C2	C3	118.8(3)	C14	C15	C16	134.7(3)
C4	C3	C2	121.1(3)	C9	C16	C15	107.1(3)
C3	C4	C5	118.6(3)	C9	C16	C17	121.9(2)
C3	C4	C7	122.6(2)	C15	C16	C17	131.0(3)
C5	C4	C7	118.8(2)	C16	C17	C18	108.2(2)
C6	C5	C4	121.3(3)	N2	C18	C17	115.2(2)
C5	C6	C1	118.9(3)	O1	C19	C20	111.8(2)
N2	C7	C4	103.81(18)	O2	C19	O1	124.2(3)

Table S19 Bond Angles for 0953_TLC_Snyder_2.

Atom	Atom	Atom	Angle/°	Atom	Atom	Atom	Angle/°
N2	C7	C8	110.4(2)	O2	C19	C20	124.0(3)
N2	C7	C9	110.2(2)	C21	C20	C19	122.7(3)
C8	C7	C4	108.2(2)	C25	C20	C19	118.0(2)
C9	C7	C4	113.5(2)	C25	C20	C21	119.3(3)
C9	C7	C8	110.5(2)	C22	C21	C20	120.1(3)
N1	C9	C7	123.5(2)	C21	C22	C23	120.4(3)
C16	C9	N1	110.2(2)	C24	C23	C22	119.9(3)
C16	C9	C7	126.3(2)	C23	C24	C25	120.2(3)
N1	C10	C11	129.5(3)	C24	C25	C20	120.2(3)

Crystal Structure of ±63a

General information: The diffraction data were measured at 100 K on a Bruker D8 VENTURE diffractometer equipped with a microfocus Mo-target X-ray tube (λ = 0.71073 Å) and PHOTON 100 CMOS detector. Data were collected using ϕ and ω scans to survey a hemisphere of reciprocal space. Data reduction and integration were performed with the Bruker APEX3 software package (Bruker AXS, version 2017.3-0, 2018). Data were scaled and corrected for absorption effects using the multi-scan procedure as implemented in SADABS (Bruker AXS, version 2014/5, Krause, Herbst-Irmer, Sheldrick & Stalke, *J. Appl. Cryst.* **2015**, *48*, 3-10). The structure was solved by SHELXT (Version 2014/5: Sheldrick, G. M. *Acta Crystallogr.* **2015**, *A71*, 3-8) and refined by a full-matrix least-squares procedure using OLEX2 (O. V. Dolomanov, L. J. Bourhis, R. J. Gildea, J. A. K. Howard and H. Puschmann. J. Appl. Crystallogr. **2009**, *42*, 339-341) (XL refinement program version 2018/3, *Sheldrick, G. M. Acta Crystallogr.* **2015**, *C71*, 3-8). Crystallographic data and details of the data collection and structure refinement are listed in Table S20.

Specific details for structure refinement: All atoms were refined with anisotropic thermal parameters. Hydrogen atoms were included in idealized positions for structure factor calculations except atom H1O and H1N attached to oxygen and nitrogen atoms, respectively. These hydrogen atom were located in the difference Fourier map. All structures are drawn with thermal ellipsoids at 50% probability.

Table S20 Crystal data and structure refinement for 0812_T.

Identification code	0812_T
Empirical formula	$C_{19}H_{20}N_2O$
Formula weight	292.37
Temperature/K	100(2)
Crystal system	triclinic
Space group	P-1
a/Å	8.5841(6)
b/Å	10.1481(7)
c/Å	10.2458(8)
α/°	112.745(2)
β/°	103.325(2)
γ/°	99.118(2)
Volume/Å^3	769.88(10)
Z	2
ρ_{calc}g/cm^3	1.261
μ/mm^{-1}	0.079
F(000)	312.0
Crystal size/mm^3	0.6 × 0.27 × 0.15
Radiation	MoKα (λ = 0.71073)
2Θ range for data collection/°	4.528 to 57.228
Index ranges	-11 ≤ h ≤ 11, -13 ≤ k ≤ 13, -13 ≤ l ≤ 13
Reflections collected	28347
Independent reflections	3937 [R_{int} = 0.0470, R_{sigma} = 0.0339]
Data/restraints/parameters	3937/2/209
Goodness-of-fit on F^2	1.031
Final R indexes [I>=2σ (I)]	R_1 = 0.0453, wR_2 = 0.1035
Final R indexes [all data]	R_1 = 0.0654, wR_2 = 0.1129
Largest diff. peak/hole / e Å^{-3}	0.39/-0.19

$R_{int} = \Sigma \mid F_o^2 - \langle F_o^2 \rangle \mid / \Sigma \mid F_o^2 \mid$

$R1 = \Sigma \mid \mid F_o \mid - \mid F_c \mid \mid / \Sigma \mid F_o \mid$

$wR2 = [\Sigma [w (F_o^2 - F_c^2)^2] / \Sigma [w (F_o^2)^2]]^{1/2}$

$\text{Goodness-of-fit} = [\Sigma [w (F_o^2 - F_c^2)^2] / (n-p)]^{1/2}$

n: number of independent reflections; p: number of refined parameters

Figure S14.

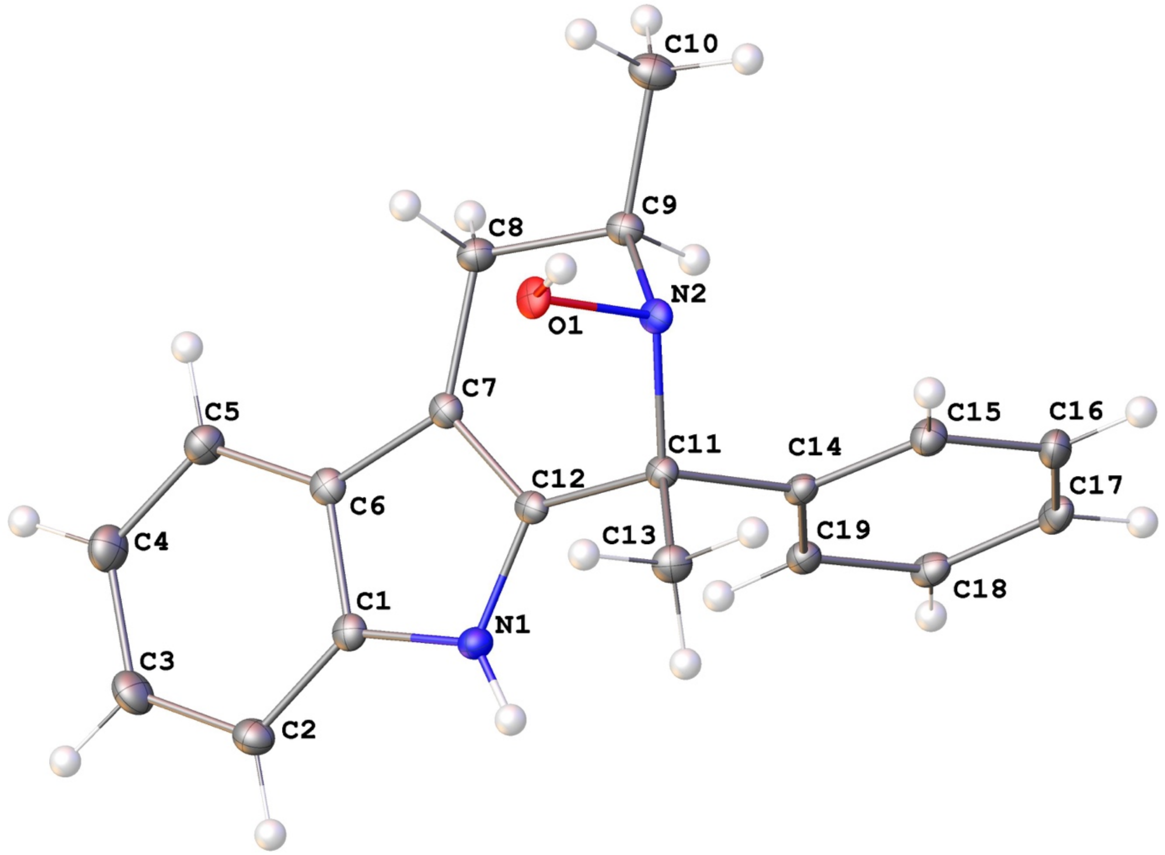

Figure S15.

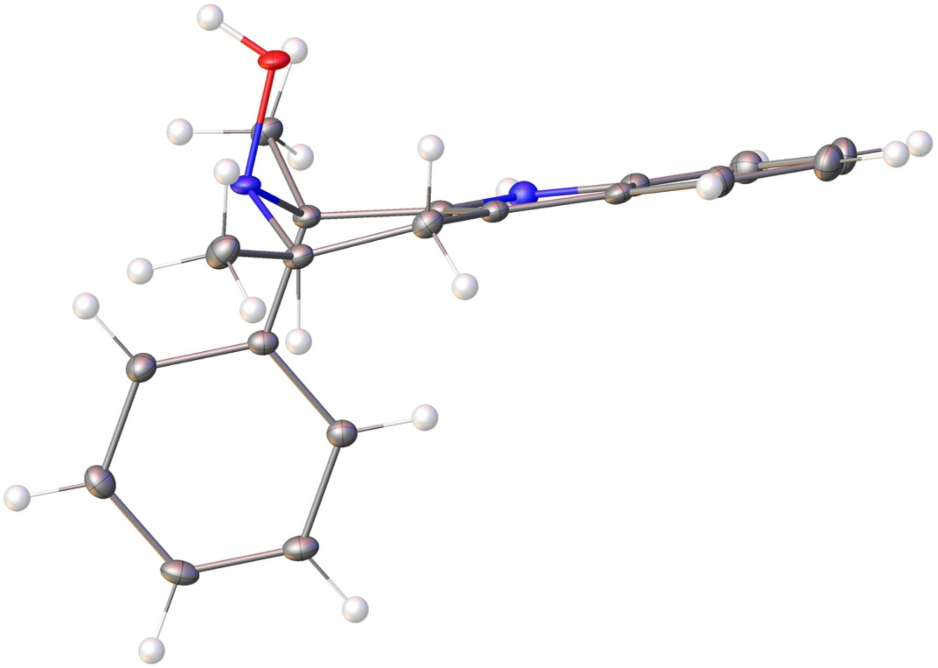

Figure S16.

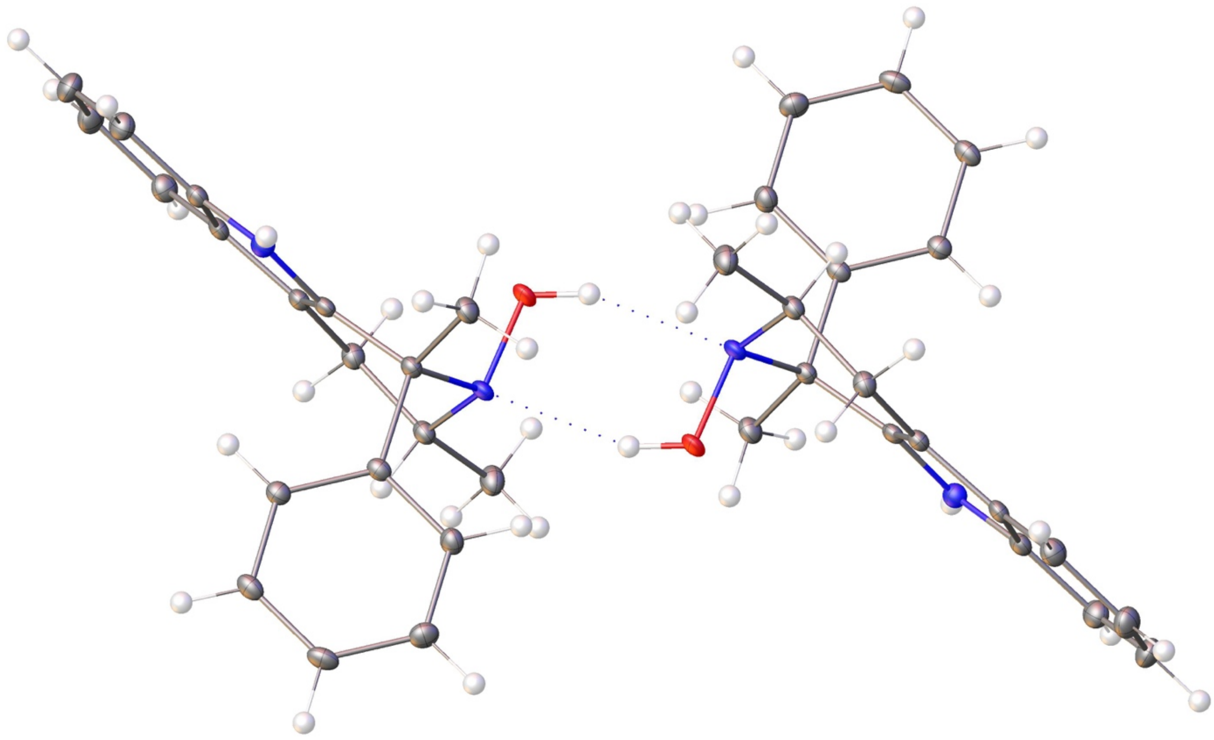

Figure S17.

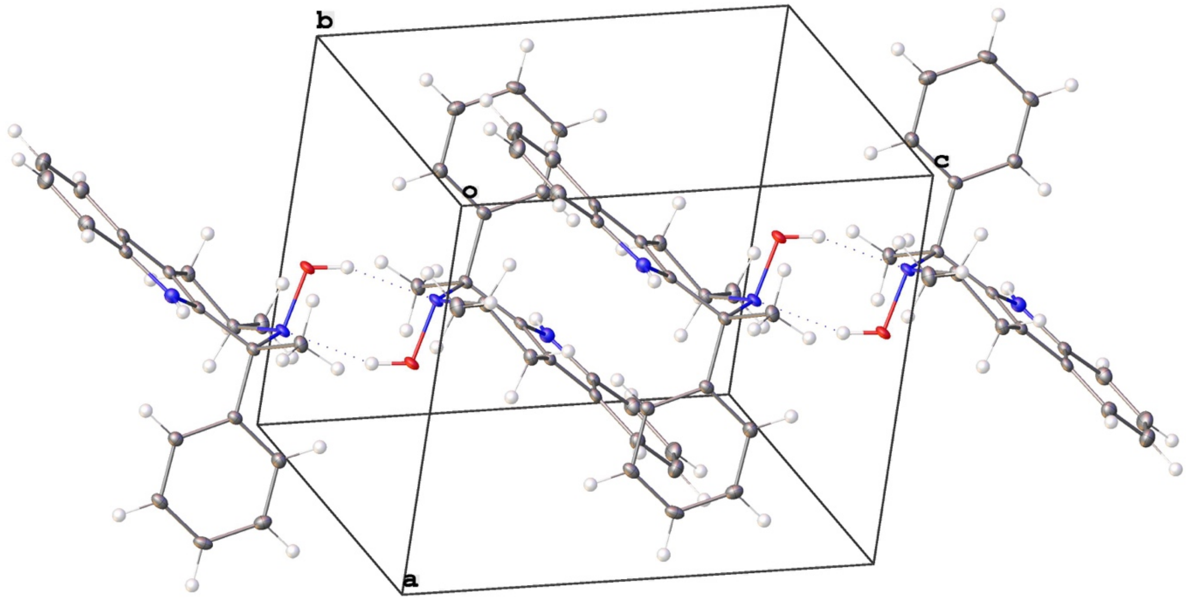

Table S21 Fractional Atomic Coordinates (×10^4) and Equivalent Isotropic Displacement Parameters (Å^2×10^3) for 0812_T. U_{eq} is defined as 1/3 of of the trace of the orthogonalised U_{IJ} tensor.

Atom	*x*	*y*	*z*	U(eq)
O1	3482.0(11)	4598.0(10)	8579.7(11)	15.7(2)
N1	2427.0(13)	1253.5(12)	4546.7(12)	14.2(2)
N2	5023.7(13)	4481.5(12)	8218.0(11)	13.0(2)
C1	1299.0(16)	1461.5(14)	3489.4(14)	14.2(3)
C2	41.8(17)	430.7(15)	2182.7(15)	18.9(3)
C3	-937.6(18)	980.5(16)	1361.2(15)	22.4(3)
C4	-674.6(18)	2510.7(16)	1811.9(15)	21.9(3)
C5	585.3(17)	3531.7(15)	3095.3(15)	18.2(3)
C6	1599.5(16)	3007.0(14)	3952.0(14)	13.9(3)
C7	2965.5(15)	3722.1(14)	5320.9(13)	13.2(3)
C8	3780.6(16)	5331.1(14)	6311.9(14)	15.3(3)
C9	5321.1(16)	5524.7(14)	7536.4(14)	14.2(3)
C10	6039.7(18)	7125.4(15)	8705.7(15)	20.0(3)
C11	4716.8(15)	2901.8(14)	7066.2(13)	12.6(2)
C12	3417.4(15)	2638.4(14)	5655.7(14)	12.5(2)
C13	4162.7(17)	1812.5(14)	7672.4(14)	16.4(3)
C14	6415.5(15)	2782.5(13)	6858.4(14)	12.8(3)
C15	7819.1(16)	3253.7(15)	8106.9(14)	16.3(3)
C16	9374.2(16)	3186.7(15)	7942.9(15)	18.0(3)
C17	9546.6(16)	2632.5(15)	6528.8(15)	17.6(3)
C18	8159.1(16)	2135.7(14)	5282.5(15)	16.1(3)
C19	6598.8(16)	2206.4(14)	5446.6(14)	14.1(3)

Table S22 Anisotropic Displacement Parameters (Å^2×10^3) for 0812_T. The Anisotropic displacement factor exponent takes the form: $-2\pi^2[h^2a^{*2}U_{11}+2hka^*b^*U_{12}+\ldots]$.

Atom	U_{11}	U_{22}	U_{33}	U_{23}	U_{13}	U_{12}
O1	13.2(4)	22.2(5)	15.2(5)	7.7(4)	10.1(4)	7.3(4)
N1	15.2(5)	12.8(5)	14.3(5)	5.1(4)	5.4(4)	4.5(4)
N2	11.4(5)	15.4(5)	13.5(5)	5.2(4)	8.1(4)	4.0(4)
C1	13.8(6)	17.7(6)	13.6(6)	7.0(5)	7.7(5)	5.6(5)
C2	20.2(7)	16.9(6)	16.5(6)	4.5(5)	6.2(5)	3.6(5)
C3	20.5(7)	24.9(7)	14.4(6)	5.1(6)	1.7(5)	2.6(6)
C4	22.9(7)	27.6(7)	17.1(7)	11.8(6)	5.0(6)	9.1(6)
C5	20.6(7)	19.2(7)	18.0(6)	10.2(5)	7.5(5)	6.6(5)
C6	14.3(6)	17.6(6)	12.4(6)	6.7(5)	8.4(5)	4.7(5)
C7	12.5(6)	15.9(6)	12.5(6)	6.0(5)	6.8(5)	3.7(5)

C8	18.2(6)	14.2(6)	14.3(6)	6.3(5)	6.9(5)	4.5(5)
C9	15.6(6)	14.3(6)	13.5(6)	5.4(5)	7.5(5)	3.4(5)
C10	24.8(7)	17.1(6)	15.3(6)	5.0(5)	7.3(6)	2.2(5)
C11	12.4(6)	13.7(6)	12.1(6)	4.9(5)	6.1(5)	3.9(5)
C12	11.5(6)	13.7(6)	12.9(6)	4.6(5)	7.5(5)	3.2(5)
C13	18.9(6)	17.4(6)	15.6(6)	8.7(5)	8.1(5)	4.5(5)
C14	13.8(6)	11.9(6)	15.4(6)	6.5(5)	7.3(5)	4.9(5)
C15	17.6(6)	18.0(6)	14.2(6)	6.3(5)	6.7(5)	6.8(5)
C16	14.7(6)	19.0(6)	20.7(7)	9.3(5)	4.0(5)	6.3(5)
C17	14.3(6)	17.5(6)	26.2(7)	10.9(6)	11.5(5)	7.4(5)
C18	18.6(6)	14.9(6)	19.1(6)	7.5(5)	11.7(5)	6.9(5)
C19	14.6(6)	13.5(6)	15.5(6)	6.8(5)	6.2(5)	4.3(5)

Table S23 Bond Lengths for 0812_T.

Atom	Atom	Length/Å	Atom	Atom	Length/Å
O1	N2	1.4644(13)	C7	C12	1.3577(18)
N1	C1	1.3833(17)	C8	C9	1.5243(18)
N1	C12	1.3893(16)	C9	C10	1.5137(18)
N2	C9	1.4973(16)	C11	C12	1.5059(17)
N2	C11	1.5139(16)	C11	C13	1.5279(17)
C1	C2	1.3945(18)	C11	C14	1.5380(17)
C1	C6	1.4113(18)	C14	C15	1.3967(18)
C2	C3	1.383(2)	C14	C19	1.3925(17)
C3	C4	1.401(2)	C15	C16	1.3922(18)
C4	C5	1.381(2)	C16	C17	1.3901(19)
C5	C6	1.4028(18)	C17	C18	1.3835(19)
C6	C7	1.4331(18)	C18	C19	1.3972(17)
C7	C8	1.4883(17)			

Table S24 Bond Angles for 0812_T.

Atom	Atom	Atom	Angle/°	Atom	Atom	Atom	Angle/°
C1	N1	C12	108.46(11)	N2	C9	C10	111.98(10)
O1	N2	C9	106.12(9)	C10	C9	C8	111.78(11)
O1	N2	C11	107.41(9)	N2	C11	C13	109.87(10)
C9	N2	C11	109.43(9)	N2	C11	C14	105.52(9)
N1	C1	C2	130.51(12)	C12	C11	N2	108.53(10)
N1	C1	C6	107.53(11)	C12	C11	C13	111.39(10)
C2	C1	C6	121.96(12)	C12	C11	C14	113.00(10)

C3	C2	C1	117.36(13)	C13	C11	C14	108.35(10)
C2	C3	C4	121.56(13)	N1	C12	C11	125.12(11)
C5	C4	C3	121.09(13)	C7	C12	N1	109.71(11)
C4	C5	C6	118.64(13)	C7	C12	C11	125.05(11)
C1	C6	C7	106.94(11)	C15	C14	C11	119.81(11)
C5	C6	C1	119.37(12)	C19	C14	C11	121.67(11)
C5	C6	C7	133.67(12)	C19	C14	C15	118.52(11)
C6	C7	C8	130.13(12)	C16	C15	C14	120.63(12)
C12	C7	C6	107.34(11)	C17	C16	C15	120.31(12)
C12	C7	C8	122.49(11)	C18	C17	C16	119.56(12)
C7	C8	C9	109.98(10)	C17	C18	C19	120.15(12)
N2	C9	C8	112.73(10)	C14	C19	C18	120.80(12)

Crystal Structure of ±64

General information: The diffraction data were measured at 100 K on a Bruker D8 VENTURE diffractometer equipped with a microfocus Mo-target X-ray tube (λ = 0.71073 Å) and PHOTON 100 CMOS detector. Data were collected using ϕ and ω scans to survey a hemisphere of reciprocal space. Data reduction and integration were performed with the Bruker APEX3 software package (Bruker AXS, version 2017.3-0, 2018). Data were scaled and corrected for absorption effects using the multi-scan procedure as implemented in SADABS (Bruker AXS, version 2014/5, Krause, Herbst-Irmer, Sheldrick & Stalke, *J. Appl. Cryst.* **2015**, *48*, 3-10). The structure was solved by SHELXT (Version 2014/5: Sheldrick, G. M. *Acta Crystallogr.* **2015**, *A**71***, 3-8) and refined by a full-matrix least-squares procedure using OLEX2 (O. V. Dolomanov, L. J. Bourhis, R. J. Gildea, J. A. K. Howard and H. Puschmann. J. Appl. Crystallogr. **2009**, *42*, 339-341) (XL refinement program version 2018/3, *Sheldrick, G. M. Acta Crystallogr.* **2015**, *C71*, 3-8). Crystallographic data and details of the data collection and structure refinement are listed in Table S25.

Specific details for structure refinement: All atoms were refined with anisotropic thermal parameters. Hydrogen atoms were included in idealized positions for structure factor calculations except atom H1O and H3N attached to oxygen and nitrogen atoms, respectively. These hydrogen atom were located in the difference Fourier map. All structures are drawn with thermal ellipsoids at 50% probability.

Table S25 Crystal data and structure refinement for 0814_t.

Identification code	0814_t
Empirical formula	$C_{19}H_{17}N_3O$
Formula weight	303.35
Temperature/K	100(2)
Crystal system	monoclinic
Space group	$P2_1/c$
a/Å	8.5899(4)
b/Å	18.3551(8)
c/Å	10.2741(5)
α/°	90
β/°	102.416(2)
γ/°	90
Volume/Å^3	1582.02(13)
Z	4
ρ_{calc}g/cm^3	1.274
μ/mm^{-1}	0.081
F(000)	640.0
Crystal size/mm^3	0.426 × 0.202 × 0.106
Radiation	MoKα (λ = 0.71073)
2Θ range for data collection/°	4.438 to 61.224
Index ranges	$-12 \leq h \leq 12$, $-26 \leq k \leq 25$, $-14 \leq l \leq 14$
Reflections collected	43653
Independent reflections	4749 [R_{int} = 0.0455, R_{sigma} = 0.0375]
Data/restraints/parameters	4749/2/217
Goodness-of-fit on F^2	1.044
Final R indexes [I>=2σ (I)]	R_1 = 0.0477, wR_2 = 0.1095
Final R indexes [all data]	R_1 = 0.0730, wR_2 = 0.1192
Largest diff. peak/hole / e Å^{-3}	0.39/-0.21

$R_{int} = \Sigma | F_o^2 - \langle F_o^2 \rangle | / \Sigma | F_o^2|$

$R1 = \Sigma || F_o| - | F_c|| / \Sigma| F_o|$

$wR2 = [\Sigma [w (F_o^2 - F_c^2)^2] / \Sigma [w (F_o^2)^2]]^{1/2}$

Goodness-of-fit $= [\Sigma [w (F_o^2 - F_c^2)^2] / (n-p)^{1/2}$

n: number of independent reflections; p: number of refined parameters

Figure S18.

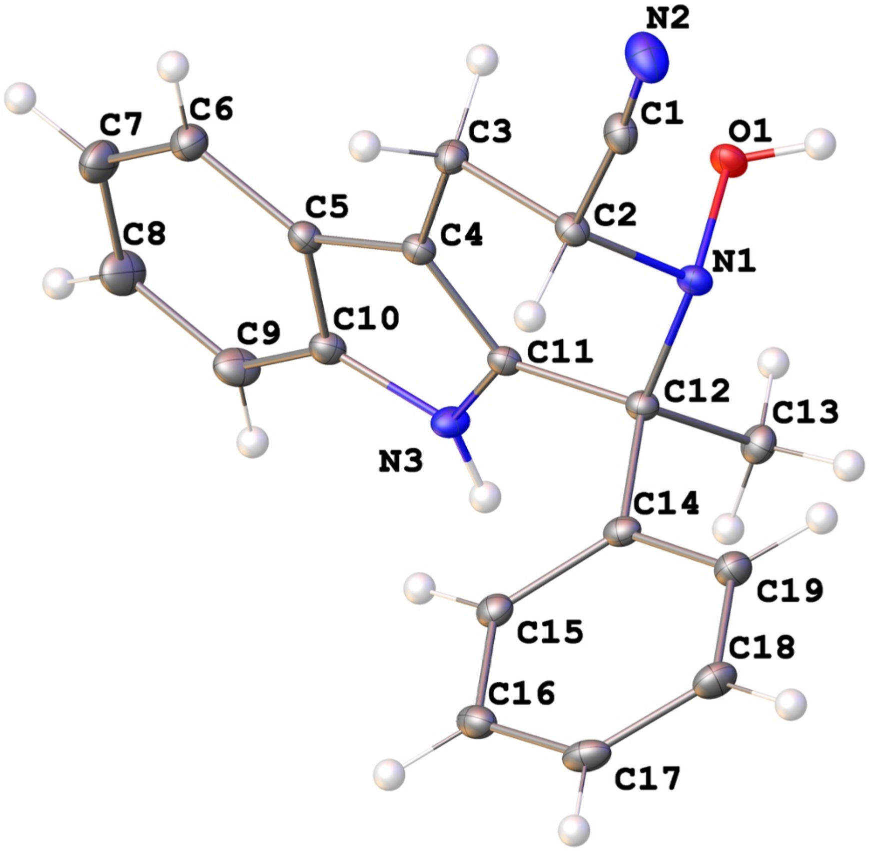

Figure S19.

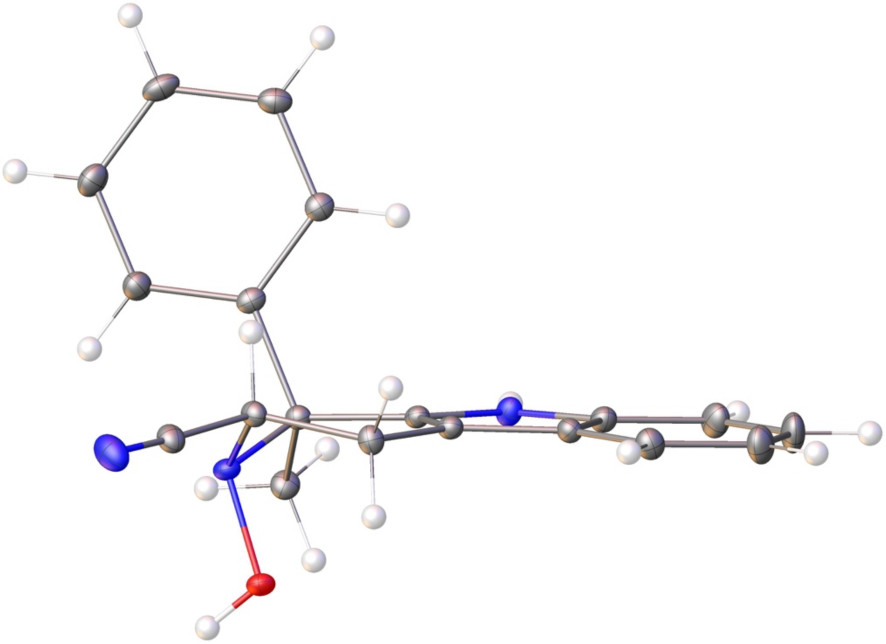

Figure S20.

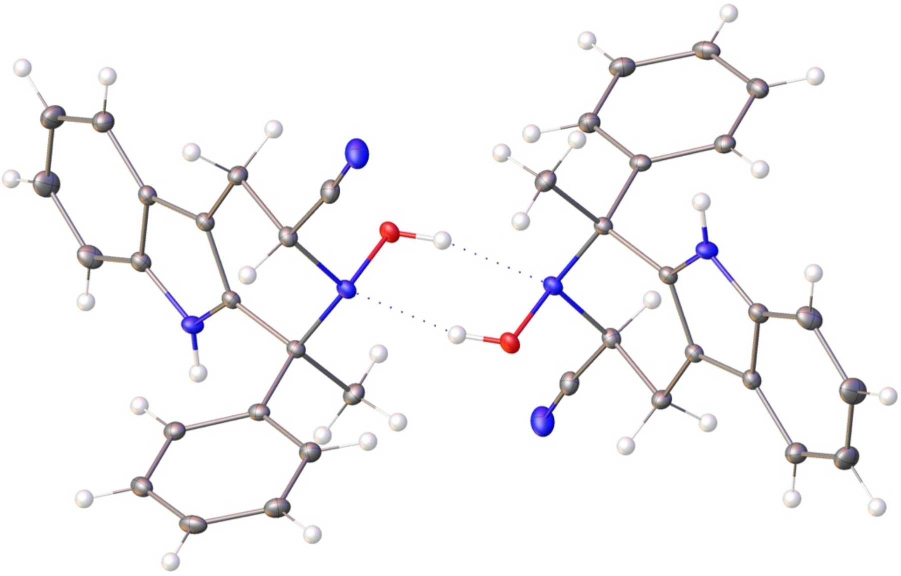

Figure S21.

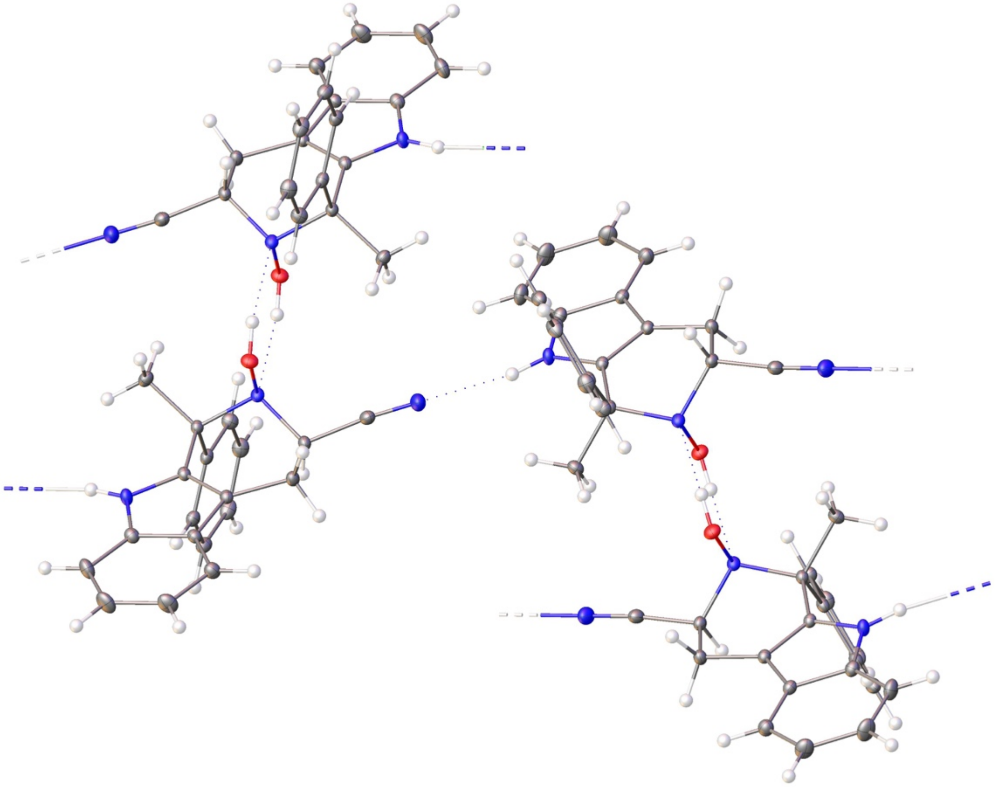

Figure S22.

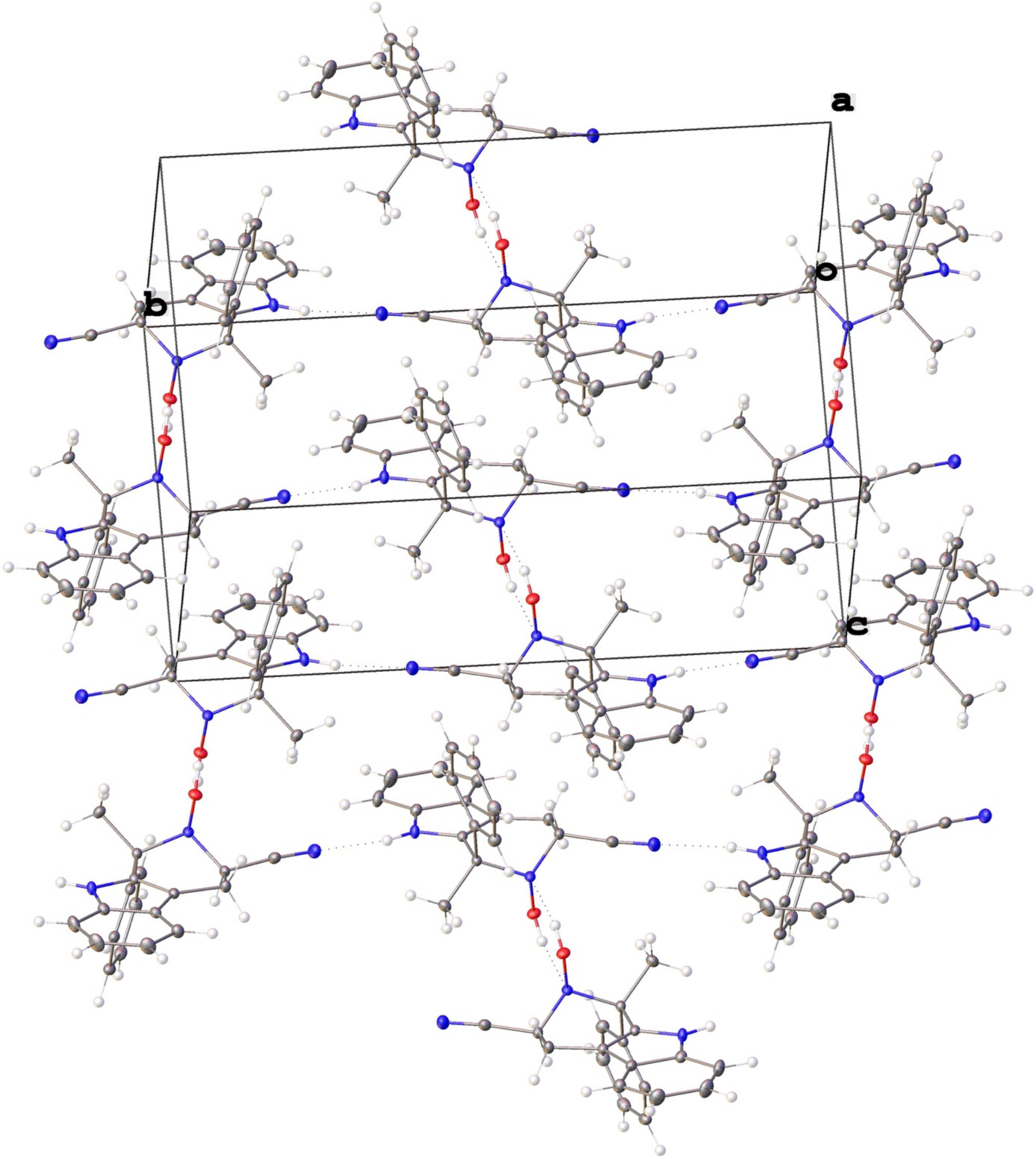

Table S26 Fractional Atomic Coordinates (×10⁴) and Equivalent Isotropic Displacement Parameters (Å²×10³) for 0814_t. U_{eq} is defined as 1/3 of of the trace of the orthogonalised U_{IJ} tensor.

Atom	*x*	*y*	*z*	U(eq)
O1	3599.9(9)	5129.1(5)	8755.2(8)	15.57(18)
N2	5571.5(14)	3344.1(6)	7926.7(11)	24.4(2)
N3	3569.0(11)	6790.8(5)	6266.7(10)	14.2(2)
N1	5172.6(11)	5211.0(5)	8459.6(9)	12.5(2)
C1	5404.5(14)	3954.9(6)	7714.2(11)	16.6(2)
C2	5174.5(14)	4722.0(6)	7306.2(11)	13.4(2)
C3	3690.7(14)	4794.3(6)	6177.1(11)	14.5(2)
C4	3388.3(13)	5584.7(6)	5913.6(11)	12.3(2)
C5	2264.8(13)	5933.3(6)	4868.7(11)	13.5(2)
C6	1124.9(14)	5680.1(7)	3773.6(12)	18.0(2)
C7	198.5(16)	6182.7(7)	2960.4(13)	23.6(3)
C8	375.2(16)	6932.2(7)	3215.3(13)	25.0(3)
C9	1478.7(15)	7197.1(7)	4293.9(13)	20.8(3)
C10	2418.4(13)	6690.1(6)	5116.5(11)	14.8(2)
C11	4141.9(13)	6117.2(6)	6739.3(11)	11.7(2)
C12	5350.6(13)	5991.4(6)	8018.1(11)	12.1(2)
C13	5091.2(14)	6509.4(6)	9114.8(12)	16.3(2)
C14	7075.7(13)	6058.0(6)	7850.9(11)	13.5(2)
C15	7435.7(14)	6342.4(6)	6693.1(12)	15.0(2)
C16	9005.9(14)	6380.5(6)	6543.4(12)	17.3(2)
C17	10236.3(14)	6137.9(7)	7560.0(13)	19.1(3)
C18	9894.0(14)	5872.4(7)	8725.4(12)	19.1(3)
C19	8327.8(14)	5833.2(7)	8876.5(12)	16.9(2)

Table S27 Anisotropic Displacement Parameters (Å²×10³) for 0814_t. The Anisotropic displacement factor exponent takes the form: $-2\pi^2[h^2a^{*2}U_{11}+2hka^*b^*U_{12}+\ldots]$.

Atom	U_{11}	U_{22}	U_{33}	U_{23}	U_{13}	U_{12}
O1	11.7(4)	19.5(4)	16.6(4)	1.9(3)	5.3(3)	-1.2(3)
N2	35.8(6)	16.6(5)	22.6(6)	2.3(4)	10.2(5)	5.5(5)
N3	14.1(4)	9.5(4)	18.0(5)	0.4(4)	1.7(4)	-0.4(4)
N1	11.2(4)	12.1(4)	15.4(5)	0.0(3)	5.4(4)	-0.1(3)
C1	19.8(6)	16.8(6)	13.8(5)	0.1(4)	4.9(4)	2.4(5)
C2	15.3(5)	10.9(5)	14.7(5)	1.4(4)	4.5(4)	1.4(4)
C3	17.0(5)	11.6(5)	14.0(5)	-0.5(4)	1.7(4)	-0.3(4)
C4	12.1(5)	11.5(5)	13.9(5)	0.6(4)	4.1(4)	0.0(4)
C5	13.4(5)	13.3(5)	14.1(5)	1.9(4)	3.7(4)	0.2(4)

C6	19.0(6)	16.9(6)	17.3(6)	0.3(4)	2.1(5)	-1.6(4)
C7	22.0(6)	25.3(7)	19.6(6)	3.5(5)	-4.0(5)	-2.2(5)
C8	22.5(6)	22.7(6)	25.9(7)	9.7(5)	-3.8(5)	2.4(5)
C9	20.1(6)	14.3(5)	27.0(6)	6.2(5)	2.4(5)	1.4(5)
C10	13.0(5)	14.5(5)	16.8(5)	2.0(4)	3.4(4)	-0.4(4)
C11	10.6(5)	11.0(5)	14.1(5)	1.0(4)	3.8(4)	0.0(4)
C12	12.4(5)	10.6(5)	13.6(5)	-0.2(4)	3.4(4)	-0.5(4)
C13	17.7(6)	16.2(5)	14.9(5)	-3.2(4)	3.5(4)	1.0(4)
C14	12.7(5)	11.4(5)	16.7(5)	-3.2(4)	3.7(4)	-1.3(4)
C15	15.2(5)	13.5(5)	16.0(5)	-3.0(4)	2.9(4)	-1.2(4)
C16	18.1(6)	16.4(5)	19.0(6)	-2.8(4)	7.8(5)	-3.4(4)
C17	12.3(5)	18.4(6)	27.7(6)	-6.0(5)	6.5(5)	-3.3(4)
C18	13.8(5)	19.2(6)	22.6(6)	-2.3(5)	-0.1(5)	-1.2(4)
C19	15.2(5)	18.1(5)	16.8(5)	0.0(4)	2.1(4)	-1.7(4)

Table S28 Bond Lengths for 0814_t.

Atom	Atom	Length/Å	Atom	Atom	Length/Å
O1	N1	1.4548(12)	C6	C7	1.3772(17)
N2	C1	1.1454(16)	C7	C8	1.4026(19)
N3	C10	1.3803(15)	C8	C9	1.3824(18)
N3	C11	1.3798(14)	C9	C10	1.3924(16)
N1	C2	1.4869(14)	C11	C12	1.5069(15)
N1	C12	1.5203(14)	C12	C13	1.5268(15)
C1	C2	1.4701(16)	C12	C14	1.5329(15)
C2	C3	1.5336(15)	C14	C15	1.3933(16)
C3	C4	1.4884(15)	C14	C19	1.3964(16)
C4	C5	1.4310(15)	C15	C16	1.3917(16)
C4	C11	1.3631(15)	C16	C17	1.3898(18)
C5	C6	1.4024(16)	C17	C18	1.3816(18)
C5	C10	1.4132(16)	C18	C19	1.3889(17)

Table S29 Bond Angles for 0814_t.

Atom	Atom	Atom	Angle/°	Atom	Atom	Atom	Angle/°
C11	N3	C10	108.47(9)	N3	C10	C9	130.17(11)
O1	N1	C2	105.32(8)	C9	C10	C5	121.88(11)
O1	N1	C12	108.48(8)	N3	C11	C12	124.81(10)
C2	N1	C12	108.28(8)	C4	C11	N3	109.74(10)
N2	C1	C2	174.53(13)	C4	C11	C12	125.37(10)

N1	C2	C3	114.05(9)	N1	C12	C13	109.06(9)
C1	C2	N1	111.96(9)	N1	C12	C14	105.67(8)
C1	C2	C3	109.72(9)	C11	C12	N1	108.05(8)
C4	C3	C2	107.83(9)	C11	C12	C13	111.44(9)
C5	C4	C3	129.45(10)	C11	C12	C14	113.05(9)
C11	C4	C3	123.01(10)	C13	C12	C14	109.33(9)
C11	C4	C5	107.44(10)	C15	C14	C12	121.52(10)
C6	C5	C4	134.07(11)	C15	C14	C19	118.59(11)
C6	C5	C10	119.49(10)	C19	C14	C12	119.88(10)
C10	C5	C4	106.41(10)	C16	C15	C14	120.76(11)
C7	C6	C5	118.51(11)	C17	C16	C15	119.95(11)
C6	C7	C8	121.24(12)	C18	C17	C16	119.68(11)
C9	C8	C7	121.51(12)	C17	C18	C19	120.45(11)
C8	C9	C10	117.36(12)	C18	C19	C14	120.52(11)
N3	C10	C5	107.93(10)				

Crystal Structure of ±65a

General information: The diffraction data were measured at 100 K on a Bruker D8 VENTURE diffractometer equipped with a microfocus Mo-target X-ray tube (λ = 0.71073 Å) and PHOTON 100 CMOS detector. Data were collected using ϕ and ω scans to survey a hemisphere of reciprocal space. Data reduction and integration were performed with the Bruker APEX3 software package (Bruker AXS, version 2017.3-0, 2018). Data were scaled and corrected for absorption effects using the multi-scan procedure as implemented in SADABS (Bruker AXS, version 2014/5, Krause, Herbst-Irmer, Sheldrick & Stalke, *J. Appl. Cryst.* **2015**, *48*, 3-10). The structure was solved by SHELXT (Version 2014/5: Sheldrick, G. M. *Acta Crystallogr.* **2015**, ***A71***, 3-8) and refined by a full-matrix least-squares procedure using OLEX2 (O. V. Dolomanov, L. J. Bourhis, R. J. Gildea, J. A. K. Howard and H. Puschmann. J. Appl. Crystallogr. **2009**, *42*, 339-341) (XL refinement program version 2018/3, *Sheldrick, G. M. Acta Crystallogr.* **2015**, *C71*, 3-8). Crystallographic data and details of the data collection and structure refinement are listed in Table S30.

Specific details for structure refinement: All atoms were refined with anisotropic thermal parameters. Hydrogen atoms were included in idealized positions for structure factor calculations except H-atoms attached to nitrogen atoms. These hydrogen atoms (H1 and H3) were located in the difference Fourier map and allowed to be refined at 0.88 Å within a default 0.02 Å standard deviation with their thermal parameters being constrained to be 1.2 times of the Ueq value of the N atoms. All structures are drawn with thermal ellipsoids at 50% probability.

Table S30 Crystal data and structure refinement for 0869_tessa_top.

Identification code	0869_tessa_top
Empirical formula	$C_{22}H_{22.3}N_2O_{3.15}$
Formula weight	365.12
Temperature/K	100(2)
Crystal system	monoclinic
Space group	$P2_1/c$
a/Å	20.9936(14)
b/Å	10.3069(7)
c/Å	17.3090(11)
α/°	90
β/°	99.457(2)
γ/°	90
Volume/Å^3	3694.4(4)
Z	8
ρ_{calc}g/cm^3	1.313
μ/mm^{-1}	0.088
F(000)	1548.0
Crystal size/mm^3	0.22 × 0.16 × 0.12
Radiation	MoKα (λ = 0.71073)
2Θ range for data collection/°	4.414 to 52.836
Index ranges	$-26 \le h \le 26$, $-12 \le k \le 12$, $-21 \le l \le 20$
Reflections collected	96894
Independent reflections	7298 [R_{int} = 0.0519, R_{sigma} = 0.0343]
Data/restraints/parameters	7298/2/504
Goodness-of-fit on F^2	1.053
Final R indexes [I>=2σ (I)]	R_1 = 0.0464, wR_2 = 0.0956
Final R indexes [all data]	R_1 = 0.0758, wR_2 = 0.1049
Largest diff. peak/hole / e Å^{-3}	0.30/-0.37

$R_{int} = \Sigma \mid F_o^2 - \langle F_o^2 \rangle \mid / \Sigma \mid F_o^2 \mid$

$R1 = \Sigma \mid \mid F_o \mid - \mid F_c \mid \mid / \Sigma \mid F_o \mid$

$wR2 = [\Sigma [w (F_o^2 - F_c^2)^2] / \Sigma [w (F_o^2)^2]]^{1/2}$

Goodness-of-fit = $[\Sigma [w (F_o^2 - F_c^2)^2] / (n-p)]^{1/2}$

n: number of independent reflections; p: number of refined parameters

Figure S23.

Independent unit

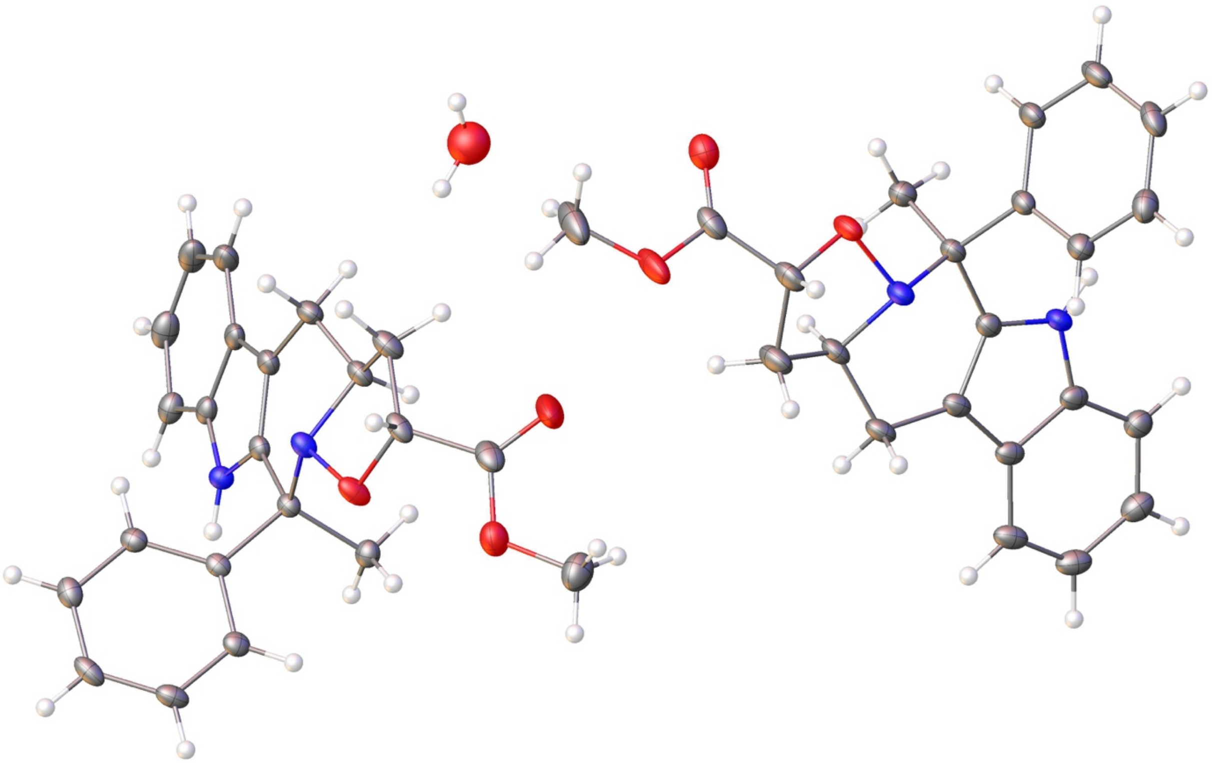

Figure S24.

Molecule 1

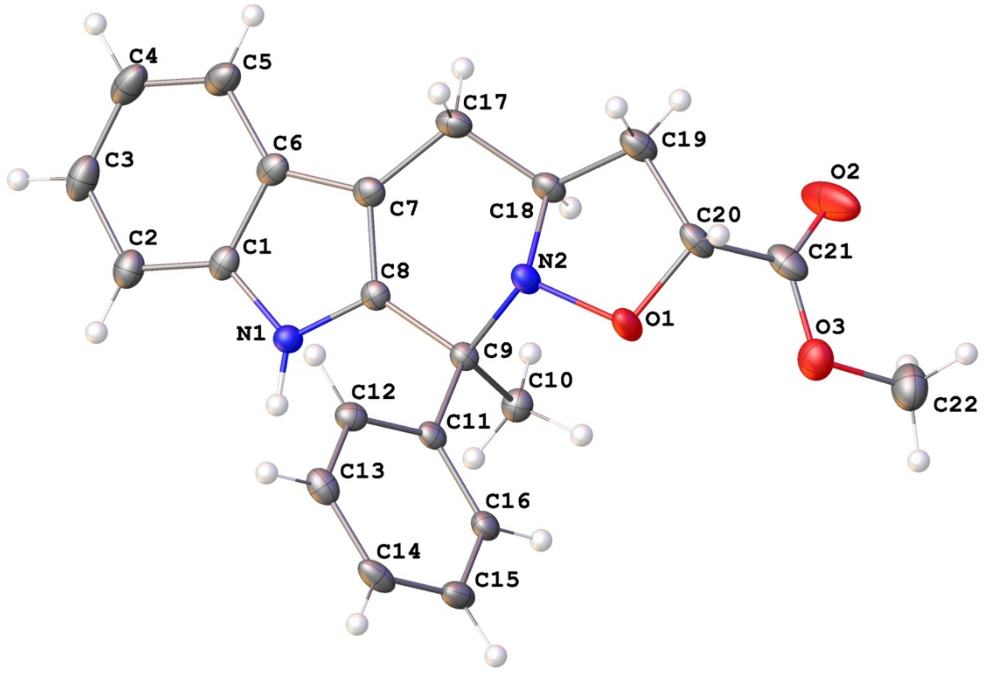

Figure S25.

Molecule 2 (different -CO_2Me group orientation)

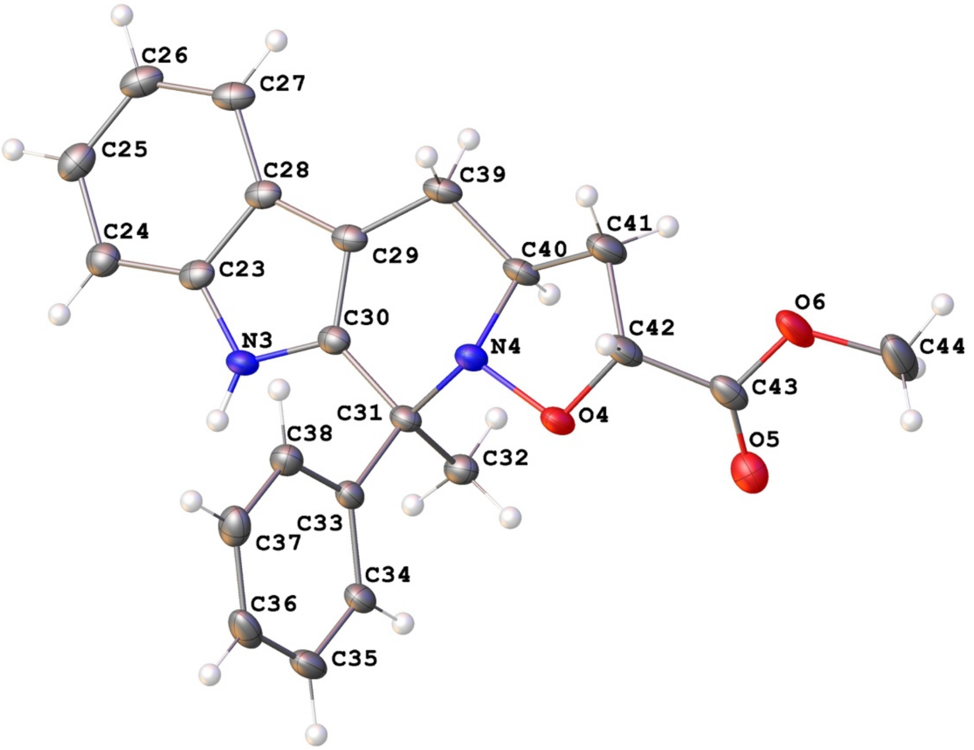

Figure S26.

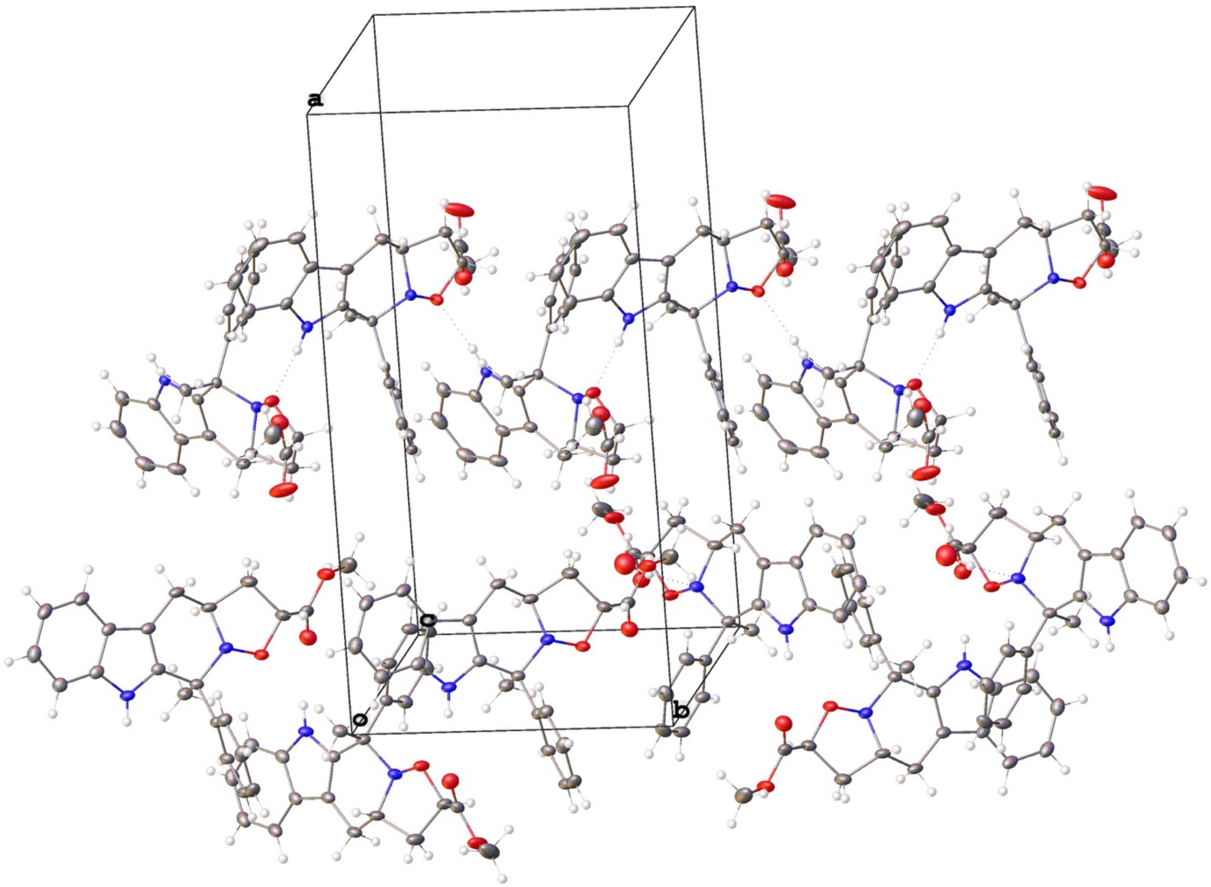

Table S31 Fractional Atomic Coordinates (×10^4) and Equivalent Isotropic Displacement Parameters (Å^2×10^3) for 0869_tessa_top. U$_{eq}$ is defined as 1/3 of of the trace of the orthogonalised U$_{IJ}$ tensor.

Atom	*x*	*y*	*z*	U(eq)
O1	4337.1(5)	6722.6(12)	6738.1(7)	28.5(3)
O2	3155.1(7)	7245(2)	5146.6(9)	70.2(6)
O3	4228.1(6)	7259.4(14)	5250.0(7)	37.3(3)
O7	1576(3)	7332(6)	6696(4)	63.1(15)
N1	4440.7(7)	3045.1(13)	8518.1(8)	19.9(3)
N2	4142.7(6)	6084.4(13)	7428.5(8)	21.6(3)
C1	3990.4(8)	2551.1(17)	8934.5(9)	22.2(4)
C2	4021.3(9)	1469.1(18)	9420.4(10)	27.9(4)
C3	3478.6(10)	1196(2)	9753.9(11)	34.9(5)
C4	2930.2(9)	1984(2)	9623.7(11)	36.8(5)
C5	2906.4(8)	3065(2)	9154.4(10)	31.6(4)
C6	3438.9(8)	3355.1(17)	8789.4(9)	23.6(4)
C7	3572.5(8)	4347.7(17)	8260.4(10)	23.0(4)
C8	4180.8(7)	4119.6(16)	8108.9(9)	19.2(4)
C9	4529.0(7)	4862.9(15)	7556.7(9)	18.6(3)
C10	4539.4(8)	4043.3(17)	6817.2(10)	23.6(4)
C11	5209.7(7)	5261.4(15)	7948.9(9)	18.2(3)
C12	5340.0(8)	5518.9(16)	8747.8(10)	21.9(4)
C13	5948.9(8)	5902.0(16)	9105.5(10)	25.8(4)
C14	6437.9(8)	6053.8(16)	8665.0(11)	27.2(4)
C15	6315.1(8)	5825.3(17)	7873.0(11)	26.5(4)
C16	5706.3(8)	5418.8(16)	7516.3(10)	22.3(4)
C17	3159.4(8)	5431.3(18)	7881.7(10)	25.9(4)
C18	3442.2(8)	5866.2(17)	7176.4(10)	23.6(4)
C19	3238.5(8)	7173.5(18)	6822.1(11)	29.5(4)
C20	3798.1(8)	7528.9(17)	6407.2(10)	25.6(4)
C21	3676.0(9)	7320.6(18)	5536.7(11)	33.4(5)
C22	4170.8(11)	7209(2)	4404.9(11)	43.7(5)
O4	831.1(5)	6653.7(12)	3115.2(7)	30.5(3)
O5	874.9(7)	7831.6(14)	4476.7(8)	39.2(3)
O6	1885.8(6)	8534.1(15)	4470.3(9)	48.2(4)
N3	484.1(7)	2774.4(15)	1500.5(8)	24.9(3)
N4	1039.0(6)	5696.3(14)	2580.0(8)	25.7(3)
C23	915.0(8)	1928.5(18)	1249.7(10)	25.2(4)
C24	805.0(9)	874.3(18)	744.2(10)	29.0(4)
C25	1334.9(9)	176.9(19)	598.8(11)	32.7(4)
C26	1960.7(9)	527.6(18)	947.7(11)	32.0(4)
C27	2071.7(8)	1587.0(18)	1437.9(11)	29.3(4)

C28	1544.2(8)	2319.2(17)	1598.2(10)	24.1(4)
C29	1468.4(8)	3444.9(17)	2058.7(10)	24.2(4)
C30	820.6(8)	3680.7(17)	1984.8(10)	23.3(4)
C31	500.1(8)	4748.0(17)	2379.8(10)	23.3(4)
C32	238.8(8)	4202.5(19)	3089.3(10)	28.0(4)
C33	-27.3(8)	5412.1(17)	1796.8(10)	23.1(4)
C34	-646.1(8)	5616.5(18)	1957.6(11)	27.6(4)
C35	-1108.0(9)	6225(2)	1412.9(12)	35.0(5)
C36	-962.9(9)	6626.9(19)	704.6(11)	36.2(5)
C37	-347.8(9)	6427.7(19)	538.5(11)	34.0(5)
C38	114.6(9)	5827.9(18)	1082.1(10)	29.0(4)
C39	1961.3(8)	4296.3(18)	2539.7(11)	27.8(4)
C40	1622.8(8)	5143.9(18)	3065.8(11)	28.6(4)
C41	1969.0(9)	6365(2)	3400.4(13)	39.3(5)
C42	1413.6(8)	7352.3(19)	3385.2(12)	32.8(4)
C43	1346.2(9)	7919.2(18)	4168.5(12)	32.8(5)
C44	1896.4(11)	9055(2)	5246.7(14)	57.8(7)

Table S32 Anisotropic Displacement Parameters (Å^2×10^3) for 0869_tessa_top. The Anisotropic displacement factor exponent takes the form: -2π^2[h^{2}a*^{2}U$_{11}$+2hka*b*U$_{12}$+…].

Atom	U$_{11}$	U$_{22}$	U$_{33}$	U$_{23}$	U$_{13}$	U$_{12}$
O1	19.5(6)	27.9(7)	36.5(7)	14.4(6)	0.1(5)	0.6(5)
O2	36.6(9)	123.9(17)	42.9(9)	-27.6(10)	-14.7(7)	28.2(10)
O3	35.8(8)	44.0(8)	30.4(7)	2.8(6)	0.1(6)	-3.4(6)
N1	18.3(7)	19.2(7)	22.2(7)	0.4(6)	3.3(6)	-0.4(6)
N2	18.2(7)	19.5(7)	25.8(8)	3.7(6)	0.1(6)	-0.6(6)
C1	24.0(9)	24.3(9)	17.7(8)	-3.3(7)	2.0(7)	-7.7(7)
C2	34.2(10)	27.6(10)	21.9(9)	-0.8(8)	4.3(8)	-5.6(8)
C3	45.0(12)	35.2(11)	24.9(10)	1.5(9)	6.9(8)	-15.5(10)
C4	31.8(11)	54.2(13)	25.2(10)	-0.1(10)	7.2(8)	-18.1(10)
C5	20.7(9)	48.6(12)	25.1(9)	-1.3(9)	3.0(7)	-7.0(9)
C6	20.4(9)	31.6(10)	17.9(8)	-4.4(7)	0.2(7)	-6.2(8)
C7	18.2(9)	28.1(9)	21.3(9)	-2.8(8)	-0.3(7)	-2.4(7)
C8	18.6(8)	19.2(8)	18.7(8)	-2.4(7)	-0.3(6)	-2.7(7)
C9	18.2(8)	15.9(8)	21.0(8)	-0.5(7)	1.4(6)	-0.6(7)
C10	25.6(9)	22.1(9)	22.6(9)	-1.6(7)	2.7(7)	-3.3(7)
C11	18.0(8)	12.0(8)	23.8(9)	1.9(7)	1.2(7)	1.3(7)
C12	20.6(9)	18.1(8)	26.9(9)	-0.7(7)	3.8(7)	0.8(7)
C13	28.3(10)	18.8(9)	27.7(9)	-1.0(8)	-3.4(7)	0.0(8)
C14	17.5(9)	19.7(9)	40.9(11)	3.1(8)	-5.9(8)	-0.6(7)

C15	17.5(9)	24.9(9)	37.7(11)	8.2(8)	5.8(7)	2.3(7)
C16	21.0(9)	20.4(9)	24.9(9)	4.7(7)	2.3(7)	4.0(7)
C17	17.0(8)	30.6(10)	29.2(9)	-2.9(8)	1.0(7)	1.5(8)
C18	16.4(8)	24.1(9)	28.2(9)	-0.4(8)	-2.4(7)	-0.5(7)
C19	19.8(9)	26.5(10)	39.7(11)	3.3(8)	-2.7(8)	1.3(8)
C20	22.0(9)	17.7(9)	33.4(10)	2.0(8)	-6.6(7)	2.1(7)
C21	30.6(11)	27.3(10)	38.7(11)	-4.6(9)	-5.3(9)	9.7(8)
C22	56.0(14)	42.6(13)	31.8(11)	2.3(10)	4.8(10)	5.0(11)
O4	16.5(6)	34.1(7)	38.8(7)	-9.6(6)	-1.7(5)	0.8(5)
O5	37.7(8)	41.6(8)	35.7(8)	-1.1(7)	-1.4(6)	-2.6(7)
O6	25.9(7)	48.9(9)	61.9(10)	-22.7(8)	-15.8(7)	4.2(7)
N3	15.0(7)	32.8(9)	26.6(8)	-1.5(7)	2.1(6)	2.5(7)
N4	17.5(7)	28.8(8)	29.9(8)	-3.0(7)	0.7(6)	3.2(6)
C23	24.5(9)	30.1(10)	22.5(9)	6.8(8)	8.2(7)	4.2(8)
C24	29.4(10)	32.0(10)	26.2(9)	0.9(8)	6.5(8)	0.7(8)
C25	40.8(11)	28.6(10)	31.3(10)	1.1(8)	13.6(9)	3.3(9)
C26	31.7(10)	29.6(10)	38.1(11)	7.1(9)	15.6(8)	7.5(9)
C27	22.2(9)	30.9(10)	36.5(10)	11.0(9)	10.1(8)	4.8(8)
C28	22.0(9)	27.1(10)	24.4(9)	8.3(8)	7.8(7)	4.1(8)
C29	18.3(9)	28.7(10)	25.9(9)	6.8(8)	4.7(7)	1.6(7)
C30	20.0(9)	27.9(9)	21.9(9)	3.7(8)	3.6(7)	0.9(8)
C31	15.6(8)	28.7(9)	24.9(9)	0.0(8)	1.6(7)	-0.9(7)
C32	22.4(9)	33.4(10)	28.2(10)	0.4(8)	3.9(7)	2.0(8)
C33	18.6(9)	24.2(9)	25.0(9)	-4.4(8)	-0.6(7)	1.4(7)
C34	18.5(9)	32.3(10)	30.5(10)	-6.0(8)	-0.3(7)	-1.6(8)
C35	18.2(9)	40.1(12)	43.9(12)	-9.5(10)	-3.3(8)	3.1(8)
C36	32.4(11)	34.2(11)	35.7(11)	-5.1(9)	-13.0(9)	8.6(9)
C37	41.0(11)	33.7(11)	25.1(10)	-0.9(9)	-1.1(8)	5.8(9)
C38	25.2(9)	32.9(10)	28.0(10)	-2.5(8)	1.8(7)	4.4(8)
C39	15.5(8)	33.4(10)	33.9(10)	6.2(8)	2.1(7)	2.9(8)
C40	14.7(8)	35.7(11)	33.1(10)	1.8(9)	-2.7(7)	1.7(8)
C41	19.4(9)	44.4(12)	51.2(13)	-9.7(10)	-3.1(9)	0.3(9)
C42	18.3(9)	34.0(11)	42.9(11)	-3.5(9)	-4.1(8)	-3.2(8)
C43	23.9(10)	28.1(10)	41.7(11)	0.7(9)	-9.0(9)	2.7(8)
C44	45.9(13)	52.9(15)	63.1(15)	-25.5(13)	-25.0(11)	12.7(12)

Table S33 Bond Lengths for 0869_tessa_top.

Atom	Atom	Length/Å	Atom	Atom	Length/Å
O1	N2	1.4791(18)	O4	N4	1.4682(19)
O1	C20	1.444(2)	O4	C42	1.430(2)

O2	C21	1.190(2)	O5	C43	1.202(2)
O3	C21	1.335(2)	O6	C43	1.328(2)
O3	C22	1.449(2)	O6	C44	1.444(3)
N1	C1	1.377(2)	N3	C23	1.377(2)
N1	C8	1.378(2)	N3	C30	1.371(2)
N2	C9	1.494(2)	N4	C31	1.492(2)
N2	C18	1.481(2)	N4	C40	1.481(2)
C1	C2	1.392(2)	C23	C24	1.390(3)
C1	C6	1.412(2)	C23	C28	1.417(2)
C2	C3	1.388(3)	C24	C25	1.382(3)
C3	C4	1.397(3)	C25	C26	1.400(3)
C4	C5	1.375(3)	C26	C27	1.379(3)
C5	C6	1.404(2)	C27	C28	1.405(2)
C6	C7	1.431(2)	C28	C29	1.431(3)
C7	C8	1.366(2)	C29	C30	1.367(2)
C7	C17	1.497(2)	C29	C39	1.500(2)
C8	C9	1.505(2)	C30	C31	1.510(2)
C9	C10	1.537(2)	C31	C32	1.532(2)
C9	C11	1.534(2)	C31	C33	1.532(2)
C11	C12	1.390(2)	C33	C34	1.389(2)
C11	C16	1.389(2)	C33	C38	1.387(2)
C12	C13	1.383(2)	C34	C35	1.387(3)
C13	C14	1.384(3)	C35	C36	1.375(3)
C14	C15	1.373(3)	C36	C37	1.384(3)
C15	C16	1.390(2)	C37	C38	1.382(3)
C17	C18	1.511(2)	C39	C40	1.520(3)
C18	C19	1.513(2)	C40	C41	1.520(3)
C19	C20	1.519(3)	C41	C42	1.545(3)
C20	C21	1.502(3)	C42	C43	1.504(3)

Table S34 Bond Angles for 0869_tessa_top.

Atom	Atom	Atom	Angle/°	Atom	Atom	Atom	Angle/°
C20	O1	N2	105.91(12)	C42	O4	N4	102.58(12)
C21	O3	C22	116.35(15)	C43	O6	C44	115.17(18)
C1	N1	C8	108.55(14)	C30	N3	C23	108.97(14)
O1	N2	C9	106.24(12)	O4	N4	C31	107.10(12)
O1	N2	C18	102.81(11)	O4	N4	C40	101.77(12)
C18	N2	C9	113.85(13)	C40	N4	C31	113.89(14)
N1	C1	C2	130.14(16)	N3	C23	C24	130.05(16)
N1	C1	C6	107.66(14)	N3	C23	C28	107.48(15)

C2	C1	C6	122.21(16)	C24	C23	C28	122.46(16)
C3	C2	C1	117.00(18)	C25	C24	C23	117.76(17)
C2	C3	C4	121.72(18)	C24	C25	C26	120.90(18)
C5	C4	C3	121.04(17)	C27	C26	C25	121.44(17)
C4	C5	C6	118.97(18)	C26	C27	C28	119.20(17)
C1	C6	C7	106.98(14)	C23	C28	C29	106.64(15)
C5	C6	C1	119.02(16)	C27	C28	C23	118.23(17)
C5	C6	C7	134.00(17)	C27	C28	C29	135.13(17)
C6	C7	C17	130.77(15)	C28	C29	C39	130.82(15)
C8	C7	C6	106.71(15)	C30	C29	C28	106.97(15)
C8	C7	C17	122.47(15)	C30	C29	C39	122.20(16)
N1	C8	C9	122.89(14)	N3	C30	C31	123.36(14)
C7	C8	N1	110.09(14)	C29	C30	N3	109.93(15)
C7	C8	C9	126.97(15)	C29	C30	C31	126.70(16)
N2	C9	C8	102.41(12)	N4	C31	C30	101.83(13)
N2	C9	C10	114.90(13)	N4	C31	C32	114.33(14)
N2	C9	C11	106.47(12)	N4	C31	C33	107.38(14)
C8	C9	C10	108.91(13)	C30	C31	C32	109.90(15)
C8	C9	C11	111.69(13)	C30	C31	C33	110.78(14)
C11	C9	C10	112.09(13)	C33	C31	C32	112.14(13)
C12	C11	C9	120.53(14)	C34	C33	C31	122.31(16)
C16	C11	C9	121.31(14)	C38	C33	C31	119.20(15)
C16	C11	C12	118.12(15)	C38	C33	C34	118.49(16)
C13	C12	C11	121.06(16)	C35	C34	C33	120.34(18)
C12	C13	C14	120.01(17)	C36	C35	C34	120.66(17)
C15	C14	C13	119.73(16)	C35	C36	C37	119.46(17)
C14	C15	C16	120.23(16)	C38	C37	C36	120.00(18)
C11	C16	C15	120.84(16)	C37	C38	C33	121.05(17)
C7	C17	C18	107.39(14)	C29	C39	C40	108.72(14)
N2	C18	C17	108.06(13)	N4	C40	C39	107.78(14)
N2	C18	C19	100.67(13)	N4	C40	C41	101.38(14)
C17	C18	C19	118.00(15)	C41	C40	C39	117.57(15)
C18	C19	C20	102.55(14)	C40	C41	C42	103.10(14)
O1	C20	C19	106.87(14)	O4	C42	C41	106.05(15)
O1	C20	C21	108.00(14)	O4	C42	C43	106.74(15)
C21	C20	C19	114.97(15)	C43	C42	C41	115.03(16)
O2	C21	O3	123.99(19)	O5	C43	O6	124.92(19)
O2	C21	C20	124.63(19)	O5	C43	C42	125.24(17)
O3	C21	C20	111.36(15)	O6	C43	C42	109.84(17)

Crystal Structure of ±65b

General information: The diffraction data were measured at 100 K on a Bruker D8 VENTURE diffractometer equipped with a microfocus Mo-target X-ray tube (λ = 0.71073 Å) and PHOTON 100 CMOS detector. Data were collected using ϕ and ω scans to survey a hemisphere of reciprocal space. Data reduction and integration were performed with the Bruker APEX3 software package (Bruker AXS, version 2017.3-0, 2018). The crystal appeared to be a 2-component twin. Unit cell parameters for each component were identified using Cell_Now. Data were scaled and corrected for absorption effects using the multi-scan procedure as implemented in TWINABS (Bruker AXS, version 2012/1, Krause, Herbst-Irmer, Sheldrick & Stalke, *J. Appl. Cryst.* **2015**, *48*, 3-10). The structure was solved by SHELXT (Version 2014/5: Sheldrick, G. M. *Acta Crystallogr*. **2015**, ***A71***, 3-8) and refined by a full-matrix least-squares procedure using OLEX2 (O. V. Dolomanov, L. J. Bourhis, R. J. Gildea, J. A. K. Howard and H. Puschmann. J. Appl. Crystallogr. **2009**, *42*, 339-341) (XL refinement program version 2018/3, *Sheldrick, G. M. Acta Crystallogr.* **2015**, *C71*, 3-8). Crystallographic data and details of the data collection and structure refinement are listed in Table S35.

Specific details for structure refinement: The structure was first solved and refined using HKLF 4 file format. The final refinement cycles were carried out with HKLF 5. Refined fractional volume contribution for the second twin component is 0.46. All atoms were refined with anisotropic thermal parameters. Hydrogen atoms were included in idealized positions for structure factor calculations except H-atoms attached to nitrogen atoms. These hydrogen atoms (H1 and H3) were located in the difference Fourier map and allowed to be refined at 0.88 Å within a default 0.02 Å standard deviation with their thermal parameters being constrained to be 1.2 times of the Ueq value of the N atoms. All structures are drawn with thermal ellipsoids at 40% probability.

Table S35 Crystal data and structure refinement for 0872_tes_btm.

Identification code	0872_tes_btm
Empirical formula	$C_{44.5}H_{45}ClN_4O_6$
Formula weight	767.29
Temperature/K	100(2)
Crystal system	triclinic
Space group	P-1
a/Å	12.3351(7)
b/Å	13.1435(7)
c/Å	13.7758(7)
α/°	93.491(2)
β/°	101.268(2)
γ/°	116.875(2)
Volume/Å^3	1925.16(18)
Z	2
ρ_{calc}g/cm^3	1.324
μ/mm^{-1}	0.155
F(000)	810.0
Crystal size/mm^3	0.32 × 0.19 × 0.14
Radiation	MoKα (λ = 0.71073)
2Θ range for data collection/°	4.256 to 52.882
Index ranges	$-15 \le h \le 14$, $-16 \le k \le 16$, $0 \le l \le 17$
Reflections collected	7910
Independent reflections	7910 [R_{int} = 0.0610, R_{sigma} = 0.0562]
Data/restraints/parameters	7910/3/522
Goodness-of-fit on F^2	1.090
Final R indexes [I>=2σ (I)]	R_1 = 0.0559, wR_2 = 0.1025
Final R indexes [all data]	R_1 = 0.0856, wR_2 = 0.1125
Largest diff. peak/hole / e Å^{-3}	0.36/-0.59

$R_{int} = \Sigma | F_o^2 - \langle F_o^2 \rangle | / \Sigma | F_o^2|$

$R1 = \Sigma || F_o| - | F_c|| / \Sigma | F_o|$

$wR2 = [\Sigma [w (F_o^2 - F_c^2)^2] / \Sigma [w (F_o^2)^2]]^{1/2}$

Goodness-of-fit = $[\Sigma [w (F_o^2 - F_c^2)^2] / (n-p)]^{1/2}$

n: number of independent reflections; p: number of refined parameters

Figure S27.

Independent unit

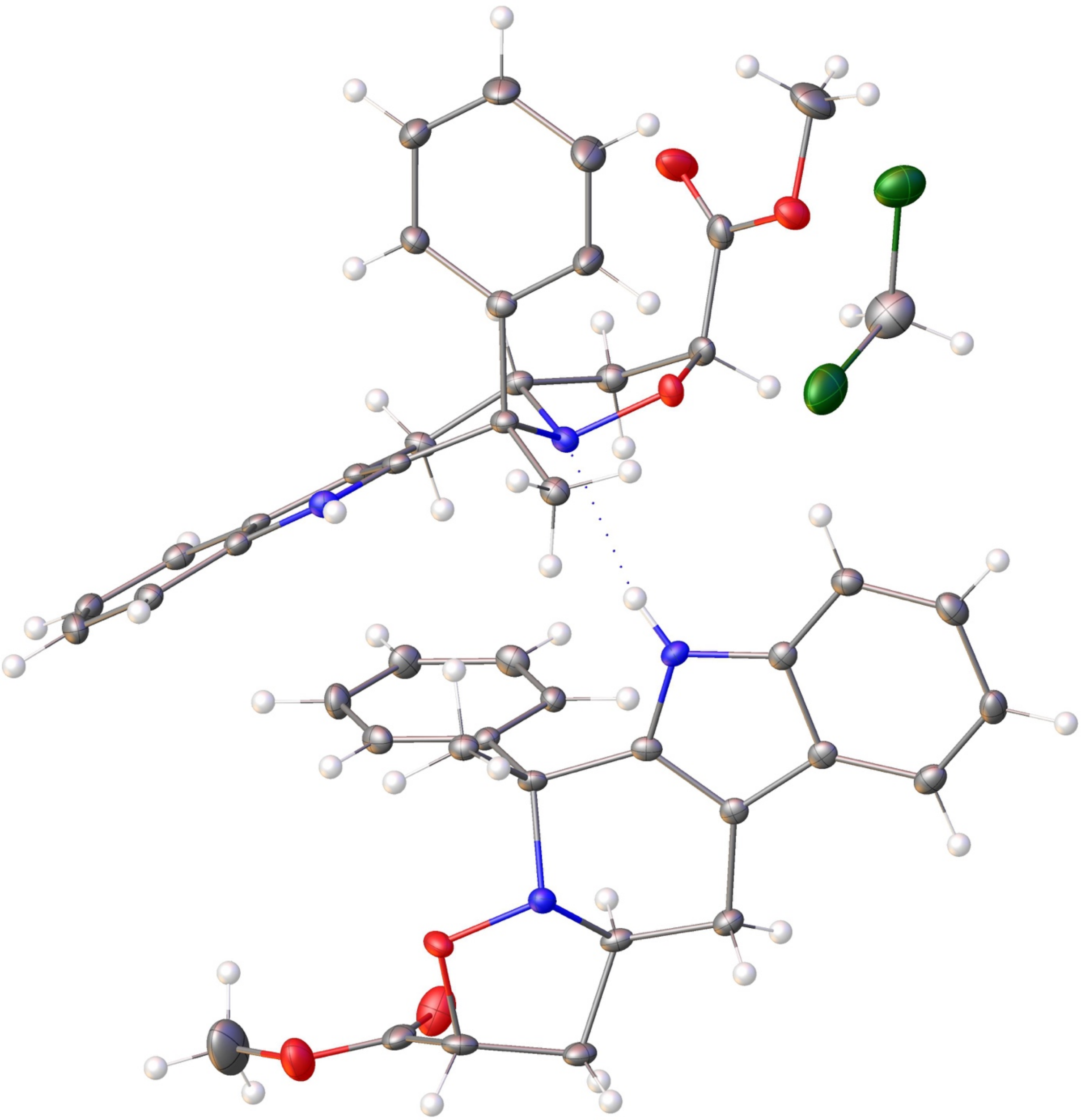

Figure S28.

Molecule 1

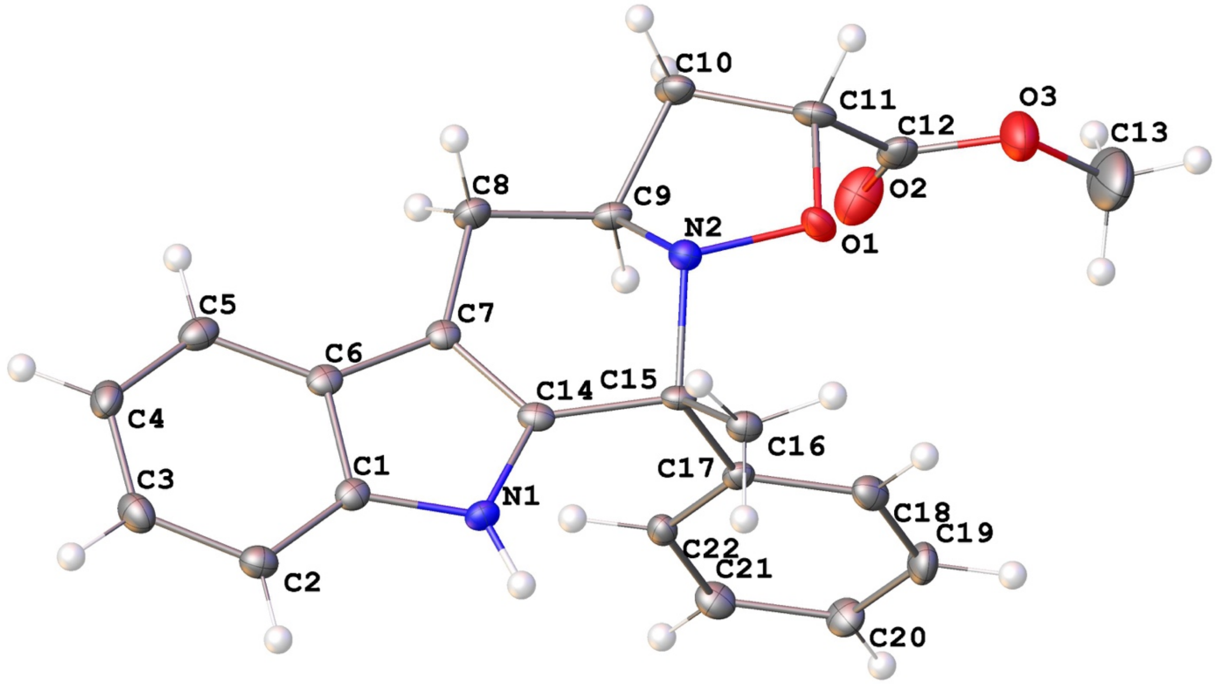

Figure S29.

Molecule 2

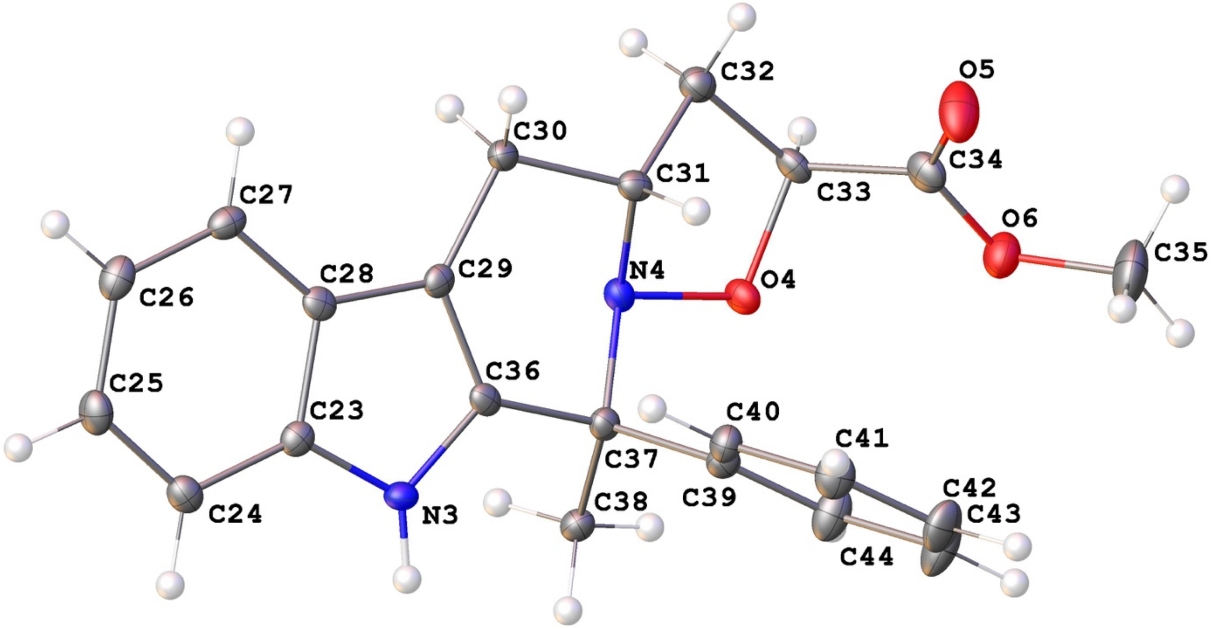

Figure S30.

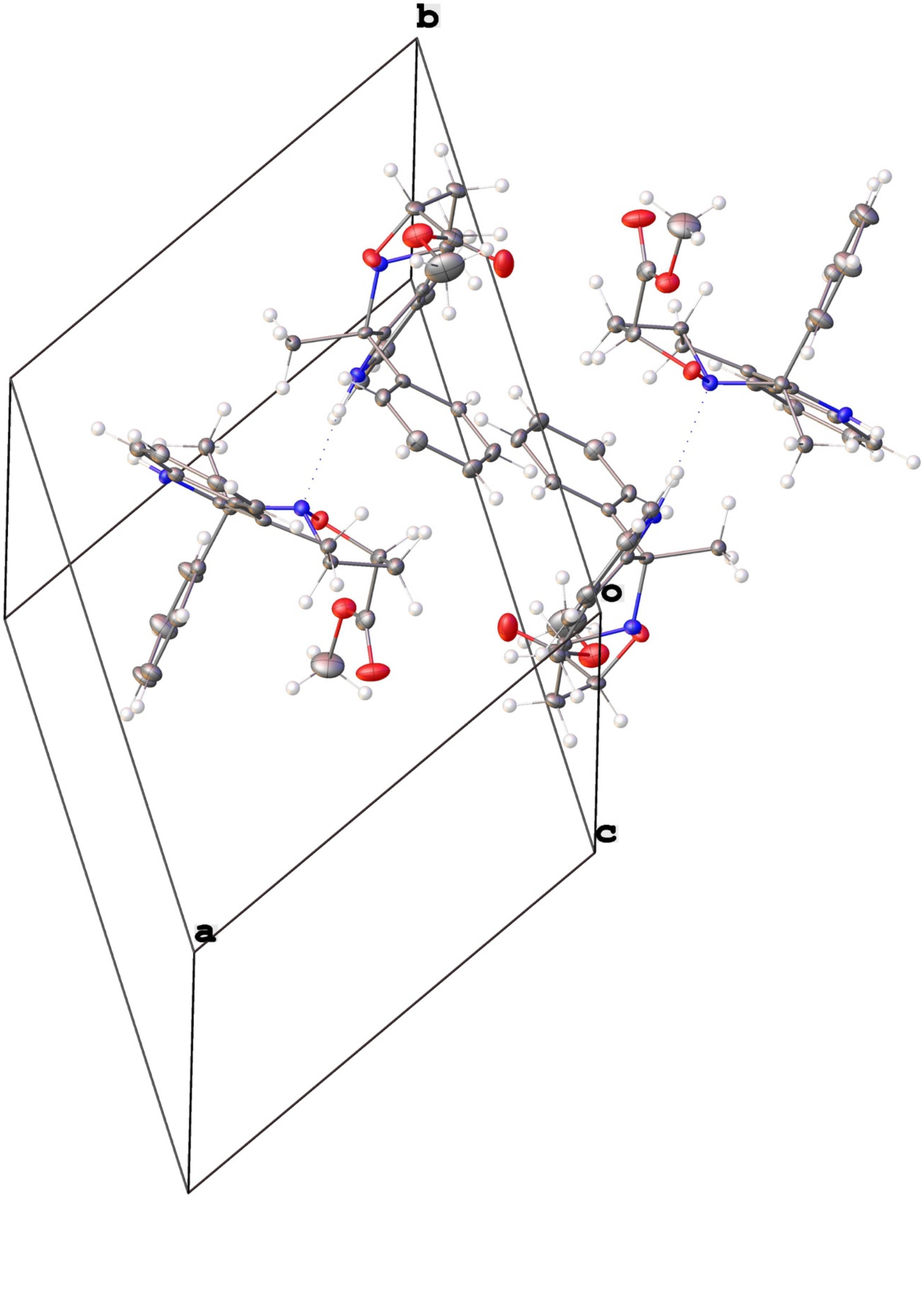

Table S36 Fractional Atomic Coordinates (×10^4) and Equivalent Isotropic Displacement Parameters (Å^2×10^3) for 0872_tes_btm. U_{eq} is defined as 1/3 of of the trace of the orthogonalised U_{IJ} tensor.

Atom	*x*	*y*	*z*	U(eq)
O1	1826.9(16)	8330.2(15)	2569.9(12)	19.9(4)
O2	-455.0(19)	6246.1(18)	1060.8(16)	36.8(5)
O3	1138(2)	7424.9(19)	454.0(16)	38.3(6)
N1	2257(2)	8030.0(18)	6110.3(16)	15.7(5)
N2	1564(2)	8483.7(18)	3551.4(15)	16.6(5)
C1	1660(2)	8270(2)	6768.9(19)	15.8(5)
C2	1960(2)	8460(2)	7812.4(19)	19.5(6)
C3	1235(3)	8768(2)	8280(2)	23.3(6)
C4	211(3)	8867(2)	7725(2)	21.8(6)
C5	-104(3)	8656(2)	6693(2)	20.3(6)
C6	624(2)	8354(2)	6191.8(19)	15.7(5)
C7	630(2)	8149(2)	5162.2(18)	14.3(5)
C8	-183(2)	8223(2)	4236.9(19)	18.3(6)
C9	178(2)	7865(2)	3338.8(18)	16.4(5)
C10	-200(3)	8215(2)	2348.7(19)	20.1(6)
C11	794(3)	8270(2)	1818.5(19)	20.5(6)
C12	387(3)	7185(3)	1070(2)	23.9(6)
C13	924(4)	6447(3)	-251(3)	59.6(11)
C14	1642(2)	7986(2)	5144.8(18)	14.6(5)
C15	2151(2)	7923(2)	4245.3(18)	14.2(5)
C16	3572(2)	8693(2)	4507.9(19)	16.6(5)
C17	1790(2)	6679(2)	3795.2(18)	14.1(5)
C18	2392(2)	6463(2)	3113.9(19)	19.1(6)
C19	2059(3)	5342(2)	2718(2)	22.8(6)
C20	1115(3)	4411(2)	2985(2)	21.6(6)
C21	514(3)	4616(2)	3653(2)	20.8(6)
C22	843(2)	5736(2)	4052.4(18)	15.1(5)
O4	3622.3(16)	7030.0(15)	7834.4(13)	18.9(4)
O5	3400(2)	4595.2(19)	8649.1(15)	38.3(6)
O6	3420.7(19)	6090.3(17)	9530.0(14)	27.5(5)
N3	6639(2)	8748.5(18)	5824.8(16)	16.0(5)
N4	4047.2(19)	7103.7(18)	6898.3(15)	16.1(5)
C23	6605(2)	8349(2)	4862.2(18)	16.2(5)
C24	7292(2)	8928(2)	4199.8(19)	18.8(6)
C25	7046(3)	8310(2)	3271(2)	21.8(6)
C26	6142(3)	7138(2)	2995(2)	21.1(6)
C27	5439(2)	6574(2)	3641.6(19)	18.2(6)
C28	5657(2)	7184(2)	4595.5(18)	15.2(5)

C29	5100(2)	6903(2)	5424.2(18)	14.8(5)
C30	3982(2)	5827(2)	5516.9(18)	16.8(6)
C31	3868(2)	5926(2)	6589.6(19)	16.2(6)
C32	2604(2)	5184(2)	6790(2)	19.9(6)
C33	2717(2)	5832(2)	7785.4(19)	18.0(6)
C34	3216(3)	5413(2)	8687(2)	23.0(6)
C35	3948(4)	5795(3)	10442(2)	45.2(9)
C36	5713(2)	7872(2)	6151.7(18)	14.8(5)
C37	5400(2)	8016(2)	7138.0(18)	15.4(5)
C38	5439(2)	9186(2)	7350.9(19)	17.8(6)
C39	6239(2)	7839(2)	8022.2(19)	17.5(6)
C40	6972(2)	7328(2)	7869.8(19)	18.9(6)
C41	7698(3)	7152(2)	8678(2)	24.5(6)
C42	7700(3)	7478(3)	9641(2)	29.3(7)
C43	6989(3)	8003(3)	9808(2)	34.8(8)
C44	6259(3)	8177(3)	9001(2)	27.5(7)
Cl1	5000	10000	10000	52.0(4)
Cl2	5009.5(18)	8656.0(15)	11478.5(13)	46.3(4)
C45	4131(6)	8781(5)	10417(4)	45.9(18)

Table S37 Anisotropic Displacement Parameters (Å^2×10^3) for 0872_tes_btm. The Anisotropic displacement factor exponent takes the form: $-2\pi^2[h^2a^{*2}U_{11}+2hka^*b^*U_{12}+\ldots]$.

Atom	U_{11}	U_{22}	U_{33}	U_{23}	U_{13}	U_{12}
O1	18.5(10)	26.9(10)	14.3(9)	6.9(8)	3.5(8)	10.7(8)
O2	26.5(12)	29.4(12)	46.1(14)	-8.0(10)	6.9(10)	8.7(10)
O3	55.8(15)	39.7(13)	33.9(12)	10.8(10)	24.3(11)	29.1(12)
N1	13.5(11)	15.5(11)	18.3(12)	2.5(9)	2.1(9)	7.9(9)
N2	18.8(12)	17.3(11)	15.6(11)	4.0(9)	4.4(9)	9.9(9)
C1	14.6(13)	10.8(12)	20.0(14)	2.6(10)	4.2(11)	4.3(11)
C2	19.2(14)	18.1(14)	20.6(14)	5.6(11)	3.9(11)	8.4(11)
C3	30.9(16)	20.8(15)	17.5(14)	3.1(11)	7.4(12)	11.0(13)
C4	27.5(15)	18.2(14)	25.5(15)	5.2(11)	13.2(13)	12.9(12)
C5	17.9(14)	15.4(13)	28.0(16)	4.9(11)	5.1(12)	8.2(11)
C6	15.1(13)	8.8(12)	20.6(13)	2.9(10)	4.1(11)	3.5(10)
C7	14.2(13)	8.4(12)	17.8(13)	2.3(10)	3.3(10)	3.4(10)
C8	16.6(14)	18.5(13)	20.6(14)	3.0(11)	2.4(11)	9.9(11)
C9	14.8(13)	13.1(13)	18.9(13)	2.0(10)	-1.4(10)	6.9(11)
C10	23.1(15)	23.0(14)	15.9(13)	2.3(11)	-1.0(11)	14.6(12)
C11	23.2(15)	20.2(14)	17.8(14)	6.8(11)	-0.5(11)	11.9(12)
C12	24.3(15)	33.1(17)	19.9(14)	4.3(12)	0.0(12)	20.5(14)

C13	97(3)	57(2)	50(2)	6.9(19)	38(2)	50(2)
C14	14.9(13)	8.8(12)	16.0(13)	1.6(10)	0.4(10)	3.4(10)
C15	15.4(13)	11.1(12)	15.8(12)	4.6(10)	1.8(10)	6.6(10)
C16	14.9(13)	15.5(13)	17.2(13)	2.9(10)	1.5(11)	6.4(11)
C17	13.4(13)	15.6(13)	14.0(13)	2.4(10)	1.3(10)	8.3(11)
C18	15.5(14)	18.6(13)	21.0(14)	6.7(11)	5.2(11)	5.6(11)
C19	27.2(16)	24.9(15)	19.6(14)	1.1(12)	10.3(12)	13.8(13)
C20	27.1(16)	14.8(13)	22.2(15)	-0.8(11)	5.9(12)	9.8(12)
C21	20.0(15)	14.4(13)	22.4(14)	4.0(11)	4.4(12)	3.8(11)
C22	15.3(13)	17.4(13)	13.0(13)	2.7(10)	3.4(10)	8.2(11)
O4	20.0(10)	17.4(9)	20.3(10)	4.6(8)	10.2(8)	7.5(8)
O5	67.8(17)	39.0(13)	27.8(12)	12.4(10)	16.3(11)	39.3(13)
O6	36.3(12)	28.7(11)	19.7(10)	4.4(9)	6.7(9)	17.5(10)
N3	16.0(12)	11.7(11)	17.2(12)	1.3(9)	1.8(9)	5.2(9)
N4	15.4(11)	16.9(11)	15.8(11)	5.2(9)	4.5(9)	7.1(9)
C23	16.2(14)	18.4(13)	16.7(13)	2.7(11)	2.3(11)	11.1(11)
C24	15.5(14)	20.5(13)	22.0(14)	6.7(11)	5.2(11)	9.4(11)
C25	21.6(15)	29.1(16)	20.3(14)	8.8(12)	10.0(12)	14.3(13)
C26	24.5(15)	28.4(15)	17.3(14)	3.0(11)	4.7(12)	18.7(13)
C27	18.0(14)	16.9(13)	19.1(14)	-0.3(11)	-0.5(11)	10.2(11)
C28	14.6(13)	15.0(13)	19.3(14)	5.4(11)	2.4(11)	10.3(11)
C29	15.5(13)	16.2(13)	14.3(13)	3.4(10)	0.6(10)	10.1(11)
C30	17.7(14)	14.5(13)	15.2(13)	3.9(10)	1.6(11)	6.0(11)
C31	14.9(14)	13.6(13)	19.3(14)	3.7(10)	1.8(11)	6.9(11)
C32	18.8(14)	18.6(14)	21.2(14)	4.5(11)	3.9(11)	8.3(12)
C33	15.7(13)	16.3(13)	23.6(14)	8.5(11)	9.3(11)	6.6(11)
C34	23.0(15)	23.4(15)	25.4(15)	7.4(12)	13.4(12)	10.1(13)
C35	65(3)	60(2)	19.6(16)	13.2(16)	10.4(16)	37(2)
C36	12.6(13)	15.2(13)	17.6(13)	6.2(11)	1.6(10)	8.0(11)
C37	14.1(13)	16.9(13)	15.0(13)	4.1(10)	1.8(10)	7.6(11)
C38	16.7(14)	17.6(13)	16.4(13)	1.7(11)	2.2(11)	6.8(11)
C39	15.0(13)	17.2(13)	17.3(14)	3.1(11)	1.7(11)	6.1(11)
C40	20.3(14)	21.8(14)	15.2(13)	3.5(11)	4.1(11)	10.4(12)
C41	23.9(15)	31.1(16)	24.6(15)	6.2(13)	5.7(12)	18.1(13)
C42	27.3(16)	44.3(19)	19.6(15)	6.2(13)	-1.2(13)	22.5(15)
C43	38.8(19)	56(2)	16.4(15)	2.3(14)	3.7(13)	30.3(17)
C44	29.2(16)	43.9(18)	19.5(15)	4.2(13)	4.6(12)	26.4(15)
Cl1	59.6(9)	67.6(9)	36.9(7)	-10.1(6)	5.9(6)	41.7(7)
Cl2	56.9(11)	39.5(10)	46.3(10)	0.8(8)	3.3(8)	30.6(9)
C45	35(4)	38(4)	54(5)	-1(3)	2(3)	13(3)

Table S38 Bond Lengths for 0872_tes_btm.

Atom	Atom	Length/Å	Atom	Atom	Length/Å
O1	N2	1.474(3)	O5	C34	1.196(3)
O1	C11	1.443(3)	O6	C34	1.326(3)
O2	C12	1.199(3)	O6	C35	1.456(3)
O3	C12	1.324(3)	N3	C23	1.382(3)
O3	C13	1.451(4)	N3	C36	1.381(3)
N1	C1	1.379(3)	N4	C31	1.483(3)
N1	C14	1.380(3)	N4	C37	1.503(3)
N2	C9	1.479(3)	C23	C24	1.391(4)
N2	C15	1.509(3)	C23	C28	1.411(4)
C1	C2	1.389(4)	C24	C25	1.374(4)
C1	C6	1.421(4)	C25	C26	1.404(4)
C2	C3	1.378(4)	C26	C27	1.377(4)
C3	C4	1.408(4)	C27	C28	1.407(4)
C4	C5	1.373(4)	C28	C29	1.429(4)
C5	C6	1.404(4)	C29	C30	1.499(3)
C6	C7	1.430(4)	C29	C36	1.370(3)
C7	C8	1.498(3)	C30	C31	1.515(3)
C7	C14	1.363(3)	C31	C32	1.512(4)
C8	C9	1.511(4)	C32	C33	1.516(4)
C9	C10	1.520(3)	C33	C34	1.522(4)
C10	C11	1.523(4)	C36	C37	1.507(4)
C11	C12	1.523(4)	C37	C38	1.525(3)
C14	C15	1.508(4)	C37	C39	1.539(3)
C15	C16	1.527(3)	C39	C40	1.385(4)
C15	C17	1.539(3)	C39	C44	1.385(4)
C17	C18	1.394(4)	C40	C41	1.388(4)
C17	C22	1.390(4)	C41	C42	1.368(4)
C18	C19	1.380(4)	C42	C43	1.380(4)
C19	C20	1.387(4)	C43	C44	1.387(4)
C20	C21	1.374(4)	Cl1	C45	1.687(5)
C21	C22	1.383(4)	Cl1	C45[1]	1.687(5)
O4	N4	1.477(3)	Cl2	C45	1.706(5)
O4	C33	1.449(3)			

[1]1-X,2-Y,2-Z

Table S39 Bond Angles for 0872_tes_btm.

Atom	Atom	Atom	Angle/°	Atom	Atom	Atom	Angle/°
C11	O1	N2	106.59(17)	C34	O6	C35	114.8(2)

C12	O3	C13	115.7(3)	C36	N3	C23	108.6(2)
C1	N1	C14	108.6(2)	O4	N4	C31	103.96(17)
O1	N2	C9	104.21(18)	O4	N4	C37	108.02(17)
O1	N2	C15	107.07(17)	C31	N4	C37	112.77(19)
C9	N2	C15	111.75(19)	N3	C23	C24	130.0(2)
N1	C1	C2	130.4(2)	N3	C23	C28	107.7(2)
N1	C1	C6	107.7(2)	C24	C23	C28	122.3(2)
C2	C1	C6	121.9(2)	C25	C24	C23	117.4(2)
C3	C2	C1	117.7(2)	C24	C25	C26	121.7(2)
C2	C3	C4	121.5(2)	C27	C26	C25	120.8(2)
C5	C4	C3	121.0(3)	C26	C27	C28	119.0(2)
C4	C5	C6	119.1(2)	C23	C28	C29	107.0(2)
C1	C6	C7	106.4(2)	C27	C28	C23	118.8(2)
C5	C6	C1	118.9(2)	C27	C28	C29	134.3(2)
C5	C6	C7	134.6(2)	C28	C29	C30	129.9(2)
C6	C7	C8	129.5(2)	C36	C29	C28	107.1(2)
C14	C7	C6	107.4(2)	C36	C29	C30	122.8(2)
C14	C7	C8	122.7(2)	C29	C30	C31	108.4(2)
C7	C8	C9	107.8(2)	N4	C31	C30	107.9(2)
N2	C9	C8	107.5(2)	N4	C31	C32	101.1(2)
N2	C9	C10	101.9(2)	C32	C31	C30	118.0(2)
C8	C9	C10	117.9(2)	C31	C32	C33	101.8(2)
C9	C10	C11	102.6(2)	O4	C33	C32	106.50(19)
O1	C11	C10	107.4(2)	O4	C33	C34	107.2(2)
O1	C11	C12	103.9(2)	C32	C33	C34	113.0(2)
C12	C11	C10	114.4(2)	O5	C34	O6	124.5(3)
O2	C12	O3	124.9(3)	O5	C34	C33	125.3(3)
O2	C12	C11	125.3(3)	O6	C34	C33	110.1(2)
O3	C12	C11	109.7(2)	N3	C36	C37	123.9(2)
N1	C14	C15	123.0(2)	C29	C36	N3	109.6(2)
C7	C14	N1	109.9(2)	C29	C36	C37	126.4(2)
C7	C14	C15	126.6(2)	N4	C37	C36	100.79(19)
N2	C15	C16	107.72(19)	N4	C37	C38	107.4(2)
N2	C15	C17	113.22(19)	N4	C37	C39	113.4(2)
C14	C15	N2	100.80(19)	C36	C37	C38	110.7(2)
C14	C15	C16	110.5(2)	C36	C37	C39	112.9(2)
C14	C15	C17	113.2(2)	C38	C37	C39	111.1(2)
C16	C15	C17	111.0(2)	C40	C39	C37	121.7(2)
C18	C17	C15	121.2(2)	C40	C39	C44	118.3(2)
C22	C17	C15	120.7(2)	C44	C39	C37	120.0(2)
C22	C17	C18	118.1(2)	C39	C40	C41	120.7(2)
C19	C18	C17	120.5(2)	C42	C41	C40	120.4(3)

C18	C19	C20	120.7(2)	C41	C42	C43	119.7(3)
C21	C20	C19	119.2(2)	C42	C43	C44	120.0(3)
C20	C21	C22	120.4(2)	C39	C44	C43	120.9(3)
C21	C22	C17	121.0(2)	C45	Cl1	C45[1]	180.0(3)
C33	O4	N4	107.03(17)	Cl1	C45	Cl2	109.9(4)

[1]1-X,2-Y,2-Z
